# Comparison of quantity, quality and antibacterial activity of essential oil *Mentha longifolia* (L.) L. under different traditional and modern extraction methods

**DOI:** 10.1371/journal.pone.0301558

**Published:** 2024-07-10

**Authors:** Masoumeh Karimnejad, Mansureh Ghavam

**Affiliations:** Department of Nature Engineering, Faculty of Natural Resources and Earth Sciences, University of Kashan, Kashan, Iran; National Institute of Agricultural Research - INRA, MOROCCO

## Abstract

Extraction is the first and most important step in obtaining the effective ingredients of medicinal plants. *Mentha longifolia* (L.) L. is of considerable economic importance as a natural raw material for the food and pharmaceutical industries. Since the effect of different extraction methods (traditional and modern methods) on the quantity, quality and antimicrobial activity of the essential oil of this plant has not been done simultaneously; the present study was designed for the first time with the aim of identifying the best extraction method in terms of these features. For this purpose, extracting the essential oil of *M*. *longifolia* with the methods of hydrodistillation with Clevenger device (HDC), steam distillation with Kaiser device (SDK), simultaneous distillation with a solvent (SDE), hydrodistillation with microwave device (HDM), pretreatment of ultrasonic waves and Clevenger (U+HDC) and supercritical fluid (SF) were performed. Chemical compounds were identified by gas chromatography coupled with mass spectrometer (GC-MS). Antimicrobial activity of essential oils against various clinical microbial strains was evaluated by agar diffusion method and determination of the minimum inhibitory concentration and minimum bactericidal concentration (MIC and MBC). The results showed that the highest and lowest yields of *M*. *longifolia* leaf essential oil belonged to HDC (1.6083%) and HDM (0.3416%). The highest number of compounds belonged to SDK essential oil and was equal to 72 compounds (with a relative percentage of 87.13%) and the lowest number of compounds was related to the SF essential oil sample (7 compounds with a relative percentage of 100%). Piperitenone (25.2–41.38%), piperitenone oxide (22.02–0%), pulegone (10.81–0%) and 1,8-cineole (5–35.0%) are the dominant and main components of *M*. *longifolia* essential oil were subjected to different extraction methods. Antimicrobial activity results showed that the lowest MIC value belonged to essential oils extracted by HDM, SDK, SDE and U+HDC methods with a value of 1000 μg/mL was observed against Gram-negative bacteria *Shigella dysenteriae*, which was 5 times weaker than rifampin and 7 times weaker than gentamicin. Therefore, it can be concluded that in terms of efficiency of the HDC method, in terms of the percentage of compounds of the HDM method, and in terms of microbial activity, the SDK, HDM and U+HDC methods performed better.

## 1-Introduction

In recent years, the interest and demand for using natural products and herbal medicines instead of artificial substances has increased in the world [1]. Among these biological compounds are essential oils, which are also called volatile oils [2]. Essential oils are concentrated hydrophobic liquids that contain a diverse group of more than 20 volatile and non-volatile aromatic flavoring compounds. These compounds are secondary metabolites obtained from different parts of plants [3].

According to the European Pharmacopoeia, an essential oil is an aromatic product, usually of complex composition, obtained from a crude plant material by water distillation, steam distillation, or a mechanical process without heat [4, 5]. Extraction is an essential step to obtain essential oils from natural plants [6]. Extraction is the first and most important step in obtaining the effective ingredients of medicinal plants. Choosing the right method for extraction has a direct effect on the quantity and quality of the resulting compounds. In recent decades, due to the increasing use of medicinal plants in various industries, choosing effective methods for extracting these compounds and increasing the extraction efficiency is one of the challenges facing researchers [7].

Various conventional methods such as water distillation, steam distillation and cold pressing have been used to extract essential oils from aromatic plants. However, the use of these methods for the sequential determination of essential oil composition has been controversial because long extraction times at high temperatures may cause changes in essential oil composition or degradation of unsaturated or esterified compounds and loss of highly volatile compounds [8]. In order to reduce the extraction time and improve the quality of essential oils, new extraction techniques have been developed, such as microwave-assisted extraction, pressurized solvent extraction, supercritical fluid extraction, and ultrasound-assisted extraction. In the face of all these innovative methods of essential oil extraction, choosing the most efficient method is relevant for better optimization of production time, performance and cost [9].

The genus *Mentha* with the Persian name "Nana" is a perennial, aromatic and medicinal plant from the lamiaceae family, which has a wide global distribution. This genus includes 25–30 known species [10]. Medicinal and therapeutic use of *Mentha* species has been reported since the Ming Dynasty in China [11]. *Mentha* has become an official item of Materia Medical in the London Pharmacopoeia [12]. Studies indicate that *Mentha* species have antimicrobial activity against various bacterial and fungal strains [13, 14].

This genus has 7 aromatic species in Iran [15]. *Mentha longifolia* [L.) L., with the Persian name "Pune", is a perennial herbaceous plant with a stem with an almost cylindrical appearance, 40–120 cm high, and grows in wet plains and at the edge of water, even in water. It is native to temperate and Mediterranean regions of Eurasia and Africa [16]. The leaves of this species are very diverse in terms of size, shape and hairy covering and are completely without petioles or the lower leaves of the stem have short petioles [17]. Commonly used as a natural remedy for several disorders in the traditional medicine of different nations including Iraq, Iran, Pakistan, Turkey and Arab countries, especially digestive diseases such as gas, indigestion, intestinal colic, intestinal ulcer, anti-diarrhea, intestinal spasm, stomach problems and ulcerative colitis [18], respiratory disorders including asthma, colds, bronchitis, tuberculosis, sinusitis and cough, it is used as an anti-hemolytic, anti-inflammatory, as well as the treatment of headache caused by lung infection and nausea [19].

Ethnobotanical studies in Iran indicate that the people of Zagheh and Biranshahr areas of Lorestan province call this plant "Pine" and use it as a carminative, digestive pain reliever, diarrhea treatment, and laxative [20]. The people of Rostam city of Fars province know this plant by the name "Pidan" and use the whole body of this plant for stomach discomfort, mouth freshener, laxative, diuretic, diarrhea, flatulence [21]. The people of Zabul city, Sistan and Baluchistan province, use "Pudneh" leaf decoction for appetite and diarrhea [22]. Native communities of Ahar city, East Azerbaijan use the aerial parts of "Nana" for digestive system problems, anti-diarrhea and heartache [23]. People of Baft city of Kerman province use the flowers and leaves of "Pudneh" for anti-flatulence and treatment of stomach pains [24]. The people of Abhar city of Zanjan province know this plant by the names of yarpiz or yarpiz tea, and the leaves, flowering branches, and aerial parts of the plant are edible, infused and boiled for anti-flatulence, anti-cough, anti-convulsant, anti-inflammatory, antiseptic, abortifacient, They are used as anti-asthma, anti-spasm, headache relief, anti-anxiety, treatment of digestive disorders [25].

In past studies, the main compounds of *M*. *longifolia* essential oil mainly oxygenated monoterpenes such as menthol, 1,8-cineole, limonene, α-terpineol, carvone, piperitenone, piperitone oxide, pulegone and menthone [26–29] have been reported. Previous research has shown that the essential oil of this plant is a strong scavenger of free radicals [30, 31], an effective antimicrobial agent against a wide range of pathogenic microbes such as *Staphylococcus aureus*, *Escherichia coli*, *Bacillus subtilis*, *Aspergillus flavus*, *Alternaria solani*, *Aspergillus niger*, *Alternaria altarnata*, *Rhizopus solani*, *Fusarium solani*, *and،Salmonella Typhimirium، Pseudomonas aeruginosa*, *Candida albicans*, and *Listeria monocytogenes* [32–34], and cytotoxic activity [35].

To the best of our knowledge, a simultaneous study in terms of the influence of different classical and modern extraction methods on the quantity, quality and biological activities of *M*. *longifolia* essential oil has not been done simultaneously. Considering the pharmacology and traditional uses of *M*. *longifolia* and the importance of the impact of extraction methods on the efficiency and chemical composition of the essential oil, the present study was designed with the aim of identifying the best extraction method to obtain *M*. *longifolia* essential oil with the highest quantity and quality for the first time in Iran.

## 2-Materials and methods

### 2-1-Preparation of plant materials

*M*. *longifolia* dry leaf was obtained from a herbalist’s shop in Tehran, Iran, and was identified and verified by Mansureh Ghavam, Faculty of Natural Resources and Earth Sciences, University of Kashan, Kashan, Iran. Plant materials were powdered by a small electric grinder before each extraction method.

### 2-2-Extraction and separation of essential oil

#### 2-2-1-Hydrodistillation with Clevenger device (HDC)

120 g of powdered plant material was poured into a 2000 mL flask. After that, two-thirds of the volume of the balloon was filled with distilled water and it was connected to the Clevenger device according to the European Pharmacopoeia equipped with a condenser (made in Germany). After boiling the material for 5 h, the extracted essential oil was collected and dehydrated by sodium sulfate (Merck, Germany).

#### 2-2-2- Steam distillation with Kaiser device (SDK)

In the direct steam distillation method, the plant is not placed in the distillation vessel. About 1000 mL of water was poured into the 2000 mL flask. 210 g of powdered plant material was placed in a special chamber for the plant. In this device, there is a mesh screen between the water source and the plant chamber, and the generated steam enters the plant chamber after traveling from the water source and then enters the refrigerant. After boiling the material for 5 h, the extracted essential oil was collected and dehydrated by sodium sulfate.

#### 2-2-3-Simultaneous distillation with a solvent (SDE)

202 g of powdered plant material was transferred to a 2000 mL flask and 1000 mL of distilled water was added to it so that the total plant sample and distilled water occupied two thirds of the volume of the flask. 40 mL of normal organic solvent pentane (Merck, Germany) was poured into a 100 mL flask. The SDE device (Ashke Shishe, Iran) was set up with two flasksand Essential oil extraction was done for 5 h. After the extraction time, the flask containing the pentane solvent and the essential oil dissolved in the solvent was separated from the device, and after dehydrating with sodium sulfate and evaporating the pentane solvent, pure essential oil was obtained [36].

#### 2-2-4-Hydrodistillation with microwave device (HDM)

Hydraulic distillation with the help of solvent free microwave extraction Labstation (Dry Dist model, Milestone company, Italy), is directly connected to a Clevenger type extractor and a cooling system to continuously condense the distillate. The excess concentrated water is returned to the extraction balloon in order to return the water to the plant material [9]. In this method, 202 g of powdered plant material were transferred to the 2000 mL chamber of the machine and soaked with 500 mL of distilled water in one h. Then the flask containing plants and water was placed in the desired location in the microwave cabin. According to the schedule, essential oil extraction was done in 35 min with 800 w of power. Then, the essential oil dissolved in the pentane solvent was collected through the bottom valve in the intended container and dehydrated by sodium sulfate.

#### 2-2-5-Pretreatment of ultrasonic waves and Clevenger (U+HDC)

In this method, 70 g of powdered plant material was placed inside the ultrasonic probe (Dr. Hilscher model) with a power of 400 amps and a temperature of 45°C for 15 min. Then the solution containing essential oil, plant and distilled water was added to the flask of the Clevenger and essential oil was extracted for 5 h [37]. Then pure essential oil was obtained after dehydrating with sodium sulfate.

#### 2-2-6-Supercritical fluid (SF)

In this method, a supercritical fluid device (discontinuous cylindrical reactor with a volume of 60 L made of 316 stainless steel with a loading capacity between 1 and 10 g with a maximum temperature of 400°C and a maximum pressure of 200 bar, made in Iran) was used. 1 g of powdered plant material was poured into 15 mL of normal hexane (Merck, Germany). Then the essential oil was extracted under the conditions of temperature of 230°C and 33 bar pressure for 15 min. The obtained organic phase was placed in a centrifuge [Herolab/Higen, Germany) at 2000 rpm for 10 min to separate the essential oil from hexane [38].

### 2-3-Determining the quantity of essential oils (yield)

Essential oil extraction by all methods was repeated three times. The quantity (yield) of essential oils was calculated in terms of dry weight w/w using Eq 1 and was reported as mean ± standard deviation [39]

100x(weightofdryplant/weightofessentialoil)=yieldofessentialoil (1)

Pure essential oils were stored in a dark, airtight glass vial at 4°C for chemical and antimicrobial analyses.

### 2-4-Identification of chemical compounds of essential oils

Identification of chemical compounds of essential oil was determined using GC-MS machine of University of Kashan. Chromatograph model 6890 coupled with mass spectrometer model 5973N made by Agilent, USA, with HP-5MS capillary column with 5% methylphenylsiloxane stationary phase (Length 30 m, Internal Diameter 0.25 mm, Layer Static Thickness 0.25 μm) and ionization energy is 70 eV. Temperature programming for the analysis started the oven temperature at 60°C and then increased to 246°C at a rate of 3°C/min. The injected sample volume was 1 microliter with a 1.50 split, the temperature of the injector and detector was 250°C, and the carrier gas was helium with a flow rate of 1.5 mL/min. The identification of the compounds in the essential oil was done according to the recommendations of the device library (Wiley-14 and NIST-14 Mass Spectral Library) and the study of the mass spectra and the comparison of these spectra and their inhibition index with the standard compounds available in the references. In the calculation of the inhibition index of essential oil compounds, normal alkane series injection (C8-C20) was also performed according to the conditions of essential oil injection to the GC-MS device [40].

### 2-5-Determination of antimicrobial activity

#### 2-5-1-Preparation and cultivation of microbial strains

Clinical strains including Gram-positive *Staphylococcus aureus* and Gram-negative bacteria *Shigella dysenteriae* and *Escherichia coli* were obtained from the University of Medical Sciences. Bacterial strains were incubated in nutrient agar medium at 37°C for 24 h in an incubator.

#### 2-5-2-Agar diffusion method

Agar diffusion method was performed according to CLSI standards [41]. For this purpose, plates containing Mueller Hinton agar culture medium were prepared. Wells with a diameter of 0.6 mm were created on the culture medium, then culture of 100 μL of bacterial suspensions with half McFarland turbidity were cultured in uniform conditions on the surface of the culture medium. The plant essential oils were dissolved in dimethylsulfoxide (DMSO) and reached a concentration of 60 mg/mL. An amount of 10 μl (equivalent to 600 μg) of essential oil was poured into the wells. The plates were placed in a 37°C incubator for 24 h. Antimicrobial activity was determined for each microorganism by measuring the halo of non-growth. Antibiotics gentamicin (10μg/disc) and rifampin (5μg/disc) were used as positive control in the same conditions of the essential oil test. The experiment was repeated three times for each sample.

#### 2-5-3-determining the minimum inhibitory concentration (MIC)

The minimum growth inhibitory concentration was calculated by microdilution method [41]. Essential oils were dissolved in a mixture of TSB and DMSO medium at an initial concentration of 4000 μg/ml. Then they were diluted appropriately using the same mixture to reach different concentrations (2000, 1000, 500, 250, 125 and 62.5 μg/mL). For this purpose, sterile 96-well microplates were prepared. 95 μL were added to each plate. In the culture medium, 5 μL of bacterial suspension with 0.5 McFarland dilution and 100 μL of different dilutions of the essential oil were added, and then the plate was heated in an incubator at 37°C for 24 h. According to the color change and turbidity of each Microplate wells were determined as MIC. The experiment was repeated three times for each sample [39].

#### 2-5-4-Determining the minimum concentration of bacterial lethality (MBC)

To determine the minimum bacterial lethal concentration test, after 24 h of heating, 5 μL from each of the microplate wells in which there was no growth were inoculated into nutrient agar medium and heated for 24 h at 37°C. Colony-forming units were counted after incubation. MBC was the minimum concentration that could effectively reduce the growth of bacteria by 99.5% [39].

### 2-6-Analysis of statistical analysis

Statistical analysis was done with SPSS 22 software. One-way analysis of variance (ANOVA) was used after checking the data’s normality. Then, using Duncan’s post hoc test at a significance level of 1%, the difference between the average values of the data was evaluated. All data were expressed as mean ± standard deviation.

## 3-Results and discussion

### 3-1-Yield of essential oils

The results of ANOVA showed that the effect of different extraction methods had a significant effect on the yield of *M*. *longifolia* leaf essential oil (p≤0.01) (Table 1). Similarly, [31] reported a significant difference between the yield of *M*. *longifolia* essential oil extracted by different methods. Our results showed that the highest yield of *M*. *longifolia* leaf essential oil belonged to the sample extracted by HDC (1.6083%). [31] obtained the highest yield of *M*. *longifolia* essential oil by lipophilic solvent extraction method (1.21 ± 0.06%), which is contrary to the present results. Past studies indicate that different methods of extracting are effective in the quantity of essential oils of aromatic medicinal plants. For example, [42] reported the highest yield of *Artemisia persica* Boiss. essential oil by the supercritical fluid method (5.7%) compared to the Clevenger method (1.7%). The properties of the extraction solvent, the particle size of raw materials, the ratio of solvent to solid, extraction temperature and extraction time affect the yield [43]. In the method of Clevenger device, high temperature increases solubility and penetration. However, very high temperatures may cause loss of solvents and lead to undesirable impurity extracts and decomposition of exothermic components. Another important factor in the extraction process is the time of the extraction process, which increases the extraction efficiency in a certain period of time. Once the equilibrium of solids in and out of solids is reached, increasing the time will have no effect on the extraction [44].

**Table 1:** Yield of *M*. *longifolia* leaf essential oil under different extraction methods.

no.	Extraction methods	Mean (%) ± SD
1	HDC	1.6083 ^a^ ±0.0033
2	SDK	0.5899 ^e^ ± 0.0001
3	SDE	1.1436 ^b^± 0.0001
4	HDM	0.3416 ^f^ ±0.0001
5	U+HDC	1.0000 ^c^ ±0.0000
6	SF	0.7090 ^d^ ± 0.0050

In previous studies on *M*. *longifolia*, its essential oil was mainly extracted by distillation with water using a Clevenger device, and various yields were reported from different regions. The highest yield of essential oil of this species has been reported from Marvdasht region of Iran (5.5%) [45].The yield of this essential oil by [33] for the population of Ifran city, Morocco as 0.76%, by [46] for the population of Mashhad, Iran as 1.83% and by [47] for the population of Kamoo, Iran is reported as 1.34%. The difference in the amount of essential oil of a species in different natural habitats can be related to factors such as the place of growth, altitude, method and time of collection and drying method of samples, soil type, climate and seasonal fluctuations and plant organs [39]. The results showed that the yield of *M*. *longifolia* leaf essential oil extracted by SDE with a value of 1.1436% (despite the same extraction time of SDE and HDC) was lower than the HDC. [48] reported the yield of *M*. *longifolia* essential oil extracted by SDE in different vegetative stages between 0.4–0.8%, which is not consistent with our results. SDE, which was introduced in 1964 by Likens and Nickerson, is one of the most widely used methods [49]. This one-step extraction technique takes less time and reduces solvent volume due to continuous recycling. Under certain conditions, higher yields and richer substances can be obtained, and essential oils obtained by SDE are free of non-volatile substances such as cuticular waxes and chlorophylls [50]. This method is usually considered superior to classical methods such as distillation or solvent extraction when it combines steam distillation with continuous extraction with a solvent or mixture of solvents [51]. As an example of [50], the highest yield of *Artemisia argyi* Lévl. et Vant by the SDE method (1.2%) compared to HDC (0.5%).

On the other hand, based on the findings, the yield of *M*. *longifolia* leaf essential oil extracted by U+HDC was in the third place (1%), which is the first time that this method has been used to extract this essential oil, and report of this method is not used for *M*. *longifolia* in previous studies. [37] reported the highest yield of the essential oil of different organs of *Moringa peregrina* (Forssk.) Fiori by U+HDC compared to HDC, which is not consistent with our results. In the extraction of essential oils, different methods such as ultrasonic waves alone and distillation with ultrasonic waves are used simultaneously. Combining new methods, in addition to maintaining their advantages and strengthening them in the combined method, also helps to reduce the disadvantages of each of them [52]. Past studies confirm the continuous use of ultrasound along with the traditional distillation process, leading to shorter extraction times and higher yields compared to methods without ultrasound [53]. During an ultrasound extraction method, an ultrasound wave passes through the sample tissue and causes the solvent to penetrate the sample and effectively extract the target molecules. The main purpose of the ultrasonic wave is to facilitate mass transfer between the sample and the extraction solvent [54]. The results indicated that the yield of *M*. *longifolia* leaf essential oil extracted by SF method was equal to 0.7090%, which was higher than the yield of HDM. [31] reported the yield of *M*. *longifolia* essential oil extracted by SF method to be 1.09% and higher than that of HDC (0.82%), which is not consistent with the present results. [42] for essential oil yield of *Artemisia persica* Boiss. and [50] for essential oil yield of *Artemisia argyi* Lévl. Et were reported yield by SF three and two times compared to HDM, respectively. In the distillation method, the step of separating the essential oil from water leads to the loss of some essential oil, while in the SF method, there is no loss of essential oil [38].

On the other hand, the results indicated that the yield of *M*. *longifolia* leaf essential oil belonging to the sample extracted by the SDK (0.5899%) is lower than all the methods of distillation with heated water and more than HDM. Our report is the first report of SDK for extracting the essential oil of *M*. *longifolia*, and it seems that the use of SDK is less efficient than the HDC to extract the essential oil of this species. Similarly, [55] reported the lowest yield of *Laurus nobilis* L. essential oil for the samples extracted under SDK compared to the HDC [56], the higher yield of the essential oil of *Syzygium aromaticum* (L.) Merr. & L.M.Perry reported the extraction by HDC compared to SDK.

The lowest yield of *M*. *longifolia* leaf essential oil belonged to the sample extracted by HDM (0.3416%). Similarly, [8] reported the yield of *M*. *longifolia* essential oil extracted by HDM from Marivan and Qazaan regions as 1.35% and 0.91%, respectively. [9], by comparing the yield of *Rosmarinus officinalis* L. essential oil with two methods of HDM and HDC, found that the extraction time of 20 min by HDM had the same yield as HDC after 180 min. In the present study, HDM with a time of 35 min was 4 times less efficient than HDC with a time of 5 h. Several studies have reported that the heat generated by microwave heating involves a partial pressure gradient of volatile compounds and internal overheating, leading to faster and more efficient embrittlement or rupture of cell walls [57, 58]. As a result, the kinetics of the extraction process of essential oils is accelerated, which explains the time difference between the two studied extraction methods. This can be explained by the speed of heat transfer between the two extraction methods. HDM uses three ways of heat transfer in the sample: radiation, conduction, and convection, while the heat transfer by HDC can only occur through conduction and convection [9]. Reduction of essential oil extraction yield, on the other hand, is related to rapid temperature changes as a result of excessive microwave radiation. This caused partial thermal decomposition of the essential oil, which had a detrimental effect on the extraction yield [59, 60]).

### 3-2-Chemical compounds of essential oils

**Fig 1:**
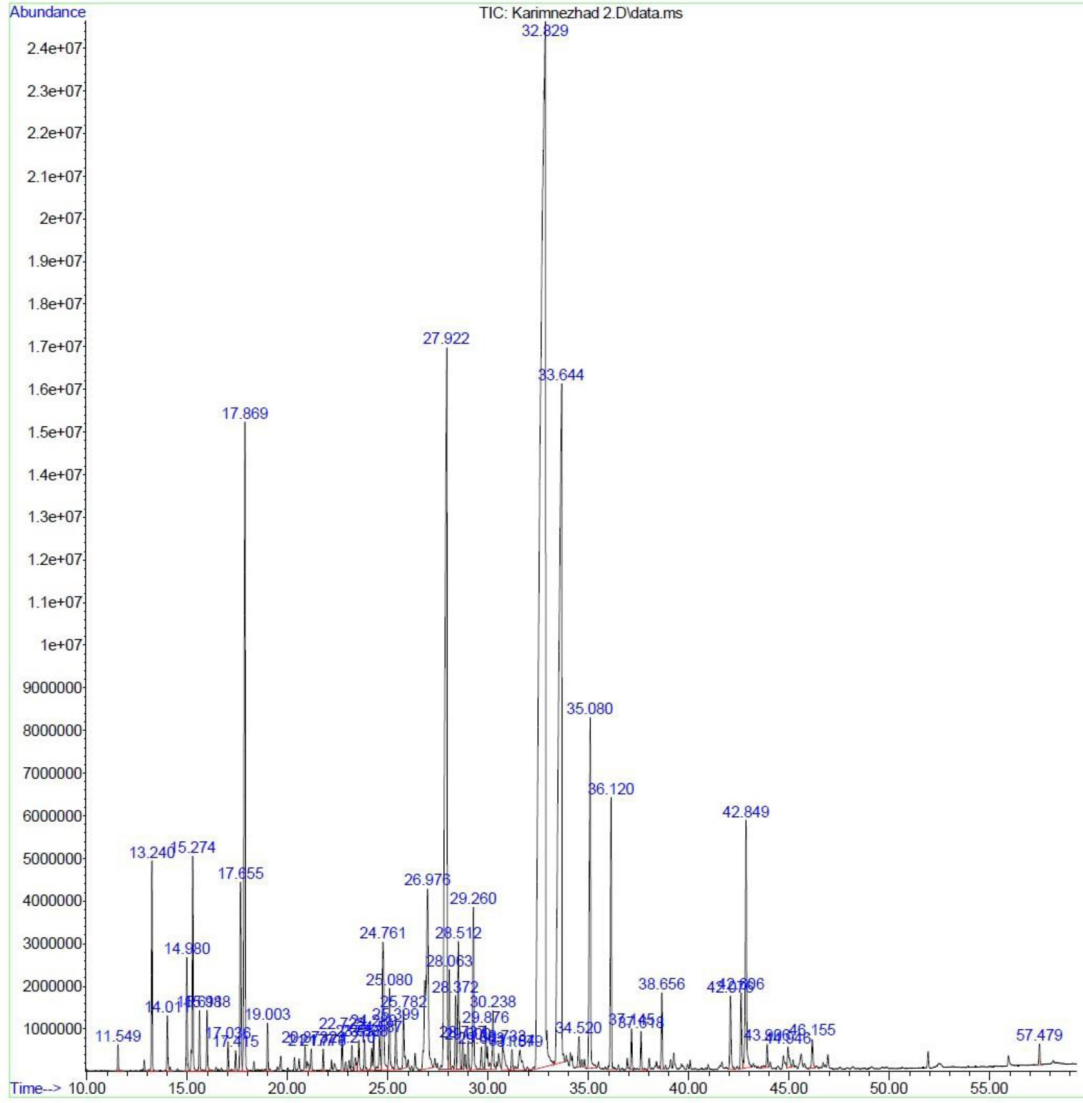
Chromatogram of *M*. *longifolia* leaf essential oil under HDC.

**Fig 2:**
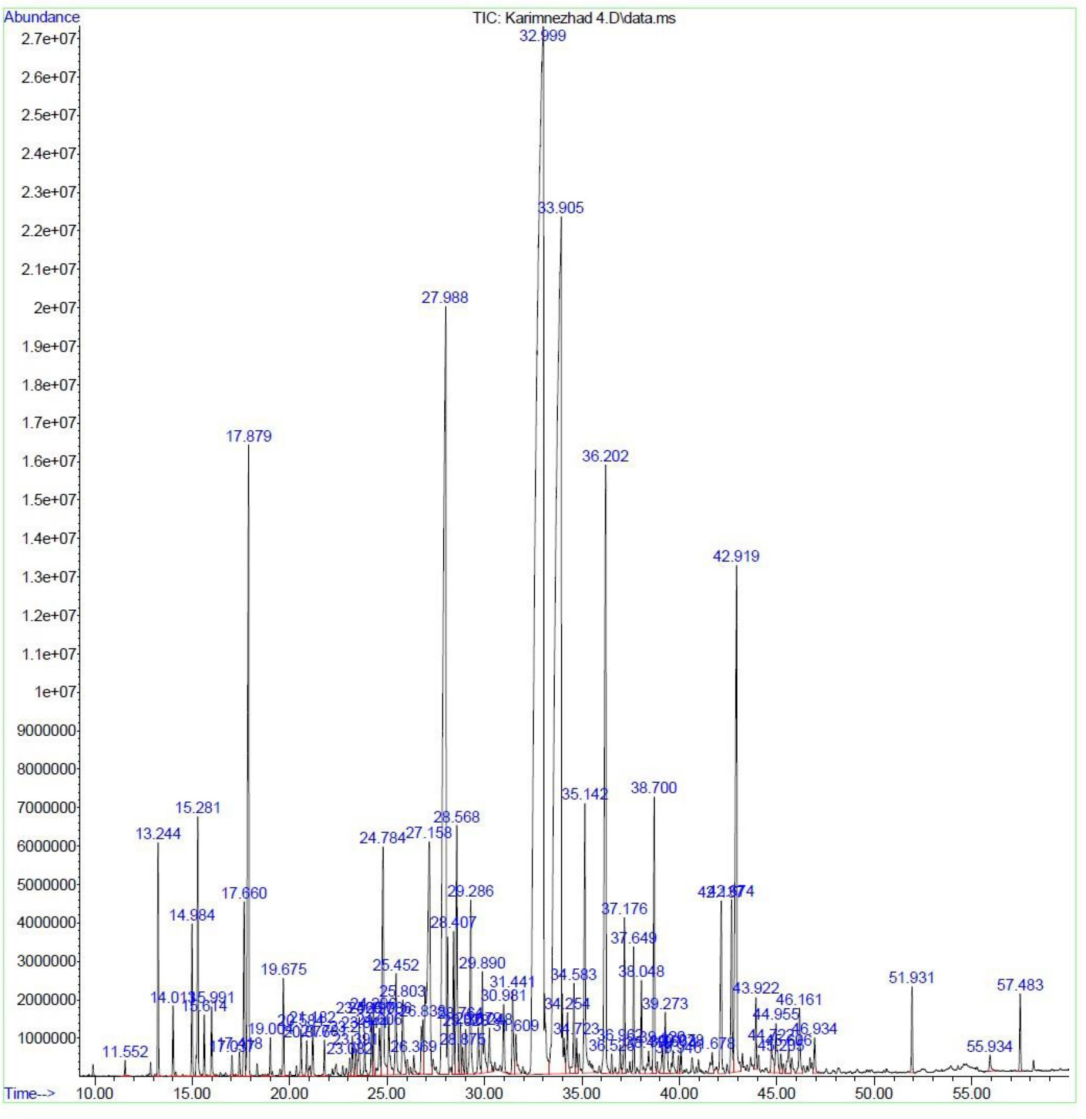
Chromatogram of *M*. *longifolia* leaf essential oil under SDK.

**Fig 3:**
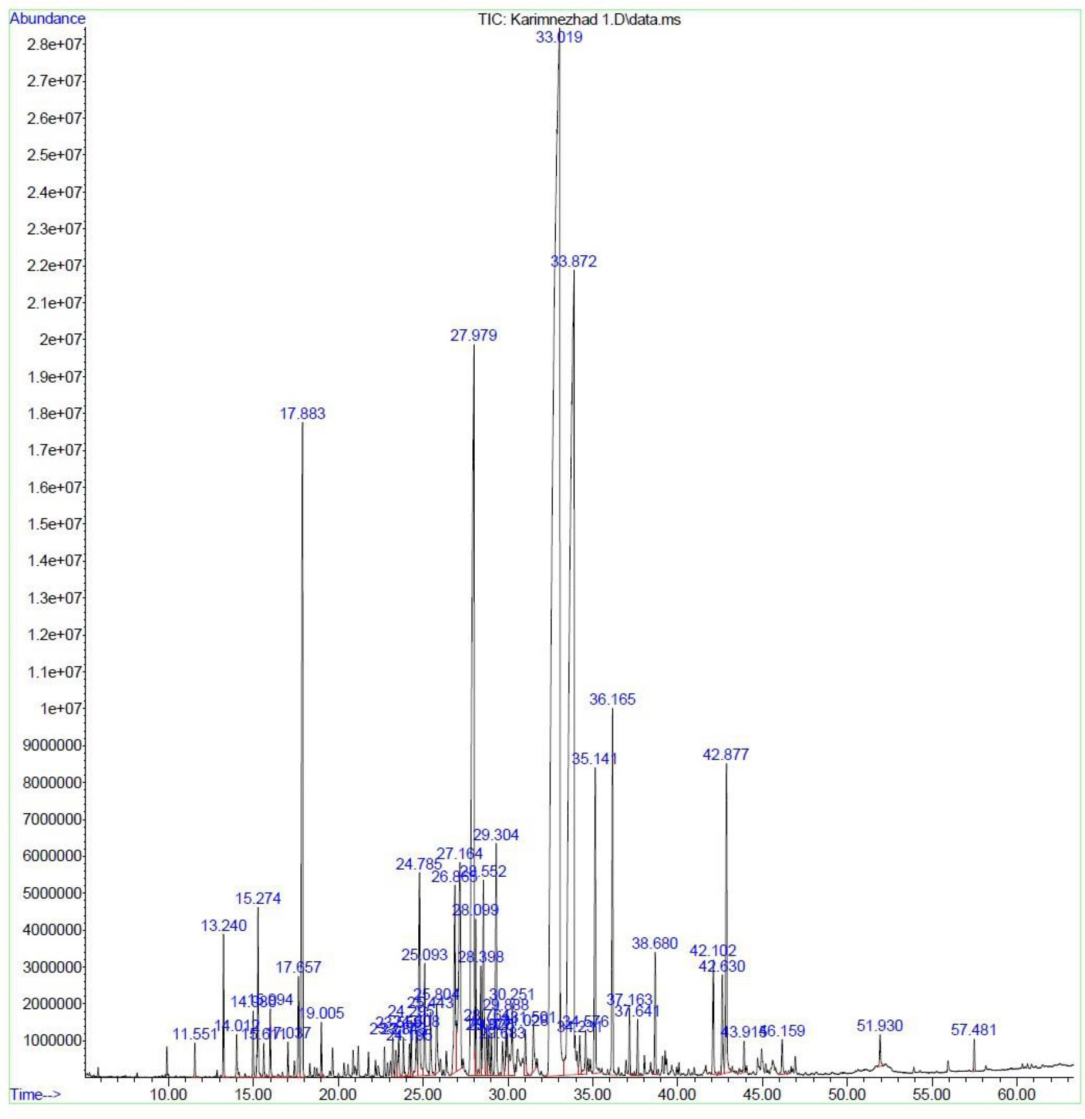
Chromatogram of *M*. *longifolia* leaf essential oil under SDE.

**Fig 4:**
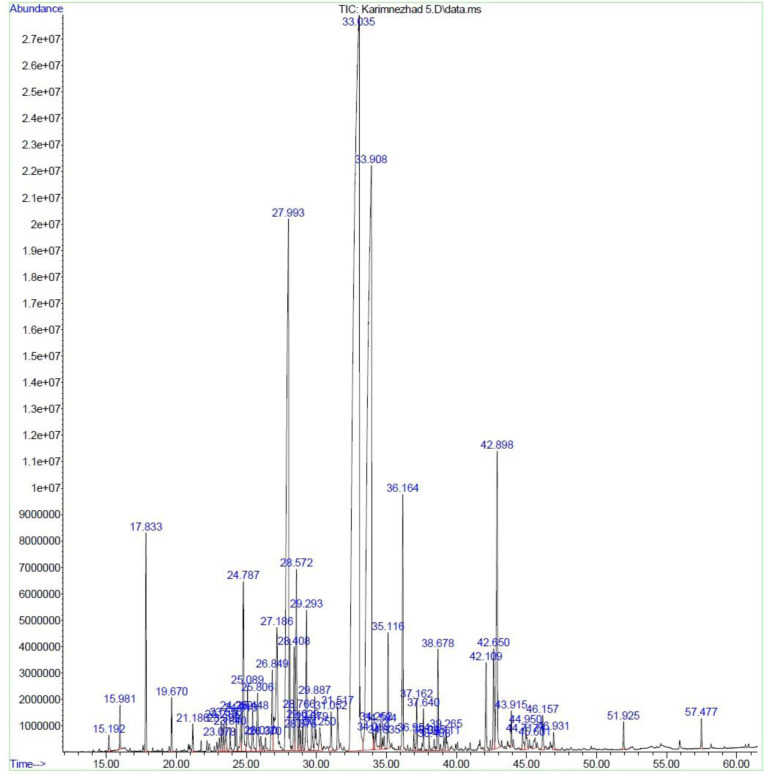
Chromatogram of *M*. *longifolia* leaf essential oil under HDM.

**Fig 5:**
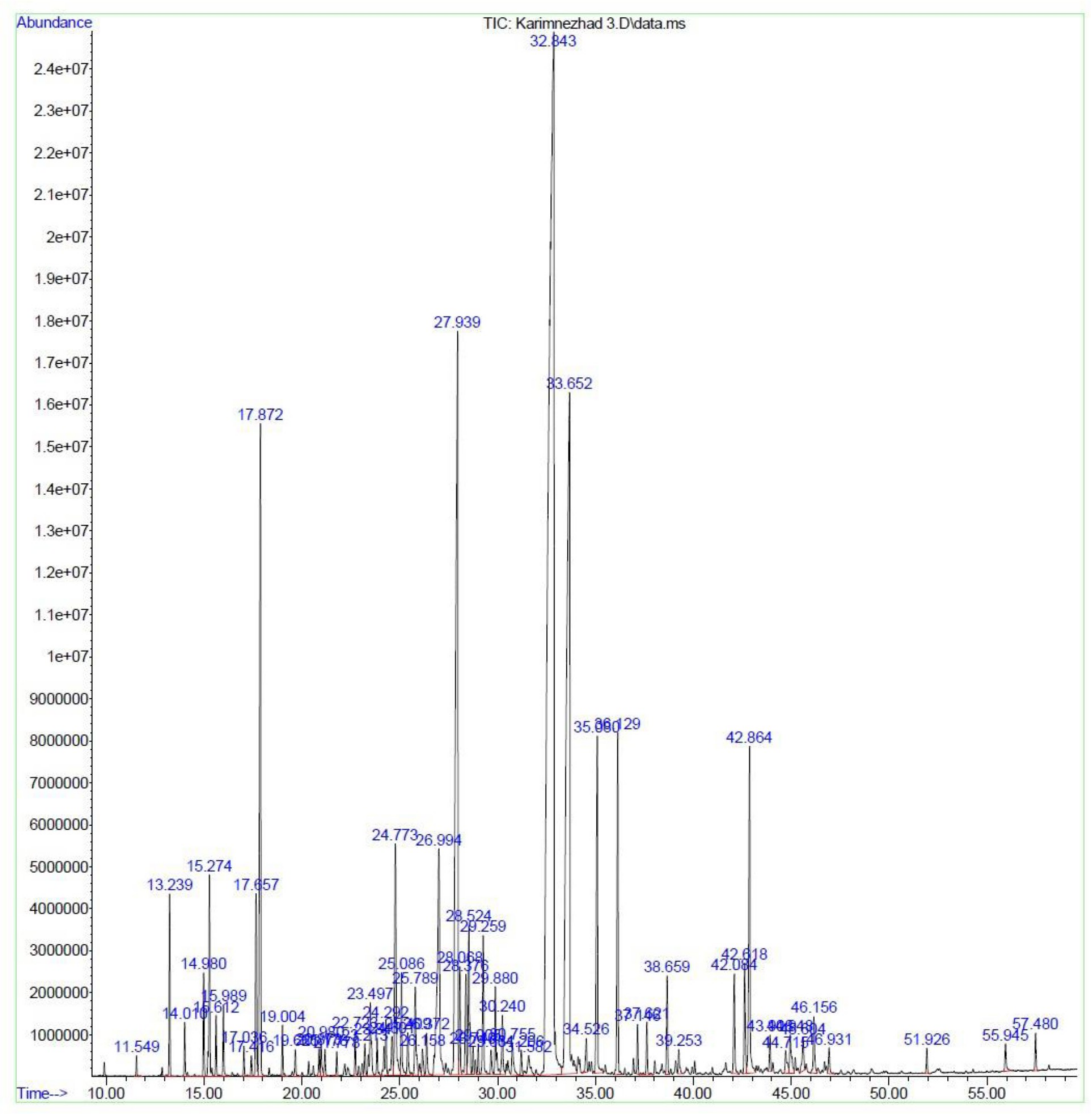
Chromatogram of *M*. *longifolia* leaf essential oil under U+HDC.

**Fig 6:**
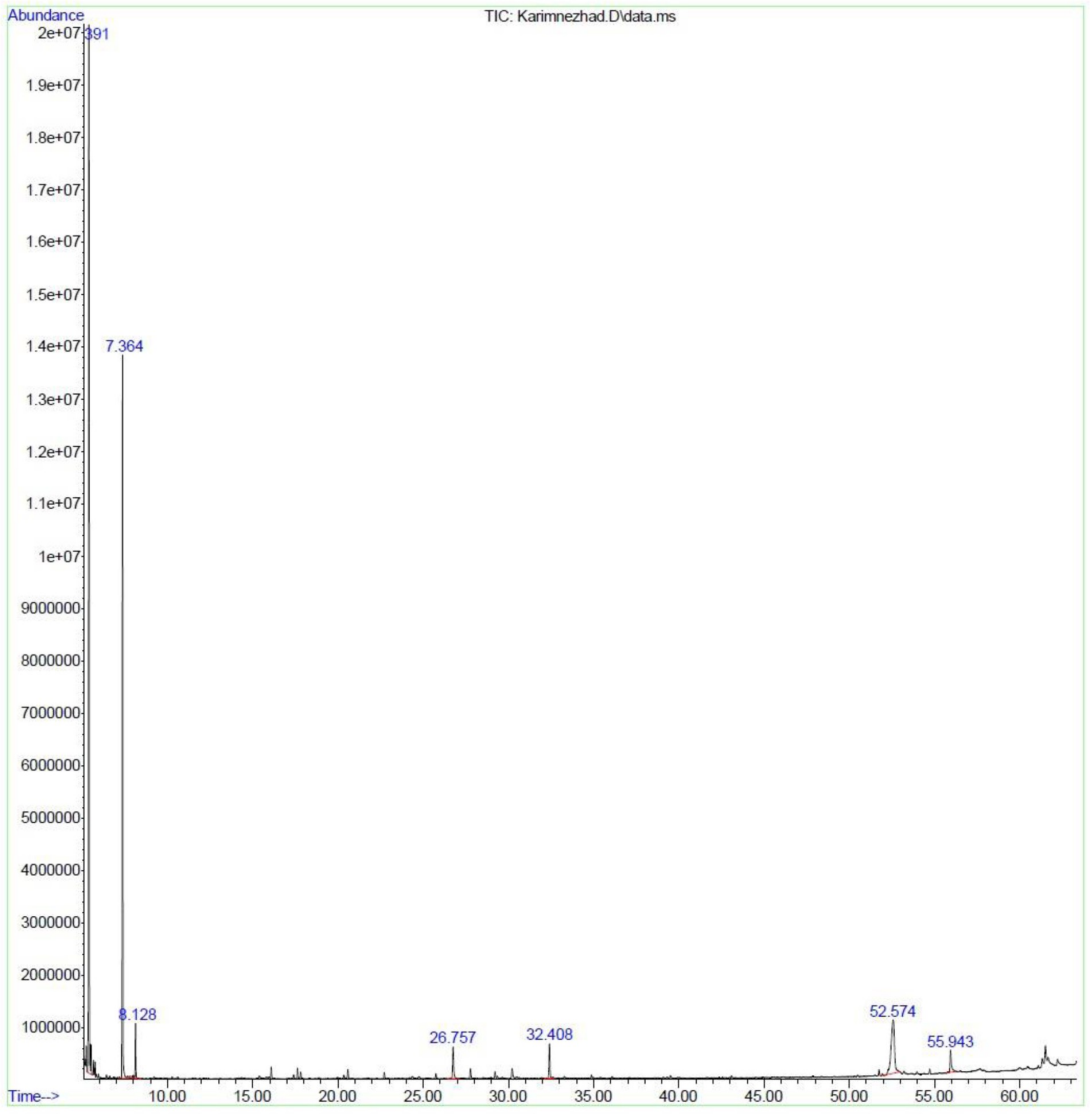
Chromatogram of *M*. *longifolia* leaf essential oil under SF.

The results of the analysis of *M*. *longifolia* leaf essential oil under different extraction methods showed that there was a significant difference between the number and relative percentage of compounds (p≤0.01) (Tables 2–7) and (Figs 1–6). Similarly, [61] reported the difference in the number and relative percentage of *M*. *longifolia* essential oil compounds under different extraction methods. The highest number of compounds belonging to the essential oil extracted by SDK and equal to 72 compounds (with a relative percentage of 87.13%) and the lowest number of compounds related to the sample of essential oil extracted by SF (7 compounds with a percentage of relative was 100 percent). [31] recorded the number of compounds of *M*. *longifolia* essential oil using SF as 27 compounds and less than HDC (32 compounds), which is in line with our results. [62] identified 39 compounds with 91.7% by SDK for this essential oil from Shahmirzad region of Semnan, Iran, which is contrary to the present results. The extraction method of plant essential oils can change the percentage and type of chemical compounds in it [63].

**Table 2:** Chemical compositions of *M*. *longifolia* leaf essential oil under HDC.

no.	Compound	RI*	RI	Mean (%) ± SD	Molecular formula
1	Furan, 2,5-diethyltetrahydro-	897	898.3	0.13	C_8_H_16_O
2	(1R)-2,6,6-Trimethylbicyclo[3.1.1]hept-2-ene = (R)-α-Pinene	936	937	1.16	C_10_H_16_
3	Camphene	953	954	0.35	C_10_H_16_
4	Bicyclo[3.1.0]hexane, 4-methylene-1-(1-methylethyl)- = Sabinene	975	977	2.06	C_10_H_16_
5	β-Myrcene	989	983	0.36	C_10_H_16_
6	3-Octanol	998	993	0.38	C_8_H_18_O
7	1,3-Cyclohexadiene, 1-methyl-4-(1-methylethyl)- = α-Terpinene	1020	1017	0.18	C_10_H_16_
8	o-Cymene	1027	1050	0.13	C_10_H_14_
9	D-Limonene	1032	-	1.44	C_10_H_16_
10	1,8-Cineole	1037	1038	5.35	C_10_H_18_O
11	γ-Terpinene	1060	1062	0.31	C_10_H_16_
12	Butanoic acid, 2-methyl-, 3-methylbutyl ester	1099	1101	0.27	C_10_H_20_O_2_
13	2H-Pyran-2-one, 6-ethyltetrahydro- = δ-lactone	1105	-	0.17	C_7_H_12_O_2_
14	3-Octanol, acetate	1117	1129	0.12	C_10_H_20_O_2_
15	Isopinocarveol	1147	1136	0.19	C_10_H_16_O
16	Pentamethylphenol	1154	-	0.27	C_11_H_16_O
17	Cyclohexanone, 5-methyl-2-(1-methylethyl)-, trans- = Menthone	1160	1157	0.23	C_10_H_18_O
18	Cyclohexanone, 5-methyl-2-(1-methylethyl)- = L-Menthone	1169	1166	0.41	C_10_H_18_O
19	Cyclohexanemethanol, α,α-dimethyl-4-methylene- = δ-Terpineol	1175	1171.3	0.34	C_10_H_18_O
20	Bicyclo[2.2.1]heptan-2-ol, 1,7,7-trimethyl-, (1S-endo)- = Borneol	1179	1173	1.44	C_10_H_18_O
21	Terpinen-4-ol	1186	1187	0.61	C_10_H_18_O
22	Benzenemethanol, α,α,4-trimethyl- = ρ-Cymene-8-ol	1192	1193	0.39	C_10_H_14_O
23	Cyclohexene, 1-methyl-3-(1-methylethenyl)-	1200	-	0.52	C_10_H_16_
24	8,9-Dehydrothymol	1226	1221.1	3.35	C_10_H_12_O
25	Cyclohexanone, 5-methyl-2-(1-methylethylidene)- = Pulegone	1246	-	10.47	C_10_H_16_O
26	D-Carvone	1249	1254	0.65	C_10_H_14_O
27	1,1’-Bicyclopentyl	1263	1080.9	0.2	C_10_H_18_
28	5-Fluoro-2-hydroxyacetophenone	1269	-	0.18	C_8_H_7_FO_2_
29	(S)-(+)-cis-Isopiperitenone	1274	-	1.3	C_10_H_14_O
30	Ethanone, 1-(2-hydroxy-5-methylphenyl)-	1283	1316	0.19	C_9_H_10_O_2_
31	Bicyclo[2.2.1]heptan-2-ol, 1,7,7-trimethyl-, acetate, (1S-endo)- = trans-Bornyl acetate	1288	1289	0.5	C_12_H_20_O_2_
32	(1s)-3,7,7-Trimethyl-bicyclo-[4.1.0]-hept-3-en-5-one	1295	-	0.47	C_10_H_14_O
33	Thymol	1306	1306	0.4	C_10_H_14_O
34	3-Methyl-4-isopropylphenol = Biosol	1316	-	0.17	C_10_H_14_O
35	5-Hepten-3-yn-2-ol, 6-methyl-5-(1-methylethyl)-	1325	-	0.44	C_11_H_18_O
36	2-Cyclohexen-1-one, 3-methyl-6-(1-methylethylidene)- = Piperitenone	1353	1349	37.77	C_10_H_14_O
37	Piperitenone oxide	1371	1369	15.94	C_10_H_14_O_2_
38	(-)-β-Bourbonene	1391	1394.5	0.26	C_15_H_24_
39	4,6-Diethyl-2-methoxypyrimidine	1404		3.27	C_9_H_14_N_2_O
40	Caryophyllene	1428	1431.8	2.03	C_15_H_24_
41	trans-β-Farnesene	1453	1457	0.27	C_15_H_24_
42	Humulene = α-Caryophyllene	1464	1477	0.27	C_15_H_24_
43	1H-Cyclopenta[1,3]cyclopropa[1,2]benzene, octahydro-7-methyl-3-methylene-4-(1-methylethyl)-, [3aS-(3aα,3bβ,4β,7α,7aS*)]- = β-Cubebene	1488	1434	0.56	C_15_H_24_
44	Cyclohexanecarboxylic acid, 1-ethyl-2-oxo-, ethyl ester	1573	-	0.64	C_11_H_18_O_3_
45	1H-Cycloprop[e]azulen-7-ol, decahydro-1,1,7-trimethyl-4-methylene-, [1ar-(1aα,4aα,7β,7aβ,7bα)]- = Spathulenol	1587	1585	0.61	C_15_H_24_O
46	Caryophyllene oxide	1593	1598	2.17	C_15_H_24_O
47	(1R,3E,7E,11R)-1,5,5,8-Tetramethyl-12-oxabicyclo[9.1.0]dodeca-3,7-diene = α-Humulene epoxide II	1620	1610	0.21	C_15_H_24_O
48	10,10-Dimethyl-2,6-dimethylenebicyclo[7.2.0]undecan-5β-ol	1647	1644.2	0.25	C_15_H_24_O
49	Presilphiperfolane-9,15-epoxide	1679	-	0.26	C_15_H_24_O
50	1H-Naphtho[2,1-b]pyran, 3-ethenyldodecahydro-3,4a,7,7,10a-pentamethyl-, [3R-(3α,4aβ,6aα,10aβ,10bα)]- = Manoyl oxide	2006	2015	0.15	C_20_H_34_O
	Total			99.79	
	Monoterpenes hydrocarbons			6.71	
	Oxygenated monoterpenes			63.54	
	Sesquiterpenes hydrocarbons			3.39	
	Oxygenated sesquiterpenes			3.5	
	Others (Nonterpenoids)			22.65	

**Table 3:** Chemical compositions of *M*. *longifolia* leaf essential oil under SDK.

no.	Compound	RI*	RI	Mean (%) ± SD	Molecular formula
1	Furan, 2,5-diethyltetrahydro-	897	898.3	0.05	C_8_H_16_O
2	α-Pinene	936	937	0.76	C_10_H_16_
3	Camphene	953	954	0.25	C_10_H_16_
4	Bicyclo[3.1.0]hexane, 4-methylene-1-(1-methylethyl)- = α-Sabinene	975	977	1.53	C_10_H_16_
5	β-Myrcene	989	983	0.22	C_10_H_16_
6	3-Octanol	998	993	0.27	C_8_H_18_O
7	1,3-Cyclohexadiene, 1-methyl-4-(1-methylethyl)- = α-Terpinene	1020	1017	0.09	C_10_H_16_
8	o-Cymene	1027	1050	0.09	C_10_H_14_
9	D-Limonene	1032	-	0.82	C_10_H_16_
10	1,8-Cineole	1037	1038	3.27	C_10_H_18_O
11	γ-Terpinene	1060	1062	0.18	C_10_H_16_
12	Bicyclo[3.1.0]hexan-2-ol, 2-methyl-5-(1-methylethyl)-, (1α,2α,5α)- = trans-Sabinene hydrate	1074	1075	0.37	C_10_H_18_O
13	Benzene, 1-methyl-4-(1-methylethenyl)- = Cymenene	1093	1095	0.19	C_10_H_12_
14	Butanoic acid, 2-methyl-, 3-methylbutyl ester	1099	1101	0.14	C_10_H_20_O_2_
15	Bicyclo[3.1.0]hexan-2-ol, 2-methyl-5-(1-methylethyl)-, (1α,2α,5α)- = trans-Sabinene hydrate	1105	1075	0.21	C_10_H_18_O
16	3-Octanol, acetate	1118	1129	0.13	C_10_H_20_O_2_
17	Bicyclo[3.1.0]hexan-3-ol, 4-methylene-1-(1-methylethyl)-, [1S-(1α,3β,5α)]- = Sabinol	1144	1139	0.08	C_10_H_16_O
18	Bicyclo[3.1.1]heptan-3-ol, 6,6-dimethyl-2-methylene-, [1S-(1α,3α,5α)]- = L-Pinocarveol	1147	1143	0.18	C_10_H_16_O
19	trans-Verbenol	1151	1148	0.13	C_10_H_16_O
20	(1S,6R)-3,7,7-Trimethylbicyclo[4.1.0]hept-3-ene-2,5-dione	1154	-	0.28	C_10_H_12_O_2_
21	Cyclohexanone, 5-methyl-2-(1-methylethyl)-, trans- = Menthone	1160	1157	0.2	C_10_H_18_O
22	Benzofuran, 4,5,6,7-tetrahydro-3,6-dimethyl-	1167	1169	0.23	C_10_H_14_O
23	Cyclohexanone, 5-methyl-2-(1-methylethyl)-, trans- = Menthone	1169	1157	0.23	C_10_H_18_O
24	Cyclohexanemethanol, α,α-dimethyl-4-methylene- = δ-Terpineol	1176	1171.3	0.3	C_10_H_18_O
25	Borneol	1179	1173	1.45	C_10_H_18_O
26	Terpinen-4-ol	1186	1176	0.4	C_10_H_18_O
27	Benzenemethanol, α,α,4-trimethyl- = p-Cymen-8-ol	1193	1193	0.54	C_10_H_14_O
28	α-Terpineol	1201	1200	0.52	C_10_H_18_O
29	8,9-Dehydrothymol	1230	1221.1	2.86	C_10_H_12_O
30	Pulegone	1247	1244	9.03	C_10_H_16_O
31	Piperitenone oxide	1256	1330	21.12	C_10_H_14_O_2_
32	Naphthalene, decahydro-	1264	1056	0.22	C_10_H_18_
33	2-Cyclopenten-1-one, 3-methyl-	1266	-	0.11	C_6_H_8_O
34	2,4-Dimethoxyphenol	1269	-	0.17	C_8_H_10_O_3_
35	(+)-Isopiperitenone	1275	-	0.92	C_10_H_14_O
36	Acetic acid, 4-methylphenyl ester = Narceol	1283	1171.4	0.27	C_9_H_10_O_2_
37	Bicyclo[2.2.1]heptan-2-ol, 1,7,7-trimethyl-, acetate, (1S-endo)- = trans-Bornyl acetate	1288	1289	0.64	C_12_H_20_O_2_
38	Benzene, 1-ethoxy-4-ethyl-	1295	-	0.29	C_10_H_14_O
39	Thymol	1312	1306	0.37	C_10_H_14_O
40	Phenol, 2-methyl-5-(1-methylethyl)- = Carvacrol	1322	1317	0.47	C_10_H_14_O
41	2,6-Dimethyl-2,6-octadiene-1,8-diol diacetate	1326	1740.6	0.27	C_14_H_22_O_4_
42	2-Cyclohexen-1-one, 3-methyl-6-(1-methylethylidene)- = Piperitenone	1357	1349	31.13	C_10_H_14_O
43	2’,6’-Dihydroxy-3’-methylacetophenone	1385		0.33	C_9_H_10_O_3_
44	(-)-β-Bourbonene	1392	1394.5	0.45	C_15_H_24_
45	Cyclohexane, 1-ethenyl-1-methyl-2,4-bis(1-methylethenyl)-, [1S-(1α,2β,4β)]- = β-Elemene	1396	1399	0.15	C_15_H_24_
46	4,6-Diethyl-2-methoxypyrimidine	1405	-	1.55	C_9_H_14_N_2_O
47	Caryophyllene	1430	1431.8	3.81	C_15_H_24_
48	β-Copaene	1438	1433	0.08	C_15_H_24_
49	5,9-Undecadien-2-one, 6,10-dimethyl-, (E)- = Geranyl acetone	1448	1446	0.14	C_13_H_22_O
50	(E)-β-Famesene	1453	1454	0.65	C_15_H_24_
51	Humulene = α-Caryophyllene	1465	1477	0.5	C_15_H_24_
52	2-Cyclohexen-1-one, 3-methyl-6-(1-methylethylidene)- = Piperitenone	1474	1349	0.41	C_10_H_14_O
53	3-Buten-2-one, 4-(2,6,6-trimethyl-1-cyclohexen-1-yl)- = β-Ionone	1483	1488.4	0.19	C_13_H_20_O
54	Germacrene D	1489	1496	1.32	C_15_H_24_
55	(1S,6R)-3,7,7-Trimethylbicyclo[4.1.0]hept-3-ene-2,5-dione	1499	-	0.16	C_10_H_12_O_2_
56	Bicyclo[8.1.0]undeca-2,6-diene, 3,7,11,11-tetramethyl-, (1R*,2Z,6E,10R*)-(.+-.)-	1503	-	0.44	C_15_H_24_
57	1-(3-Methyl-cyclopent-2-enyl)-cyclohexene	1513	-	0.17	C_12_H_18_
58	Naphthalene, 1,2,3,4,4a,5,6,8a-octahydro-7-methyl-4-methylene-1-(1-methylethyl)-, (1α,4aβ,8aα)- = γ-Cadinene	1520	1524	0.08	C_15_H_24_
59	Naphthalene, 1,2,3,5,6,8a-hexahydro-4,7-dimethyl-1-(1-methylethyl)-, (1S-cis)- = δ-Cadinene	1523	1530	0.1	C_15_H_24_
60	Caryophylla-3,8(13)-dien-5β-ol	1563	1641	0.4	C_15_H_24_O
61	Cyclohexanecarboxylic acid, 1-ethyl-2-oxo-, ethyl ester	1575	-	0.99	C_11_H_18_O_3_
62	1H-Cycloprop[e]azulen-7-ol, decahydro-1,1,7-trimethyl-4-methylene-, [1ar-(1aα,4aα,7β,7aβ,7bα)]- = Spatulenol	1588	1593	0.91	C_15_H_24_O
63	Caryophyllene oxide	1594	1598	3.4	C_15_H_24_O
64	(1R,3E,7E,11R)-1,5,5,8-Tetramethyl-12-oxabicyclo[9.1.0]dodeca-3,7-diene = α-Humulene epoxide II	1620	1610	0.28	C_15_H_24_O
65	Isospathulenol	1642	1640	0.23	C_15_H_24_O
66	10,10-Dimethyl-2,6-dimethylenebicyclo[7.2.0]undecan-5β-ol	1648	1644.2	0.34	C_15_H_24_O
67	3-Buten-2-one, 4-(2,6,6-trimethyl-1-cyclohexen-1-yl)- = β-Ionone	1654	1496	0.09	C_13_H_20_O
68	Caryophyllenol-II	1679	1676	0.36	C_15_H_24_O
69	Pyrazino[2’,3’:4,5]thieno[3,2-d]pyrimidine-4(1H)-thione	1700	-	0.18	C_8_H_4_N_4_S_2_
70	2-Pentadecanone, 6,10,14-trimethyl-	1841	1846.7	0.34	C_18_H_36_O
71	n-Hexadecanoic acid	1961	1964	0.1	C_16_H_32_O_2_
72	1H-Naphtho[2,1-b]pyran, 3-ethenyldodecahydro-3,4a,7,7,10a-pentamethyl-, [3R-(3α,4aβ,6aα,10aβ,10bα)]- = Manoyl oxide	2006	2015	0.32	C_20_H_34_O
	**Total**			78.13	
	Monoterpenes hydrocarbons			4.35	
	Oxygenated monoterpenes			53.29	
	Sesquiterpenes hydrocarbons			7.58	
	Oxygenated sesquiterpenes			5.92	
	Others (Nonterpenoids)			6.89	

**Table 4:** Chemical compositions of *M*. *longifolia* leaf essential oil under SDE.

no.	Compound	RI*	RI	Mean (%) ± SD	Molecular formula
1	Furan, 2,5-diethyltetrahydro-	897	898.3	0.12	C_8_H_16_O
2	(1R)-2,6,6-Trimethylbicyclo[3.1.1]hept-2-ene	936	935	0.54	C_10_H_16_
3	Camphene	953	954	0.22	C_10_H_16_
4	Bicyclo[3.1.0]hexane, 4-methylene-1-(1-methylethyl)- = α-Sabinene	975	977	1.1	C_10_H_16_
5	β-Myrcene	989	983	0.14	C_10_H_16_
6	3-Octanol	998	993	0.31	C_8_H_18_O
7	1,3-Cyclohexadiene, 1-methyl-4-(1-methylethyl)- = α-Terpinene	1020	1017	0.21	C_10_H_16_
8	D-Limonene	1032	-	0.59	C_10_H_16_
9	1,8-Cineole	1037	1038	4.28	C_10_H_18_O
10	γ-Terpinene	1060	1062	0.22	C_10_H_16_
11	Isopinocarveol	1147	1136	0.21	C_10_H_16_O
12	Pentamethylphenol	1154	-	0.28	C_11_H_16_O
13	Cyclohexanone, 5-methyl-2-(1-methylethyl)-, trans- = Menthone	1160	1157	0.46	C_10_H_18_O
14	Bicyclo[3.1.1]heptane, 6,6-dimethyl-2-methylene-, (1S)-	1167	-	0.15	C_10_H_16_
15	Cyclohexanemethanol, α,α-dimethyl-4-methylene- = δ-Terpineol	1176	1171.3	0.36	C_10_H_18_O
16	Bicyclo[2.2.1]heptan-2-ol, 1,7,7-trimethyl-, (1S-endo)- = Borneol	1179	1173	1.51	C_10_H_18_O
17	Terpinen-4-ol	1186	1176	0.68	C_10_H_18_O
18	Benzenemethanol, α,α,4-trimethyl- = p-Cymen-8-ol	1193	1193	0.4	C_10_H_14_O
19	Cyclohexene, 1-methyl-3-(1-methylethenyl)-	1201		0.52	C_10_H_16_
20	8,9-Dehydrothymol	1230	1221.1	2.29	C_10_H_12_O
21	Pulegone	1247	1244	9.34	C_10_H_16_O
22	(-)-Carvone	1250	-	0.6	C_10_H_14_O
23	Piperitenone oxide	1256	1330	20.38	C_10_H_14_O_2_
24	7-Oxabicyclo[4.1.0]heptan-2-one, 6-methyl-3-(1-methylethyl)- = Pipertone, oxide	1259	1259	1.27	C_10_H_16_O_2_
25	1,1’-Bicyclopentyl	1264	1080.9	0.28	C_10_H_18_
26	2-methyl-2-vinyl-5-isopropyltetrahydrofuran	1266	-	0.21	C_10_H_18_O
27	5-Fluoro-2-hydroxyacetophenone	1269	-	0.2	C_8_H_7_FO_2_
28	(+)-Isopiperitenone	1275	-	1.52	C_10_H_14_O
29	4-Hydroxy-3-methylacetophenone	1283	1323	0.25	C_9_H_10_O_2_
30	Bicyclo[2.2.1]heptan-2-ol, 1,7,7-trimethyl-, acetate, (1S-endo)- = trans-Bornyl acetate	1288	1289	0.53	C_12_H_20_O_2_
31	Benzene, 1-ethoxy-4-ethyl-	1296	-	0.69	C_10_H_14_O
32	Thymol	1313	1306	0.31	C_10_H_14_O
33	3-Methyl-4-isopropylphenol = Biosol	1323	-	0.35	C_10_H_14_O
34	2-Cyclohexen-1-one, 3-methyl-6-(1-methylethylidene)- = Piperitenone	1357	1349	38.06	C_10_H_14_O
35	2’,6’-Dihydroxy-3’-methylacetophenone	1385	-	0.26	C_9_H_10_O_3_
36	(-)-β-Bourbonene	1392	1394.5	0.28	C_15_H_24_
37	4,6-Diethyl-2-methoxypyrimidine	1405	-	1.88	C_9_H_14_N_2_O
38	Caryophyllene	1429	1431.8	2.09	C_15_H_24_
39	beta-Farnesene	1453		0.3	C_15_H_24_
40	Humulene = α-Caryophyllene	1464	1477	0.31	C_15_H_24_
41	1H-Cyclopenta[1,3]cyclopropa[1,2]benzene, octahydro-7-methyl-3-methylene-4-(1-methylethyl)-, [3aS-(3aα,3bβ,4β,7α,7aS*)]- = β-Cubebene	1489	1434	0.66	C_15_H_24_
42	Cyclohexanecarboxylic acid, 1-ethyl-2-oxo-, ethyl ester	1574	-	0.68	C_11_H_18_O_3_
43	(-)-Spathulenol	1587	1582	0.59	C_15_H_24_O
44	Caryophyllene oxide	1593	1598	1.97	C_15_H_24_O
45	(1R,3E,7E,11R)-1,5,5,8-Tetramethyl-12-oxabicyclo[9.1.0]dodeca-3,7-diene = α-Humulene epoxide II	1620	1610	0.21	C_15_H_24_O
46	Caryophyllenol-II	1679	1676	0.25	C_15_H_24_O
47	2-Pentadecanone, 6,10,14-trimethyl-	1841	1846.7	0.19	C_18_H_36_O
48	1H-Naphtho[2,1-b]pyran, 3-ethenyldodecahydro-3,4a,7,7,10a-pentamethyl-, [3R-(3α,4aβ,6aα,10aβ,10bα)]- = Manoyl oxide	2006	2015	0.16	C_20_H_34_O
	**Total**			95.61	
	Monoterpenes hydrocarbons			3.97	
	Oxygenated monoterpenes			61.27	
	Sesquiterpenes hydrocarbons			3.64	
	Oxygenated sesquiterpenes			3.02	
	Others (Nonterpenoids)			26.71	

**Table 5:** Chemical compositions of *M*. *longifolia* leaf essential oil under HDM.

no.	Compound	RI*	RI	Mean (%) ± SD	Molecular formula
1	1-Octen-3-ol = Vinyl hexanol	980	986	0.12	C_8_H_16_O
2	3-Octanol	998	993	0.25	C_8_H_18_O
3	1,8-Cineole	1036	1038	1.38	C_10_H_18_O
4	Bicyclo[3.1.0]hexan-2-ol, 2-methyl-5-(1-methylethyl)-, (1α,2α,5α)- = trans-Sabinene hydrate	1074	1075	0.52	C_10_H_18_O
5	α-Phellandrene epoxide	1144	1187	0.12	C_10_H_16_O
6	Bicyclo[3.1.1]heptan-3-ol, 6,6-dimethyl-2-methylene-, [1S-(1α,3α,5α)]- = L-Pinocarveol	1147	1143	0.23	C_10_H_16_O
7	trans-Verbenol	1151	1148	0.22	C_10_H_16_O
8	1(2H)-Naphthalenone, 3,4,5,6,7,8-hexahydro-7-methyl-	1154	-	0.27	C_11_H_16_O
9	2-Isopropyl-5-methylcyclohexane-1,3-dione	1160	-	0.19	C_10_H_16_O_2_
10	Cyclohexanone, 5-methyl-2-(1-methylethyl)-, trans- = Menthone	1169	1157	0.4	C_10_H_18_O
11	Cyclohexanemethanol, α,α-dimethyl-4-methylene- = δ-Terpineol	1176	1171.3	0.42	C_10_H_18_O
12	Bicyclo[2.2.1]heptan-2-ol, 1,7,7-trimethyl-, (1S-endo)- = Borneol	1179	1173	1.79	C_10_H_18_O
13	Terpinen-4-ol	1185	1176	0.59	C_10_H_18_O
14	Benzenemethanol, α,α,4-trimethyl- = p-Cymen-8-ol	1193	1193	0.37	C_10_H_14_O
15	Cyclohexene, 1-methyl-3-(1-methylethenyl)-	1201		0.67	C_10_H_16_
16	(-)-trans-Isopiperitenol	1205	1210.2	0.15	C_10_H_16_O
17	1-Indanone, 4,5,6,7-tetrahydro-3-methyl-	1213		0.13	C_9_H_12_O
18	3-Heptene, 2,2,3,5,6-pentamethyl-	1223		0.79	C_12_H_24_
19	8,9-Dehydrothymol	1230	1221.1	2.41	C_10_H_12_O
20	Pulegone	1247	1244	10.81	C_10_H_16_O
21	Piperitenone oxide	1256	1330	22.02	C_10_H_14_O_2_
22	7-Oxabicyclo[4.1.0]heptan-2-one, 6-methyl-3-(1-methylethyl)- = Pipertone, oxide	1260	1259	1.71	C_10_H_16_O_2_
23	3a,4,5,6,7,7a-Hexahydro-4,7-methanoindene	1264	-	0.28	C_10_H_14_
24	2-methyl-2-vinyl-5-isopropyltetrahydrofuran	1266	-	0.14	C_10_H_18_O
25	5-Fluoro-2-hydroxyacetophenone	1269	-	0.19	C_8_H_7_FO_2_
26	(+)-Isopiperitenone	1275		1.24	C_10_H_14_O
27	4-(Methoxymethoxy)benzaldehyde	1283	-	0.3	C_9_H_10_O_3_
28	Bicyclo[2.2.1]heptan-2-ol, 1,7,7-trimethyl-, acetate, (1S-endo)- = trans-Bornyl acetate	1288	1289	0.57	C_12_H_20_O_2_
29	Benzene, 1-ethoxy-4-ethyl-	1296	-	0.34	C_10_H_14_O
30	Thymol	1313	1306	0.37	C_10_H_14_O
31	Phenol, 2-methyl-5-(1-methylethyl)- = Carvacrol	1324	1317	0.42	C_10_H_14_O
32	2-Cyclohexen-1-one, 3-methyl-6-(1-methylethylidene)- = Piperitenone	1358	1349	38.41	C_10_H_14_O
33	Benzoic acid, 4-methoxy-, methyl ester	1381	1376	0.15	C_9_H_10_O_3_
34	2’,6’-Dihydroxy-3’-methylacetophenone	1385	-	0.29	C_9_H1_0_O_3_
35	(-)-β-Bourbonene	1392	1394.5	0.17	C_15_H_24_
36	2-Cyclopenten-1-one, 3-methyl-2-(2-pentenyl)-, (Z)- = Jasmone	1398	1396	0.18	C_11_H_16_O
37	4,6-Diethyl-2-methoxypyrimidine	1405		0.84	C_9_H_14_N_2_O
38	Caryophyllene	1429	1431.8	2.04	C_15_H_24_
39	5,9-Undecadien-2-one, 6,10-dimethyl-, (E)- = Geranyl acetone	1448	1446	0.13	C_13_H_22_O
40	(E)-β-Famesene	1453	1454	0.33	C_15_H_24_
41	Humulene = α-Caryophyllene	1464	1477	0.34	C_15_H_24_
42	1,3-Hexadiene, 4-chloro-2,3-dimethyl-, (E)-	1474	-	0.14	C_8_H1_3_C_l_
43	trans-β-Ionone	1483	1485	0.11	C_13_H_20_O
44	1H-Cyclopenta[1,3]cyclopropa[1,2]benzene, octahydro-7-methyl-3-methylene-4-(1-methylethyl)-, [3aS-(3aα,3bβ,4β,7α,7aS*)]- = β-Cubebene	1489	1434	0.81	C_15_H_24_
45	(1S,6R)-3,7,7-Trimethylbicyclo[4.1.0]hept-3-ene-2,5-dione	1499	-	0.12	C_10_H_12_O_2_
46	Bicyclo[8.1.0]undeca-2,6-diene, 3,7,11,11-tetramethyl-, (1R*,2Z,6E,10R*)-(.+-.)-	1503	-	0.2	C_15_H_24_
47	Cyclohexanecarboxylic acid, 1-ethyl-2-oxo-, ethyl ester	1574	-	0.75	C_11_H_18_O_3_
48	(-)-Spathulenol	1588	1582	0.85	C_15_H_24_O
49	Caryophyllene oxide	1594	1598	3.48	C_15_H_24_O
50	(1R,3E,7E,11R)-1,5,5,8-Tetramethyl-12-oxabicyclo[9.1.0]dodeca-3,7-diene = α-Humulene epoxide II	1620	1610	0.24	C_15_H_24_O
51	Isospathulenol	1641	1640	0.2	C_15_H_24_O
52	Caryophylla-4(12),8(13)-dien-5-β-ol = α-Caryophylladienol	1648	1661	0.29	C_15_H_24_O
53	(E)-3-(4-Aminophenyl)-3-phenyl-2-propenenitrile	1700	-	0.15	C_15_H_10_FN
54	2-Pentadecanone, 6,10,14-trimethyl-	1838	1846.7	0.18	C_18_H_36_O
55	1H-Naphtho[2,1-b]pyran, 3-ethenyldodecahydro-3,4a,7,7,10a-pentamethyl-, [3R-(3α,4aβ,6aα,10aβ,10bα)]- = Manoyl oxide	2006	2015	0.21	C_20_H_34_O
	**Total**			100.00	
	Monoterpenes hydrocarbons			0.95	
	Oxygenated monoterpenes			60.33	
	Sesquiterpenes hydrocarbons			3.89	
	Oxygenated sesquiterpenes			5.06	
	Others (Nonterpenoids)			29.97	

**Table 6:** Chemical compositions of *M*. *longifolia* leaf essential oil under U+HDC.

no.	Compound	RI*	RI	Mean (%) ± SD	Molecular formula
1	Furan, 2,5-diethyltetrahydro-	897	898.3	0.09	C_8_H_16_O
2	(1S)-2,6,6-Trimethylbicyclo[3.1.1]hept-2-ene = L-α-Pinene	936	936	0.88	C_10_H_16_
3	Camphene	953	954	0.29	C_10_H_16_
4	Bicyclo[3.1.0]hexane, 4-methylene-1-(1-methylethyl)- = α-Sabinene	975	977	0.55	C_10_H_16_
5	β-Pinene	982	980	1.22	C_10_H_16_
6	β-Myrcene	989	983	0.31	C_10_H_16_
7	3-Octanol	998	993	0.4	C_8_H_18_O
8	1,3-Cyclohexadiene, 1-methyl-4-(1-methylethyl)- = α-Terpinene	1020	1017	0.16	C_10_H_16_
9	p-Cymene = Cymol	1027	1026	0.12	C_10_H_14_
10	D-Limonene	1032	-	1.26	C_10_H_16_
11	1,8-Cineole	1037	1038	4.9	C_10_H_18_O
12	γ-Terpinene	1060	1062	0.27	C_10_H_16_
13	Bicyclo[3.1.0]hexan-2-ol, 2-methyl-5-(1-methylethyl)-, (1α,2α,5α)- = trans-Sabinene hydrate	1074	1075	0.16	C_10_H_18_O
14	Butanoic acid, 2-methyl-, 3-methylbutyl ester	1083	1101	0.15	C_10_H_20_O_2_
15	Linalool	1101	1106	0.25	C_10_H_18_O
16	Pentane, 3-ethyl-	1105	-	0.17	C_7_H_16_
17	3-Octanol, acetate	1117	1129	0.13	C_10_H_20_O_2_
18	2-Methoxy-1,4-benzenediamine	1137	-	0.26	C_7_H_10_N_2_O
19	Bicyclo[3.1.1]heptan-3-ol, 6,6-dimethyl-2-methylene-, [1S-(1α,3α,5α)]- = L-Pinocarveol	1147	1143	0.21	C_10_H_16_O
20	(+)-2-Bornanone = Camphor USP	1153	1144	0.78	C_10_H_16_O
21	Cyclohexanone, 5-methyl-2-(1-methylethyl)-, trans- = Menthone	1160	1157	0.76	C_10_H_18_O
22	Cyclohexanemethanol, α,α-dimethyl-4-methylene- = δ-Terpineol	1176	1171.3	0.4	C_10_H_18_O
23	Bicyclo[2.2.1]heptan-2-ol, 1,7,7-trimethyl-, (1S-endo)- = Borneol	1179	1173	2.18	C_10_H_18_O
24	Terpinen-4-ol	1186	1176	0.7	C_10_H_18_O
25	m-Cymen-8-ol	1192	1187	0.35	C_10_H_14_O
26	Cyclohexene, 1-methyl-3-(1-methylethenyl)-	1200	-	0.78	C_10_H_16_
27	Bicyclo[3.1.1]hept-2-ene-2-ethanol, 6,6-dimethyl- = Nopol	1208	1212	0.22	C_11_H_18_O
28	Bicyclo[3.1.1]hept-3-en-2-one, 4,6,6-trimethyl- = Verbenone	1213	1207	0.32	C_10_H_14_O
29	8,9-Dehydrothymol	1226	1221.1	3.57	C_10_H_12_O
30	Pulegone	1246	1244	10.23	C_10_H_16_O
31	D-Carvone	1249	1254	0.65	C_10_H_14_O
32	Piperitenone oxide	1256	1330	15.74	C_10_H_14_O_2_
33	1,1’-Bicyclopentyl	1263	1080.9	0.17	C_10_H_18_
34	1-ethyl-3-methyl-2H-imidazole	1269	-	0.18	C_6_H_12_N_2_
35	(+)-Isopiperitenone	1274	-	0.99	C_10_H_14_O
36	4-Hydroxy-3-methylacetophenone	1283	1323	0.18	C_9_H_10_O_2_
37	Bicyclo[2.2.1]heptan-2-ol, 1,7,7-trimethyl-, acetate, (1S-endo)- = trans-Bornyl acetate	1288	1289	0.67	C_12_H_20_O_2_
38	Benzene, 1-ethoxy-4-ethyl-	1295	-	0.45	C_10_H_14_O
39	Thymol	1307	1306	0.36	C_10_H_14_O
40	3-Methyl-4-isopropylphenol = Biosol	1317	-	0.18	C_10_H_14_O
41	Durohydroquinone	1325	-	0.38	C_10_H_14_O_2_
42	2-Cyclohexen-1-one, 3-methyl-6-(1-methylethylidene)- = Piperitenone	1353	1349	34.44	C_10_H_14_O
43	(-)-β-Bourbonene	1391	1394.5	0.26	C_15_H_24_
44	2-hydroxy-7-methoxy-4-methyl cyclohepta-2,4,6-trien-1-one	1404	-	2.61	C_9_H_10_O_3_
45	Caryophyllene	1429	1431.8	2.33	C_15_H_24_
46	beta-Farnesene	1453	-	0.29	C_15_H_24_
47	Humulene = α-Caryophyllene	1464	1477	0.36	C_15_H_24_
48	1H-Cyclopenta[1,3]cyclopropa[1,2]benzene, octahydro-7-methyl-3-methylene-4-(1-methylethyl)-, [3aS-(3aα,3bβ,4β,7α,7aS*)]- = β-Cubebene	1488	1434	0.65	C_15_H_24_
49	Bicyclo[8.1.0]undeca-2,6-diene, 3,7,11,11-tetramethyl-, (1R*,2Z,6E,10R*)-(.+-.)-	1503	-	0.19	C_15_H_24_
50	3-Chloro-4-t-butyl-6-methylpyridazine	1573	-	0.74	C_9_H_13_ClN_2_
51	(-)-Spathulenol	1587	1582	0.8	C1_5_H_24_O
52	Caryophyllene oxide	1593	1598	2.71	C1_5_H_24_O
53	(1R,3E,7E,11R)-1,5,5,8-Tetramethyl-12-oxabicyclo[9.1.0]dodeca-3,7-diene = α-Humulene epoxide II	1620	1610	0.23	C1_5_H_24_O
54	Isospathulenol	1641	1640	0.27	C1_5_H_24_O
55	Caryophylla-4(12),8(13)-dien-5-β-ol = α-Caryophylladienol	1647	1637	0.44	C1_5_H_24_O
56	Presilphiperfolane-9,15-epoxide	1665		0.34	C1_5_H_24_O
57	Caryophyllenol-II	1679	1676	0.47	C1_5_H_24_O
58	12-norcyercene-B	1700	-	0.21	C_13_H_16_O_3_
59	2-Pentadecanone, 6,10,14-trimethyl-	1841	1846.7	0.15	C_18_H_36_O
60	n-Hexadecanoic acid	1961	1964	0.23	C_16_H_32_O_2_
61	1H-Naphtho[2,1-b]pyran, 3-ethenyldodecahydro-3,4a,7,7,10a-pentamethyl-, [3R-(3α,4aβ,6aα,10aβ,10bα)]- = Manoyl oxide	2006	2015	0.23	C_20_H_34_O
	**Total**			99.97	
	Monoterpenes hydrocarbons			6.01	
	Oxygenated monoterpenes			61.88	
	Sesquiterpenes hydrocarbons			4.08	
	Oxygenated sesquiterpenes			5.26	
	Others (Nonterpenoids)			22.74	

**Table 7:** Chemical compositions of *M*. *longifolia* leaf essential oil under SF.

no.	Compound	RI*	RI	Mean (%) ± SD	Molecular formula
1	Cyclohexane	643	654.9	46.73	C_6_H_12_
2	Toluene	700	762	31.53	C_7_H_8_
3	Octane	900	-	2.23	C_8_H_18_
4	2-Propenal, 2-methyl-3-phenyl- = α-Methylcinnimal	1221	-	2.48	C_10_H_10_O
5	2-Cyclohexen-1-one, 3-methyl-6-(1- = Piperitenone	1344	1344	2.25	C_10_H_14_O
6	Hexanedioic acid	1860	-	12.69	C_22_H_42_O_4_
7	n-Hexadecanoic acid	1961	1964	2.10	C_16_H_32_O_2_
8	Total			100.01	
9	Monoterpenes hydrocarbons				
10	Oxygenated monoterpenes			2.25	
11	Sesquiterpenes hydrocarbons				
12	Oxygenated sesquiterpenes				
15	Others (Nonterpenoids)			97.76	

The results showed that the number of compounds of the essential oil of *M*. *longifolia* leaf was 50 (99.79%) by HDC. The review of the studies indicates that the number and percentage of *M*. *longifolia* essential oil compounds with HDC from different regions have different values. [45], 26–33 compounds from habitats of Fars province, [35], 43 compounds from Egypt, [29], 46 compounds from Al-Baha city, Egypt, [64], 39 compounds From Giri River near Yashwant Nagar, Himachal Pradesh, India, [28] 12 compounds from Babol, [47] 24 compounds from Kamoo. Also, the number of essential compounds of *M*. *longifolia* leaf by HDM was 55 (100 percent), which is not consistent with the results of [65] with 13 compounds from Qazaan region by HDM. The difference in the number and type of compounds in different regions can be caused by the difference in climatic conditions, altitude above sea level, the topography of the region and the type of soil and the phenological stage of the plant [66].

According to the results, oxygenated monoterpenes were the dominant group of essential oils in all the studied extraction methods, which the highest and lowest amounts belonged to the HDC (63.54%) and the SF (2.25%), respectively. Previous studies show that oxygenated monoterpenes have always been the main group of *M*. *longifolia* essential oil compounds under different classical and modern extraction methods [62, 31]. The highest amount of oxygenated monoterpenes was reported for *M*. *longifolia* essential oil from Lorestan region with a value of 92.03% [45]. Oxygenated monoterpenes by [34] from Kashan with an amount of 89.47%, by [64] with an amount of 87.95% from Egypt and by [47] with an amount of 29.81% from Kamoo has been registered as the dominant group of compounds of this essential oil.

The results of ANOVA showed that different extraction methods had a significant effect on the amount of different compounds of *M*. *longifolia* essential oil (p≤0.01). Studies have confirmed that the extraction method has a significant effect on the chemical compounds of essential oils [67]. Piperitenone (25.2–41.38%), piperitenone oxide (22.02–0%), pulegone (10.81–0%) and 1,8-Cineole (5–35.0%) are the dominant and main components of *M*. *longifolia* leaf essential oil were subjected to different extraction methods. [31] reported the main components of *M*. *longifolia* essential oil with different methods of HDC, SF and LS were carvone (52.81–33.07%), limonene (30.10–0.23%), and trans-caryophyllene (2.51–4.87%), [61] reported pulegone (41.4–64.0%), 1,8-cineole (21.7–11.7%), piperitanone (0.6–4.5%), and p-menth-3-en-8-ol (5–2.7%) as the main components of *M*. *longifolia* essential oil under HDC and static headspace methods. [47] reported oxygenated monoterpenes such as piperitenone and piperitenone oxide as the dominant components of the essential oil of this species from the Triol region of Spain by SDE. The existence of some differences in our results with previous studies, such as the decrease or change in terms of the amount and type of different compounds of this essential oil, is probably due to the difference in the environmental conditions of the habitat and extraction conditions [62].

Based on the results in *M*. *longifolia* leaf essential oil extracted by HDC, the dominant compounds include piperitenone (37.77%), piperitenone oxide (15.94%), pulegone (10.47%), 1,8-Cineole (5.35%), 8,9-Dehydrothymol (3.35%), and 4,6-Diethyl-2-methoxypyrimidine (3.27%). The main compounds of this essential oil were determined by the HDC by [47] from Kamoo, 1,8-cineole (37.16%), piperitenone oxide (18.97%), sabinene (13.94%), α-pinene (8.92%), and pulegone (6.14%), by [28] from Babol, cis-piperitenone oxide (67.064%), piperitenone oxide (9.135%), L-menthone (5.745%), trans caryophyllene (5.271%), and 1,8-cineole (3.524%), and by [64] from India, piperitone oxide (53.83%), piperitenone oxide (11.52%), thymol (5.80%), and (E)-caryophyllene (4.88%) has been reported. The diversity of the chemical composition of the essential oil of a species in different regions indicates different chemotypes [68].

Piperitenone was the dominant and first compound of *M*. *longifolia* leaf essential oil extracted by all methods (except SF method with 2.25%). The highest amount of this compound belonged to the sample extracted by HDM (38.41%). Similarly, this composition was confirmed by [29] with 30.77% from Al-Baha city and by [69], with 29.3% from Al-Shatar, Lorestan as the first composition of the essential oil of this species under HDC, which is less than the amount was present in the study. The amount of piperitenone in *M*. *longifolia* essential oil was recorded by [65] under HDM from Marivan region (4.30%) and by [61] under HDC (4.50%) as the third dominant compound. In other previous studies, the amount of piperitenone was mostly insignificant and was one of the subcomponents of this essential oil. For example, [62] under SDK (0.77%), ([31] under HDC (0.27%), SF (1.03%), and lipophilic solvent (1.50%), [35] under HDC (1.83%), and [64] under HDC (0.17%) reported.

Piperitenone oxide was the second dominant compound of *M*. *longifolia* leaf essential oil in all different extraction methods, but it was not observed at all in the sample extracted by SF. The highest amount of this compound was observed in the essential oil extracted by HDM (22.02%). Similarly, [65] reported this composition with a value of 23.53% as the second dominant composition of this essential oil under HDM from Ghazaan region. Also, piperitenone oxide from Shahrza with an amount of 26.71% [70], from Kamoo with an amount of 18.97% [47], from Larestan with an amount of 19.33% (45], and from Alshatar of Lorestan with an amount of 35 14% [69] was recorded as the second dominant compound of this essential oil by HDC. The highest amount of this compound was 62.91% by HDC and as the first compound of this essential oil from KrishiVigyan Kendra, Shikohpur, Haryana, India. [27, 62] identified this compound in the fifth position with a value of 8.73% for this essential oil by SDK. On the other hand, piperitenone oxide in some studies for essential oil *M*. *longifolia* has not been reported under different extraction methods [29, 31, 34, 35]. Piperitenone oxide is the main component (more than 50%) of many essential oils of *Mentha* sp. [71]. Piperitenone oxide currently used as a flavoring agent in various commercial products (such as creams, lotions, detergents, and other personal and household products). This compound has biological activities such as anti-parasitic [72], insecticidal [73], antibacterial, antiviral, and antifungal activities [74, 75].

The results showed that pulegone was the third compound of *M*. *longifolia* leaf essential oil extracted by different methods. The highest amount of this compound was observed in the sample of essential oil extracted by HDM (10.81%), and it was not observed at all in the sample extracted by SF. Previous studies indicate that pulegone is the first dominant compound of *M*. *longifolia* essential oil from Fars by HDC (53.44–25.36%), from Ghazaan and Marivan by HDM (48.29 and 81.45%), from Kashan by HDC (66.95%), from Isfahan by HDC (44.97 percent) and from Fars by HDC and static headspace (64% and 41.4%) [45, 61, 65]. Some studies also reported the absence of pulegone in *M*. *longifolia* essential oil under different extraction methods [29, 31]. Pulegone is a monoterpene ketone found in the leaves and flowers of a number of plants in the Lamiaceae family. [76]. Pulegone has various medicinal properties, such as antimicrobial activity against many strains [77], anti-inflammatory and analgesic activity [78], antihistamine and antipyretic [79]. It used for flavor foods, beverages and dental products as fragrance and medicines [80].

1,8-Cineole was another dominant compound of *M*. *longifolia* leaf essential oil in most extraction methods, it was often the fourth dominant compound (except HDM and SF). The highest amount of this compound was obtained by HDC (5.35%), and in the sample extracted by SDK, it was reduced to 3.27% and the sixth place. Similarly, [35] identified 1,8-cineole as the fourth compound (9.11%) for this essential oil by HDC. The highest amount of this compound by HDC by [69] from Khorramabad (22.05%), by [70] from Isfahan (13.82%), and [29] from Al-Baha city (14.85%) and has been reported as the second composition of this essential oil. The lowest amount of this compound is reported to be 0.10% from Egypt ([64]. 1,8-Cineole has multiple biological properties such as anti-inflammatory, antioxidant, mucolytic/secretory, bronchodilatory and antimicrobial effects [81]. In addition, recent studies have highlighted the neuroprotective, analgesic, and pro-apoptotic properties of 1,8-cineole, underscoring its potential beneficial role in a wide variety of conditions such as alzheimer’s disease, neuropathic pain, and cancer [82].

Cyclohexen with a value of 46.73% was the dominant and main compound of *M*. *longifolia* leaf essential oil by SF and was not observed in other methods. Ahmadpour [28] reported 1.14% of Cyclohexen for this essential oil from the Babol region by HDC. Cyclohexane is generally used as a chemical intermediate. Specifically, 54% of what is produced is used in the production of adipic acid for nylon 6.6, 39% for caprolactam for nylon-6, and 7% for products such as solvents, insecticides, and softeners. The demand for nylon (and thus cyclohexane) in engineering thermoplastics in resins and films is growing at about 6% annually [83]. *M*. *longifolia* has been found to contain cyclohexane and is aromatic, which has a specific aroma ranging from spicy to balsamic. [84]. The essential oils of *M*. *longifolia* extracted by SF are better than those extracted by other methods [85]. Therefore, it has been confirmed that the quality of the essential oil of the species depends on environmental factors, the time of ripening and then on the conditions of extraction and storage [86].

### 3-3-Antimicrobial activity

The results of ANOVA showed that there was a significant difference between antimicrobial activity by agar diffusion method, MIC and MBC values of essential oils and control antibiotics against the studied bacteria (*Staphylococcus aureus*, *Shigella dysenteriae*, and *Escherichia coli*) (P≤ 0.01) (Tables 8 and 9). Based on the results of *M*. *longifohia* leaf essential oil, they did not create any inhibition zone against the studied strains. Similarly, [65] reported the absence of inhibition zone by *M*. *longifolia* essential oil extracted by HDM from Marivan region against *S*. *aureus*, *S*. *dysenteriae* and *E*. *coli*. This similarity in terms of lack of inhibitory activity can be due to different mechanisms of antimicrobial activity of essential oils [87]. The diameter of the inhibition zone of *M*. *longifolia* essential oil extracted by HDC by [34] against *E*. *coli* (14 mm) and by [29] against *S*. *aureus* (14 mm) and *E*. *coli* (24 mm) has been reported. The variation in the inhibitory activity of the essential oils of one species in different regions is due to the difference in the chemical composition of the essential oils in different environmental conditions [88].

**Table 8:** Antibiogram results of antibiotic controls on the tested clinical strains.

Antibiotics	Rifampin			Gentamicin		
	DZ (mm)	MIC (μg/mL)	MBC (μg/mL)	DZ (mm)	MIC (μg/mL)	MBC (μg/mL)
*Staphylococcus aureus*	36	3.9	3.9	31	7.8	7.8
*Escherichia coli*	10	15.63	15.63	23	31.25	31.25
*Shigella dysenteriae*	9	15.63	15.63	17	3.90	3.90

**Table 9:** Antimicrobial activity of *M*. *longifolia* leaf essential oil under different extraction methods on tested clinical strains.

Test microorganism	HDC			SF			U+HDC			SDE			SDK			HDM		
	DZ (mm)	MIC (μg/mL)	MBC (μg/mL)	DZ (mm)	MIC (μg/mL)	MBC (μg/mL)	DZ (mm)	MIC (μg/mL)	MBC (μg/mL)	DZ (mm)	MIC (μg/mL)	MBC (μg/mL)	DZ (mm)	MIC (μg/mL)	MBC (μg/mL)	DZ (mm)	MIC (μg/mL)	MBC (μg/mL)
*Staphylococcus* *aureus*	―	2000	2000	―	4000	>4000	―	2000	2000	―	4000	>4000	―	2000	2000	―	2000	2000
*Escherichia coli*	―	4000	4000	―	2000	4000	―	2000	2000	―	2000	2000	―	2000	2000	―	2000	2000
*Shigella dysenteriae*	―	2000	2000	―	2000	2000	―	1000	2000	―	1000	2000	―	1000	2000	―	1000	2000

On the other hand, the findings of the minimum concentration of lethality and inhibition by the microdilution method showed that MIC and MBC values of *M*. *longifohia* leaf essential oil against all studied microorganisms ranged from 1000 to 4000 μg/mL and from 2000 to 4000 μg/mL, which had performed very poorly compared to the control antibiotics. The lowest MIC value of *M*. *longifohia* leaf essential oil extracted by HDM, SDK, SDE and U+HDC with a value of 1000 μg/mL was observed against the Gram-negative *Shigella dysenteriae*, which is 5 times weaker than rifampin MIC =  15.63 μg/mL) and 7 times weaker than gentamicin (MIC =  3.90 μg/mL). The lowest MIC value of *M*. *longifohia* essential oil against *Sh*. *dysenteriae* was recorded by [65] with a value of 250 μg/mL, which was extracted by HDM. The weaker antimicrobial activity of essential oils extracted by the HDC and SF is due to the fact that the compounds are difficult to diffuse, and those with high hydrophobicity have little effect on the antimicrobial activity of the resulting oils. [89]. Distillation with water (HDC) requires a large amount of water and higher temperature, which causes hydrolysis reaction and damage to the active compound. In the SF, it will bring some miscellaneous materials such as wax and pigment. These substances may be the reason for the lower inhibitory effect of the essential oil extracted by this method [90]. Similarly, [50], for the essential oil of *Artemisia argyi* Lévl. et Vant reported the superior activity of SDE essential oil compared to SF and HDC.

The difference in the amount of antibacterial effects observed in this study and other researches can be due to the difference in the growth places of the plant and the use of different methods for extraction, etc. The difference in antimicrobial effects indicates the differences in the composition of essential oils [91]. Therefore, it seems that the predominance of oxygenated monoterpene compounds such as piperitenone and pulegone, 1,8-Cineole, as well as piperitenone oxide in *M*. *longifohia* leaf essential oil samples under water distillation extraction methods by HDM, SDK, SDE and U+HDC is one of the main factors of this antibacterial activity. Similarly, [69] related the antibacterial activity of *M*. *longifohia* essential oil to oxygenated monoterpenes, especially piperitenone and pulegone. Oxygenated monoterpenes in nature are lipophilic and play their role in the cell membrane and cause many morphological damages, which eventually change the permeability of the membrane and release the cell contents [92, 93]. If membrane integrity is disrupted, its function not only as a barrier, but also as a matrix for enzymes and as an energy converter is compromised. However, the specific mechanisms involved in the antimicrobial action of monoterpenes are not well defined [94]. Piperitenone and pulegone cause disruption of the structure of different layers of polysaccharides, fatty acids and phospholipids in the bacterial membrane by altering the cell membrane and destroying the bacterial wall [95]. The effect of pulegone against *S*. *aureus*, *S*. *typhimurium*, and *E*. *coli* has been confirmed [96]. The effect of low activity of 1,8-Cineole on *Shigella sp*. confirmed [97].

*Sh*. *dysenteriae* is the predominant cause of dysentery and a major public health problem in many countries [98]. Plant essential oils can prevent the growth of microorganisms through various mechanisms such as affecting the cell wall, preventing the production of proteins, preventing the function of the cytoplasmic membrane, etc. Gram-negative bacteria are usually less sensitive to the effects of essential oils than Gram-positive bacteria because of their outer membrane that surrounds the cell wall and limits the diffusion of hydrophobic compounds through its polysaccharide coating. It seems that this effect is dependent on the lipid composition and net surface charge of microbial membranes [99].

## 4-Conclusion

It is important to use practical methods to obtain essential oils with higher quality due to their large use in the industry. Today, with the new methods of extracting essential oils, scientists are looking for increasing the stability of essential oils and increasing their solubility in solvents with low percentage of alcohol in food and water, as well as reducing storage and transportation costs of essential oils. The extraction method of plant essential oils can change the extraction efficiency, the percentage and type of chemical compounds and as a result the biological activities in it. By comparing the different characteristics of *M*. *longifolia* leaf essential oil extracted under different traditional and modern methods, this research showed that the extraction method affects the efficiency, type and percentage of chemical compounds and antimicrobial activity of the essential oil of this species. It seems that the traditional HDC and modern HDM method were the best methods to obtain higher yields and higher percentage chemical compounds. Although there was not much difference between the methods in terms of antimicrobial activity, but the HDM, with less time and more efficiency, can be a good option for producing essential oil with higher quality and promising potential antimicrobial effects. Therefore, with the targeted selection of the extraction method, the essential oil of this species can be approached as a suitable option for use in various related industries.

## Supporting information

S1 File (ZIP)

S2 File (ZIP)
