# Supplementary material for: Comparison of quantity, quality and antibacterial activity of essential oil Mentha longifolia (L.) L. under different traditional and modern extraction methods
Source: PLoS One. 2024 Jul 10;19(7):e0301558. doi: 10.1371/journal.pone.0301558 (PMC11236116; doi:10.1371/journal.pone.0301558)
Supplement: S1 File — (ZIP) [file pone.0301558.s001.zip › Karimnezhad/PrintText+summery.pdf]

Library Search Report

Data Path : D:\msdchem\1\data\
Data File : Karimnezhad.D
Acq On : 22 Feb 2022 18:58
Operator : Jafari
Sample : 1
Misc :
ALS Vial : 23 Sample Multiplier: 1

Search Libraries: D:\Database\W10N14.L Minimum Quality: 0

Unknown Spectrum: Apex
Integration Events: ChemStation Integrator - events.e

Pk#	RT	Area%	Library/ID	Ref#	CAS#	Qual
1	5.390	46.73	D:\Database\W10N14.L			
			Cyclohexane	4621	000110-82-7	91
			Cyclohexane	4626	000110-82-7	91
			Cyclohexane	4619	000110-82-7	90
2	7.362	31.53	D:\Database\W10N14.L			
			BENZENE, METHYL-	7602	000108-88-3	91
			Benzene, methyl- (CAS)	7611	000108-88-3	91
			Toluene	7614	000108-88-3	91
3	8.128	2.23	D:\Database\W10N14.L			
			Octane	22544	000111-65-9	91
			OCTANE	22537	000111-65-9	86
			Octane	22539	000111-65-9	86
4	26.760	2.48	D:\Database\W10N14.L			
			(2E)-2-METHYL-3-PHENYL-2-PROPENAL	65175	000101-39-3	87
			8,9-Dehydrothymol	68975	018612-99-2	83
			Benzaldehyde, 4-(1-methylethenyl)-	65305	010133-50-3	49
5	32.406	2.25	D:\Database\W10N14.L			
			2-Cyclohexen-1-one, 3-methyl-6-(1-methylethylidene)-	73417	000491-09-8	98
			2-Cyclohexen-1-one, 3-methyl-6-(1-methylethylidene)-	73419	000491-09-8	94
			2-Cyclohexen-1-one, 3-methyl-6-(1-methylethylidene)-	73416	000491-09-8	93
6	52.576	12.69	D:\Database\W10N14.L			
			Hexanedioic acid, dioctyl ester	725817	000123-79-5	97
			Hexanedioic acid, bis(2-ethylhexyl) ester	725748	000103-23-1	96
			Hexanedioic acid, bis(2-ethylhexyl) ester	725745	000103-23-1	95
7	55.942	2.10	D:\Database\W10N14.L			
			n-Hexadecanoic acid	387914	000057-10-3	99
			n-Hexadecanoic acid	387919	000057-10-3	98
			n-Hexadecanoic acid	387916	000057-10-3	98
