# Supplementary material for: Comparison of quantity, quality and antibacterial activity of essential oil Mentha longifolia (L.) L. under different traditional and modern extraction methods
Source: PLoS One. 2024 Jul 10;19(7):e0301558. doi: 10.1371/journal.pone.0301558 (PMC11236116; doi:10.1371/journal.pone.0301558)
Supplement: S1 File — (ZIP) [file pone.0301558.s001.zip › Karimnezhad/QualKarimnezhad.pdf]

Library Search Report

Data Path : D:\msdchem\1\data\
Data File : Karimnezhad.D
Acq On : 22 Feb 2022 18:58
Operator : Jafari
Sample : 1
Misc :
ALS Vial : 23 Sample Multiplier: 1

Search Libraries: D:\Database\W10N14.L Minimum Quality: 0

Unknown Spectrum: Apex
Integration Events: ChemStation Integrator - events.e

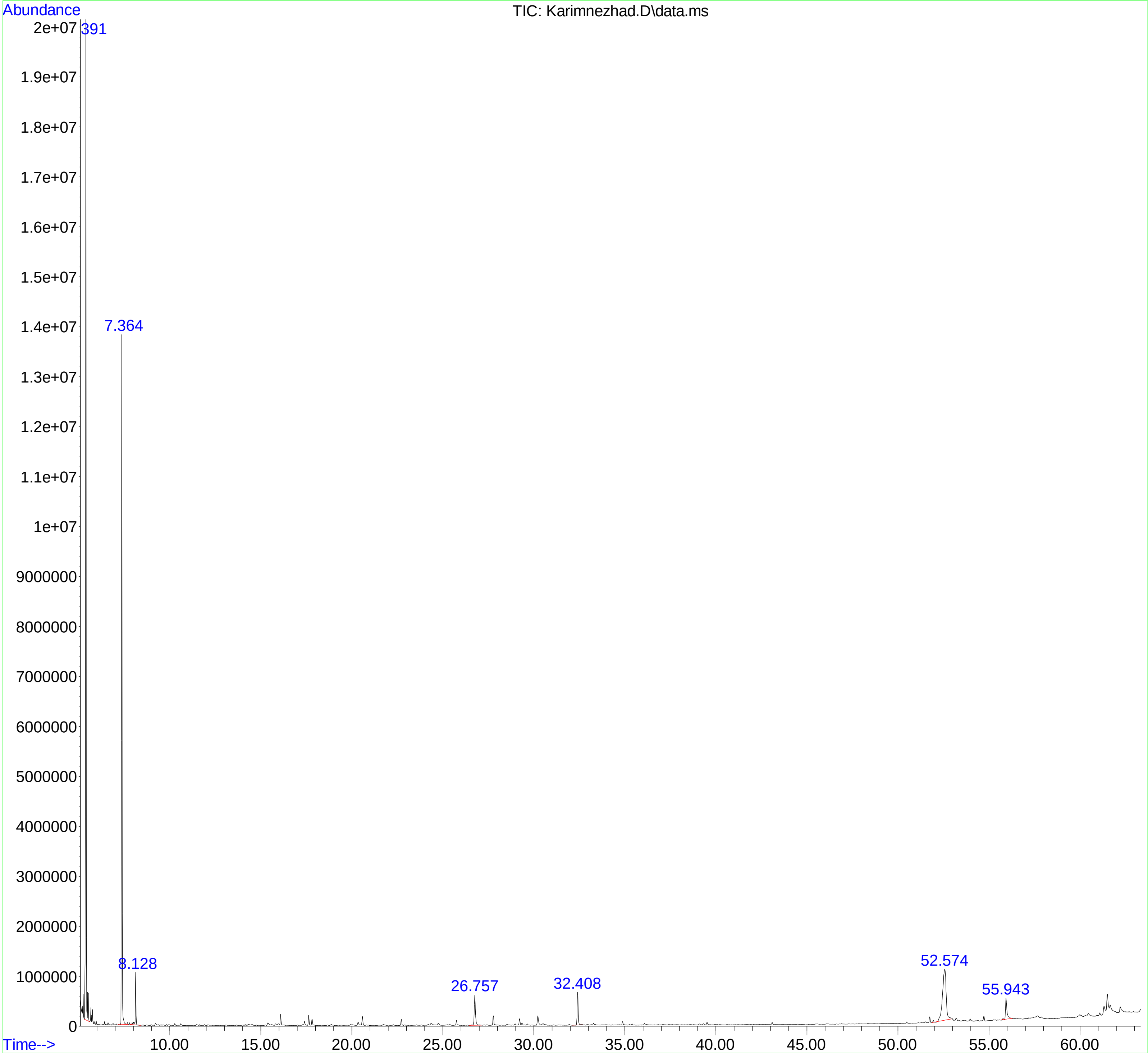

Unknown Spectrum based on Apex

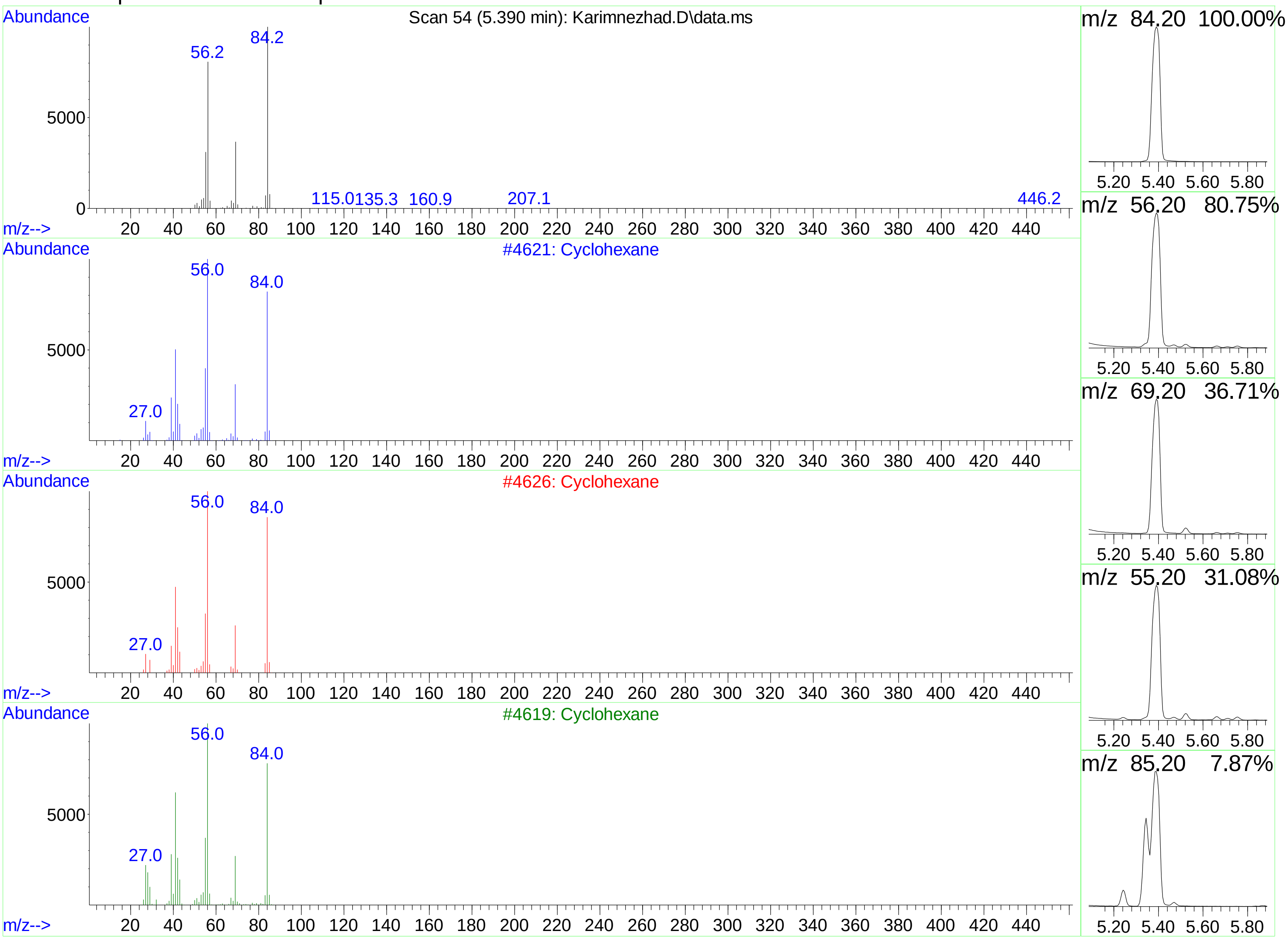

Data File: D:\msdchem\1\data\Karimnezhad.D
Sample : 1

Peak Number: 1 at 5.390 min Area: 524825449 Area % 46.73

The 3 best hits from each library.	Ref\#	CAS\#	Qual
D:\Database\W10N14.L			
1 Cyclohexane	4621	000110-82-7	91
2 Cyclohexane	4626	000110-82-7	91
3 Cyclohexane	4619	000110-82-7	90

Unknown Spectrum based on Apex

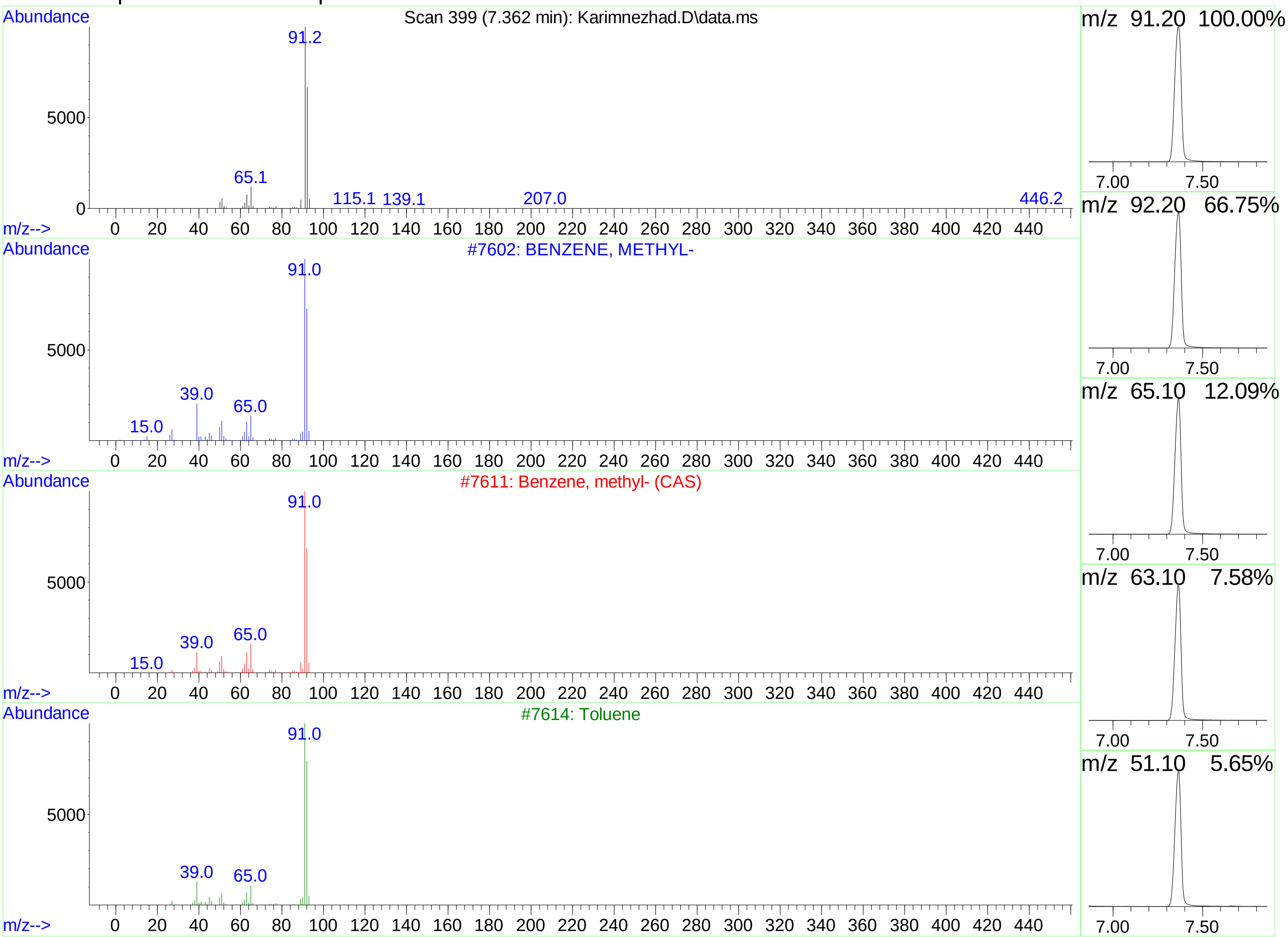

Data File: D:\msdchem\1\data\Karimnezhad.D
Sample : 1

Peak Number: 2 at 7.362 min Area: 354072827 Area % 31.53

The 3 best hits from each library.	Ref#	CAS#	Qual
D:\Database\W10N14.L			
1 BENZENE, METHYL-	7602	000108-88-3	91
2 Benzene, methyl- (CAS)	7611	000108-88-3	91
3 Toluene	7614	000108-88-3	91

Unknown Spectrum based on Apex

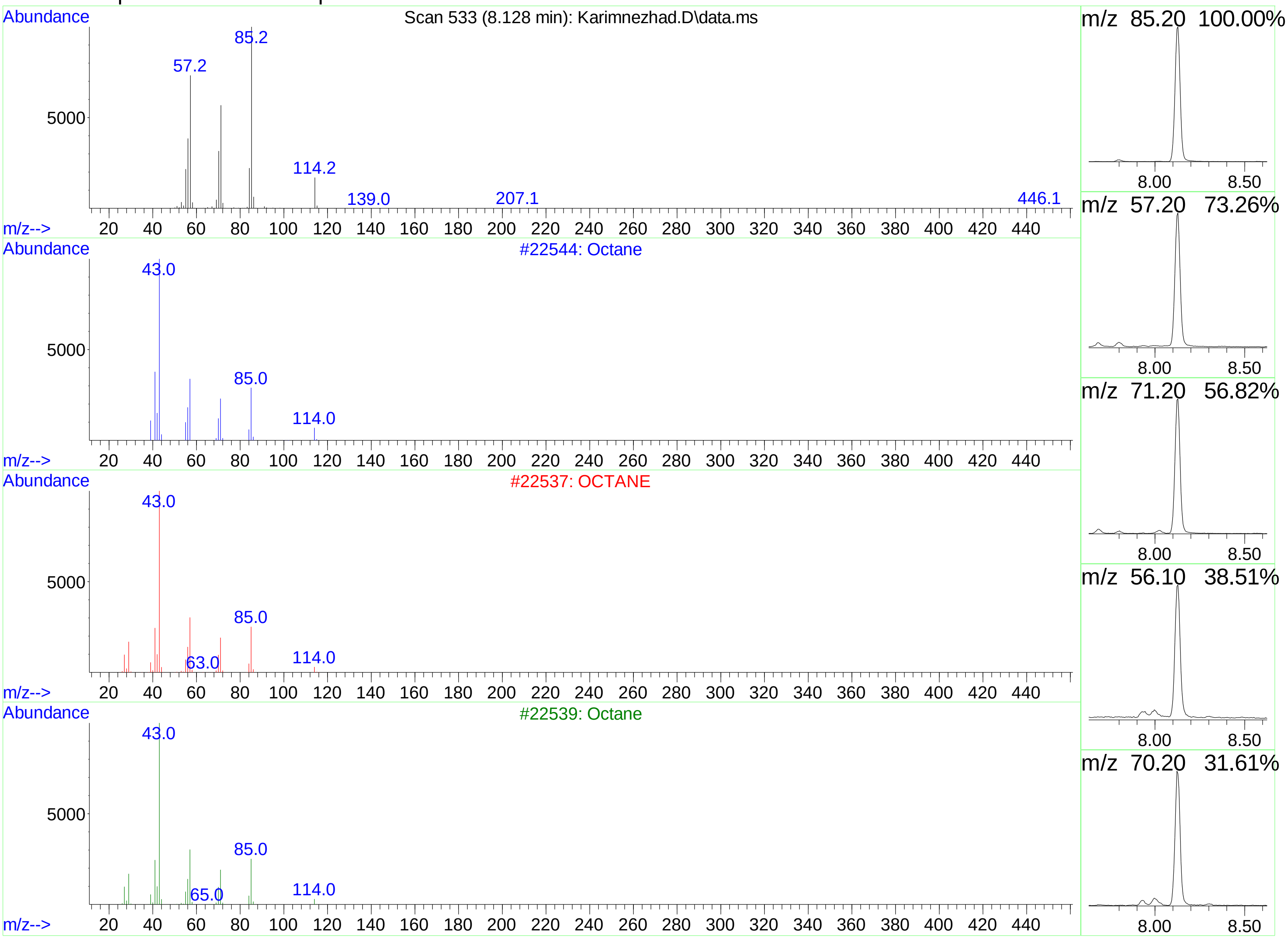

Data File: D:\msdchem\1\data\Karimnezhad.D
Sample : 1

Peak Number: 3 at 8.128 min Area: 25013353 Area % 2.23

The 3 best hits from each library.	Ref\#	CAS\#	Qual
D:\Database\W10N14.L			
1 Octane	22544	000111-65-9	91
2 OCTANE	22537	000111-65-9	86
3 Octane	22539	000111-65-9	86

Unknown Spectrum based on Apex

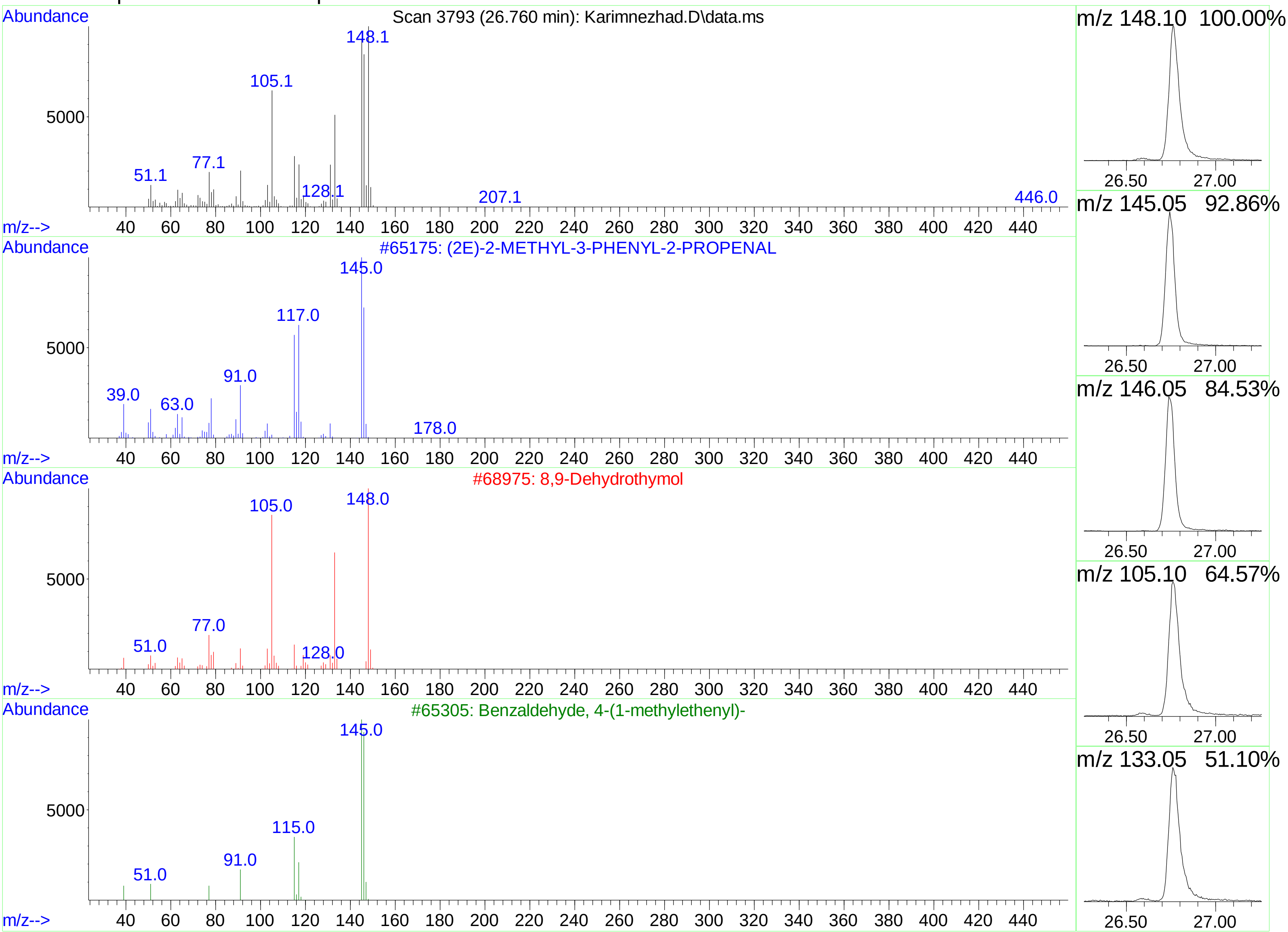

Data File: D:\msdchem\1\data\Karimnezhad.D
Sample : 1

Peak Number: 4 at 26.760 min Area: 27800320 Area % 2.48

The 3 best hits from each library.	Ref#	CAS#	Qual
D:\Database\W10N14.L			
1 (2E)-2-METHYL-3-PHENYL-2-PROPENAL	65175	000101-39-3	87
2 8,9-Dehydrothymol	68975	018612-99-2	83
3 Benzaldehyde, 4-(1-methylethenyl)-	65305	010133-50-3	49

Unknown Spectrum based on Apex

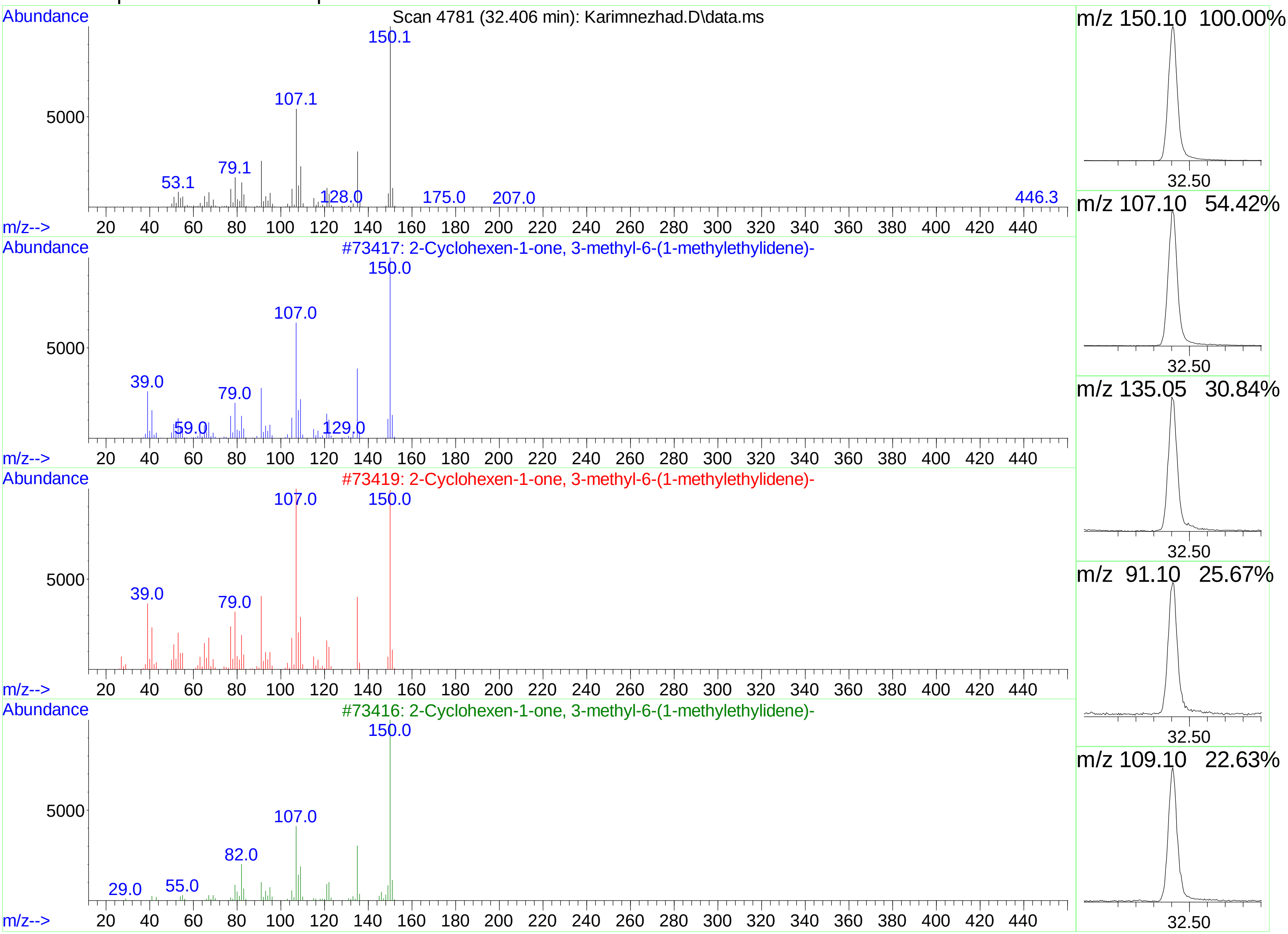

Data File: D:\msdchem\1\data\Karimnezhad.D
Sample : 1

Peak Number: 5 at 32.406 min Area: 25259162 Area % 2.25

The 3 best hits from each library.	Ref\#	CAS\#	Qual
D:\Database\W10N14.L			
1 2-Cyclohexen-1-one, 3-methyl-6-(...	73417	000491-09-8	98
2 2-Cyclohexen-1-one, 3-methyl-6-(...	73419	000491-09-8	94
3 2-Cyclohexen-1-one, 3-methyl-6-(...	73416	000491-09-8	93

Unknown Spectrum based on Apex

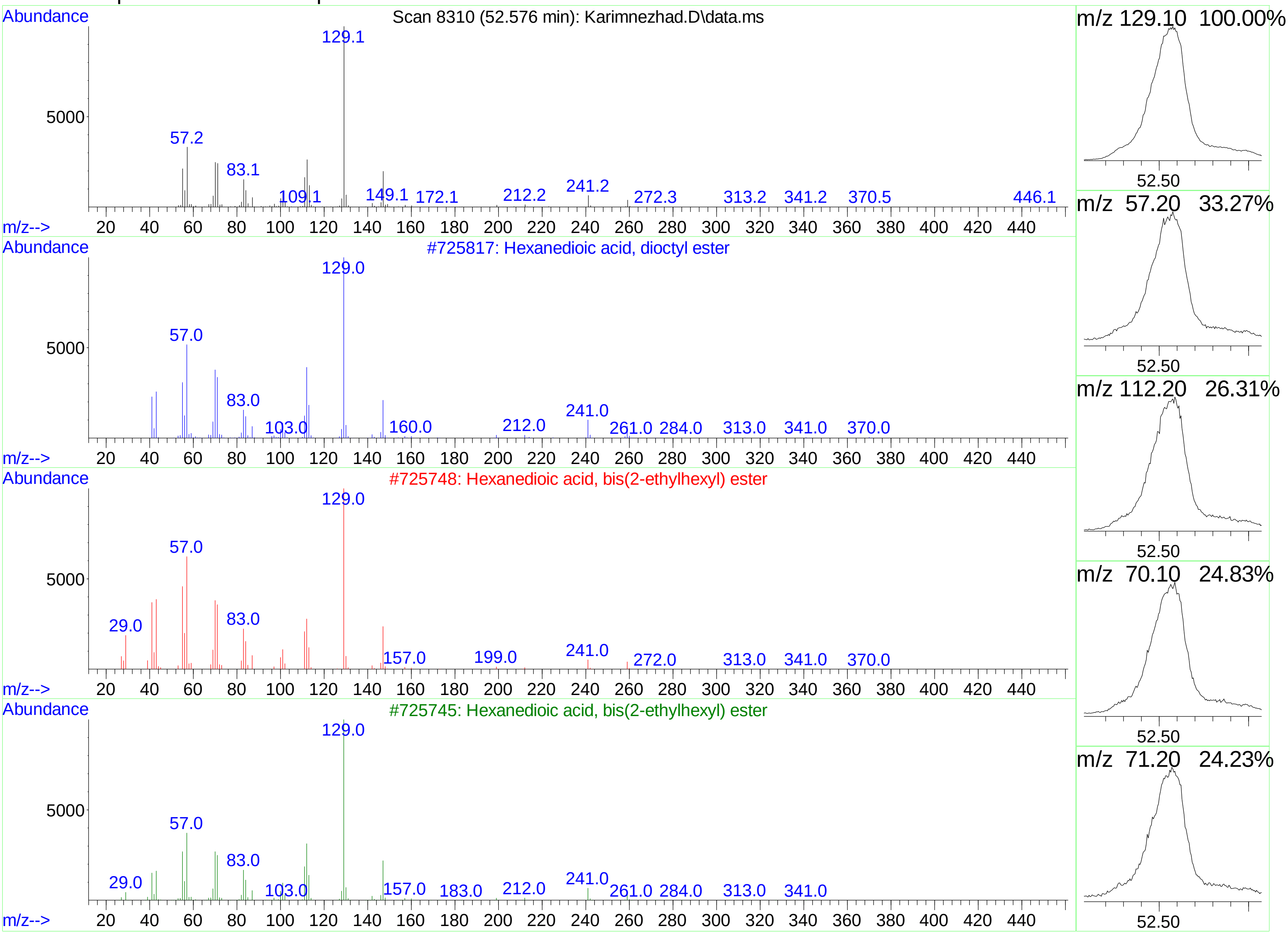

Data File: D:\msdchem\1\data\Karimnezhad.D
Sample : 1

Peak Number: 6 at 52.576 min Area: 142567078 Area % 12.69

The 3 best hits from each library.	Ref#	CAS#	Qual
D:\Database\W10N14.L			
1 Hexanedioic acid, dioctyl ester	725817	000123-79-5	97
2 Hexanedioic acid, bis(2-ethylhex...	725748	000103-23-1	96
3 Hexanedioic acid, bis(2-ethylhex...	725745	000103-23-1	95

Library Search Report - ChemStation Integrator

Unknown Spectrum based on Apex

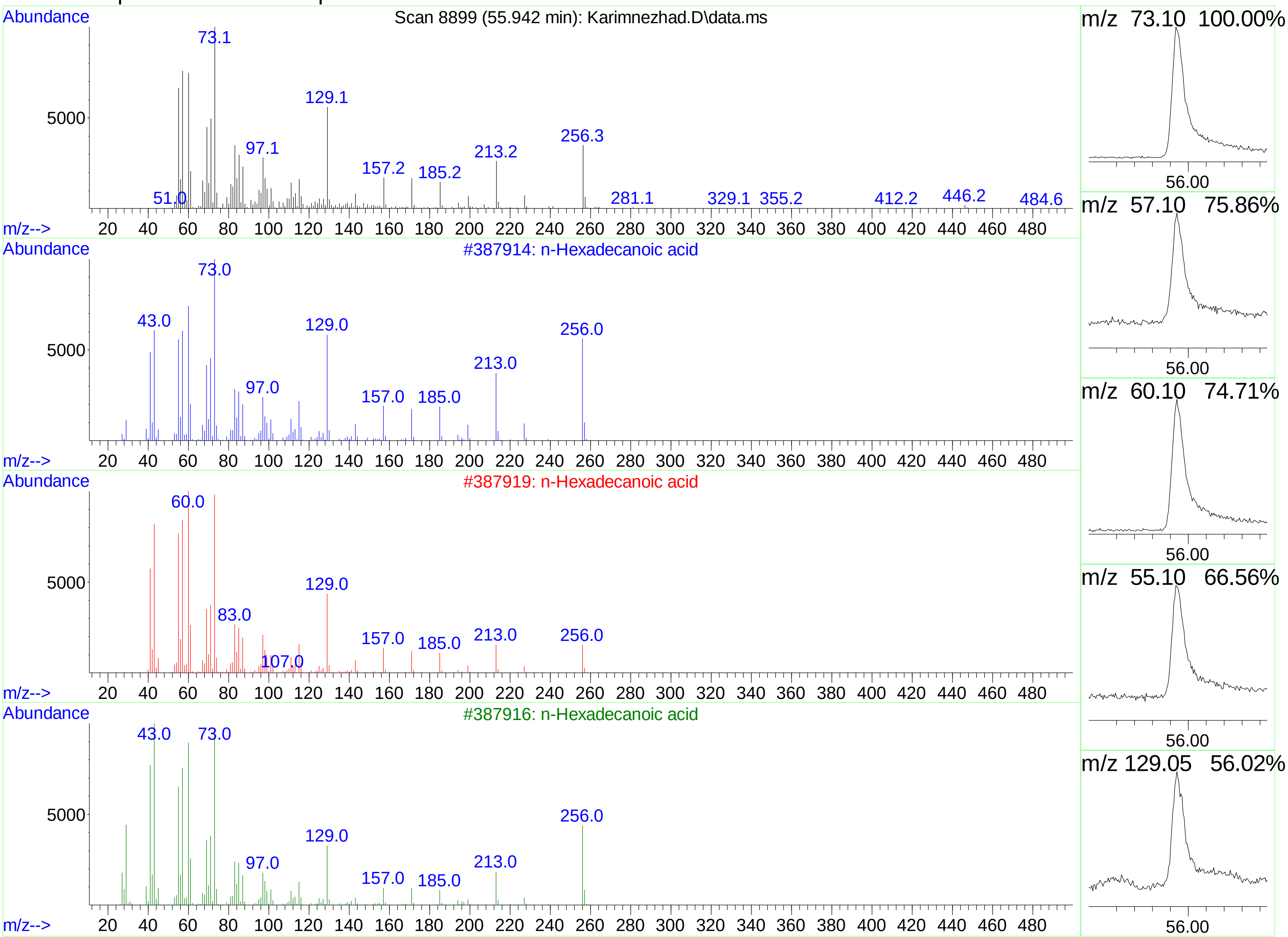

Data File: D:\msdchem\1\data\Karimnezhad.D
Sample : 1

Peak Number: 7 at 55.942 min Area: 23579437 Area % 2.10

The 3 best hits from each library.	Ref#	CAS#	Qual
D:\Database\W10N14.L			
1 n-Hexadecanoic acid	387914	000057-10-3	99
2 n-Hexadecanoic acid	387919	000057-10-3	98
3 n-Hexadecanoic acid	387916	000057-10-3	98
