# Supplementary material for: Comparison of quantity, quality and antibacterial activity of essential oil Mentha longifolia (L.) L. under different traditional and modern extraction methods
Source: PLoS One. 2024 Jul 10;19(7):e0301558. doi: 10.1371/journal.pone.0301558 (PMC11236116; doi:10.1371/journal.pone.0301558)
Supplement: S2 File — (ZIP) [file pone.0301558.s002.zip › Karimnezhad/SDE/QualKarimnezhad 1.pdf]

Library Search Report

Data Path : D:\msdchem\1\data\
Data File : Karimnezhad 1.D
Acq On : 15 Mar 2022 6:21
Operator : Jafari
Sample : SDE
Misc :
ALS Vial : 29 Sample Multiplier: 1

Search Libraries: D:\Database\W10N14.L Minimum Quality: 0

Unknown Spectrum: Apex
Integration Events: ChemStation Integrator - events.e

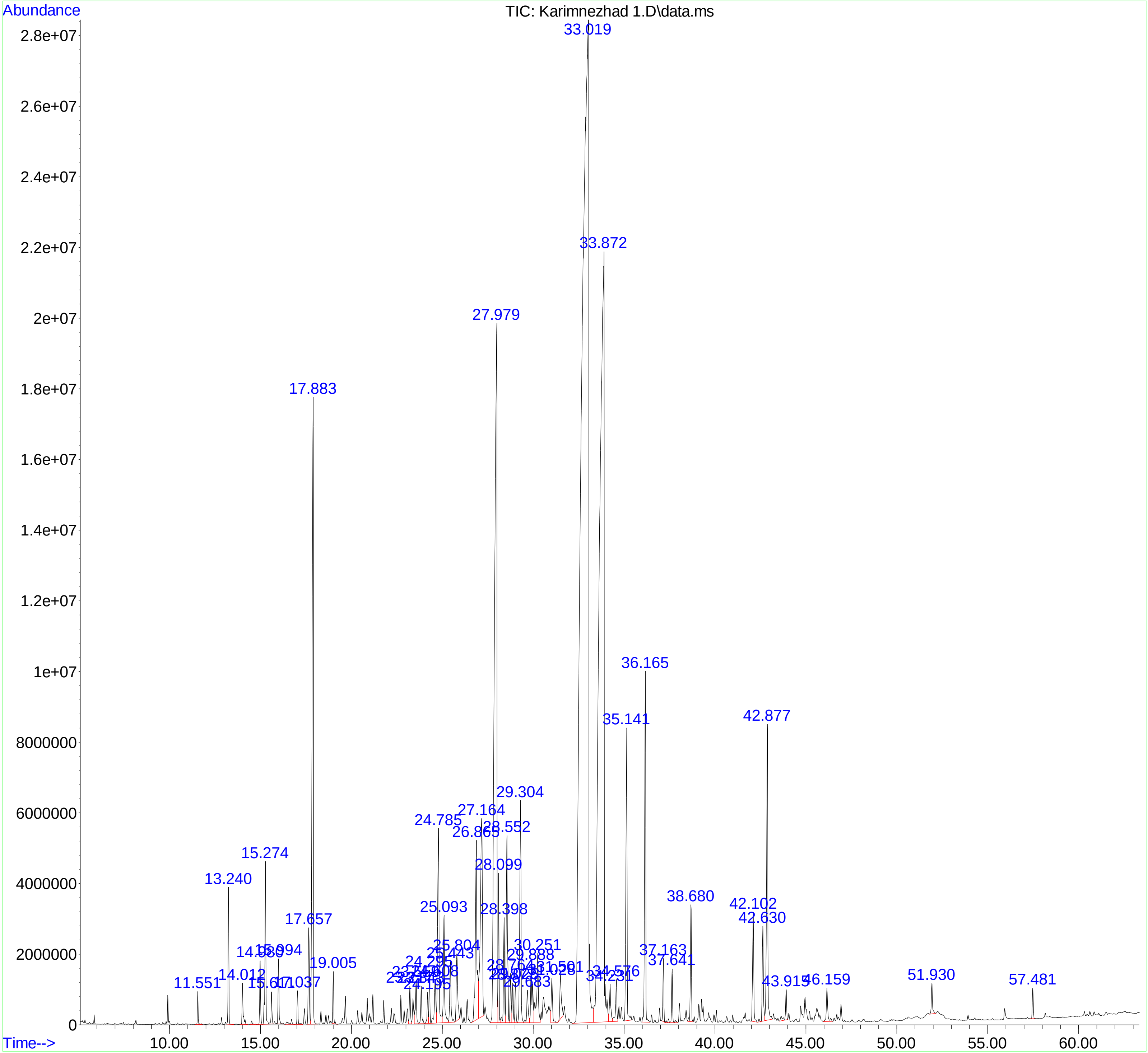

Unknown Spectrum based on Apex

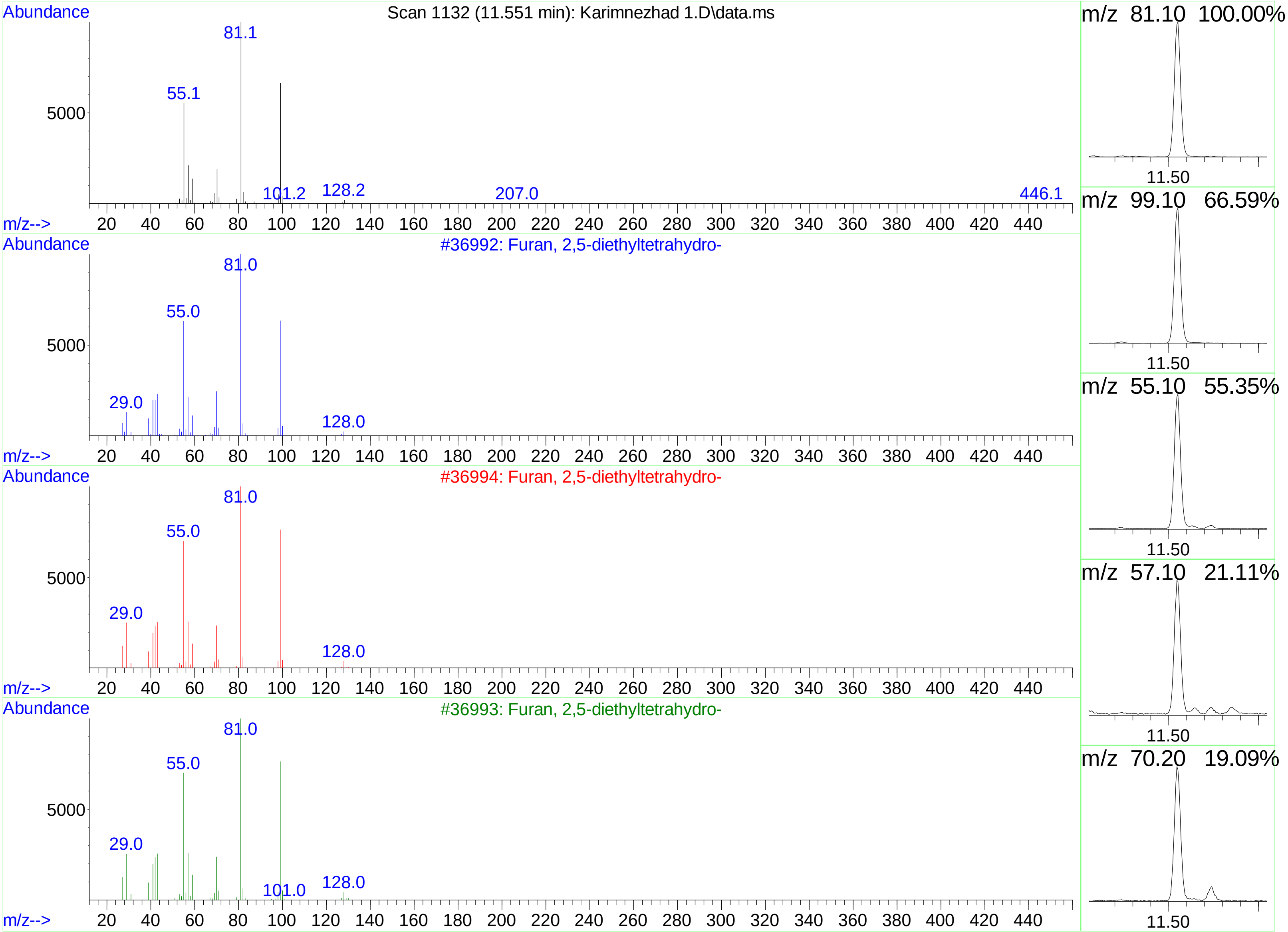

Data File: D:\msdchem\1\data\Karimnezhad 1.D
Sample : SDE

Peak Number: 1 at 11.551 min Area: 24540182 Area % 0.12

The 3 best hits from each library.	Ref#	CAS#	Qual
D:\Database\W10N14.L			
1 Furan, 2,5-diethyltetrahydro-	36992	041239-48-9	95
2 Furan, 2,5-diethyltetrahydro-	36994	041239-48-9	78
3 Furan, 2,5-diethyltetrahydro-	36993	041239-48-9	78

Unknown Spectrum based on Apex

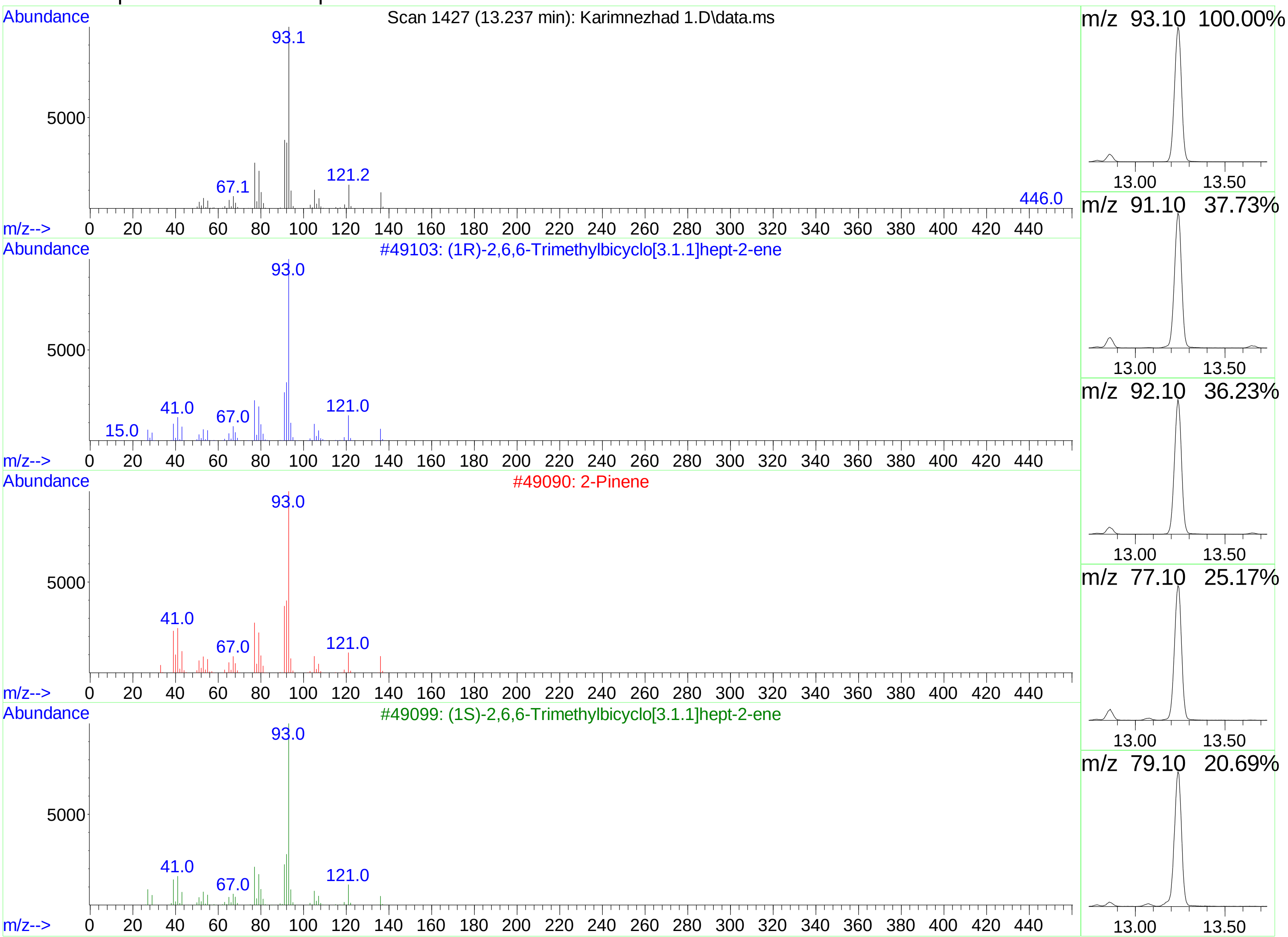

Data File: D:\msdchem\1\data\Karimnezhad 1.D
Sample : SDE

Peak Number: 2 at 13.237 min Area: 107135950 Area % 0.54

The 3 best hits from each library.	Ref#	CAS#	Qual
D:\Database\W10N14.L			
1 (1R)-2,6,6-Trimethylbicyclo[3.1....	49103	007785-70-8	96
2 2-Pinene	49090	000080-56-8	96
3 (1S)-2,6,6-Trimethylbicyclo[3.1....	49099	007785-26-4	96

Unknown Spectrum based on Apex

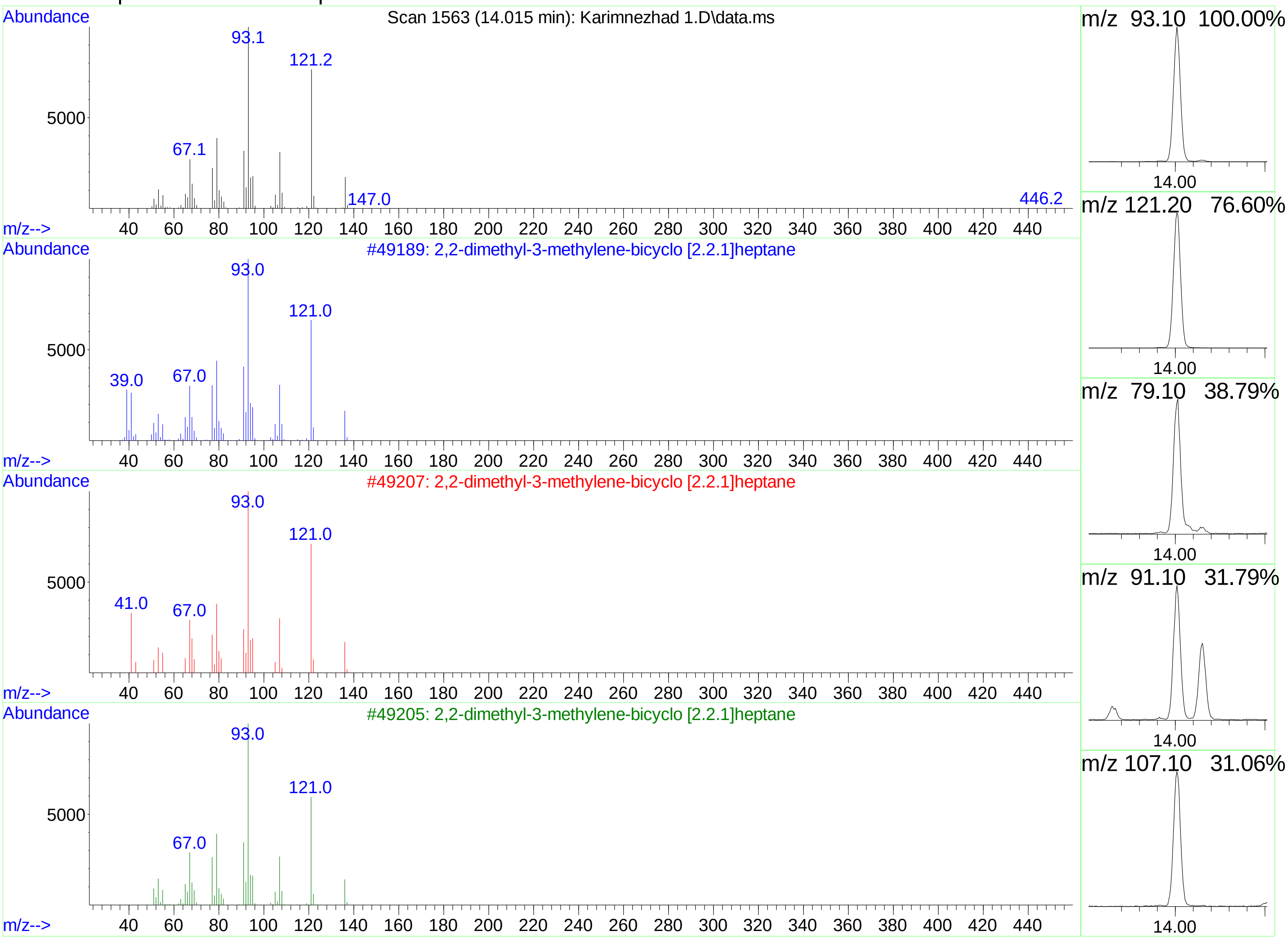

Data File: D:\msdchem\1\data\Karimnezhad 1.D
Sample : SDE

Peak Number: 3 at 14.015 min Area: 43376878 Area % 0.22

The 3 best hits from each library.	Ref#	CAS#	Qual
D:\Database\W10N14.L			
1 2,2-dimethyl-3-methylene-bicyclo...	49189	000079-92-5	97
2 2,2-dimethyl-3-methylene-bicyclo...	49207	000079-92-5	97
3 2,2-dimethyl-3-methylene-bicyclo...	49205	000079-92-5	97

Unknown Spectrum based on Apex

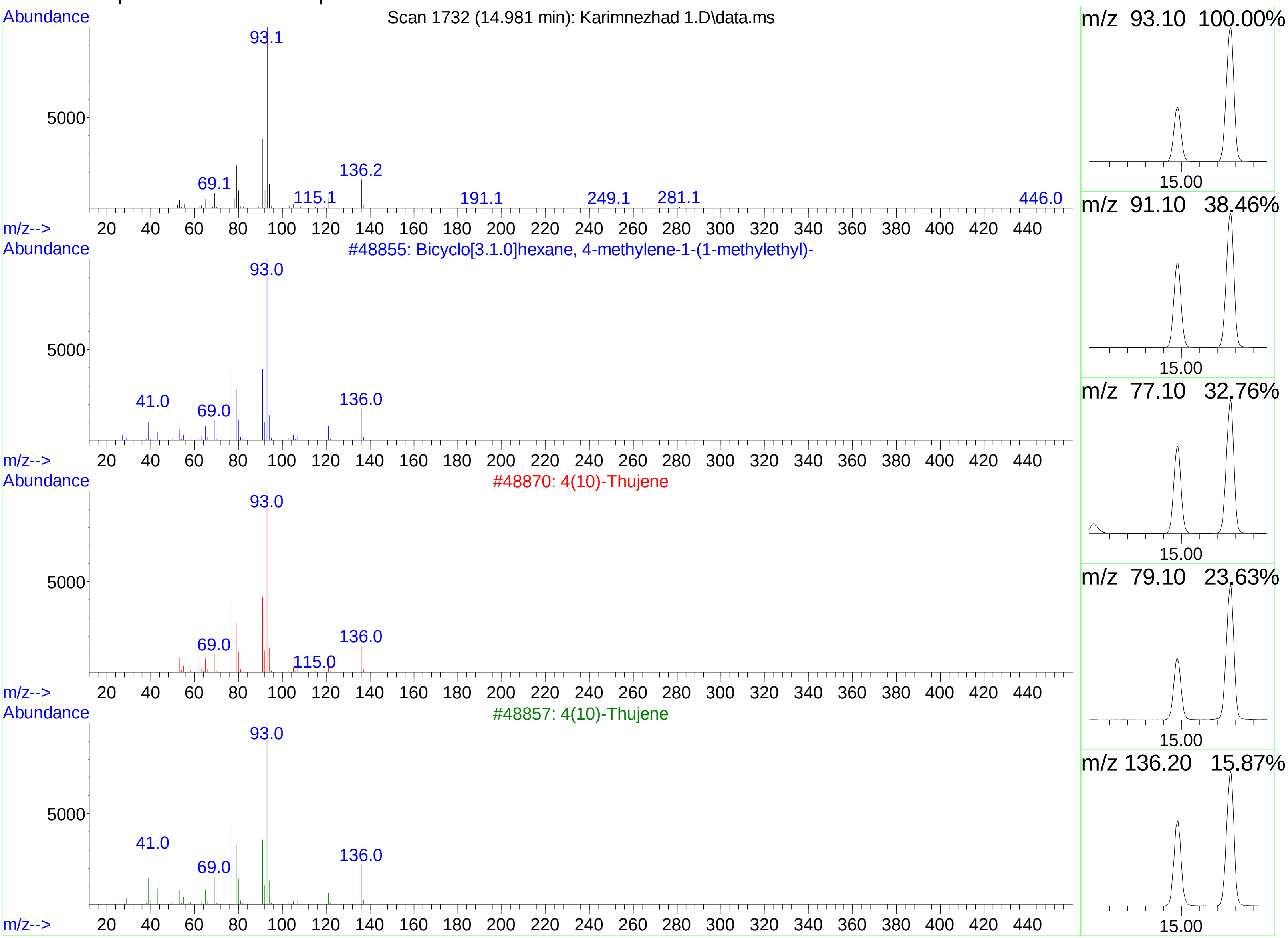

Data File: D:\msdchem\1\data\Karimnezhad 1.D
Sample : SDE

Peak Number: 4 at 14.981 min Area: 60007289 Area % 0.30

The 3 best hits from each library.	Ref#	CAS#	Qual
D:\Database\W10N14.L			
1 Bicyclo[3.1.0]hexane, 4-methylen...	48855	003387-41-5	96
2 4(10)-Thujene	48870	003387-41-5	96
3 4(10)-Thujene	48857	003387-41-5	96

Library Search Report - ChemStation Integrator

Unknown Spectrum based on Apex

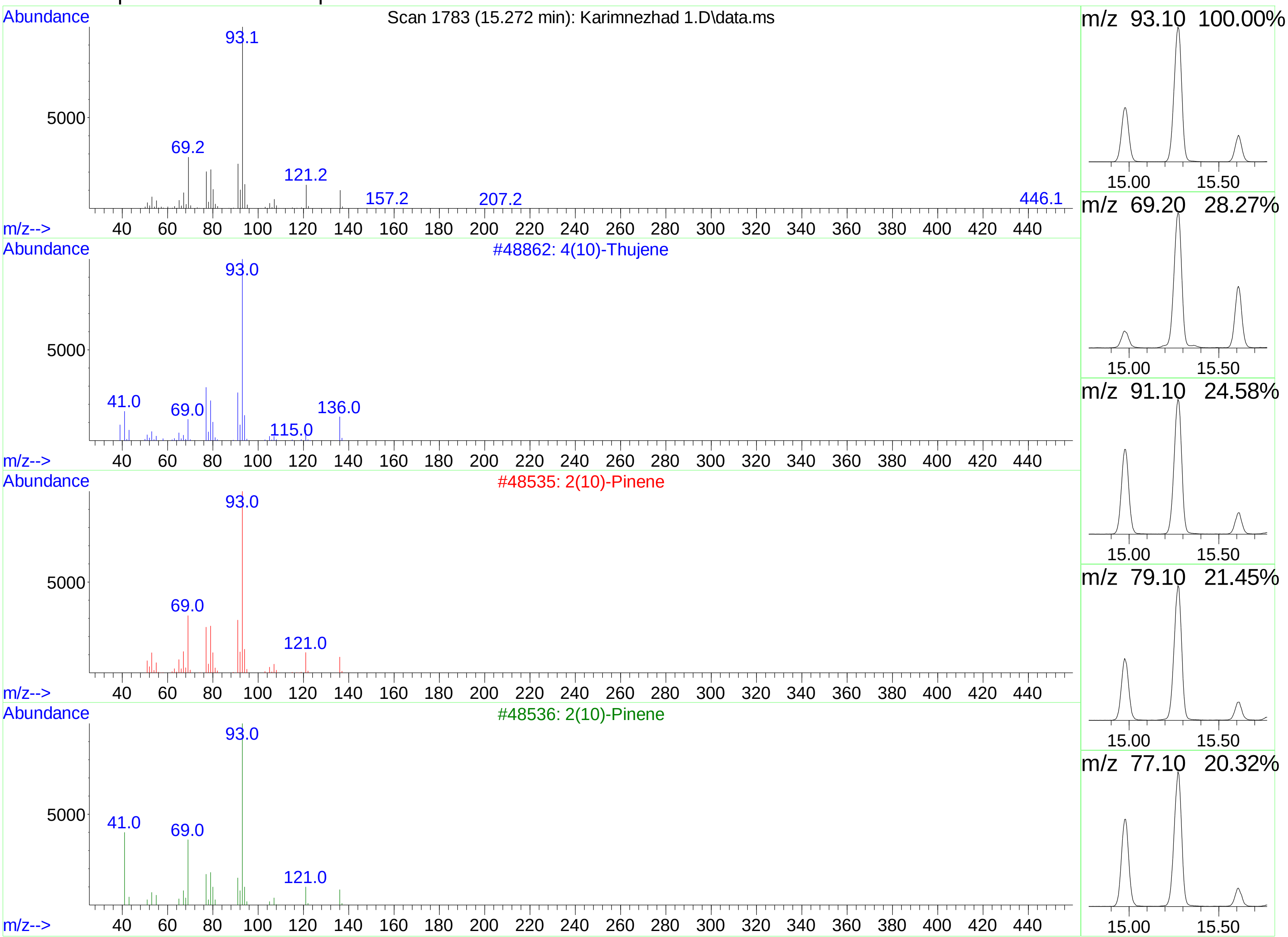

Data File: D:\msdchem\1\data\Karimnezhad 1.D
Sample : SDE

Peak Number: 5 at 15.272 min Area: 160623471 Area % 0.80

The 3 best hits from each library.	Ref\#	CAS\#	Qual
D:\Database\W10N14.L			
1 4(10)-Thujene	48862	003387-41-5	94
2 2(10)-Pinene	48535	000127-91-3	94
3 2(10)-Pinene	48536	000127-91-3	94

Unknown Spectrum based on Apex

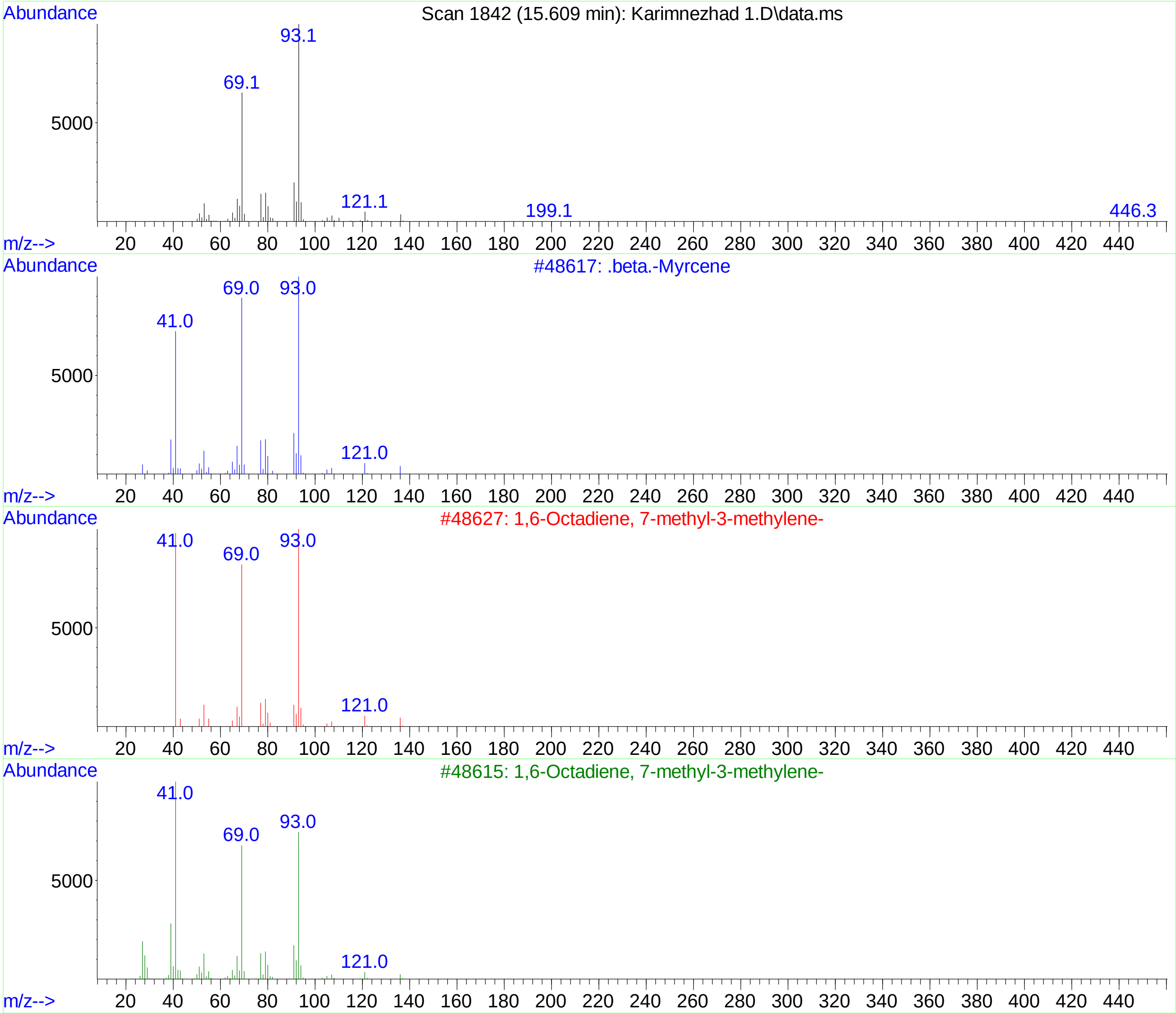

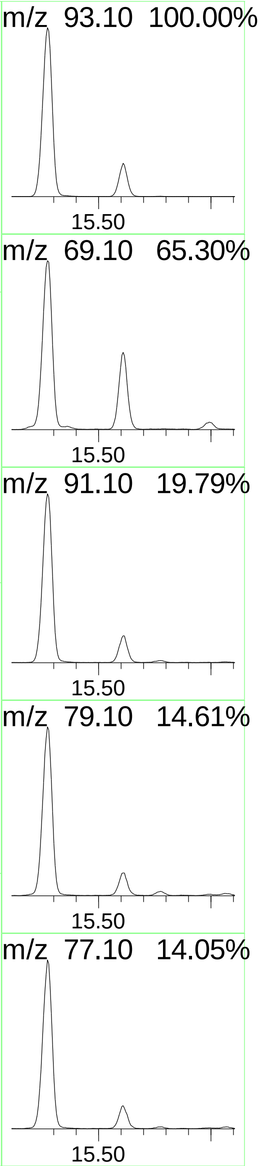

Data File: D:\msdchem\1\data\Karimnezhad 1.D
Sample : SDE

Peak Number: 6 at 15.609 min Area: 28540231 Area % 0.14

The 3 best hits from each library.	Ref#	CAS#	Qual
D:\Database\W10N14.L			
1 .beta.-Myrcene	48617	000123-35-3	96
2 1,6-Octadiene, 7-methyl-3-methyl...	48627	000123-35-3	95
3 1,6-Octadiene, 7-methyl-3-methyl...	48615	000123-35-3	94

Library Search Report - ChemStation Integrator

Unknown Spectrum based on Apex

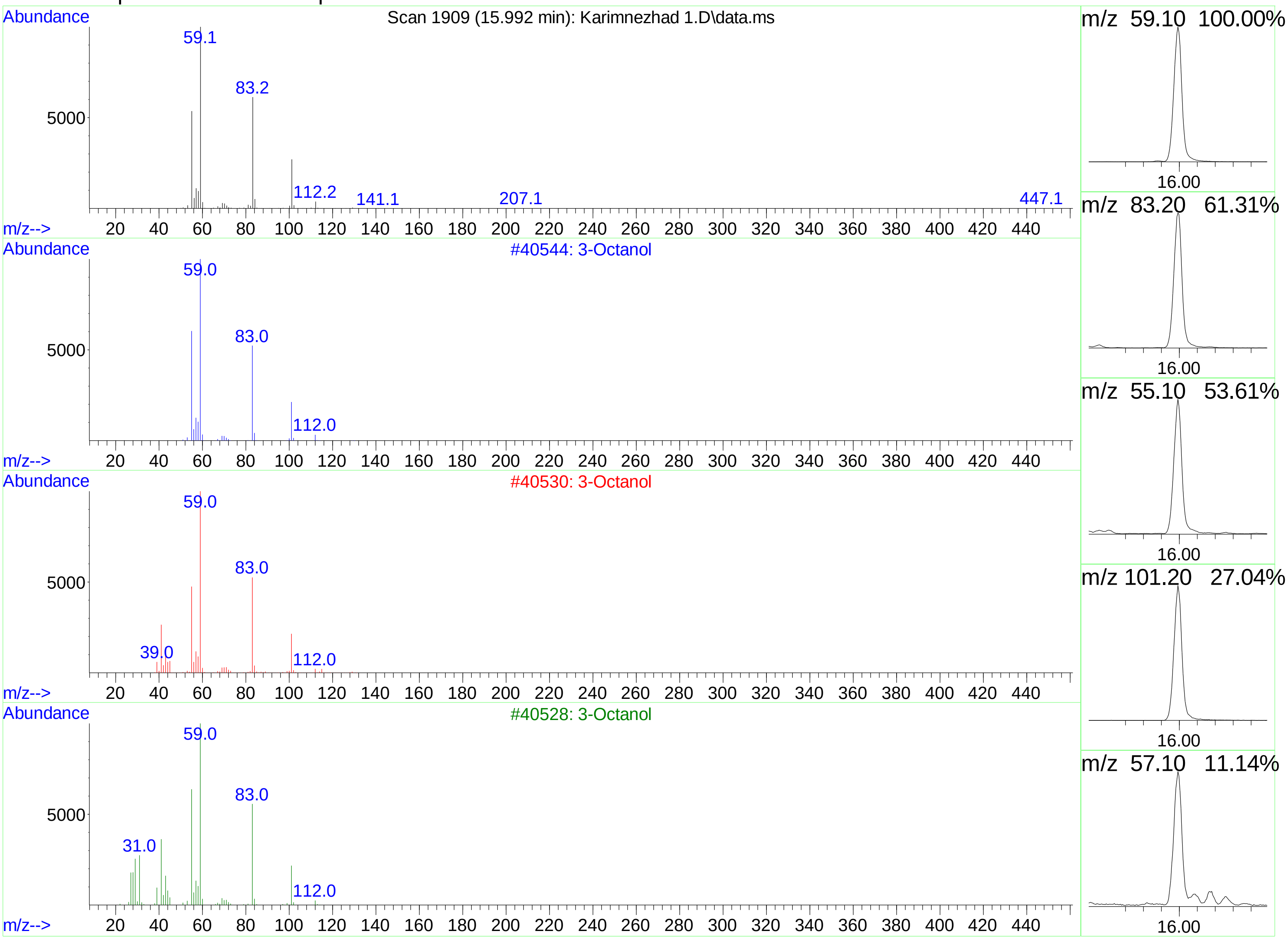

Data File: D:\msdchem\1\data\Karimnezhad 1.D
Sample : SDE

Peak Number: 7 at 15.992 min Area: 61434126 Area % 0.31

The 3 best hits from each library.	Ref\#	CAS\#	Qual
D:\Database\W10N14.L			
1 3-Octanol	40544	000589-98-0	90
2 3-Octanol	40530	000589-98-0	83
3 3-Octanol	40528	000589-98-0	83

Library Search Report - ChemStation Integrator

Unknown Spectrum based on Apex

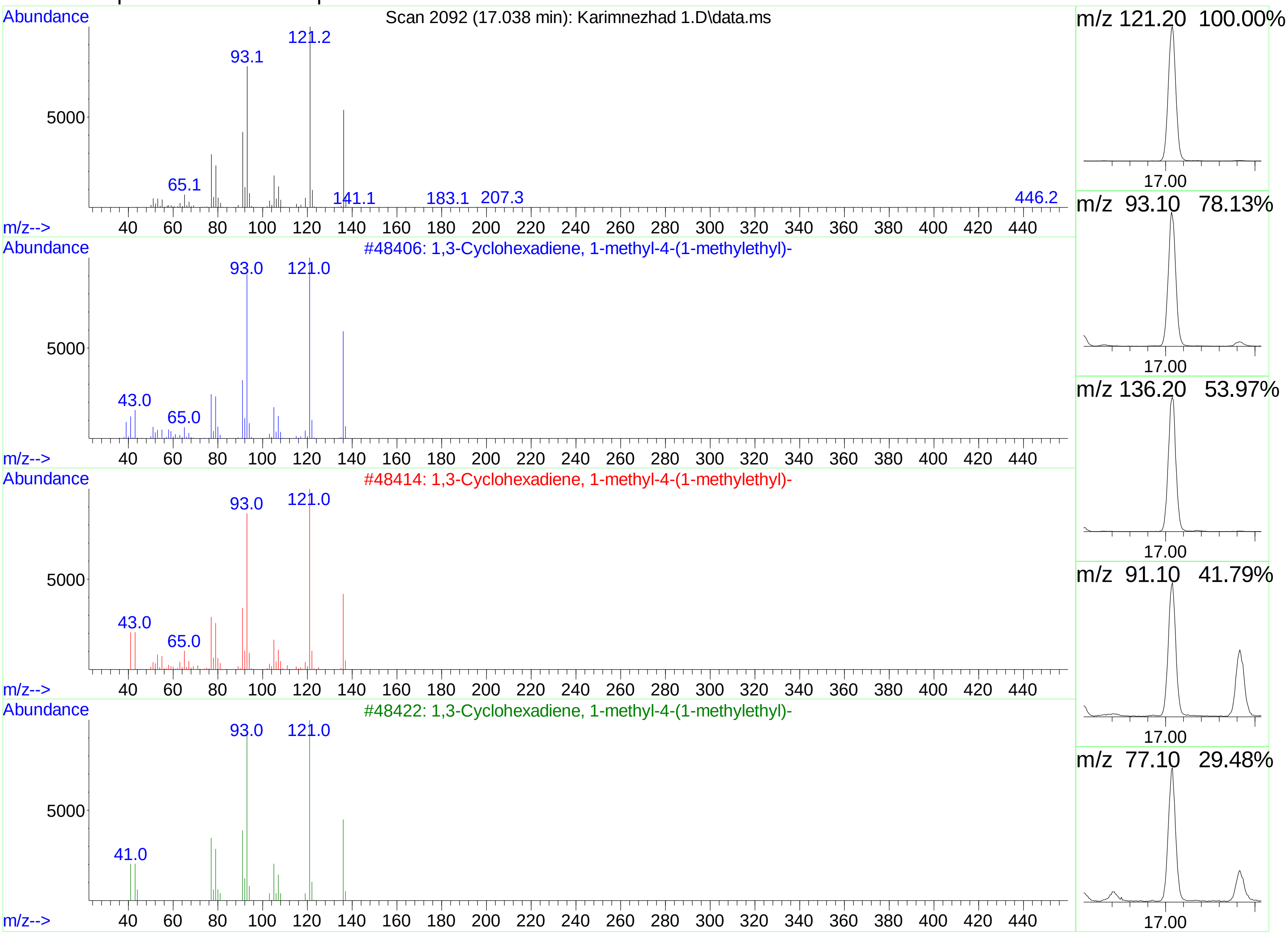

Data File: D:\msdchem\1\data\Karimnezhad 1.D
Sample : SDE

Peak Number: 8 at 17.038 min Area: 41640180 Area % 0.21

The 3 best hits from each library.	Ref#	CAS#	Qual
D:\Database\W10N14.L			
1 1,3-Cyclohexadiene, 1-methyl-4-(...	48406	000099-86-5	98
2 1,3-Cyclohexadiene, 1-methyl-4-(...	48414	000099-86-5	98
3 1,3-Cyclohexadiene, 1-methyl-4-(...	48422	000099-86-5	98

Unknown Spectrum based on Apex

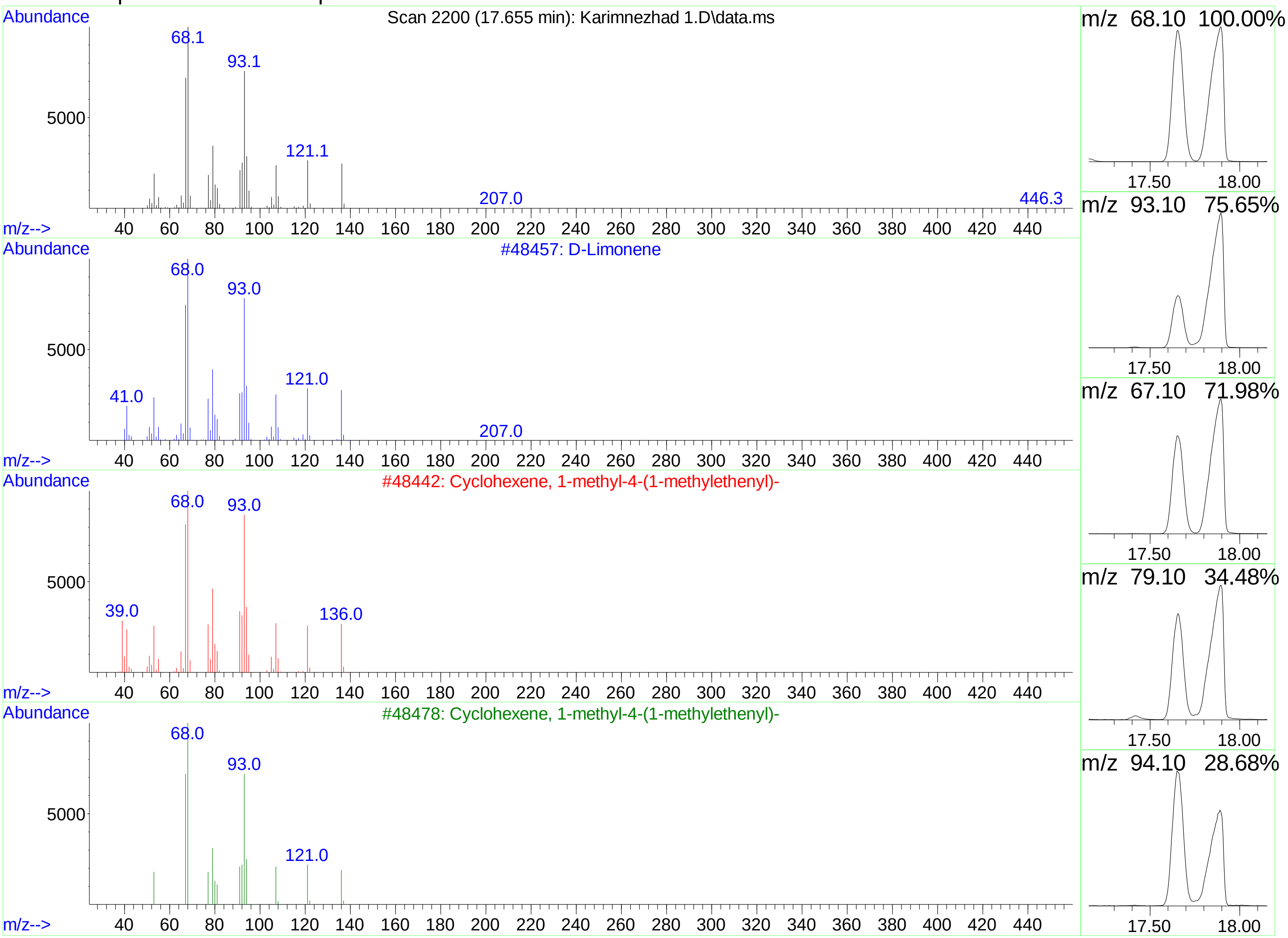

Data File: D:\msdchem\1\data\Karimnezhad 1.D
Sample : SDE

Peak Number: 9 at 17.655 min Area: 117794679 Area % 0.59

The 3 best hits from each library.	Ref\#	CAS\#	Qual
D:\Database\W10N14.L			
1 D-Limonene	48457	005989-27-5	99
2 Cyclohexene, 1-methyl-4-(1-methy...	48442	000138-86-3	98
3 Cyclohexene, 1-methyl-4-(1-methy...	48478	000138-86-3	98

Library Search Report - ChemStation Integrator

Unknown Spectrum based on Apex

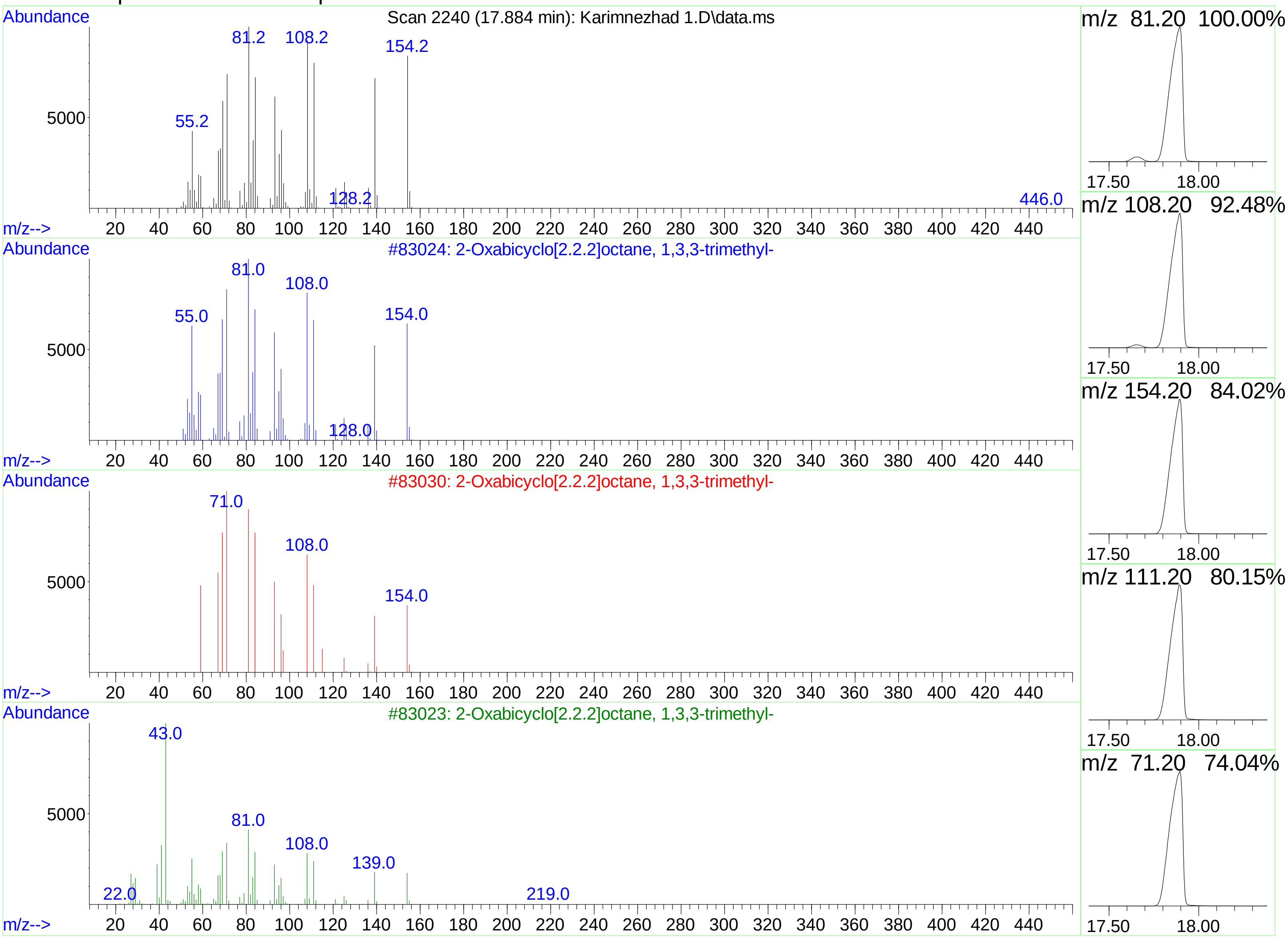

Data File: D:\msdchem\1\data\Karimnezhad 1.D
Sample : SDE

Peak Number: 10 at 17.884 min Area: 854868417 Area % 4.28

The 3 best hits from each library.	Ref#	CAS#	Qual
D:\Database\W10N14.L			
1 2-Oxabicyclo[2.2.2]octane, 1,3,3...	83024	000470-82-6	98
2 2-Oxabicyclo[2.2.2]octane, 1,3,3...	83030	000470-82-6	98
3 2-Oxabicyclo[2.2.2]octane, 1,3,3...	83023	000470-82-6	97

Library Search Report - ChemStation Integrator

Unknown Spectrum based on Apex

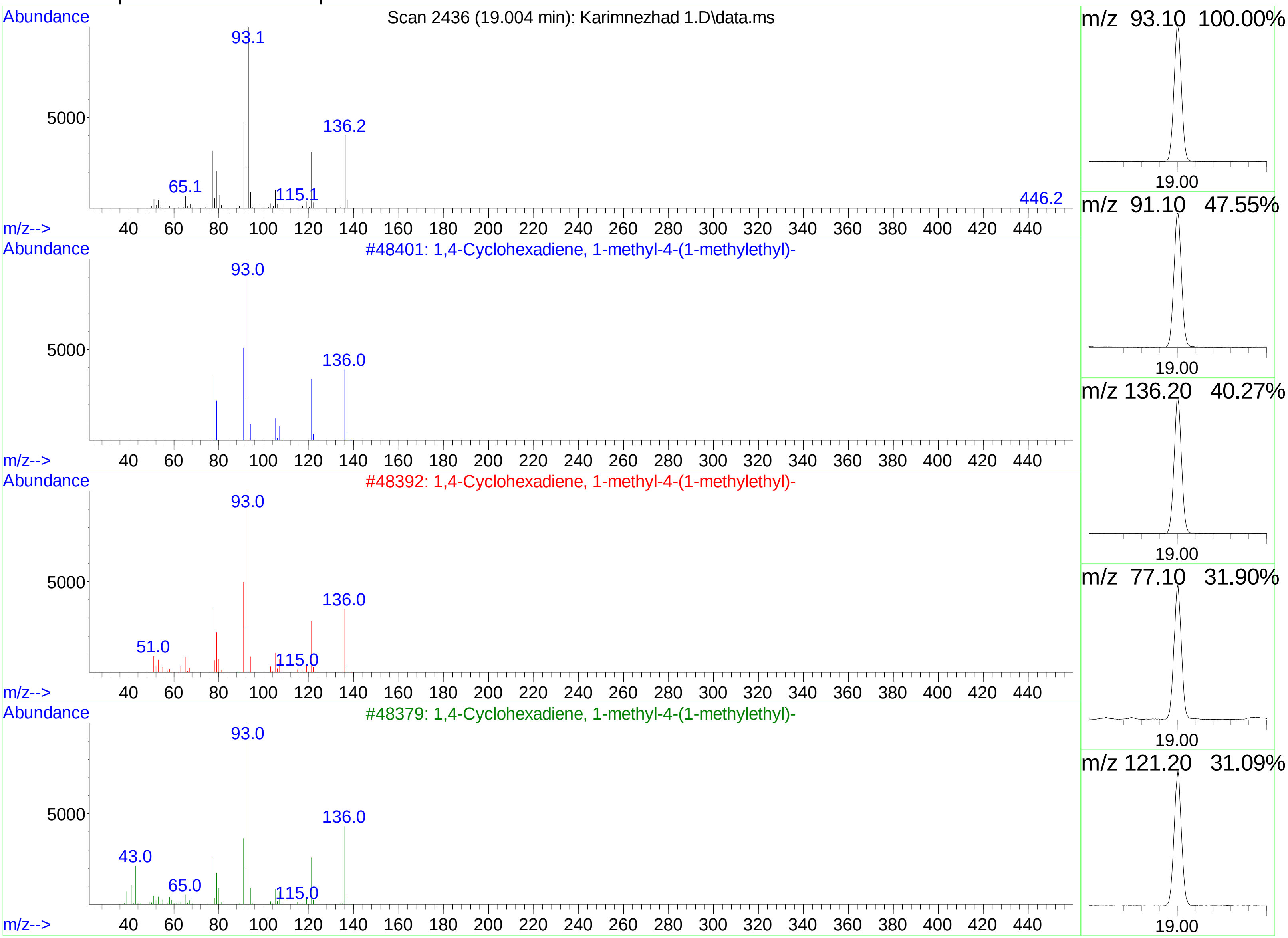

Data File: D:\msdchem\1\data\Karimnezhad 1.D
Sample : SDE

Peak Number: 11 at 19.004 min Area: 44651533 Area % 0.22

The 3 best hits from each library. Ref# CAS# Qual

D:\Database\W10N14.L
1 1,4-Cyclohexadiene, 1-methyl-4-(... 48401 000099-85-4 97
2 1,4-Cyclohexadiene, 1-methyl-4-(... 48392 000099-85-4 96
3 1,4-Cyclohexadiene, 1-methyl-4-(... 48379 000099-85-4 96

Unknown Spectrum based on Apex

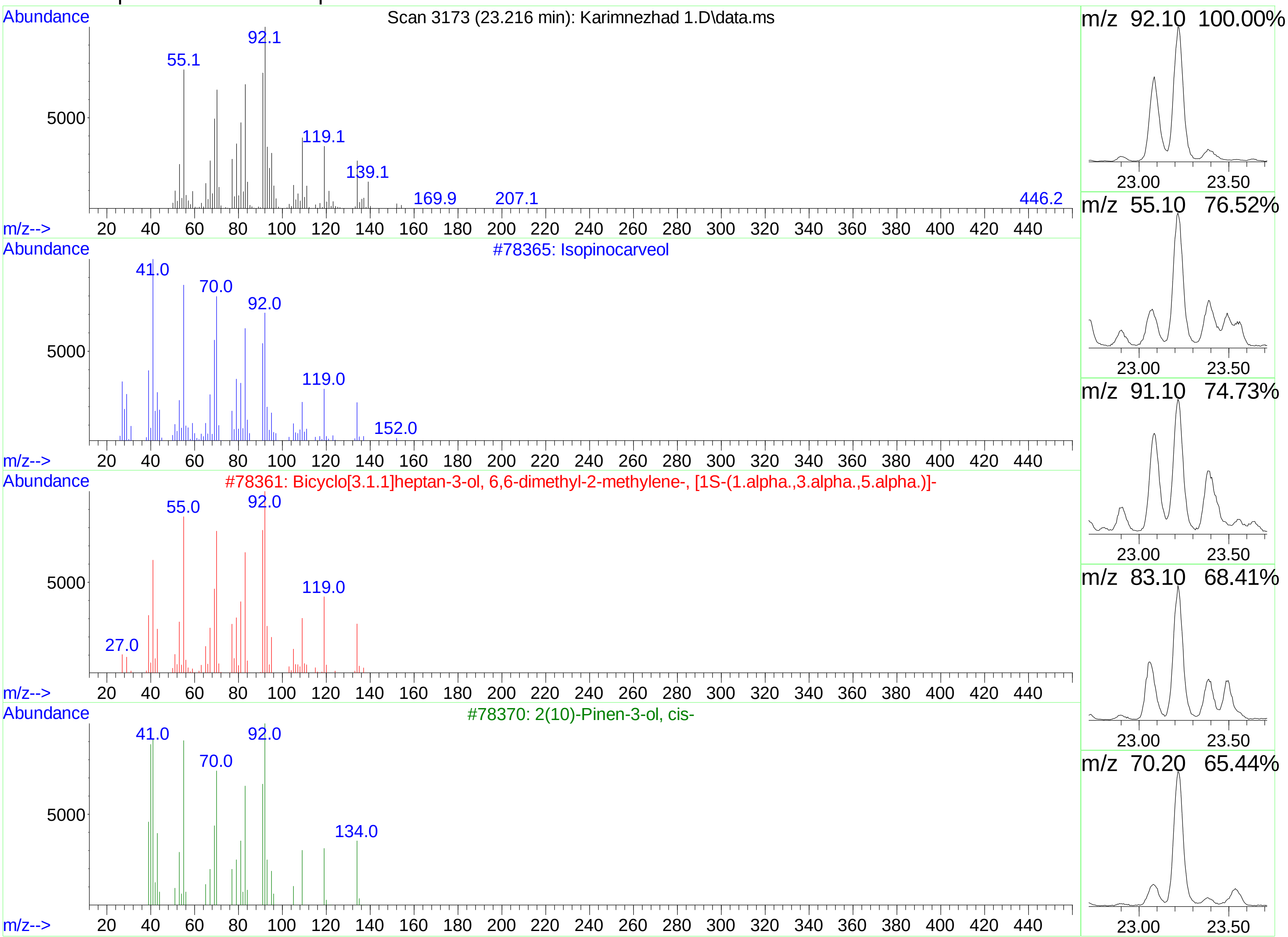

Data File: D:\msdchem\1\data\Karimnezhad 1.D
Sample : SDE

Peak Number: 12 at 23.216 min Area: 42470064 Area % 0.21

The 3 best hits from each library. Ref\# CAS\# Qual

D:\Database\W10N14.L

		Ref\#	CAS\#	Qual
1	Isopinocarveol	78365	006712-79-4	90
2	Bicyclo[3.1.1]heptan-3-ol, 6,6-d...	78361	000547-61-5	68
3	2(10)-Pinen-3-ol, cis-	78370	006712-79-4	59

Unknown Spectrum based on Apex

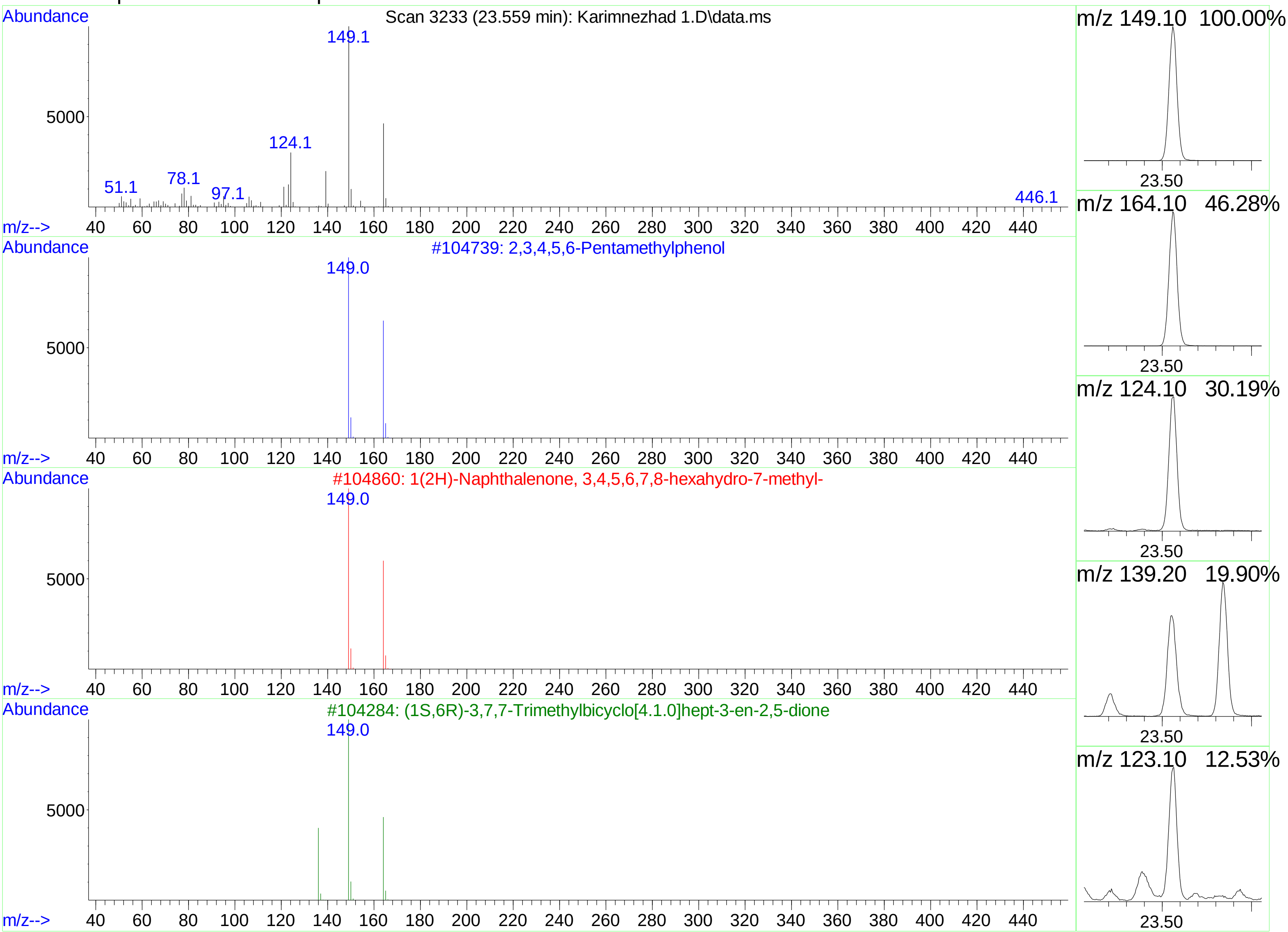

Data File: D:\msdchem\1\data\Karimnezhad 1.D
Sample : SDE

Peak Number: 13 at 23.559 min Area: 56521918 Area % 0.28

The 3 best hits from each library.	Ref#	CAS#	Qual
D:\Database\W10N14.L			
1 2,3,4,5,6-Pentamethylphenol	104739	2000104-73-9	83
2 1(2H)-Naphthalenone, 3,4,5,6,7,8...	104860	059177-21-8	83
3 (1S,6R)-3,7,7-Trimethylbicyclo[4...	104284	2000104-28-4	80

Unknown Spectrum based on Apex

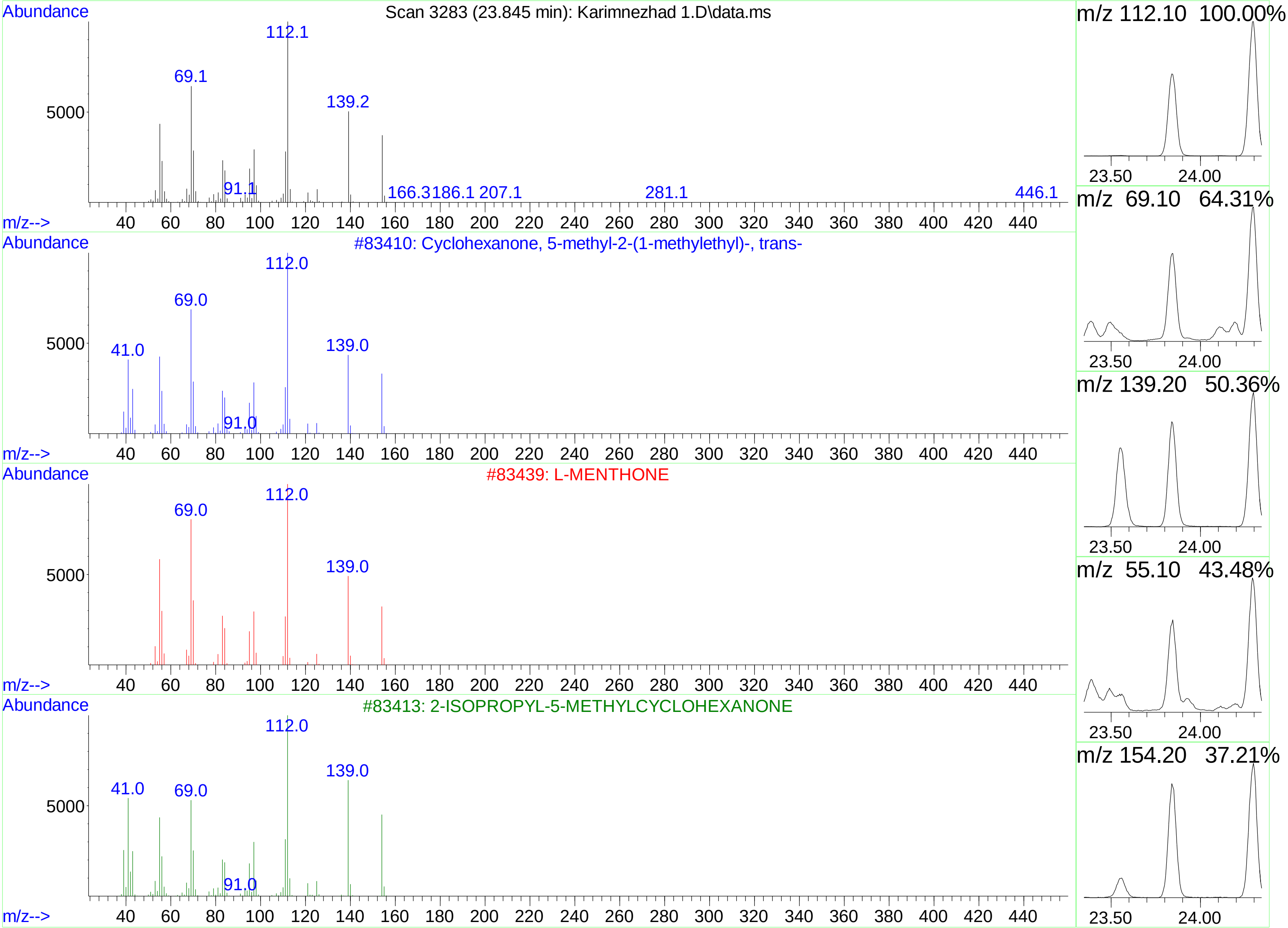

Data File: D:\msdchem\1\data\Karimnezhad 1.D
Sample : SDE

Peak Number: 14 at 23.845 min Area: 42597508 Area % 0.21

The 3 best hits from each library.	Ref\#	CAS\#	Qual
D:\Database\W10N14.L			
1 Cyclohexanone, 5-methyl-2-(1-met...	83410	000089-80-5	98
2 L-MENTHONE	83439	010458-14-7	98
3 2-ISOPROPYL-5-METHYLCYCLOHEXANONE	83413	000089-80-5	98

Unknown Spectrum based on Apex

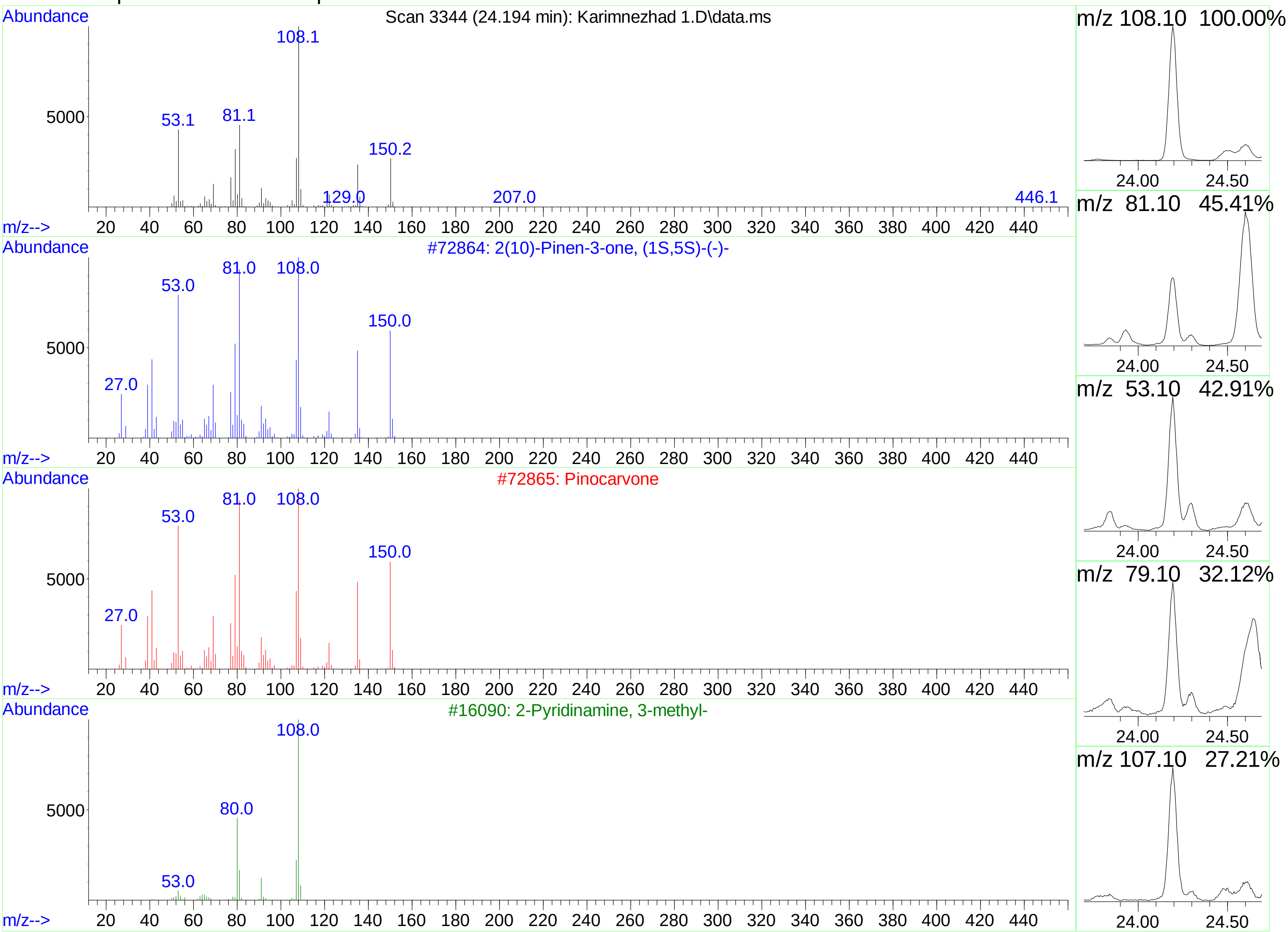

Data File: D:\msdchem\1\data\Karimnezhad 1.D
Sample : SDE

Peak Number: 15 at 24.194 min Area: 30473062 Area % 0.15

The 3 best hits from each library.	Ref\#	CAS\#	Qual
D:\Database\W10N14.L			
1 2(10)-Pinen-3-one, (1S,5S)-(-)-	72864	019890-00-7	91
2 Pinocarvone	72865	030460-92-5	91
3 2-Pyridinamine, 3-methyl-	16090	001603-40-3	72

Unknown Spectrum based on Apex

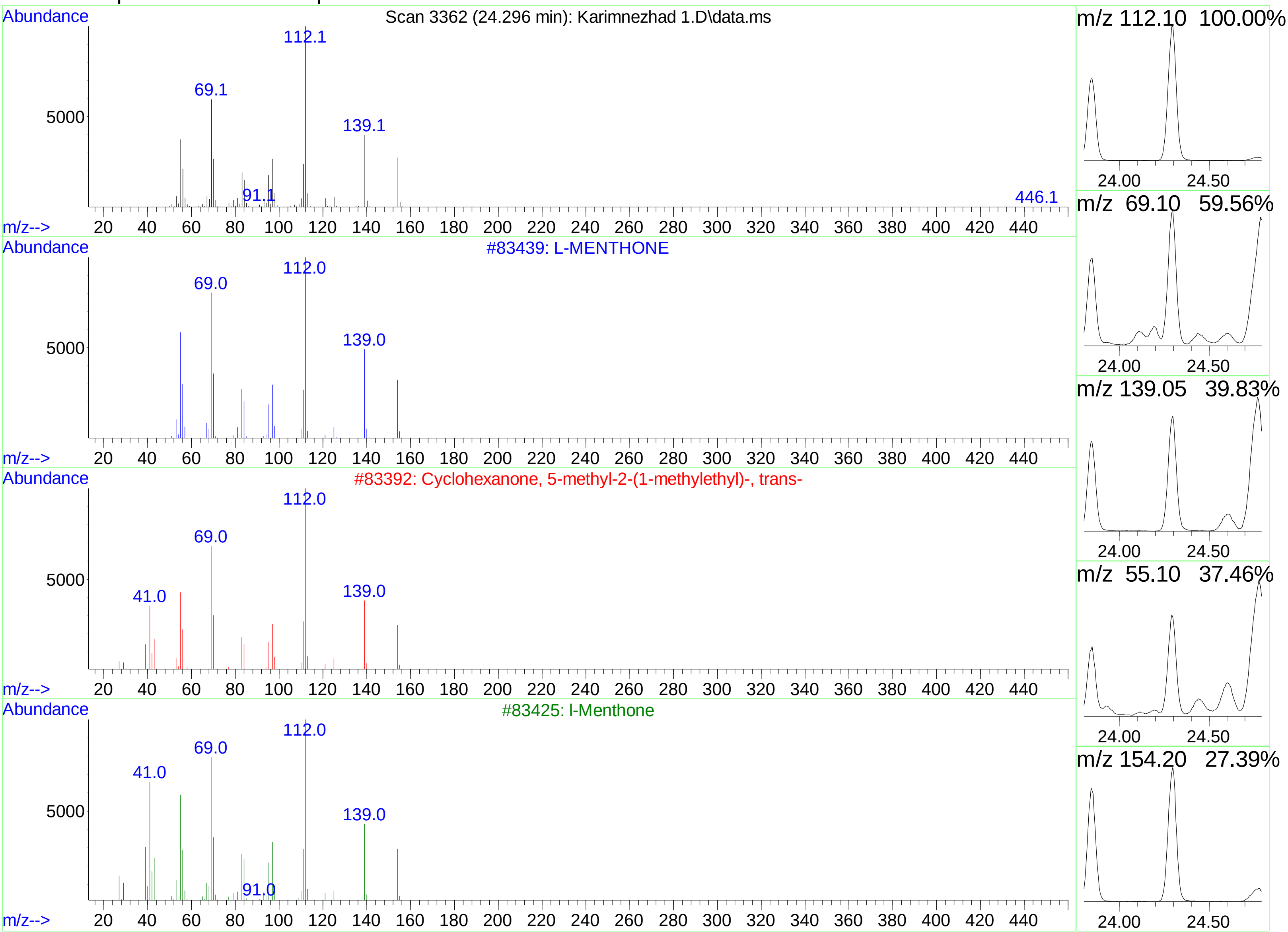

Data File: D:\msdchem\1\data\Karimnezhad 1.D
Sample : SDE

Peak Number: 16 at 24.296 min Area: 49932678 Area % 0.25

The 3 best hits from each library.	Ref#	CAS#	Qual
D:\Database\W10N14.L			
1 L-MENTHONE	83439	010458-14-7	98
2 Cyclohexanone, 5-methyl-2-(1-met...	83392	000089-80-5	98
3 l-Menthone	83425	014073-97-3	98

Library Search Report - ChemStation Integrator

Unknown Spectrum based on Apex

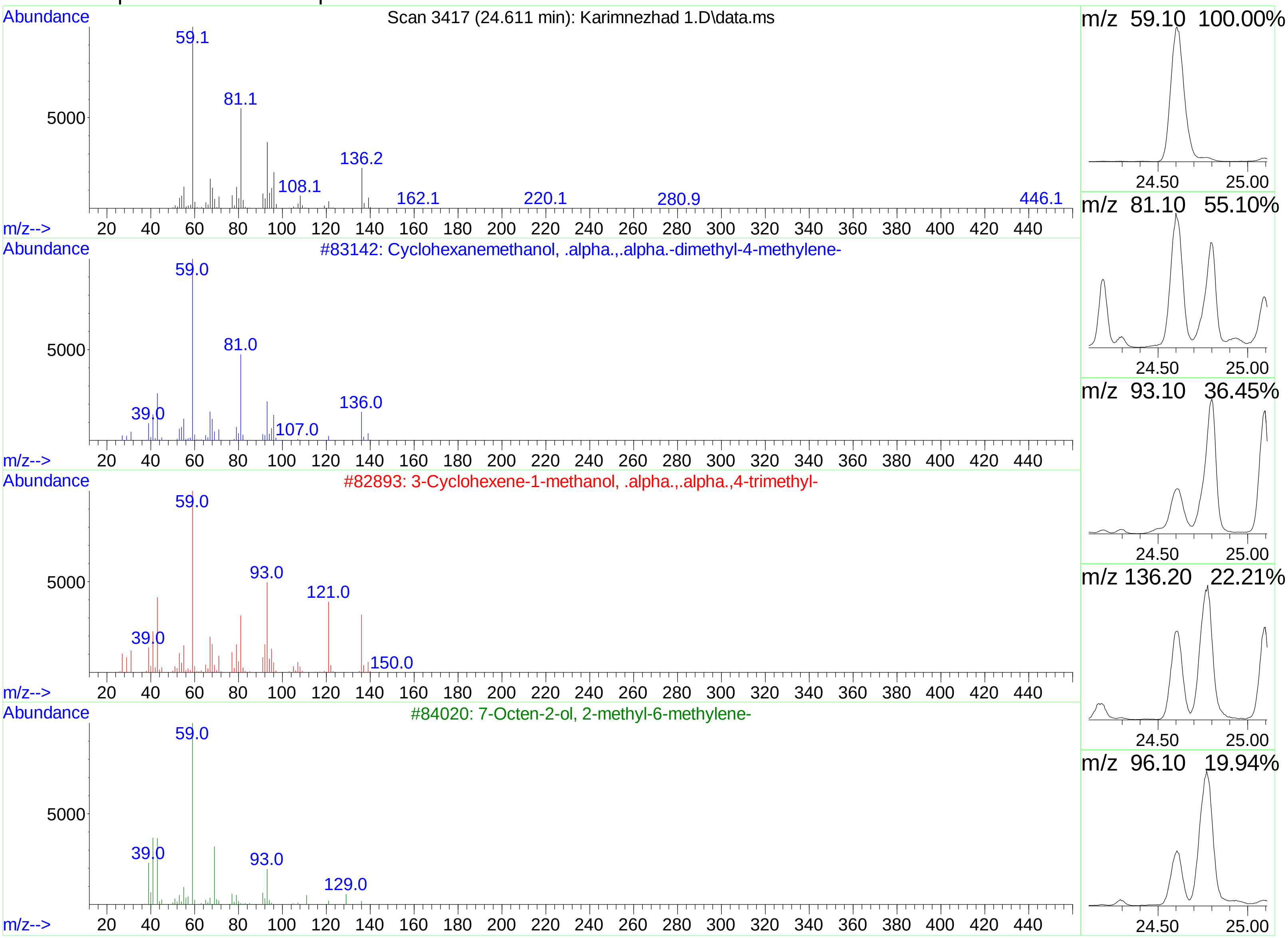

Data File: D:\msdchem\1\data\Karimnezhad 1.D
Sample : SDE

Peak Number: 17 at 24.611 min Area: 71502162 Area % 0.36

The 3 best hits from each library.	Ref#	CAS#	Qual
D:\Database\W10N14.L			
1 Cyclohexanemethanol, .alpha.,.al...	83142	007299-42-5	90
2 3-Cyclohexene-1-methanol, .alpha...	82893	010482-56-1	64
3 7-Octen-2-ol, 2-methyl-6-methylene-	84020	000543-39-5	59

Unknown Spectrum based on Apex

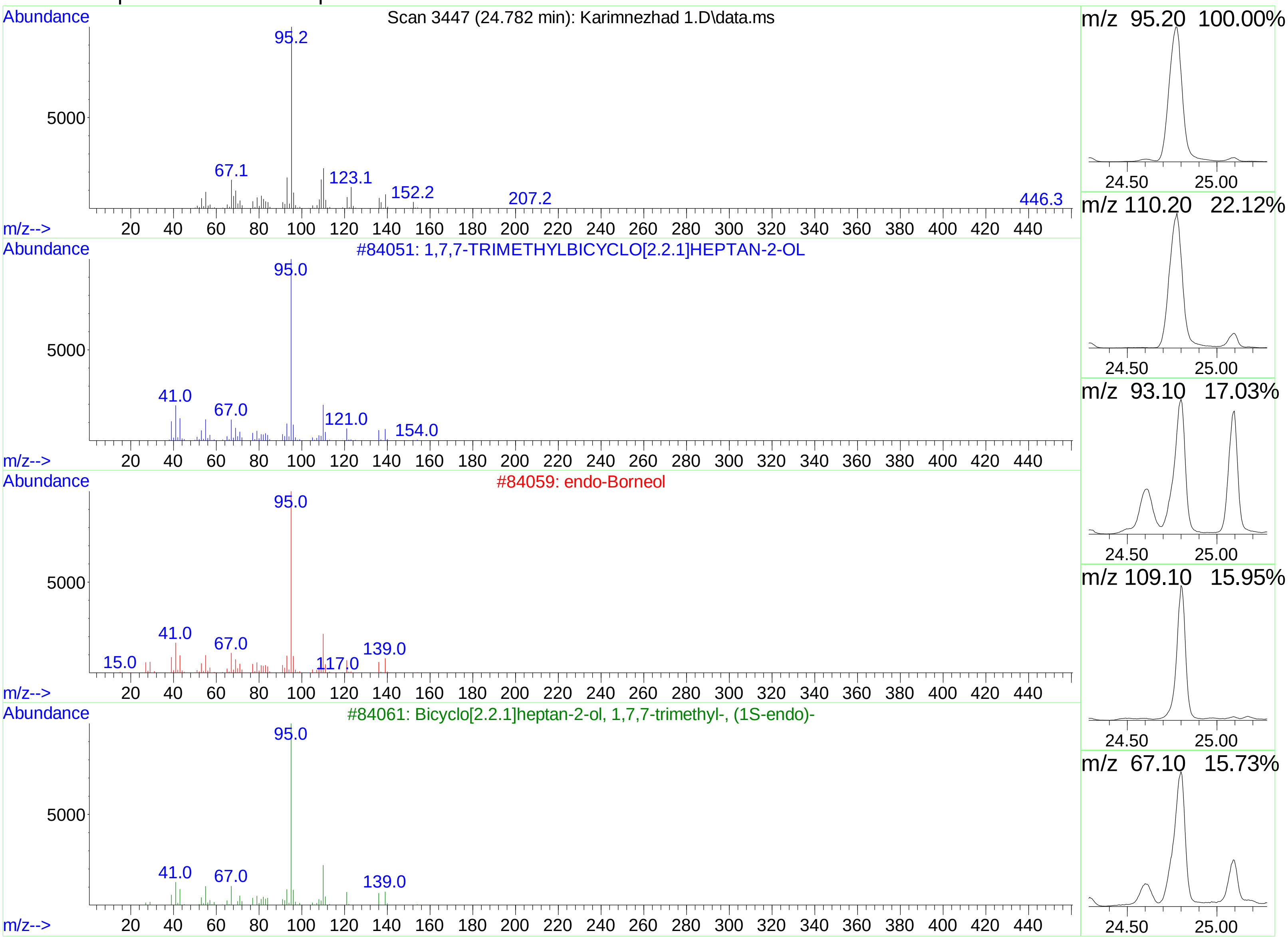

Data File: D:\msdchem\1\data\Karimnezhad 1.D
Sample : SDE

Peak Number: 18 at 24.782 min Area: 302062473 Area % 1.51

The 3 best hits from each library.	Ref#	CAS#	Qual
D:\Database\W10N14.L			
1 1,7,7-TRIMETHYLBICYCLO[2.2.1]HEP...	84051	000464-45-9	94
2 endo-Borneol	84059	000507-70-0	93
3 Bicyclo[2.2.1]heptan-2-ol, 1,7,7...	84061	000464-45-9	93

Unknown Spectrum based on Apex

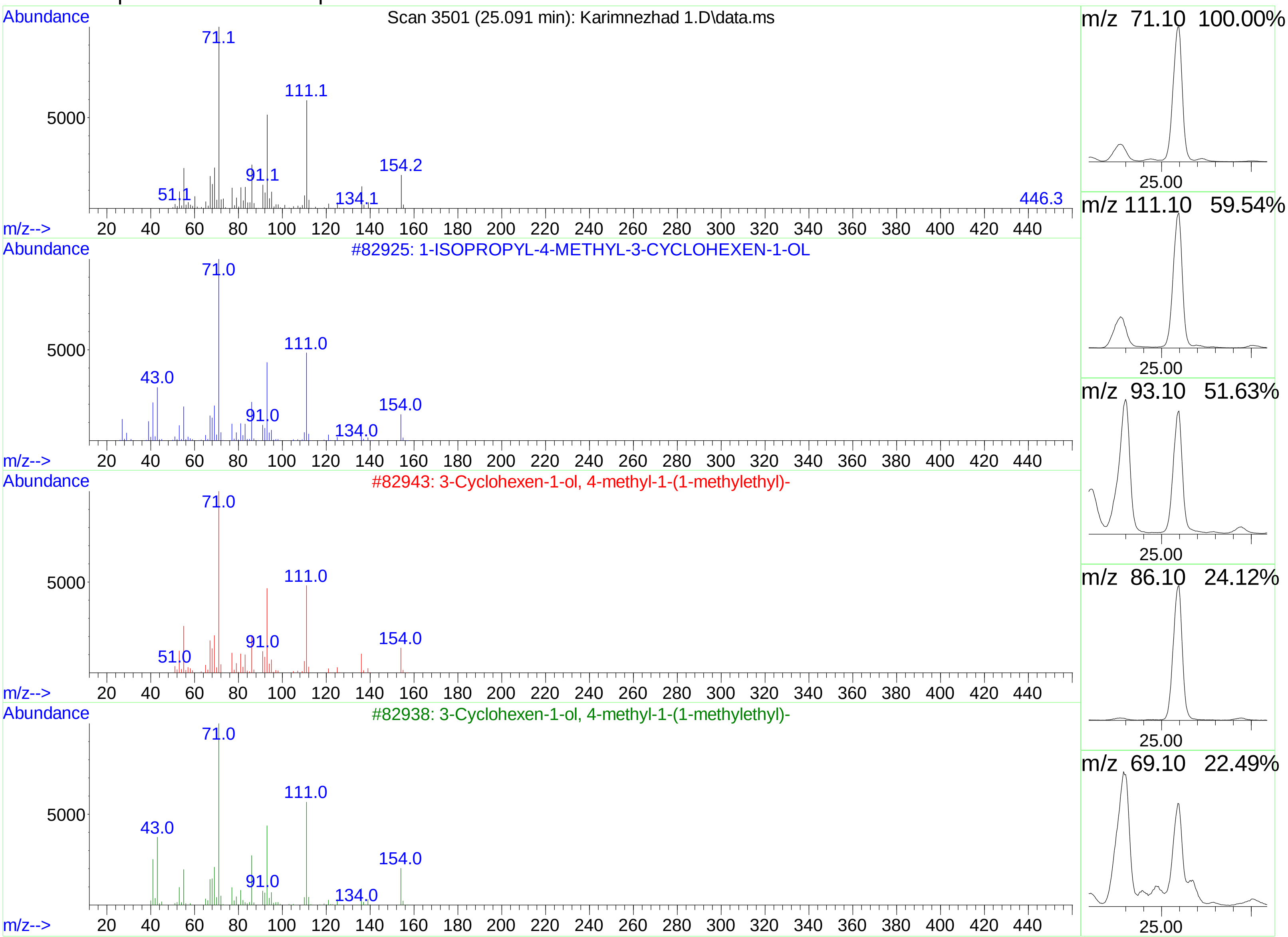

Data File: D:\msdchem\1\data\Karimnezhad 1.D
Sample : SDE

Peak Number: 19 at 25.091 min Area: 135317412 Area % 0.68

The 3 best hits from each library.	Ref\#	CAS\#	Qual
D:\Database\W10N14.L			
1 1-ISOPROPYL-4-METHYL-3-CYCLOHEXE...	82925	000562-74-3	98
2 3-Cyclohexen-1-ol, 4-methyl-1-(1...	82943	000562-74-3	98
3 3-Cyclohexen-1-ol, 4-methyl-1-(1...	82938	000562-74-3	97

Library Search Report - ChemStation Integrator

Unknown Spectrum based on Apex

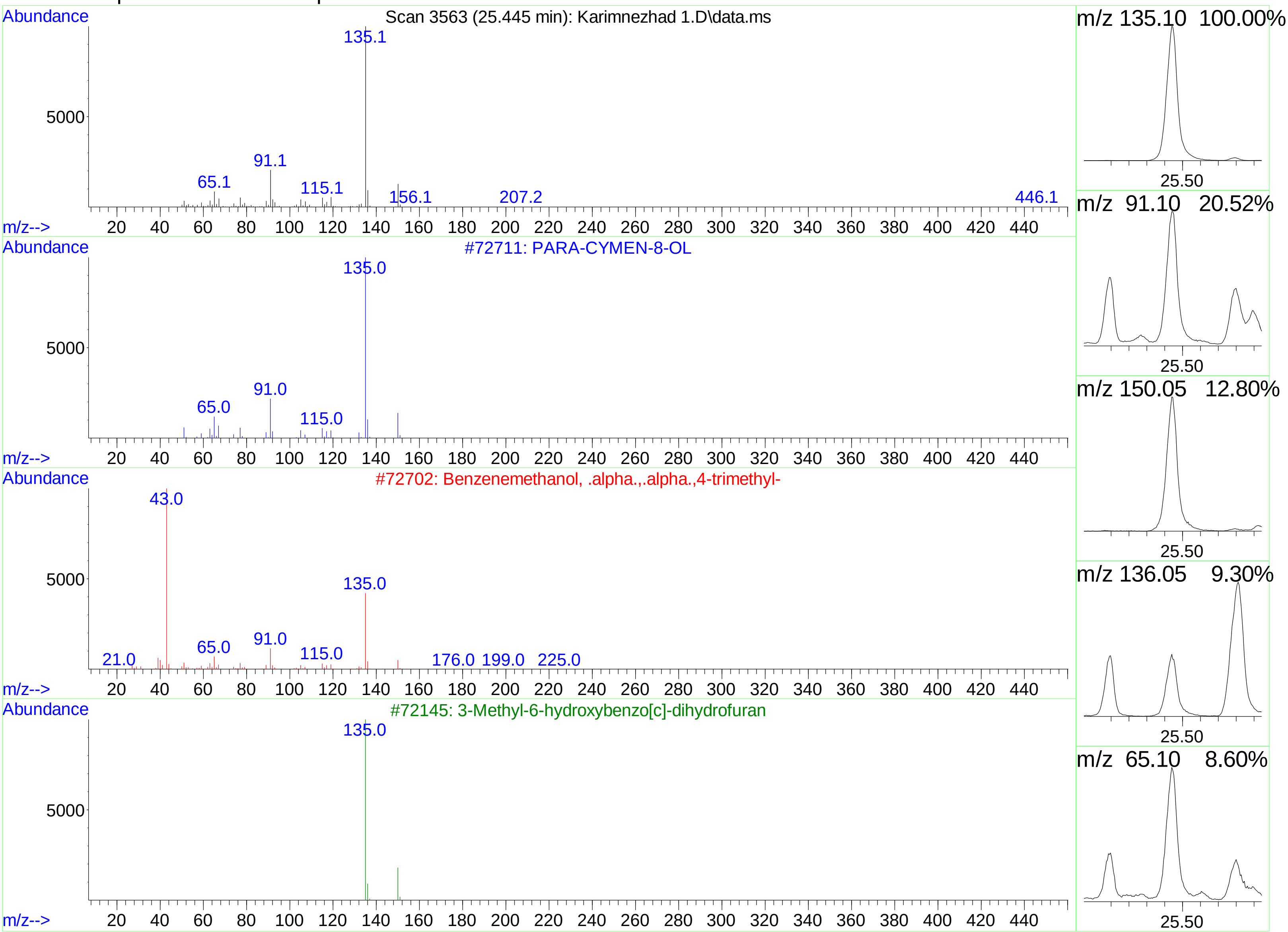

Data File: D:\msdchem\1\data\Karimnezhad 1.D
Sample : SDE

Peak Number: 20 at 25.445 min Area: 80844071 Area % 0.40

The 3 best hits from each library.	Ref\#	CAS\#	Qual
D:\Database\W10N14.L			
1 PARA-CYMEN-8-OL	72711	001197-01-9	90
2 Benzenemethanol, .alpha.,.alpha....	72702	001197-01-9	87
3 3-Methyl-6-hydroxybenzo[c]-dihyd...	72145	2000072-14-5	83

Unknown Spectrum based on Apex

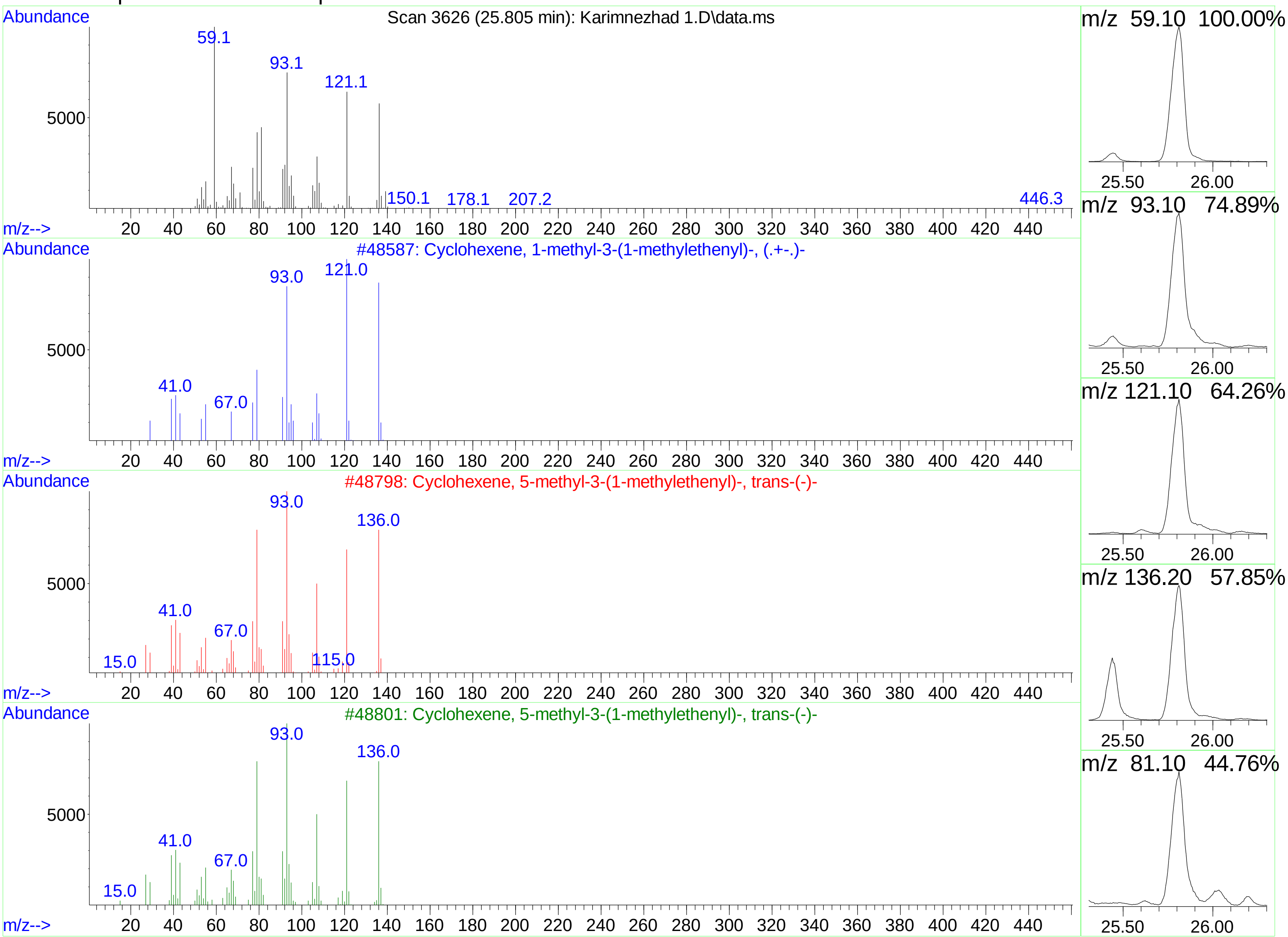

Data File: D:\msdchem\1\data\Karimnezhad 1.D
Sample : SDE

Peak Number: 21 at 25.805 min Area: 104584200 Area % 0.52

The 3 best hits from each library.	Ref\#	CAS\#	Qual
D:\Database\W10N14.L			
1 Cyclohexene, 1-methyl-3-(1-methy...	48587	000499-03-6	89
2 Cyclohexene, 5-methyl-3-(1-methy...	48798	056816-08-1	60
3 Cyclohexene, 5-methyl-3-(1-methy...	48801	056816-08-1	60

Unknown Spectrum based on Apex

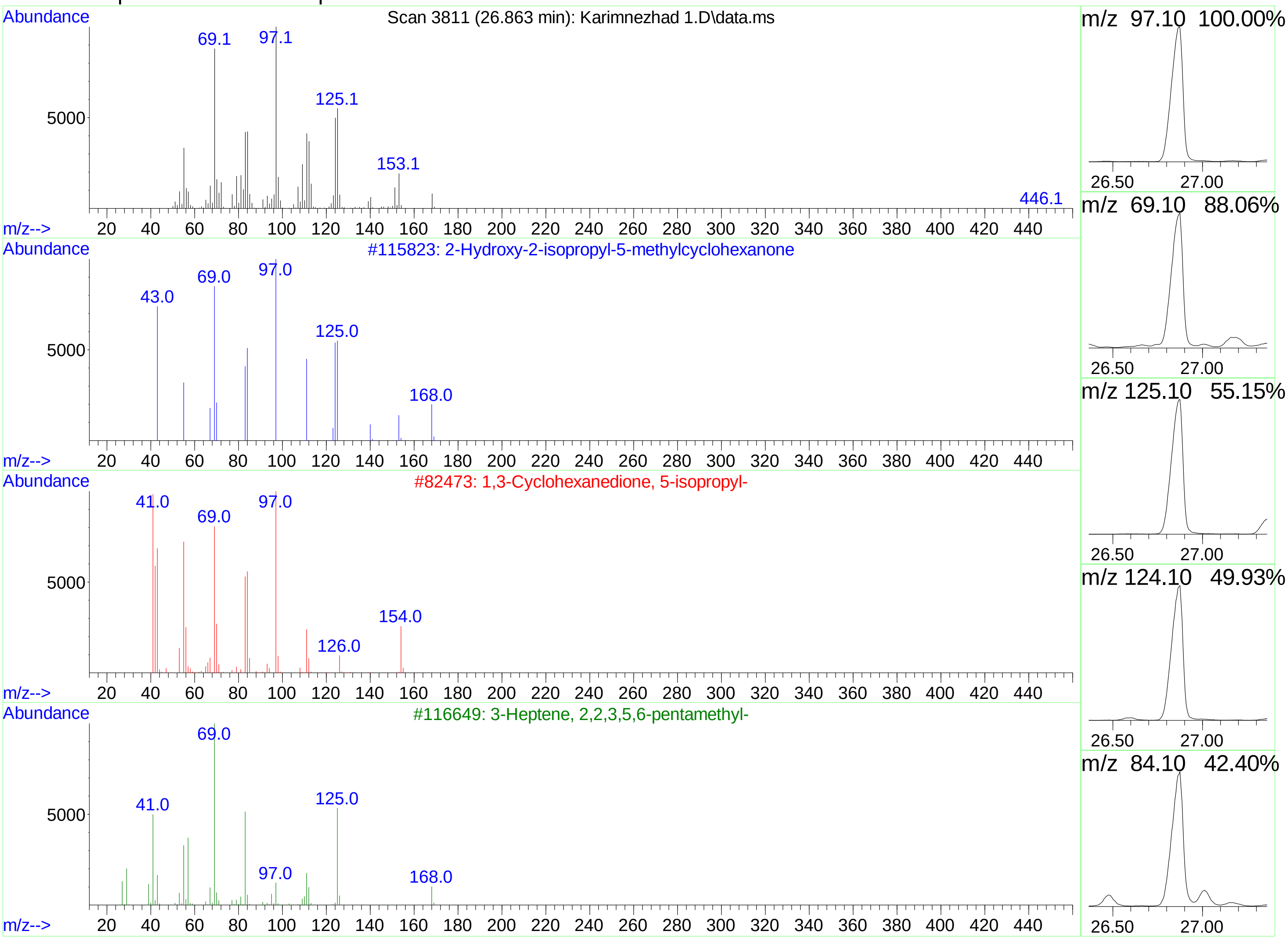

Data File: D:\msdchem\1\data\Karimnezhad 1.D
Sample : SDE

Peak Number: 22 at 26.863 min Area: 311017647 Area % 1.56

The 3 best hits from each library.	Ref\#	CAS\#	Qual
D:\Database\W10N14.L			
1 2-Hydroxy-2-isopropyl-5-methylcy...	115823	000000-00-0	80
2 1,3-Cyclohexanedione, 5-isopropyl-	82473	018456-87-6	41
3 3-Heptene, 2,2,3,5,6-pentamethyl-	116649	116164-06-8	30

Unknown Spectrum based on Apex

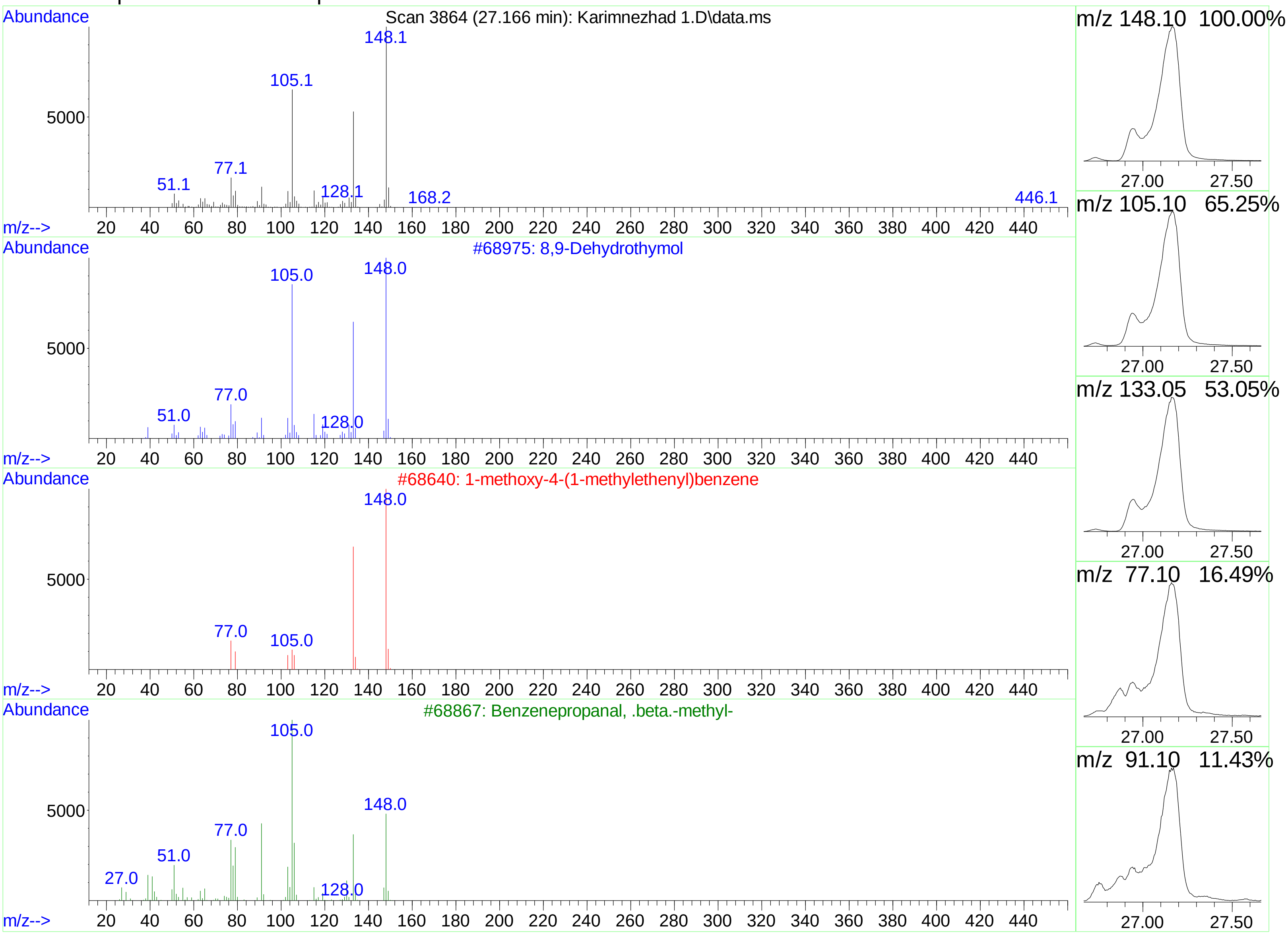

Data File: D:\msdchem\1\data\Karimnezhad 1.D
Sample : SDE

Peak Number: 23 at 27.166 min Area: 457182272 Area % 2.29

The 3 best hits from each library.	Ref\#	CAS\#	Qual
D:\Database\W10N14.L			
1 8,9-Dehydrothymol	68975	018612-99-2	95
2 1-methoxy-4-(1-methylethenyl)ben...	68640	2000068-64-0	81
3 Benzenepropanal, .beta.-methyl-	68867	016251-77-7	80

Unknown Spectrum based on Apex

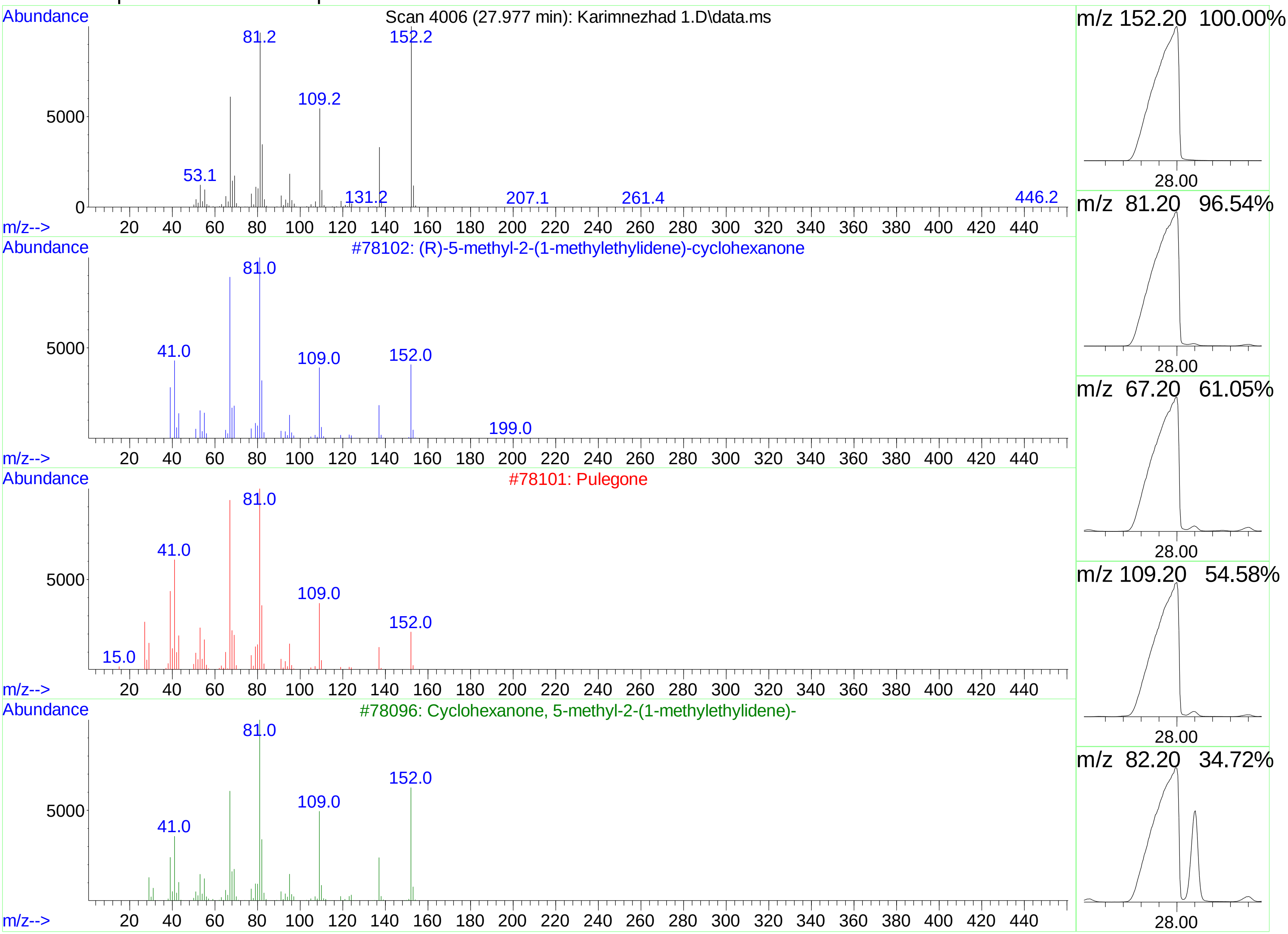

Data File: D:\msdchem\1\data\Karimnezhad 1.D
Sample : SDE

Peak Number: 24 at 27.977 min Area: 1865718373 Area % 9.34

The 3 best hits from each library.	Ref\#	CAS\#	Qual
D:\Database\W10N14.L			
1 (R)-5-methyl-2-(1-methylethylide...	78102	000089-82-7	97
2 Pulegone	78101	000089-82-7	97
3 Cyclohexanone, 5-methyl-2-(1-met...	78096	015932-80-6	96

Unknown Spectrum based on Apex

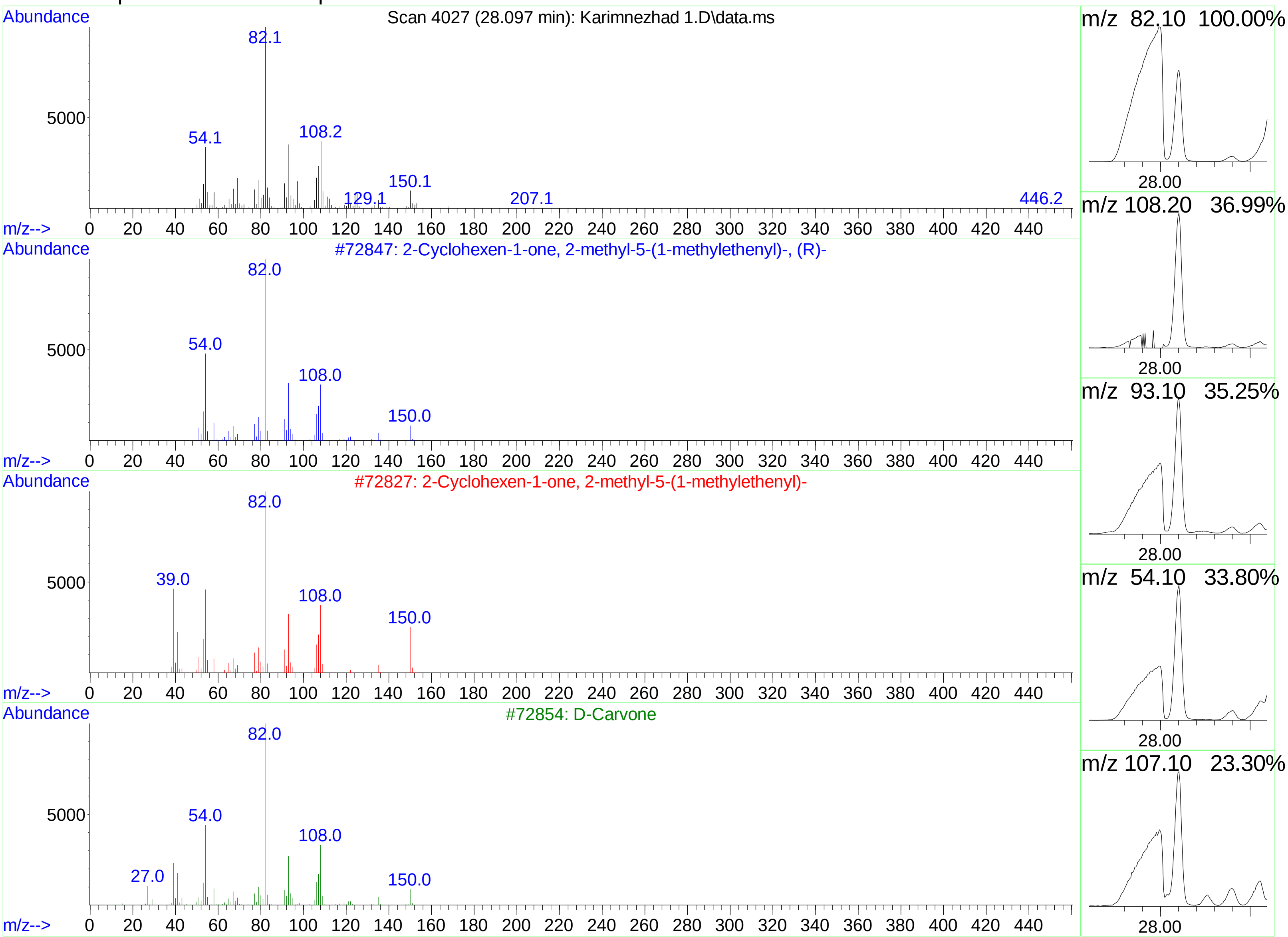

Data File: D:\msdchem\1\data\Karimnezhad 1.D
Sample : SDE

Peak Number: 25 at 28.097 min Area: 120388632 Area % 0.60

The 3 best hits from each library.	Ref\#	CAS\#	Qual
D:\Database\W10N14.L			
1 2-Cyclohexen-1-one, 2-methyl-5-(...	72847	006485-40-1	96
2 2-Cyclohexen-1-one, 2-methyl-5-(...	72827	000099-49-0	95
3 D-Carvone	72854	002244-16-8	95

Unknown Spectrum based on Apex

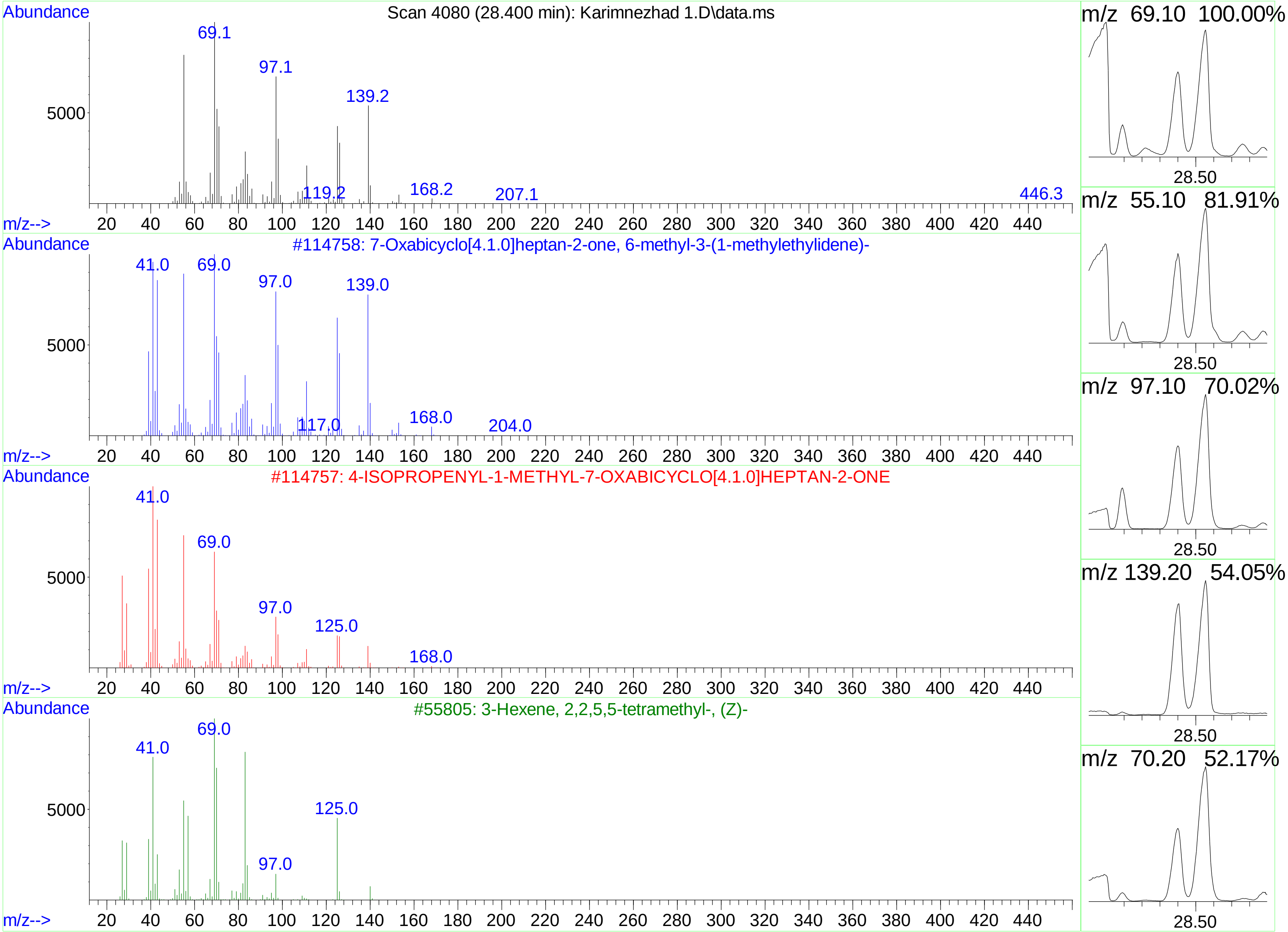

Data File: D:\msdchem\1\data\Karimnezhad 1.D
Sample : SDE

Peak Number: 26 at 28.400 min Area: 118182296 Area % 0.59

The 3 best hits from each library. Ref\# CAS\# Qual

D:\Database\W10N14.L

	Name	Ref\#	CAS\#	Qual
1	7-Oxabicyclo[4.1.0]heptan-2-one,...	114758	035178-55-3	97
2	4-ISOPROPENYL-1-METHYL-7-OXABICY...	114757	035178-55-3	52
3	3-Hexene, 2,2,5,5-tetramethyl-, ...	55805	000692-47-7	46

Unknown Spectrum based on Apex

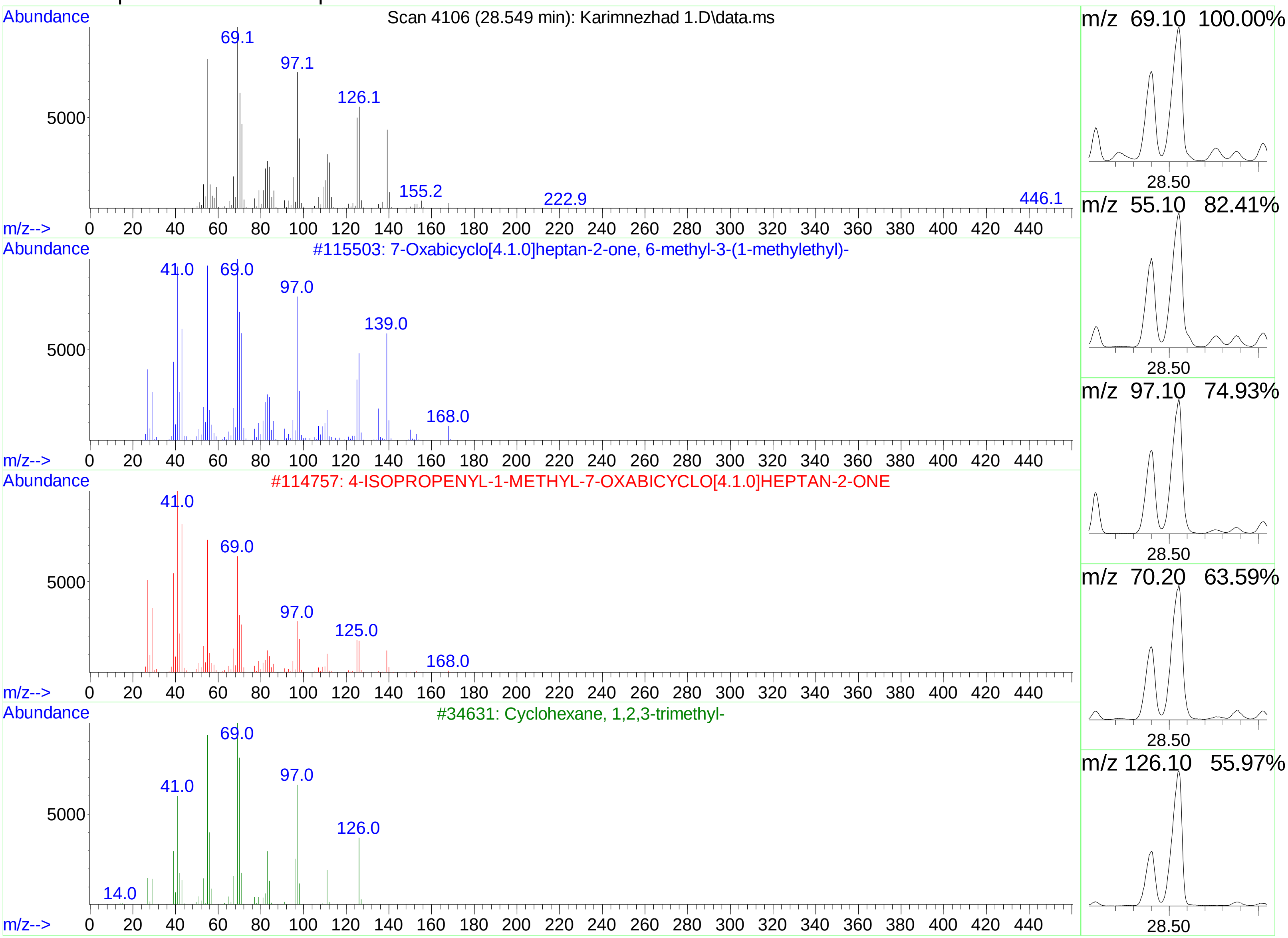

Data File: D:\msdchem\1\data\Karimnezhad 1.D
Sample : SDE

Peak Number: 27 at 28.549 min Area: 254294530 Area % 1.27

The 3 best hits from each library.	Ref#	CAS#	Qual
D:\Database\W10N14.L			
1 7-Oxabicyclo[4.1.0]heptan-2-one,...	115503	005286-38-4	91
2 4-ISOPROPENYL-1-METHYL-7-OXABICY...	114757	035178-55-3	70
3 Cyclohexane, 1,2,3-trimethyl-	34631	001678-97-3	60

Library Search Report - ChemStation Integrator

Unknown Spectrum based on Apex

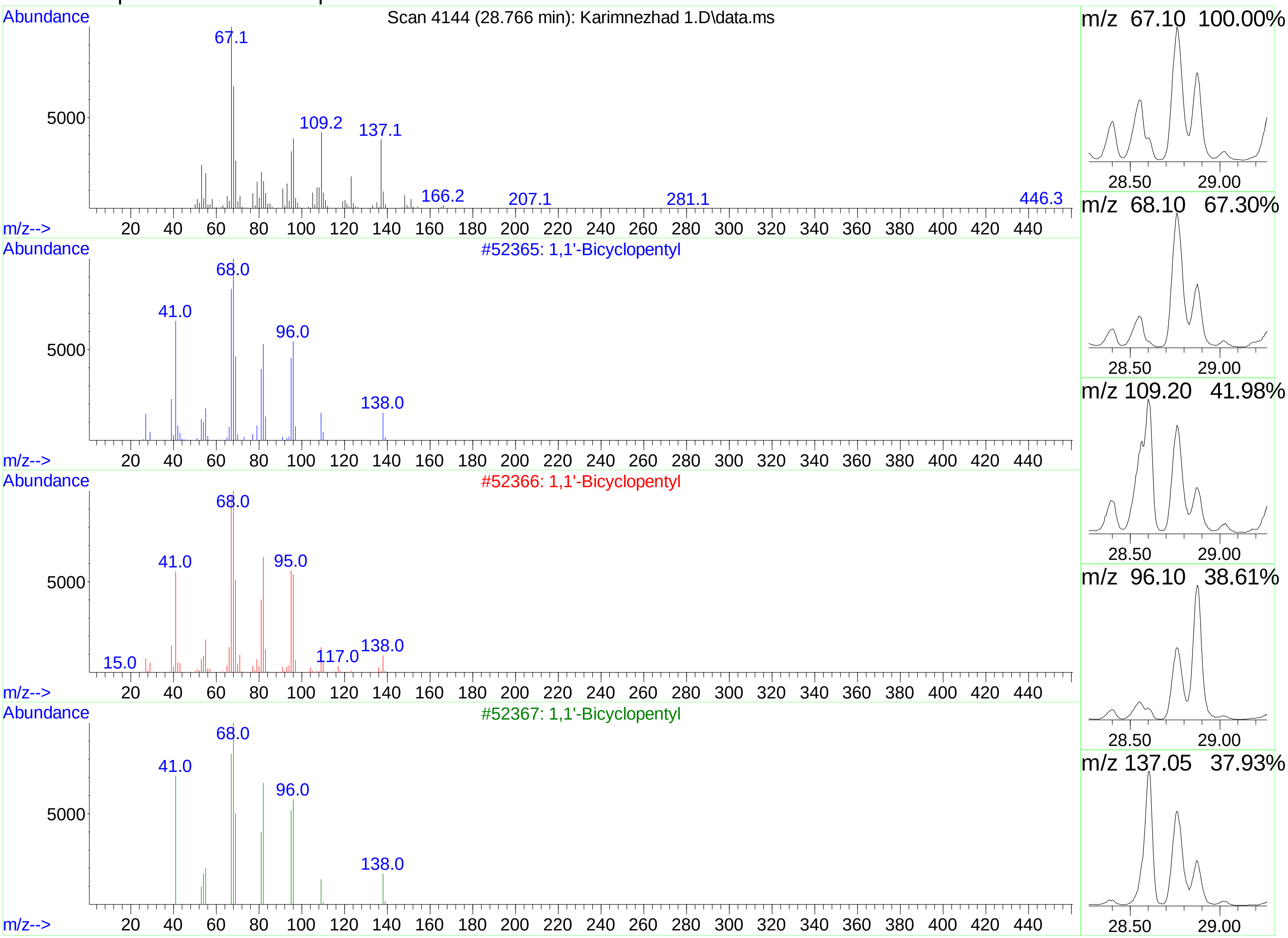

Data File: D:\msdchem\1\data\Karimnezhad 1.D
Sample : SDE

Peak Number: 28 at 28.766 min Area: 56039217 Area % 0.28

The 3 best hits from each library.	Ref\#	CAS\#	Qual
D:\Database\W10N14.L			
1 1,1'-Bicyclopentyl	52365	001636-39-1	55
2 1,1'-Bicyclopentyl	52366	001636-39-1	46
3 1,1'-Bicyclopentyl	52367	001636-39-1	46

Unknown Spectrum based on Apex

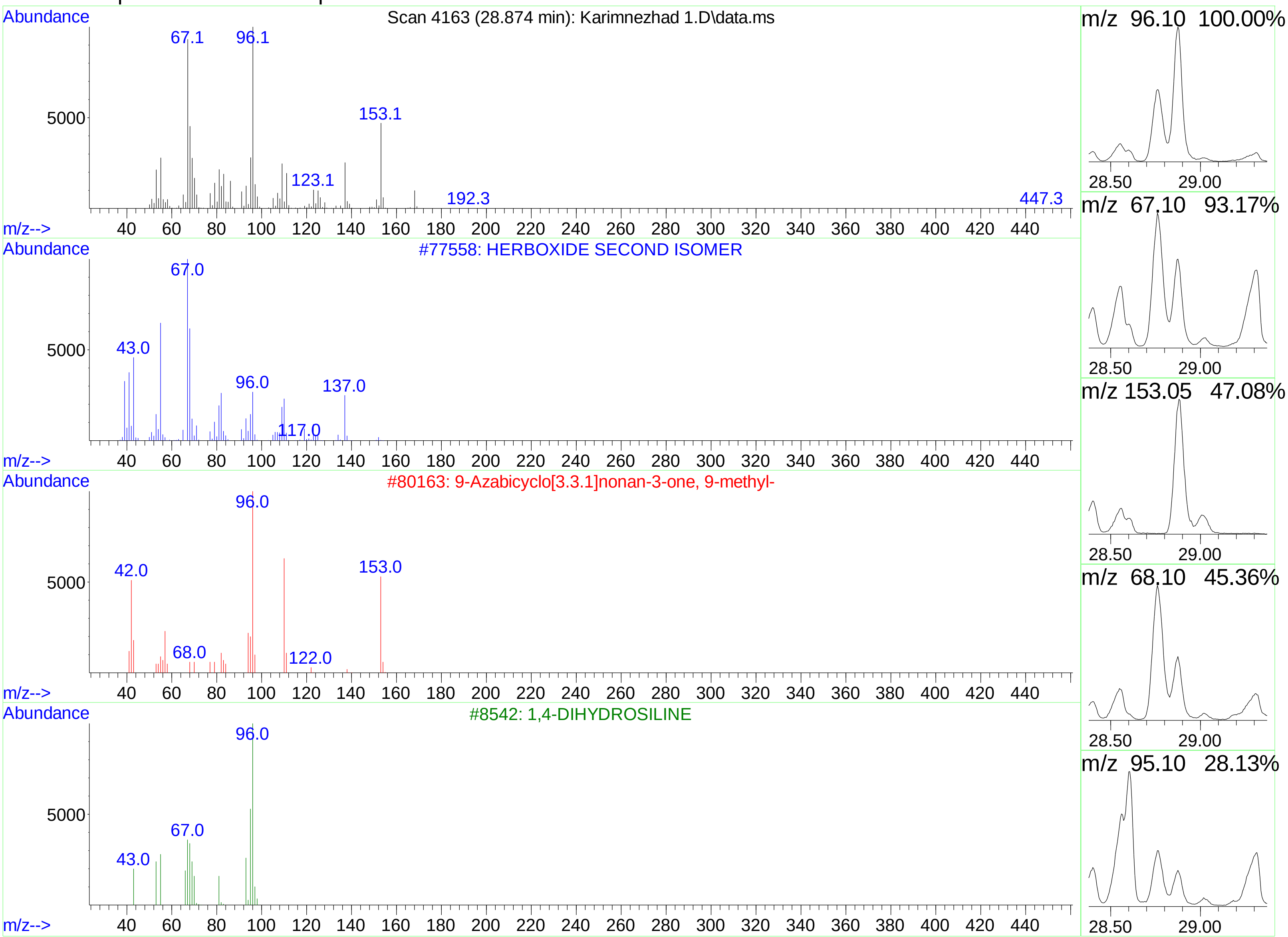

Data File: D:\msdchem\1\data\Karimnezhad 1.D
Sample : SDE

Peak Number: 29 at 28.874 min Area: 41818667 Area % 0.21

The 3 best hits from each library.	Ref\#	CAS\#	Qual
D:\Database\W10N14.L			
1 HERBOXIDE SECOND ISOMER	77558	013679-86-2	38
2 9-Azabicyclo[3.3.1]nonan-3-one, ...	80163	000552-70-5	30
3 1,4-DIHYDROSILINE	8542	081200-77-3	30

Library Search Report - ChemStation Integrator

Unknown Spectrum based on Apex

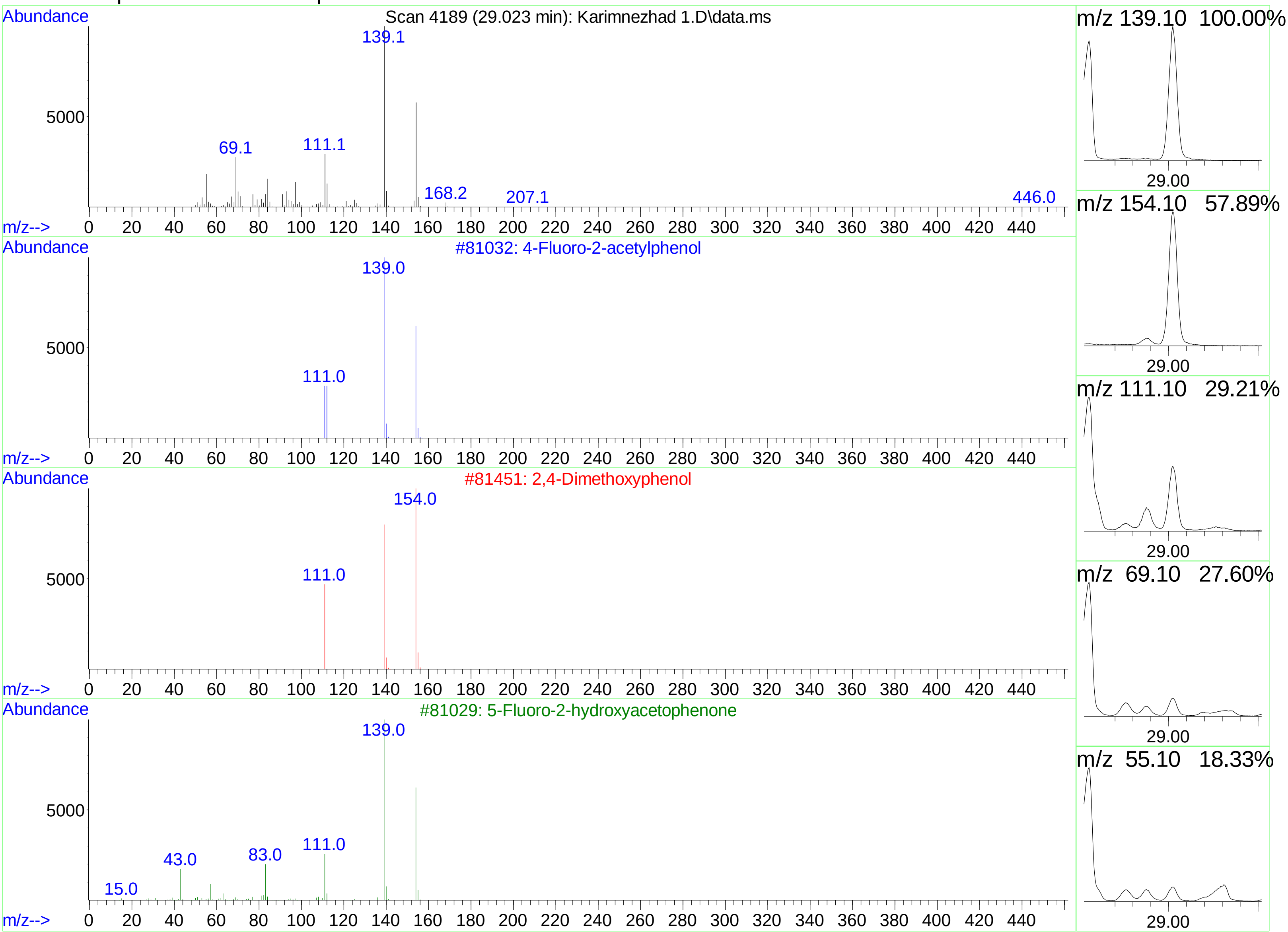

Data File: D:\msdchem\1\data\Karimnezhad 1.D
Sample : SDE

Peak Number: 30 at 29.023 min Area: 39003691 Area % 0.20

The 3 best hits from each library. Ref\# CAS\# Qual

D:\Database\W10N14.L

1 4-Fluoro-2-acetylphenol 81032 000394-32-1 80
2 2,4-Dimethoxyphenol 81451 013330-65-9 80
3 5-Fluoro-2-hydroxyacetophenone 81029 000394-32-1 74

Unknown Spectrum based on Apex

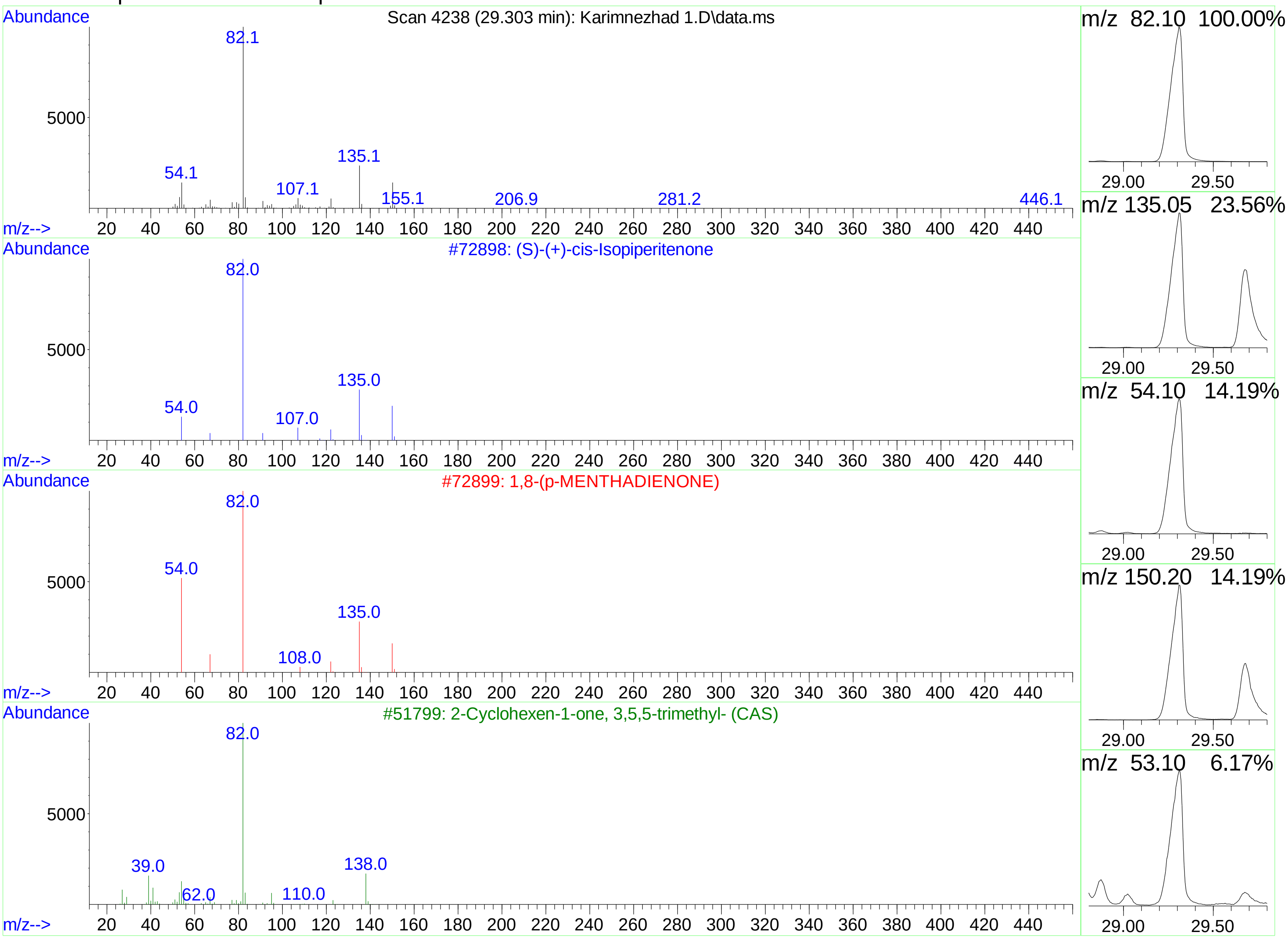

Data File: D:\msdchem\1\data\Karimnezhad 1.D
Sample : SDE

Peak Number: 31 at 29.303 min Area: 303110788 Area % 1.52

The 3 best hits from each library.	Ref#	CAS#	Qual
D:\Database\W10N14.L			
1 (S)-(+)-cis-Isopiperitenone	72898	2000072-89-8	91
2 1,8-(p-MENTHADIENONE)	72899	2000072-89-9	72
3 2-Cyclohexen-1-one, 3,5,5-trimet...	51799	000078-59-1	50

Unknown Spectrum based on Apex

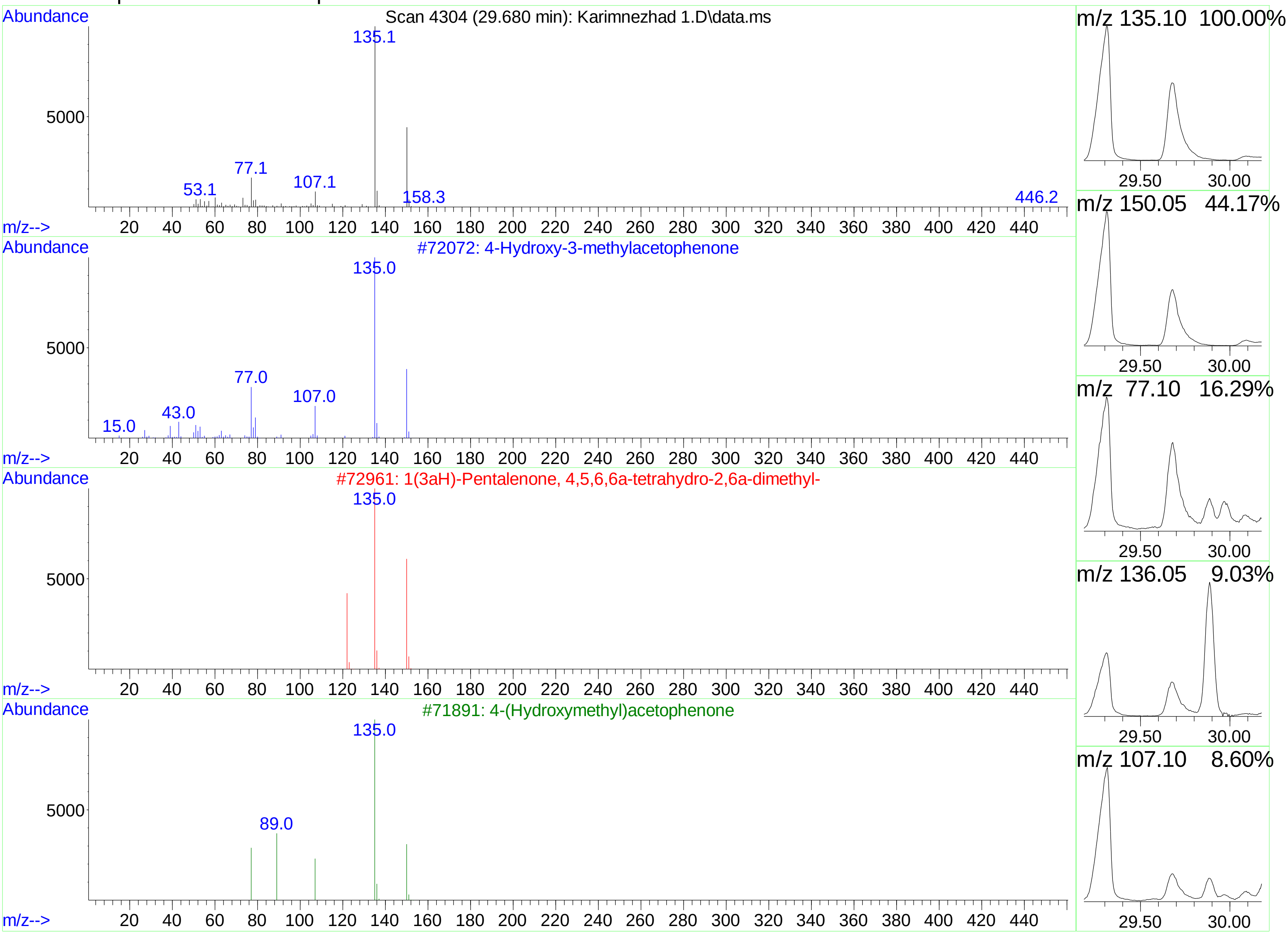

Data File: D:\msdchem\1\data\Karimnezhad 1.D
Sample : SDE

Peak Number: 32 at 29.680 min Area: 50012965 Area % 0.25

The 3 best hits from each library.	Ref#	CAS#	Qual
D:\Database\W10N14.L			
1 4-Hydroxy-3-methylacetophenone	72072	000876-02-8	90
2 1(3aH)-Pentalenone, 4,5,6,6a-tet...	72961	070640-02-7	90
3 4-(Hydroxymethyl)acetophenone	71891	075633-63-5	90

Unknown Spectrum based on Apex

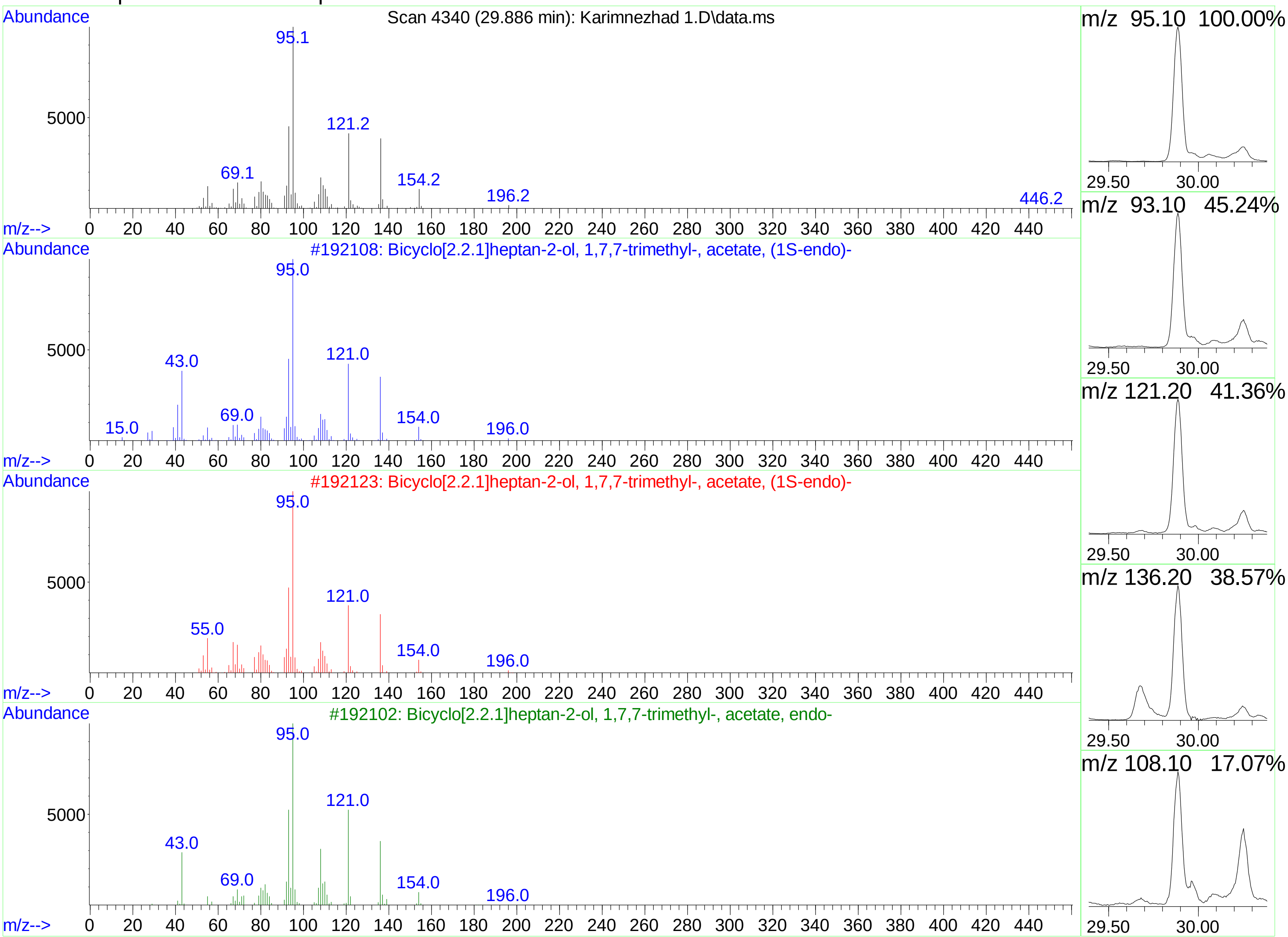

Data File: D:\msdchem\1\data\Karimnezhad 1.D
Sample : SDE

Peak Number: 33 at 29.886 min Area: 105049257 Area % 0.53

The 3 best hits from each library.	Ref\#	CAS\#	Qual
D:\Database\W10N14.L			
1 Bicyclo[2.2.1]heptan-2-ol, 1,7,7...	192108	005655-61-8	99
2 Bicyclo[2.2.1]heptan-2-ol, 1,7,7...	192123	005655-61-8	99
3 Bicyclo[2.2.1]heptan-2-ol, 1,7,7...	192102	000076-49-3	98

Unknown Spectrum based on Apex

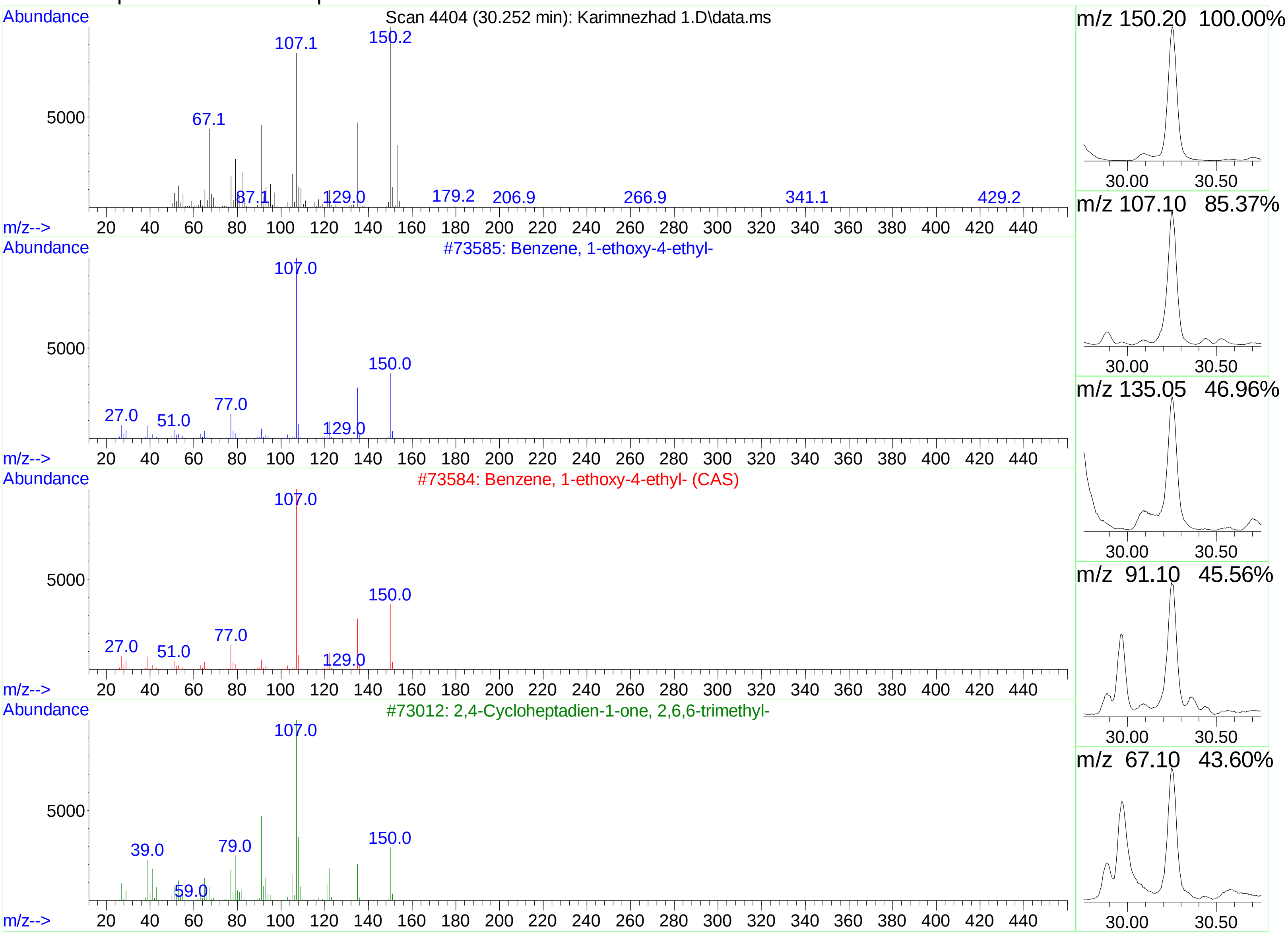

Data File: D:\msdchem\1\data\Karimnezhad 1.D
Sample : SDE

Peak Number: 34 at 30.252 min Area: 138524437 Area % 0.69

The 3 best hits from each library.	Ref#	CAS#	Qual
D:\Database\W10N14.L			
1 Benzene, 1-ethoxy-4-ethyl-	73585	001585-06-4	94
2 Benzene, 1-ethoxy-4-ethyl- (CAS)	73584	001585-06-4	94
3 2,4-Cycloheptadien-1-one, 2,6,6-...	73012	000503-93-5	86

Unknown Spectrum based on Apex

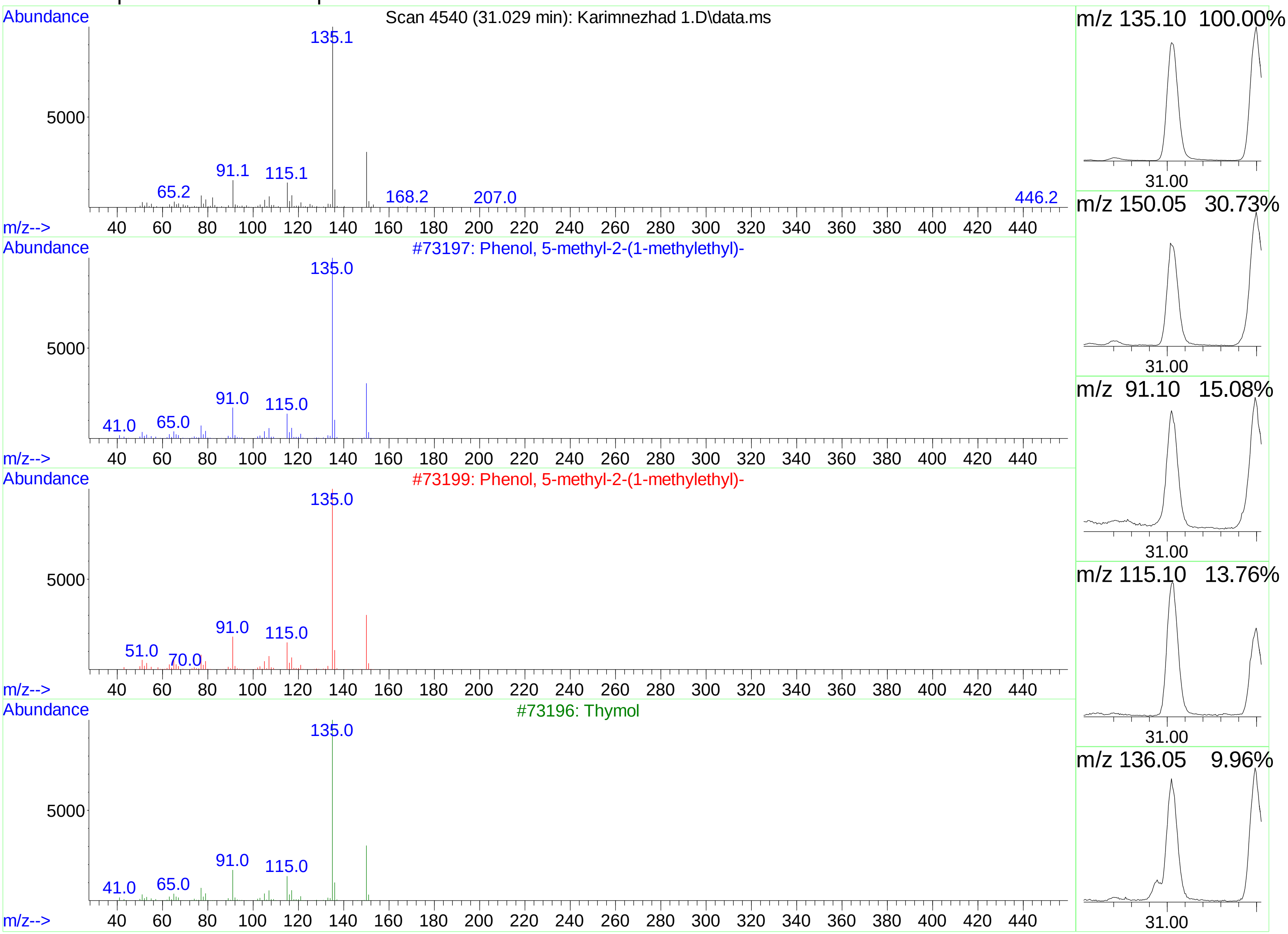

Data File: D:\msdchem\1\data\Karimnezhad 1.D
Sample : SDE

Peak Number: 35 at 31.029 min Area: 62245552 Area % 0.31

The 3 best hits from each library.	Ref\#	CAS\#	Qual
D:\Database\W10N14.L			
1 Phenol, 5-methyl-2-(1-methylethyl)-	73197	000089-83-8	95
2 Phenol, 5-methyl-2-(1-methylethyl)-	73199	000089-83-8	95
3 Thymol	73196	000089-83-8	95

Unknown Spectrum based on Apex

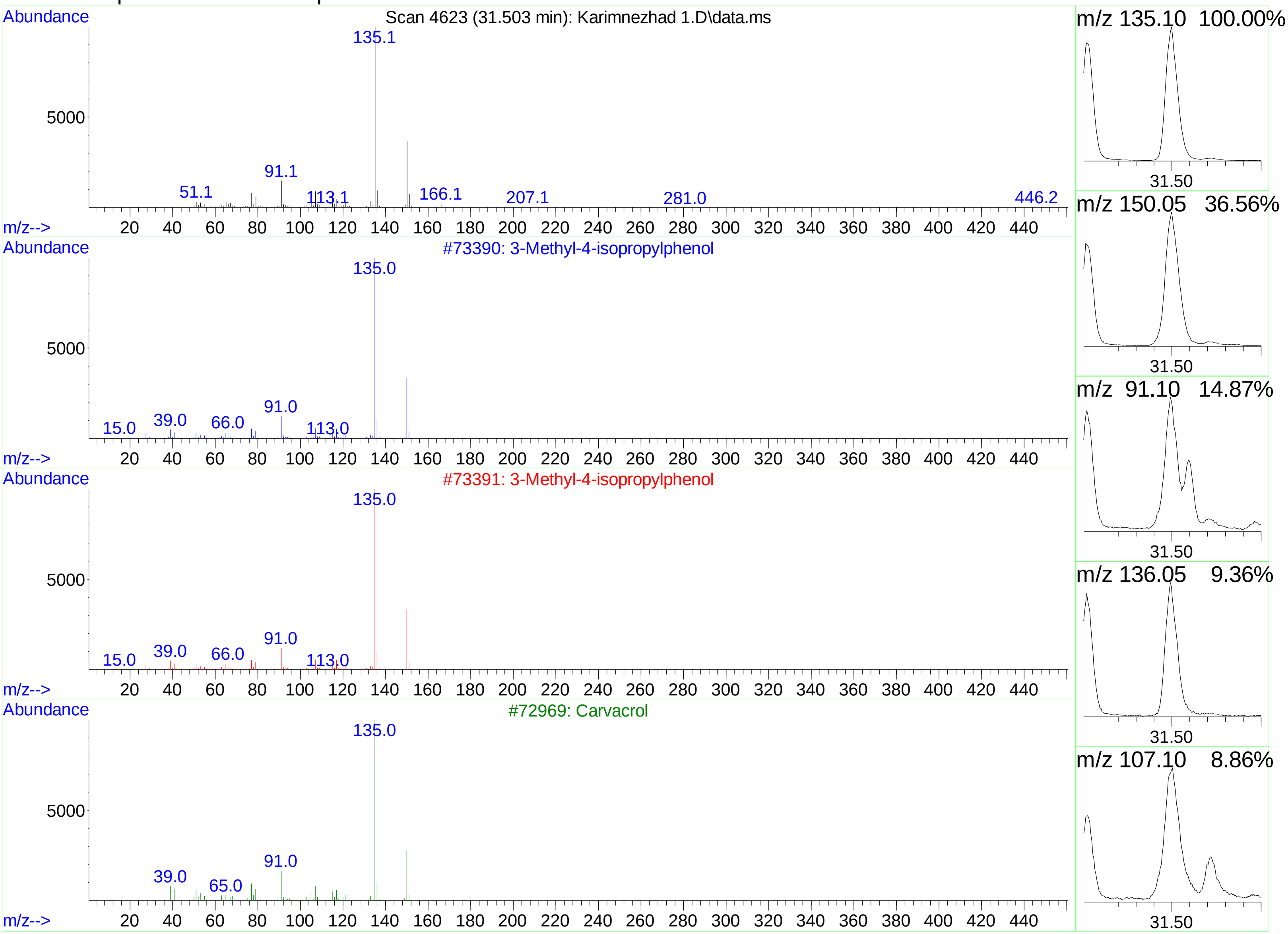

Data File: D:\msdchem\1\data\Karimnezhad 1.D
Sample : SDE

Peak Number: 36 at 31.503 min Area: 69668614 Area % 0.35

The 3 best hits from each library. Ref\# CAS\# Qual

D:\Database\W10N14.L

1	3-Methyl-4-isopropylphenol	73390	003228-02-2	95
2	3-Methyl-4-isopropylphenol	73391	003228-02-2	94
3	Carvacrol	72969	000499-75-2	94

Unknown Spectrum based on Apex

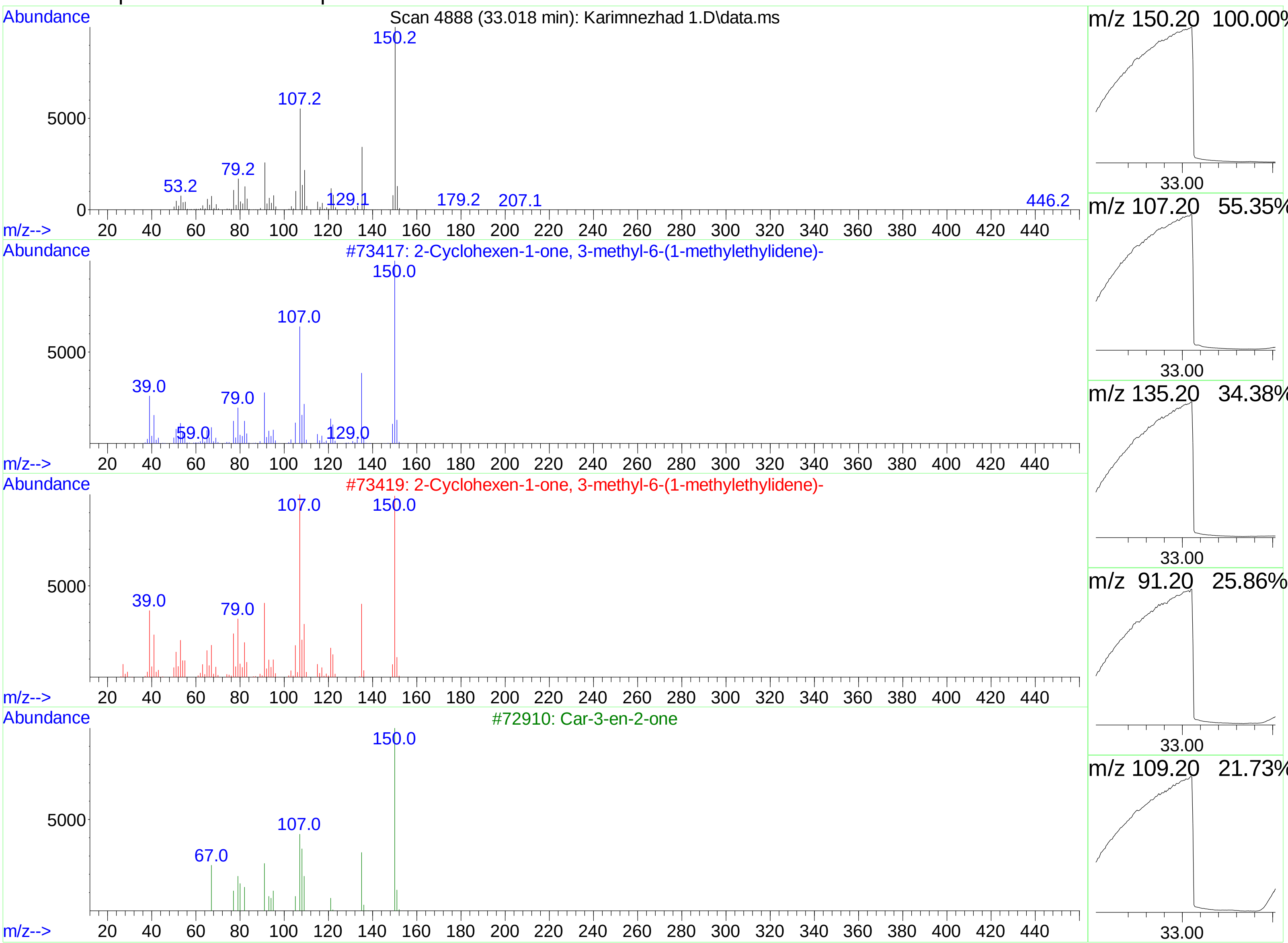

Data File: D:\msdchem\1\data\Karimnezhad 1.D
Sample : SDE

Peak Number: 37 at 33.018 min Area: 7602164727 Area % 38.06

The 3 best hits from each library. Ref\# CAS\# Qual

D:\Database\W10N14.L

1 2-Cyclohexen-1-one, 3-methyl-6-(... 73417 000491-09-8 98
2 2-Cyclohexen-1-one, 3-methyl-6-(... 73419 000491-09-8 95
3 Car-3-en-2-one 72910 2000072-91-0 90

Unknown Spectrum based on Apex

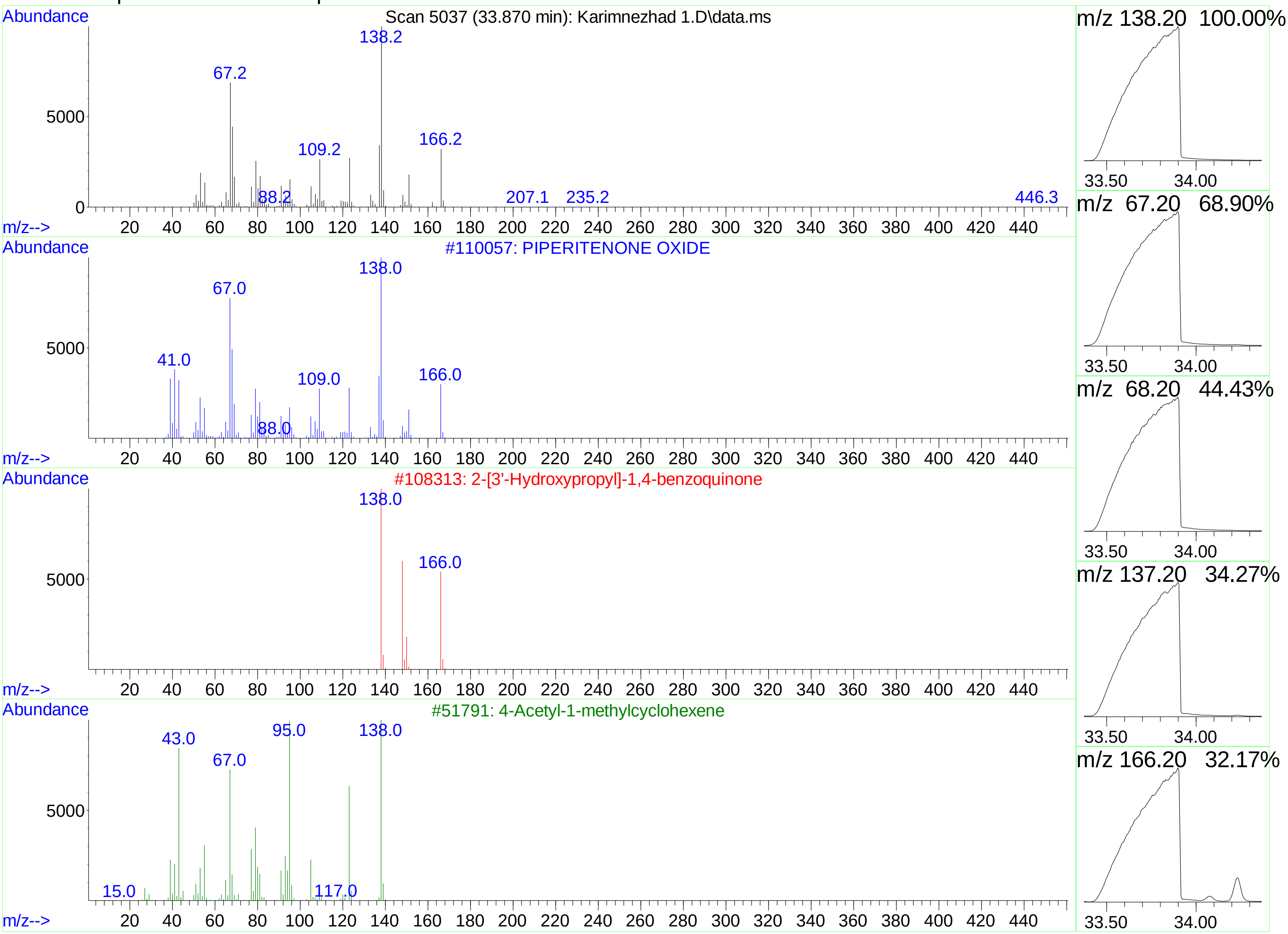

Data File: D:\msdchem\1\data\Karimnezhad 1.D
Sample : SDE

Peak Number: 38 at 33.870 min Area: 3952644428 Area % 19.79

The 3 best hits from each library.	Ref\#	CAS\#	Qual
D:\Database\W10N14.L			
1 PIPERITENONE OXIDE	110057	003564-96-3	99
2 2-[3'-Hydroxypropyl]-1,4-benzoqu...	108313	2000108-31-3	86
3 4-Acetyl-1-methylcyclohexene	51791	006090-09-1	60

Unknown Spectrum based on Apex

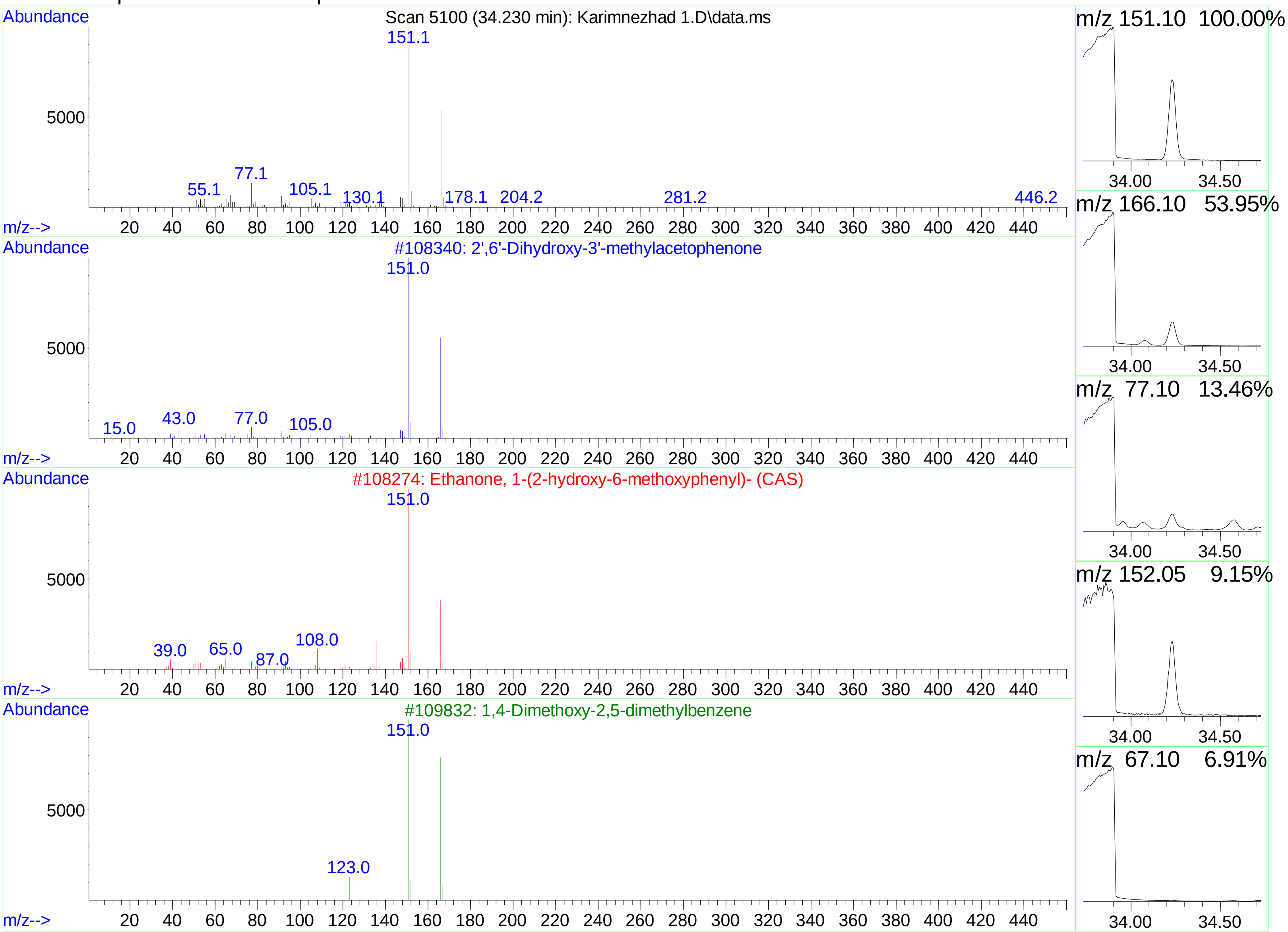

Data File: D:\msdchem\1\data\Karimnezhad 1.D
Sample : SDE

Peak Number: 39 at 34.230 min Area: 52892711 Area % 0.26

The 3 best hits from each library. Ref\# CAS\# Qual

D:\Database\W10N14.L

1 2',6'-Dihydroxy-3'-methylacetoph... 108340 029183-78-6 87
2 Ethanone, 1-(2-hydroxy-6-methoxy... 108274 000703-23-1 80
3 1,4-Dimethoxy-2,5-dimethylbenzene 109832 2000109-83-2 80

Unknown Spectrum based on Apex

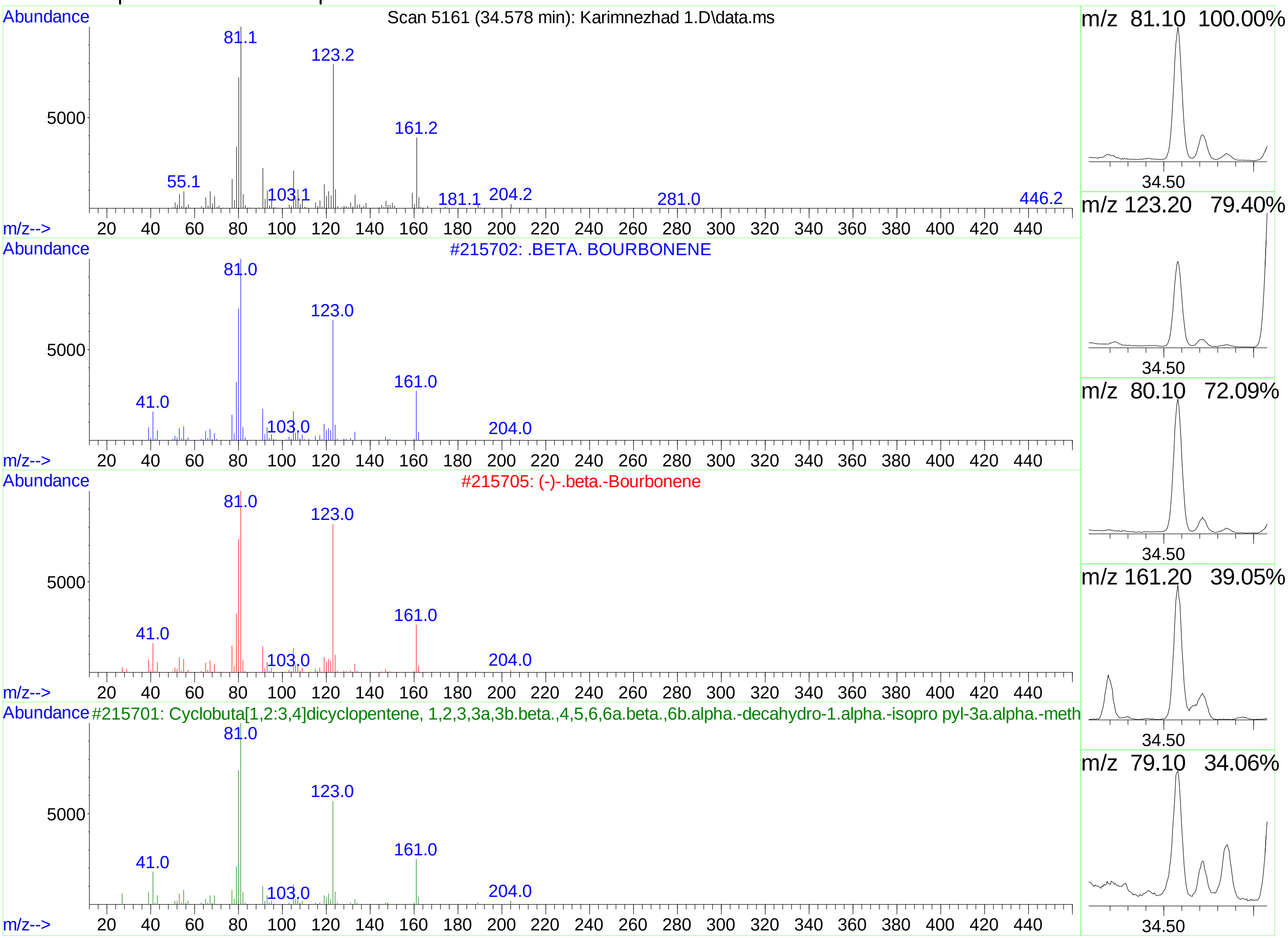

Data File: D:\msdchem\1\data\Karimnezhad 1.D
Sample : SDE

Peak Number: 40 at 34.578 min Area: 56249209 Area % 0.28

The 3 best hits from each library.	Ref\#	CAS\#	Qual
D:\Database\W10N14.L			
1 .BETA. BOURBONENE	215702	005208-59-3	99
2 (-)-.beta.-Bourbonene	215705	005208-59-3	96
3 Cyclobuta[1,2:3,4]dicyclopentene...	215701	005208-59-3	95

Unknown Spectrum based on Apex

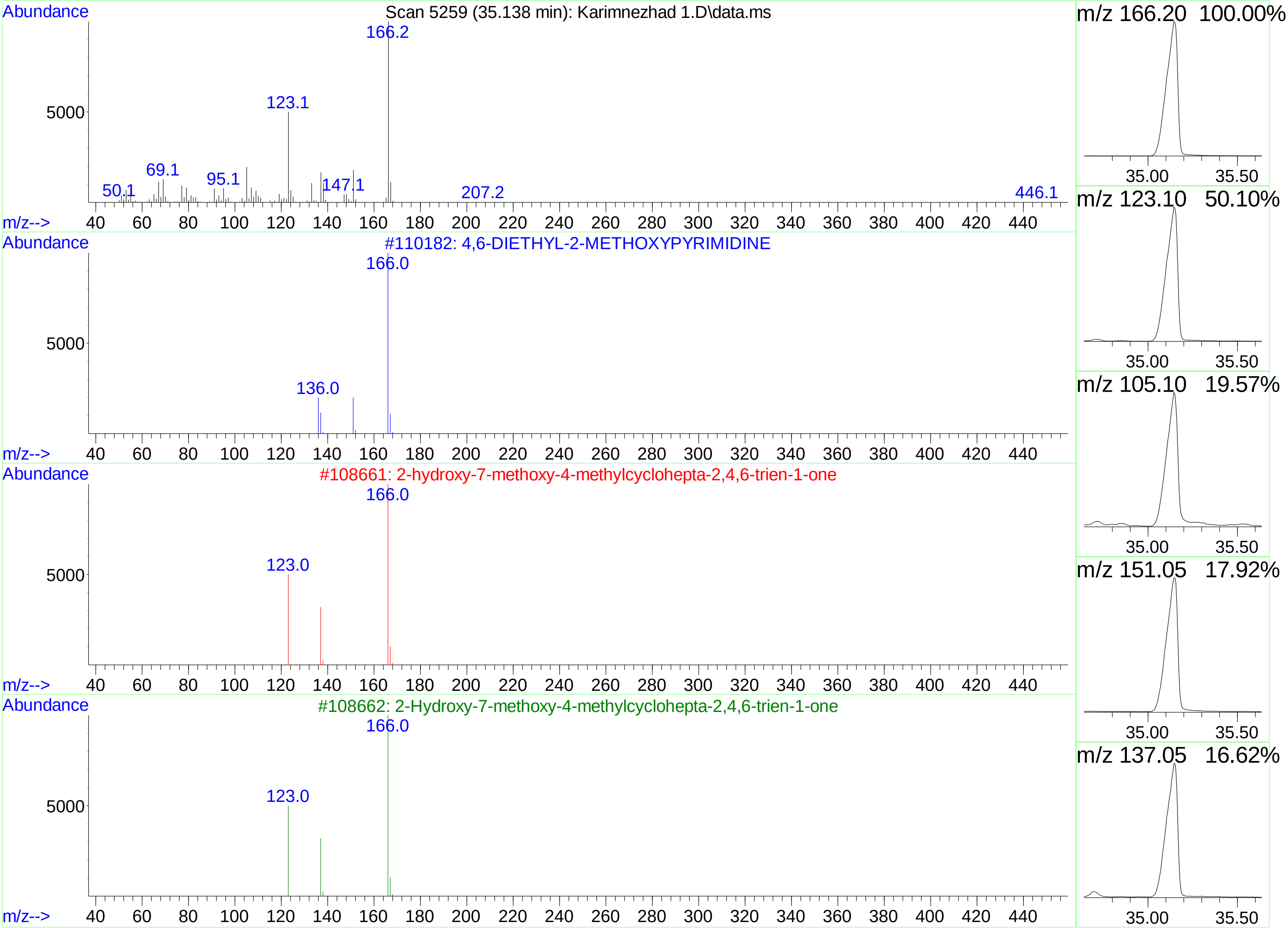

Data File: D:\msdchem\1\data\Karimnezhad 1.D
Sample : SDE

Peak Number: 41 at 35.138 min Area: 375903996 Area % 1.88

The 3 best hits from each library.	Ref\#	CAS\#	Qual
D:\Database\W10N14.L			
1 4,6-DIETHYL-2-METHOXYPYRIMIDINE	110182	2000110-18-2	72
2 2-hydroxy-7-methoxy-4-methylcycl...	108661	2000108-66-1	64
3 2-Hydroxy-7-methoxy-4-methylcycl...	108662	2000108-66-2	64

Library Search Report - ChemStation Integrator

Unknown Spectrum based on Apex

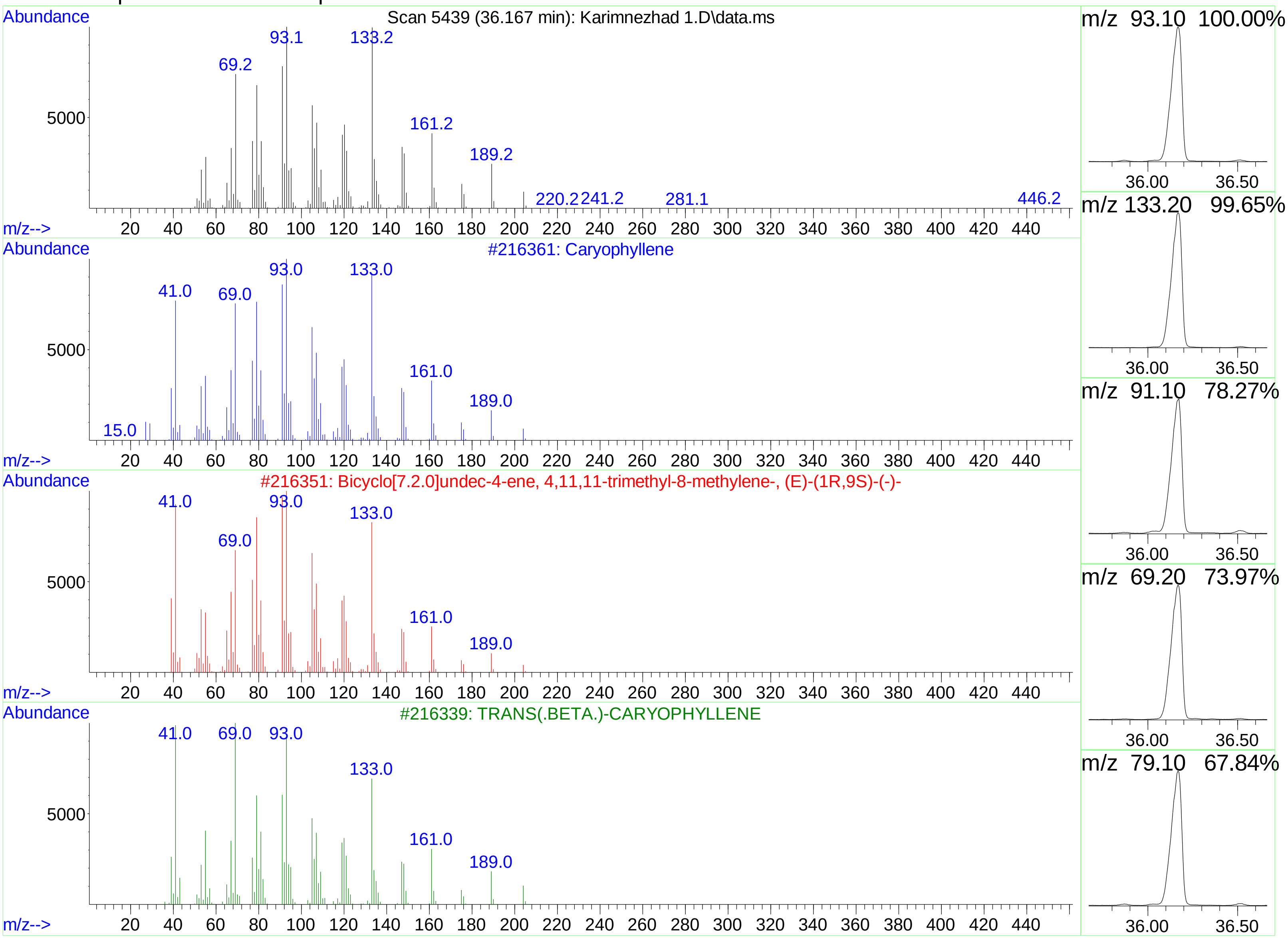

Data File: D:\msdchem\1\data\Karimnezhad 1.D
Sample : SDE

Peak Number: 42 at 36.167 min Area: 417122559 Area % 2.09

The 3 best hits from each library.	Ref\#	CAS\#	Qual
D:\Database\W10N14.L			
1 Caryophyllene	216361	000087-44-5	99
2 Bicyclo[7.2.0]undec-4-ene, 4,11,...	216351	000087-44-5	99
3 TRANS(.BETA.)-CARYOPHYLLENE	216339	2000216-33-9	99

Library Search Report - ChemStation Integrator

Unknown Spectrum based on Apex

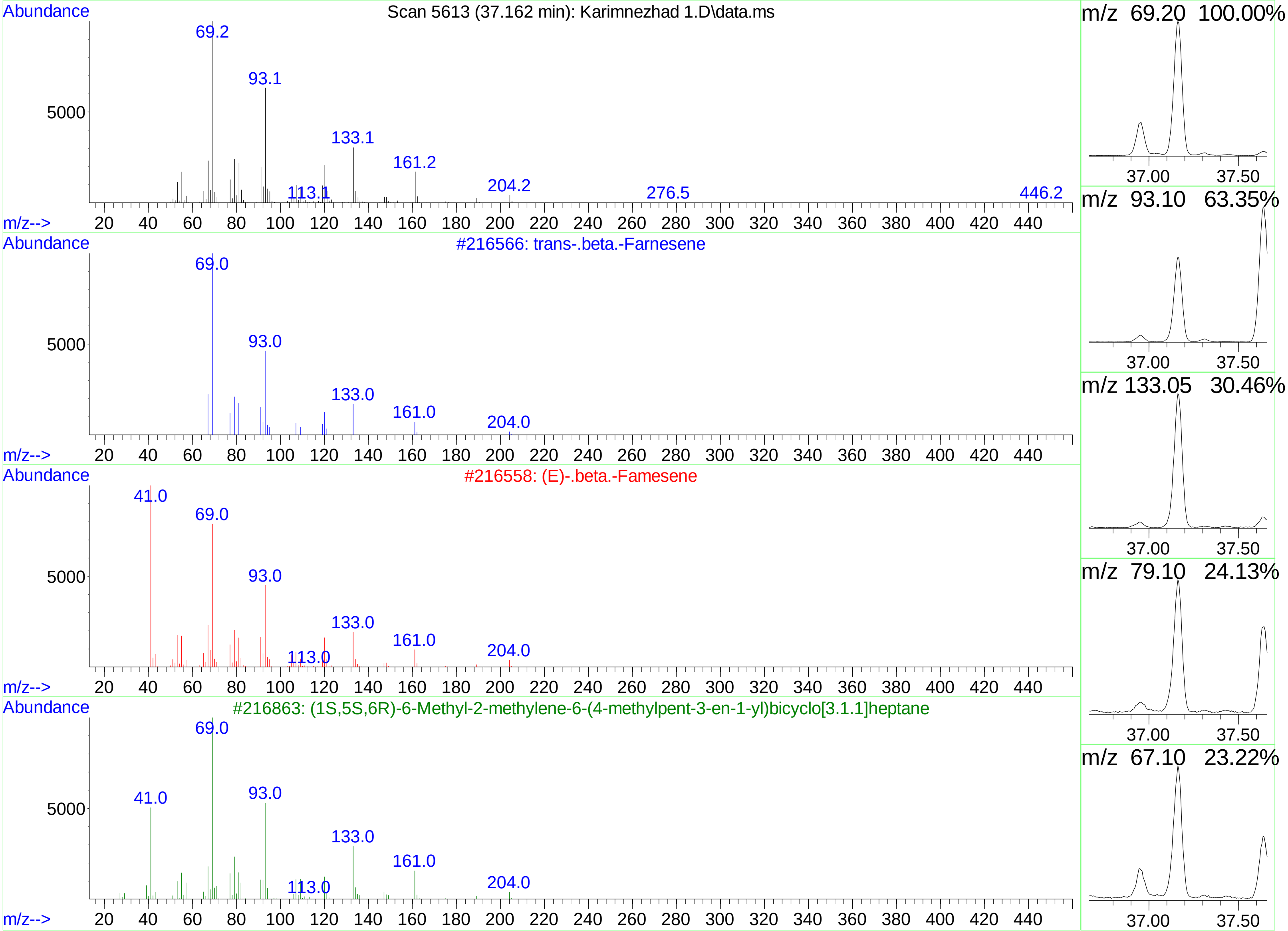

Data File: D:\msdchem\1\data\Karimnezhad 1.D
Sample : SDE

Peak Number: 43 at 37.162 min Area: 58998980 Area % 0.30

The 3 best hits from each library.	Ref#	CAS#	Qual
D:\Database\W10N14.L			
1 trans-.beta.-Farnesene	216566	000502-60-3	96
2 (E)-.beta.-Famesene	216558	018794-84-8	96
3 (1S,5S,6R)-6-Methyl-2-methylene-...	216863	015438-94-5	95

Unknown Spectrum based on Apex

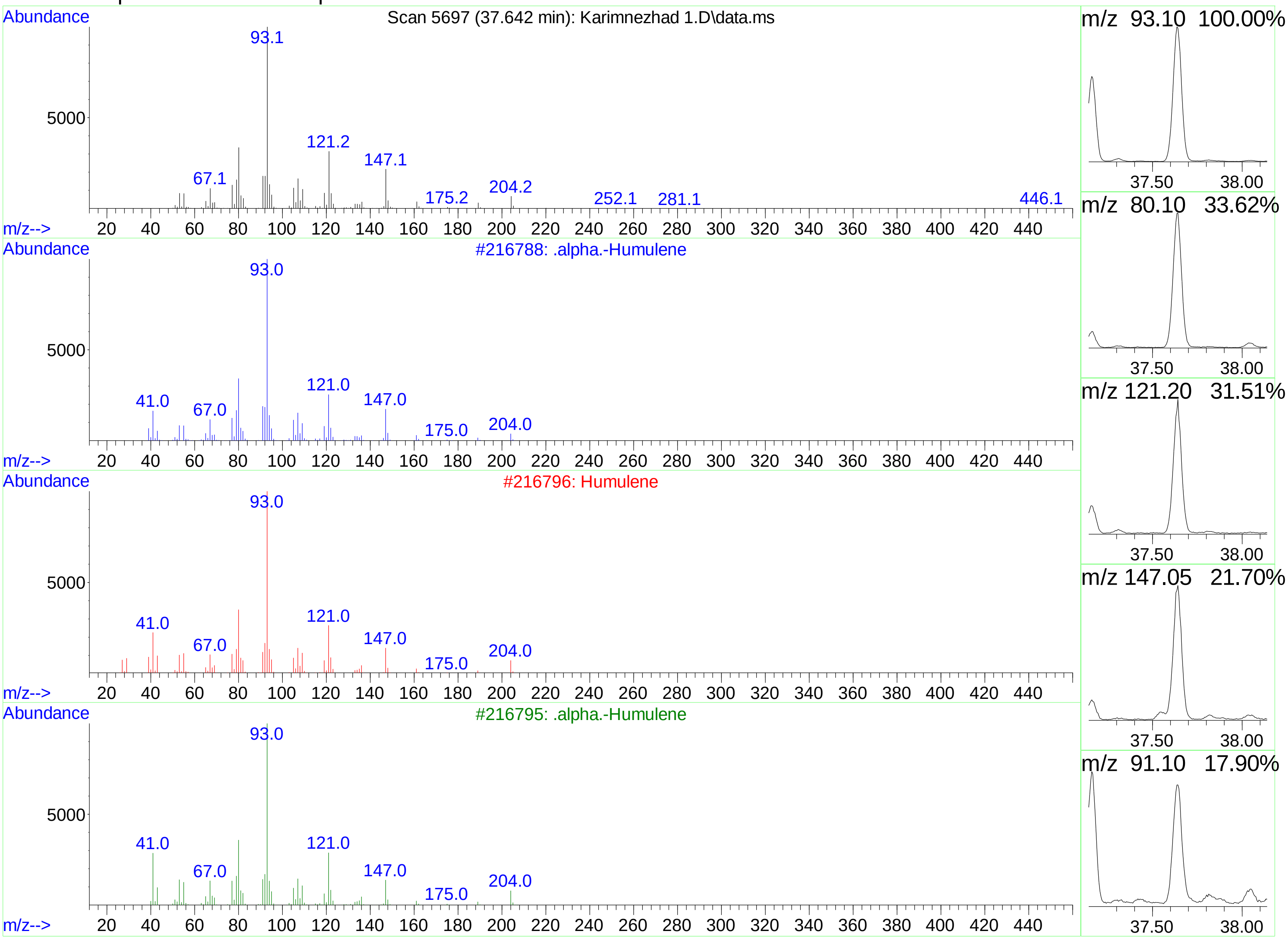

Data File: D:\msdchem\1\data\Karimnezhad 1.D
Sample : SDE

Peak Number: 44 at 37.642 min Area: 62118411 Area % 0.31

The 3 best hits from each library.	Ref\#	CAS\#	Qual
D:\Database\W10N14.L			
1 .alpha.-Humulene	216788	006753-98-6	99
2 Humulene	216796	006753-98-6	98
3 .alpha.-Humulene	216795	006753-98-6	98

Unknown Spectrum based on Apex

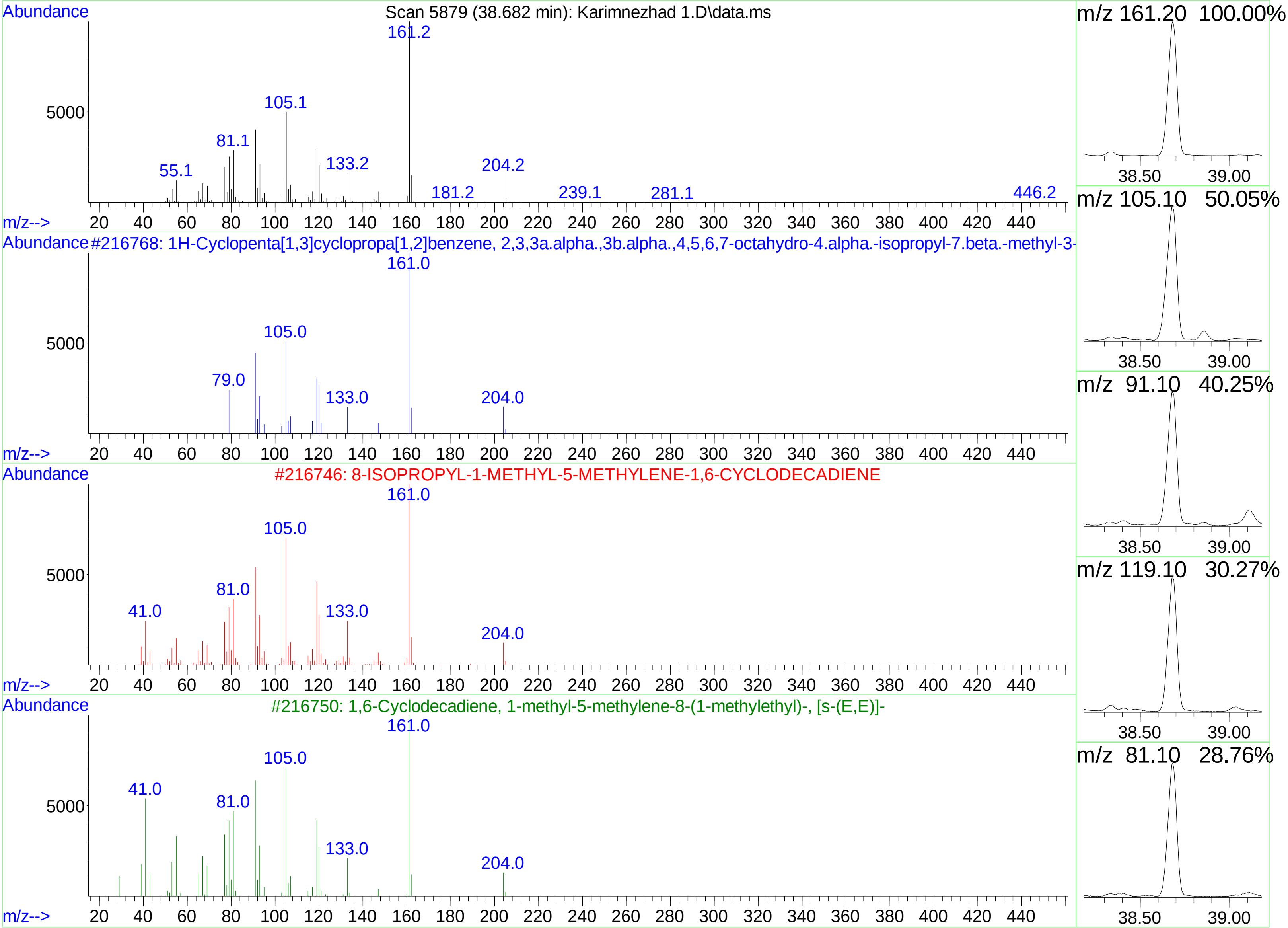

Data File: D:\msdchem\1\data\Karimnezhad 1.D
Sample : SDE

Peak Number: 45 at 38.682 min Area: 130972127 Area % 0.66

The 3 best hits from each library. Ref\# CAS\# Qual

D:\Database\W10N14.L

1	1H-Cyclopenta[1,3]cyclopropa[1,2...	216768	013744-15-5	99
2	8-ISOPROPYL-1-METHYL-5-METHYLENE...	216746	023986-74-5	98
3	1,6-Cyclodecadiene, 1-methyl-5-m...	216750	023986-74-5	98

Unknown Spectrum based on Apex

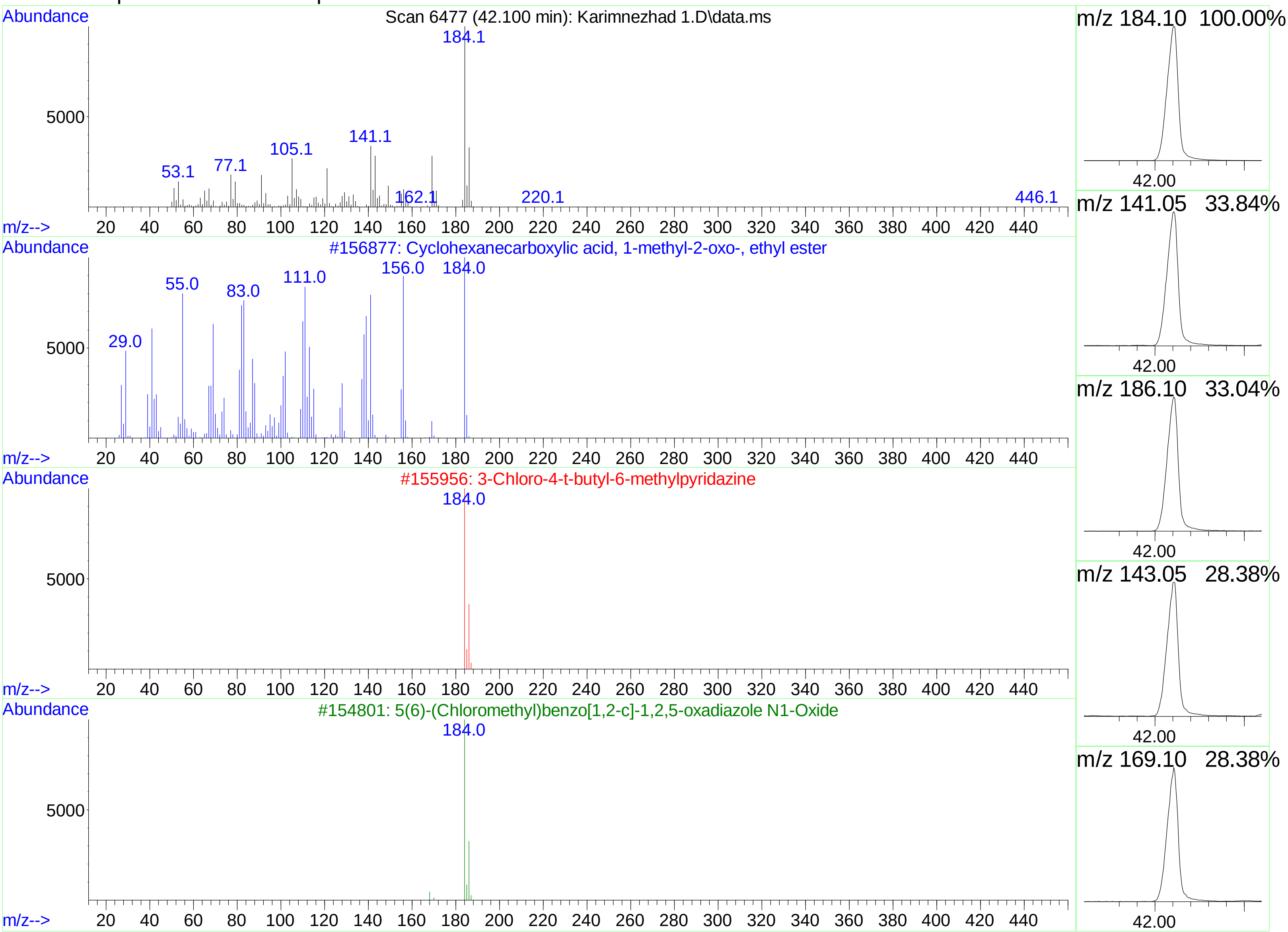

Data File: D:\msdchem\1\data\Karimnezhad 1.D
Sample : SDE

Peak Number: 46 at 42.100 min Area: 136329861 Area % 0.68

The 3 best hits from each library.	Ref\#	CAS\#	Qual
D:\Database\W10N14.L			
1 Cyclohexanecarboxylic acid, 1-me...	156877	005453-94-1	83
2 3-Chloro-4-t-butyl-6-methylpyrid...	155956	2000155-95-6	83
3 5(6)-(Chloromethyl)benzo[1,2-c]-...	154801	2000154-80-1	59

Unknown Spectrum based on Apex

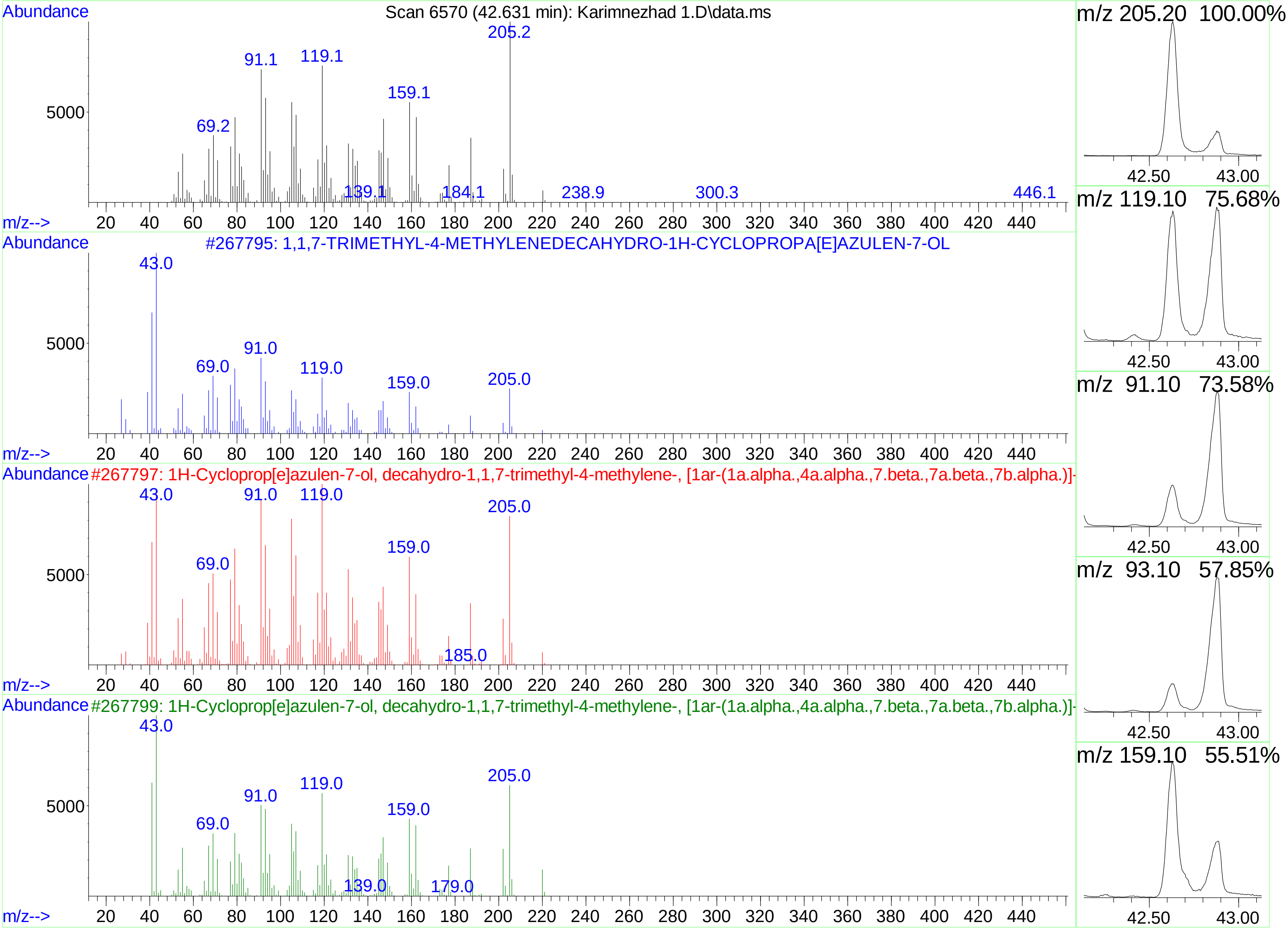

Data File: D:\msdchem\1\data\Karimnezhad 1.D
Sample : SDE

Peak Number: 47 at 42.631 min Area: 117863442 Area % 0.59

The 3 best hits from each library. Ref\# CAS\# Qual

D:\Database\W10N14.L

1	1,1,7-TRIMETHYL-4-METHYLENEDECAH...	267795	077171-55-2	98
2	1H-Cycloprop[e]azulen-7-ol, deca...	267797	006750-60-3	96
3	1H-Cycloprop[e]azulen-7-ol, deca...	267799	006750-60-3	93

Unknown Spectrum based on Apex

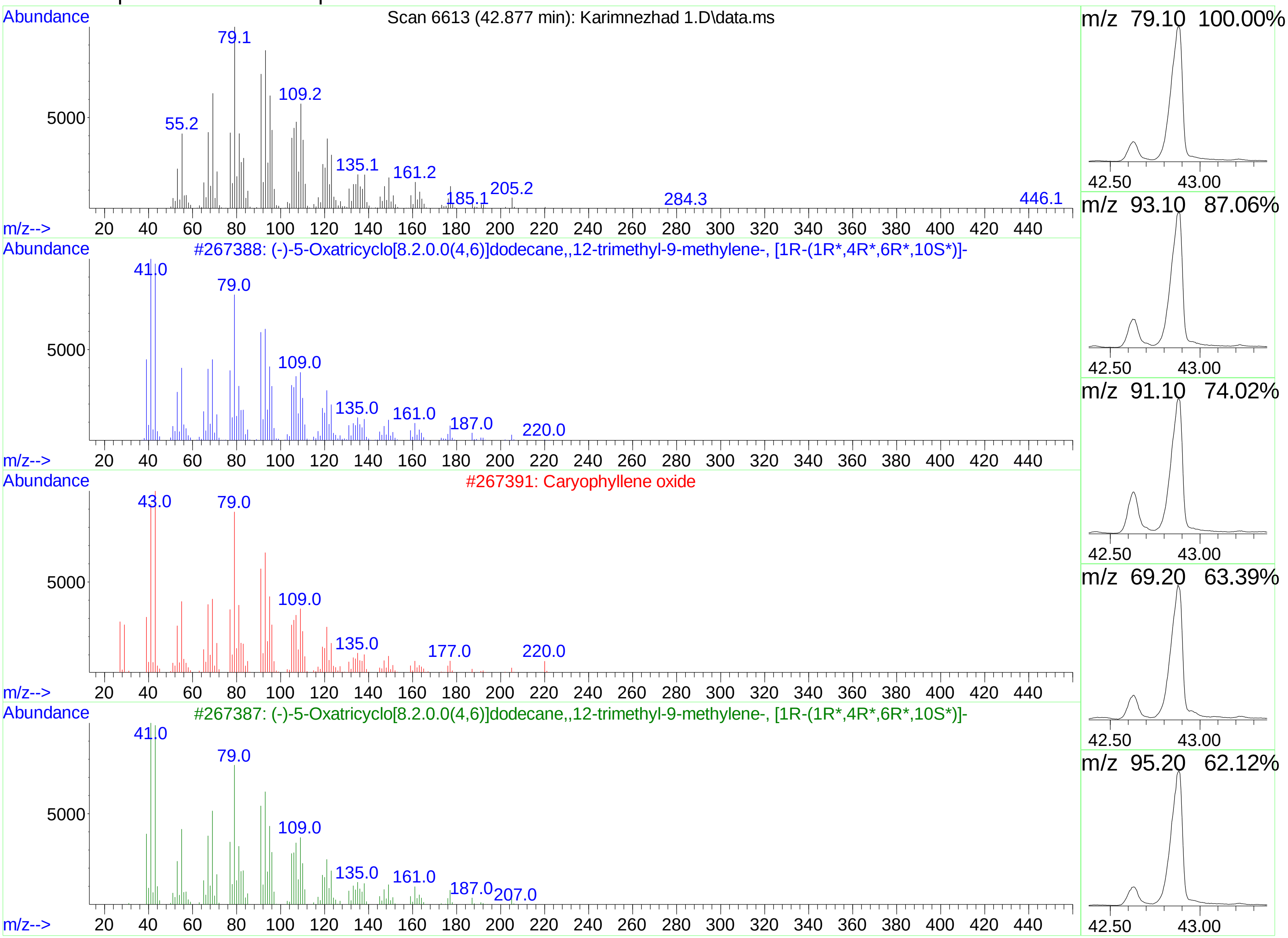

Data File: D:\msdchem\1\data\Karimnezhad 1.D
Sample : SDE

Peak Number: 48 at 42.877 min Area: 394355361 Area % 1.97

The 3 best hits from each library.	Ref\#	CAS\#	Qual
D:\Database\W10N14.L			
1 (-)-5-Oxatricyclo[8.2.0.0(4,6)]d...	267388	001139-30-6	99
2 Caryophyllene oxide	267391	001139-30-6	99
3 (-)-5-Oxatricyclo[8.2.0.0(4,6)]d...	267387	001139-30-6	95

Unknown Spectrum based on Apex

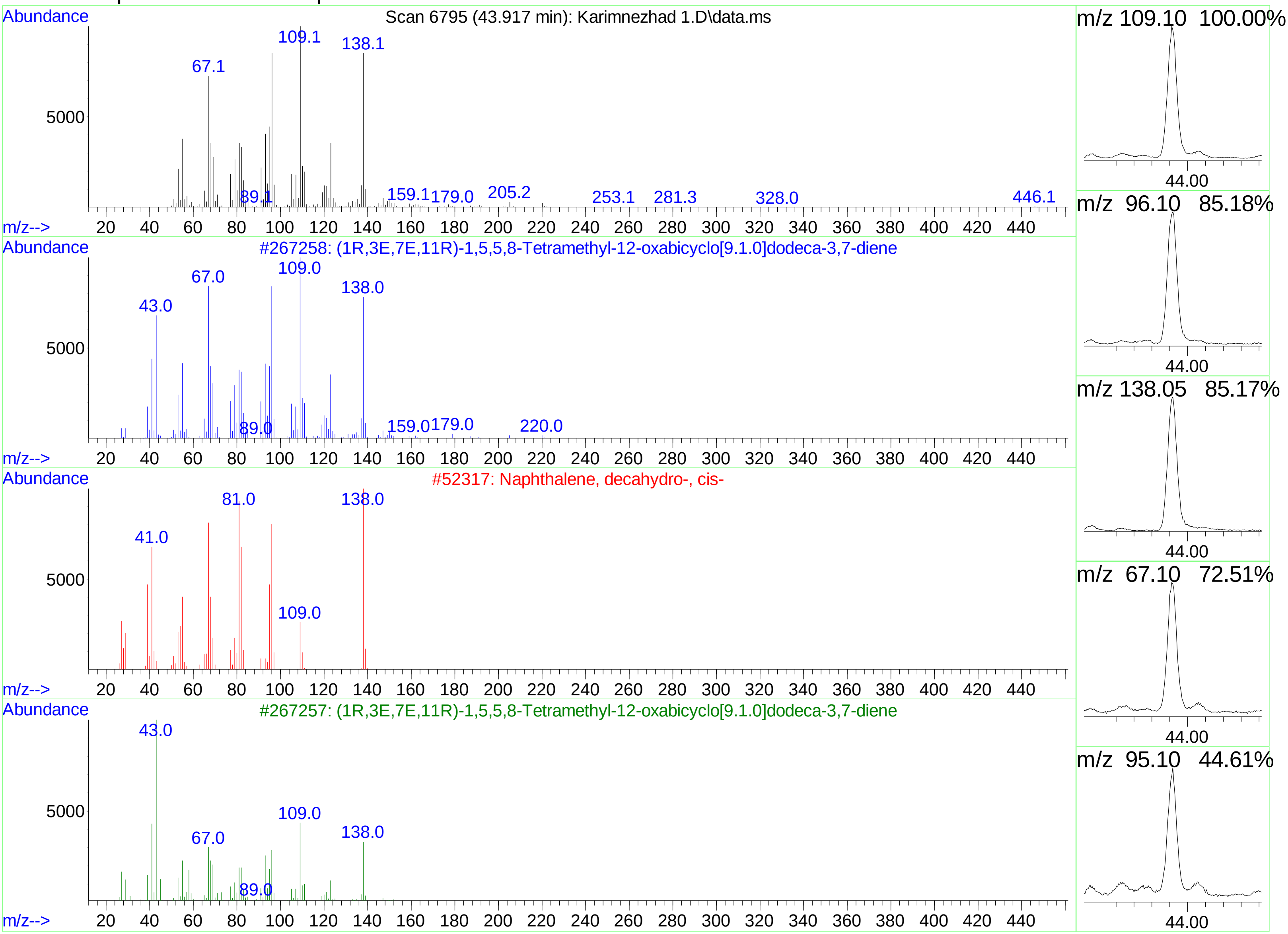

Data File: D:\msdchem\1\data\Karimnezhad 1.D
Sample : SDE

Peak Number: 49 at 43.917 min Area: 42034622 Area % 0.21

The 3 best hits from each library. Ref\# CAS\# Qual

D:\Database\W10N14.L

1 (1R,3E,7E,11R)-1,5,5,8-Tetrameth... 267258 019888-34-7 99
2 Naphthalene, decahydro-, cis- 52317 000493-01-6 78
3 (1R,3E,7E,11R)-1,5,5,8-Tetrameth... 267257 019888-34-7 74

Unknown Spectrum based on Apex

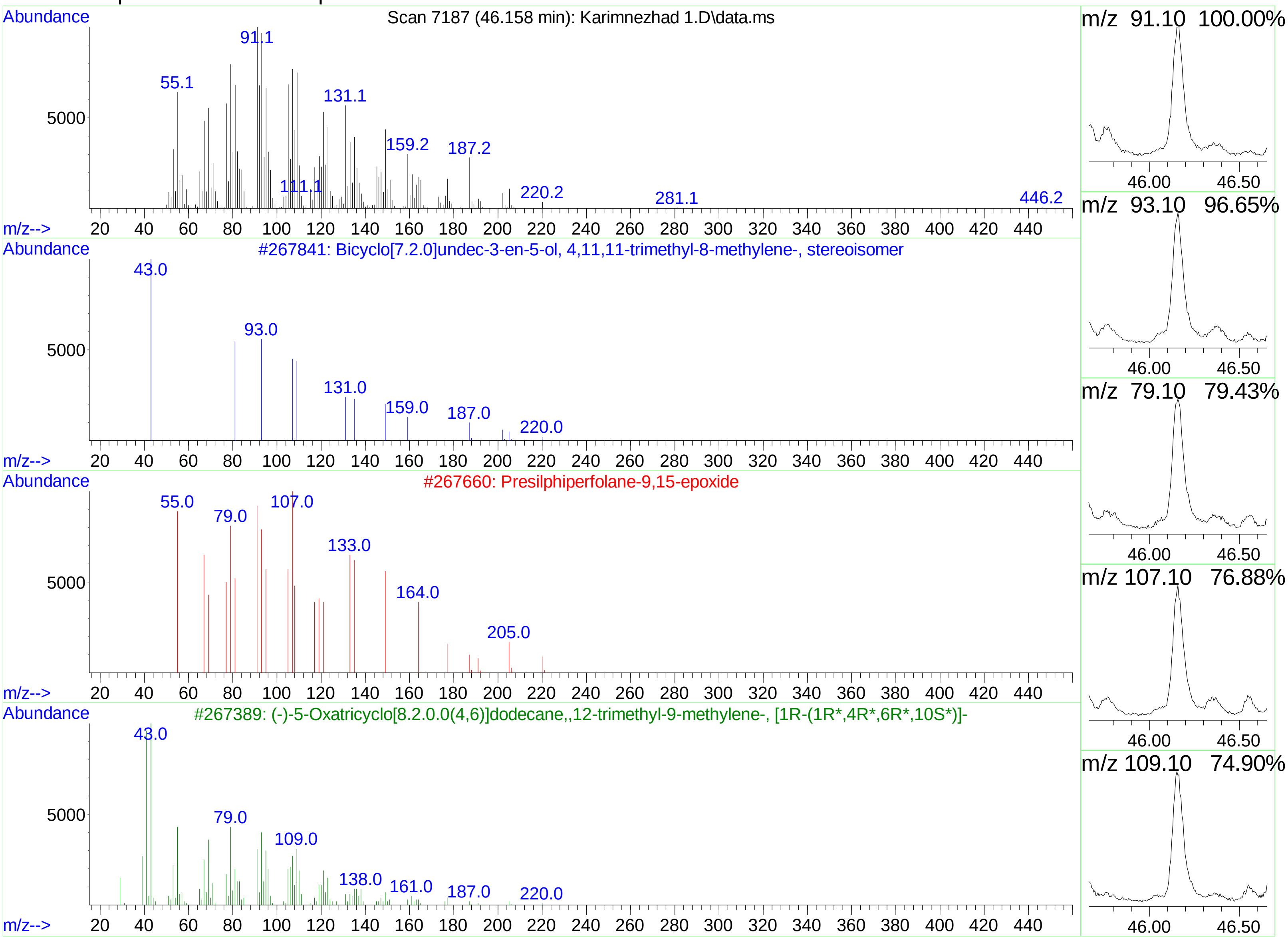

Data File: D:\msdchem\1\data\Karimnezhad 1.D
Sample : SDE

Peak Number: 50 at 46.158 min Area: 50505790 Area % 0.25

The 3 best hits from each library.	Ref\#	CAS\#	Qual
D:\Database\W10N14.L			
1 Bicyclo[7.2.0]undec-3-en-5-ol, 4...	267841	032214-89-4	96
2 Presilphiperfolane-9,15-epoxide	267660	2000267-66-0	87
3 (-)-5-Oxatricyclo[8.2.0.0(4,6)]d...	267389	001139-30-6	83

Unknown Spectrum based on Apex

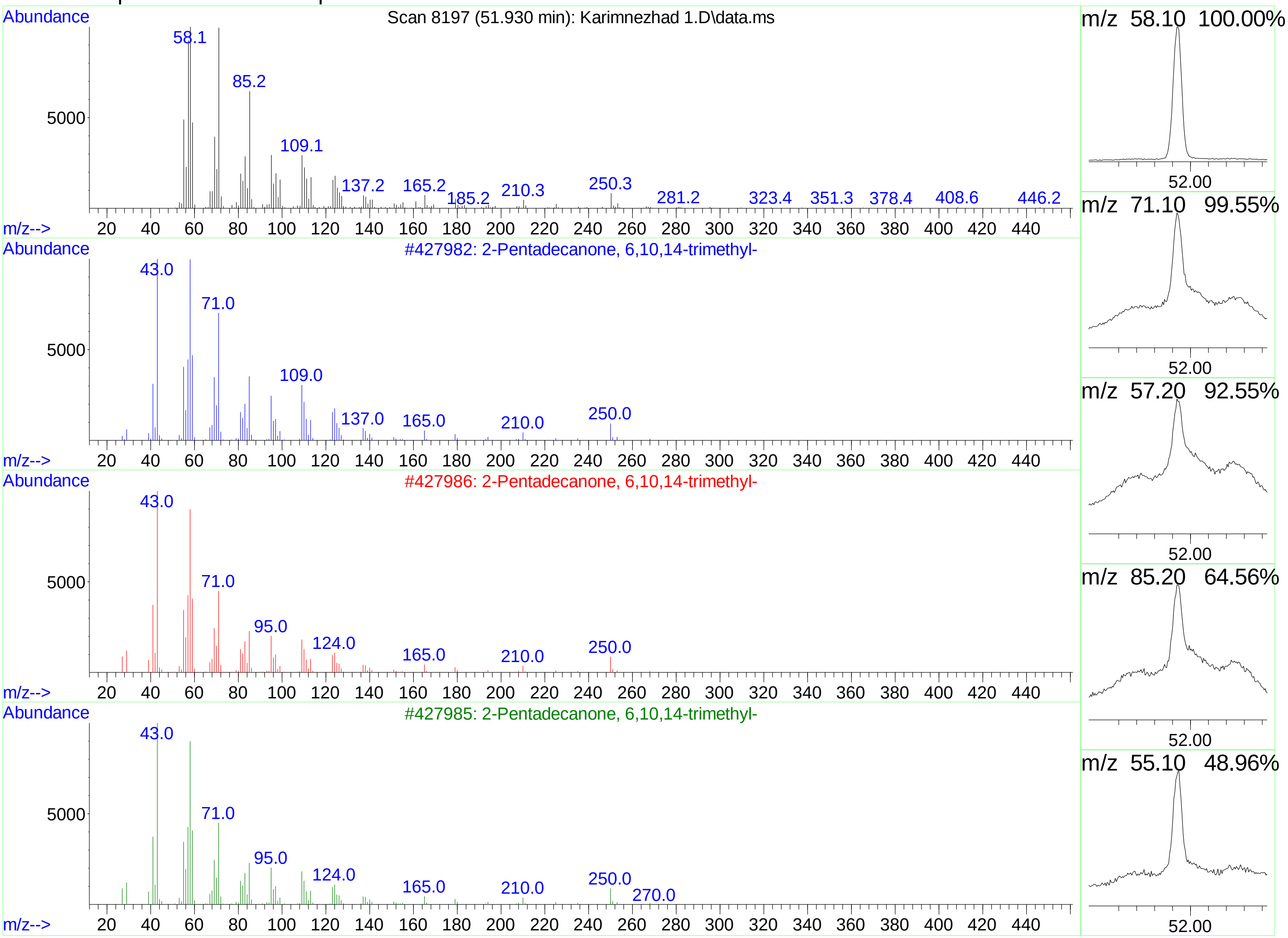

Data File: D:\msdchem\1\data\Karimnezhad 1.D
Sample : SDE

Peak Number: 51 at 51.930 min Area: 36951372 Area % 0.19

The 3 best hits from each library.	Ref#	CAS#	Qual
D:\Database\W10N14.L			
1 2-Pentadecanone, 6,10,14-trimethyl-	427982	000502-69-2	97
2 2-Pentadecanone, 6,10,14-trimethyl-	427986	000502-69-2	91
3 2-Pentadecanone, 6,10,14-trimethyl-	427985	000502-69-2	91

Unknown Spectrum based on Apex

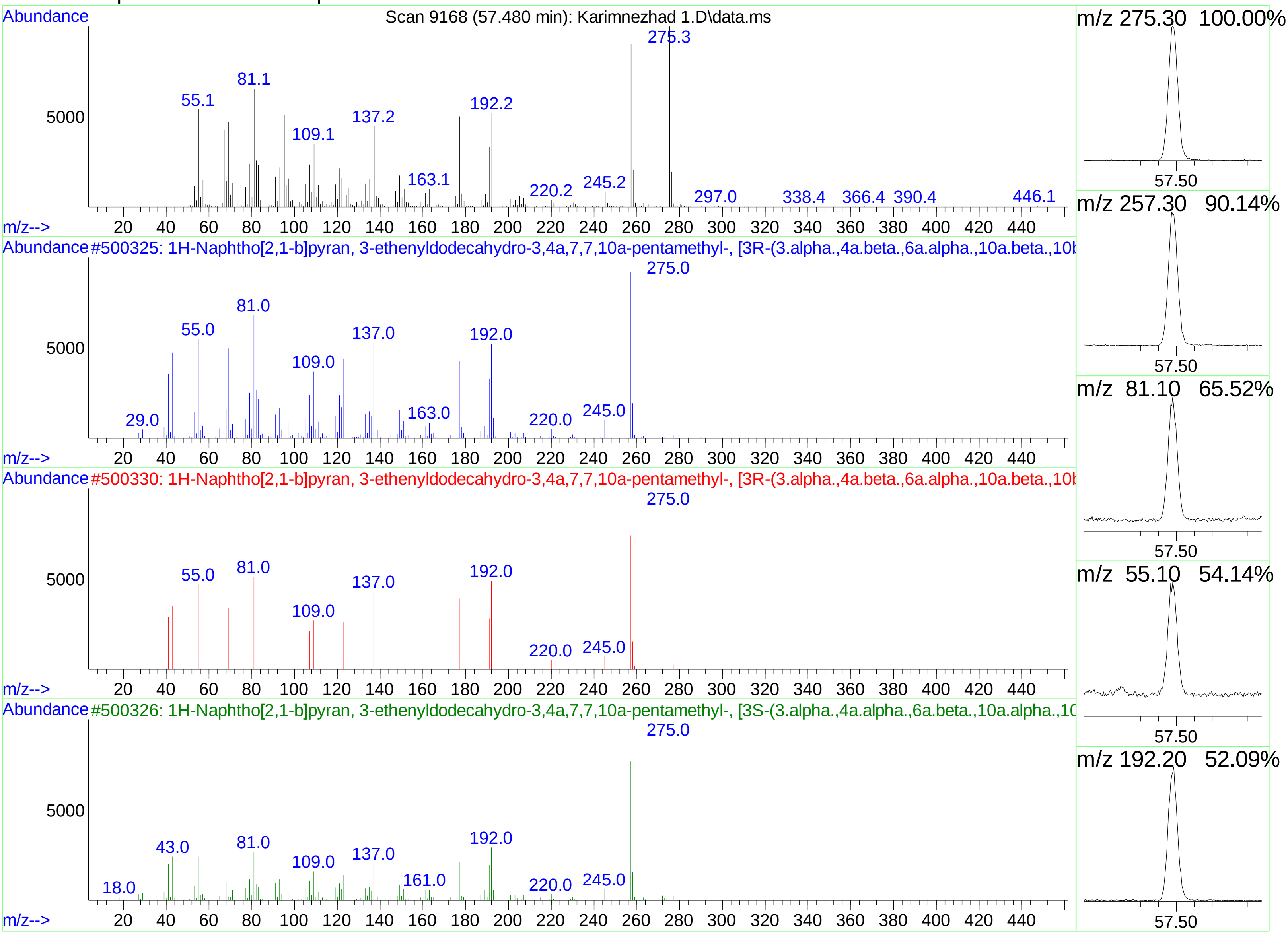

Data File: D:\msdchem\1\data\Karimnezhad 1.D
Sample : SDE

Peak Number: 52 at 57.480 min Area: 32334216 Area % 0.16

The 3 best hits from each library. Ref\# CAS\# Qual

D:\Database\W10N14.L

1	1H-Naphtho[2,1-b]pyran, 3-etheny...	500325	000596-84-9	99
2	1H-Naphtho[2,1-b]pyran, 3-etheny...	500330	000596-84-9	94
3	1H-Naphtho[2,1-b]pyran, 3-etheny...	500326	001227-93-6	94
