# Supplementary material for: Comparison of quantity, quality and antibacterial activity of essential oil Mentha longifolia (L.) L. under different traditional and modern extraction methods
Source: PLoS One. 2024 Jul 10;19(7):e0301558. doi: 10.1371/journal.pone.0301558 (PMC11236116; doi:10.1371/journal.pone.0301558)
Supplement: S2 File — (ZIP) [file pone.0301558.s002.zip › Karimnezhad/SDE/PrintText+summery.pdf]

Library Search Report

Data Path : D:\msdchem\1\data\
Data File : Karimnezhad 1.D
Acq On : 15 Mar 2022 6:21
Operator : Jafari
Sample : SDE
Misc :
ALS Vial : 29 Sample Multiplier: 1

Search Libraries: D:\Database\W10N14.L Minimum Quality: 0

Unknown Spectrum: Apex
Integration Events: ChemStation Integrator - events.e

Pk#	RT	Area%	Library/ID	Ref#	CAS#	Qual
1	11.551	0.12	D:\Database\W10N14.L			
			Furan, 2,5-diethyltetrahydro-	36992	041239-48-9	95
			Furan, 2,5-diethyltetrahydro-	36994	041239-48-9	78
			Furan, 2,5-diethyltetrahydro-	36993	041239-48-9	78
2	13.237	0.54	D:\Database\W10N14.L			
			(1R)-2,6,6-Trimethylbicyclo[3.1.1] hept-2-ene	49103	007785-70-8	96
			2-Pinene	49090	000080-56-8	96
			(1S)-2,6,6-Trimethylbicyclo[3.1.1] hept-2-ene	49099	007785-26-4	96
3	14.015	0.22	D:\Database\W10N14.L			
			2,2-dimethyl-3-methylene-bicyclo [2.2.1]heptane	49189	000079-92-5	97
			2,2-dimethyl-3-methylene-bicyclo [2.2.1]heptane	49207	000079-92-5	97
			2,2-dimethyl-3-methylene-bicyclo [2.2.1]heptane	49205	000079-92-5	97
4	14.981	0.30	D:\Database\W10N14.L			
			Bicyclo[3.1.0]hexane, 4-methylene- 1-(1-methylethyl)-	48855	003387-41-5	96
			4(10)-Thujene	48870	003387-41-5	96
			4(10)-Thujene	48857	003387-41-5	96
5	15.272	0.80	D:\Database\W10N14.L			
			4(10)-Thujene	48862	003387-41-5	94
			2(10)-Pinene	48535	000127-91-3	94
			2(10)-Pinene	48536	000127-91-3	94
6	15.609	0.14	D:\Database\W10N14.L			
			.beta.-Myrcene	48617	000123-35-3	96
			1,6-Octadiene, 7-methyl-3-methylen e-	48627	000123-35-3	95
			1,6-Octadiene, 7-methyl-3-methylen e-	48615	000123-35-3	94
7	15.992	0.31	D:\Database\W10N14.L			
			3-Octanol	40544	000589-98-0	90
			3-Octanol	40530	000589-98-0	83
			3-Octanol	40528	000589-98-0	83

Library Search Report

Data Path : D:\msdchem\1\data\
Data File : Karimnezhad 1.D
Acq On : 15 Mar 2022 6:21
Operator : Jafari
Sample : SDE
Misc :
ALS Vial : 29 Sample Multiplier: 1

Search Libraries: D:\Database\W10N14.L Minimum Quality: 0

Unknown Spectrum: Apex
Integration Events: ChemStation Integrator - events.e

Pk#	RT	Area%	Library/ID	Ref#	CAS#	Qual
8	17.038	0.21	D:\Database\W10N14.L			
			1,3-Cyclohexadiene, 1-methyl-4-(1-methylethyl)-	48406	000099-86-5	98
			1,3-Cyclohexadiene, 1-methyl-4-(1-methylethyl)-	48414	000099-86-5	98
			1,3-Cyclohexadiene, 1-methyl-4-(1-methylethyl)-	48422	000099-86-5	98
9	17.655	0.59	D:\Database\W10N14.L			
			D-Limonene	48457	005989-27-5	99
			Cyclohexene, 1-methyl-4-(1-methylethenyl)-	48442	000138-86-3	98
			Cyclohexene, 1-methyl-4-(1-methylethenyl)-	48478	000138-86-3	98
10	17.884	4.28	D:\Database\W10N14.L			
			2-Oxabicyclo[2.2.2]octane, 1,3,3-trimethyl-	83024	000470-82-6	98
			2-Oxabicyclo[2.2.2]octane, 1,3,3-trimethyl-	83030	000470-82-6	98
			2-Oxabicyclo[2.2.2]octane, 1,3,3-trimethyl-	83023	000470-82-6	97
11	19.004	0.22	D:\Database\W10N14.L			
			1,4-Cyclohexadiene, 1-methyl-4-(1-methylethyl)-	48401	000099-85-4	97
			1,4-Cyclohexadiene, 1-methyl-4-(1-methylethyl)-	48392	000099-85-4	96
			1,4-Cyclohexadiene, 1-methyl-4-(1-methylethyl)-	48379	000099-85-4	96
12	23.216	0.21	D:\Database\W10N14.L			
			Isopinocarveol	78365	006712-79-4	90
			Bicyclo[3.1.1]heptan-3-ol, 6,6-dimethyl-2-methylene-, [1S-(1.alpha., 3.alpha.,5.alpha.)]-	78361	000547-61-5	68
			2(10)-Pinen-3-ol, cis-	78370	006712-79-4	59
13	23.559	0.28	D:\Database\W10N14.L			
			2,3,4,5,6-Pentamethylphenol	104739	2000104-73-9	83
			1(2H)-Naphthalenone, 3,4,5,6,7,8-hexahydro-7-methyl-	104860	059177-21-8	83
			(1S,6R)-3,7,7-Trimethylbicyclo[4.1	104284	2000104-28-4	80

Data Path : D:\msdchem\1\data\
Data File : Karimnezhad 1.D
Acq On : 15 Mar 2022 6:21
Operator : Jafari
Sample : SDE
Misc :
ALS Vial : 29 Sample Multiplier: 1

Search Libraries: D:\Database\W10N14.L Minimum Quality: 0

Unknown Spectrum: Apex
Integration Events: ChemStation Integrator - events.e

Pk# RT Area% Library/ID Ref# CAS# Qual

.0]hept-3-en-2,5-dione

14 23.845 0.21 D:\Database\W10N14.L
Cyclohexanone, 5-methyl-2-(1-methy 83410 000089-80-5 98
lethyl)-, trans-
L-MENTHONE 83439 010458-14-7 98
2-ISOPROPYL-5-METHYLCYCLOHEXANONE 83413 000089-80-5 98

15 24.194 0.15 D:\Database\W10N14.L
2(10)-Pinen-3-one, (1S,5S)-(-)- 72864 019890-00-7 91
Pinocarvone 72865 030460-92-5 91
2-Pyridinamine, 3-methyl- 16090 001603-40-3 72

16 24.296 0.25 D:\Database\W10N14.L
L-MENTHONE 83439 010458-14-7 98
Cyclohexanone, 5-methyl-2-(1-methy 83392 000089-80-5 98
lethyl)-, trans-
l-Menthone 83425 014073-97-3 98

17 24.611 0.36 D:\Database\W10N14.L
Cyclohexanemethanol, .alpha.,.alph 83142 007299-42-5 90
a.-dimethyl-4-methylene-
3-Cyclohexene-1-methanol, .alpha., 82893 010482-56-1 64
.alpha.,4-trimethyl-
7-Octen-2-ol, 2-methyl-6-methylene 84020 000543-39-5 59

18 24.782 1.51 D:\Database\W10N14.L
1,7,7-TRIMETHYLBICYCLO[2.2.1]HEPTA 84051 000464-45-9 94
N-2-OL
endo-Borneol 84059 000507-70-0 93
Bicyclo[2.2.1]heptan-2-ol, 1,7,7-t 84061 000464-45-9 93
rimethyl-, (1S-endo)-

19 25.091 0.68 D:\Database\W10N14.L
1-ISOPROPYL-4-METHYL-3-CYCLOHEXEN- 82925 000562-74-3 98
1-OL
3-Cyclohexen-1-ol, 4-methyl-1-(1-m 82943 000562-74-3 98
ethylethyl)-
3-Cyclohexen-1-ol, 4-methyl-1-(1-m 82938 000562-74-3 97
ethylethyl)-

20 25.445 0.40 D:\Database\W10N14.L
PARA-CYMEN-8-OL 72711 001197-01-9 90

Data Path : D:\msdchem\1\data\
Data File : Karimnezhad 1.D
Acq On : 15 Mar 2022 6:21
Operator : Jafari
Sample : SDE
Misc :
ALS Vial : 29 Sample Multiplier: 1

Search Libraries: D:\Database\W10N14.L Minimum Quality: 0

Unknown Spectrum: Apex
Integration Events: ChemStation Integrator - events.e

Pk#	RT	Area%	Library/ID	Ref#	CAS#	Qual
			Benzenemethanol, .alpha.,.alpha.,4 -trimethyl-	72702	001197-01-9	87
			3-Methyl-6-hydroxybenzo[c]-dihydro furan	72145	2000072-14-5	83
21	25.805	0.52	D:\Database\W10N14.L			
			Cyclohexene, 1-methyl-3-(1-methylethenyl)-, (.+-.)-	48587	000499-03-6	89
			Cyclohexene, 5-methyl-3-(1-methylethenyl)-, trans-(-)-	48798	056816-08-1	60
			Cyclohexene, 5-methyl-3-(1-methylethenyl)-, trans-(-)-	48801	056816-08-1	60
22	26.863	1.56	D:\Database\W10N14.L			
			2-Hydroxy-2-isopropyl-5-methylcyclohexanone	115823	000000-00-0	80
			1,3-Cyclohexanedione, 5-isopropyl-	82473	018456-87-6	41
			3-Heptene, 2,2,3,5,6-pentamethyl-	116649	116164-06-8	30
23	27.166	2.29	D:\Database\W10N14.L			
			8,9-Dehydrothymol	68975	018612-99-2	95
			1-methoxy-4-(1-methylethenyl)benzene	68640	2000068-64-0	81
			Benzenepropanal, .beta.-methyl-	68867	016251-77-7	80
24	27.977	9.34	D:\Database\W10N14.L			
			(R)-5-methyl-2-(1-methylethylidene)-cyclohexanone	78102	000089-82-7	97
			Pulegone	78101	000089-82-7	97
			Cyclohexanone, 5-methyl-2-(1-methylethylidene)-	78096	015932-80-6	96
25	28.097	0.60	D:\Database\W10N14.L			
			2-Cyclohexen-1-one, 2-methyl-5-(1-methylethenyl)-, (R)-	72847	006485-40-1	96
			2-Cyclohexen-1-one, 2-methyl-5-(1-methylethenyl)-	72827	000099-49-0	95
			D-Carvone	72854	002244-16-8	95
26	28.400	0.59	D:\Database\W10N14.L			
			7-Oxabicyclo[4.1.0]heptan-2-one, 6-methyl-3-(1-methylethylidene)-	114758	035178-55-3	97
			4-ISOPROPENYL-1-METHYL-7-OXABICYCL	114757	035178-55-3	52

Data Path : D:\msdchem\1\data\
Data File : Karimnezhad 1.D
Acq On : 15 Mar 2022 6:21
Operator : Jafari
Sample : SDE
Misc :
ALS Vial : 29 Sample Multiplier: 1

Search Libraries: D:\Database\W10N14.L Minimum Quality: 0

Unknown Spectrum: Apex
Integration Events: ChemStation Integrator - events.e

Pk#	RT	Area%	Library/ID	Ref#	CAS#	Qual
			O[4.1.0]HEPTAN-2-ONE			
			3-Hexene, 2,2,5,5-tetramethyl-, (Z)-	55805	000692-47-7	46
27	28.549	1.27	D:\Database\W10N14.L			
			7-Oxabicyclo[4.1.0]heptan-2-one, 6 -methyl-3-(1-methylethyl)-	115503	005286-38-4	91
			4-ISOPROPENYL-1-METHYL-7-OXABICYCL O[4.1.0]HEPTAN-2-ONE	114757	035178-55-3	70
			Cyclohexane, 1,2,3-trimethyl-	34631	001678-97-3	60
28	28.766	0.28	D:\Database\W10N14.L			
			1,1'-Bicyclopentyl	52365	001636-39-1	55
			1,1'-Bicyclopentyl	52366	001636-39-1	46
			1,1'-Bicyclopentyl	52367	001636-39-1	46
29	28.874	0.21	D:\Database\W10N14.L			
			HERBOXIDE SECOND ISOMER	77558	013679-86-2	38
			9-Azabicyclo[3.3.1]nonan-3-one, 9- methyl-	80163	000552-70-5	30
			1,4-DIHYDROSILINE	8542	081200-77-3	30
30	29.023	0.20	D:\Database\W10N14.L			
			4-Fluoro-2-acetylphenol	81032	000394-32-1	80
			2,4-Dimethoxyphenol	81451	013330-65-9	80
			5-Fluoro-2-hydroxyacetophenone	81029	000394-32-1	74
31	29.303	1.52	D:\Database\W10N14.L			
			(S)-(+)-cis-Isopiperitenone	72898	2000072-89-8	91
			1,8-(p-MENTHADIENONE)	72899	2000072-89-9	72
			2-Cyclohexen-1-one, 3,5,5-trimethy l- (CAS)	51799	000078-59-1	50
32	29.680	0.25	D:\Database\W10N14.L			
			4-Hydroxy-3-methylacetophenone	72072	000876-02-8	90
			1(3aH)-Pentalenone, 4,5,6,6a-tetra hydro-2,6a-dimethyl-	72961	070640-02-7	90
			4-(Hydroxymethyl)acetophenone	71891	075633-63-5	90
33	29.886	0.53	D:\Database\W10N14.L			
			Bicyclo[2.2.1]heptan-2-ol, 1,7,7-t rimethyl-, acetate, (1S-endo)-	192108	005655-61-8	99
			Bicyclo[2.2.1]heptan-2-ol, 1,7,7-t	192123	005655-61-8	99

Data Path : D:\msdchem\1\data\
Data File : Karimnezhad 1.D
Acq On : 15 Mar 2022 6:21
Operator : Jafari
Sample : SDE
Misc :
ALS Vial : 29 Sample Multiplier: 1

Search Libraries: D:\Database\W10N14.L Minimum Quality: 0

Unknown Spectrum: Apex
Integration Events: ChemStation Integrator - events.e

Pk#	RT	Area%	Library/ID	Ref#	CAS#	Qual
			rimethyl-, acetate, (1S-endo)-			
			Bicyclo[2.2.1]heptan-2-ol, 1,7,7-trimethyl-, acetate, endo-	192102	000076-49-3	98
34	30.252	0.69	D:\Database\W10N14.L			
			Benzene, 1-ethoxy-4-ethyl-	73585	001585-06-4	94
			Benzene, 1-ethoxy-4-ethyl- (CAS)	73584	001585-06-4	94
			2,4-Cycloheptadien-1-one, 2,6,6-trimethyl-	73012	000503-93-5	86
35	31.029	0.31	D:\Database\W10N14.L			
			Phenol, 5-methyl-2-(1-methylethyl)	73197	000089-83-8	95
			Phenol, 5-methyl-2-(1-methylethyl)	73199	000089-83-8	95
			Thymol	73196	000089-83-8	95
36	31.503	0.35	D:\Database\W10N14.L			
			3-Methyl-4-isopropylphenol	73390	003228-02-2	95
			3-Methyl-4-isopropylphenol	73391	003228-02-2	94
			Carvacrol	72969	000499-75-2	94
37	33.018	38.06	D:\Database\W10N14.L			
			2-Cyclohexen-1-one, 3-methyl-6-(1-methylethylidene)-	73417	000491-09-8	98
			2-Cyclohexen-1-one, 3-methyl-6-(1-methylethylidene)-	73419	000491-09-8	95
			Car-3-en-2-one	72910	2000072-91-0	90
38	33.870	19.79	D:\Database\W10N14.L			
			PIPERITENONE OXIDE	110057	003564-96-3	99
			2-[3'-Hydroxypropyl]-1,4-benzoquinone	108313	2000108-31-3	86
			4-Acetyl-1-methylcyclohexene	51791	006090-09-1	60
39	34.230	0.26	D:\Database\W10N14.L			
			2',6'-Dihydroxy-3'-methylacetophenone	108340	029183-78-6	87
			Ethanone, 1-(2-hydroxy-6-methoxyphenyl)- (CAS)	108274	000703-23-1	80
			1,4-Dimethoxy-2,5-dimethylbenzene	109832	2000109-83-2	80
40	34.578	0.28	D:\Database\W10N14.L			
			.BETA. BOURBONENE	215702	005208-59-3	99
			(-)-.beta.-Bourbonene	215705	005208-59-3	96

Data Path : D:\msdchem\1\data\
Data File : Karimnezhad 1.D
Acq On : 15 Mar 2022 6:21
Operator : Jafari
Sample : SDE
Misc :
ALS Vial : 29 Sample Multiplier: 1

Search Libraries: D:\Database\W10N14.L Minimum Quality: 0

Unknown Spectrum: Apex
Integration Events: ChemStation Integrator - events.e

```
Pk#  RT   Area%      Library/ID             Ref#    CAS#   Qual
_______________________________________________________________
          Cyclobuta[1,2:3,4]dicyclopentene,  215701 005208-59-3 95
          1,2,3,3a,3b.beta.,4,5,6,6a.beta.,6
          b.alpha.-decahydro-1.alpha.-isopro
           pyl-3a.alpha.-methyl-6-methylene-

 41  35.138  1.88 D:\Database\W10N14.L
          4,6-DIETHYL-2-METHOXYPYRIMIDINE    110182 2000110-18-2 72
          2-hydroxy-7-methoxy-4-methylcycloh 108661 2000108-66-1 64
          epta-2,4,6-trien-1-one
          2-Hydroxy-7-methoxy-4-methylcycloh 108662 2000108-66-2 64
          epta-2,4,6-trien-1-one

 42  36.167  2.09 D:\Database\W10N14.L
          Caryophyllene                      216361 000087-44-5 99
          Bicyclo[7.2.0]undec-4-ene, 4,11,11 216351 000087-44-5 99
          -trimethyl-8-methylene-, (E)-(1R,9
          S)-(-)-
          TRANS(.BETA.)-CARYOPHYLLENE        216339 2000216-33-9 99

 43  37.162  0.30 D:\Database\W10N14.L
          trans-.beta.-Farnesene             216566 000502-60-3 96
          (E)-.beta.-Famesene                216558 018794-84-8 96
          (1S,5S,6R)-6-Methyl-2-methylene-6- 216863 015438-94-5 95
          (4-methylpent-3-en-1-yl)bicyclo[3.
          1.1]heptane

 44  37.642  0.31 D:\Database\W10N14.L
          .alpha.-Humulene                   216788 006753-98-6 99
          Humulene                           216796 006753-98-6 98
          .alpha.-Humulene                   216795 006753-98-6 98

 45  38.682  0.66 D:\Database\W10N14.L
          1H-Cyclopenta[1,3]cyclopropa[1,2]b 216768 013744-15-5 99
          enzene, 2,3,3a.alpha.,3b.alpha.,4,
          5,6,7-octahydro-4.alpha.-isopropyl
          -7.beta.-methyl-3-methylene-
          8-ISOPROPYL-1-METHYL-5-METHYLENE-1 216746 023986-74-5 98
          ,6-CYCLODECADIENE
          1,6-Cyclodecadiene, 1-methyl-5-met 216750 023986-74-5 98
          hylene-8-(1-methylethyl)-, [s-(E,E
          )]-

 46  42.100  0.68 D:\Database\W10N14.L
```

Data Path : D:\msdchem\1\data\
Data File : Karimnezhad 1.D
Acq On : 15 Mar 2022 6:21
Operator : Jafari
Sample : SDE
Misc :
ALS Vial : 29 Sample Multiplier: 1

Search Libraries: D:\Database\W10N14.L Minimum Quality: 0

Unknown Spectrum: Apex
Integration Events: ChemStation Integrator - events.e

Pk#	RT	Area%	Library/ID	Ref#	CAS#	Qual
			Cyclohexanecarboxylic acid, 1-methyl-2-oxo-, ethyl ester	156877	005453-94-1	83
			3-Chloro-4-t-butyl-6-methylpyridazine	155956	2000155-95-6	83
			5(6)-(Chloromethyl)benzo[1,2-c]-1,2,5-oxadiazole N1-Oxide	154801	2000154-80-1	59
47	42.631	0.59	D:\Database\W10N14.L			
			1,1,7-TRIMETHYL-4-METHYLENEDECAHYDRO-1H-CYCLOPROPA[E]AZULEN-7-OL	267795	077171-55-2	98
			1H-Cycloprop[e]azulen-7-ol, decahydro-1,1,7-trimethyl-4-methylene-, [1ar-(1a.alpha.,4a.alpha.,7.beta., 7a.beta.,7b.alpha.)]-	267797	006750-60-3	96
			1H-Cycloprop[e]azulen-7-ol, decahydro-1,1,7-trimethyl-4-methylene-, [1ar-(1a.alpha.,4a.alpha.,7.beta., 7a.beta.,7b.alpha.)]-	267799	006750-60-3	93
48	42.877	1.97	D:\Database\W10N14.L			
			(-)-5-Oxatricyclo[8.2.0.0(4,6)]dodecane,,12-trimethyl-9-methylene-, [1R-(1R*,4R*,6R*,10S*)]-	267388	001139-30-6	99
			Caryophyllene oxide	267391	001139-30-6	99
			(-)-5-Oxatricyclo[8.2.0.0(4,6)]dodecane,,12-trimethyl-9-methylene-, [1R-(1R*,4R*,6R*,10S*)]-	267387	001139-30-6	95
49	43.917	0.21	D:\Database\W10N14.L			
			(1R,3E,7E,11R)-1,5,5,8-Tetramethyl-12-oxabicyclo[9.1.0]dodeca-3,7-diene	267258	019888-34-7	99
			Naphthalene, decahydro-, cis-	52317	000493-01-6	78
			(1R,3E,7E,11R)-1,5,5,8-Tetramethyl-12-oxabicyclo[9.1.0]dodeca-3,7-diene	267257	019888-34-7	74
50	46.158	0.25	D:\Database\W10N14.L			
			Bicyclo[7.2.0]undec-3-en-5-ol, 4,11,11-trimethyl-8-methylene-, stereoisomer	267841	032214-89-4	96
			Presilphiperfolane-9,15-epoxide	267660	2000267-66-0	87
			(-)-5-Oxatricyclo[8.2.0.0(4,6)]dod	267389	001139-30-6	83

Data Path : D:\msdchem\1\data\
Data File : Karimnezhad 1.D
Acq On : 15 Mar 2022 6:21
Operator : Jafari
Sample : SDE
Misc :
ALS Vial : 29 Sample Multiplier: 1

Search Libraries: D:\Database\W10N14.L Minimum Quality: 0

Unknown Spectrum: Apex
Integration Events: ChemStation Integrator - events.e

Pk#	RT	Area%	Library/ID	Ref#	CAS#	Qual
			ecane,,12-trimethyl-9-methylene-, [1R-(1R*,4R*,6R*,10S*)]-			
51	51.930	0.19	D:\Database\W10N14.L			
			2-Pentadecanone, 6,10,14-trimethyl	427982	000502-69-2	97
			2-Pentadecanone, 6,10,14-trimethyl	427986	000502-69-2	91
			2-Pentadecanone, 6,10,14-trimethyl	427985	000502-69-2	91
52	57.480	0.16	D:\Database\W10N14.L			
			1H-Naphtho[2,1-b]pyran, 3-ethenyld odecahydro-3,4a,7,7,10a-pentamethy l-, [3R-(3.alpha.,4a.beta.,6a.alph a.,10a.beta.,10b.alpha.)]-	500325	000596-84-9	99
			1H-Naphtho[2,1-b]pyran, 3-ethenyld odecahydro-3,4a,7,7,10a-pentamethy l-, [3R-(3.alpha.,4a.beta.,6a.alph a.,10a.beta.,10b.alpha.)]-	500330	000596-84-9	94
			1H-Naphtho[2,1-b]pyran, 3-ethenyld odecahydro-3,4a,7,7,10a-pentamethy l-, [3S-(3.alpha.,4a.alpha.,6a.bet a.,10a.alpha.,10b.beta.)]- (CAS)	500326	001227-93-6	94
