# Supplementary material for: Comparison of quantity, quality and antibacterial activity of essential oil Mentha longifolia (L.) L. under different traditional and modern extraction methods
Source: PLoS One. 2024 Jul 10;19(7):e0301558. doi: 10.1371/journal.pone.0301558 (PMC11236116; doi:10.1371/journal.pone.0301558)
Supplement: S2 File — (ZIP) [file pone.0301558.s002.zip › Karimnezhad/M10/PrintText+summery.pdf]

Library Search Report

Data Path : D:\msdchem\1\data\
Data File : Karimnezhad 2.D
Acq On : 15 Mar 2022 7:36
Operator : Jafari
Sample : M10
Misc :
ALS Vial : 30 Sample Multiplier: 1

Search Libraries: D:\Database\W10N14.L Minimum Quality: 0

Unknown Spectrum: Apex
Integration Events: ChemStation Integrator - events.e

Pk#	RT	Area%	Library/ID	Ref#	CAS#	Qual
1	11.551	0.13	D:\Database\W10N14.L			
			Furan, 2,5-diethyltetrahydro-	36996	041239-48-9	64
			Furan, 2,5-diethyltetrahydro-	36992	041239-48-9	53
			Furan, 2,5-diethyltetrahydro-	36993	041239-48-9	49
2	13.237	1.16	D:\Database\W10N14.L			
			(1R)-2,6,6-Trimethylbicyclo[3.1.1] hept-2-ene	49103	007785-70-8	96
			2-Pinene	49090	000080-56-8	96
			(1S)-2,6,6-Trimethylbicyclo[3.1.1] hept-2-ene	49099	007785-26-4	96
3	14.009	0.35	D:\Database\W10N14.L			
			2,2-dimethyl-3-methylene-bicyclo [2.2.1]heptane	49202	000079-92-5	97
			2,2-dimethyl-3-methylene-bicyclo [2.2.1]heptane	49190	000079-92-5	97
			2,2-dimethyl-3-methylene-bicyclo [2.2.1]heptane	49189	000079-92-5	97
4	14.981	0.67	D:\Database\W10N14.L			
			4(10)-Thujene	48867	003387-41-5	97
			Bicyclo[3.1.0]hexane, 4-methylene- 1-(1-methylethyl)-	48855	003387-41-5	96
			4(10)-Thujene	48870	003387-41-5	96
5	15.272	1.39	D:\Database\W10N14.L			
			4(10)-Thujene	48862	003387-41-5	94
			2(10)-Pinene	48535	000127-91-3	94
			2(10)-Pinene	48531	000127-91-3	94
6	15.609	0.36	D:\Database\W10N14.L			
			.beta.-Myrcene	48617	000123-35-3	96
			1,6-Octadiene, 7-methyl-3-methylen e-	48627	000123-35-3	95
			1,6-Octadiene, 7-methyl-3-methylen e-	48620	000123-35-3	94
7	15.986	0.38	D:\Database\W10N14.L			
			3-Octanol	40544	000589-98-0	90
			3-Octanol	40527	000589-98-0	83
			3-Octanol	40530	000589-98-0	83

Library Search Report

Data Path : D:\msdchem\1\data\
Data File : Karimnezhad 2.D
Acq On : 15 Mar 2022 7:36
Operator : Jafari
Sample : M10
Misc :
ALS Vial : 30 Sample Multiplier: 1

Search Libraries: D:\Database\W10N14.L Minimum Quality: 0

Unknown Spectrum: Apex
Integration Events: ChemStation Integrator - events.e

Pk#	RT	Area%	Library/ID	Ref#	CAS#	Qual
8	17.038	0.18	D:\Database\W10N14.L			
			1,3-Cyclohexadiene, 1-methyl-4-(1-methylethyl)-	48406	000099-86-5	98
			1,3-Cyclohexadiene, 1-methyl-4-(1-methylethyl)-	48418	000099-86-5	97
			1,3-Cyclohexadiene, 1-methyl-4-(1-methylethyl)-	48414	000099-86-5	97
9	17.415	0.13	D:\Database\W10N14.L			
			o-Cymene	45121	000527-84-4	97
			p-Cymene	45379	000099-87-6	97
			Benzene, 1-methyl-2-(1-methylethyl)-	45118	000527-84-4	97
10	17.655	1.44	D:\Database\W10N14.L			
			D-Limonene	48457	005989-27-5	99
			Cyclohexene, 1-methyl-4-(1-methylethenyl)-	48442	000138-86-3	98
			Cyclohexene, 1-methyl-4-(1-methylethenyl)-	48478	000138-86-3	98
11	17.867	5.35	D:\Database\W10N14.L			
			2-Oxabicyclo[2.2.2]octane, 1,3,3-trimethyl-	83024	000470-82-6	98
			2-Oxabicyclo[2.2.2]octane, 1,3,3-trimethyl-	83030	000470-82-6	98
			2-Oxabicyclo[2.2.2]octane, 1,3,3-trimethyl-	83023	000470-82-6	96
12	19.004	0.31	D:\Database\W10N14.L			
			.gamma.-Terpinene	48386	000099-85-4	97
			1,4-Cyclohexadiene, 1-methyl-4-(1-methylethyl)-	48401	000099-85-4	97
			1,4-Cyclohexadiene, 1-methyl-4-(1-methylethyl)-	48376	000099-85-4	96
13	20.873	0.27	D:\Database\W10N14.L			
			ISOAMYL-2-METHYL BUTYRATE	126282	027625-35-0	86
			Butanoic acid, 2-methyl-, 3-methylbutyl ester	126283	027625-35-0	86
			Butanoic acid, 2-methyl-, 3-methylbutyl ester	126288	027625-35-0	86

Data Path : D:\msdchem\1\data\
Data File : Karimnezhad 2.D
Acq On : 15 Mar 2022 7:36
Operator : Jafari
Sample : M10
Misc :
ALS Vial : 30 Sample Multiplier: 1

Search Libraries: D:\Database\W10N14.L Minimum Quality: 0

Unknown Spectrum: Apex
Integration Events: ChemStation Integrator - events.e

Pk#	RT	Area%	Library/ID	Ref#	CAS#	Qual
14	21.176	0.17	D:\Database\W10N14.L			
			2H-Pyran-2-one, 6-ethyltetrahydro-	36615	003301-90-4	43
			2H-Pyran-2-one, 6-ethyltetrahydro-	36617	003301-90-4	43
			Cyclohexanol, 1-ethyl-	37501	001940-18-7	38
15	21.776	0.12	D:\Database\W10N14.L			
			3-Octanol, acetate	126339	004864-61-3	91
			3-Octanol, acetate	126344	004864-61-3	50
			3-OCTANYL ACETATE	126340	004864-61-3	43
16	22.719	0.25	D:\Database\W10N14.L			
			2-Methyl-7-oxabicyclo[3.3.0]oct-1-en-3-one	50887	2000050-88-7	86
			3-Methyl-4-methylenecycloheptanone	51463	2000051-46-3	83
			4-Methyl-3-isopropenylthiophene	50627	2000050-62-7	83
17	23.211	0.19	D:\Database\W10N14.L			
			Isopinocarveol	78365	006712-79-4	94
			Bicyclo[3.1.1]heptan-3-ol, 6,6-dimethyl-2-methylene-, [1S-(1.alpha.,3.alpha.,5.alpha.)]-	78364	000547-61-5	87
			Bicyclo[3.1.1]heptan-3-ol, 6,6-dimethyl-2-methylene-, [1S-(1.alpha.,3.alpha.,5.alpha.)]-	78361	000547-61-5	83
18	23.554	0.27	D:\Database\W10N14.L			
			2,3,4,5,6-Pentamethylphenol	104739	2000104-73-9	83
			1-(4-Ethyl-2-hydroxyphenyl)ethanon	103396	2000103-39-6	83
			1(2H)-Naphthalenone, 3,4,5,6,7,8-hexahydro-7-methyl-	104860	059177-21-8	83
19	23.839	0.23	D:\Database\W10N14.L			
			Cyclohexanone, 5-methyl-2-(1-methylethyl)-, trans-	83410	000089-80-5	98
			l-Menthone	83419	014073-97-3	97
			l-Menthone	83425	014073-97-3	97
20	24.291	0.41	D:\Database\W10N14.L			
			L-MENTHONE	83439	010458-14-7	98
			Cyclohexanone, 5-methyl-2-(1-methylethyl)-, trans-	83392	000089-80-5	98
			Cyclohexanone, 5-methyl-2-(1-methylethyl)-, trans-	83410	000089-80-5	98

Data Path : D:\msdchem\1\data\
Data File : Karimnezhad 2.D
Acq On : 15 Mar 2022 7:36
Operator : Jafari
Sample : M10
Misc :
ALS Vial : 30 Sample Multiplier: 1

Search Libraries: D:\Database\W10N14.L Minimum Quality: 0

Unknown Spectrum: Apex
Integration Events: ChemStation Integrator - events.e

Pk#	RT	Area%	Library/ID	Ref#	CAS#	Qual
21	24.588	0.34	D:\Database\W10N14.L			
			Cyclohexanemethanol, .alpha.,.alpha.-dimethyl-4-methylene-	83142	007299-42-5	86
			.alpha.-Terpineol	82900	000098-55-5	64
			3-Cyclohexene-1-methanol, .alpha.,.alpha.,4-trimethyl-	82893	010482-56-1	59
22	24.759	1.44	D:\Database\W10N14.L			
			1,7,7-TRIMETHYLBICYCLO[2.2.1]HEPTAN-2-OL	84051	000464-45-9	94
			endo-Borneol	84059	000507-70-0	94
			Bicyclo[2.2.1]heptan-2-ol, 1,7,7-trimethyl-, (1S-endo)-	84056	000464-45-9	90
23	25.079	0.61	D:\Database\W10N14.L			
			1-ISOPROPYL-4-METHYL-3-CYCLOHEXEN-1-OL	82925	000562-74-3	98
			3-Cyclohexen-1-ol, 4-methyl-1-(1-methylethyl)-	82943	000562-74-3	98
			3-Cyclohexen-1-ol, 4-methyl-1-(1-methylethyl)-	82938	000562-74-3	97
24	25.400	0.39	D:\Database\W10N14.L			
			PARA-CYMEN-8-OL	72711	001197-01-9	94
			Benzenemethanol, .alpha.,.alpha.,4-trimethyl-	72702	001197-01-9	91
			Silane, trimethylphenyl-	72516	000768-32-1	80
25	25.782	0.52	D:\Database\W10N14.L			
			Cyclohexene, 1-methyl-3-(1-methylethenyl)-, (.+-.)-	48587	000499-03-6	89
			Cycloheptene, 5-ethylidene-1-methyl-	49058	015402-94-5	60
			Cyclohexene, 5-methyl-3-(1-methylethenyl)-, trans-(-)-	48798	056816-08-1	60
26	26.977	3.35	D:\Database\W10N14.L			
			8,9-Dehydrothymol	68975	018612-99-2	95
			1-methoxy-4-(1-methylethenyl)benzene	68640	2000068-64-0	81
			Benzenepropanal, .beta.-methyl-	68867	016251-77-7	80

Data Path : D:\msdchem\1\data\
Data File : Karimnezhad 2.D
Acq On : 15 Mar 2022 7:36
Operator : Jafari
Sample : M10
Misc :
ALS Vial : 30 Sample Multiplier: 1

Search Libraries: D:\Database\W10N14.L Minimum Quality: 0

Unknown Spectrum: Apex
Integration Events: ChemStation Integrator - events.e

Pk#	RT	Area%	Library/ID	Ref#	CAS#	Qual
27	27.920	10.47	D:\Database\W10N14.L			
			Cyclohexanone, 5-methyl-2-(1-methylethylidene)-	78096	015932-80-6	96
			(R)-5-methyl-2-(1-methylethylidene)-cyclohexanone	78102	000089-82-7	96
			(R)-5-methyl-2-(1-methylethylidene)-cyclohexanone	78091	000089-82-7	95
28	28.063	0.65	D:\Database\W10N14.L			
			D-Carvone	72855	002244-16-8	97
			2-Cyclohexen-1-one, 2-methyl-5-(1-methylethenyl)-, (S)-	72823	002244-16-8	97
			2-Cyclohexen-1-one, 2-methyl-5-(1-methylethenyl)-	72837	000099-49-0	97
29	28.372	0.53	D:\Database\W10N14.L			
			7-Oxabicyclo[4.1.0]heptan-2-one, 6-methyl-3-(1-methylethylidene)-	114758	035178-55-3	97
			4-ISOPROPENYL-1-METHYL-7-OXABICYCLO[4.1.0]HEPTAN-2-ONE	114757	035178-55-3	52
			3-Hexene, 2,2,5,5-tetramethyl-, (Z)-	55805	000692-47-7	46
30	28.514	1.17	D:\Database\W10N14.L			
			4-ISOPROPENYL-1-METHYL-7-OXABICYCLO[4.1.0]HEPTAN-2-ONE	114757	035178-55-3	93
			7-Oxabicyclo[4.1.0]heptan-2-one, 6-methyl-3-(1-methylethyl)-	115503	005286-38-4	87
			Cyclohexane, 1,2,3-trimethyl-	34631	001678-97-3	60
31	28.737	0.20	D:\Database\W10N14.L			
			1,1'-Bicyclopentyl	52365	001636-39-1	50
			2(1H)-Pyridinone, 1-isopropyl-	49895	022973-00-8	49
			3a,4,5,6,7,7a-hexahydro-4,7-methanobenz[d]isoxazole	49858	015166-80-0	46
32	29.000	0.18	D:\Database\W10N14.L			
			4-Fluoro-2-acetylphenol	81032	000394-32-1	80
			1-ethyl-3-methyl-2-propylidenimidazolidine	84359	109153-29-9	78
			5-Fluoro-2-hydroxyacetophenone	81029	000394-32-1	68
33	29.257	1.30	D:\Database\W10N14.L			

Data Path : D:\msdchem\1\data\
Data File : Karimnezhad 2.D
Acq On : 15 Mar 2022 7:36
Operator : Jafari
Sample : M10
Misc :
ALS Vial : 30 Sample Multiplier: 1

Search Libraries: D:\Database\W10N14.L Minimum Quality: 0

Unknown Spectrum: Apex
Integration Events: ChemStation Integrator - events.e

Pk#	RT	Area%	Library/ID	Ref#	CAS#	Qual
			(S)-(+)-cis-Isopiperitenone	72898	2000072-89-8	91
			1,8-(p-MENTHADIENONE)	72899	2000072-89-9	72
			2-Cyclohexen-1-one, 3,5,5-trimethyl-	51811	000078-59-1	50
34	29.663	0.19	D:\Database\W10N14.L			
			Ethanone, 1-(2-hydroxy-5-methylphenyl)-	71787	001450-72-2	90
			4-Methyl-1-(acetoxy)benzene	72241	000140-39-6	90
			4-(Methoxymethyl)benzenaldehyde	72078	2000072-07-8	90
35	29.875	0.50	D:\Database\W10N14.L			
			Bicyclo[2.2.1]heptan-2-ol, 1,7,7-trimethyl-, acetate, (1S-endo)-	192108	005655-61-8	99
			Bicyclo[2.2.1]heptan-2-ol, 1,7,7-trimethyl-, acetate, (1S-endo)-	192123	005655-61-8	99
			Acetic acid, 1,7,7-trimethyl-bicyclo[2.2.1]hept-2-yl ester	192116	092618-89-8	98
36	30.240	0.47	D:\Database\W10N14.L			
			(1S,6R)-3,7,7-Trimethylbicyclo[4.1.0]hept-3-en-5-one	72779	2000072-77-9	86
			Benzene, 1-ethoxy-4-ethyl-	73585	001585-06-4	83
			Benzene, 1-ethoxy-4-ethyl- (CAS)	73584	001585-06-4	83
37	30.732	0.40	D:\Database\W10N14.L			
			Phenol, 5-methyl-2-(1-methylethyl)	73200	000089-83-8	95
			Phenol, 5-methyl-2-(1-methylethyl)	73204	000089-83-8	95
			Phenol, 5-methyl-2-(1-methylethyl)	73199	000089-83-8	95
38	31.183	0.17	D:\Database\W10N14.L			
			3-Methyl-4-isopropylphenol	73390	003228-02-2	94
			Phenol, 2-methyl-5-(1-methylethyl)	72967	000499-75-2	94
			3-Methyl-4-isopropylphenol	73391	003228-02-2	94
39	31.578	0.44	D:\Database\W10N14.L			
			5-Hepten-3-yn-2-ol, 6-methyl-5-(1-methylethyl)-	110760	063922-41-8	87
			5-Hepten-3-yn-2-ol, 6-methyl-5-(1-methylethyl)-	110759	063922-41-8	87
			Ethanone, 1-(2-hydroxy-5-methoxyphenyl)-	108437	000705-15-7	81

Data Path : D:\msdchem\1\data\
Data File : Karimnezhad 2.D
Acq On : 15 Mar 2022 7:36
Operator : Jafari
Sample : M10
Misc :
ALS Vial : 30 Sample Multiplier: 1

Search Libraries: D:\Database\W10N14.L Minimum Quality: 0

Unknown Spectrum: Apex
Integration Events: ChemStation Integrator - events.e

Pk#	RT	Area%	Library/ID	Ref#	CAS#	Qual
40	32.829	37.77	D:\Database\W10N14.L			
			2-Cyclohexen-1-one, 3-methyl-6-(1-methylethylidene)-	73417	000491-09-8	98
			2-Cyclohexen-1-one, 3-methyl-6-(1-methylethylidene)-	73419	000491-09-8	97
			4,7,7-Trimethylbicyclo[4.1.0]hept-3-en-2-one	72780	081800-50-2	93
41	33.641	14.24	D:\Database\W10N14.L			
			PIPERITENONE OXIDE	110057	003564-96-3	98
			4-Acetyl-1-methylcyclohexene	51791	006090-09-1	60
			2,4-Heptadienal, 2-methyl-6-oxo-, (E,E)-	51105	129454-99-5	58
42	34.521	0.26	D:\Database\W10N14.L			
			(-)-.beta.-Bourbonene	215705	005208-59-3	95
			(-)-.beta.-Bourbonene	215707	005208-59-3	93
			.BETA. BOURBONENE	215702	005208-59-3	93
43	35.081	3.27	D:\Database\W10N14.L			
			4,6-DIETHYL-2-METHOXYPYRIMIDINE	110182	2000110-18-2	72
			2-hydroxy-7-methoxy-4-methylcyclohepta-2,4,6-trien-1-one	108661	2000108-66-1	64
			3-Hydroxy-4-methoxy-5-methylbenzaldehyde	108182	2000108-18-2	64
44	36.121	2.03	D:\Database\W10N14.L			
			Caryophyllene	216361	000087-44-5	99
			Bicyclo[7.2.0]undec-4-ene, 4,11,11-trimethyl-8-methylene-, (E)-(1R,9S)-(-)-	216351	000087-44-5	99
			TRANS(.BETA.)-CARYOPHYLLENE	216339	2000216-33-9	99
45	37.145	0.27	D:\Database\W10N14.L			
			trans-.beta.-Farnesene	216566	000502-60-3	96
			(E)-.beta.-Famesene	216558	018794-84-8	96
			(1S,5S,6R)-6-Methyl-2-methylene-6-(4-methylpent-3-en-1-yl)bicyclo[3.1.1]heptane	216863	015438-94-5	96
46	37.619	0.27	D:\Database\W10N14.L			
			.alpha.-Humulene	216788	006753-98-6	99
			.alpha.-Humulene	216803	006753-98-6	98

Data Path : D:\msdchem\1\data\
Data File : Karimnezhad 2.D
Acq On : 15 Mar 2022 7:36
Operator : Jafari
Sample : M10
Misc :
ALS Vial : 30 Sample Multiplier: 1

Search Libraries: D:\Database\W10N14.L Minimum Quality: 0

Unknown Spectrum: Apex
Integration Events: ChemStation Integrator - events.e

```
Pk#     RT  Area%        Library/ID                 Ref#     CAS#   Qual
_______________________________________________________________________________
               .alpha.-Humulene                  216789 006753-98-6 98

 47  38.653  0.56 D:\Database\W10N14.L
               1H-Cyclopenta[1,3]cyclopropa[1,2]b 216768 013744-15-5 99
               enzene, 2,3,3a.alpha.,3b.alpha.,4,
               5,6,7-octahydro-4.alpha.-isopropyl
               -7.beta.-methyl-3-methylene-
               Germacrene D                      216742 023986-74-5 99
               8-ISOPROPYL-1-METHYL-5-METHYLENE-1 216746 023986-74-5 98
               ,6-CYCLODECADIENE

 48  42.077  0.64 D:\Database\W10N14.L
               Cyclohexanecarboxylic acid, 1-meth 156877 005453-94-1 83
               yl-2-oxo-, ethyl ester
               3-Chloro-4-t-butyl-6-methylpyridaz 155956 2000155-95-6 83
               ine
               2-Methylthiocyclohexa[c]thiophene  155216 000000-00-0 64

 49  42.608  0.61 D:\Database\W10N14.L
               1H-Cycloprop[e]azulen-7-ol, decahy 267797 006750-60-3 99
               dro-1,1,7-trimethyl-4-methylene-,
               [1ar-(1a.alpha.,4a.alpha.,7.beta.,
               7a.beta.,7b.alpha.)]-
               1,1,7-TRIMETHYL-4-METHYLENEDECAHYD 267795 077171-55-2 98
               RO-1H-CYCLOPROPA[E]AZULEN-7-OL
               1,1,7-TRIMETHYL-4-METHYLENEDECAHYD 267794 006750-60-3 93
               RO-1H-CYCLOPROPA[E]AZULEN-7-OL

 50  42.848  2.17 D:\Database\W10N14.L
               (-)-5-Oxatricyclo[8.2.0.0(4,6)]dod 267388 001139-30-6 99
               ecane,,12-trimethyl-9-methylene-,
               [1R-(1R*,4R*,6R*,10S*)]-
               (-)-5-Oxatricyclo[8.2.0.0(4,6)]dod 267387 001139-30-6 95
               ecane,,12-trimethyl-9-methylene-,
               [1R-(1R*,4R*,6R*,10S*)]-
               Caryophyllene oxide               267393 001139-30-6 94

 51  43.906  0.21 D:\Database\W10N14.L
               (1R,3E,7E,11R)-1,5,5,8-Tetramethyl 267258 019888-34-7 99
               -12-oxabicyclo[9.1.0]dodeca-3,7-di
               ene
               (1R,3E,7E,11R)-1,5,5,8-Tetramethyl 267257 019888-34-7 87
               -12-oxabicyclo[9.1.0]dodeca-3,7-di
```

Data Path : D:\msdchem\1\data\
Data File : Karimnezhad 2.D
Acq On : 15 Mar 2022 7:36
Operator : Jafari
Sample : M10
Misc :
ALS Vial : 30 Sample Multiplier: 1

Search Libraries: D:\Database\W10N14.L Minimum Quality: 0

Unknown Spectrum: Apex
Integration Events: ChemStation Integrator - events.e

Pk#	RT	Area%	Library/ID	Ref#	CAS#	Qual
			ene			
			o-Menth-8-ene	52484	015193-25-6	55
52	44.946	0.25	D:\Database\W10N14.L			
			10,10-Dimethyl-2,6-dimethylenebicy clo[7.2.0]undecan-5.beta.-ol	267895	019431-80-2	98
			caryophylla-4(12),8(13)-dien-5.bet a.-ol	267136	2000267-13-6	95
			10,10-Dimethyl-2,6-dimethylenebicy clo[7.2.0]undecan-5.beta.-ol	267897	019431-80-2	95
53	46.158	0.26	D:\Database\W10N14.L			
			Presilphiperfolane-9,15-epoxide	267660	2000267-66-0	87
			2-(trans-2-Methylcyclohexyl)buta-1 ,3-diene	74040	2000074-04-0	70
			(-)-5-Oxatricyclo[8.2.0.0(4,6)]dod ecane,,12-trimethyl-9-methylene-, [1R-(1R*,4R*,6R*,10S*)]-	267389	001139-30-6	70
54	57.480	0.15	D:\Database\W10N14.L			
			1H-Naphtho[2,1-b]pyran, 3-ethenyld odecahydro-3,4a,7,7,10a-pentamethy l-, [3R-(3.alpha.,4a.beta.,6a.alph a.,10a.beta.,10b.alpha.)]-	500325	000596-84-9	99
			1H-Naphtho[2,1-b]pyran, 3-ethenyld odecahydro-3,4a,7,7,10a-pentamethy l-, [3R-(3.alpha.,4a.beta.,6a.alph a.,10a.beta.,10b.alpha.)]-	500330	000596-84-9	95
			1H-Naphtho[2,1-b]pyran, 3-ethenyld odecahydro-3,4a,7,7,10a-pentamethy l-, [3R-(3.alpha.,4a.beta.,6a.alph a.,10a.beta.,10b.alpha.)]-	500323	000596-84-9	94
