# Supplementary material for: Comparison of quantity, quality and antibacterial activity of essential oil Mentha longifolia (L.) L. under different traditional and modern extraction methods
Source: PLoS One. 2024 Jul 10;19(7):e0301558. doi: 10.1371/journal.pone.0301558 (PMC11236116; doi:10.1371/journal.pone.0301558)
Supplement: S2 File — (ZIP) [file pone.0301558.s002.zip › Karimnezhad/M10/QualKarimnezhad 2.pdf]

Library Search Report

Data Path : D:\msdchem\1\data\
Data File : Karimnezhad 2.D
Acq On : 15 Mar 2022 7:36
Operator : Jafari
Sample : M10
Misc :
ALS Vial : 30 Sample Multiplier: 1

Search Libraries: D:\Database\W10N14.L Minimum Quality: 0

Unknown Spectrum: Apex
Integration Events: ChemStation Integrator - events.e

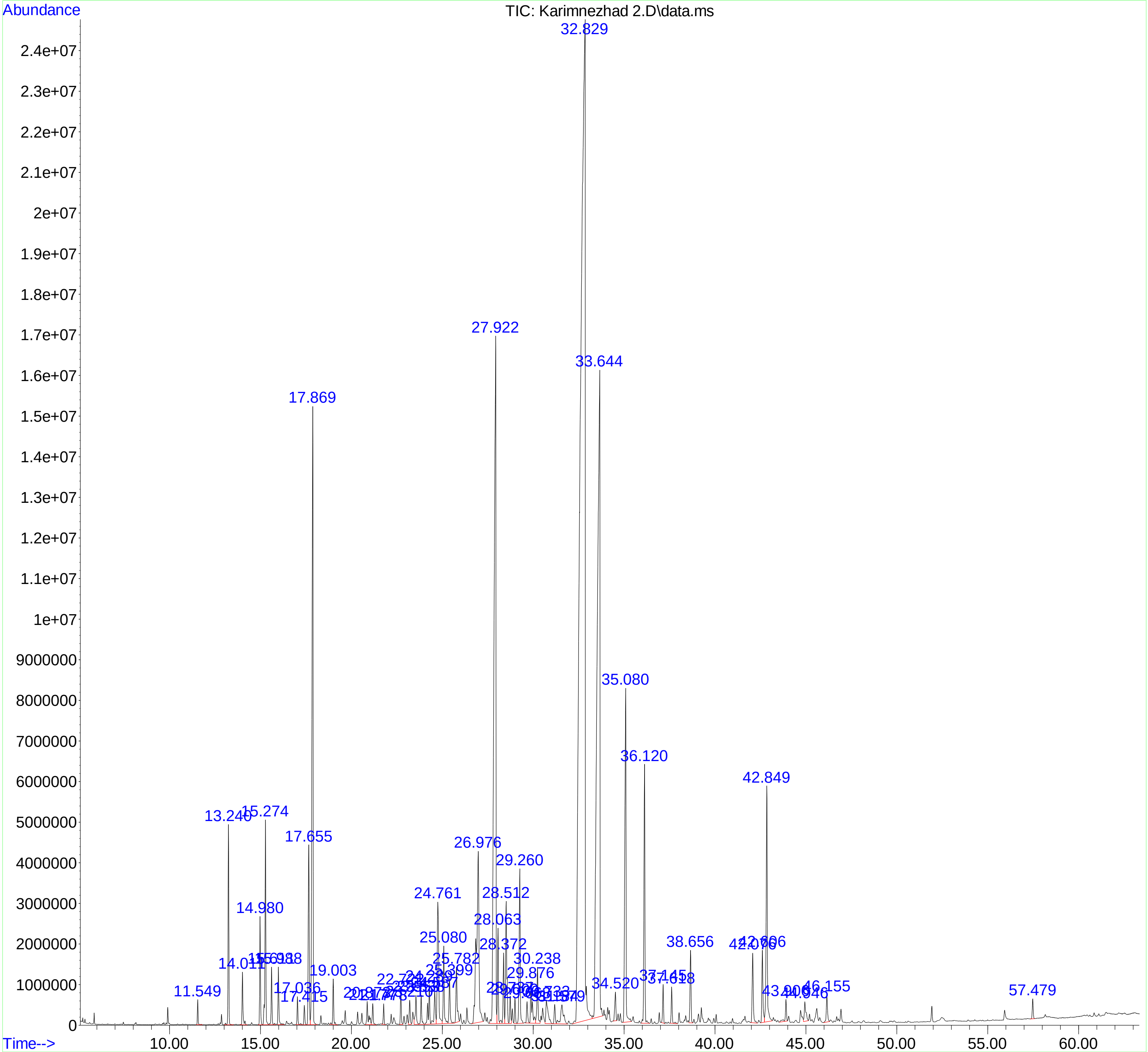

Library Search Report - ChemStation Integrator

Unknown Spectrum based on Apex

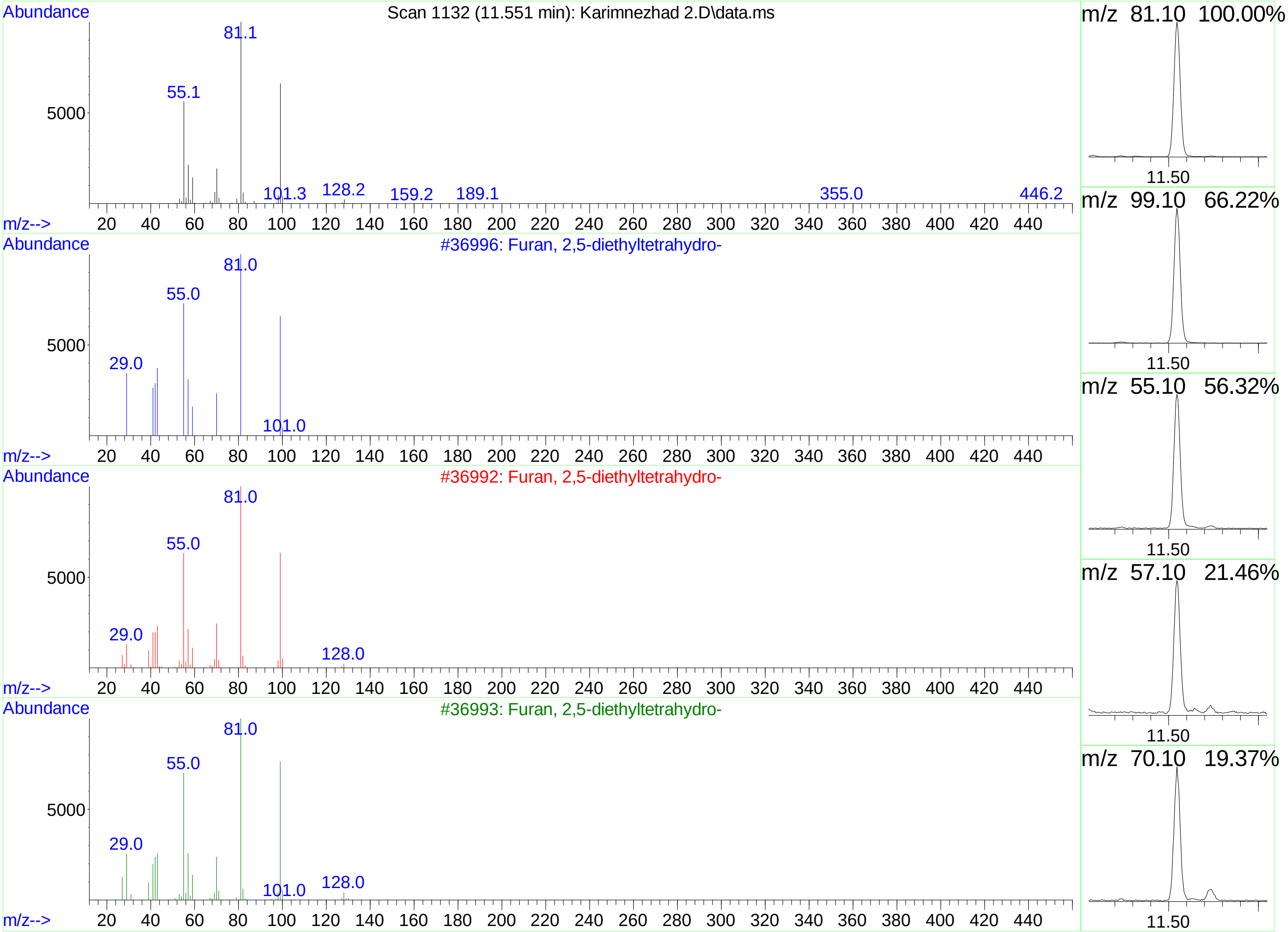

Data File: D:\msdchem\1\data\Karimnezhad 2.D
Sample : M10

Peak Number: 1 at 11.551 min Area: 15846599 Area % 0.13

The 3 best hits from each library.	Ref#	CAS#	Qual
D:\Database\W10N14.L			
1 Furan, 2,5-diethyltetrahydro-	36996	041239-48-9	64
2 Furan, 2,5-diethyltetrahydro-	36992	041239-48-9	53
3 Furan, 2,5-diethyltetrahydro-	36993	041239-48-9	49

Unknown Spectrum based on Apex

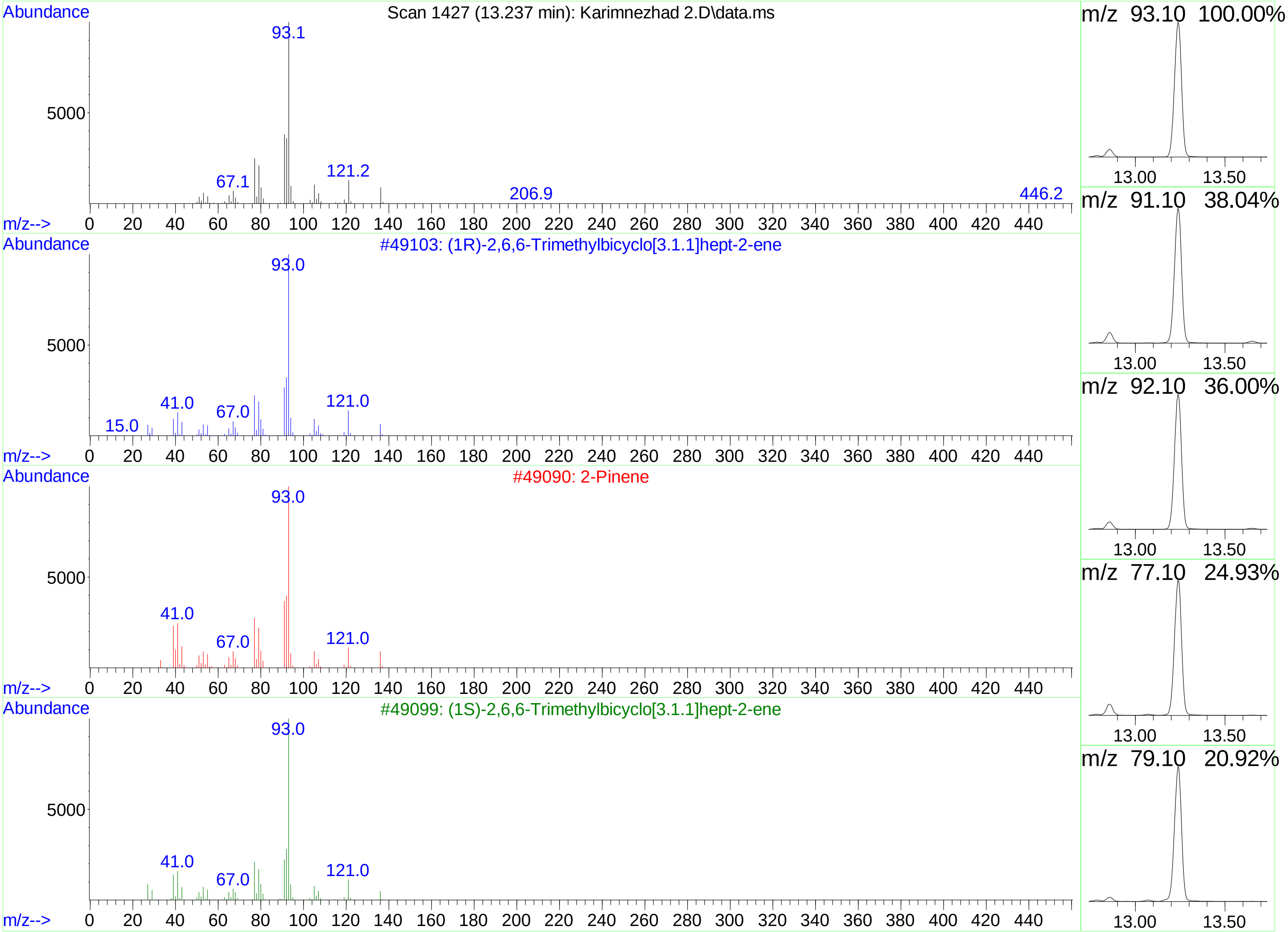

Data File: D:\msdchem\1\data\Karimnezhad 2.D
Sample : M10

Peak Number: 2 at 13.237 min Area: 138838603 Area % 1.16

The 3 best hits from each library. Ref\# CAS\# Qual

D:\Database\W10N14.L

1 (1R)-2,6,6-Trimethylbicyclo[3.1.... 49103 007785-70-8 96
2 2-Pinene 49090 000080-56-8 96
3 (1S)-2,6,6-Trimethylbicyclo[3.1.... 49099 007785-26-4 96

Unknown Spectrum based on Apex

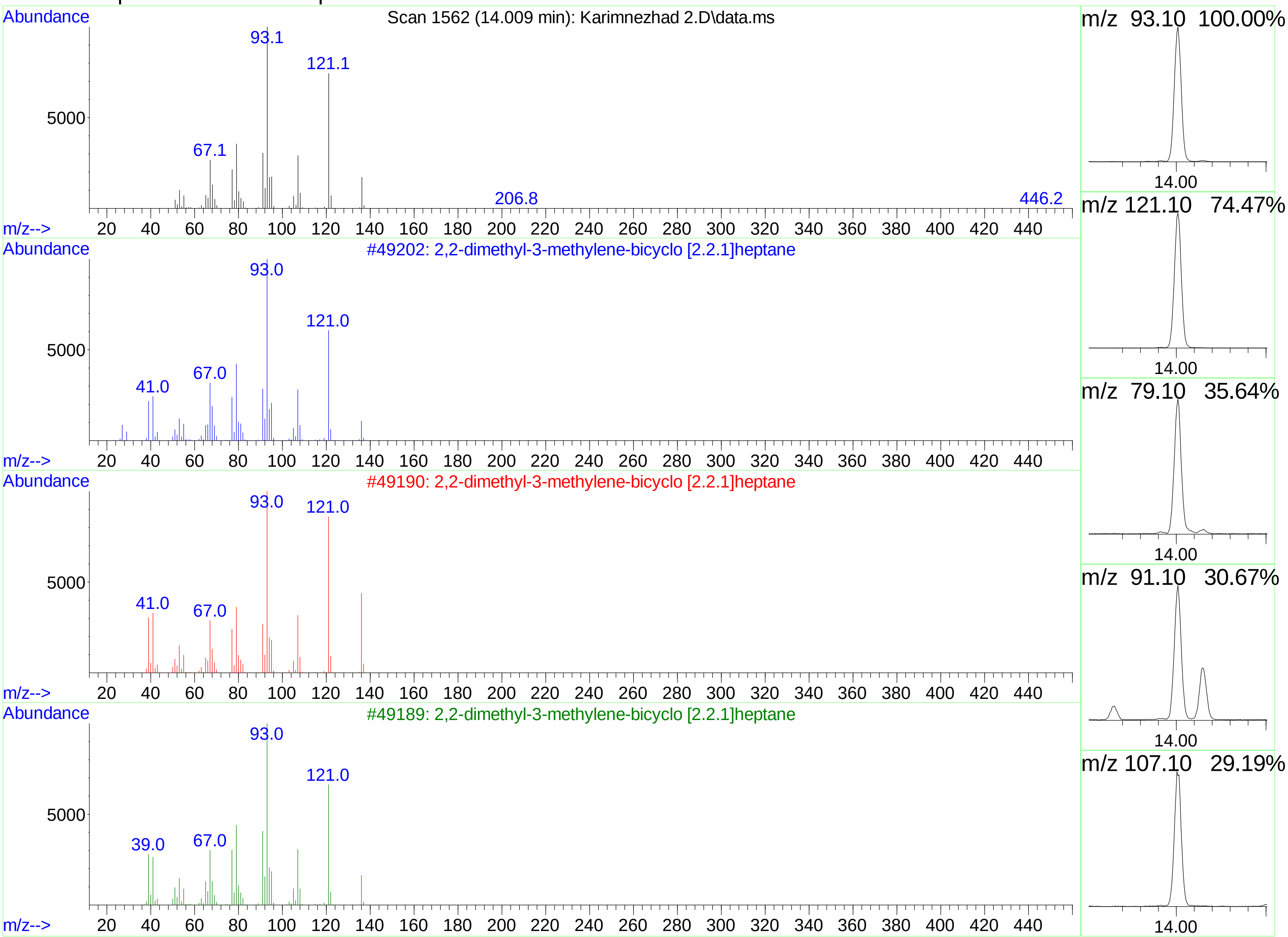

Data File: D:\msdchem\1\data\Karimnezhad 2.D
Sample : M10

Peak Number: 3 at 14.009 min Area: 41752618 Area % 0.35

The 3 best hits from each library.	Ref\#	CAS\#	Qual
D:\Database\W10N14.L			
1 2,2-dimethyl-3-methylene-bicyclo...	49202	000079-92-5	97
2 2,2-dimethyl-3-methylene-bicyclo...	49190	000079-92-5	97
3 2,2-dimethyl-3-methylene-bicyclo...	49189	000079-92-5	97

Unknown Spectrum based on Apex

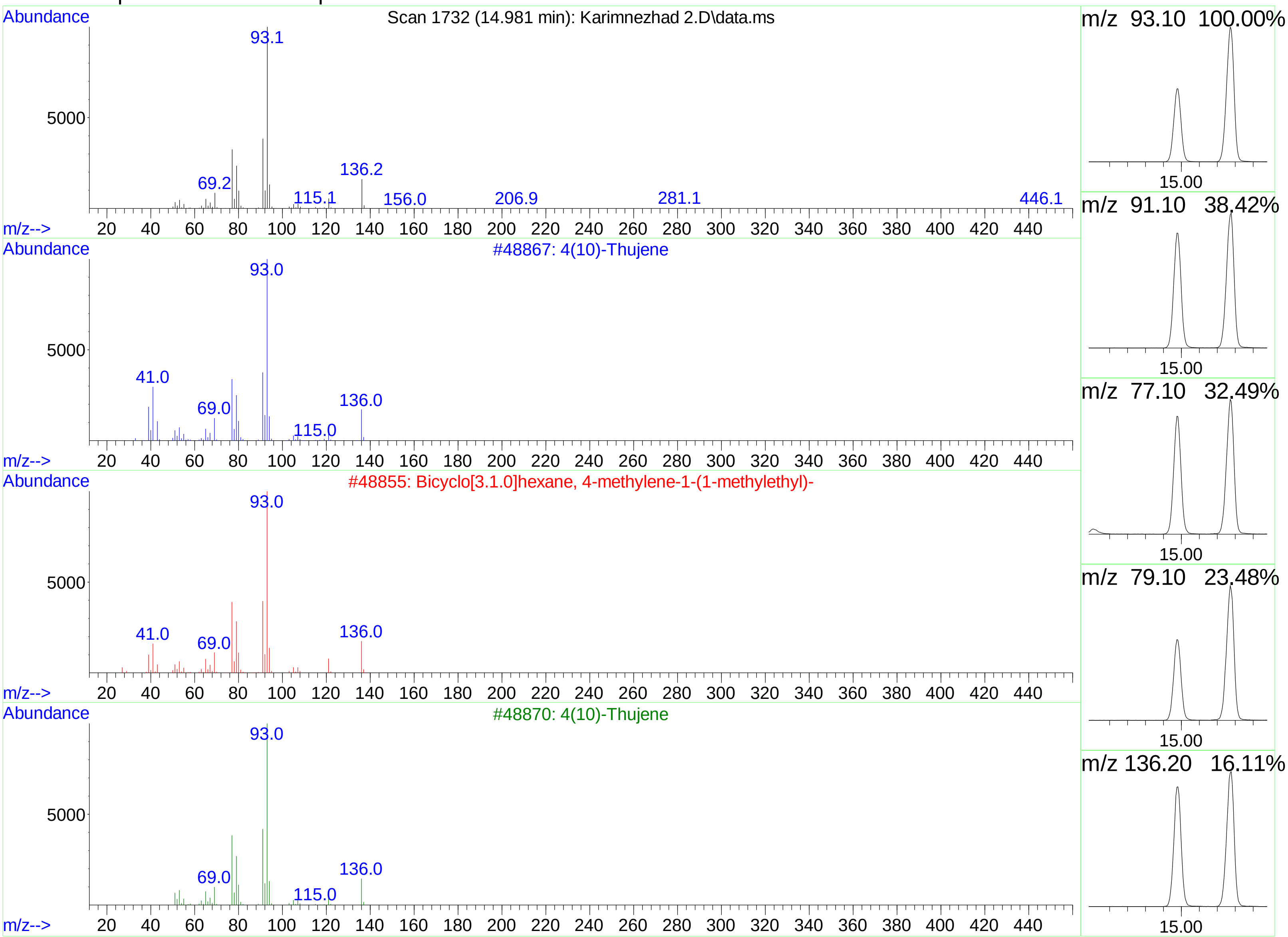

Data File: D:\msdchem\1\data\Karimnezhad 2.D
Sample : M10

Peak Number: 4 at 14.981 min Area: 79560001 Area % 0.67

The 3 best hits from each library.	Ref\#	CAS\#	Qual
D:\Database\W10N14.L			
1 4(10)-Thujene	48867	003387-41-5	97
2 Bicyclo[3.1.0]hexane, 4-methylen...	48855	003387-41-5	96
3 4(10)-Thujene	48870	003387-41-5	96

Unknown Spectrum based on Apex

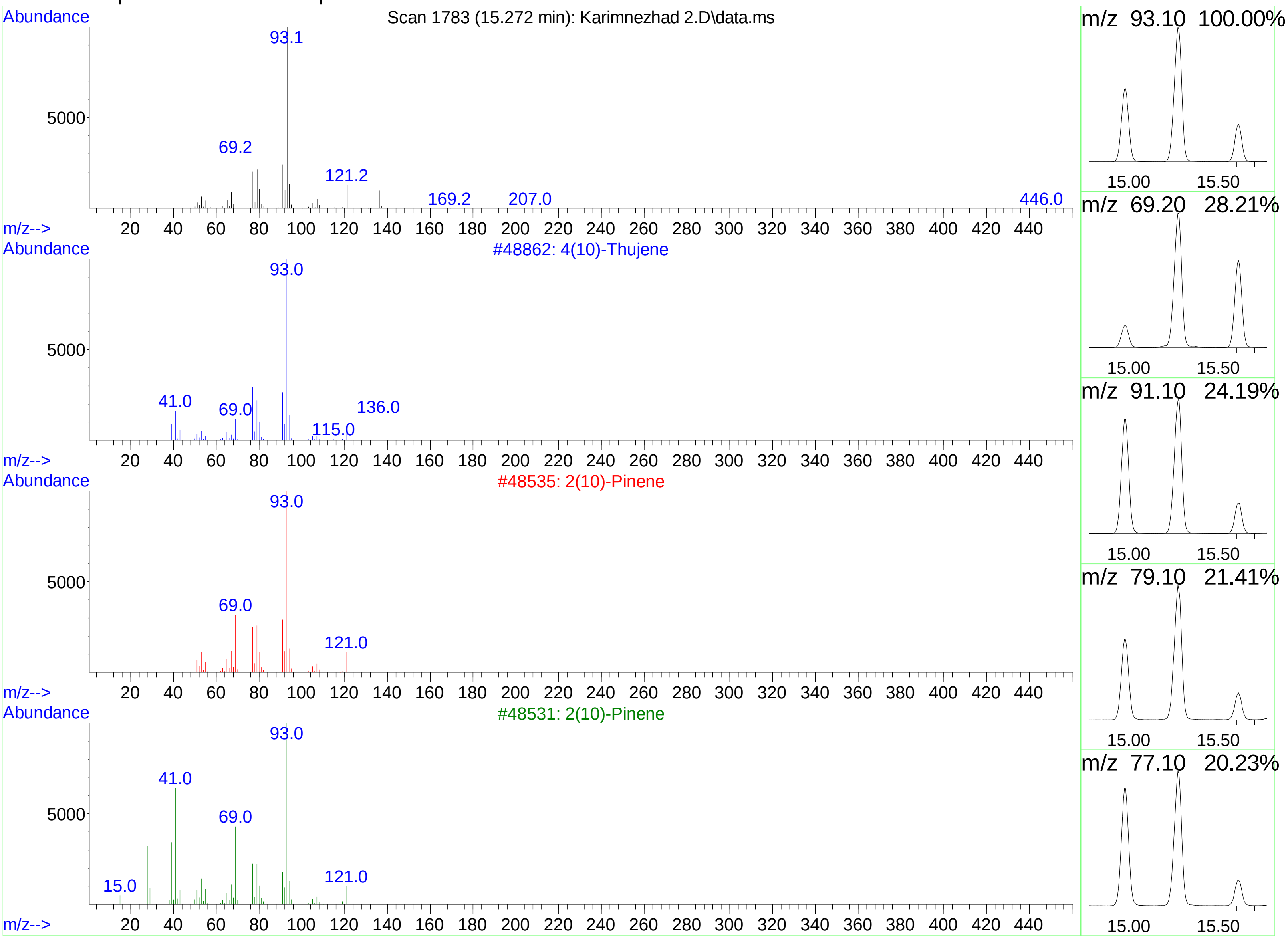

Data File: D:\msdchem\1\data\Karimnezhad 2.D
Sample : M10

Peak Number: 5 at 15.272 min Area: 165926368 Area % 1.39

The 3 best hits from each library.	Ref\#	CAS\#	Qual
D:\Database\W10N14.L			
1 4(10)-Thujene	48862	003387-41-5	94
2 2(10)-Pinene	48535	000127-91-3	94
3 2(10)-Pinene	48531	000127-91-3	94

Unknown Spectrum based on Apex

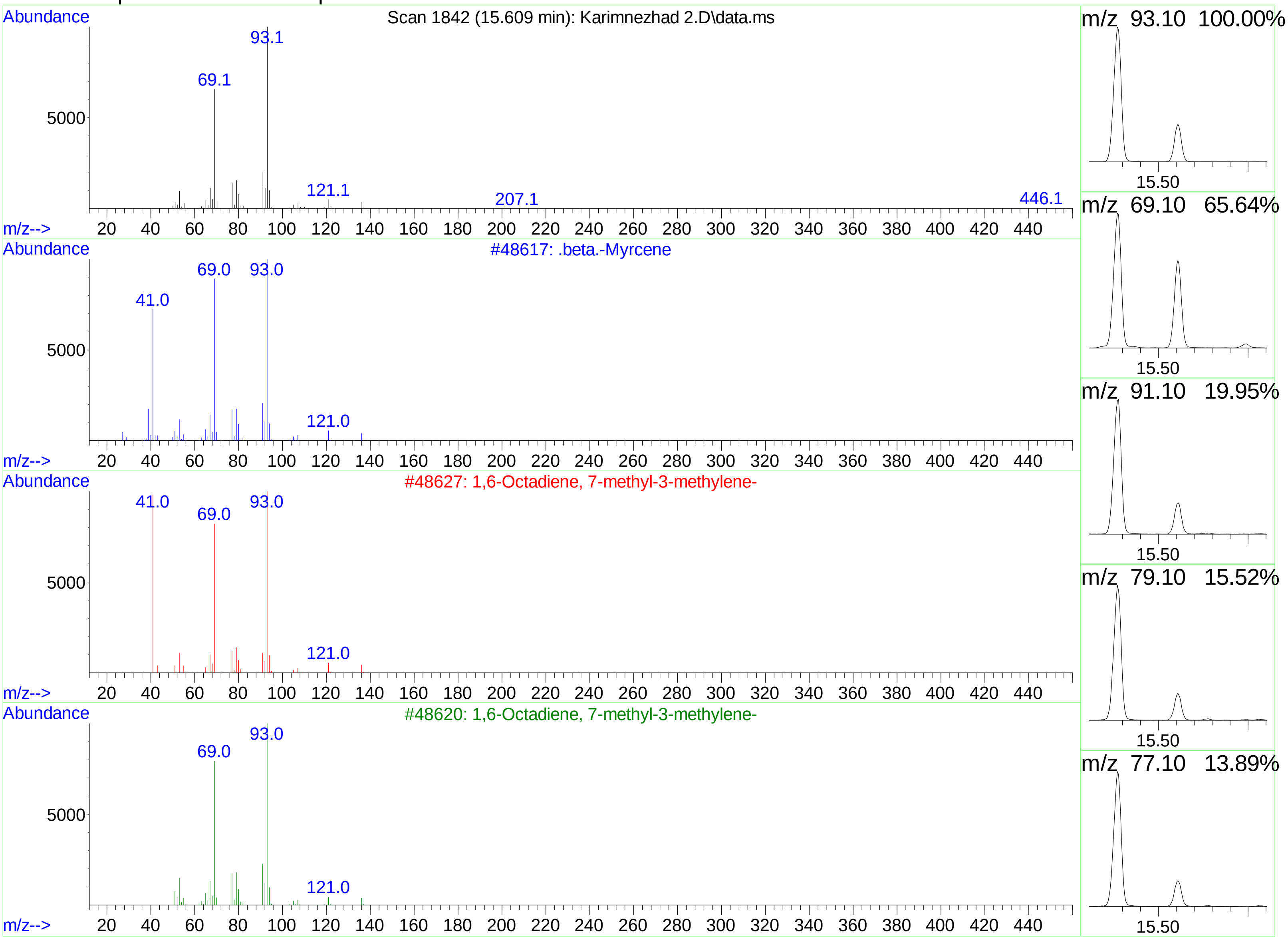

Data File: D:\msdchem\1\data\Karimnezhad 2.D
Sample : M10

Peak Number: 6 at 15.609 min Area: 43352717 Area % 0.36

The 3 best hits from each library.	Ref#	CAS#	Qual
D:\Database\W10N14.L			
1 .beta.-Myrcene	48617	000123-35-3	96
2 1,6-Octadiene, 7-methyl-3-methyl...	48627	000123-35-3	95
3 1,6-Octadiene, 7-methyl-3-methyl...	48620	000123-35-3	94

Library Search Report - ChemStation Integrator

Unknown Spectrum based on Apex

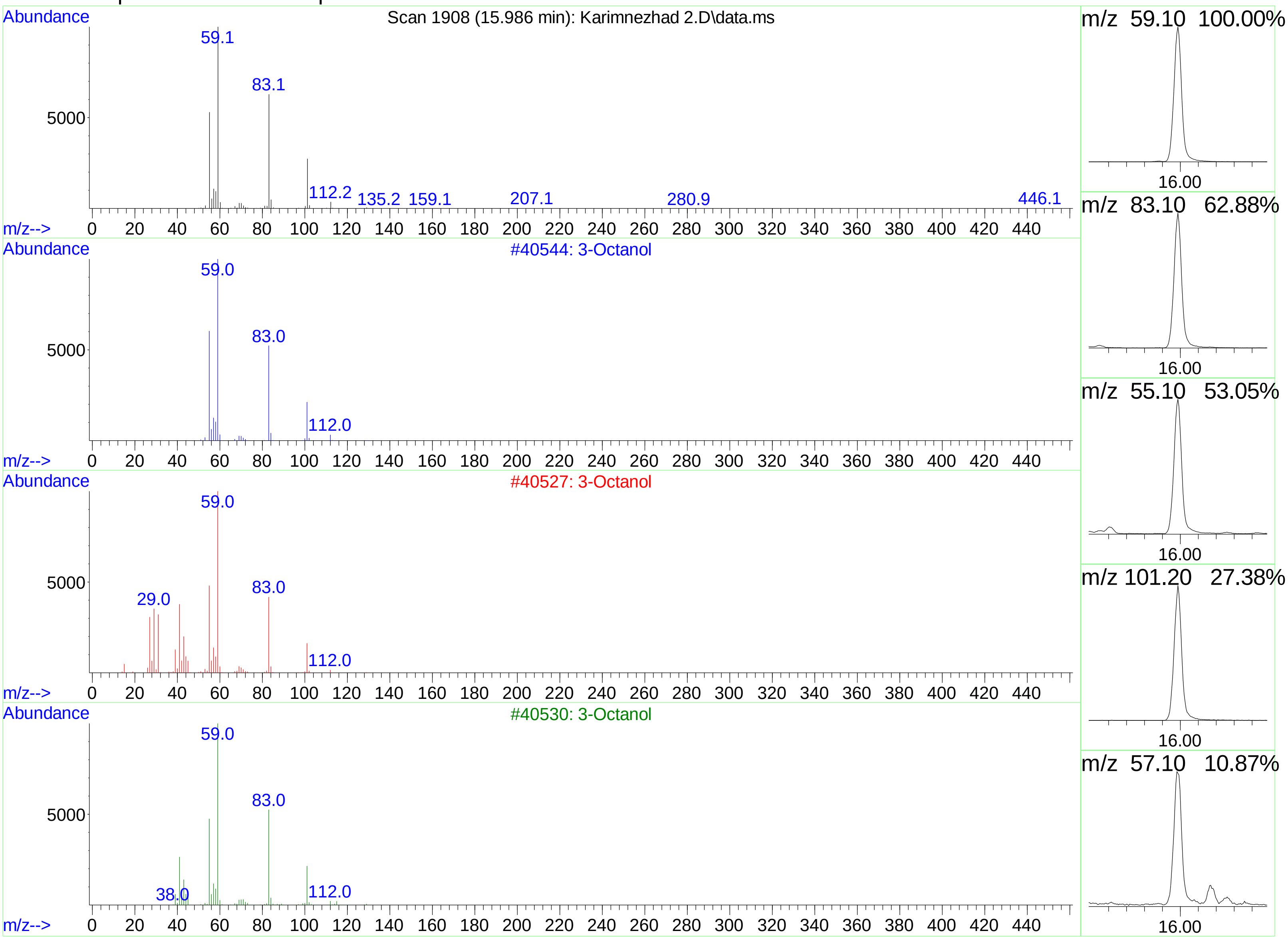

Data File: D:\msdchem\1\data\Karimnezhad 2.D
Sample : M10

Peak Number: 7 at 15.986 min Area: 45375237 Area % 0.38

The 3 best hits from each library.	Ref\#	CAS\#	Qual
D:\Database\W10N14.L			
1 3-Octanol	40544	000589-98-0	90
2 3-Octanol	40527	000589-98-0	83
3 3-Octanol	40530	000589-98-0	83

Library Search Report - ChemStation Integrator

Unknown Spectrum based on Apex

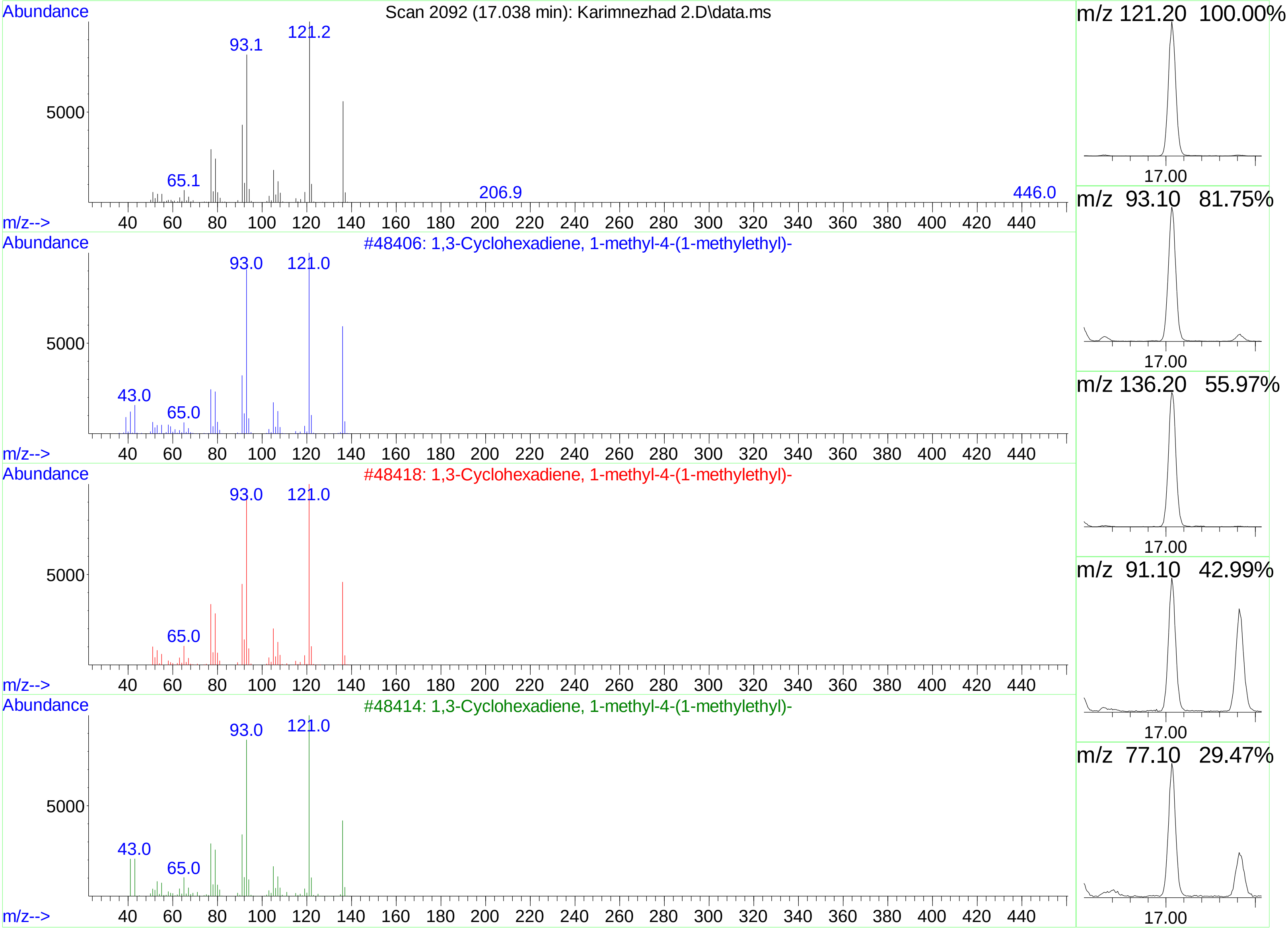

Data File: D:\msdchem\1\data\Karimnezhad 2.D
Sample : M10

Peak Number: 8 at 17.038 min Area: 21002637 Area % 0.18

The 3 best hits from each library.	Ref\#	CAS\#	Qual
D:\Database\W10N14.L			
1 1,3-Cyclohexadiene, 1-methyl-4-(...	48406	000099-86-5	98
2 1,3-Cyclohexadiene, 1-methyl-4-(...	48418	000099-86-5	97
3 1,3-Cyclohexadiene, 1-methyl-4-(...	48414	000099-86-5	97

Unknown Spectrum based on Apex

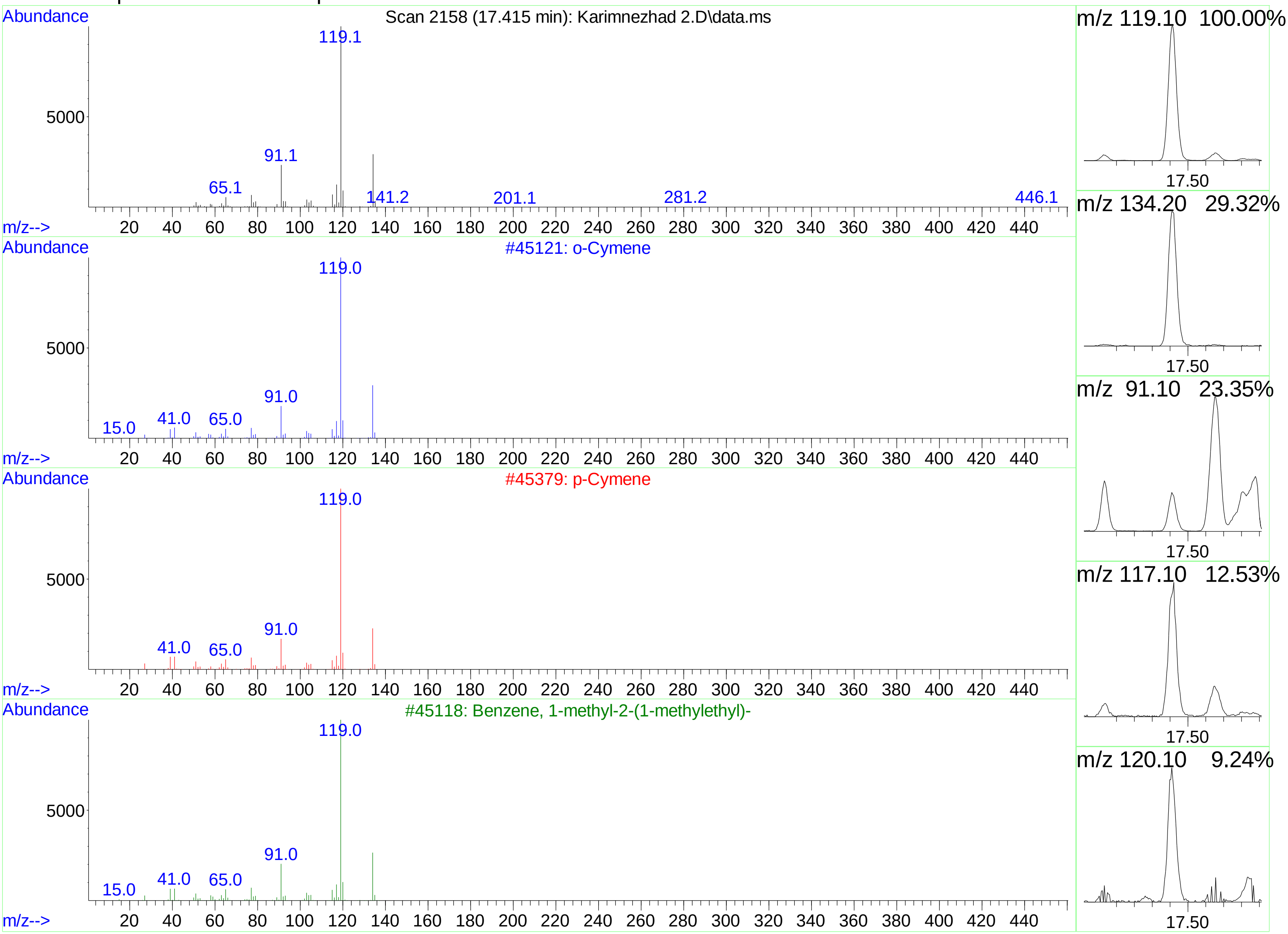

Data File: D:\msdchem\1\data\Karimnezhad 2.D
Sample : M10

Peak Number: 9 at 17.415 min Area: 16028600 Area % 0.13

The 3 best hits from each library.	Ref#	CAS#	Qual
D:\Database\W10N14.L			
1 o-Cymene	45121	000527-84-4	97
2 p-Cymene	45379	000099-87-6	97
3 Benzene, 1-methyl-2-(1-methyleth...	45118	000527-84-4	97

Library Search Report - ChemStation Integrator

Unknown Spectrum based on Apex

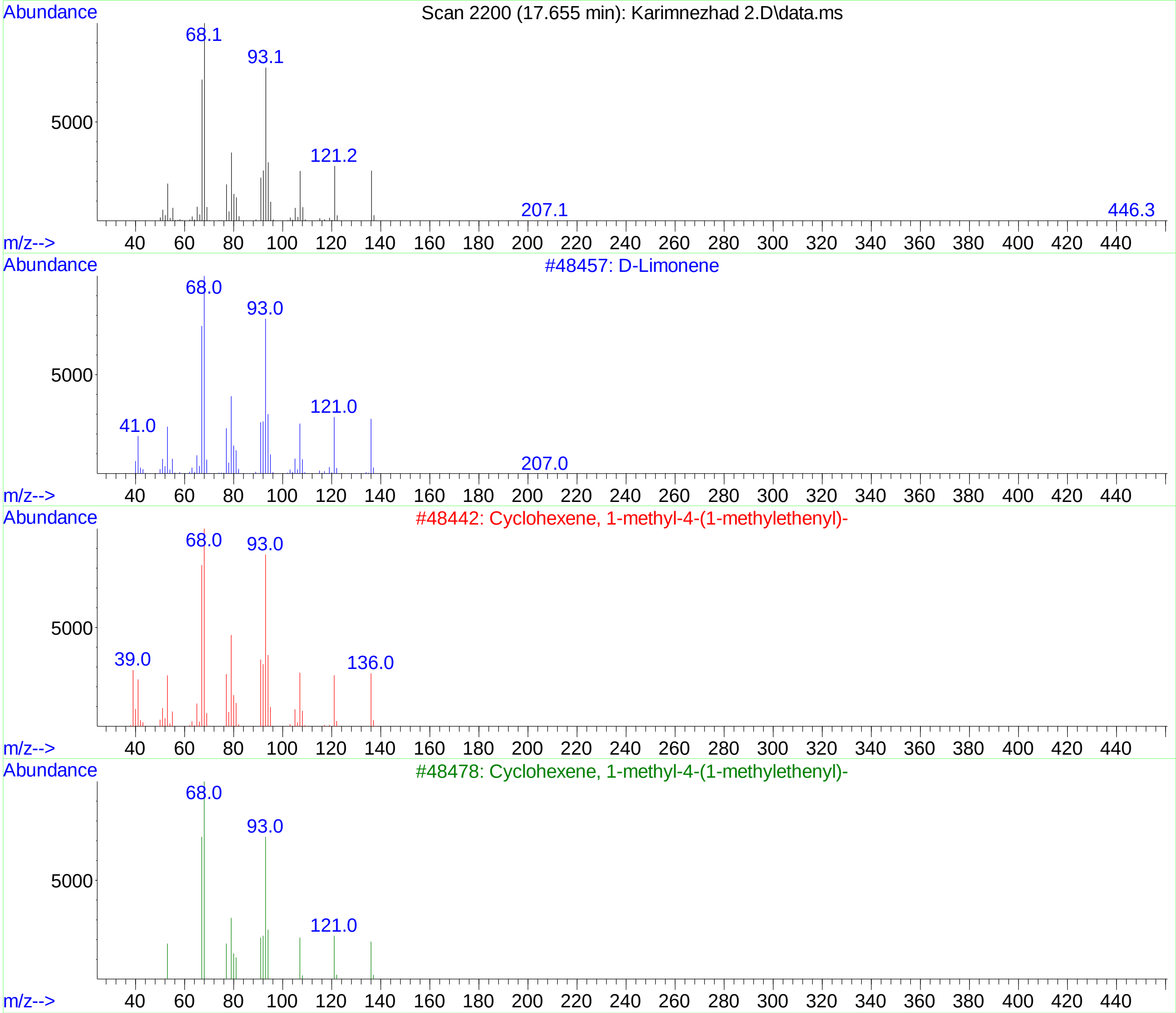

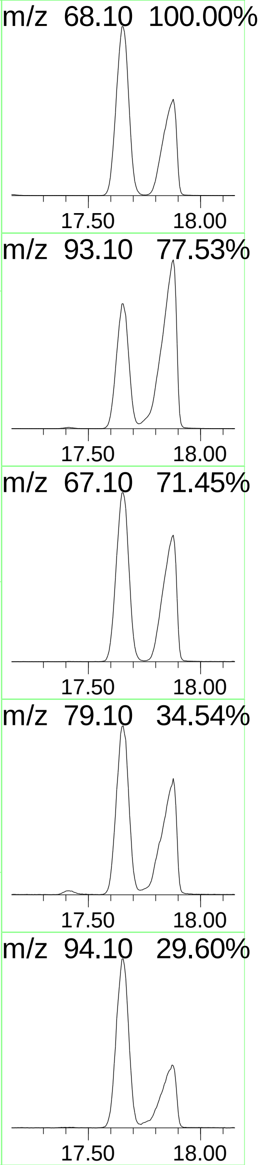

Data File: D:\msdchem\1\data\Karimnezhad 2.D
Sample : M10

Peak Number: 10 at 17.655 min Area: 172086173 Area % 1.44

The 3 best hits from each library.	Ref\#	CAS\#	Qual
D:\Database\W10N14.L			
1 D-Limonene	48457	005989-27-5	99
2 Cyclohexene, 1-methyl-4-(1-methy...	48442	000138-86-3	98
3 Cyclohexene, 1-methyl-4-(1-methy...	48478	000138-86-3	98

Unknown Spectrum based on Apex

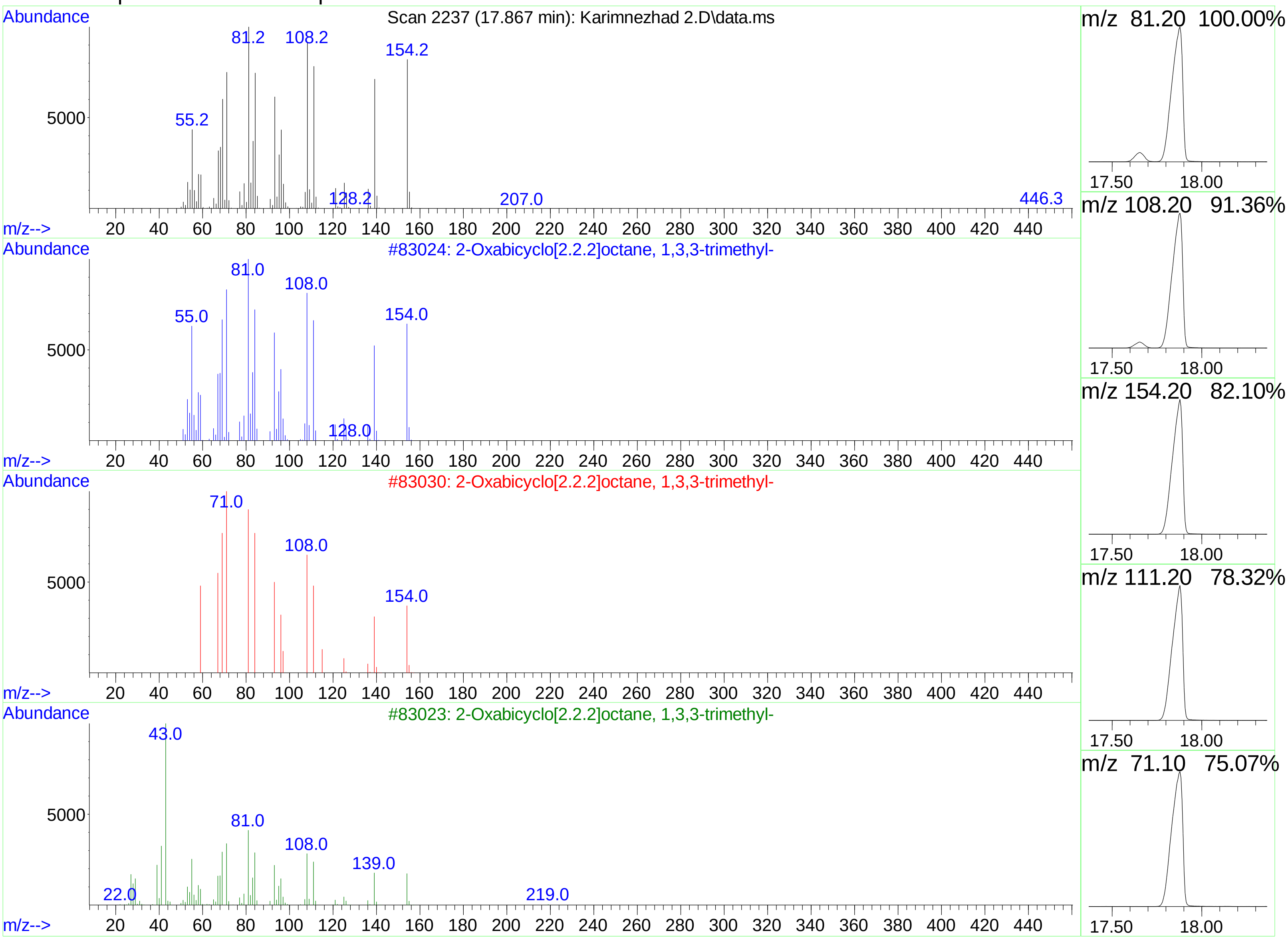

Data File: D:\msdchem\1\data\Karimnezhad 2.D
Sample : M10

Peak Number: 11 at 17.867 min Area: 639391148 Area % 5.35

The 3 best hits from each library.	Ref#	CAS#	Qual
D:\Database\W10N14.L			
1 2-Oxabicyclo[2.2.2]octane, 1,3,3...	83024	000470-82-6	98
2 2-Oxabicyclo[2.2.2]octane, 1,3,3...	83030	000470-82-6	98
3 2-Oxabicyclo[2.2.2]octane, 1,3,3...	83023	000470-82-6	96

Unknown Spectrum based on Apex

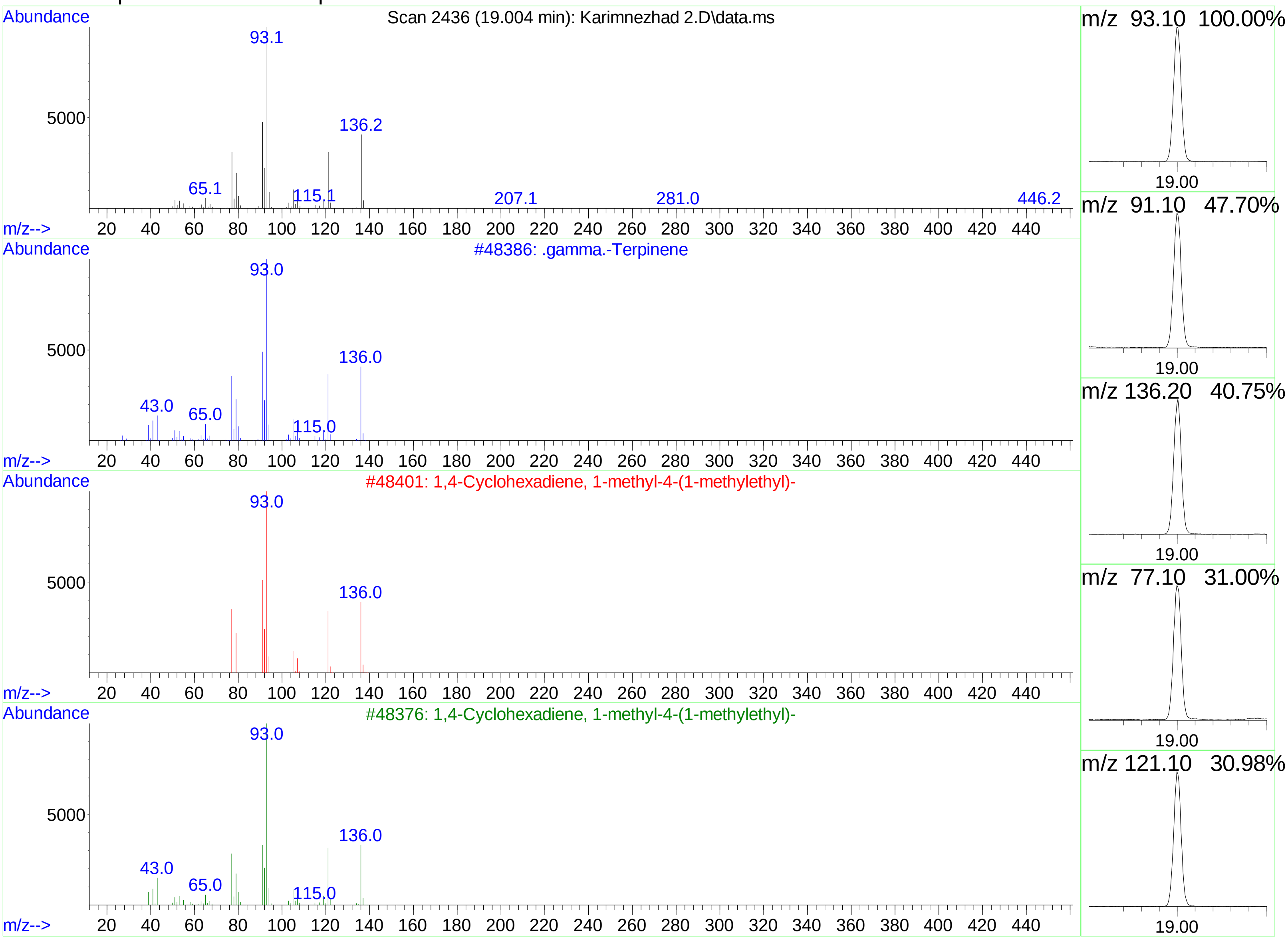

Data File: D:\msdchem\1\data\Karimnezhad 2.D
Sample : M10

Peak Number: 12 at 19.004 min Area: 37014532 Area % 0.31

The 3 best hits from each library.	Ref#	CAS#	Qual
D:\Database\W10N14.L			
1 .gamma.-Terpinene	48386	000099-85-4	97
2 1,4-Cyclohexadiene, 1-methyl-4-(...	48401	000099-85-4	97
3 1,4-Cyclohexadiene, 1-methyl-4-(...	48376	000099-85-4	96

Unknown Spectrum based on Apex

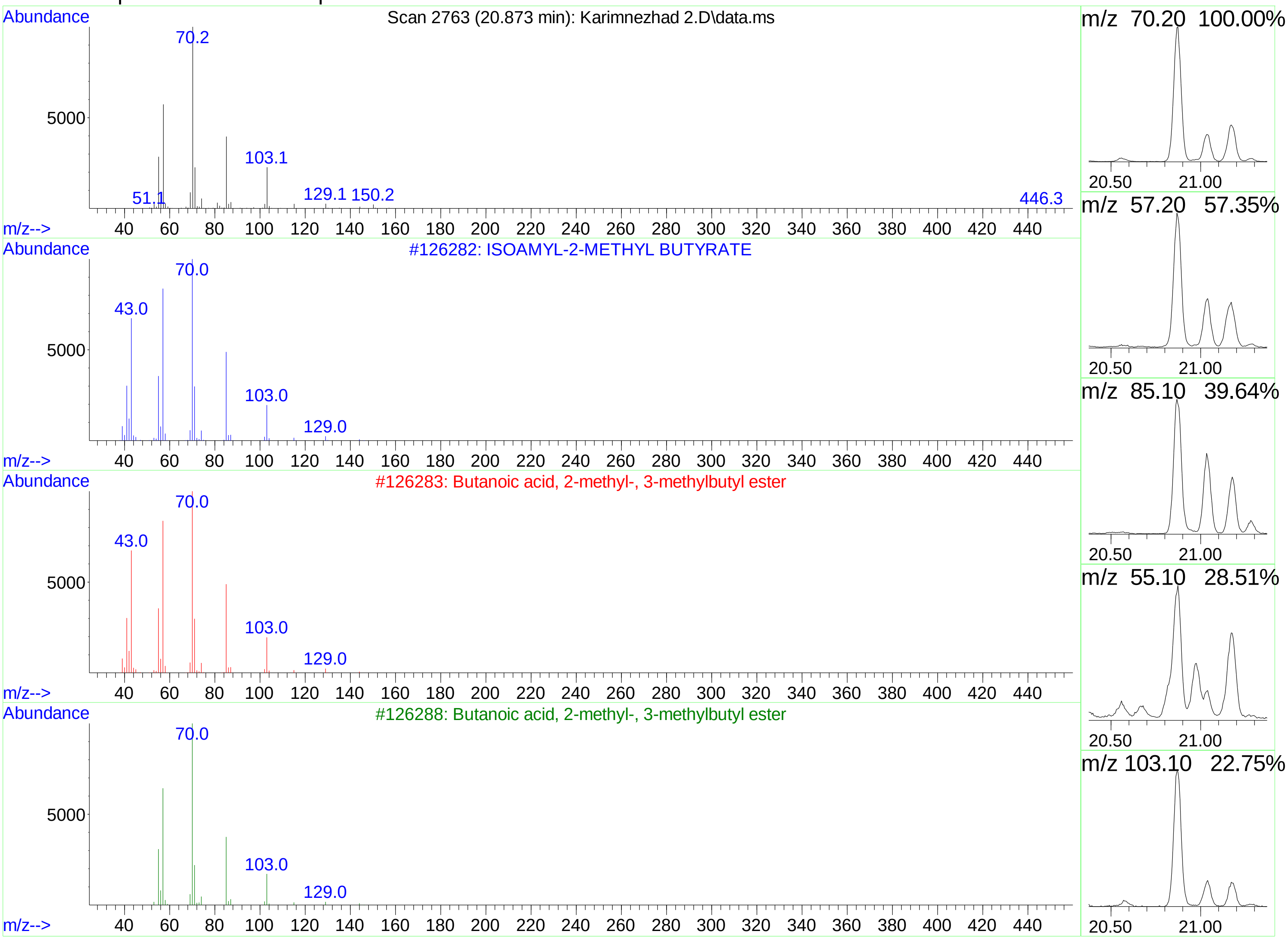

Data File: D:\msdchem\1\data\Karimnezhad 2.D
Sample : M10

Peak Number: 13 at 20.873 min Area: 31779700 Area % 0.27

The 3 best hits from each library.	Ref#	CAS#	Qual
D:\Database\W10N14.L			
1 ISOAMYL-2-METHYL BUTYRATE	126282	027625-35-0	86
2 Butanoic acid, 2-methyl-, 3-meth...	126283	027625-35-0	86
3 Butanoic acid, 2-methyl-, 3-meth...	126288	027625-35-0	86

Unknown Spectrum based on Apex

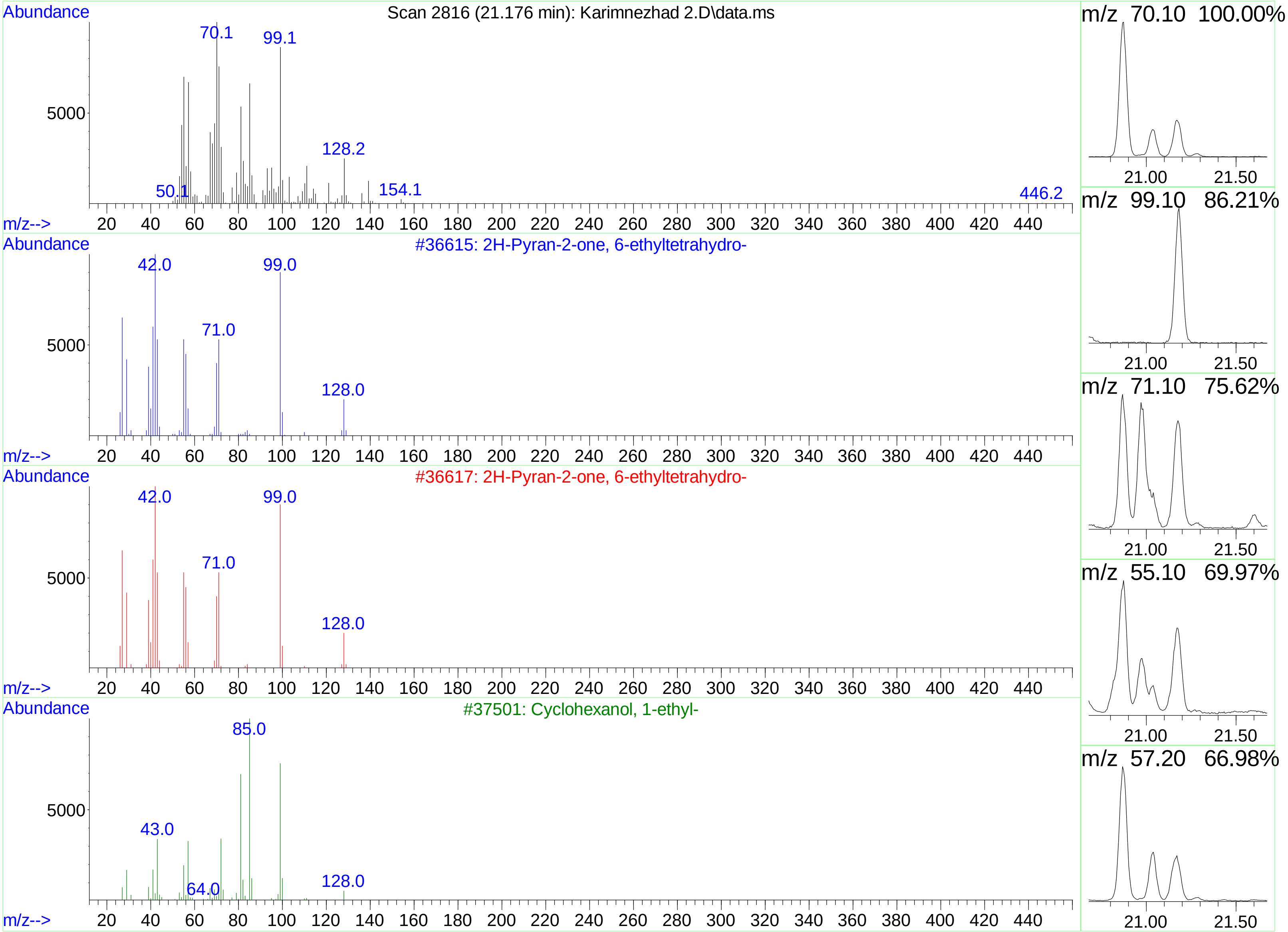

Data File: D:\msdchem\1\data\Karimnezhad 2.D
Sample : M10

Peak Number: 14 at 21.176 min Area: 19985000 Area % 0.17

The 3 best hits from each library.	Ref#	CAS#	Qual
D:\Database\W10N14.L			
1 2H-Pyran-2-one, 6-ethyltetrahydro-	36615	003301-90-4	43
2 2H-Pyran-2-one, 6-ethyltetrahydro-	36617	003301-90-4	43
3 Cyclohexanol, 1-ethyl-	37501	001940-18-7	38

Library Search Report - ChemStation Integrator

Unknown Spectrum based on Apex

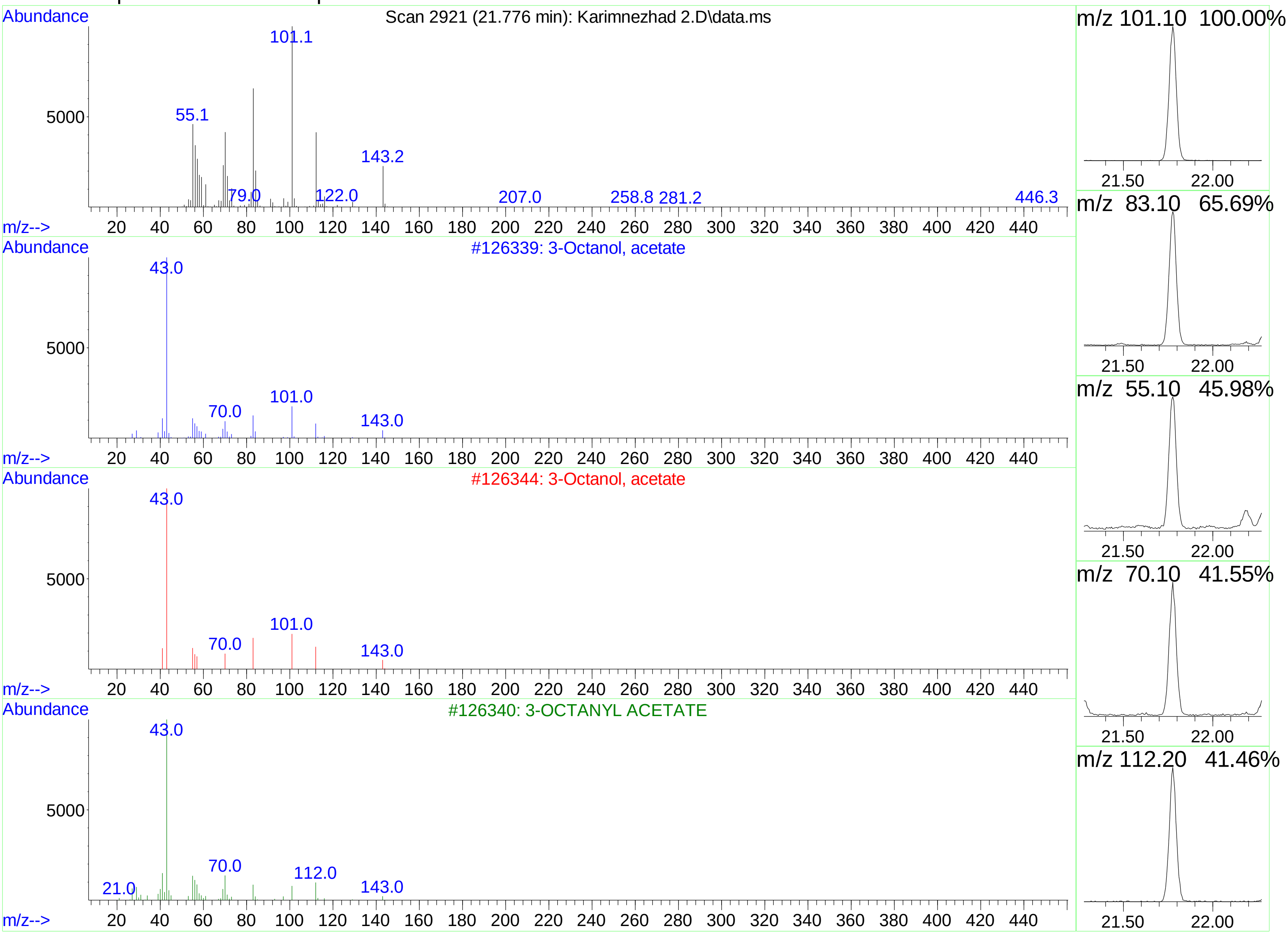

Data File: D:\msdchem\1\data\Karimnezhad 2.D
Sample : M10

Peak Number: 15 at 21.776 min Area: 14896747 Area % 0.12

The 3 best hits from each library.	Ref\#	CAS\#	Qual
D:\Database\W10N14.L			
1 3-Octanol, acetate	126339	004864-61-3	91
2 3-Octanol, acetate	126344	004864-61-3	50
3 3-OCTANYL ACETATE	126340	004864-61-3	43

Library Search Report - ChemStation Integrator

Unknown Spectrum based on Apex

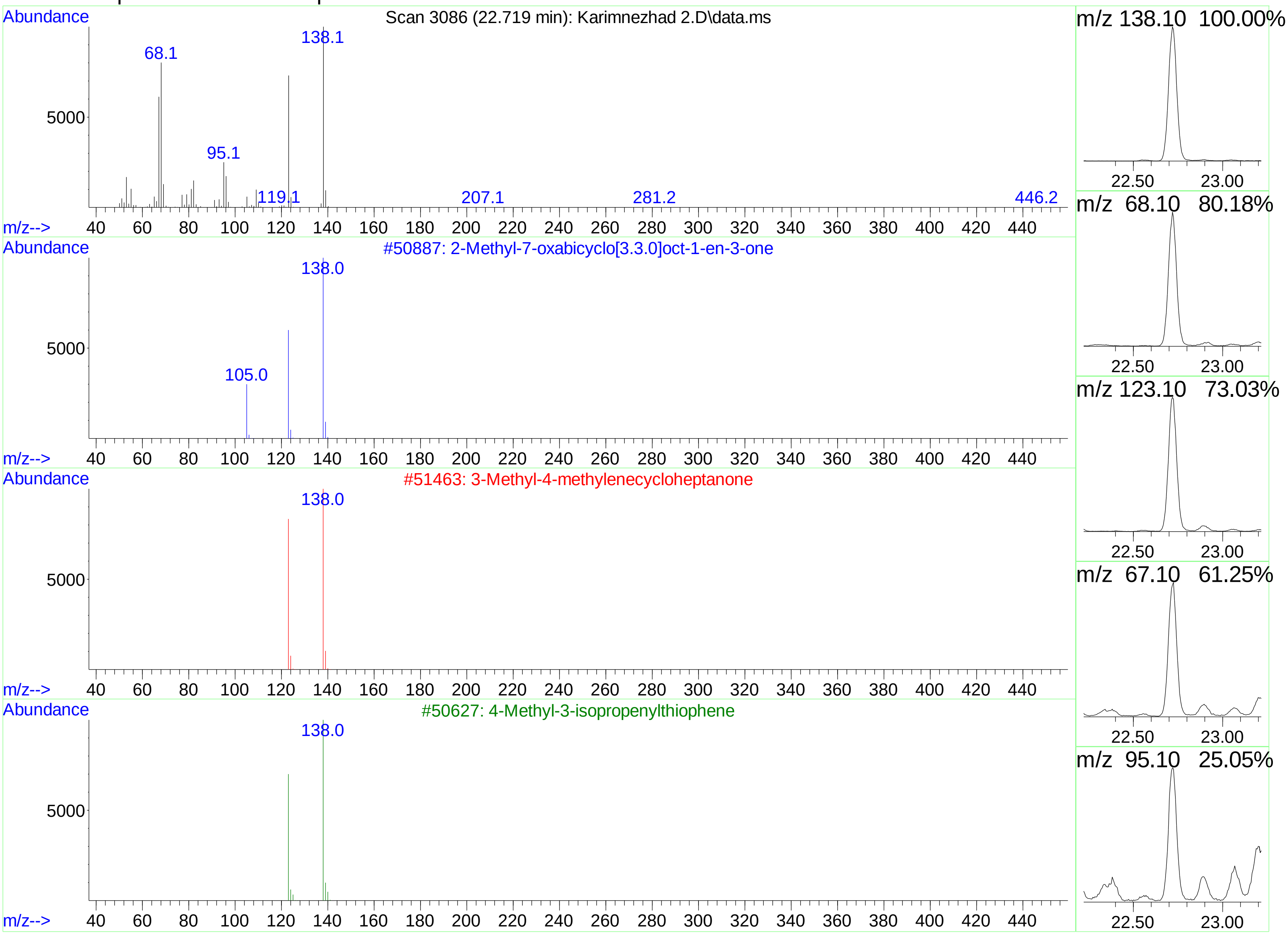

Data File: D:\msdchem\1\data\Karimnezhad 2.D
Sample : M10

Peak Number: 16 at 22.719 min Area: 29982610 Area % 0.25

The 3 best hits from each library. Ref\# CAS\# Qual

D:\Database\W10N14.L

1	2-Methyl-7-oxabicyclo[3.3.0]oct-...	50887	2000050-88-7	86
2	3-Methyl-4-methylenecycloheptanone	51463	2000051-46-3	83
3	4-Methyl-3-isopropenylthiophene	50627	2000050-62-7	83

Library Search Report - ChemStation Integrator

Unknown Spectrum based on Apex

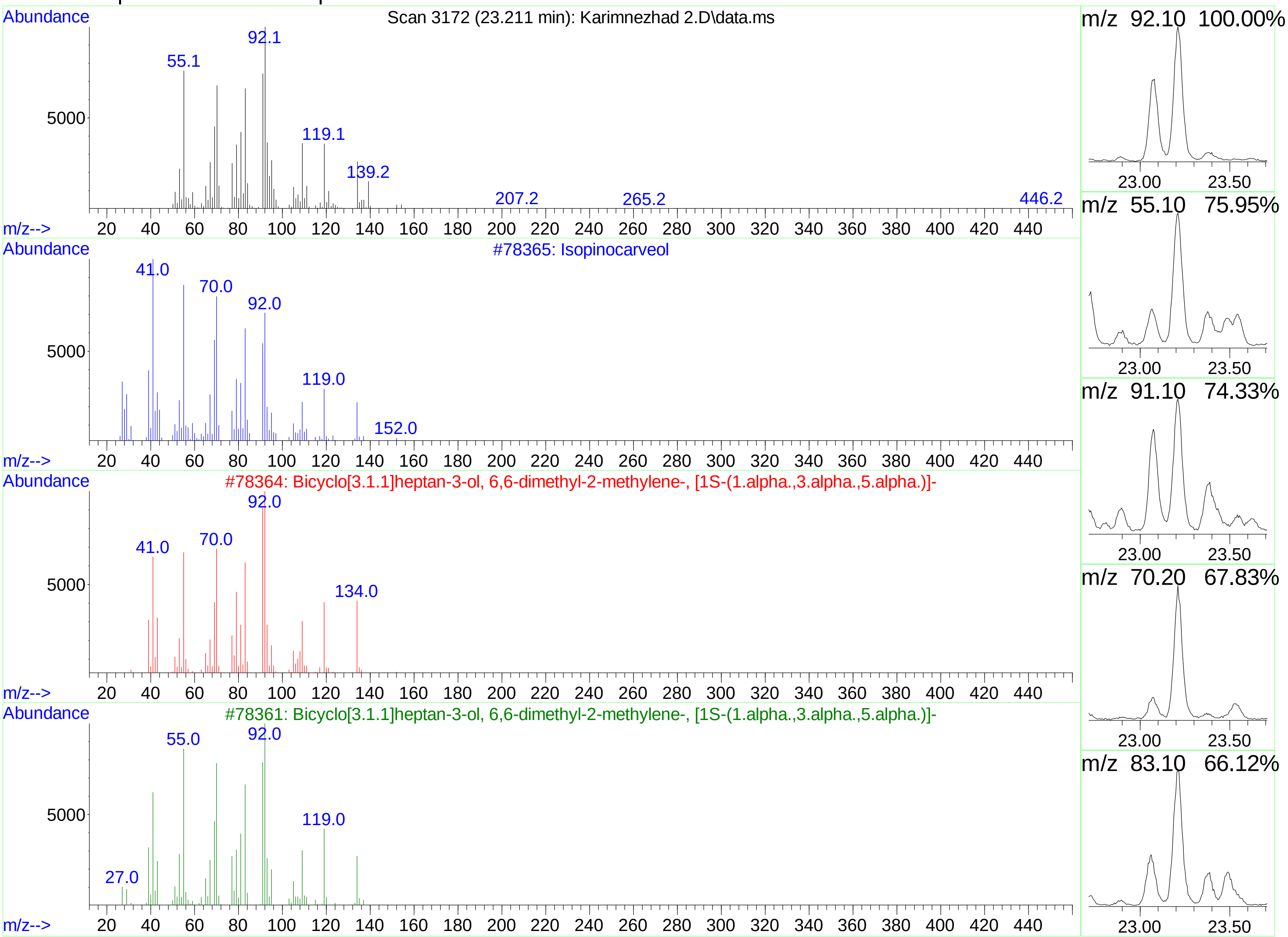

Data File: D:\msdchem\1\data\Karimnezhad 2.D
Sample : M10

Peak Number: 17 at 23.211 min Area: 22806977 Area % 0.19

The 3 best hits from each library.	Ref#	CAS#	Qual
D:\Database\W10N14.L			
1 Isopinocarveol	78365	006712-79-4	94
2 Bicyclo[3.1.1]heptan-3-ol, 6,6-d...	78364	000547-61-5	87
3 Bicyclo[3.1.1]heptan-3-ol, 6,6-d...	78361	000547-61-5	83

Unknown Spectrum based on Apex

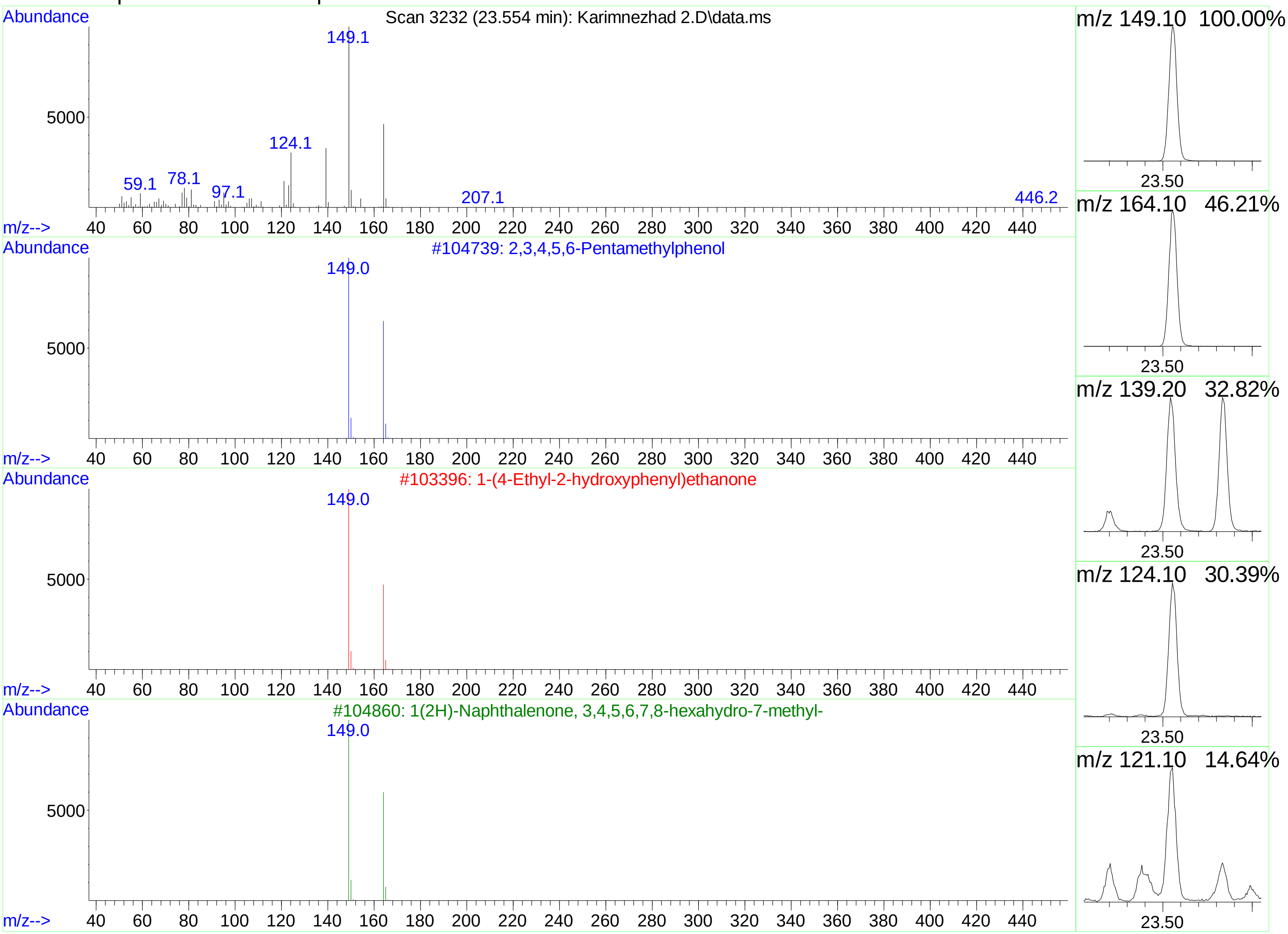

Data File: D:\msdchem\1\data\Karimnezhad 2.D
Sample : M10

Peak Number: 18 at 23.554 min Area: 32826877 Area % 0.27

The 3 best hits from each library.	Ref\#	CAS\#	Qual
D:\Database\W10N14.L			
1 2,3,4,5,6-Pentamethylphenol	104739	2000104-73-9	83
2 1-(4-Ethyl-2-hydroxyphenyl)ethanone	103396	2000103-39-6	83
3 1(2H)-Naphthalenone, 3,4,5,6,7,8...	104860	059177-21-8	83

Library Search Report - ChemStation Integrator

Unknown Spectrum based on Apex

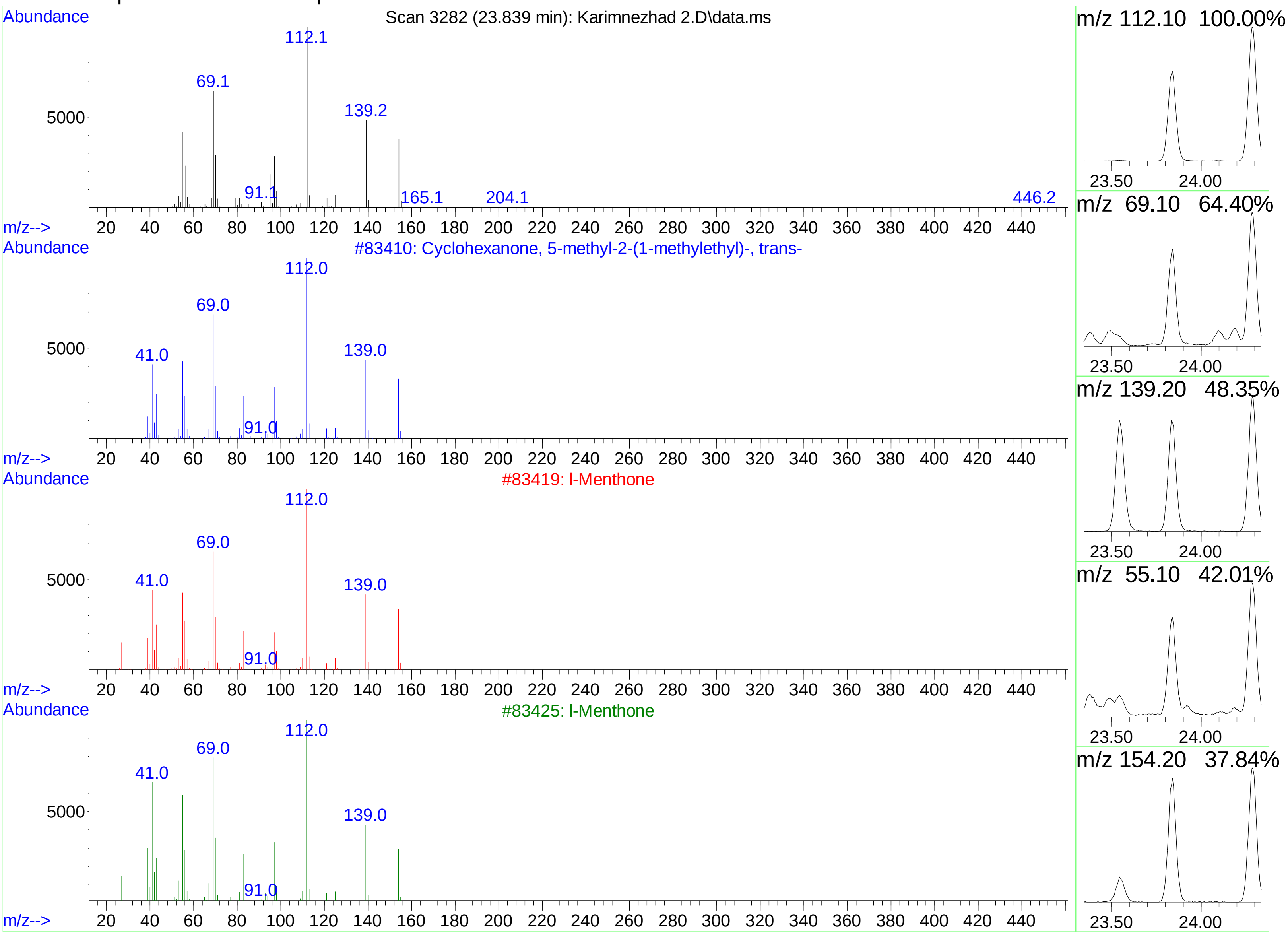

Data File: D:\msdchem\1\data\Karimnezhad 2.D
Sample : M10

Peak Number: 19 at 23.839 min Area: 27295124 Area % 0.23

The 3 best hits from each library.	Ref\#	CAS\#	Qual
D:\Database\W10N14.L			
1 Cyclohexanone, 5-methyl-2-(1-met...	83410	000089-80-5	98
2 l-Menthone	83419	014073-97-3	97
3 l-Menthone	83425	014073-97-3	97

Unknown Spectrum based on Apex

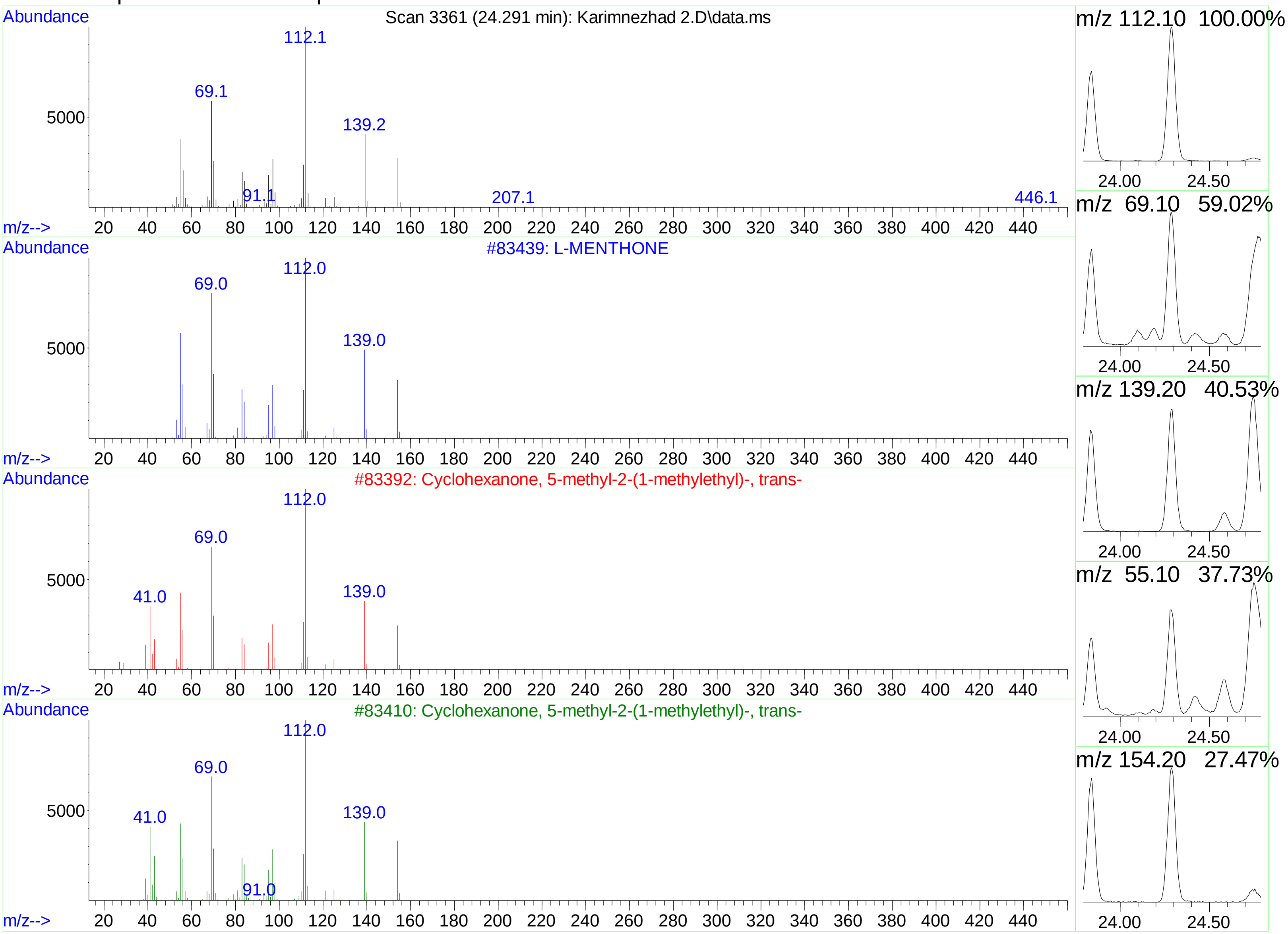

Data File: D:\msdchem\1\data\Karimnezhad 2.D
Sample : M10

Peak Number: 20 at 24.291 min Area: 49517247 Area % 0.41

The 3 best hits from each library.	Ref#	CAS#	Qual
D:\Database\W10N14.L			
1 L-MENTHONE	83439	010458-14-7	98
2 Cyclohexanone, 5-methyl-2-(1-met...	83392	000089-80-5	98
3 Cyclohexanone, 5-methyl-2-(1-met...	83410	000089-80-5	98

Unknown Spectrum based on Apex

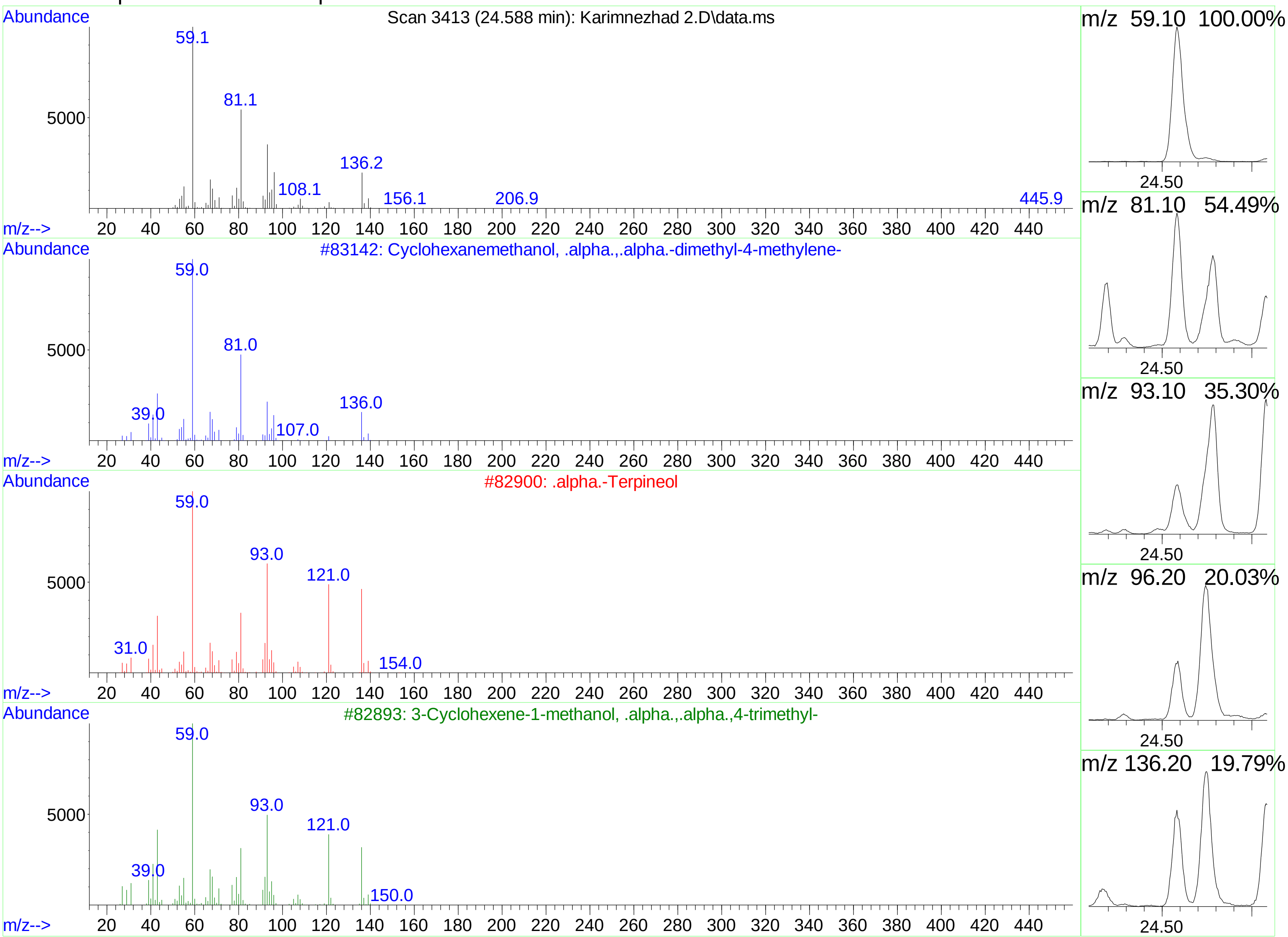

Data File: D:\msdchem\1\data\Karimnezhad 2.D
Sample : M10

Peak Number: 21 at 24.588 min Area: 40182750 Area % 0.34

The 3 best hits from each library. Ref# CAS# Qual

D:\Database\W10N14.L

1	Cyclohexanemethanol, .alpha.,.al...	83142	007299-42-5	86
2	.alpha.-Terpineol	82900	000098-55-5	64
3	3-Cyclohexene-1-methanol, .alpha...	82893	010482-56-1	59

Unknown Spectrum based on Apex

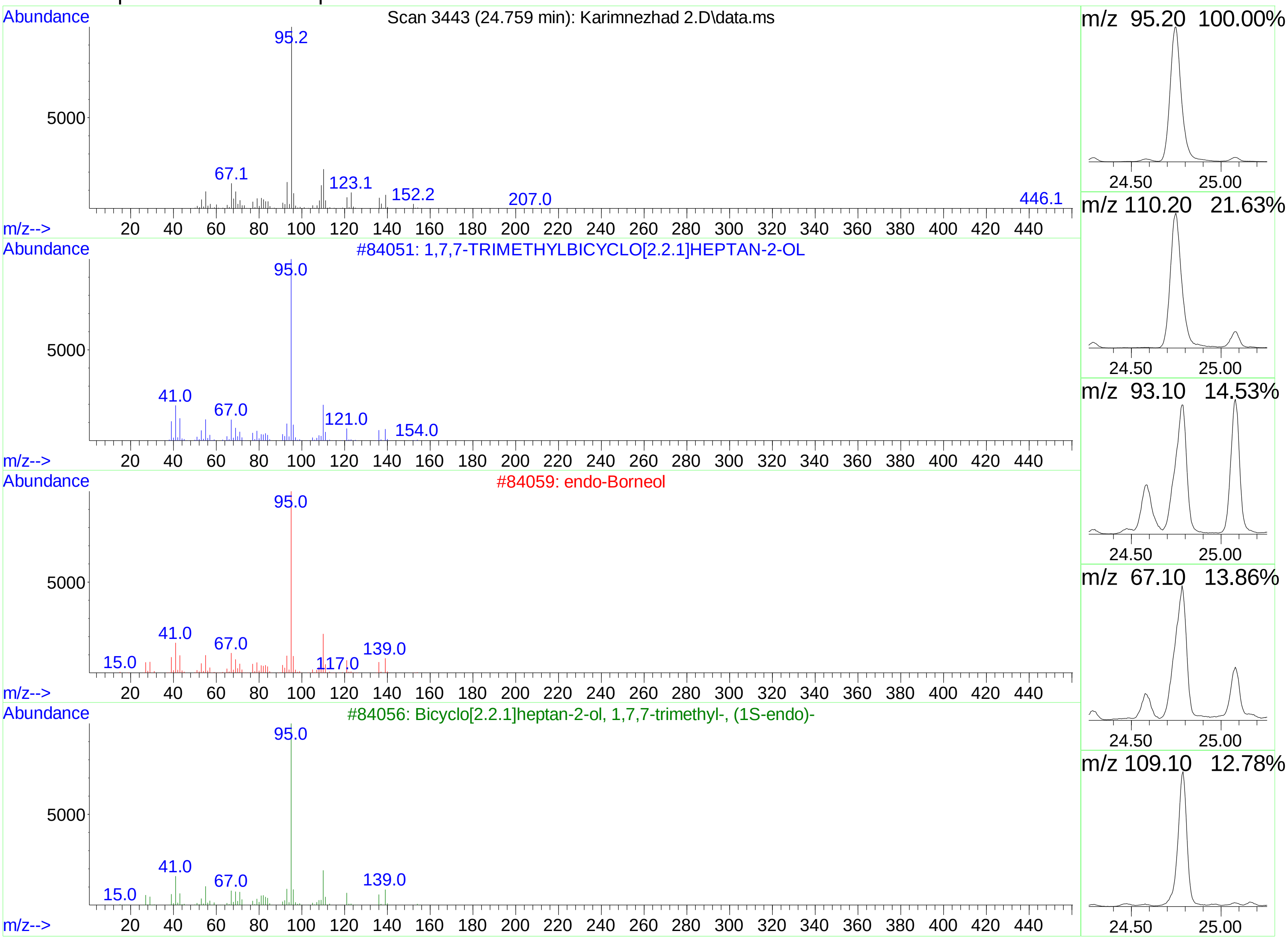

Data File: D:\msdchem\1\data\Karimnezhad 2.D
Sample : M10

Peak Number: 22 at 24.759 min Area: 171653266 Area % 1.44

The 3 best hits from each library. Ref\# CAS\# Qual

D:\Database\W10N14.L

1	1,7,7-TRIMETHYLBICYCLO[2.2.1]HEP...	84051	000464-45-9	94
2	endo-Borneol	84059	000507-70-0	94
3	Bicyclo[2.2.1]heptan-2-ol, 1,7,7...	84056	000464-45-9	90

Unknown Spectrum based on Apex

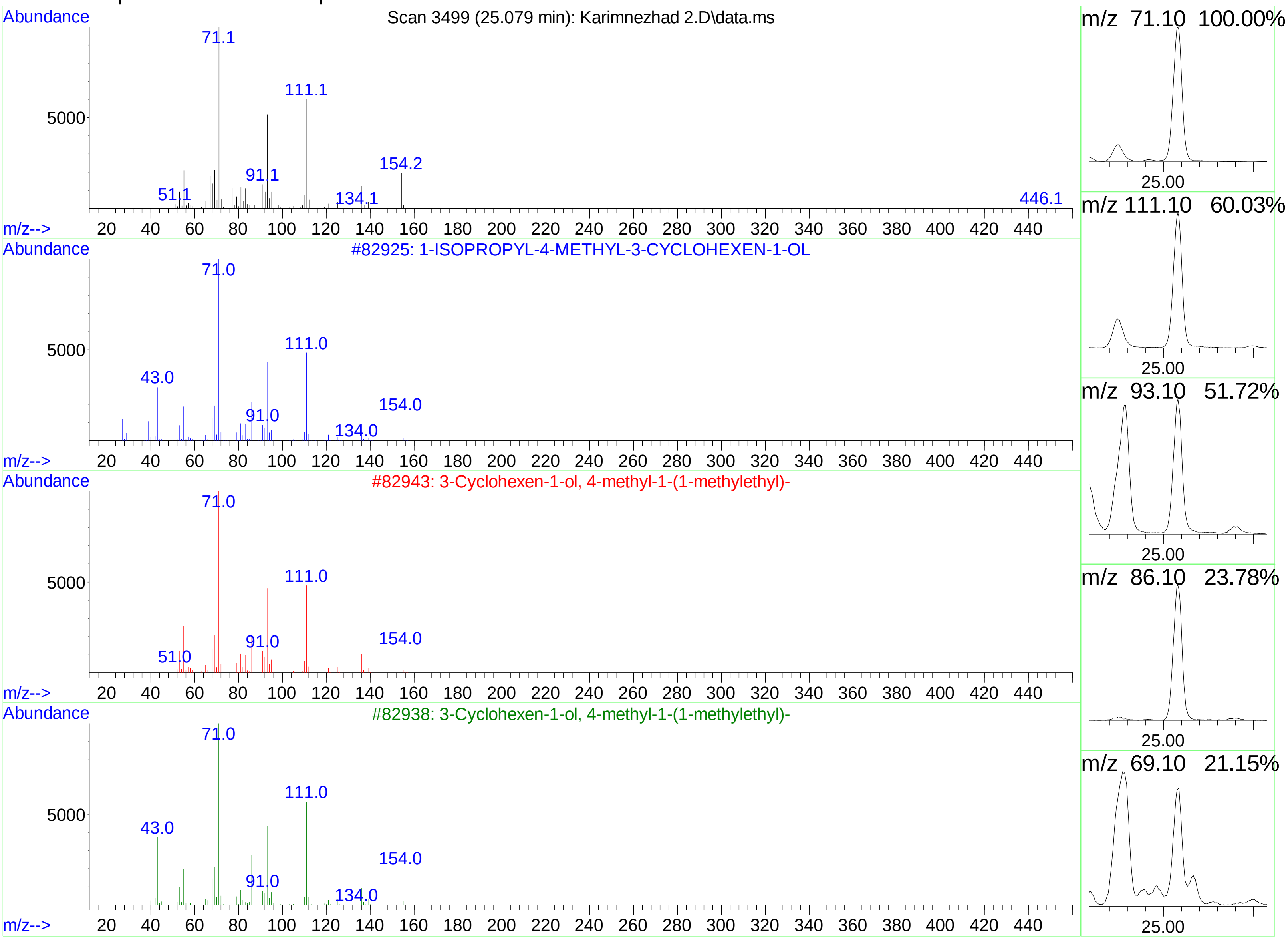

Data File: D:\msdchem\1\data\Karimnezhad 2.D
Sample : M10

Peak Number: 23 at 25.079 min Area: 73419391 Area % 0.61

The 3 best hits from each library. Ref\# CAS\# Qual

D:\Database\W10N14.L

1	1-ISOPROPYL-4-METHYL-3-CYCLOHEXE...	82925	000562-74-3	98
2	3-Cyclohexen-1-ol, 4-methyl-1-(1...	82943	000562-74-3	98
3	3-Cyclohexen-1-ol, 4-methyl-1-(1...	82938	000562-74-3	97

Unknown Spectrum based on Apex

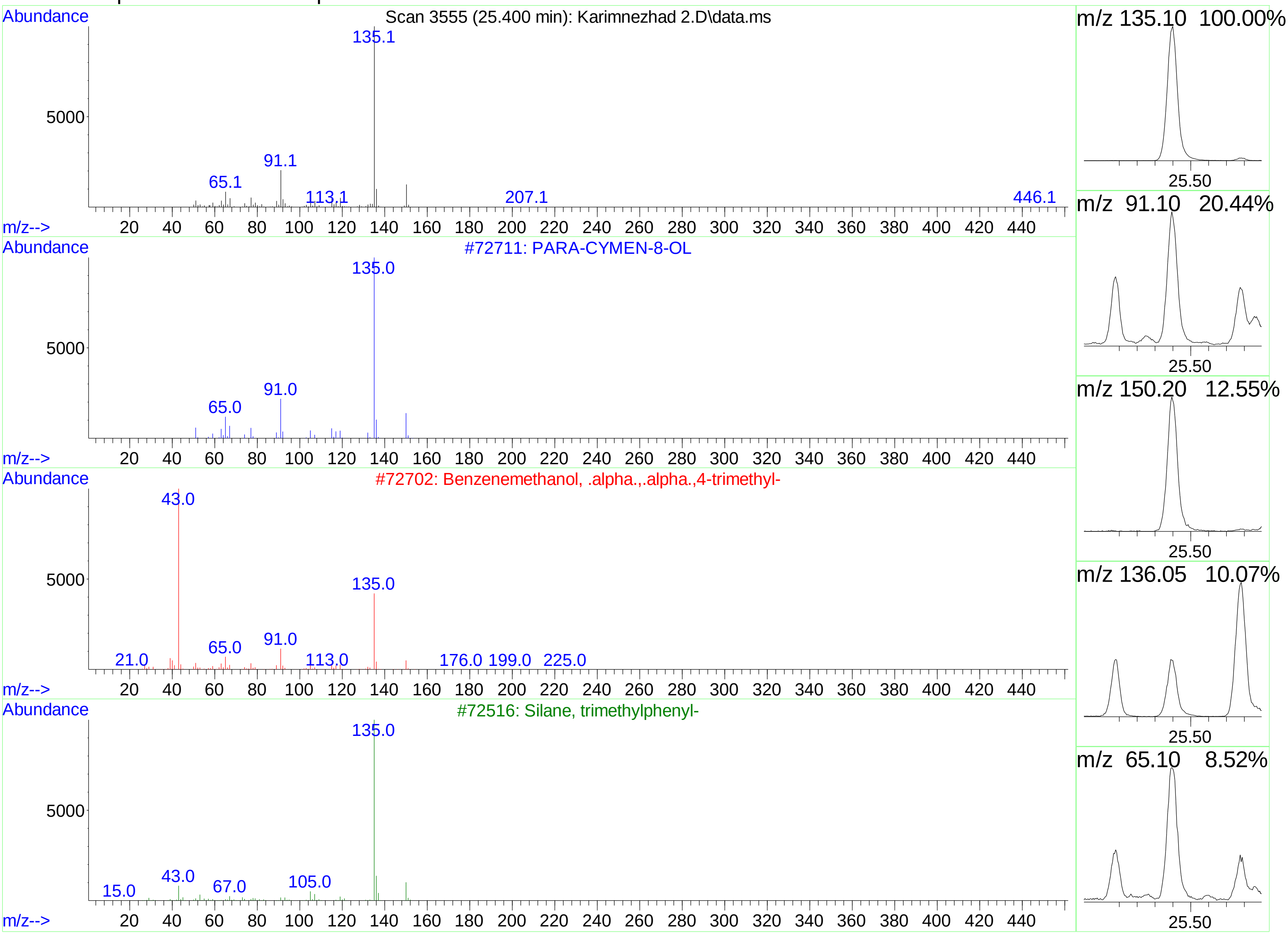

Data File: D:\msdchem\1\data\Karimnezhad 2.D
Sample : M10

Peak Number: 24 at 25.400 min Area: 46537460 Area % 0.39

The 3 best hits from each library. Ref\# CAS\# Qual

D:\Database\W10N14.L

1	PARA-CYMEN-8-OL	72711	001197-01-9	94
2	Benzenemethanol, .alpha.,.alpha....	72702	001197-01-9	91
3	Silane, trimethylphenyl-	72516	000768-32-1	80

Unknown Spectrum based on Apex

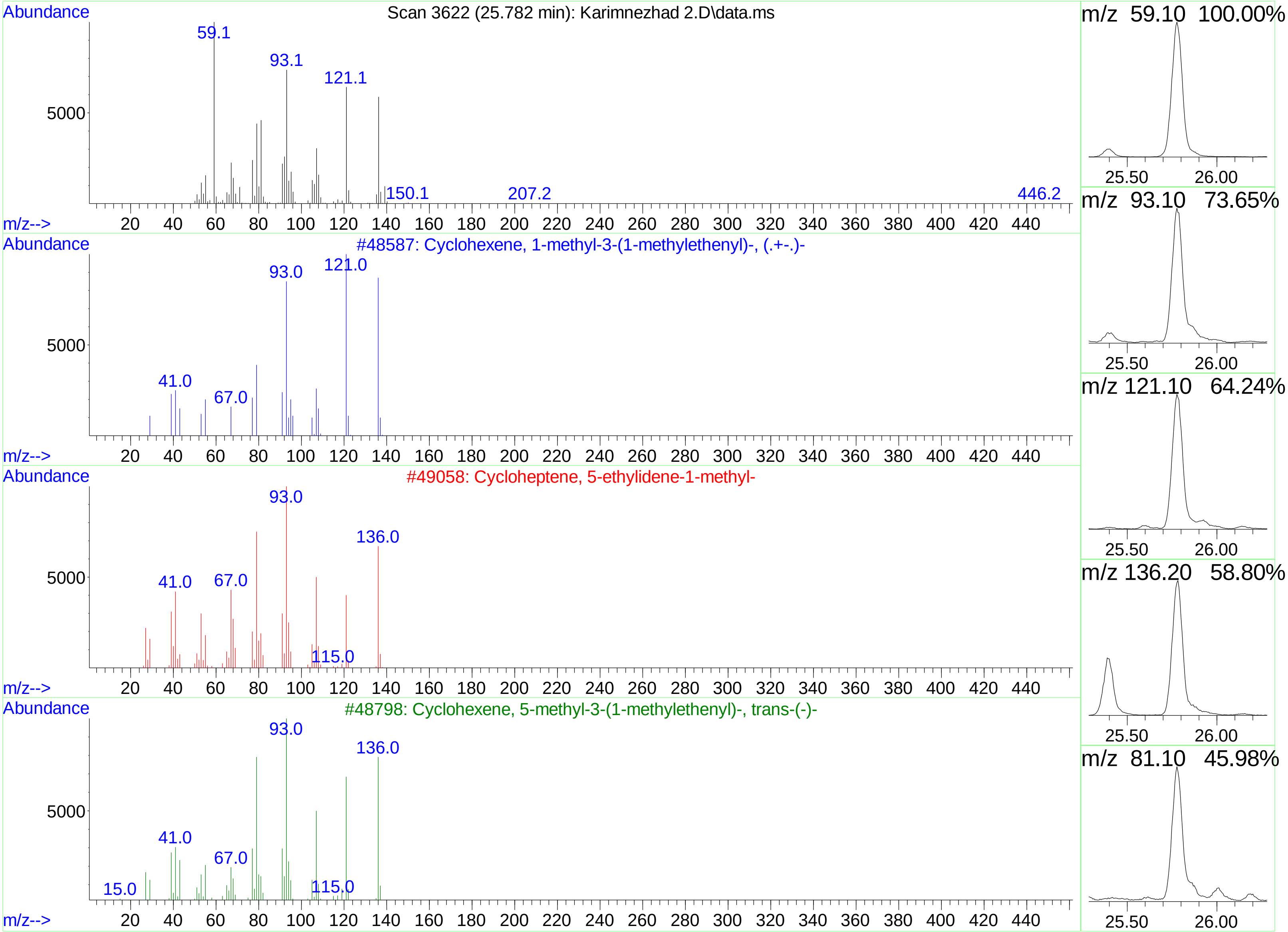

Data File: D:\msdchem\1\data\Karimnezhad 2.D
Sample : M10

Peak Number: 25 at 25.782 min Area: 62046715 Area % 0.52

The 3 best hits from each library. Ref\# CAS\# Qual

D:\Database\W10N14.L

1	Cyclohexene, 1-methyl-3-(1-methy...	48587	000499-03-6	89
2	Cycloheptene, 5-ethylidene-1-met...	49058	015402-94-5	60
3	Cyclohexene, 5-methyl-3-(1-methy...	48798	056816-08-1	60

Unknown Spectrum based on Apex

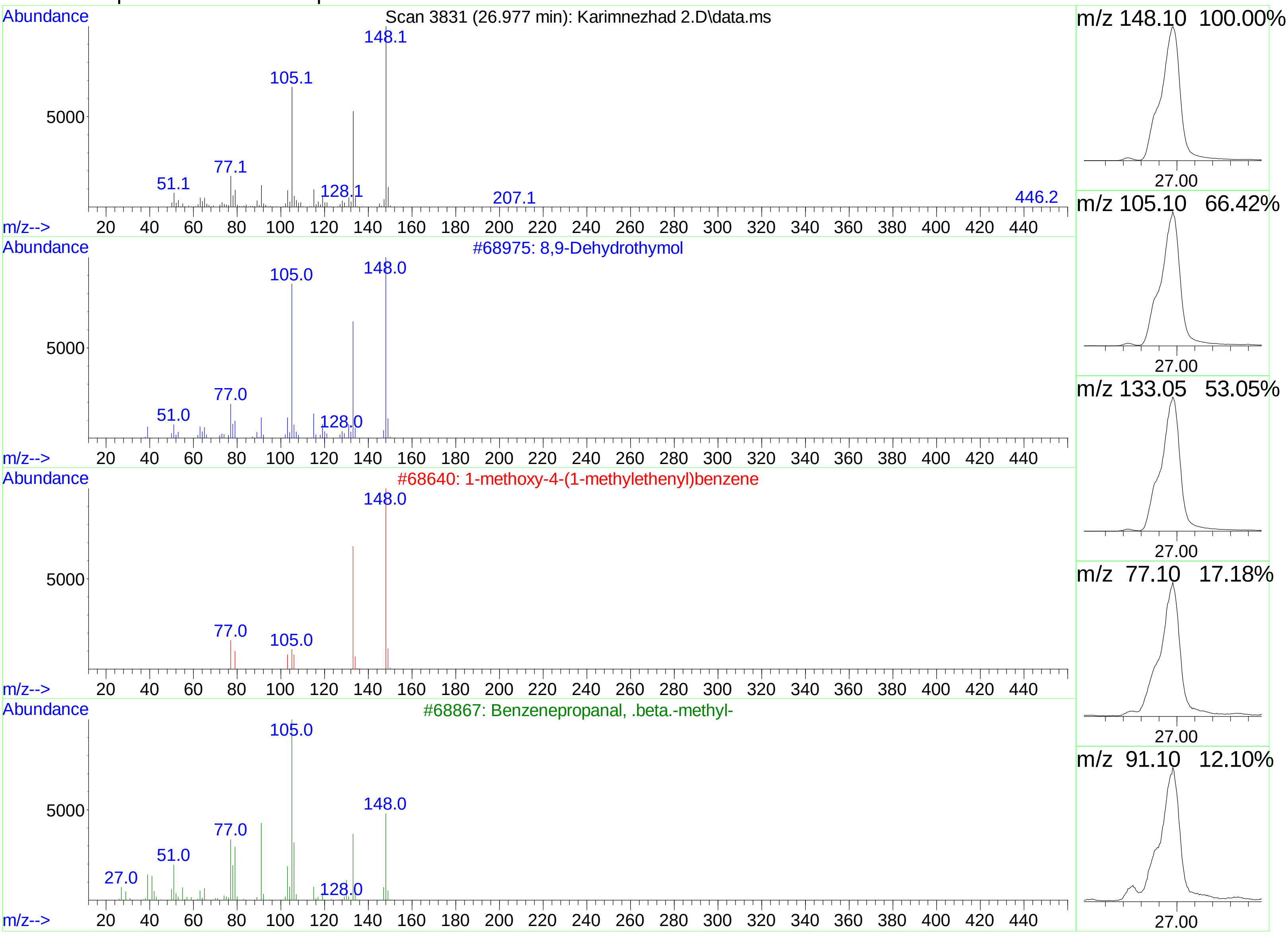

Data File: D:\msdchem\1\data\Karimnezhad 2.D
Sample : M10

Peak Number: 26 at 26.977 min Area: 400182687 Area % 3.35

The 3 best hits from each library. Ref\# CAS\# Qual

D:\Database\W10N14.L

1	8,9-Dehydrothymol	68975	018612-99-2	95
2	1-methoxy-4-(1-methylethenyl)ben...	68640	2000068-64-0	81
3	Benzenepropanal, .beta.-methyl-	68867	016251-77-7	80

Unknown Spectrum based on Apex

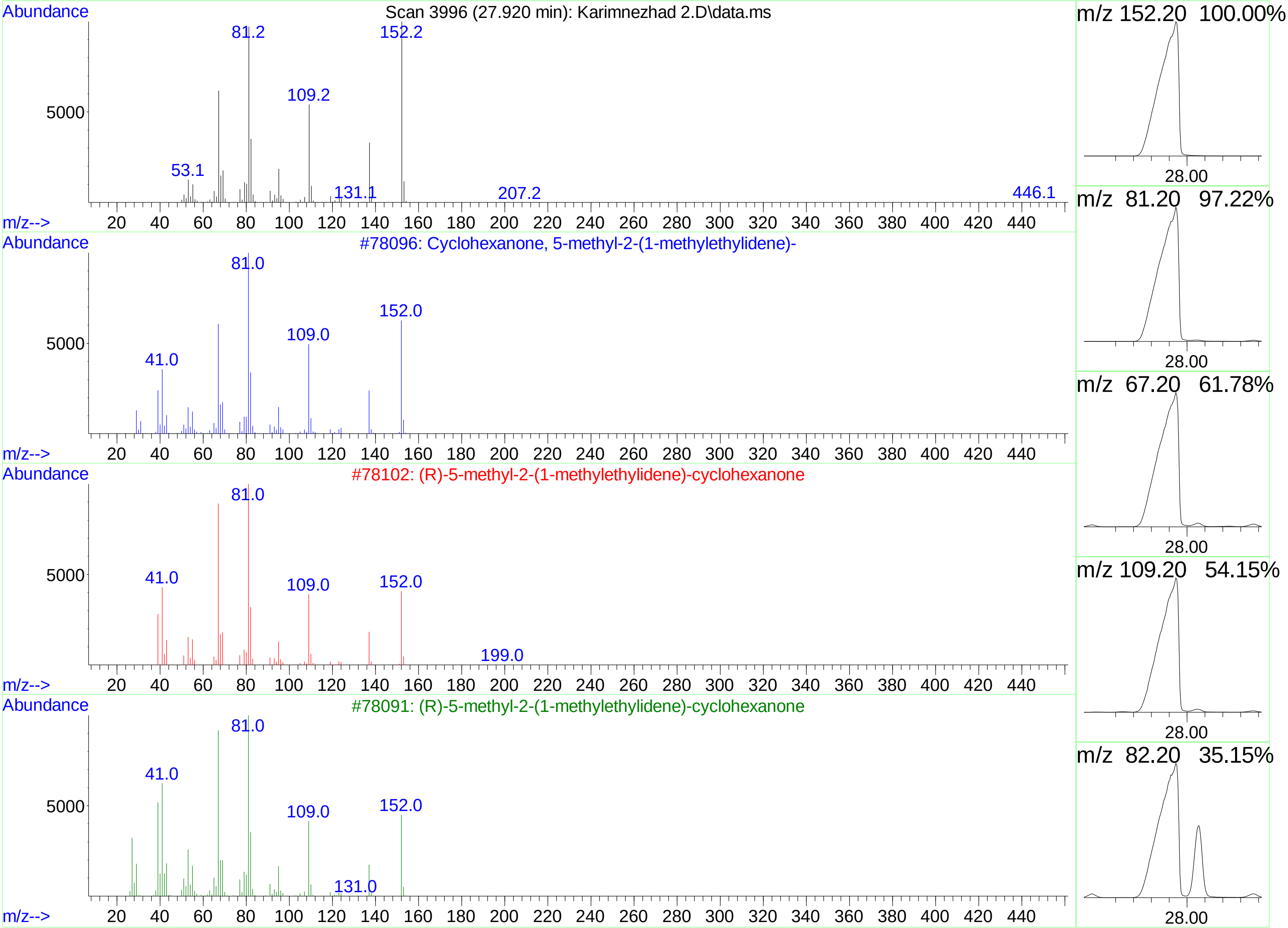

Data File: D:\msdchem\1\data\Karimnezhad 2.D
Sample : M10

Peak Number: 27 at 27.920 min Area: 1252175999 Area % 10.47

The 3 best hits from each library.	Ref#	CAS#	Qual
D:\Database\W10N14.L			
1 Cyclohexanone, 5-methyl-2-(1-met...	78096	015932-80-6	96
2 (R)-5-methyl-2-(1-methylethylide...	78102	000089-82-7	96
3 (R)-5-methyl-2-(1-methylethylide...	78091	000089-82-7	95

Unknown Spectrum based on Apex

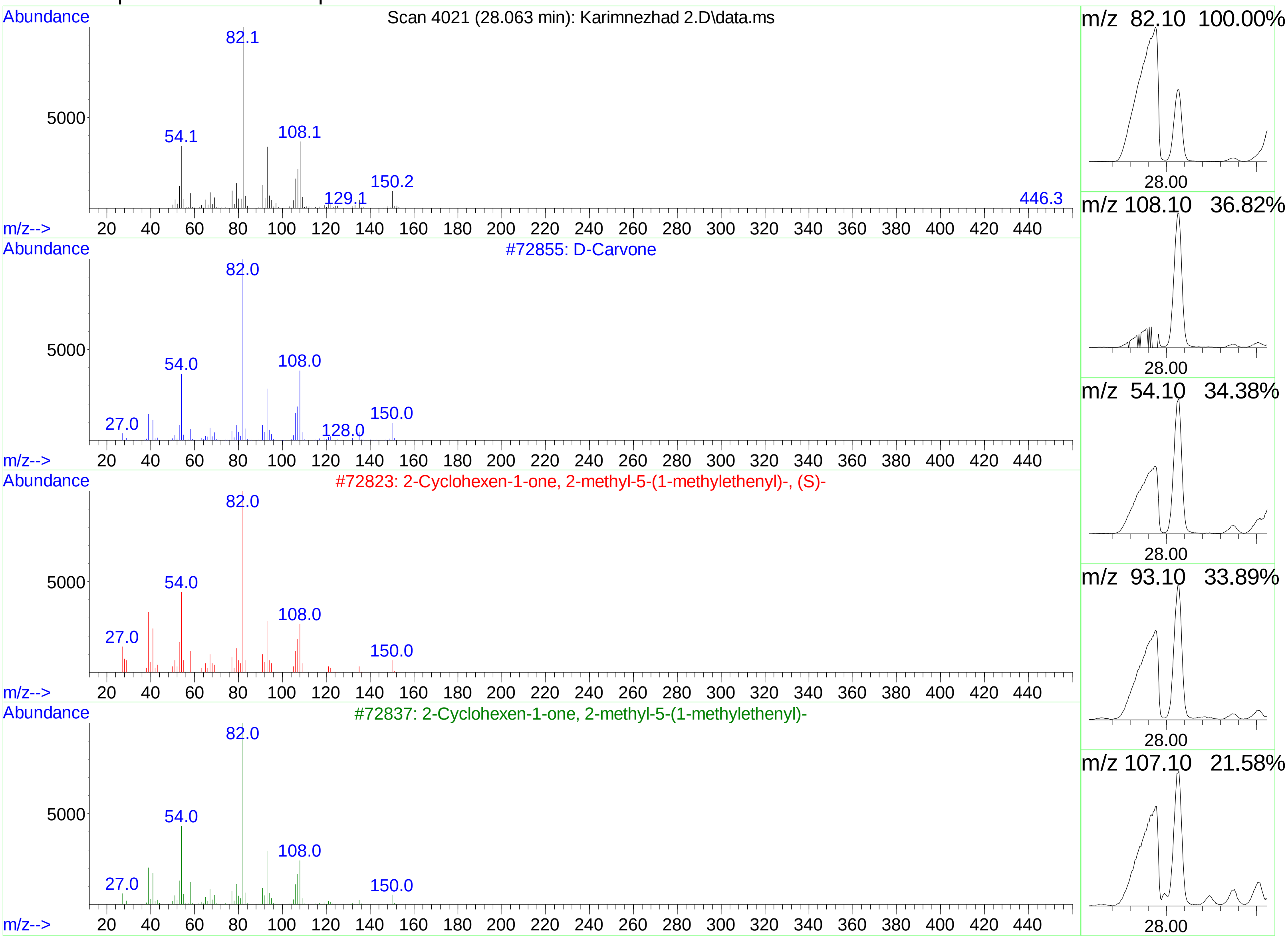

Data File: D:\msdchem\1\data\Karimnezhad 2.D
Sample : M10

Peak Number: 28 at 28.063 min Area: 78190238 Area % 0.65

The 3 best hits from each library. Ref\# CAS\# Qual

D:\Database\W10N14.L

1	D-Carvone	72855	002244-16-8	97
2	2-Cyclohexen-1-one, 2-methyl-5-(...	72823	002244-16-8	97
3	2-Cyclohexen-1-one, 2-methyl-5-(...	72837	000099-49-0	97

Unknown Spectrum based on Apex

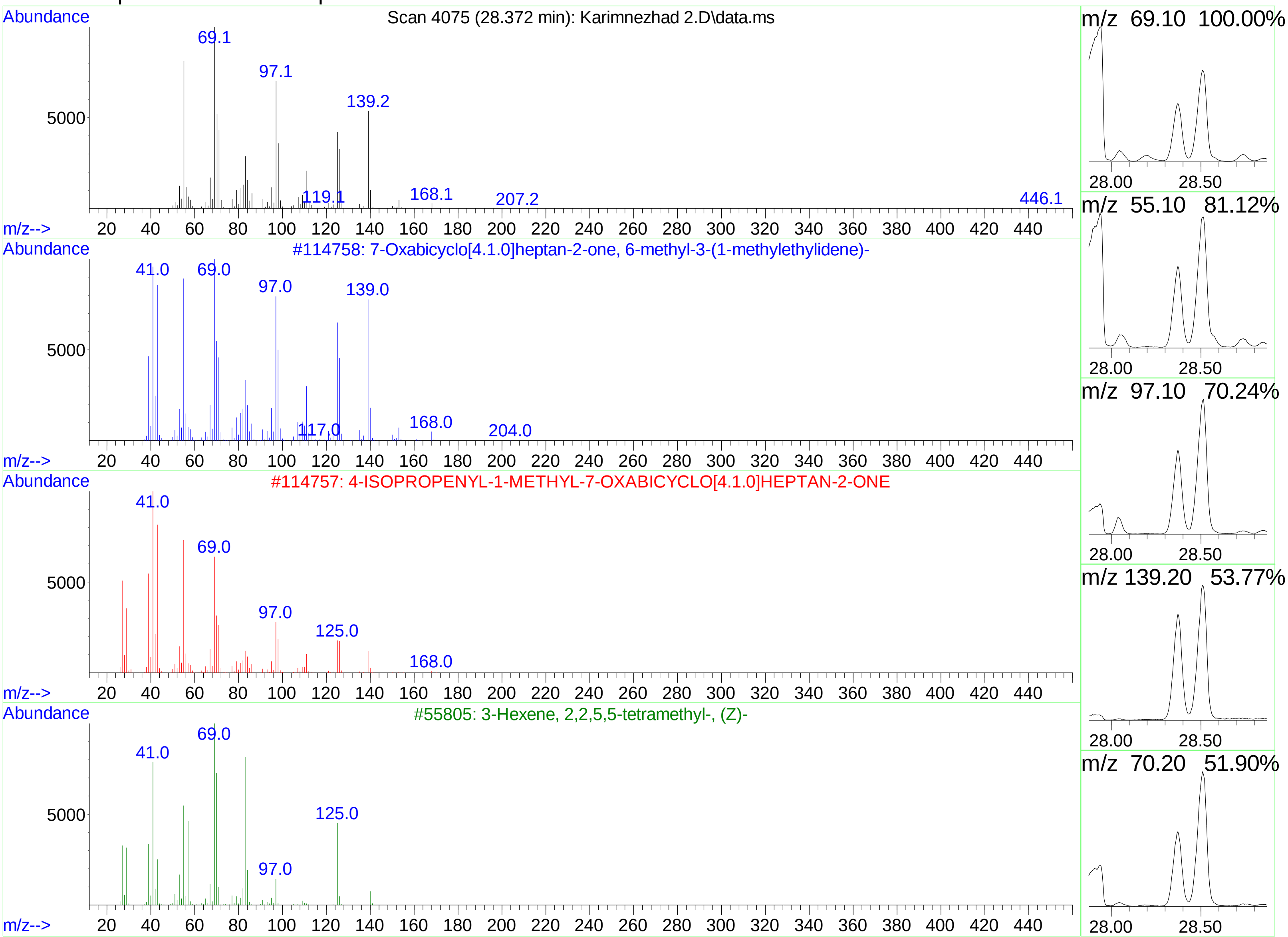

Data File: D:\msdchem\1\data\Karimnezhad 2.D
Sample : M10

Peak Number: 29 at 28.372 min Area: 63522022 Area % 0.53

The 3 best hits from each library. Ref\# CAS\# Qual

D:\Database\W10N14.L

	Name	Ref\#	CAS\#	Qual
1	7-Oxabicyclo[4.1.0]heptan-2-one,...	114758	035178-55-3	97
2	4-ISOPROPENYL-1-METHYL-7-OXABICY...	114757	035178-55-3	52
3	3-Hexene, 2,2,5,5-tetramethyl-, ...	55805	000692-47-7	46

Unknown Spectrum based on Apex

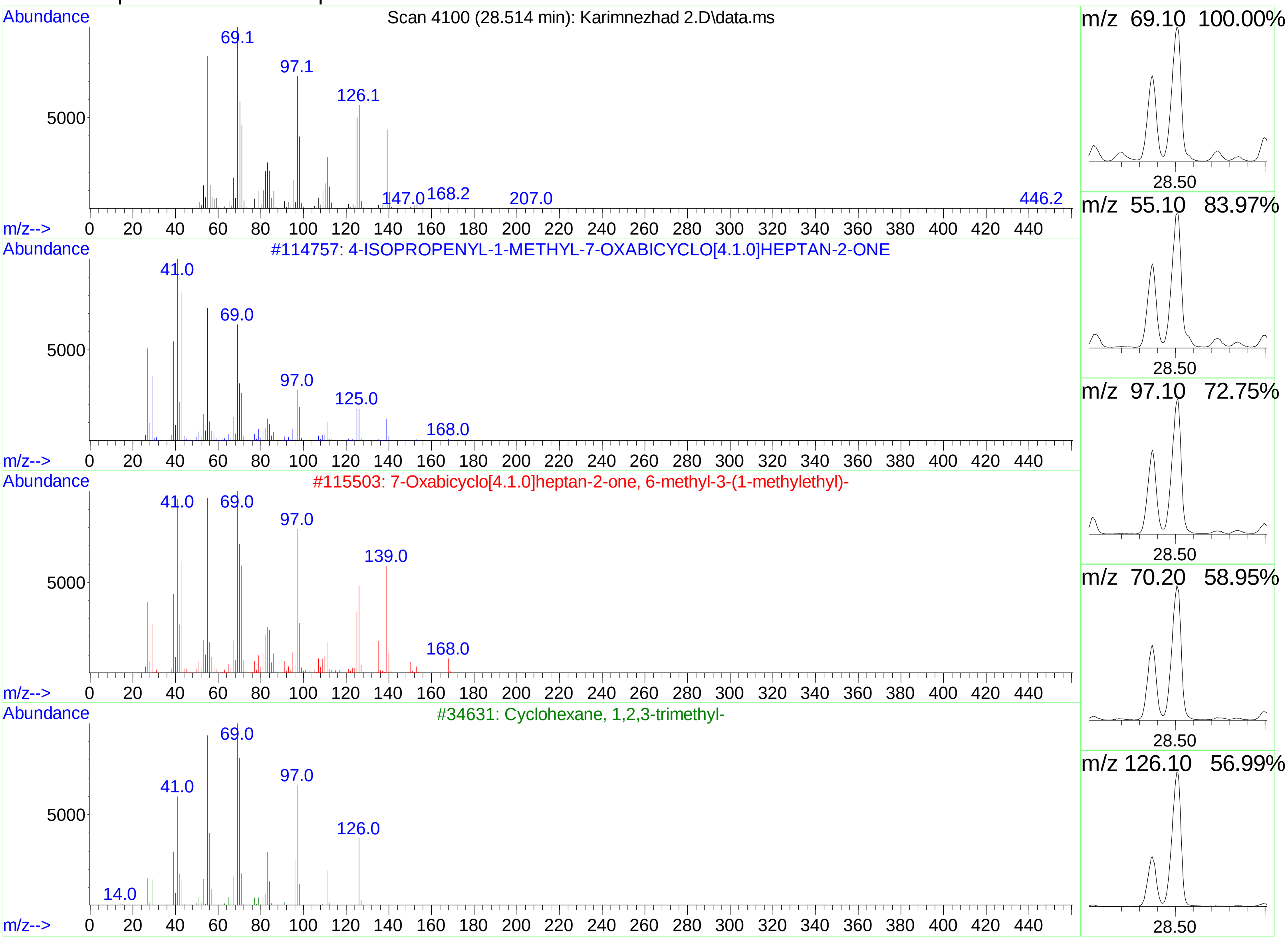

Data File: D:\msdchem\1\data\Karimnezhad 2.D
Sample : M10

Peak Number: 30 at 28.514 min Area: 140167969 Area % 1.17

The 3 best hits from each library. Ref\# CAS\# Qual

D:\Database\W10N14.L

1	4-ISOPROPENYL-1-METHYL-7-OXABICY...	114757	035178-55-3	93
2	7-Oxabicyclo[4.1.0]heptan-2-one,...	115503	005286-38-4	87
3	Cyclohexane, 1,2,3-trimethyl-	34631	001678-97-3	60

Unknown Spectrum based on Apex

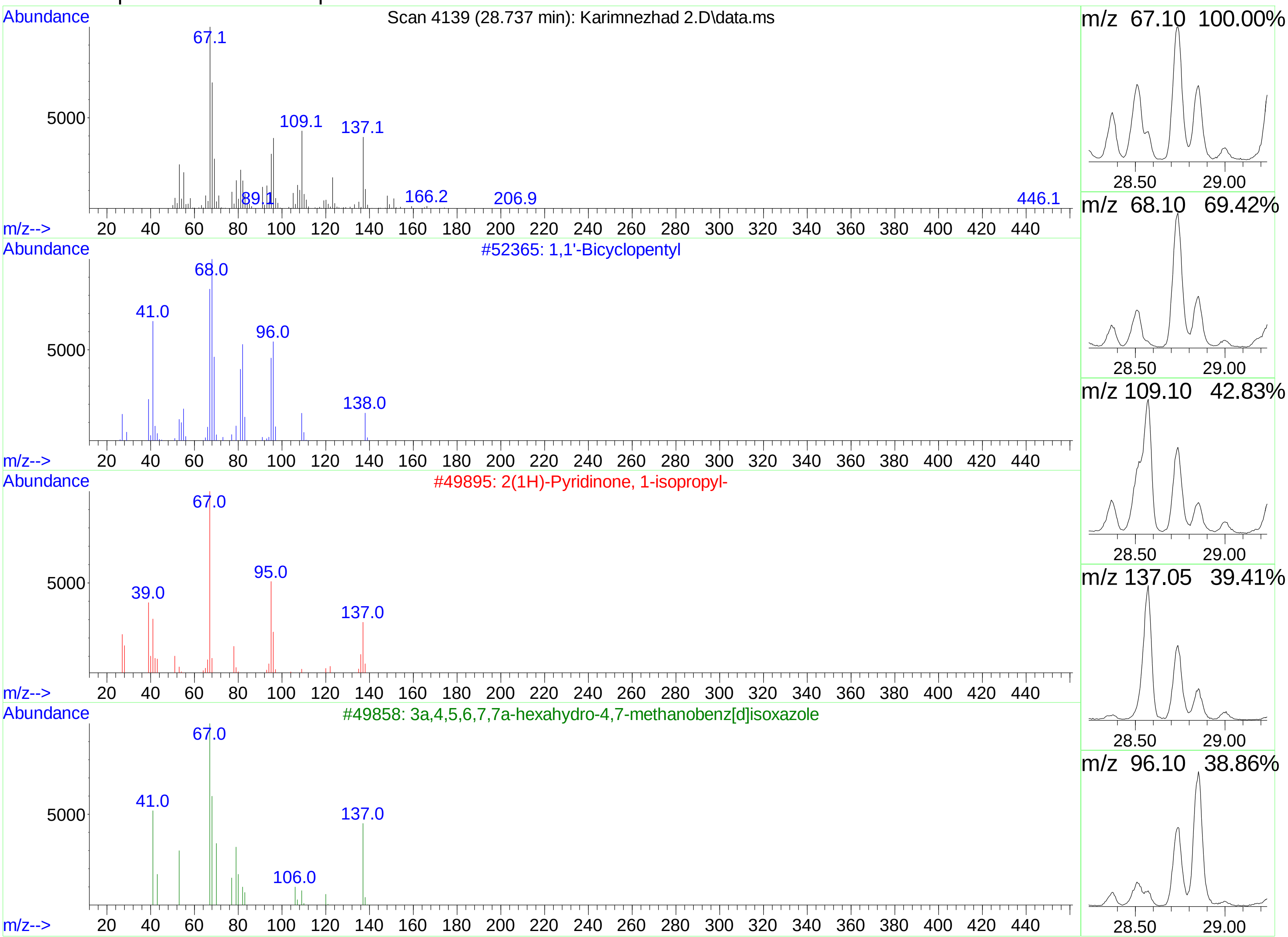

Data File: D:\msdchem\1\data\Karimnezhad 2.D
Sample : M10

Peak Number: 31 at 28.737 min Area: 24282538 Area % 0.20

The 3 best hits from each library. Ref\# CAS\# Qual

D:\Database\W10N14.L

1	1,1'-Bicyclopentyl	52365	001636-39-1	50
2	2(1H)-Pyridinone, 1-isopropyl-	49895	022973-00-8	49
3	3a,4,5,6,7,7a-hexahydro-4,7-meth...	49858	015166-80-0	46

Unknown Spectrum based on Apex

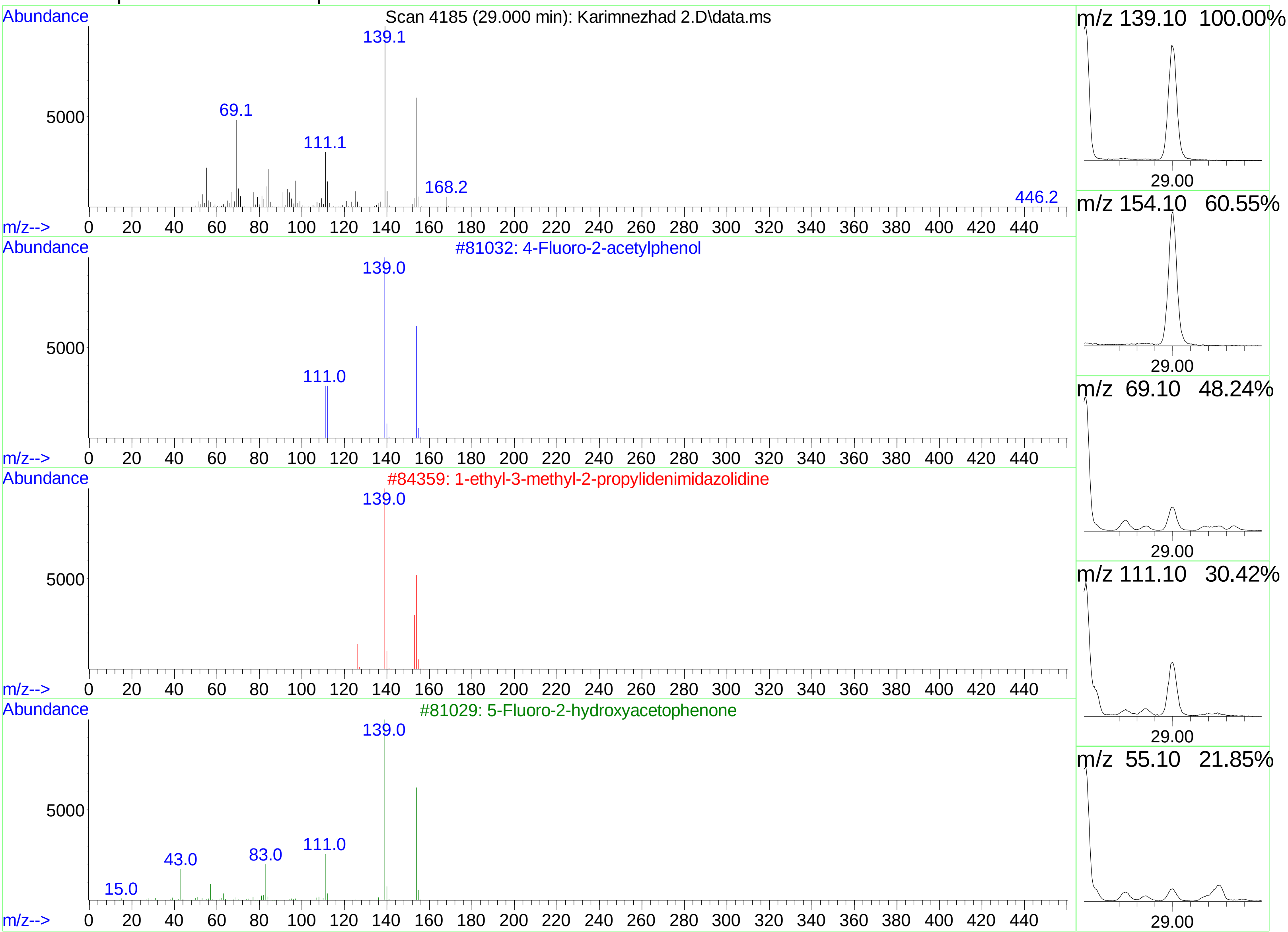

Data File: D:\msdchem\1\data\Karimnezhad 2.D
Sample : M10

Peak Number: 32 at 29.000 min Area: 21535302 Area % 0.18

The 3 best hits from each library. Ref\# CAS\# Qual

D:\Database\W10N14.L

1	4-Fluoro-2-acetylphenol	81032	000394-32-1	80
2	1-ethyl-3-methyl-2-propylidenimi...	84359	109153-29-9	78
3	5-Fluoro-2-hydroxyacetophenone	81029	000394-32-1	68

Unknown Spectrum based on Apex

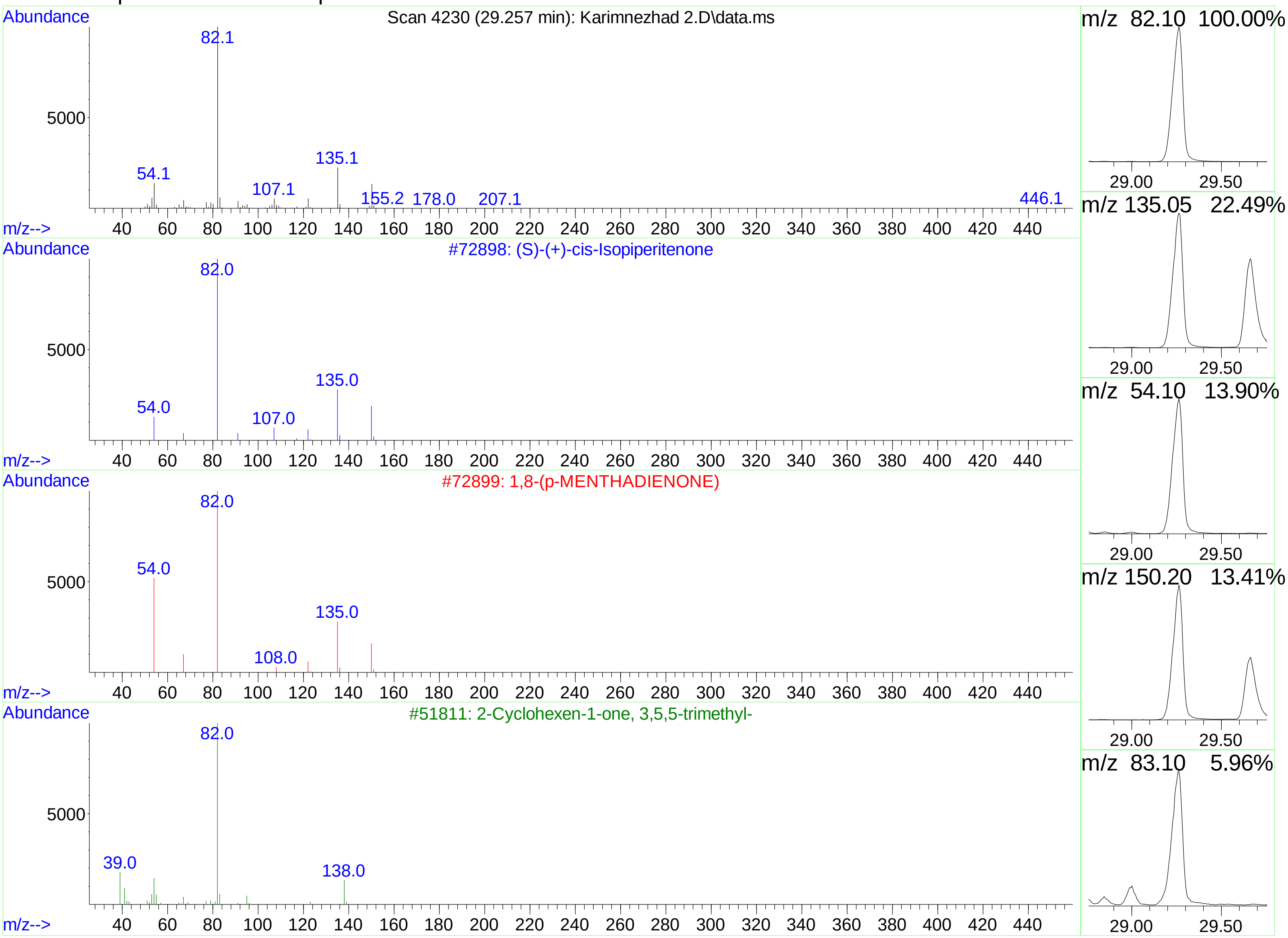

Data File: D:\msdchem\1\data\Karimnezhad 2.D
Sample : M10

Peak Number: 33 at 29.257 min Area: 155000849 Area % 1.30

The 3 best hits from each library.	Ref\#	CAS\#	Qual
D:\Database\W10N14.L			
1 (S)-(+)-cis-Isopiperitenone	72898	2000072-89-8	91
2 1,8-(p-MENTHADIENONE)	72899	2000072-89-9	72
3 2-Cyclohexen-1-one, 3,5,5-trimet...	51811	000078-59-1	50

Unknown Spectrum based on Apex

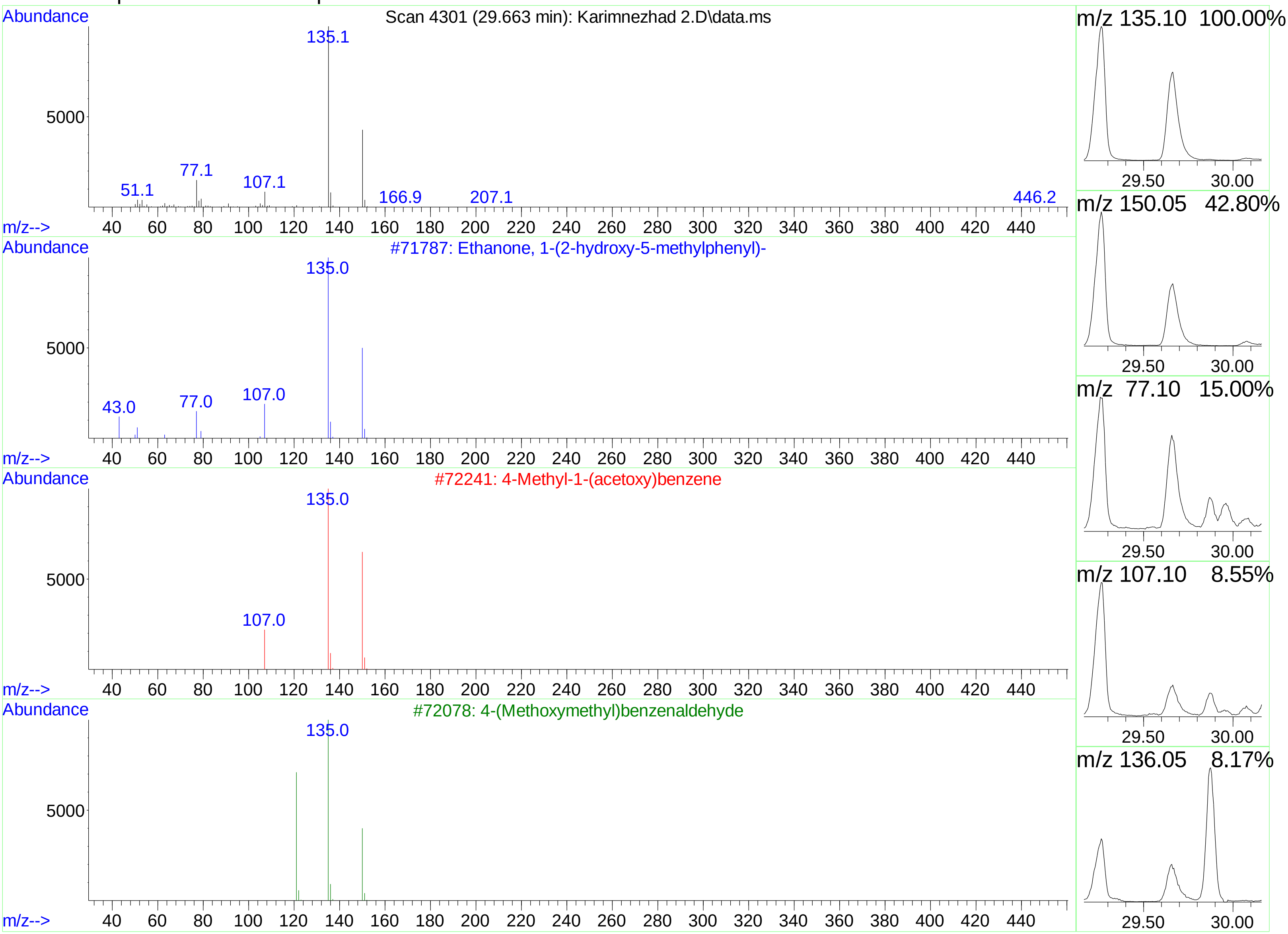

Data File: D:\msdchem\1\data\Karimnezhad 2.D
Sample : M10

Peak Number: 34 at 29.663 min Area: 22252158 Area % 0.19

The 3 best hits from each library. Ref\# CAS\# Qual

D:\Database\W10N14.L

1	Ethanone, 1-(2-hydroxy-5-methylp...	71787	001450-72-2	90
2	4-Methyl-1-(acetoxy)benzene	72241	000140-39-6	90
3	4-(Methoxymethyl)benzenaldehyde	72078	2000072-07-8	90

Unknown Spectrum based on Apex

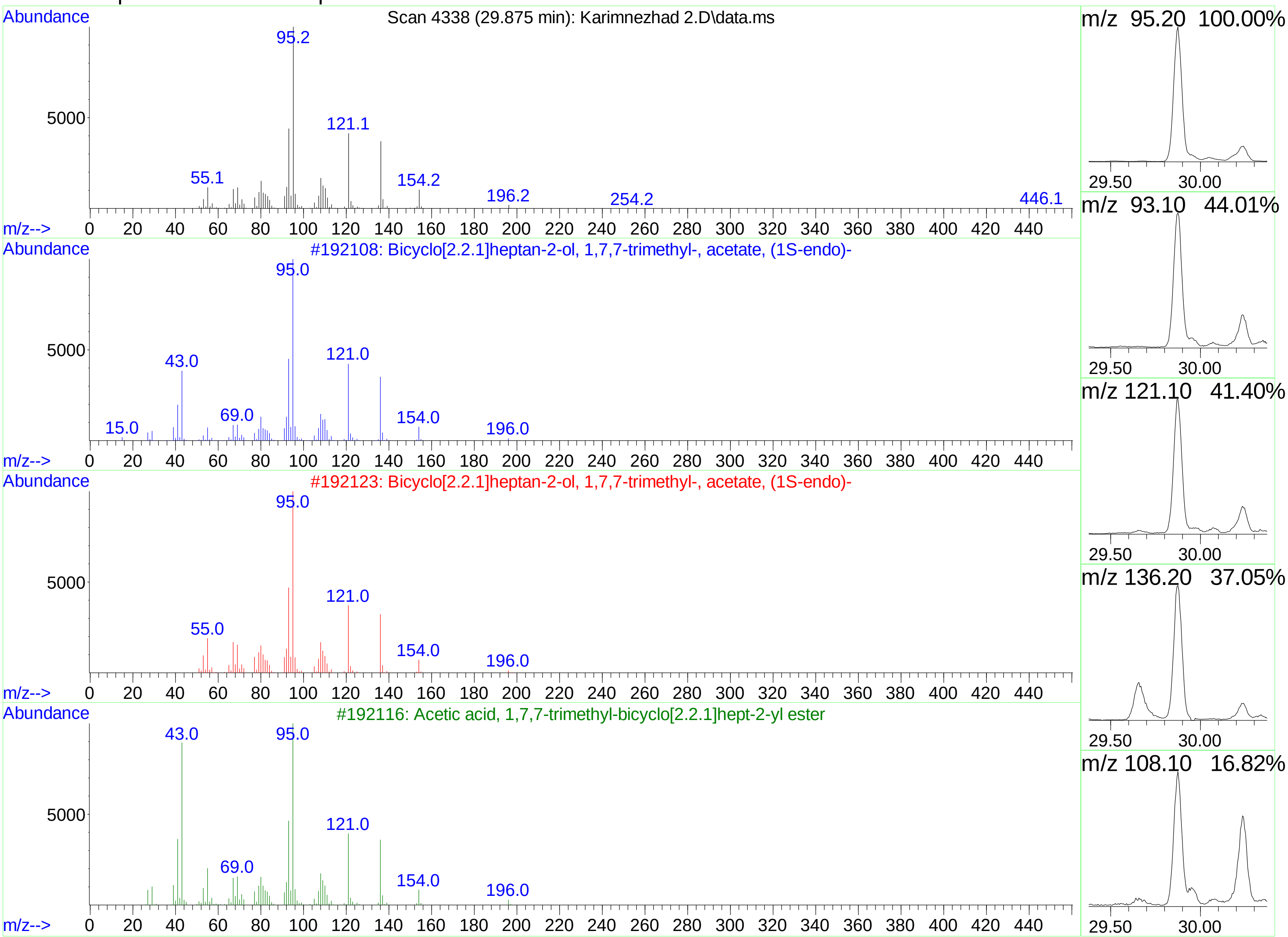

Data File: D:\msdchem\1\data\Karimnezhad 2.D
Sample : M10

Peak Number: 35 at 29.875 min Area: 60300580 Area % 0.50

The 3 best hits from each library.	Ref#	CAS#	Qual
D:\Database\W10N14.L			
1 Bicyclo[2.2.1]heptan-2-ol, 1,7,7...	192108	005655-61-8	99
2 Bicyclo[2.2.1]heptan-2-ol, 1,7,7...	192123	005655-61-8	99
3 Acetic acid, 1,7,7-trimethyl-bic...	192116	092618-89-8	98

Unknown Spectrum based on Apex

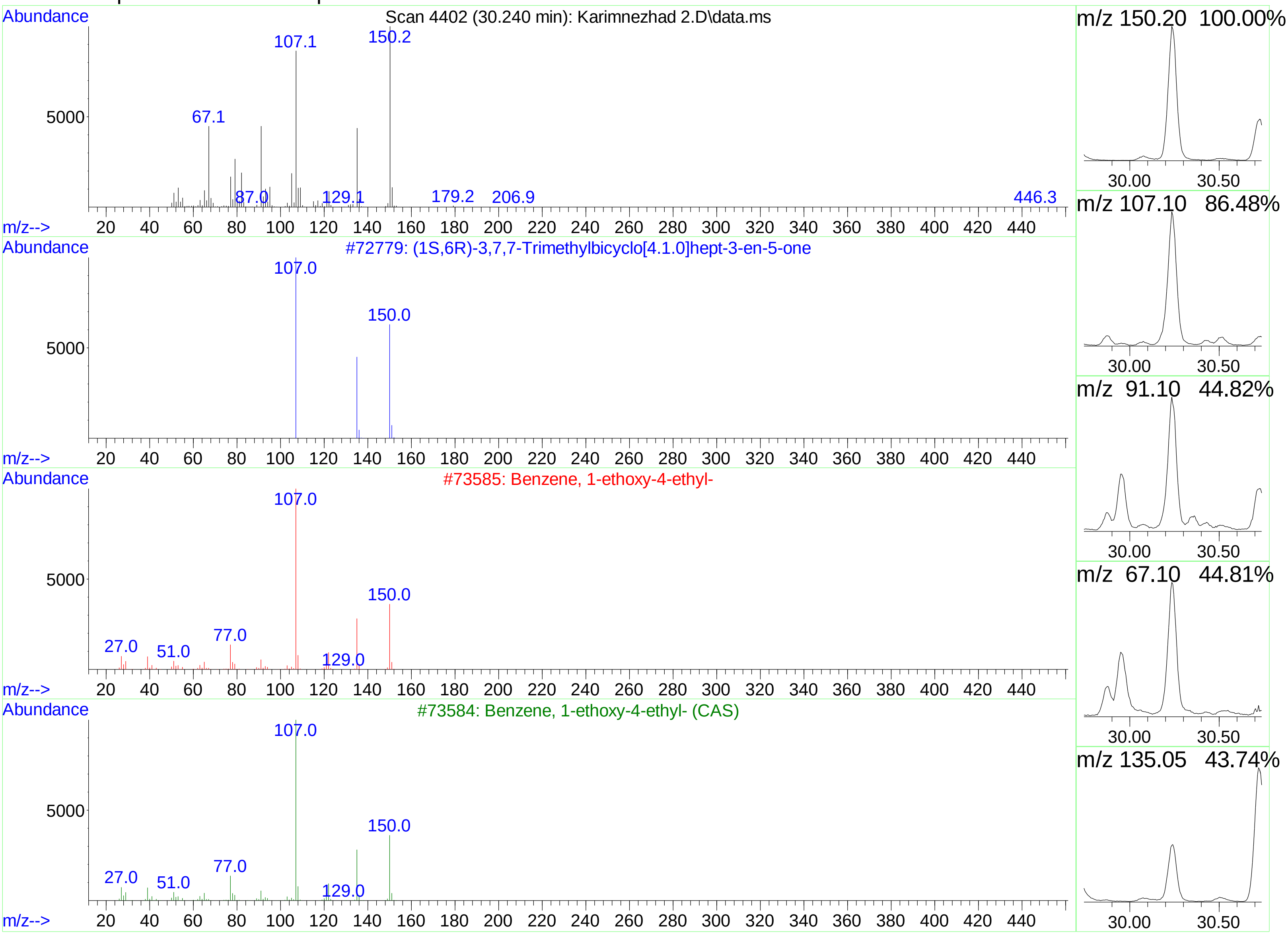

Data File: D:\msdchem\1\data\Karimnezhad 2.D
Sample : M10

Peak Number: 36 at 30.240 min Area: 56561967 Area % 0.47

The 3 best hits from each library.	Ref\#	CAS\#	Qual
D:\Database\W10N14.L			
1 (1S,6R)-3,7,7-Trimethylbicyclo[4...	72779	2000072-77-9	86
2 Benzene, 1-ethoxy-4-ethyl-	73585	001585-06-4	83
3 Benzene, 1-ethoxy-4-ethyl- (CAS)	73584	001585-06-4	83

Unknown Spectrum based on Apex

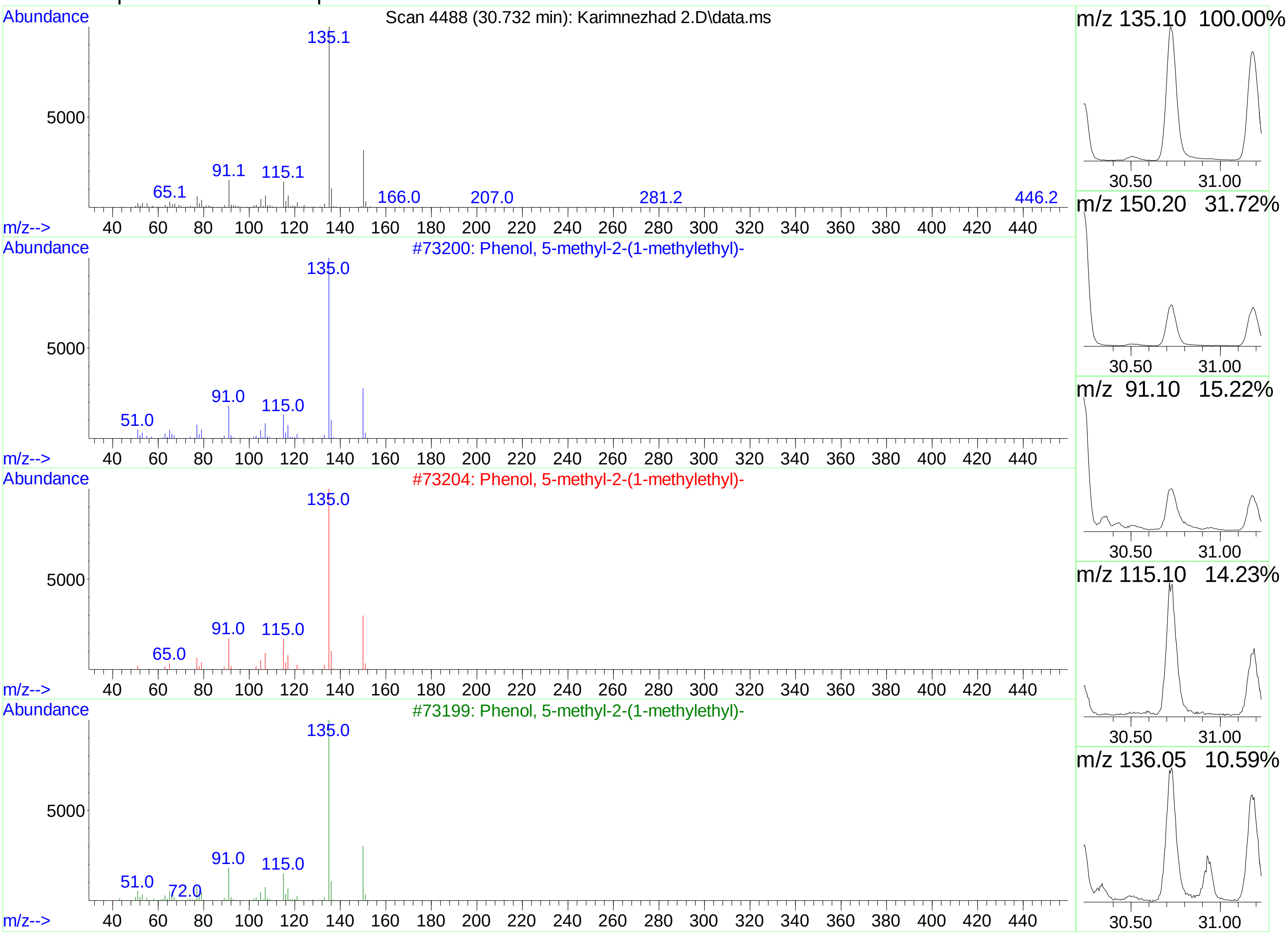

Data File: D:\msdchem\1\data\Karimnezhad 2.D
Sample : M10

Peak Number: 37 at 30.732 min Area: 48062896 Area % 0.40

The 3 best hits from each library.	Ref\#	CAS\#	Qual
D:\Database\W10N14.L			
1 Phenol, 5-methyl-2-(1-methylethyl)-	73200	000089-83-8	95
2 Phenol, 5-methyl-2-(1-methylethyl)-	73204	000089-83-8	95
3 Phenol, 5-methyl-2-(1-methylethyl)-	73199	000089-83-8	95

Unknown Spectrum based on Apex

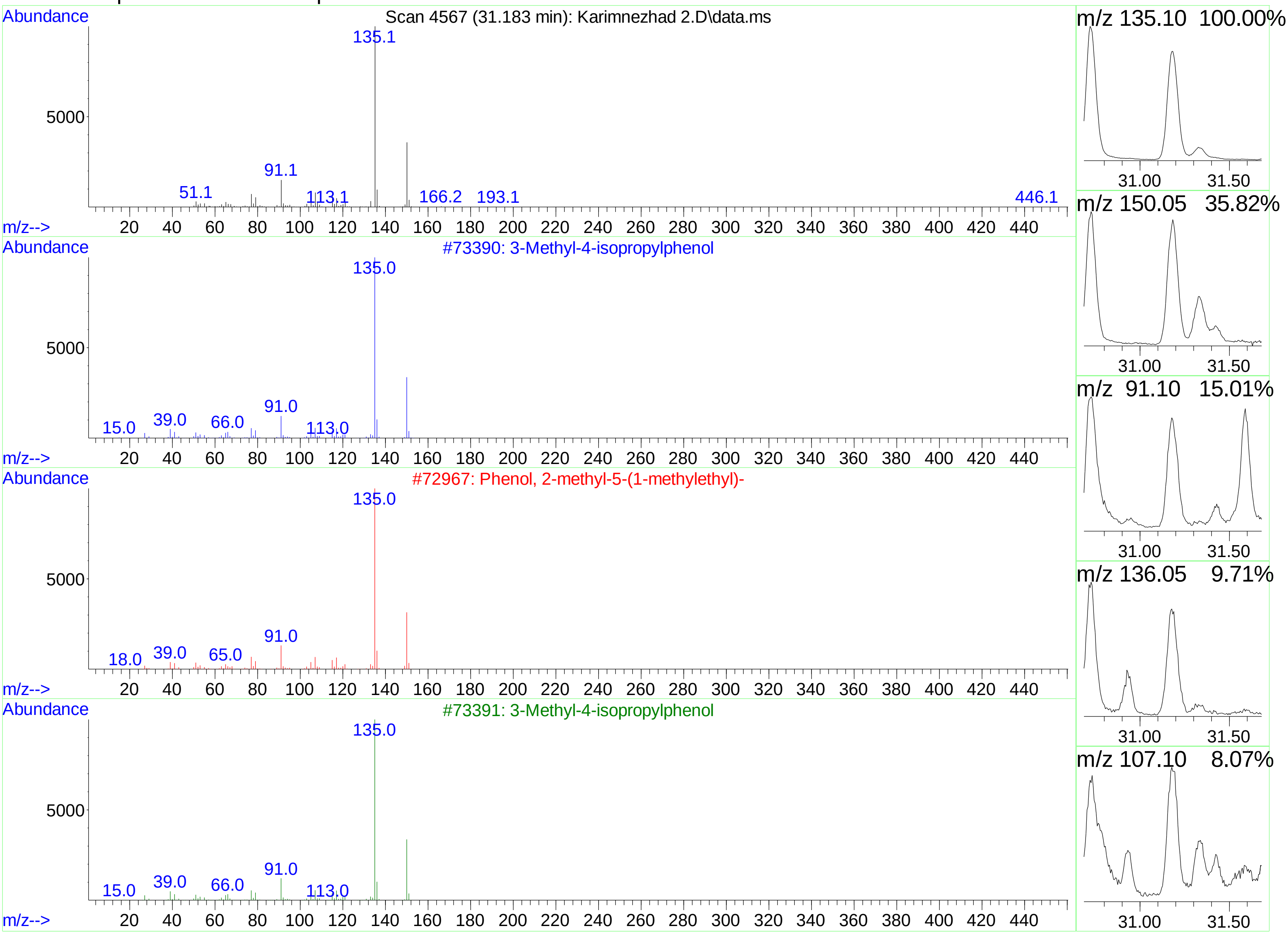

Data File: D:\msdchem\1\data\Karimnezhad 2.D
Sample : M10

Peak Number: 38 at 31.183 min Area: 19975570 Area % 0.17

The 3 best hits from each library.	Ref\#	CAS\#	Qual
D:\Database\W10N14.L			
1 3-Methyl-4-isopropylphenol	73390	003228-02-2	94
2 Phenol, 2-methyl-5-(1-methylethyl)-	72967	000499-75-2	94
3 3-Methyl-4-isopropylphenol	73391	003228-02-2	94

Unknown Spectrum based on Apex

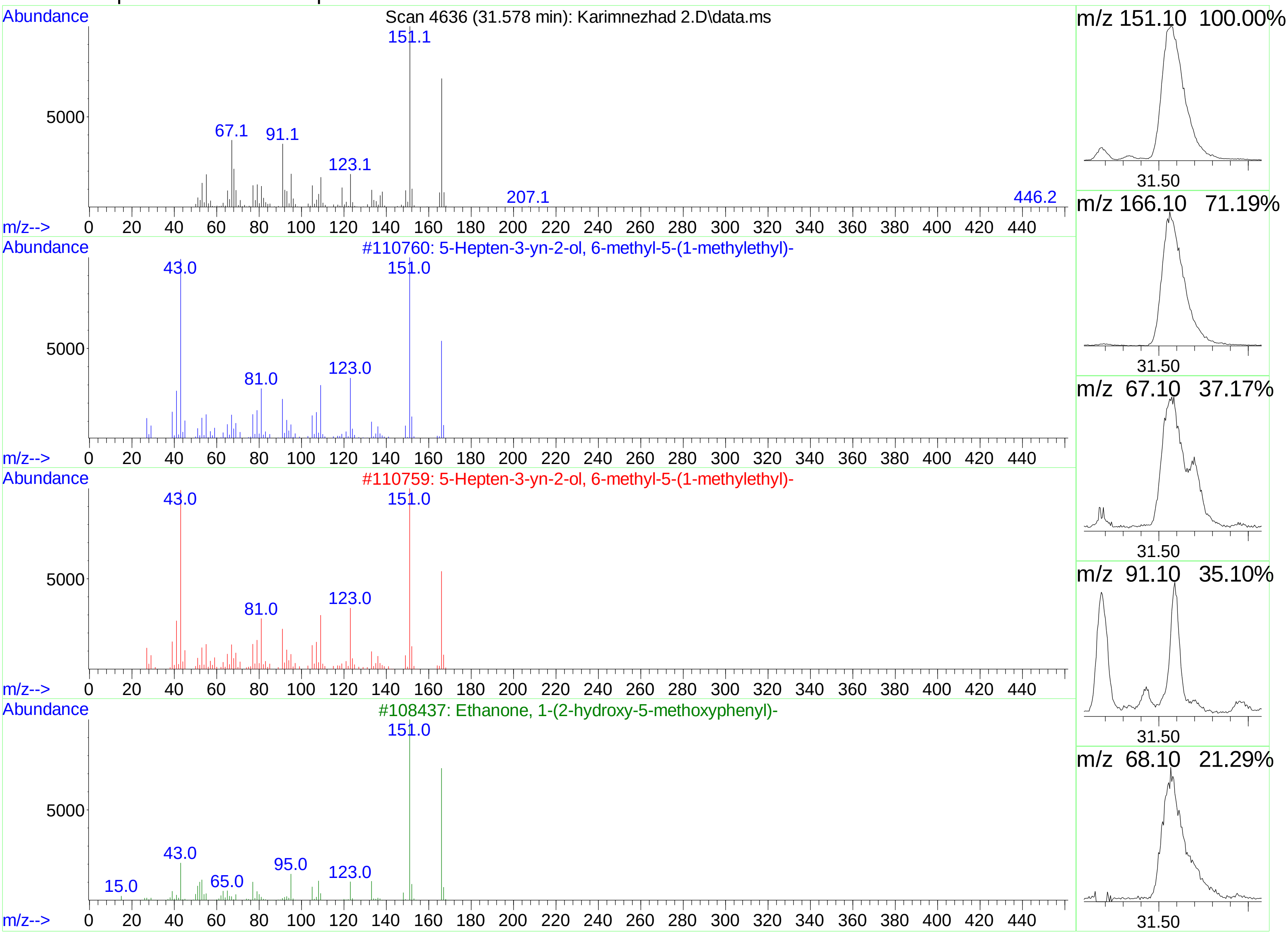

Data File: D:\msdchem\1\data\Karimnezhad 2.D
Sample : M10

Peak Number: 39 at 31.578 min Area: 52097189 Area % 0.44

The 3 best hits from each library.	Ref\#	CAS\#	Qual
D:\Database\W10N14.L			
1 5-Hepten-3-yn-2-ol, 6-methyl-5-(...	110760	063922-41-8	87
2 5-Hepten-3-yn-2-ol, 6-methyl-5-(...	110759	063922-41-8	87
3 Ethanone, 1-(2-hydroxy-5-methoxy...	108437	000705-15-7	81

Library Search Report - ChemStation Integrator

Unknown Spectrum based on Apex

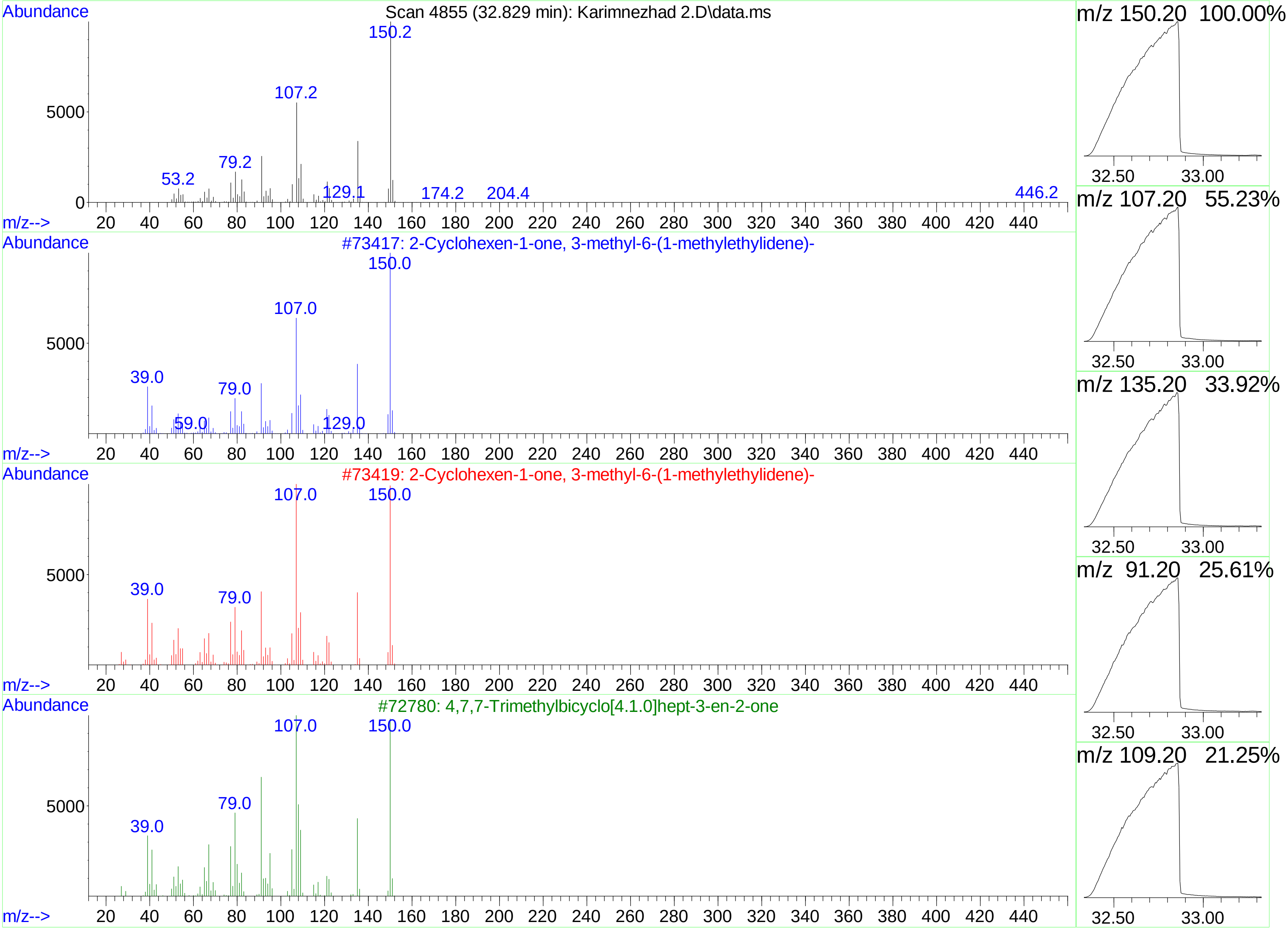

Data File: D:\msdchem\1\data\Karimnezhad 2.D
Sample : M10

Peak Number: 40 at 32.829 min Area: 4515274199 Area % 37.77

The 3 best hits from each library.	Ref#	CAS#	Qual
D:\Database\W10N14.L			
1 2-Cyclohexen-1-one, 3-methyl-6-(...	73417	000491-09-8	98
2 2-Cyclohexen-1-one, 3-methyl-6-(...	73419	000491-09-8	97
3 4,7,7-Trimethylbicyclo[4.1.0]hep...	72780	081800-50-2	93

Unknown Spectrum based on Apex

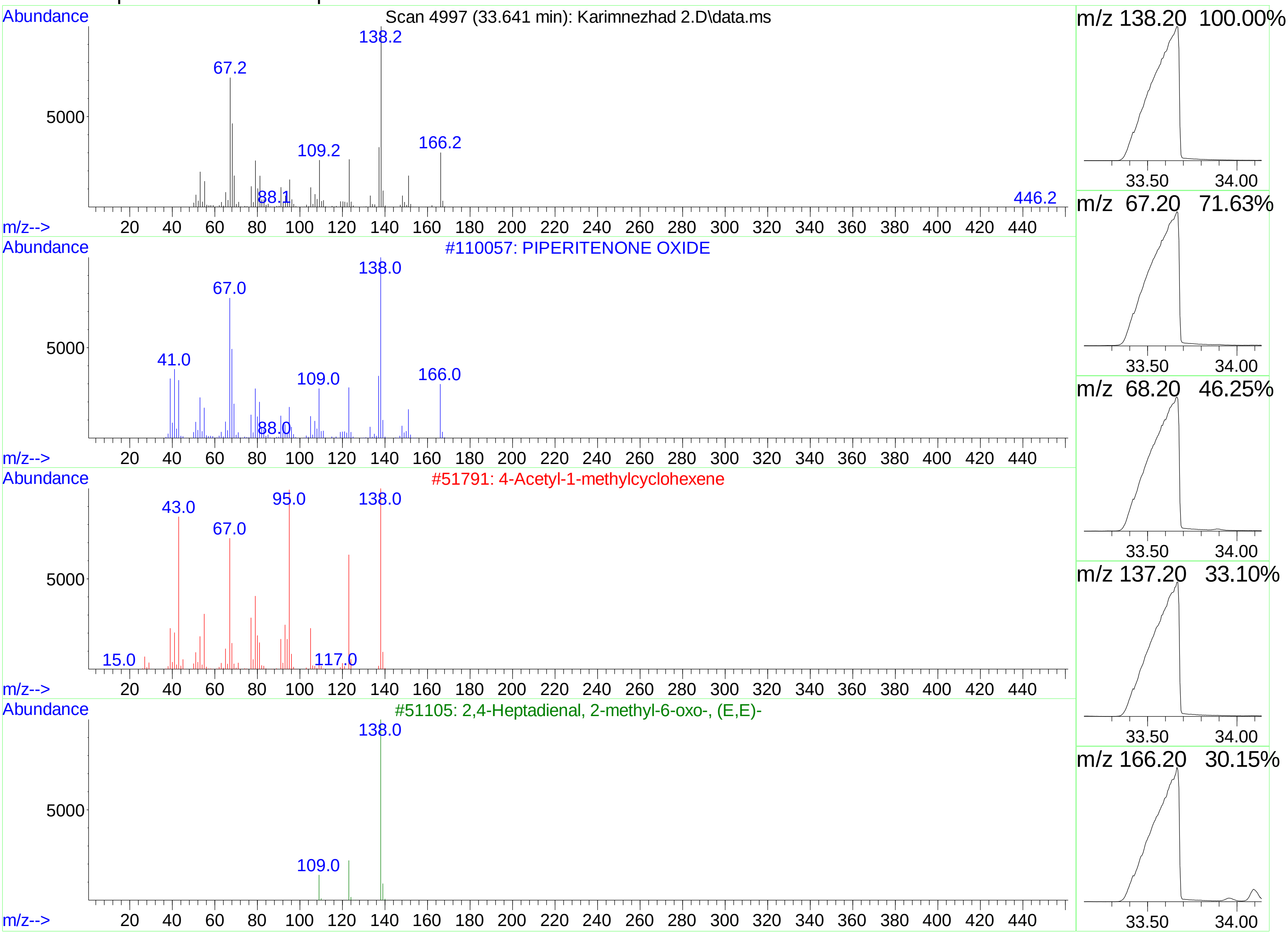

Data File: D:\msdchem\1\data\Karimnezhad 2.D
Sample : M10

Peak Number: 41 at 33.641 min Area: 1702627424 Area % 14.24

The 3 best hits from each library.	Ref\#	CAS\#	Qual
D:\Database\W10N14.L			
1 PIPERITENONE OXIDE	110057	003564-96-3	98
2 4-Acetyl-1-methylcyclohexene	51791	006090-09-1	60
3 2,4-Heptadienal, 2-methyl-6-oxo-...	51105	129454-99-5	58

Unknown Spectrum based on Apex

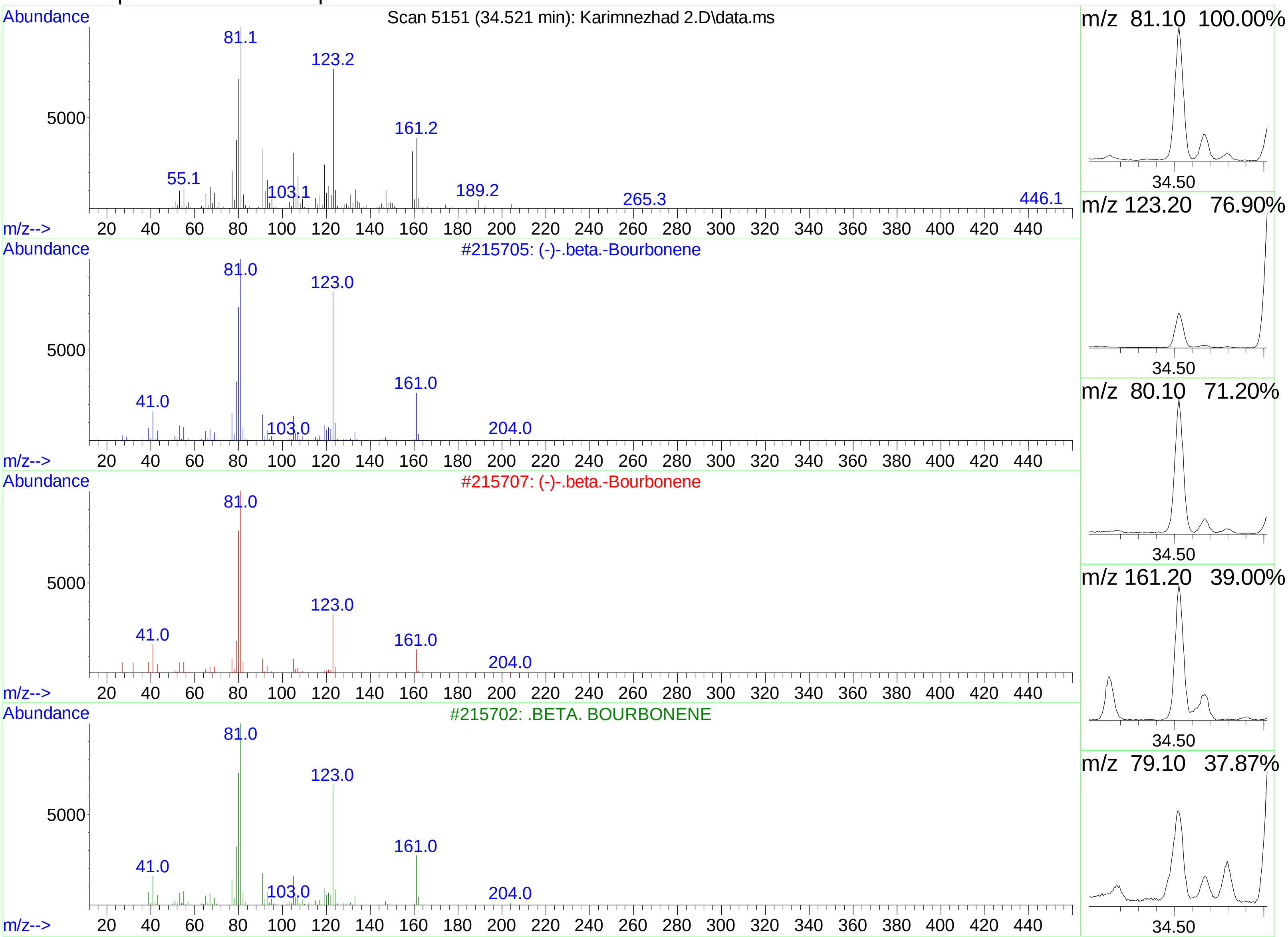

Data File: D:\msdchem\1\data\Karimnezhad 2.D
Sample : M10

Peak Number: 42 at 34.521 min Area: 30661144 Area % 0.26

The 3 best hits from each library.	Ref#	CAS#	Qual
D:\Database\W10N14.L			
1 (-)-.beta.-Bourbonene	215705	005208-59-3	95
2 (-)-.beta.-Bourbonene	215707	005208-59-3	93
3 .BETA. BOURBONENE	215702	005208-59-3	93

Unknown Spectrum based on Apex

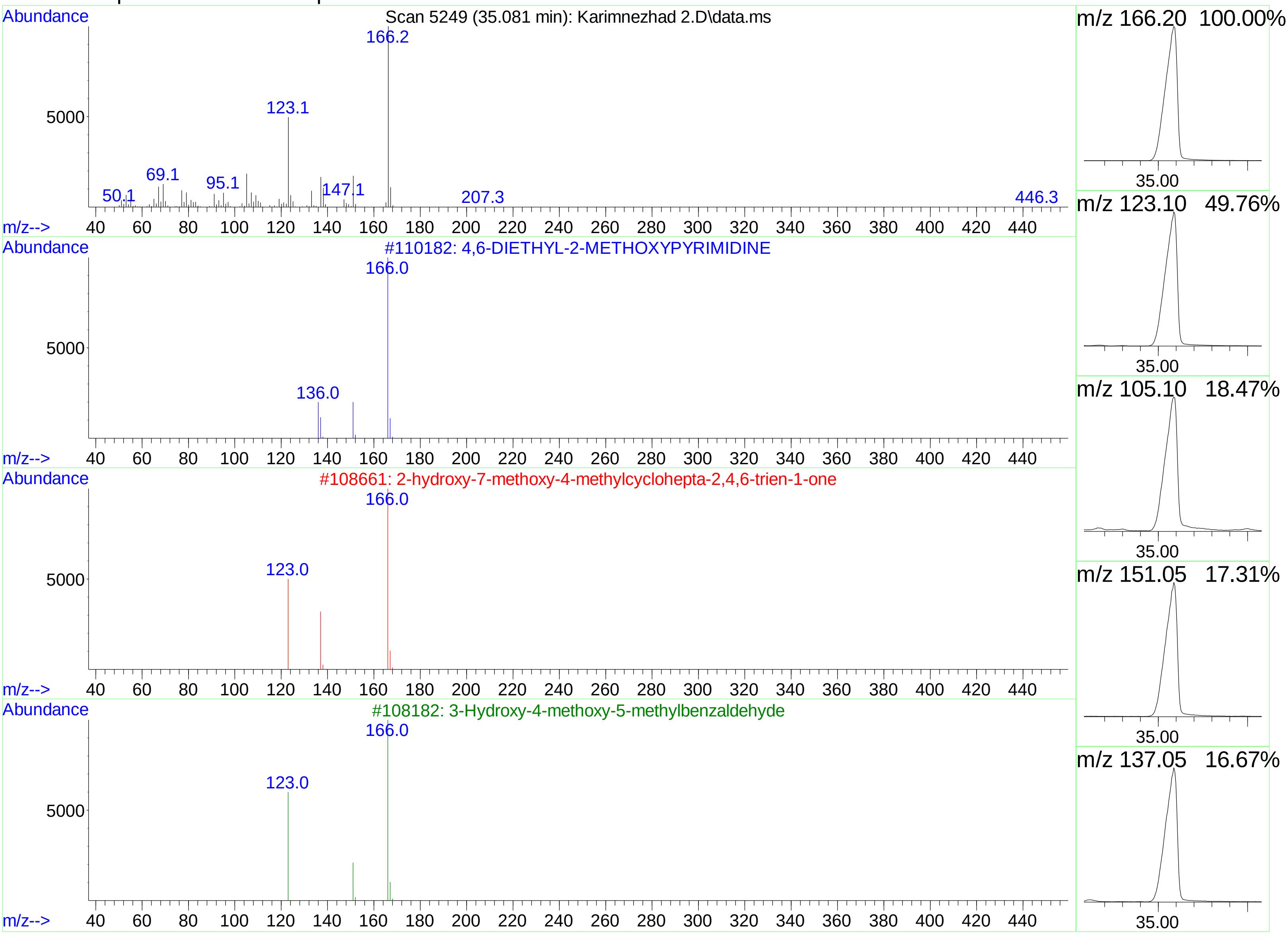

Data File: D:\msdchem\1\data\Karimnezhad 2.D
Sample : M10

Peak Number: 43 at 35.081 min Area: 390762852 Area % 3.27

The 3 best hits from each library.	Ref\#	CAS\#	Qual
D:\Database\W10N14.L			
1 4,6-DIETHYL-2-METHOXYPYRIMIDINE	110182	2000110-18-2	72
2 2-hydroxy-7-methoxy-4-methylcycl...	108661	2000108-66-1	64
3 3-Hydroxy-4-methoxy-5-methylbenz...	108182	2000108-18-2	64

Unknown Spectrum based on Apex

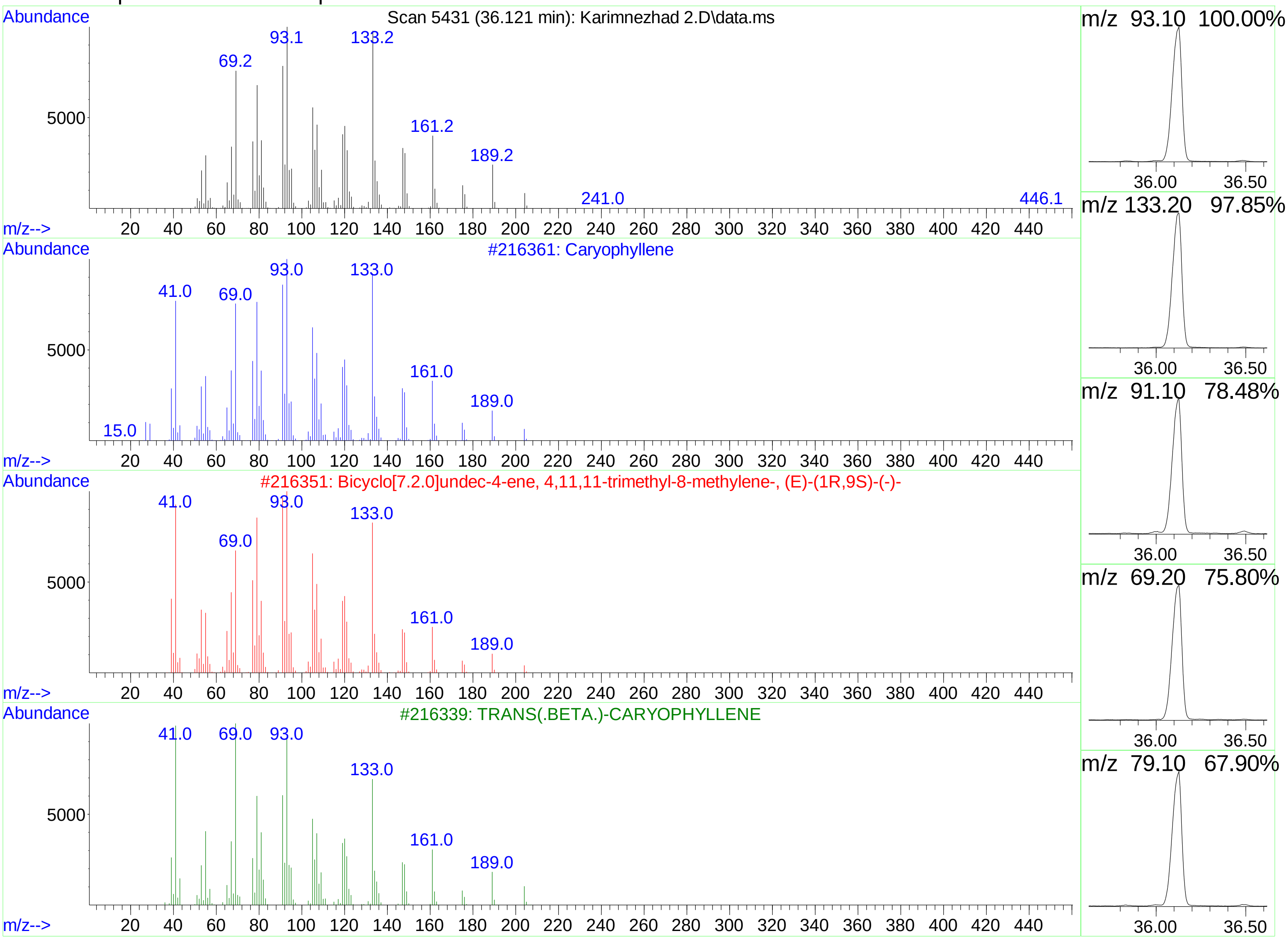

Data File: D:\msdchem\1\data\Karimnezhad 2.D
Sample : M10

Peak Number: 44 at 36.121 min Area: 242198032 Area % 2.03

The 3 best hits from each library. Ref\# CAS\# Qual

D:\Database\W10N14.L

1	Caryophyllene	216361	000087-44-5	99
2	Bicyclo[7.2.0]undec-4-ene, 4,11,...	216351	000087-44-5	99
3	TRANS(.BETA.)-CARYOPHYLLENE	216339	2000216-33-9	99

Unknown Spectrum based on Apex

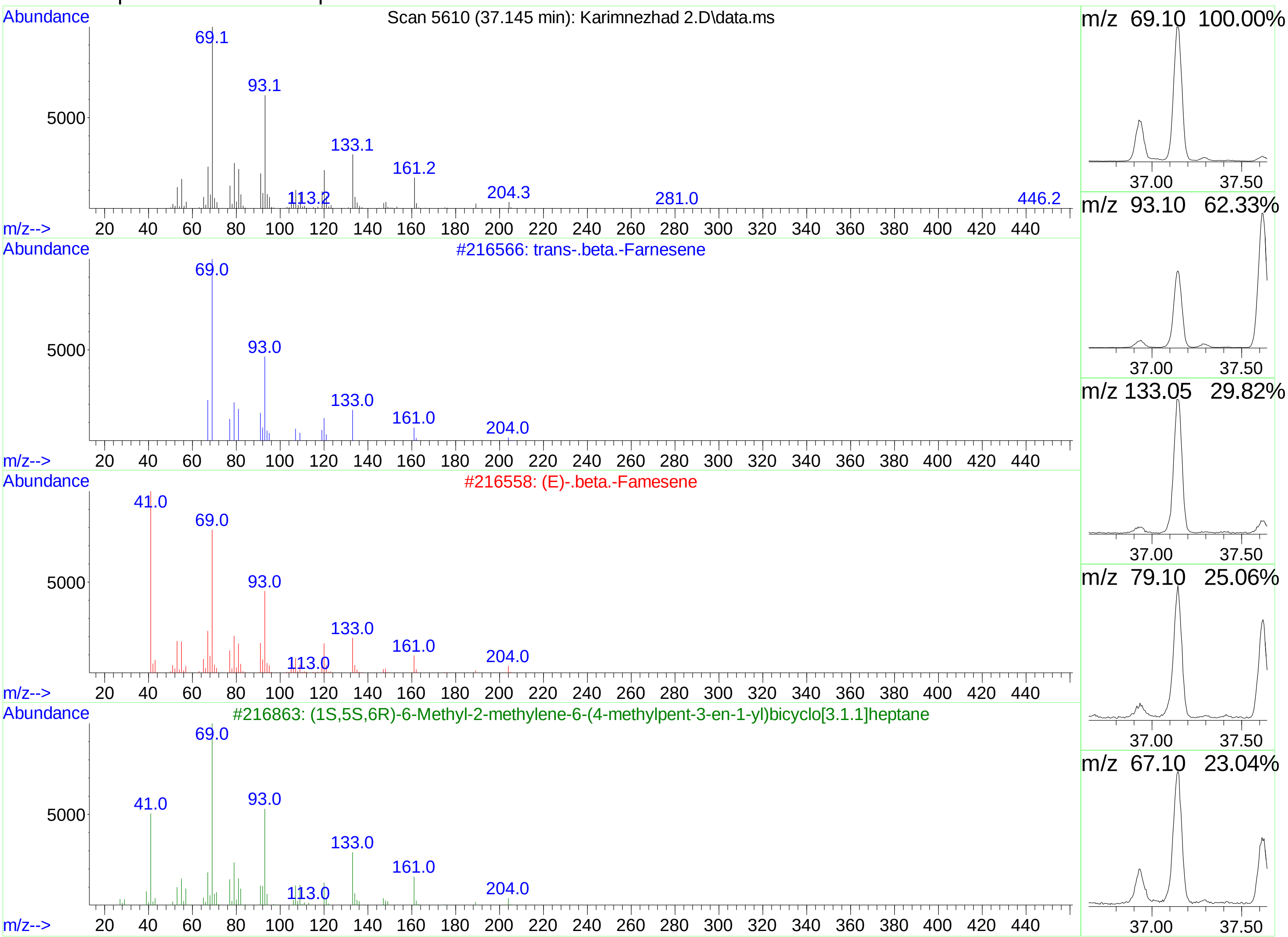

Data File: D:\msdchem\1\data\Karimnezhad 2.D
Sample : M10

Peak Number: 45 at 37.145 min Area: 31699958 Area % 0.27

The 3 best hits from each library.	Ref#	CAS#	Qual
D:\Database\W10N14.L			
1 trans-.beta.-Farnesene	216566	000502-60-3	96
2 (E)-.beta.-Famesene	216558	018794-84-8	96
3 (1S,5S,6R)-6-Methyl-2-methylene-...	216863	015438-94-5	96

Unknown Spectrum based on Apex

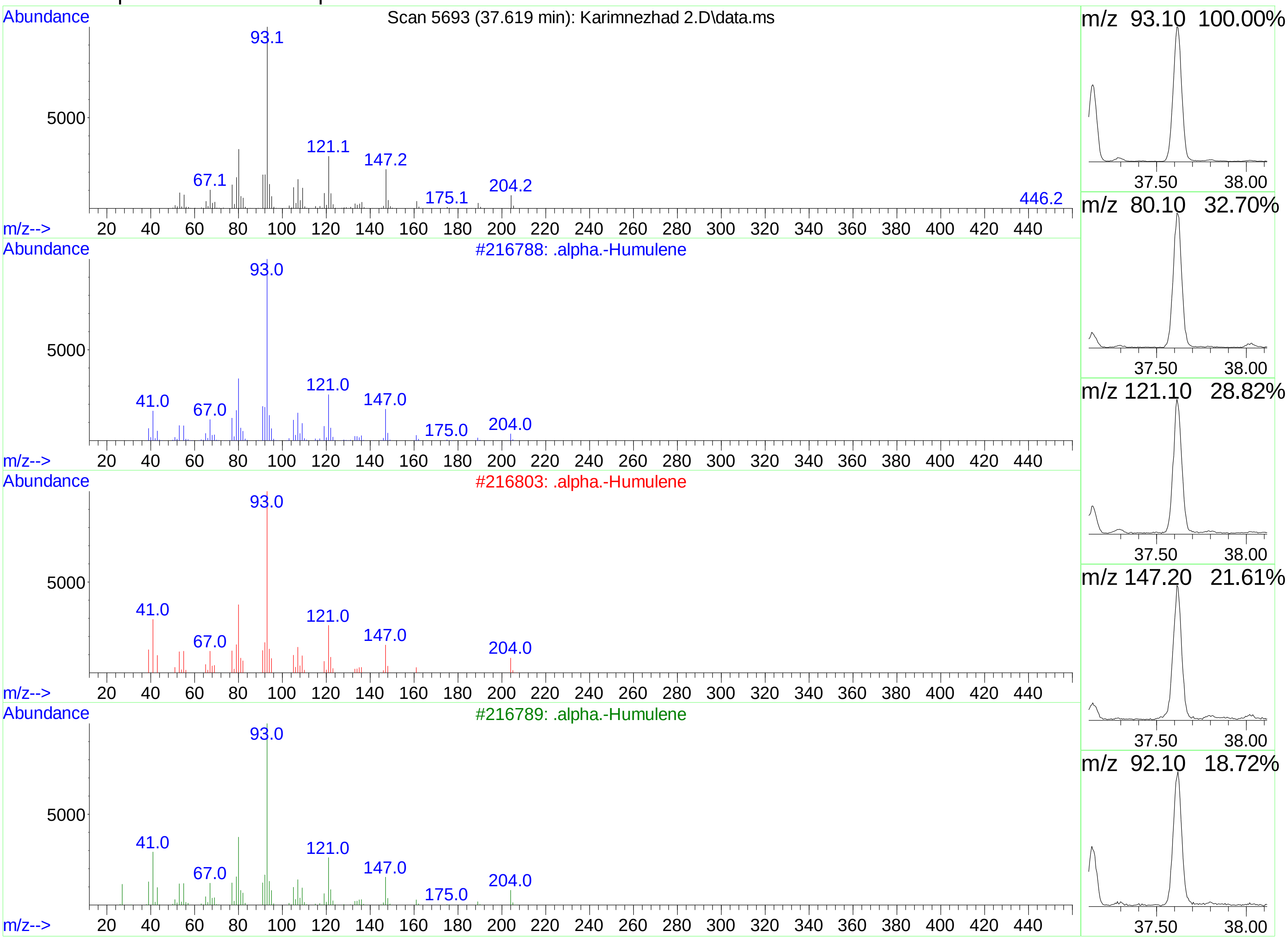

Data File: D:\msdchem\1\data\Karimnezhad 2.D
Sample : M10

Peak Number: 46 at 37.619 min Area: 31872964 Area % 0.27

The 3 best hits from each library.	Ref\#	CAS\#	Qual
D:\Database\W10N14.L			
1 .alpha.-Humulene	216788	006753-98-6	99
2 .alpha.-Humulene	216803	006753-98-6	98
3 .alpha.-Humulene	216789	006753-98-6	98

Unknown Spectrum based on Apex

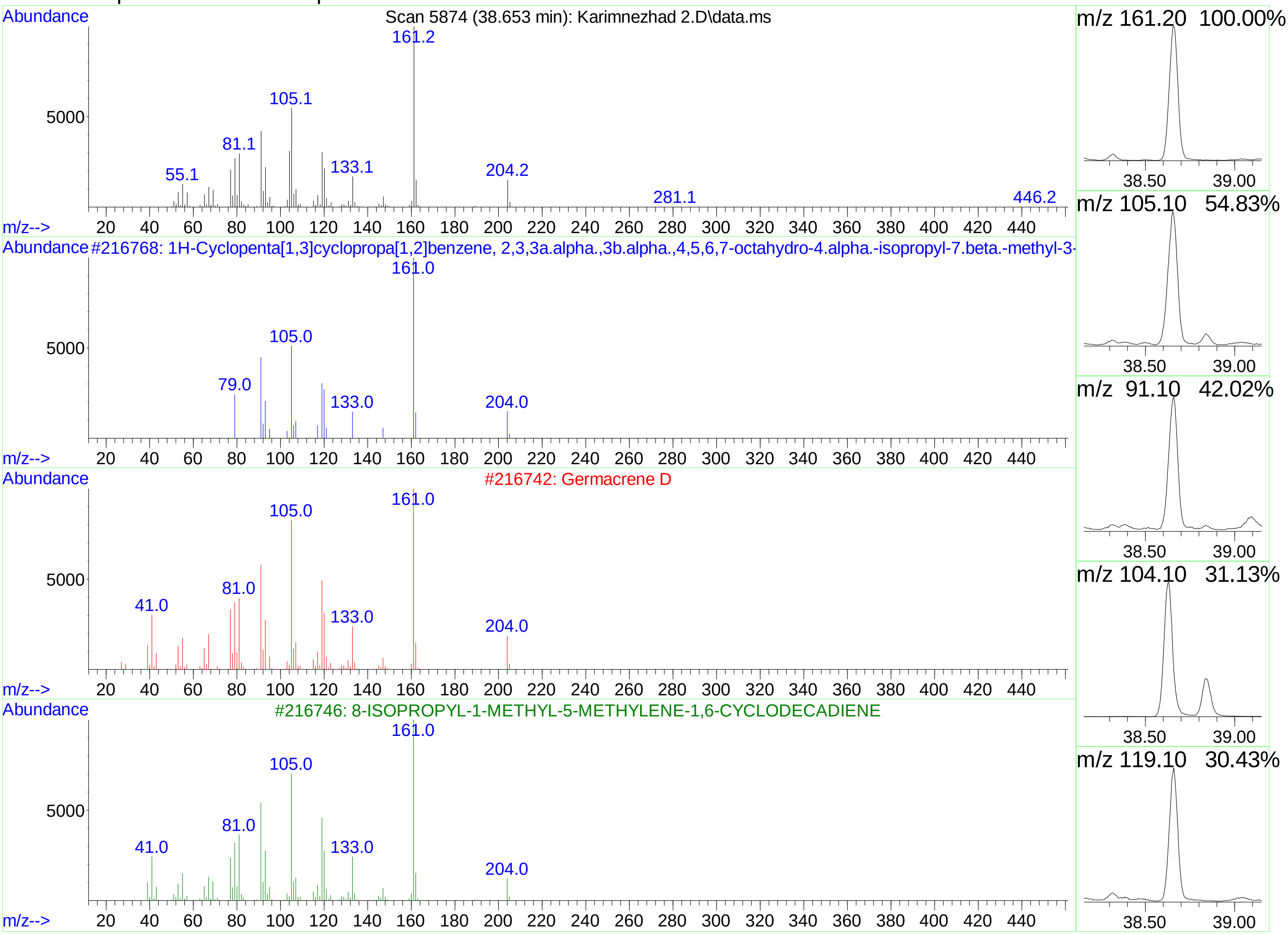

Data File: D:\msdchem\1\data\Karimnezhad 2.D
Sample : M10

Peak Number: 47 at 38.653 min Area: 66608551 Area % 0.56

The 3 best hits from each library.	Ref\#	CAS\#	Qual
D:\Database\W10N14.L			
1 1H-Cyclopenta[1,3]cyclopropa[1,2...	216768	013744-15-5	99
2 Germacrene D	216742	023986-74-5	99
3 8-ISOPROPYL-1-METHYL-5-METHYLENE...	216746	023986-74-5	98

Library Search Report - ChemStation Integrator

Unknown Spectrum based on Apex

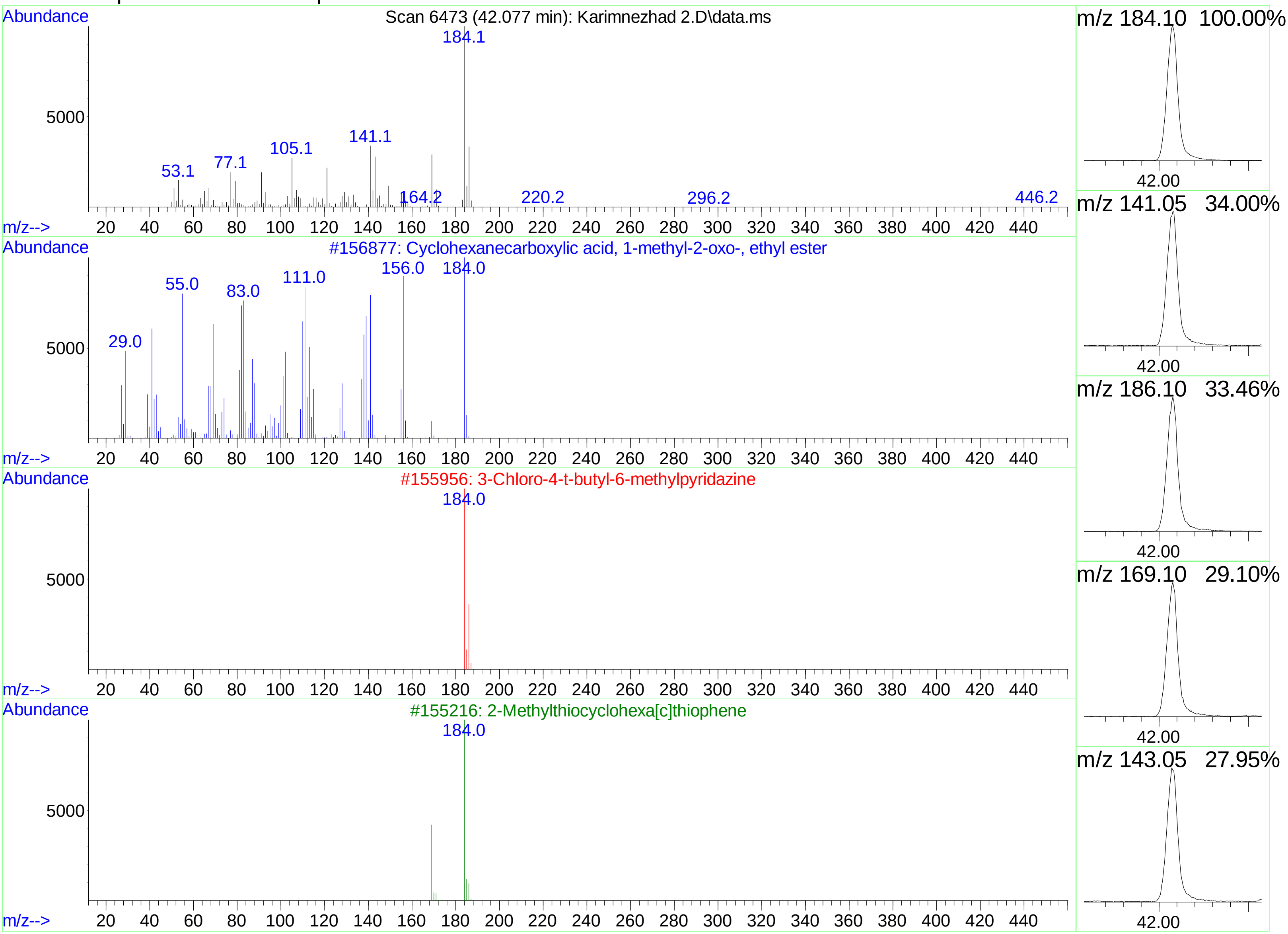

Data File: D:\msdchem\1\data\Karimnezhad 2.D
Sample : M10

Peak Number: 48 at 42.077 min Area: 75962261 Area % 0.64

The 3 best hits from each library.	Ref\#	CAS\#	Qual
D:\Database\W10N14.L			
1 Cyclohexanecarboxylic acid, 1-me...	156877	005453-94-1	83
2 3-Chloro-4-t-butyl-6-methylpyrid...	155956	2000155-95-6	83
3 2-Methylthiocyclohexa[c]thiophene	155216	000000-00-0	64

Unknown Spectrum based on Apex

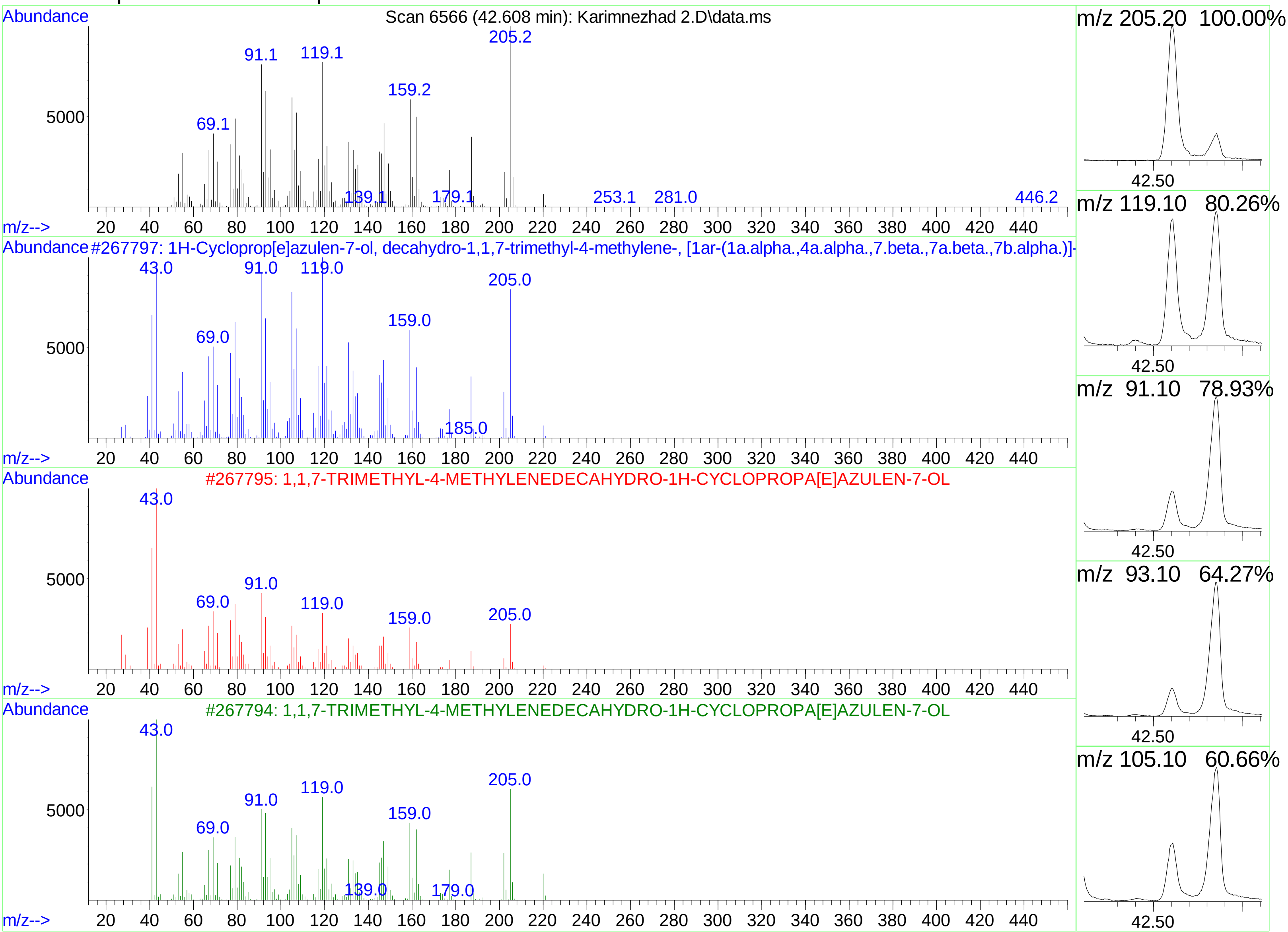

Data File: D:\msdchem\1\data\Karimnezhad 2.D
Sample : M10

Peak Number: 49 at 42.608 min Area: 72480059 Area % 0.61

The 3 best hits from each library. Ref\# CAS\# Qual

D:\Database\W10N14.L

1 1H-Cycloprop[e]azulen-7-ol, deca... 267797 006750-60-3 99
2 1,1,7-TRIMETHYL-4-METHYLENEDECAH... 267795 077171-55-2 98
3 1,1,7-TRIMETHYL-4-METHYLENEDECAH... 267794 006750-60-3 93

Unknown Spectrum based on Apex

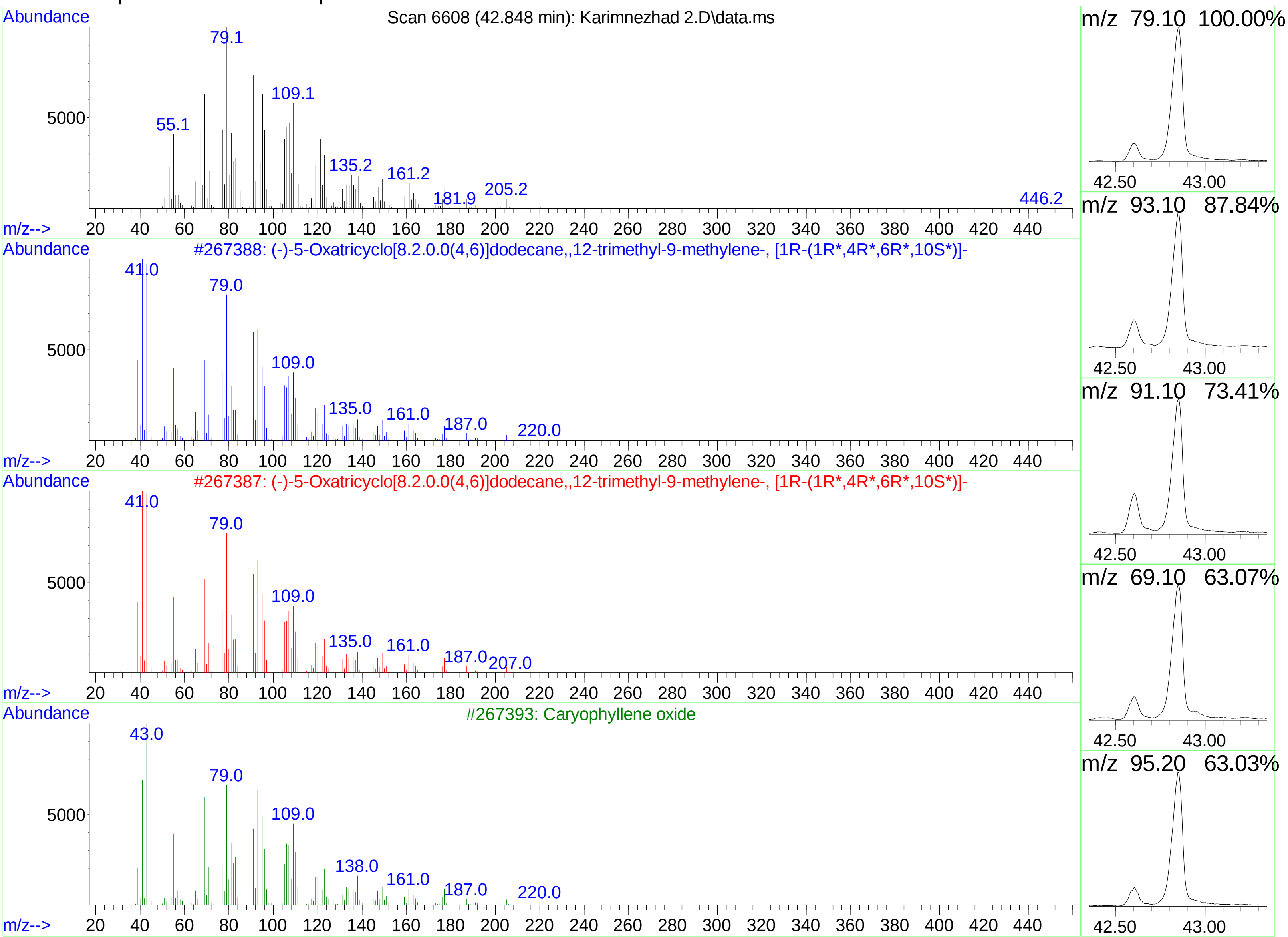

Data File: D:\msdchem\1\data\Karimnezhad 2.D
Sample : M10

Peak Number: 50 at 42.848 min Area: 259253339 Area % 2.17

The 3 best hits from each library. Ref\# CAS\# Qual

D:\Database\W10N14.L

1	(-)-5-Oxatricyclo[8.2.0.0(4,6)]d...	267388	001139-30-6	99
2	(-)-5-Oxatricyclo[8.2.0.0(4,6)]d...	267387	001139-30-6	95
3	Caryophyllene oxide	267393	001139-30-6	94

Unknown Spectrum based on Apex

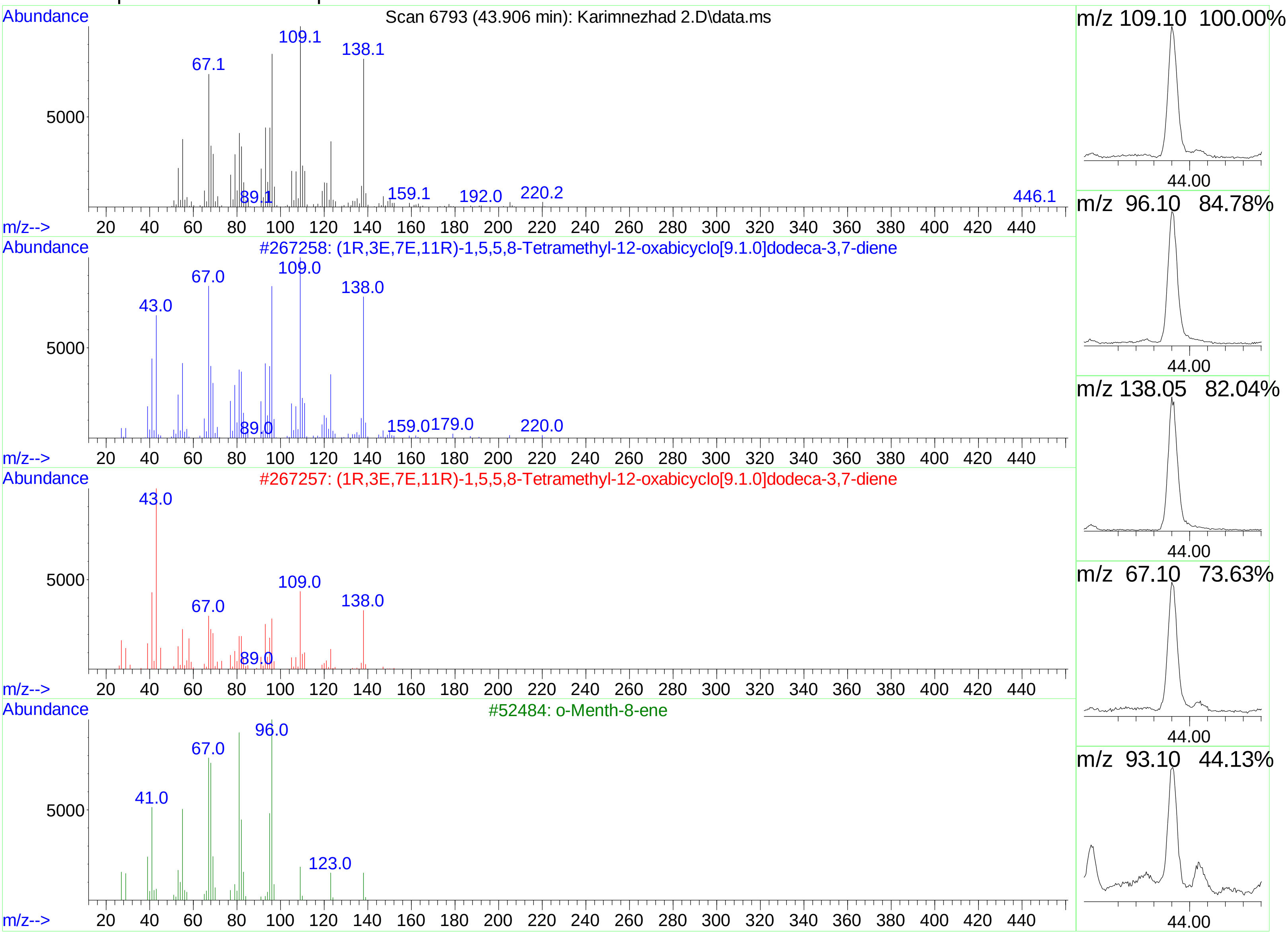

Data File: D:\msdchem\1\data\Karimnezhad 2.D
Sample : M10

Peak Number: 51 at 43.906 min Area: 24521060 Area % 0.21

The 3 best hits from each library. Ref\# CAS\# Qual

D:\Database\W10N14.L

1	(1R,3E,7E,11R)-1,5,5,8-Tetrameth...	267258	019888-34-7	99
2	(1R,3E,7E,11R)-1,5,5,8-Tetrameth...	267257	019888-34-7	87
3	o-Menth-8-ene	52484	015193-25-6	55

Unknown Spectrum based on Apex

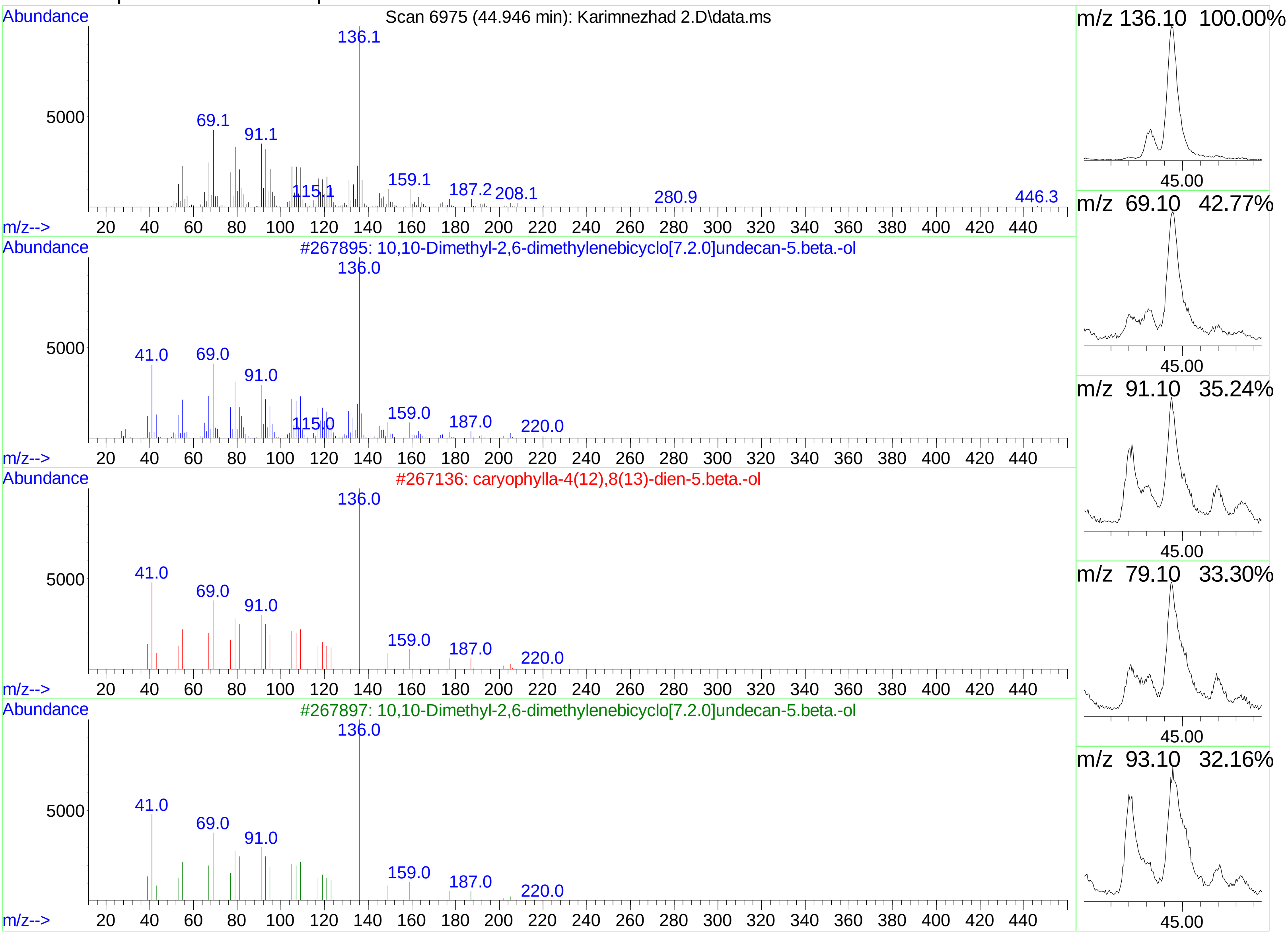

Data File: D:\msdchem\1\data\Karimnezhad 2.D
Sample : M10

Peak Number: 52 at 44.946 min Area: 29474686 Area % 0.25

The 3 best hits from each library.	Ref\#	CAS\#	Qual
D:\Database\W10N14.L			
1 10,10-Dimethyl-2,6-dimethylenebi...	267895	019431-80-2	98
2 caryophylla-4(12),8(13)-dien-5.b...	267136	2000267-13-6	95
3 10,10-Dimethyl-2,6-dimethylenebi...	267897	019431-80-2	95

Unknown Spectrum based on Apex

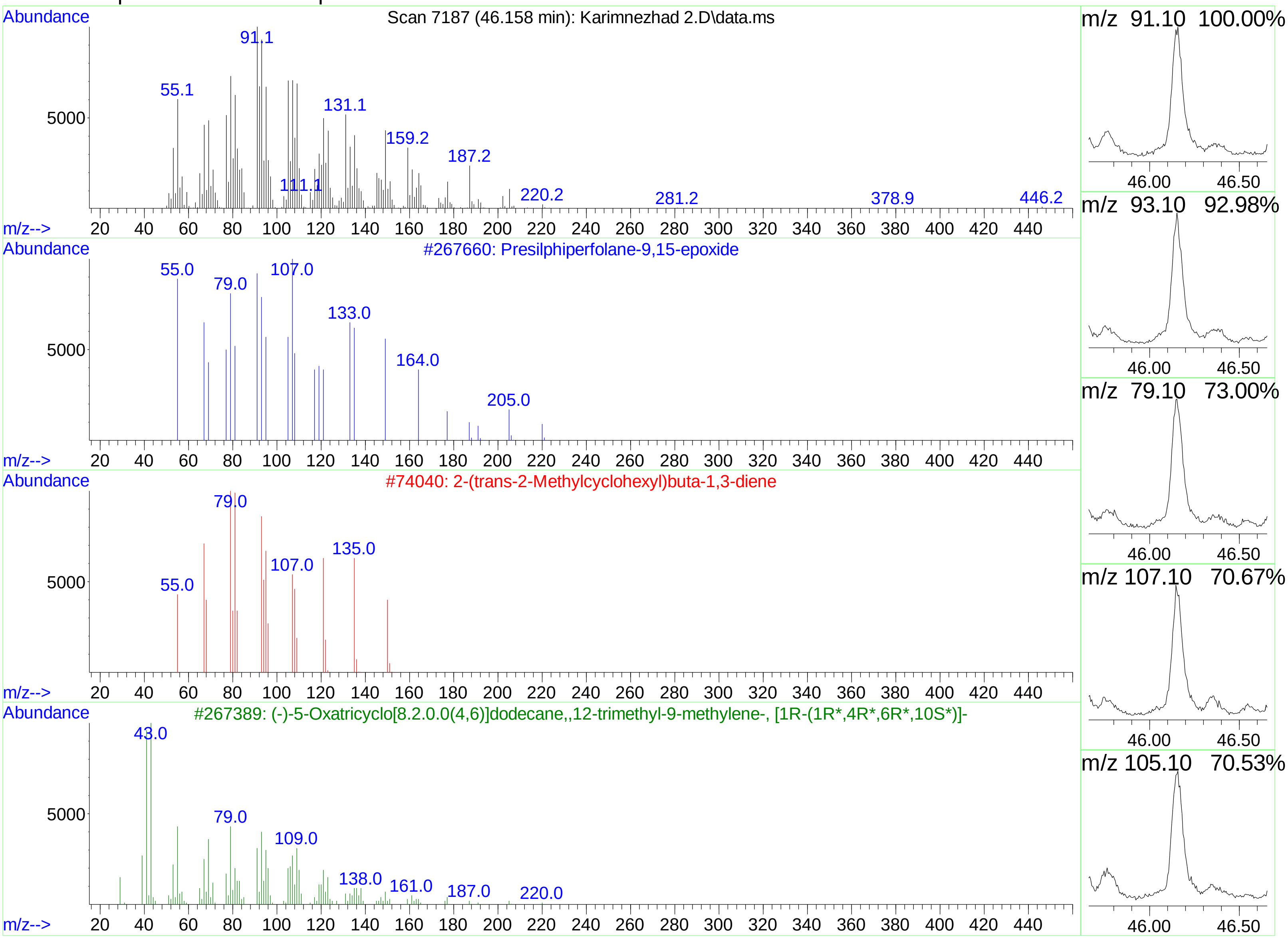

Data File: D:\msdchem\1\data\Karimnezhad 2.D
Sample : M10

Peak Number: 53 at 46.158 min Area: 31284468 Area % 0.26

The 3 best hits from each library.	Ref\#	CAS\#	Qual
D:\Database\W10N14.L			
1 Presilphiperfolane-9,15-epoxide	267660	2000267-66-0	87
2 2-(trans-2-Methylcyclohexyl)buta...	74040	2000074-04-0	70
3 (-)-5-Oxatricyclo[8.2.0.0(4,6)]d...	267389	001139-30-6	70

Library Search Report - ChemStation Integrator

Unknown Spectrum based on Apex

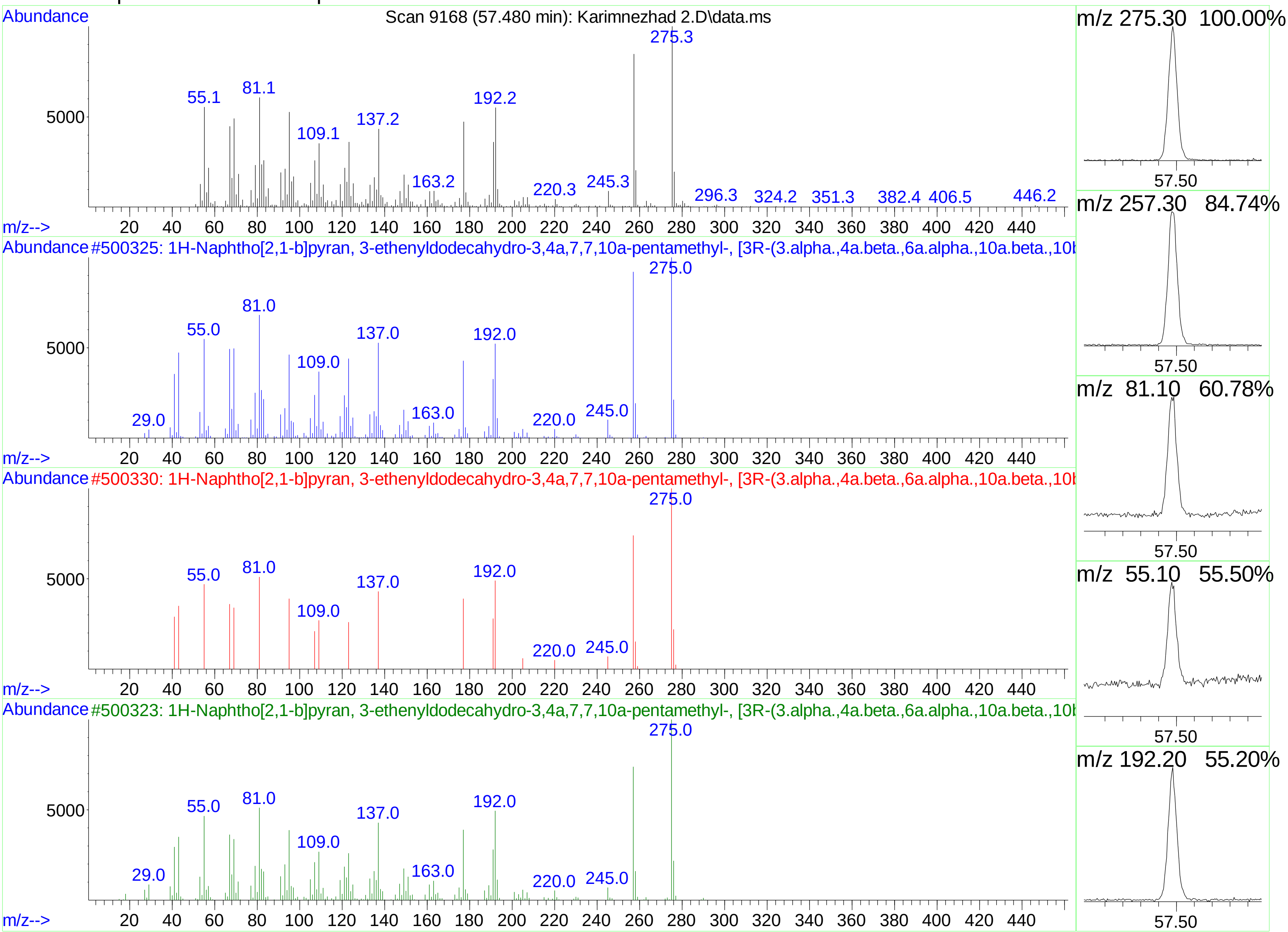

Data File: D:\msdchem\1\data\Karimnezhad 2.D
Sample : M10

Peak Number: 54 at 57.480 min Area: 17686005 Area % 0.15

The 3 best hits from each library.	Ref\#	CAS\#	Qual
D:\Database\W10N14.L			
1 1H-Naphtho[2,1-b]pyran, 3-etheny...	500325	000596-84-9	99
2 1H-Naphtho[2,1-b]pyran, 3-etheny...	500330	000596-84-9	95
3 1H-Naphtho[2,1-b]pyran, 3-etheny...	500323	000596-84-9	94
