# Supplementary material for: Comparison of quantity, quality and antibacterial activity of essential oil Mentha longifolia (L.) L. under different traditional and modern extraction methods
Source: PLoS One. 2024 Jul 10;19(7):e0301558. doi: 10.1371/journal.pone.0301558 (PMC11236116; doi:10.1371/journal.pone.0301558)

File :D:\msdchem\1\data\Karimnezhad 2.D
Operator : Jafari
Acquired : 15 Mar 2022 7:36 using AcqMethod QC.M
Instrument : GC MS
Sample Name: M10
Misc Info :
Vial Number: 30

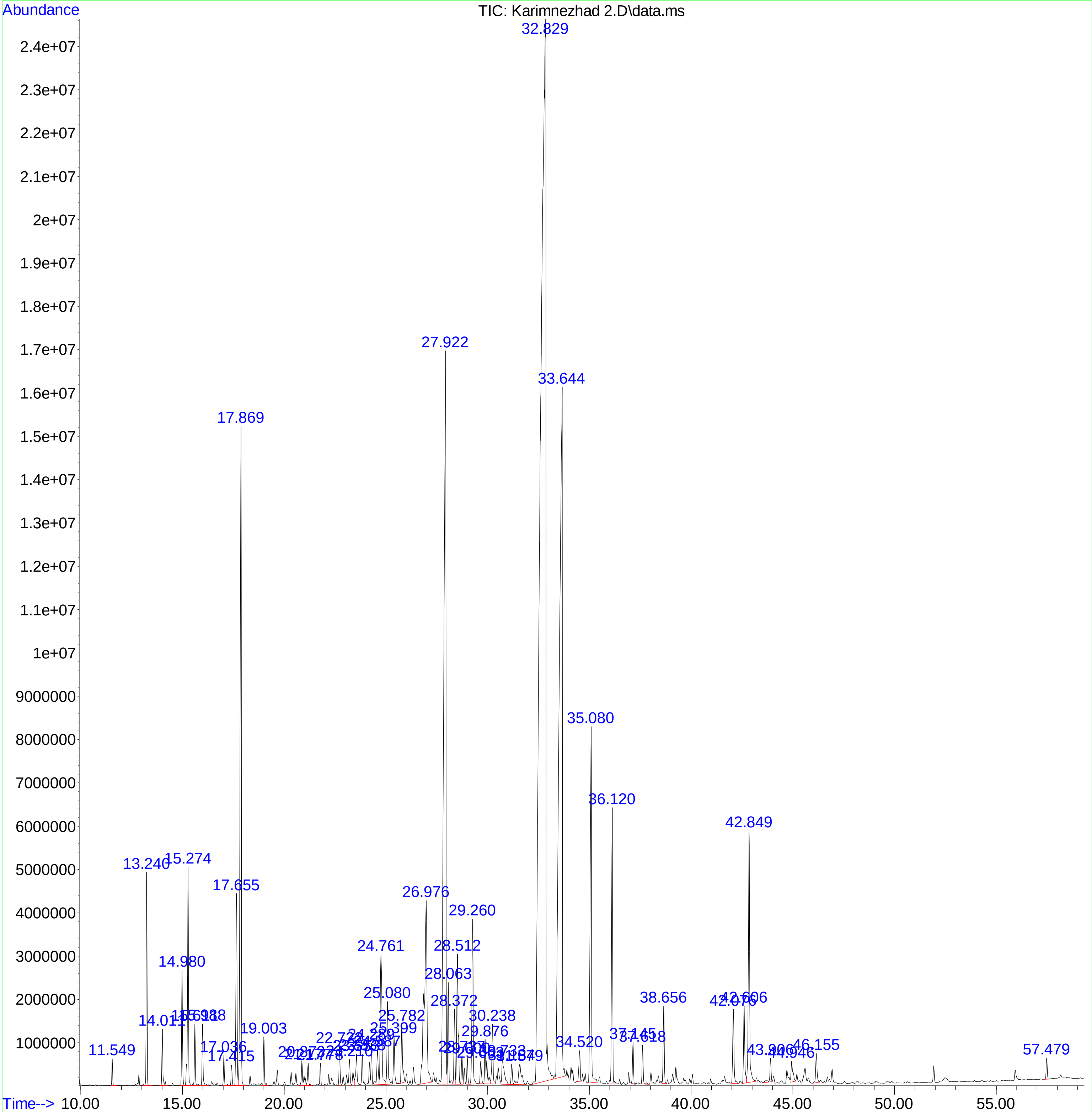

Supplement: S2 File — (ZIP) [file pone.0301558.s002.zip › Karimnezhad/M10/MSDA Print.pdf]
