# Supplementary material for: Comparison of quantity, quality and antibacterial activity of essential oil Mentha longifolia (L.) L. under different traditional and modern extraction methods
Source: PLoS One. 2024 Jul 10;19(7):e0301558. doi: 10.1371/journal.pone.0301558 (PMC11236116; doi:10.1371/journal.pone.0301558)
Supplement: S2 File — (ZIP) [file pone.0301558.s002.zip › Karimnezhad/M12/PrintText+summery.pdf]

Library Search Report

Data Path : D:\msdchem\1\data\
Data File : Karimnezhad 3.D
Acq On : 15 Mar 2022 8:52
Operator : Jafari
Sample : M12
Misc :
ALS Vial : 31 Sample Multiplier: 1

Search Libraries: D:\Database\W10N14.L Minimum Quality: 0

Unknown Spectrum: Apex
Integration Events: ChemStation Integrator - events.e

Pk#	RT	Area%	Library/ID	Ref#	CAS#	Qual
1	11.551	0.09	D:\Database\W10N14.L			
			Furan, 2,5-diethyltetrahydro-	36992	041239-48-9	95
			Furan, 2,5-diethyltetrahydro-	36993	041239-48-9	78
			Furan, 2,5-diethyltetrahydro-	36994	041239-48-9	78
2	13.237	0.88	D:\Database\W10N14.L			
			(1S)-2,6,6-Trimethylbicyclo[3.1.1] hept-2-ene	49099	007785-26-4	96
			2-Pinene	49090	000080-56-8	96
			(1R)-2,6,6-Trimethylbicyclo[3.1.1] hept-2-ene	49103	007785-70-8	96
3	14.009	0.29	D:\Database\W10N14.L			
			Camphene	49196	000079-92-5	97
			2,2-dimethyl-3-methylene-bicyclo [2.2.1]heptane	49207	000079-92-5	97
			Camphene	49195	000079-92-5	96
4	14.980	0.55	D:\Database\W10N14.L			
			Bicyclo[3.1.0]hexane, 4-methylene- 1-(1-methylethyl)-	48855	003387-41-5	96
			4(10)-Thujene	48870	003387-41-5	96
			4(10)-Thujene	48857	003387-41-5	96
5	15.272	1.22	D:\Database\W10N14.L			
			2(10)-Pinene	48535	000127-91-3	94
			2(10)-Pinene	48526	000127-91-3	94
			2(10)-Pinene	48529	000127-91-3	94
6	15.609	0.31	D:\Database\W10N14.L			
			.beta.-Myrcene	48617	000123-35-3	96
			1,6-Octadiene, 7-methyl-3-methylen e-	48627	000123-35-3	95
			1,6-Octadiene, 7-methyl-3-methylen e-	48620	000123-35-3	94
7	15.986	0.40	D:\Database\W10N14.L			
			3-Octanol	40544	000589-98-0	90
			3-Octanol	40530	000589-98-0	83
			ETHYL AMYL CARBINOL	40533	000589-98-0	83
8	17.038	0.16	D:\Database\W10N14.L			
			1,3-Cyclohexadiene, 1-methyl-4-(1-	48406	000099-86-5	98

Data Path : D:\msdchem\1\data\
Data File : Karimnezhad 3.D
Acq On : 15 Mar 2022 8:52
Operator : Jafari
Sample : M12
Misc :
ALS Vial : 31 Sample Multiplier: 1

Search Libraries: D:\Database\W10N14.L Minimum Quality: 0

Unknown Spectrum: Apex
Integration Events: ChemStation Integrator - events.e

Pk#	RT	Area%	Library/ID	Ref#	CAS#	Qual
			methylethyl)-			
			Cyclohexene, 1-methyl-4-(1-methylethylidene)-	48893	000586-62-9	97
			1,3-Cyclohexadiene, 1-methyl-4-(1-methylethyl)-	48409	000099-86-5	97
9	17.415	0.12	D:\Database\W10N14.L			
			Benzene, 1-methyl-4-(1-methylethyl)-	45373	000099-87-6	97
			p-Cymene	45379	000099-87-6	97
			Benzene, 1-methyl-2-(1-methylethyl)-	45118	000527-84-4	95
10	17.655	1.26	D:\Database\W10N14.L			
			D-Limonene	48457	005989-27-5	99
			Cyclohexene, 1-methyl-4-(1-methylethenyl)-	48442	000138-86-3	98
			Cyclohexene, 1-methyl-4-(1-methylethenyl)-	48478	000138-86-3	98
11	17.872	4.90	D:\Database\W10N14.L			
			2-Oxabicyclo[2.2.2]octane, 1,3,3-trimethyl-	83024	000470-82-6	98
			2-Oxabicyclo[2.2.2]octane, 1,3,3-trimethyl-	83030	000470-82-6	98
			2-Oxabicyclo[2.2.2]octane, 1,3,3-trimethyl-	83023	000470-82-6	97
12	19.004	0.27	D:\Database\W10N14.L			
			1,4-Cyclohexadiene, 1-methyl-4-(1-methylethyl)-	48401	000099-85-4	97
			1,4-Cyclohexadiene, 1-methyl-4-(1-methylethyl)-	48392	000099-85-4	96
			1,4-Cyclohexadiene, 1-methyl-4-(1-methylethyl)-	48376	000099-85-4	96
13	19.667	0.16	D:\Database\W10N14.L			
			Bicyclo[3.1.0]hexan-2-ol, 2-methyl-5-(1-methylethyl)-, (1.alpha.,2.alpha.,5.alpha.)-	83607	017699-16-0	98
			Cyclohexanol, 1-methyl-4-(1-methylethenyl)-, cis-	83200	007299-41-4	96
			4-Thujanol, stereoisomer	83602	017699-16-0	96

Library Search Report

Data Path : D:\msdchem\1\data\
Data File : Karimnezhad 3.D
Acq On : 15 Mar 2022 8:52
Operator : Jafari
Sample : M12
Misc :
ALS Vial : 31 Sample Multiplier: 1

Search Libraries: D:\Database\W10N14.L Minimum Quality: 0

Unknown Spectrum: Apex
Integration Events: ChemStation Integrator - events.e

Pk#	RT	Area%	Library/ID	Ref#	CAS#	Qual
14	20.873	0.15	D:\Database\W10N14.L			
			Butanoic acid, 2-methyl-, 3-methyl butyl ester	126288	027625-35-0	86
			isopentyl 2-methylbutanoate	126284	027625-35-0	83
			ISOAMYL-2-METHYL BUTYRATE	126282	027625-35-0	72
15	20.982	0.25	D:\Database\W10N14.L			
			Linalool	84218	000078-70-6	97
			LINALOOL L	84211	000078-70-6	97
			1,6-OCTADIEN-3-OL, 3,7-DIMETHYL-	84202	000078-70-6	96
16	21.176	0.17	D:\Database\W10N14.L			
			Pentane, 3-ethyl-	12123	000617-78-7	38
			Cyclohexanol, 1-ethyl-	37501	001940-18-7	38
			1-OCTEN-3-YL ACETATE	92518	002442-10-6	30
17	21.776	0.13	D:\Database\W10N14.L			
			3-Octanol, acetate	126339	004864-61-3	91
			3-Octanol, acetate	126344	004864-61-3	53
			3-Octanol, acetate	126341	004864-61-3	47
18	22.725	0.26	D:\Database\W10N14.L			
			2-Methoxy-1,4-benzenediamine	51301	005307-02-8	86
			1-Fluoro-2,4,5-trimethylbenzene	51346	000400-01-1	72
			1,1'-Bicyclopentyl	52364	001636-39-1	70
19	23.211	0.21	D:\Database\W10N14.L			
			Bicyclo[3.1.1]heptan-3-ol, 6,6-dim ethyl-2-methylene-, [1S-(1.alpha., 3.alpha.,5.alpha.)]-	78364	000547-61-5	90
			2(10)-Pinen-3-ol, (1S,3R,5S)-(-)-	78358	000547-61-5	90
			Isopinocarveol	78365	006712-79-4	81
20	23.496	0.78	D:\Database\W10N14.L			
			(+)-2-Bornanone	78661	000464-49-3	98
			Camphor	78669	000076-22-2	98
			Bicyclo[2.2.1]heptan-2-one, 1,7,7-trimethyl-	78649	000076-22-2	98
21	23.839	0.25	D:\Database\W10N14.L			
			Cyclohexanone, 5-methyl-2-(1-methy lethyl)-, trans-	83410	000089-80-5	98

Data Path : D:\msdchem\1\data\
Data File : Karimnezhad 3.D
Acq On : 15 Mar 2022 8:52
Operator : Jafari
Sample : M12
Misc :
ALS Vial : 31 Sample Multiplier: 1

Search Libraries: D:\Database\W10N14.L Minimum Quality: 0

Unknown Spectrum: Apex
Integration Events: ChemStation Integrator - events.e

```
Pk#    RT  Area%        Library/ID              Ref#    CAS#   Qual
____________________________________________________________________
           2-ISOPROPYL-5-METHYLCYCLOHEXANONE   83421 014073-97-3 98
           2-ISOPROPYL-5-METHYLCYCLOHEXANONE   83413 000089-80-5 98

 22  24.291  0.51 D:\Database\W10N14.L
           Cyclohexanone, 5-methyl-2-(1-methy  83410 000089-80-5 98
           lethyl)-, trans-
           Cyclohexanone, 5-methyl-2-(1-methy  83407 000491-07-6 97
           lethyl)-, cis-
           Cyclohexanone, 5-methyl-2-(1-methy  83423 010458-14-7 97
           lethyl)-

 23  24.599  0.40 D:\Database\W10N14.L
           Cyclohexanemethanol, .alpha.,.alph  83142 007299-42-5 80
           a.-dimethyl-4-methylene-
           L-.alpha.-Terpineol                 82880 010482-56-1 59
           3-Cyclohexene-1-methanol, .alpha.,  82895 010482-56-1 59
           .alpha.,4-trimethyl-, (S)- (CAS)

 24  24.771  2.18 D:\Database\W10N14.L
           Bicyclo[2.2.1]heptan-2-ol, 1,7,7-t  84061 000464-45-9 95
           rimethyl-, (1S-endo)-
           endo-Borneol                        84059 000507-70-0 95
           1,7,7-TRIMETHYLBICYCLO[2.2.1]HEPTA  84051 000464-45-9 94
           N-2-OL

 25  25.085  0.70 D:\Database\W10N14.L
           1-ISOPROPYL-4-METHYL-3-CYCLOHEXEN-  82925 000562-74-3 98
           1-OL
           3-Cyclohexen-1-ol, 4-methyl-1-(1-m  82943 000562-74-3 98
           ethylethyl)-
           3-Cyclohexen-1-ol, 4-methyl-1-(1-m  82938 000562-74-3 97
           ethylethyl)-

 26  25.411  0.35 D:\Database\W10N14.L
           m-Cymen-8-ol                        73157 005208-37-7 91
           PARA-CYMEN-8-OL                     72711 001197-01-9 90
           Benzenemethanol, .alpha.,.alpha.,4  72702 001197-01-9 87
           -trimethyl-

 27  25.788  0.78 D:\Database\W10N14.L
           Cyclohexene, 1-methyl-3-(1-methyle  48587 000499-03-6 95
           thenyl)-, (.+-.)-
           3-Cyclohexene-1-methanol, .alpha.,  82910 000098-55-5 87
```

Library Search Report

Data Path : D:\msdchem\1\data\
Data File : Karimnezhad 3.D
Acq On : 15 Mar 2022 8:52
Operator : Jafari
Sample : M12
Misc :
ALS Vial : 31 Sample Multiplier: 1

Search Libraries: D:\Database\W10N14.L Minimum Quality: 0

Unknown Spectrum: Apex
Integration Events: ChemStation Integrator - events.e

Pk# RT Area% Library/ID Ref# CAS# Qual

.alpha.,4-trimethyl-
2-(4-METHYL-3-CYCLOHEXEN-1-YL)-2-P 82897 000098-55-5 87
ROPANOL

28 26.160 0.22 D:\Database\W10N14.L
2-Norpinene-2-ethanol, 6,6-dimethy 110473 000128-50-7 91
l-
Bicyclo[2.2.1]heptan-2-ol, 1,7,7-t 84042 000124-76-5 86
rimethyl-, exo-
Isoborneol 84072 000124-76-5 86

29 26.371 0.32 D:\Database\W10N14.L
2-Pinen-4-one 73532 000080-57-9 96
2-Pinen-4-one 73515 000080-57-9 96
Bicyclo[3.1.1]hept-3-en-2-one, 4,6 73516 000080-57-9 96
,6-trimethyl-

30 26.994 3.57 D:\Database\W10N14.L
8,9-Dehydrothymol 68975 018612-99-2 95
1-methoxy-4-(1-methylethenyl)benze 68640 2000068-64-0 81
ne
1-Isopropenyl-4-methoxy-benzene 68642 2000068-64-2 80

31 27.937 10.23 D:\Database\W10N14.L
Pulegone 78101 000089-82-7 97
Cyclohexanone, 5-methyl-2-(1-methy 78096 015932-80-6 96
lethylidene)-
(R)-5-methyl-2-(1-methylethylidene 78102 000089-82-7 96
)-cyclohexanone

32 28.069 0.65 D:\Database\W10N14.L
D-Carvone 72855 002244-16-8 96
2-Cyclohexen-1-one, 2-methyl-5-(1- 72847 006485-40-1 95
methylethenyl)-, (R)-
2-Cyclohexen-1-one, 2-methyl-5-(1- 72827 000099-49-0 95
methylethenyl)-

33 28.377 0.80 D:\Database\W10N14.L
7-Oxabicyclo[4.1.0]heptan-2-one, 6 114758 035178-55-3 98
-methyl-3-(1-methylethylidene)-
4-ISOPROPENYL-1-METHYL-7-OXABICYCL 114757 035178-55-3 52
O[4.1.0]HEPTAN-2-ONE
CYCLOHEXANE, 1,2,3-TRIMETHYL- 34625 001678-97-3 45

Data Path : D:\msdchem\1\data\
Data File : Karimnezhad 3.D
Acq On : 15 Mar 2022 8:52
Operator : Jafari
Sample : M12
Misc :
ALS Vial : 31 Sample Multiplier: 1

Search Libraries: D:\Database\W10N14.L Minimum Quality: 0

Unknown Spectrum: Apex
Integration Events: ChemStation Integrator - events.e

Pk#	RT	Area%	Library/ID	Ref#	CAS#	Qual
34	28.526	1.20	D:\Database\W10N14.L			
			7-Oxabicyclo[4.1.0]heptan-2-one, 6 -methyl-3-(1-methylethylidene)-	114758	035178-55-3	94
			4-ISOPROPENYL-1-METHYL-7-OXABICYCL O[4.1.0]HEPTAN-2-ONE	114757	035178-55-3	93
			7-Oxabicyclo[4.1.0]heptan-2-one, 6 -methyl-3-(1-methylethyl)-	115503	005286-38-4	87
35	28.737	0.17	D:\Database\W10N14.L			
			1,1'-Bicyclopentyl	52363	001636-39-1	52
			1,1'-Bicyclopentyl	52365	001636-39-1	50
			3a,4,5,6,7,7a-hexahydro-4,7-methan obenz[d]isoxazole	49858	015166-80-0	49
36	29.006	0.18	D:\Database\W10N14.L			
			1-ethyl-3-methyl-2-propylidenimida zolidine	84359	109153-29-9	83
			4-Fluoro-2-acetylphenol	81032	000394-32-1	80
			2,4-Dimethoxyphenol	81451	013330-65-9	80
37	29.257	0.99	D:\Database\W10N14.L			
			(S)-(+)-cis-Isopiperitenone	72898	2000072-89-8	91
			1,8-(p-MENTHADIENONE)	72899	2000072-89-9	72
			2-Cyclohexen-1-one, 3,5,5-trimethy l-	51808	000078-59-1	50
38	29.663	0.18	D:\Database\W10N14.L			
			4-Hydroxy-3-methylacetophenone	72072	000876-02-8	91
			4-Methyl-1-(acetoxy)benzene	72241	000140-39-6	90
			4-(Methoxymethyl)benzenaldehyde	72078	2000072-07-8	90
39	29.880	0.67	D:\Database\W10N14.L			
			Bicyclo[2.2.1]heptan-2-ol, 1,7,7-t rimethyl-, acetate, (1S-endo)-	192108	005655-61-8	99
			Bicyclo[2.2.1]heptan-2-ol, 1,7,7-t rimethyl-, acetate, (1S-endo)-	192123	005655-61-8	99
			2-Norbornanol, 1,3,3-trimethyl-, a cetate, endo-	192153	004057-31-2	98
40	30.240	0.45	D:\Database\W10N14.L			
			Benzene, 1-ethoxy-4-ethyl-	73585	001585-06-4	89
			Benzene, 1-ethoxy-4-ethyl- (CAS)	73584	001585-06-4	89

Data Path : D:\msdchem\1\data\
Data File : Karimnezhad 3.D
Acq On : 15 Mar 2022 8:52
Operator : Jafari
Sample : M12
Misc :
ALS Vial : 31 Sample Multiplier: 1

Search Libraries: D:\Database\W10N14.L Minimum Quality: 0

Unknown Spectrum: Apex
Integration Events: ChemStation Integrator - events.e

Pk#	RT	Area%	Library/ID	Ref#	CAS#	Qual
			2-Pinen-7-one	73397	000473-06-3	81
41	30.755	0.36	D:\Database\W10N14.L			
			Phenol, 5-methyl-2-(1-methylethyl)	73199	000089-83-8	95
			Phenol, 5-methyl-2-(1-methylethyl)	73200	000089-83-8	95
			Thymol	73196	000089-83-8	94
42	31.206	0.18	D:\Database\W10N14.L			
			3-Methyl-4-isopropylphenol	73390	003228-02-2	94
			3-Methyl-4-isopropylphenol	73391	003228-02-2	94
			Carvacrol	72968	000499-75-2	94
43	31.583	0.38	D:\Database\W10N14.L			
			Durohydroquinone	109438	000527-18-4	81
			Phenol, 3-methoxy-2,4,6-trimethyl-	109755	034883-05-1	76
			Ethanone, 1-(2-hydroxy-5-methoxyph enyl)-	108435	000705-15-7	76
44	32.841	34.44	D:\Database\W10N14.L			
			2-Cyclohexen-1-one, 3-methyl-6-(1- methylethylidene)-	73417	000491-09-8	98
			2-Cyclohexen-1-one, 3-methyl-6-(1- methylethylidene)-	73419	000491-09-8	97
			4,7,7-Trimethylbicyclo[4.1.0]hept- 3-en-2-one	72780	081800-50-2	93
45	33.652	13.74	D:\Database\W10N14.L			
			PIPERITENONE OXIDE	110057	003564-96-3	99
			4-Acetyl-1-methylcyclohexene	51791	006090-09-1	60
			2,4-Heptadienal, 2-methyl-6-oxo-, (E,E)-	51105	129454-99-5	58
46	34.527	0.26	D:\Database\W10N14.L			
			.BETA. BOURBONENE	215702	005208-59-3	96
			(-)-.beta.-Bourbonene	215705	005208-59-3	96
			(-)-.beta.-Bourbonene	215706	005208-59-3	87
47	35.081	2.61	D:\Database\W10N14.L			
			2-hydroxy-7-methoxy-4-methylcycloh epta-2,4,6-trien-1-one	108661	2000108-66-1	64
			3-Hydroxy-4-methoxy-5-methylbenzal dehyde	108182	2000108-18-2	64
			2-Hydroxy-7-methoxy-4-methylcycloh	108662	2000108-66-2	64

Data Path : D:\msdchem\1\data\
Data File : Karimnezhad 3.D
Acq On : 15 Mar 2022 8:52
Operator : Jafari
Sample : M12
Misc :
ALS Vial : 31 Sample Multiplier: 1

Search Libraries: D:\Database\W10N14.L Minimum Quality: 0

Unknown Spectrum: Apex
Integration Events: ChemStation Integrator - events.e

Pk#	RT	Area%	Library/ID	Ref#	CAS#	Qual
			epta-2,4,6-trien-1-one			
48	36.127	2.33	D:\Database\W10N14.L			
			Caryophyllene	216361	000087-44-5	99
			Bicyclo[7.2.0]undec-4-ene, 4,11,11 -trimethyl-8-methylene-, (E)-(1R,9 S)-(-)-	216351	000087-44-5	99
			TRANS(.BETA.)-CARYOPHYLLENE	216339	2000216-33-9	99
49	37.144	0.29	D:\Database\W10N14.L			
			trans-.beta.-Farnesene	216566	000502-60-3	97
			cis-.beta.-Farnesene	216569	028973-97-9	97
			(E)-.beta.-Famesene	216558	018794-84-8	96
50	37.619	0.36	D:\Database\W10N14.L			
			.alpha.-Humulene	216788	006753-98-6	99
			.alpha.-Humulene	216803	006753-98-6	98
			.alpha.-Humulene	216789	006753-98-6	98
51	38.659	0.65	D:\Database\W10N14.L			
			1H-Cyclopenta[1,3]cyclopropa[1,2]b enzene, 2,3,3a.alpha.,3b.alpha.,4, 5,6,7-octahydro-4.alpha.-isopropyl -7.beta.-methyl-3-methylene-	216768	013744-15-5	99
			Germacrene D	216742	023986-74-5	99
			8-ISOPROPYL-1-METHYL-5-METHYLENE-1 ,6-CYCLODECADIENE	216746	023986-74-5	98
52	39.253	0.19	D:\Database\W10N14.L			
			Bicyclo[8.1.0]undeca-2,6-diene, 3, 7,11,11-tetramethyl-, (1R*,2Z,6E,1 0R*)-(.+-.)-	215949	100762-46-7	98
			(1S,2E,6E,10R)-3,7,11,11-Tetrameth ylbicyclo[8.1.0]undeca-2,6-diene	215953	024703-35-3	95
			Bicyclo[8.1.0]undeca-2,6-diene, 3, 7,11,11-tetramethyl-, (1R*,2Z,6E,1 0R*)-(.+-.)-	215948	100762-46-7	94
53	42.083	0.74	D:\Database\W10N14.L			
			3-Chloro-4-t-butyl-6-methylpyridaz ine	155956	2000155-95-6	83
			5(6)-(Chloromethyl)benzo[1,2-c]-1, 2,5-oxadiazole N1-Oxide	154801	2000154-80-1	59

Data Path : D:\msdchem\1\data\
Data File : Karimnezhad 3.D
Acq On : 15 Mar 2022 8:52
Operator : Jafari
Sample : M12
Misc :
ALS Vial : 31 Sample Multiplier: 1

Search Libraries: D:\Database\W10N14.L Minimum Quality: 0

Unknown Spectrum: Apex
Integration Events: ChemStation Integrator - events.e

Pk#	RT	Area%	Library/ID	Ref#	CAS#	Qual
			2,4,6(1H,3H,5H)-Pyrimidinetrione, 1,3-diethyl-	155968	032479-73-5	58
54	42.620	0.80	D:\Database\W10N14.L			
			1,1,7-TRIMETHYL-4-METHYLENEDECAHYDRO-1H-CYCLOPROPA[E]AZULEN-7-OL	267795	077171-55-2	98
			1H-Cycloprop[e]azulen-7-ol, decahydro-1,1,7-trimethyl-4-methylene-, [1ar-(1a.alpha.,4a.alpha.,7.beta., 7a.beta.,7b.alpha.)]-	267797	006750-60-3	98
			(-)-Spathulenol	267796	077171-55-2	93
55	42.865	2.71	D:\Database\W10N14.L			
			(-)-5-Oxatricyclo[8.2.0.0(4,6)]dodecane,,12-trimethyl-9-methylene-, [1R-(1R*,4R*,6R*,10S*)]-	267388	001139-30-6	99
			Caryophyllene oxide	267391	001139-30-6	99
			(-)-5-Oxatricyclo[8.2.0.0(4,6)]dodecane,,12-trimethyl-9-methylene-, [1R-(1R*,4R*,6R*,10S*)]-	267387	001139-30-6	95
56	43.906	0.23	D:\Database\W10N14.L			
			(1R,3E,7E,11R)-1,5,5,8-Tetramethyl-12-oxabicyclo[9.1.0]dodeca-3,7-diene	267258	019888-34-7	99
			(1R,3E,7E,11R)-1,5,5,8-Tetramethyl-12-oxabicyclo[9.1.0]dodeca-3,7-diene	267257	019888-34-7	87
			Naphthalene, decahydro-, cis-	52317	000493-01-6	78
57	44.717	0.27	D:\Database\W10N14.L			
			isospathulenol	267807	2000267-80-7	99
			(-)-Spathulenol	267798	077171-55-2	86
			Isospathulenol	267808	088395-46-4	62
58	44.946	0.44	D:\Database\W10N14.L			
			caryophylla-4(12),8(13)-dien-5.beta.-ol	267136	2000267-13-6	98
			10,10-Dimethyl-2,6-dimethylenebicyclo[7.2.0]undecan-5.beta.-ol	267897	019431-80-2	98
			10,10-Dimethyl-2,6-dimethylenebicyclo[7.2.0]undecan-5.beta.-ol	267895	019431-80-2	96

Data Path : D:\msdchem\1\data\
Data File : Karimnezhad 3.D
Acq On : 15 Mar 2022 8:52
Operator : Jafari
Sample : M12
Misc :
ALS Vial : 31 Sample Multiplier: 1

Search Libraries: D:\Database\W10N14.L Minimum Quality: 0

Unknown Spectrum: Apex
Integration Events: ChemStation Integrator - events.e

Pk#	RT	Area%	Library/ID	Ref#	CAS#	Qual
59	45.603	0.34	D:\Database\W10N14.L			
			Presilphiperfolane-9,15-epoxide	267660	2000267-66-0	95
			caryophylla-3,8(13)-dien-5.beta.-o	267103	2000267-10-3	64
			(-)-5-Oxatricyclo[8.2.0.0(4,6)]dod ecane,,12-trimethyl-9-methylene-, [1R-(1R*,4R*,6R*,10S*)]-	267389	001139-30-6	62
60	46.158	0.47	D:\Database\W10N14.L			
			Bicyclo[7.2.0]undec-3-en-5-ol, 4,1 1,11-trimethyl-8-methylene-, stere oisomer	267841	032214-89-4	90
			Presilphiperfolane-9,15-epoxide	267660	2000267-66-0	83
			(-)-5-Oxatricyclo[8.2.0.0(4,6)]dod ecane,,12-trimethyl-9-methylene-, [1R-(1R*,4R*,6R*,10S*)]-	267389	001139-30-6	83
61	46.929	0.21	D:\Database\W10N14.L			
			12-Norcyercene-B	265281	2000265-28-1	90
			7R,8R-8-Hydroxy-4-isopropylidene-7 -methylbicyclo[5.3.1]undec-1-ene	267570	161362-94-3	49
			VULGAROL B	267978	011056-03-4	43
62	51.924	0.15	D:\Database\W10N14.L			
			2-Pentadecanone, 6,10,14-trimethyl	427982	000502-69-2	99
			2-Pentadecanone, 6,10,14-trimethyl	427988	000502-69-2	94
			2-Pentadecanone, 6,10,14-trimethyl	427987	000502-69-2	94
63	55.942	0.23	D:\Database\W10N14.L			
			n-Hexadecanoic acid	387914	000057-10-3	99
			n-Hexadecanoic acid	387919	000057-10-3	99
			Myristic acid	295117	000544-63-8	97
64	57.480	0.23	D:\Database\W10N14.L			
			1H-Naphtho[2,1-b]pyran, 3-ethenyld odecahydro-3,4a,7,7,10a-pentamethy l-, [3R-(3.alpha.,4a.beta.,6a.alph a.,10a.beta.,10b.alpha.)]-	500325	000596-84-9	99
			1H-Naphtho[2,1-b]pyran, 3-ethenyld odecahydro-3,4a,7,7,10a-pentamethy l-, [3R-(3.alpha.,4a.beta.,6a.alph a.,10a.beta.,10b.alpha.)]-	500323	000596-84-9	94
			1H-Naphtho[2,1-b]pyran, 3-ethenyld odecahydro-3,4a,7,7,10a-pentamethy	500327	000596-84-9	94

Library Search Report

Data Path : D:\msdchem\1\data\
Data File : Karimnezhad 3.D
Acq On : 15 Mar 2022 8:52
Operator : Jafari
Sample : M12
Misc :
ALS Vial : 31 Sample Multiplier: 1

Search Libraries: D:\Database\W10N14.L Minimum Quality: 0

Unknown Spectrum: Apex
Integration Events: ChemStation Integrator - events.e

Pk#	RT	Area%	Library/ID	Ref#	CAS#	Qual
			l-, [3R-(3.alpha.,4a.beta.,6a.alph a.,10a.beta.,10b.alpha.)]-			
