# Supplementary material for: Comparison of quantity, quality and antibacterial activity of essential oil Mentha longifolia (L.) L. under different traditional and modern extraction methods
Source: PLoS One. 2024 Jul 10;19(7):e0301558. doi: 10.1371/journal.pone.0301558 (PMC11236116; doi:10.1371/journal.pone.0301558)
Supplement: S2 File — (ZIP) [file pone.0301558.s002.zip › Karimnezhad/M12/QualKarimnezhad 3.pdf]

Library Search Report

Data Path : D:\msdchem\1\data\
Data File : Karimnezhad 3.D
Acq On : 15 Mar 2022 8:52
Operator : Jafari
Sample : M12
Misc :
ALS Vial : 31 Sample Multiplier: 1

Search Libraries: D:\Database\W10N14.L Minimum Quality: 0

Unknown Spectrum: Apex
Integration Events: ChemStation Integrator - events.e

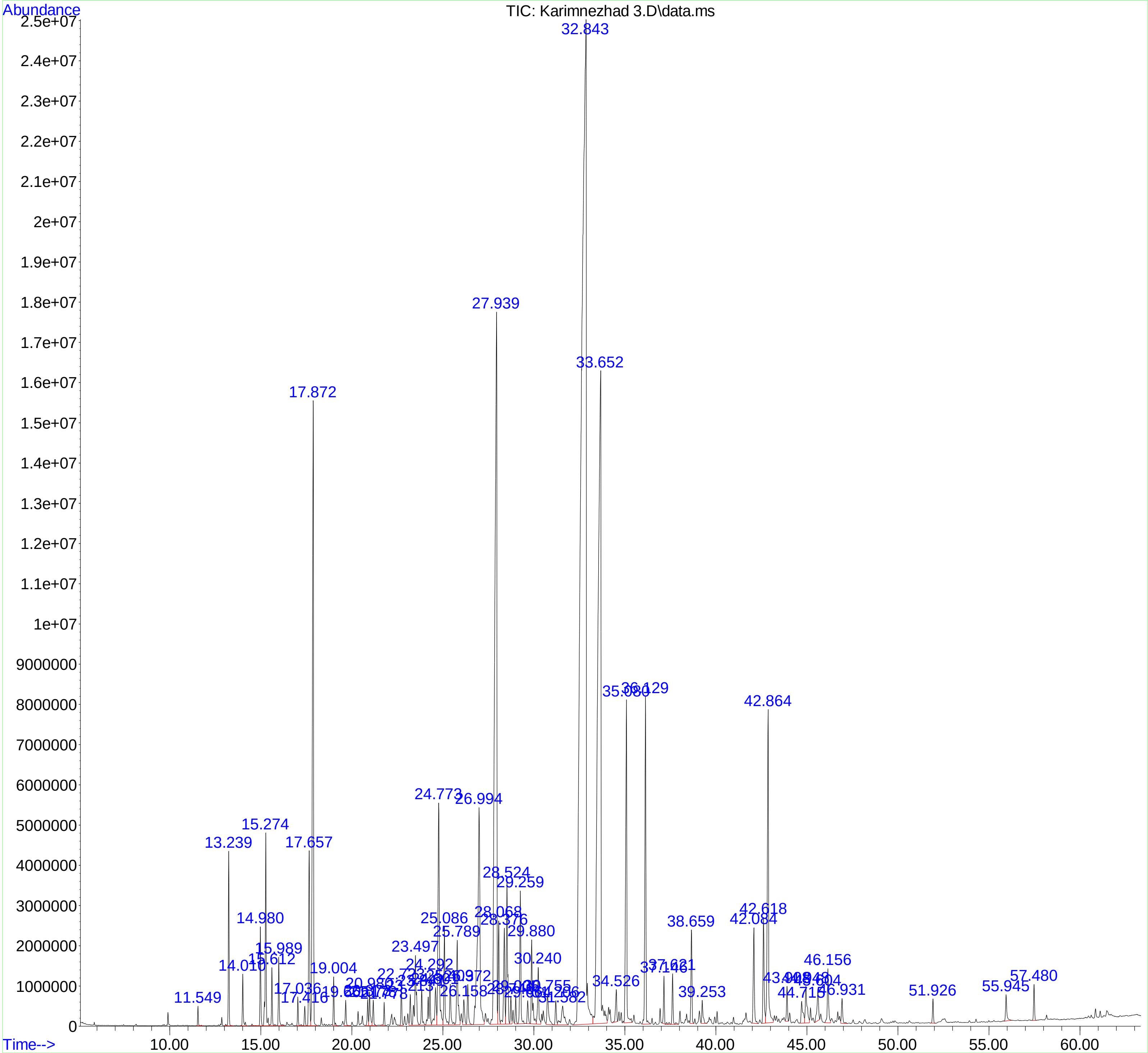

Library Search Report - ChemStation Integrator

Unknown Spectrum based on Apex

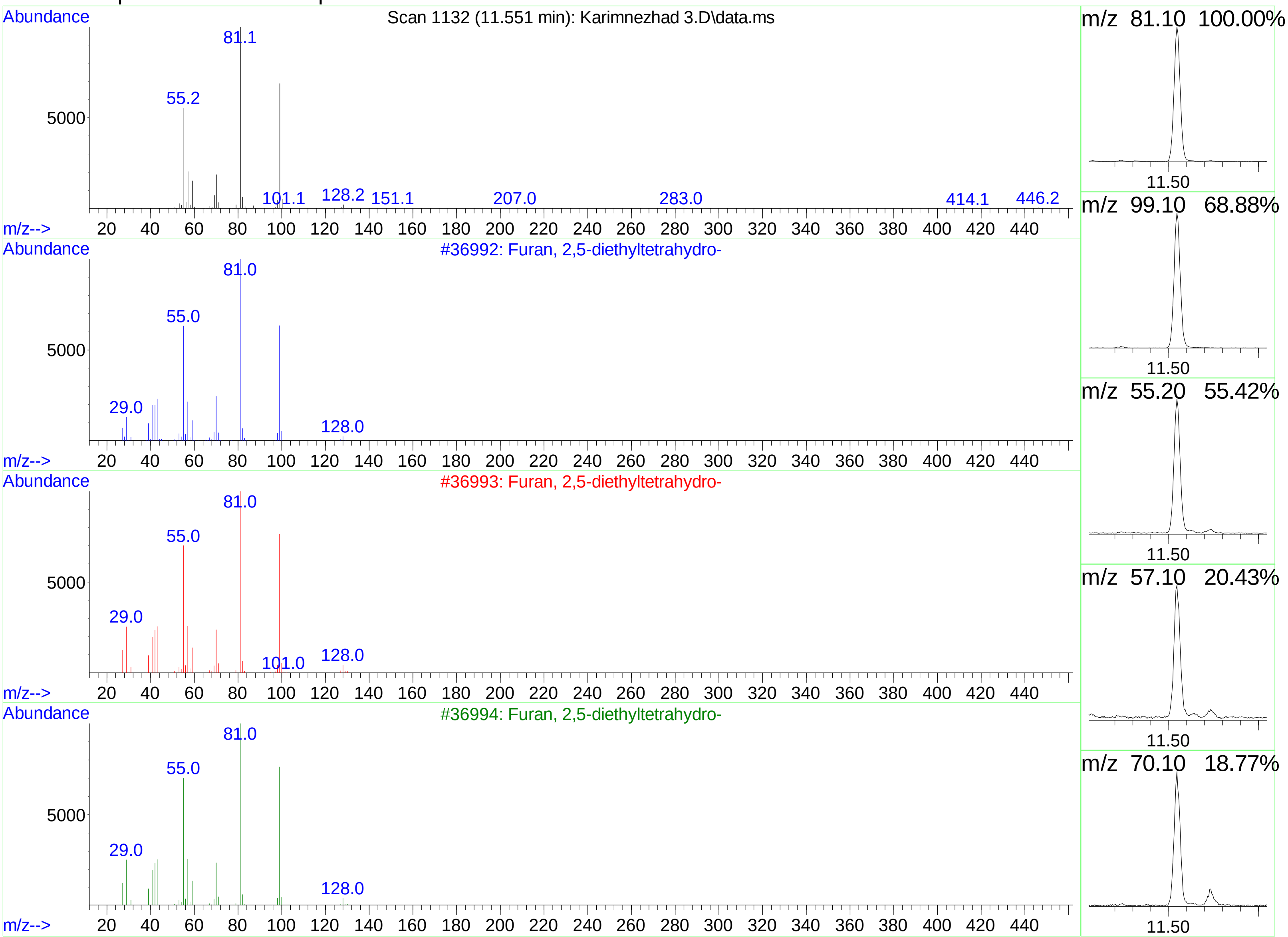

Data File: D:\msdchem\1\data\Karimnezhad 3.D
Sample : M12

Peak Number: 1 at 11.551 min Area: 12640509 Area % 0.09

The 3 best hits from each library.	Ref\#	CAS\#	Qual
D:\Database\W10N14.L			
1 Furan, 2,5-diethyltetrahydro-	36992	041239-48-9	95
2 Furan, 2,5-diethyltetrahydro-	36993	041239-48-9	78
3 Furan, 2,5-diethyltetrahydro-	36994	041239-48-9	78

Unknown Spectrum based on Apex

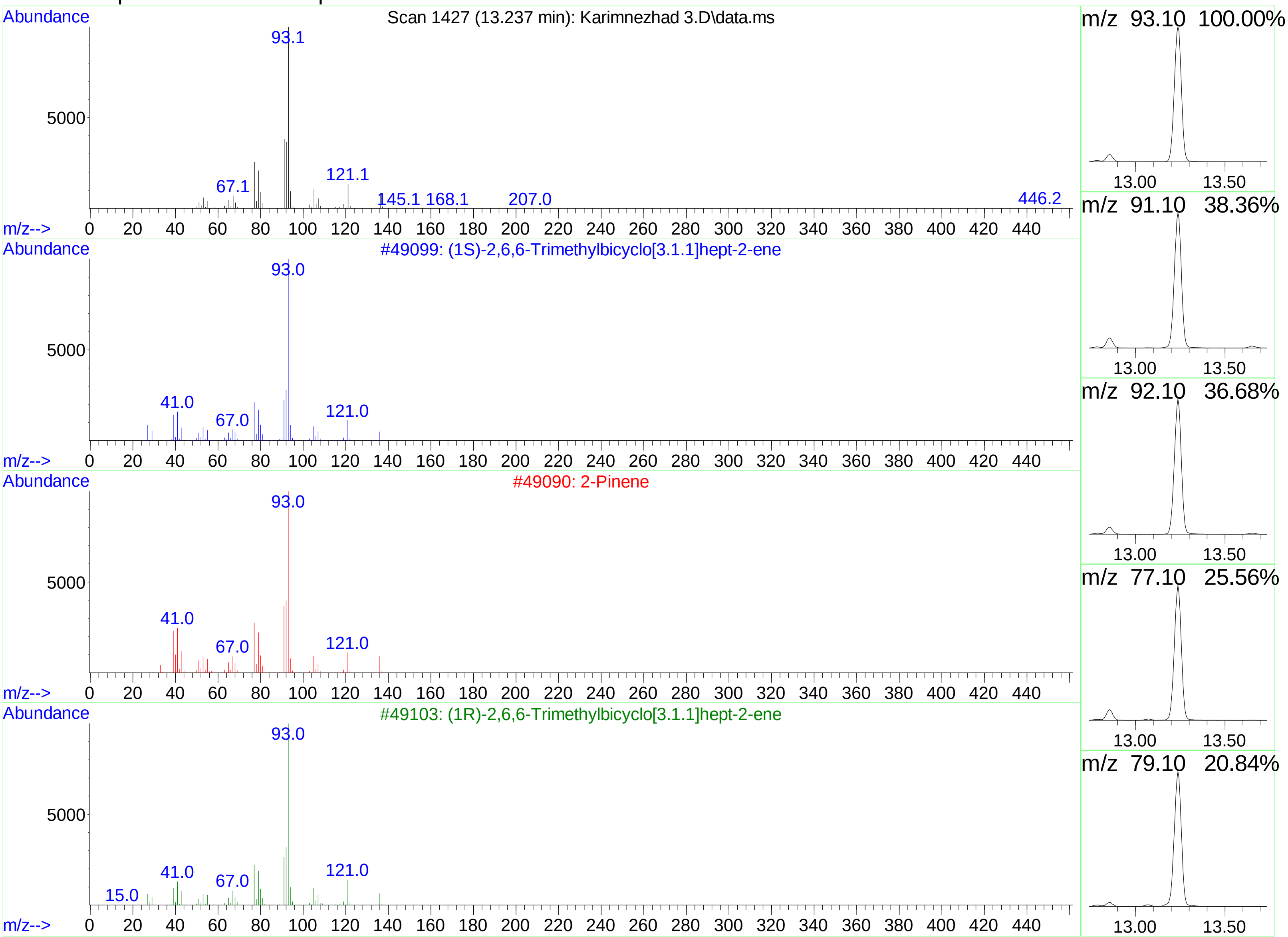

Data File: D:\msdchem\1\data\Karimnezhad 3.D
Sample : M12

Peak Number: 2 at 13.237 min Area: 120874782 Area % 0.88

The 3 best hits from each library. Ref\# CAS\# Qual

D:\Database\W10N14.L

1	(1S)-2,6,6-Trimethylbicyclo[3.1....	49099	007785-26-4	96
2	2-Pinene	49090	000080-56-8	96
3	(1R)-2,6,6-Trimethylbicyclo[3.1....	49103	007785-70-8	96

Unknown Spectrum based on Apex

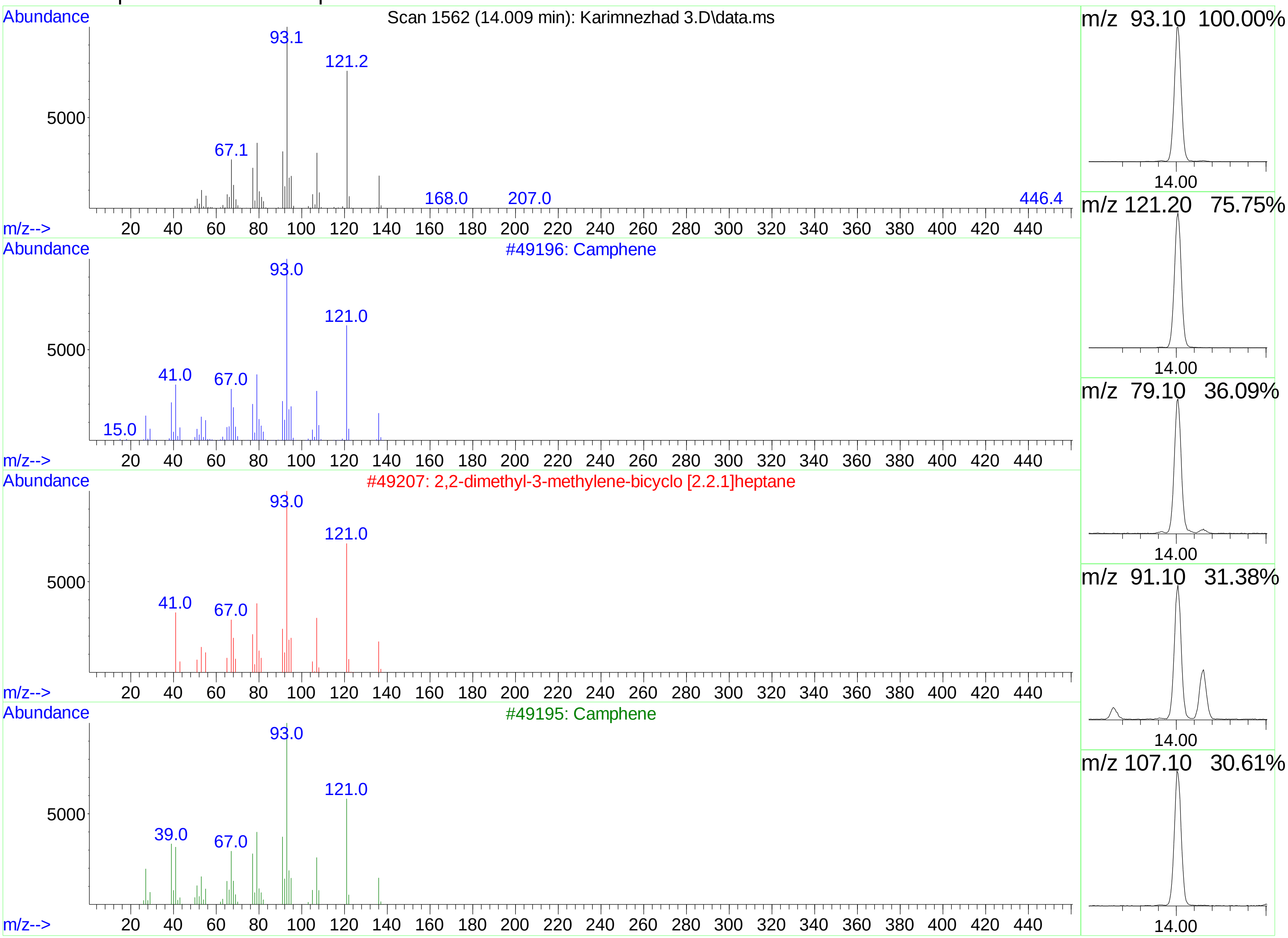

Data File: D:\msdchem\1\data\Karimnezhad 3.D
Sample : M12

Peak Number: 3 at 14.009 min Area: 39545847 Area % 0.29

The 3 best hits from each library.	Ref\#	CAS\#	Qual
D:\Database\W10N14.L			
1 Camphene	49196	000079-92-5	97
2 2,2-dimethyl-3-methylene-bicyclo...	49207	000079-92-5	97
3 Camphene	49195	000079-92-5	96

Library Search Report - ChemStation Integrator

Unknown Spectrum based on Apex

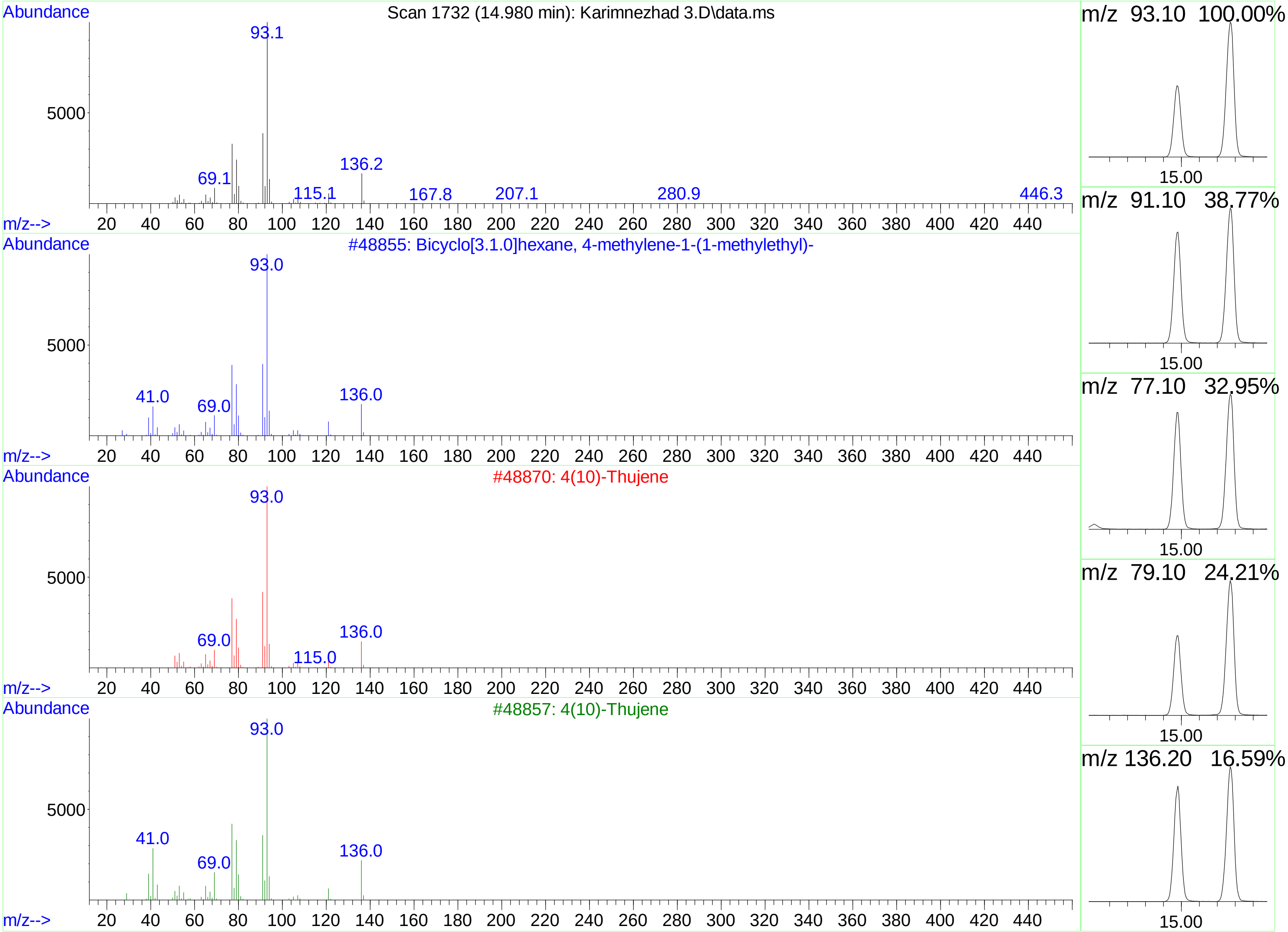

Data File: D:\msdchem\1\data\Karimnezhad 3.D
Sample : M12

Peak Number: 4 at 14.980 min Area: 75017457 Area % 0.55

The 3 best hits from each library.	Ref#	CAS#	Qual
D:\Database\W10N14.L			
1 Bicyclo[3.1.0]hexane, 4-methylen...	48855	003387-41-5	96
2 4(10)-Thujene	48870	003387-41-5	96
3 4(10)-Thujene	48857	003387-41-5	96

Library Search Report - ChemStation Integrator

Unknown Spectrum based on Apex

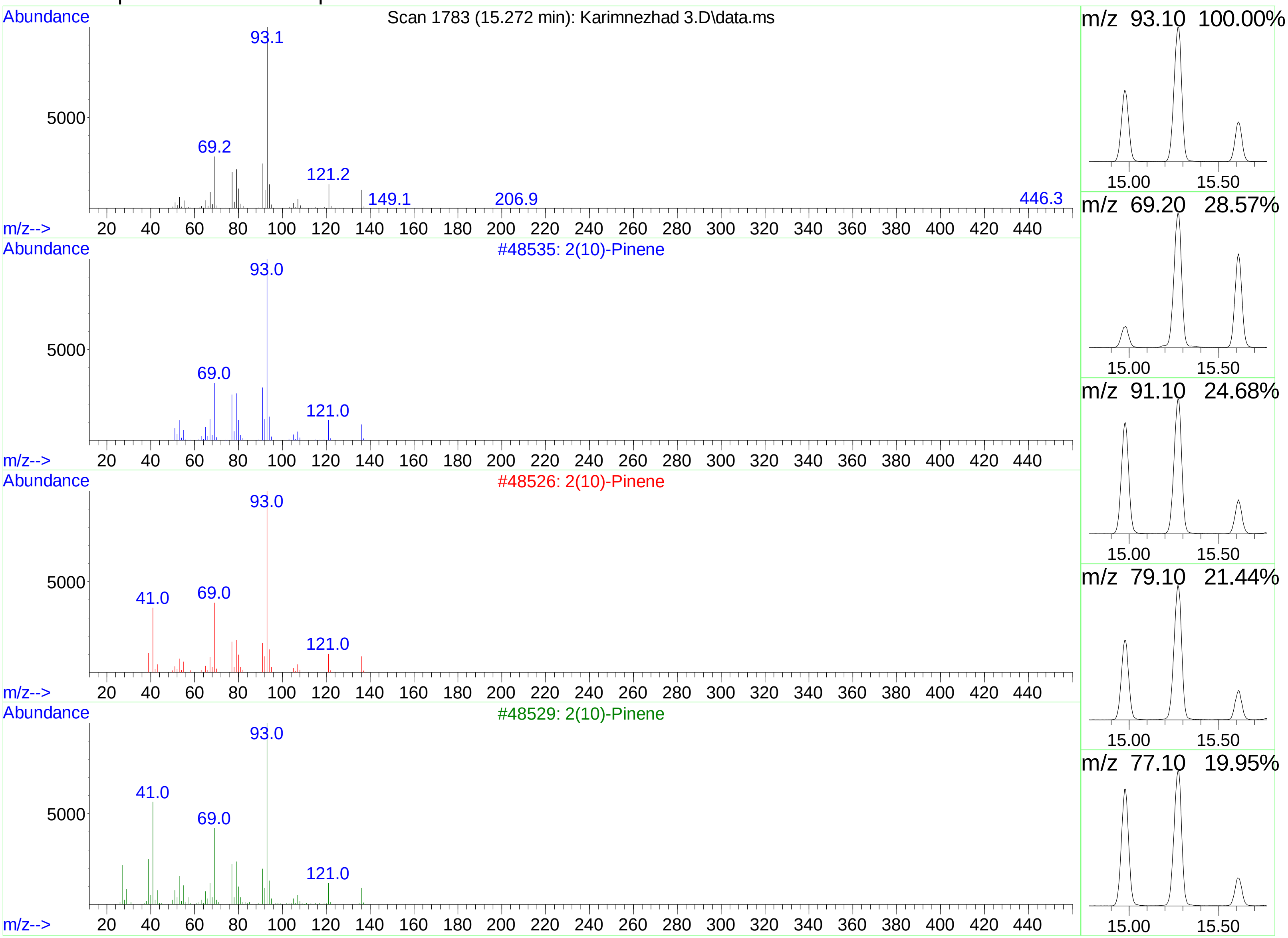

Data File: D:\msdchem\1\data\Karimnezhad 3.D
Sample : M12

Peak Number: 5 at 15.272 min Area: 167693649 Area % 1.22

The 3 best hits from each library.	Ref\#	CAS\#	Qual
D:\Database\W10N14.L			
1 2(10)-Pinene	48535	000127-91-3	94
2 2(10)-Pinene	48526	000127-91-3	94
3 2(10)-Pinene	48529	000127-91-3	94

Unknown Spectrum based on Apex

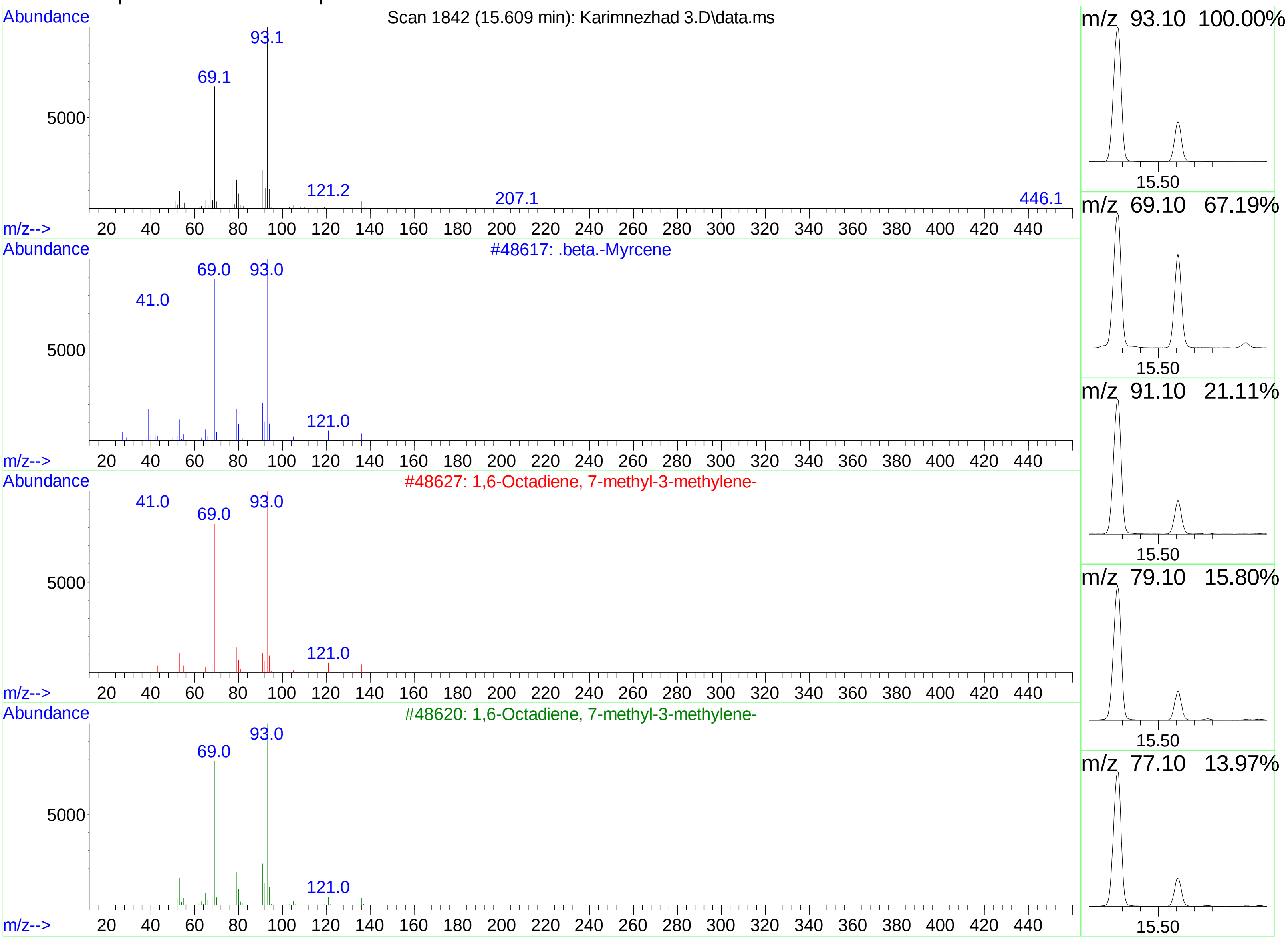

Data File: D:\msdchem\1\data\Karimnezhad 3.D
Sample : M12

Peak Number: 6 at 15.609 min Area: 43160983 Area % 0.31

The 3 best hits from each library.	Ref#	CAS#	Qual
D:\Database\W10N14.L			
1 .beta.-Myrcene	48617	000123-35-3	96
2 1,6-Octadiene, 7-methyl-3-methyl...	48627	000123-35-3	95
3 1,6-Octadiene, 7-methyl-3-methyl...	48620	000123-35-3	94

Unknown Spectrum based on Apex

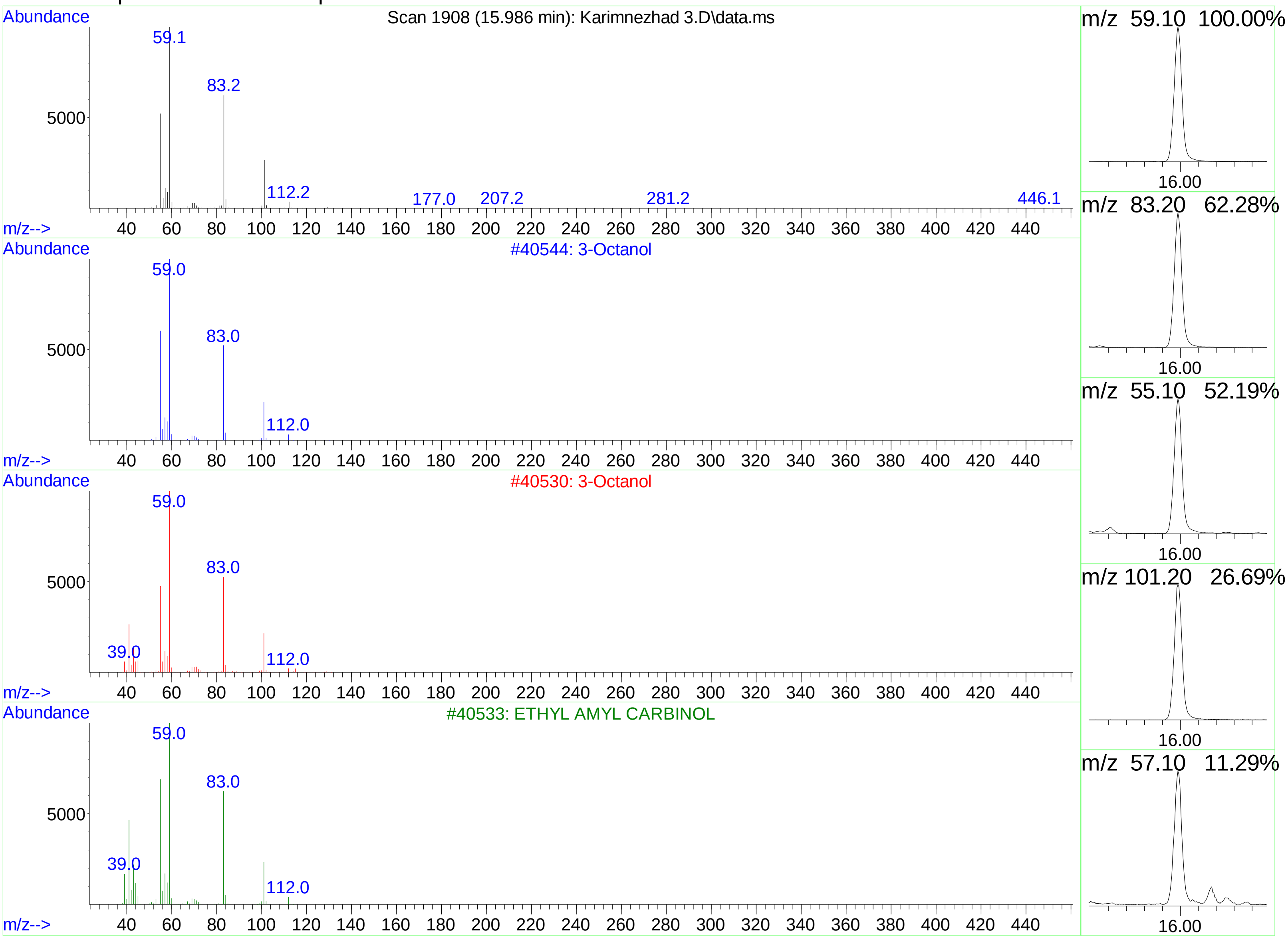

Data File: D:\msdchem\1\data\Karimnezhad 3.D
Sample : M12

Peak Number: 7 at 15.986 min Area: 54507847 Area % 0.40

The 3 best hits from each library.	Ref#	CAS#	Qual
D:\Database\W10N14.L			
1 3-Octanol	40544	000589-98-0	90
2 3-Octanol	40530	000589-98-0	83
3 ETHYL AMYL CARBINOL	40533	000589-98-0	83

Unknown Spectrum based on Apex

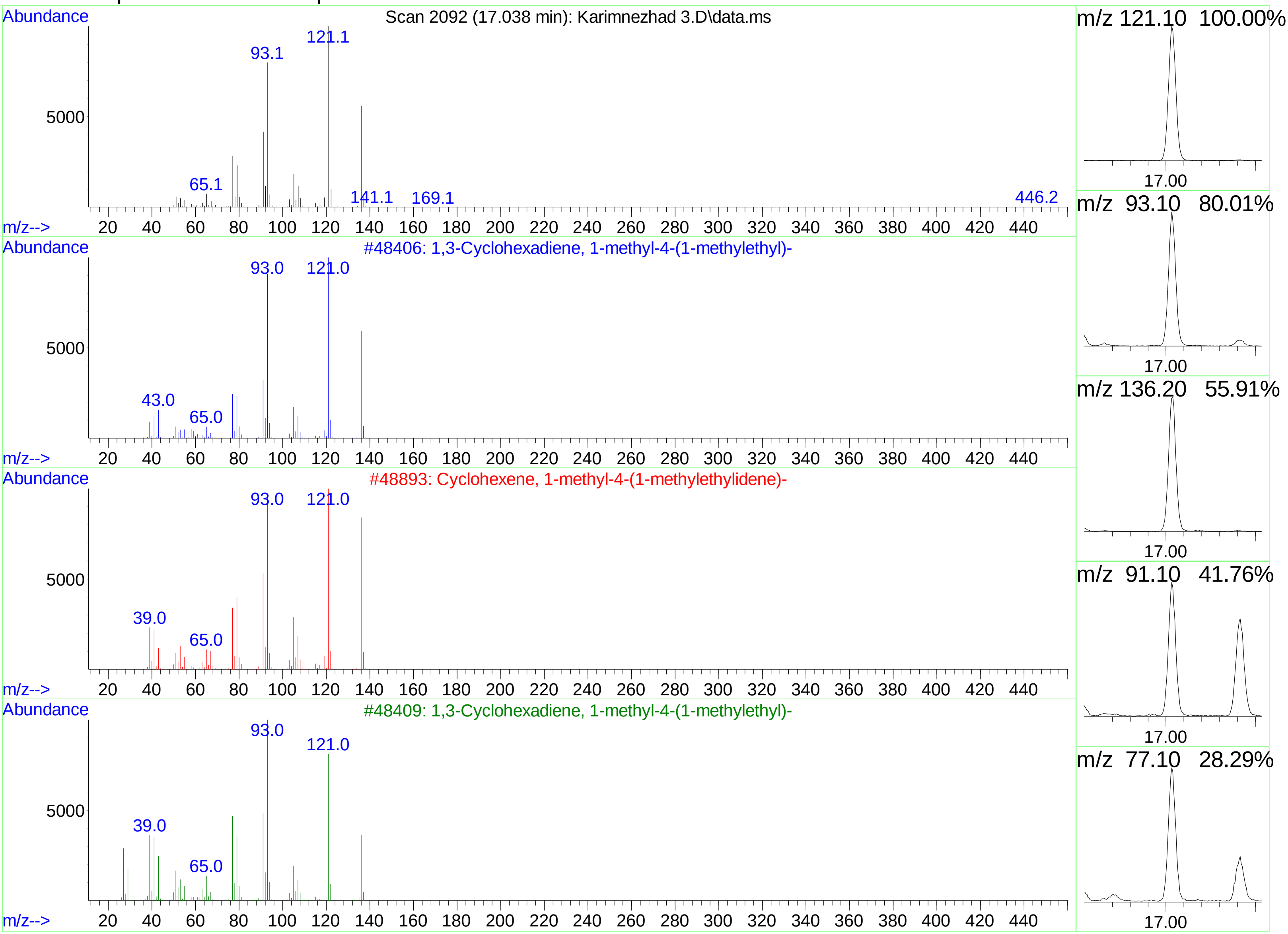

Data File: D:\msdchem\1\data\Karimnezhad 3.D
Sample : M12

Peak Number: 8 at 17.038 min Area: 21792554 Area % 0.16

The 3 best hits from each library.	Ref#	CAS#	Qual
D:\Database\W10N14.L			
1 1,3-Cyclohexadiene, 1-methyl-4-(...	48406	000099-86-5	98
2 Cyclohexene, 1-methyl-4-(1-methy...	48893	000586-62-9	97
3 1,3-Cyclohexadiene, 1-methyl-4-(...	48409	000099-86-5	97

Unknown Spectrum based on Apex

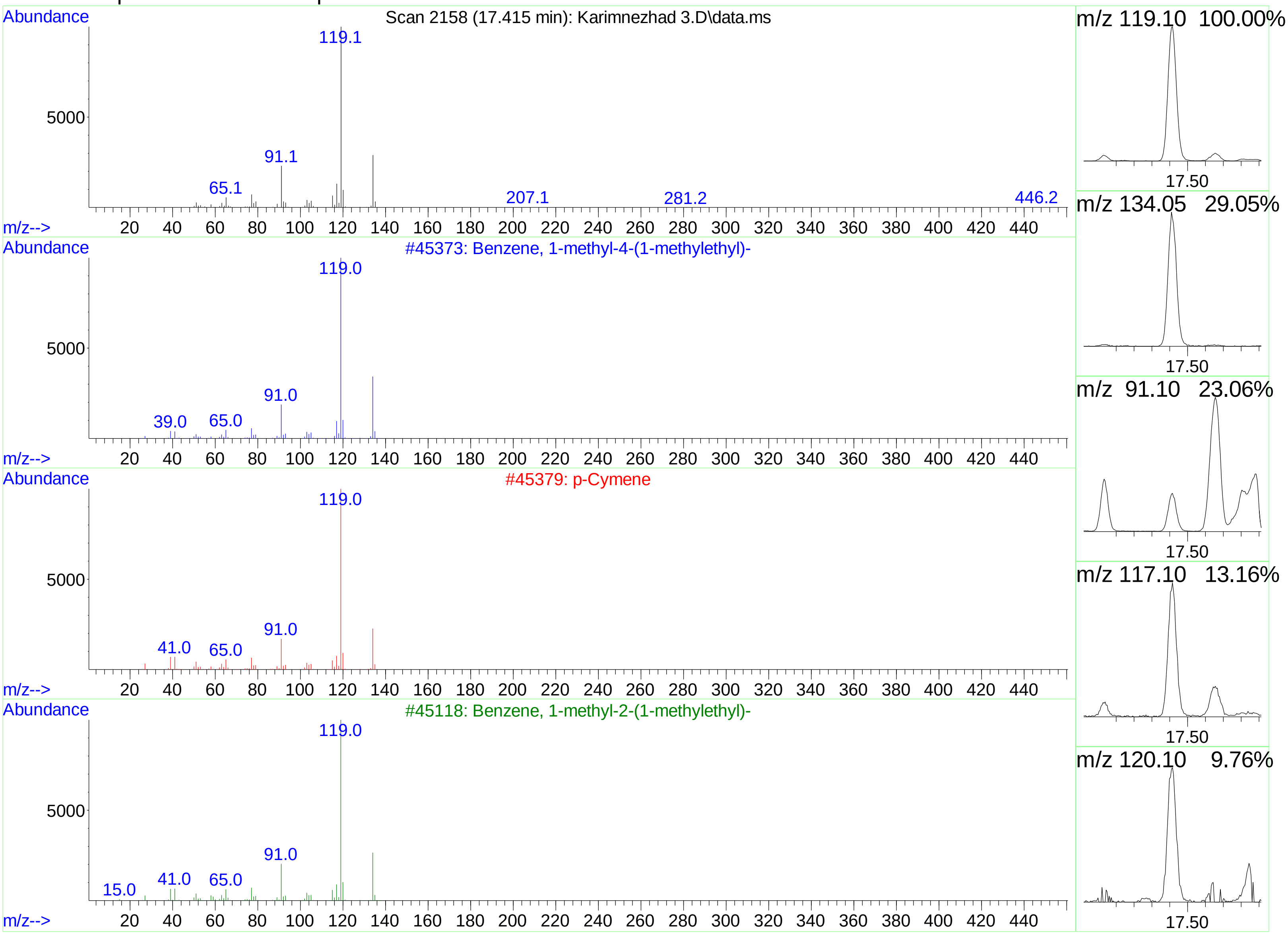

Data File: D:\msdchem\1\data\Karimnezhad 3.D
Sample : M12

Peak Number: 9 at 17.415 min Area: 16472360 Area % 0.12

The 3 best hits from each library. Ref\# CAS\# Qual

D:\Database\W10N14.L

1 Benzene, 1-methyl-4-(1-methyleth... 45373 000099-87-6 97
2 p-Cymene 45379 000099-87-6 97
3 Benzene, 1-methyl-2-(1-methyleth... 45118 000527-84-4 95

Library Search Report - ChemStation Integrator

Unknown Spectrum based on Apex

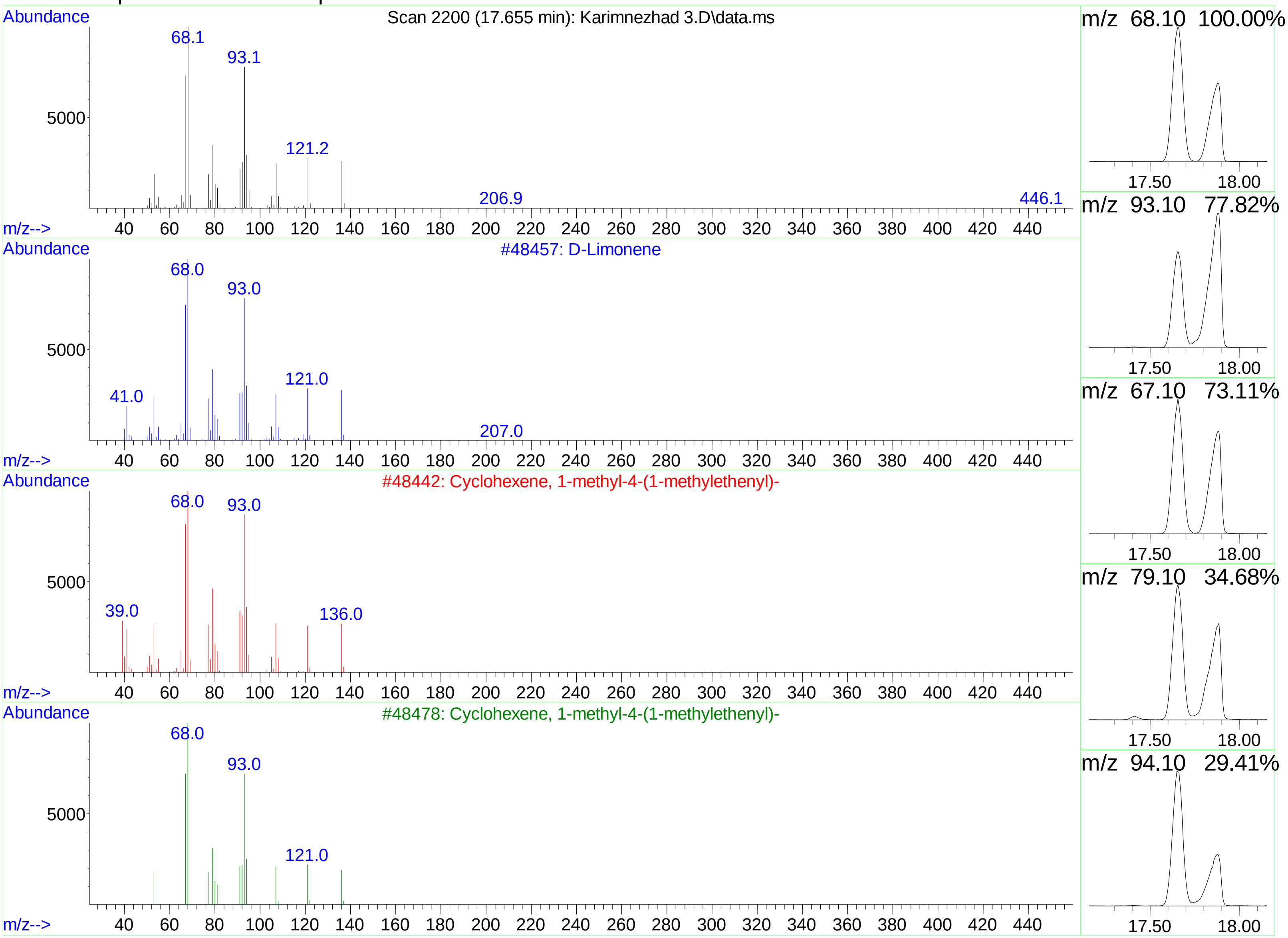

Data File: D:\msdchem\1\data\Karimnezhad 3.D
Sample : M12

Peak Number: 10 at 17.655 min Area: 173671017 Area % 1.26

The 3 best hits from each library.	Ref#	CAS#	Qual
D:\Database\W10N14.L			
1 D-Limonene	48457	005989-27-5	99
2 Cyclohexene, 1-methyl-4-(1-methy...	48442	000138-86-3	98
3 Cyclohexene, 1-methyl-4-(1-methy...	48478	000138-86-3	98

Unknown Spectrum based on Apex

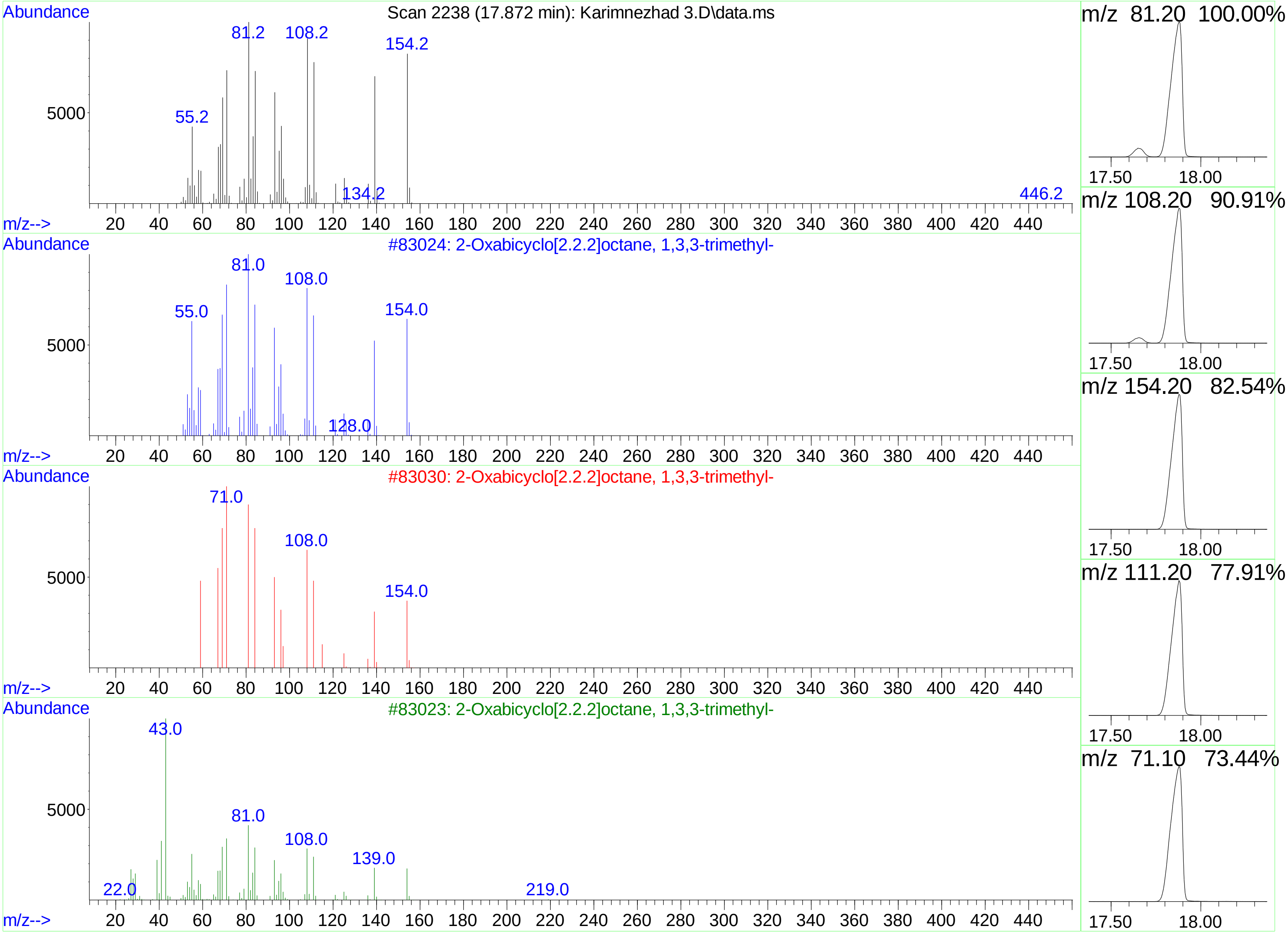

Data File: D:\msdchem\1\data\Karimnezhad 3.D
Sample : M12

Peak Number: 11 at 17.872 min Area: 672712090 Area % 4.90

The 3 best hits from each library.	Ref#	CAS#	Qual
D:\Database\W10N14.L			
1 2-Oxabicyclo[2.2.2]octane, 1,3,3...	83024	000470-82-6	98
2 2-Oxabicyclo[2.2.2]octane, 1,3,3...	83030	000470-82-6	98
3 2-Oxabicyclo[2.2.2]octane, 1,3,3...	83023	000470-82-6	97

Library Search Report - ChemStation Integrator

Unknown Spectrum based on Apex

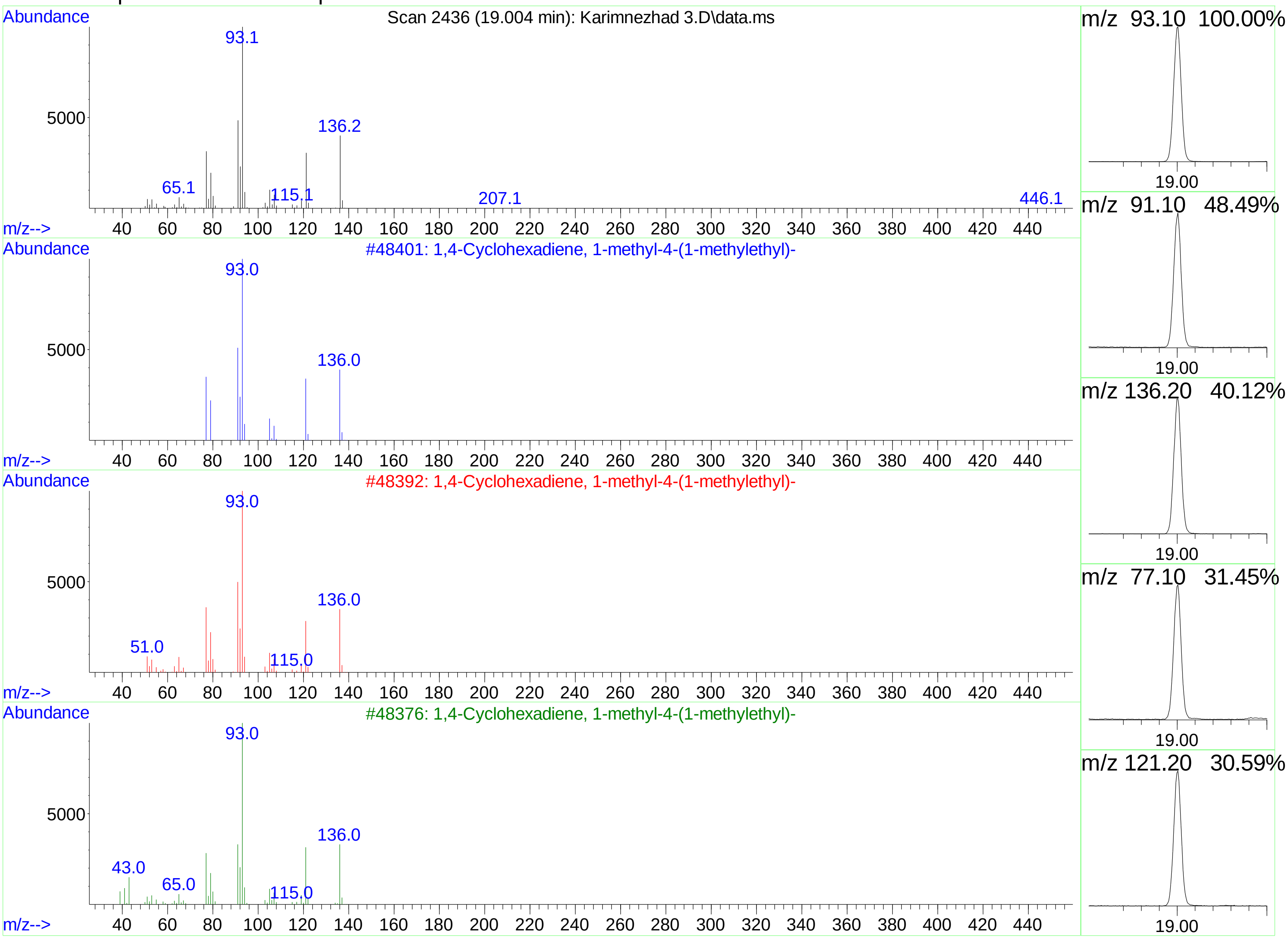

Data File: D:\msdchem\1\data\Karimnezhad 3.D
Sample : M12

Peak Number: 12 at 19.004 min Area: 37346258 Area % 0.27

The 3 best hits from each library.	Ref#	CAS#	Qual
D:\Database\W10N14.L			
1 1,4-Cyclohexadiene, 1-methyl-4-(...	48401	000099-85-4	97
2 1,4-Cyclohexadiene, 1-methyl-4-(...	48392	000099-85-4	96
3 1,4-Cyclohexadiene, 1-methyl-4-(...	48376	000099-85-4	96

Unknown Spectrum based on Apex

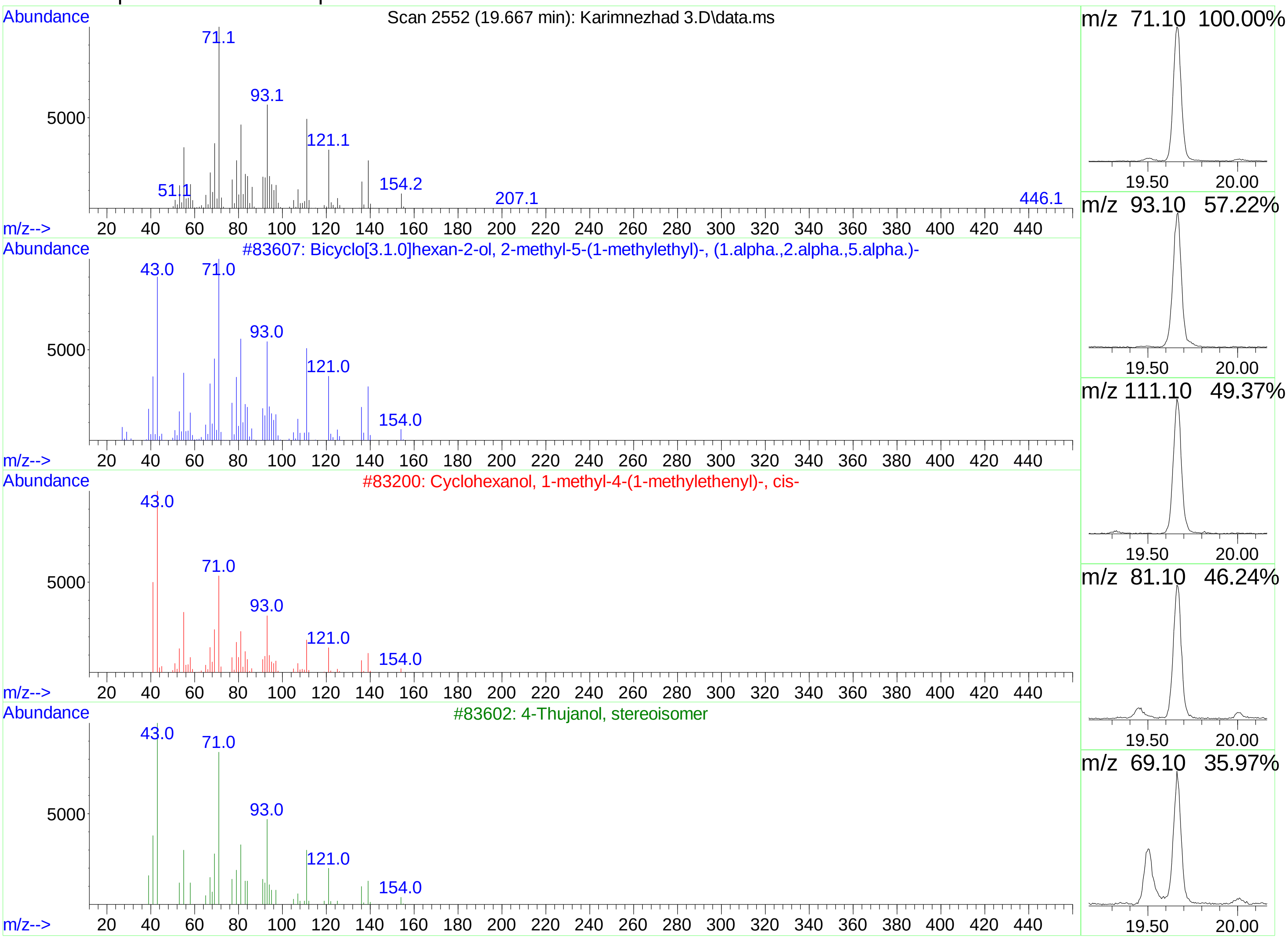

Data File: D:\msdchem\1\data\Karimnezhad 3.D
Sample : M12

Peak Number: 13 at 19.667 min Area: 22410862 Area % 0.16

The 3 best hits from each library.	Ref\#	CAS\#	Qual
D:\Database\W10N14.L			
1 Bicyclo[3.1.0]hexan-2-ol, 2-meth...	83607	017699-16-0	98
2 Cyclohexanol, 1-methyl-4-(1-meth...	83200	007299-41-4	96
3 4-Thujanol, stereoisomer	83602	017699-16-0	96

Library Search Report - ChemStation Integrator

Unknown Spectrum based on Apex

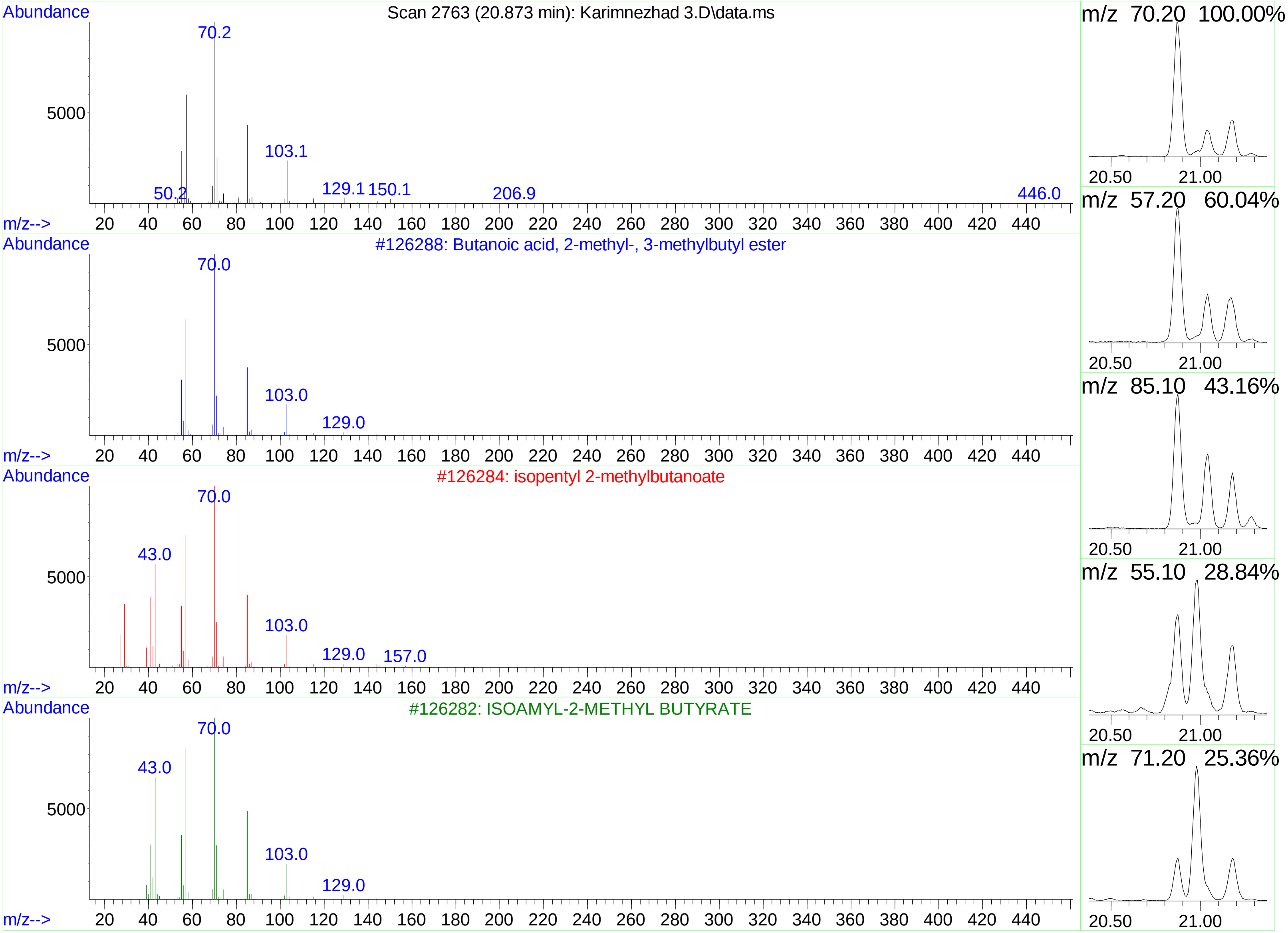

Data File: D:\msdchem\1\data\Karimnezhad 3.D
Sample : M12

Peak Number: 14 at 20.873 min Area: 21206285 Area % 0.15

The 3 best hits from each library. Ref\# CAS\# Qual

D:\Database\W10N14.L

1	Butanoic acid, 2-methyl-, 3-meth...	126288	027625-35-0	86
2	isopentyl 2-methylbutanoate	126284	027625-35-0	83
3	ISOAMYL-2-METHYL BUTYRATE	126282	027625-35-0	72

Unknown Spectrum based on Apex

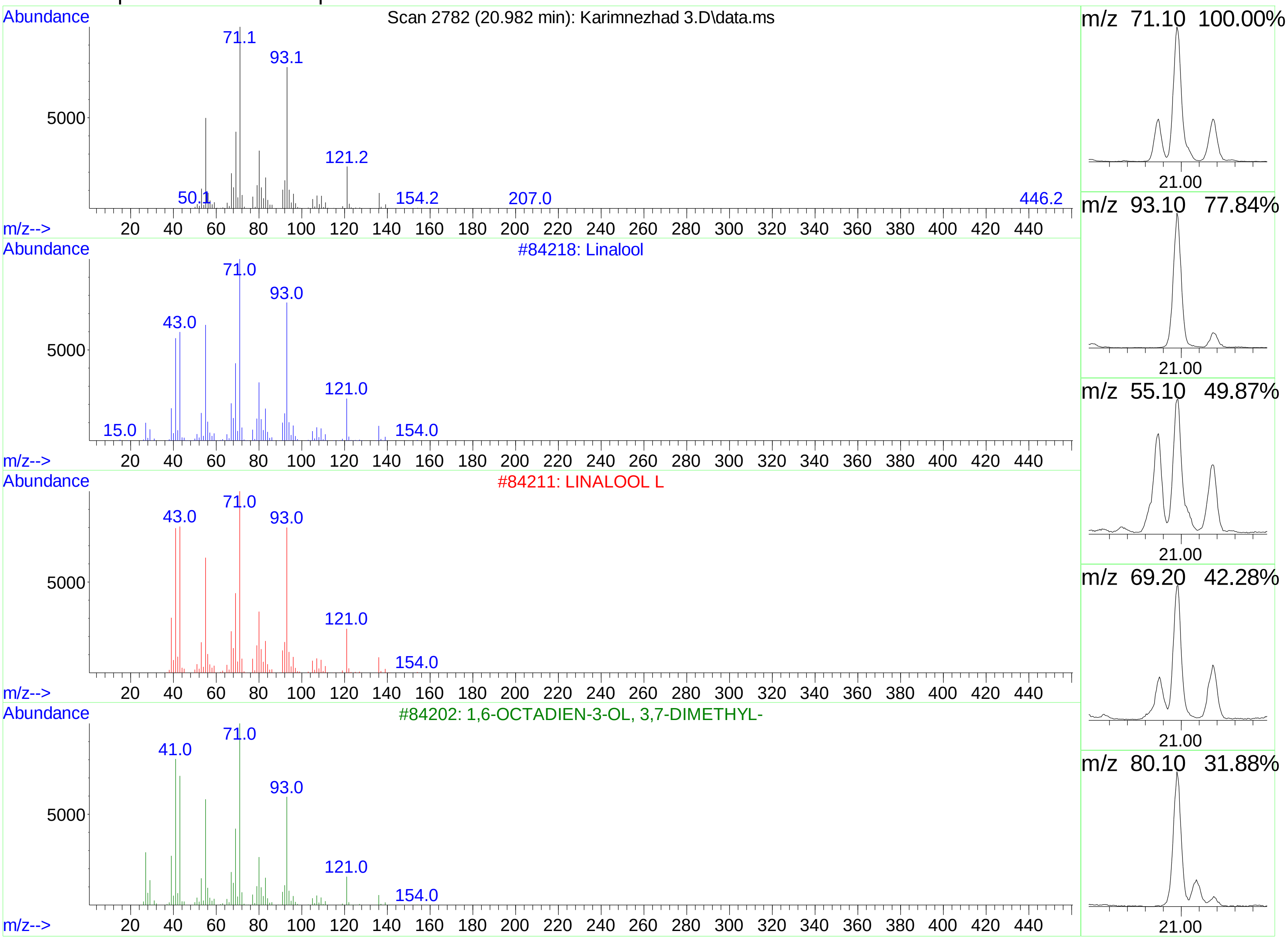

Data File: D:\msdchem\1\data\Karimnezhad 3.D
Sample : M12

Peak Number: 15 at 20.982 min Area: 34026633 Area % 0.25

The 3 best hits from each library.	Ref#	CAS#	Qual
D:\Database\W10N14.L			
1 Linalool	84218	000078-70-6	97
2 LINALOOL L	84211	000078-70-6	97
3 1,6-OCTADIEN-3-OL, 3,7-DIMETHYL-	84202	000078-70-6	96

Library Search Report - ChemStation Integrator

Unknown Spectrum based on Apex

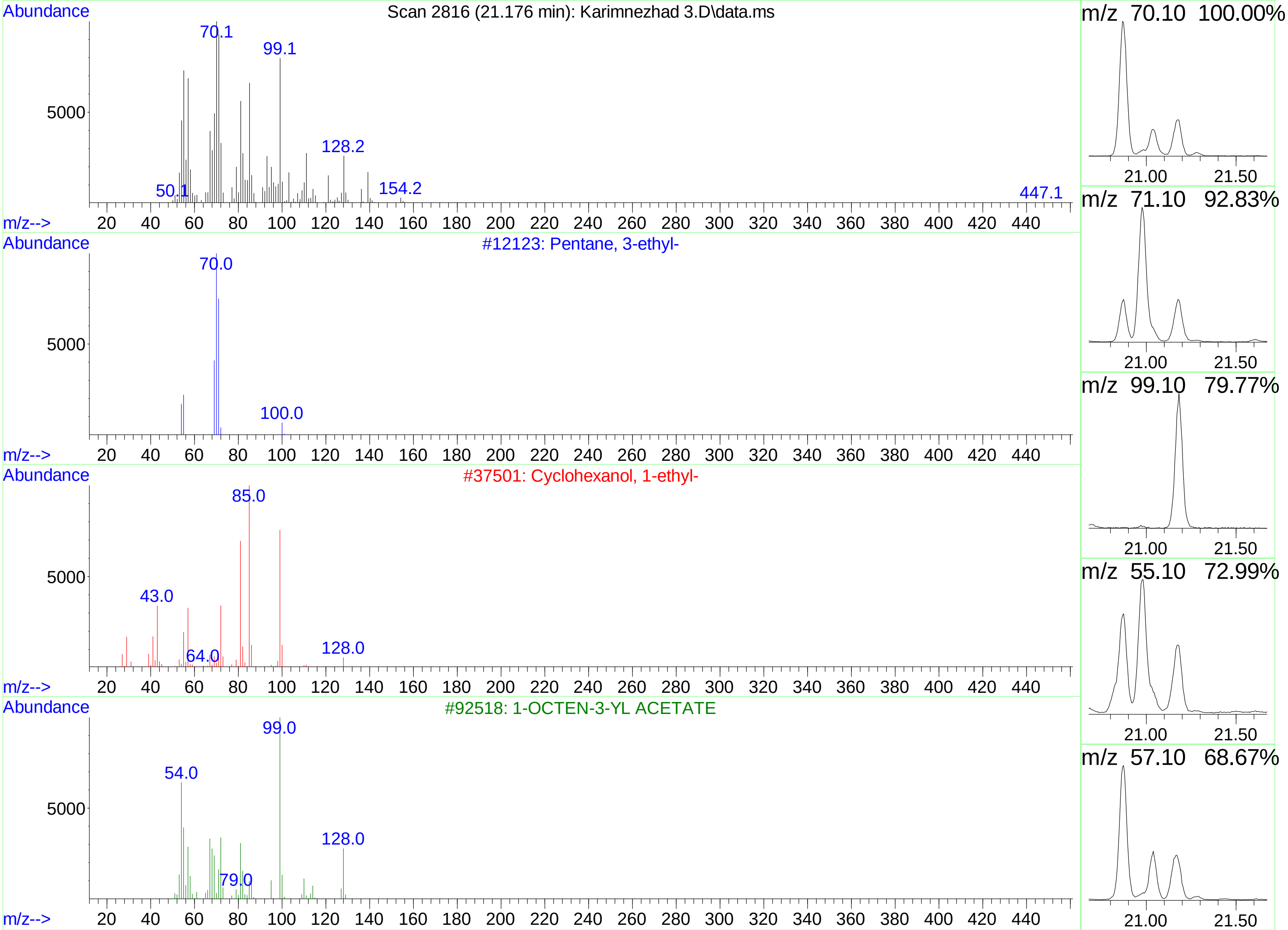

Data File: D:\msdchem\1\data\Karimnezhad 3.D
Sample : M12

Peak Number: 16 at 21.176 min Area: 23576419 Area % 0.17

The 3 best hits from each library.	Ref#	CAS#	Qual
D:\Database\W10N14.L			
1 Pentane, 3-ethyl-	12123	000617-78-7	38
2 Cyclohexanol, 1-ethyl-	37501	001940-18-7	38
3 1-OCTEN-3-YL ACETATE	92518	002442-10-6	30

Unknown Spectrum based on Apex

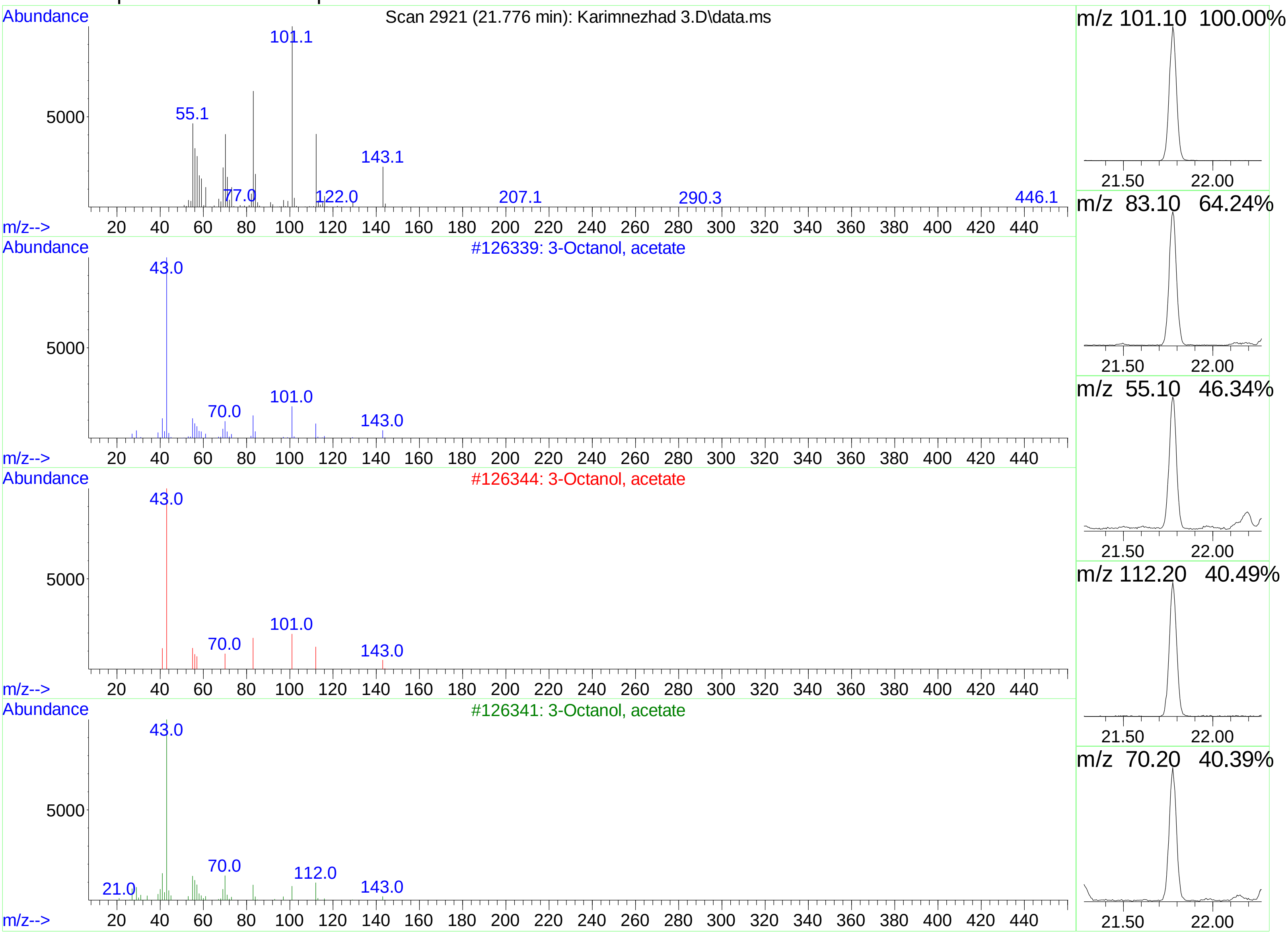

Data File: D:\msdchem\1\data\Karimnezhad 3.D
Sample : M12

Peak Number: 17 at 21.776 min Area: 17240192 Area % 0.13

The 3 best hits from each library.	Ref\#	CAS\#	Qual
D:\Database\W10N14.L			
1 3-Octanol, acetate	126339	004864-61-3	91
2 3-Octanol, acetate	126344	004864-61-3	53
3 3-Octanol, acetate	126341	004864-61-3	47

Unknown Spectrum based on Apex

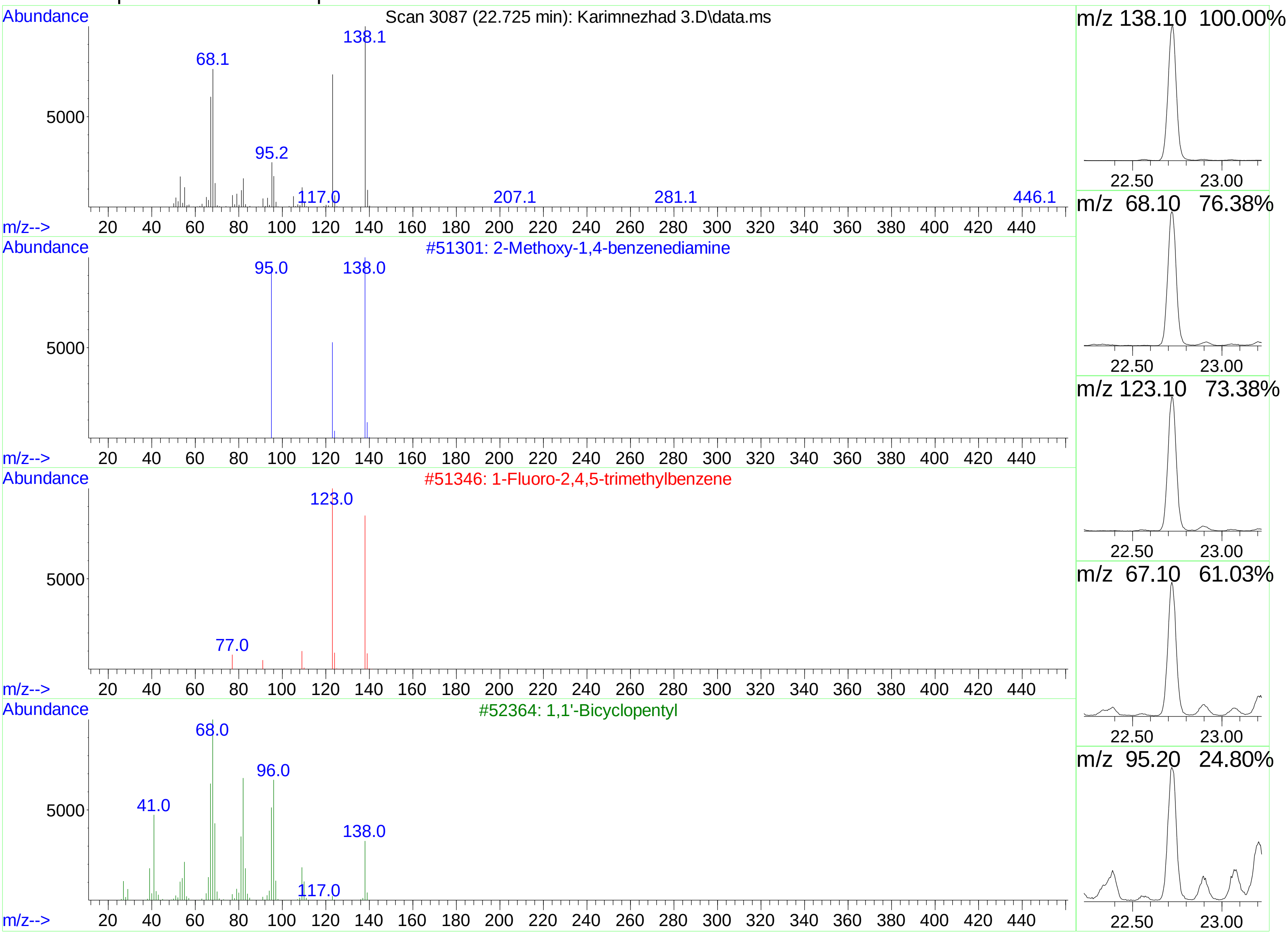

Data File: D:\msdchem\1\data\Karimnezhad 3.D
Sample : M12

Peak Number: 18 at 22.725 min Area: 35370679 Area % 0.26

The 3 best hits from each library.	Ref#	CAS#	Qual
D:\Database\W10N14.L			
1 2-Methoxy-1,4-benzenediamine	51301	005307-02-8	86
2 1-Fluoro-2,4,5-trimethylbenzene	51346	000400-01-1	72
3 1,1'-Bicyclopentyl	52364	001636-39-1	70

Library Search Report - ChemStation Integrator

Unknown Spectrum based on Apex

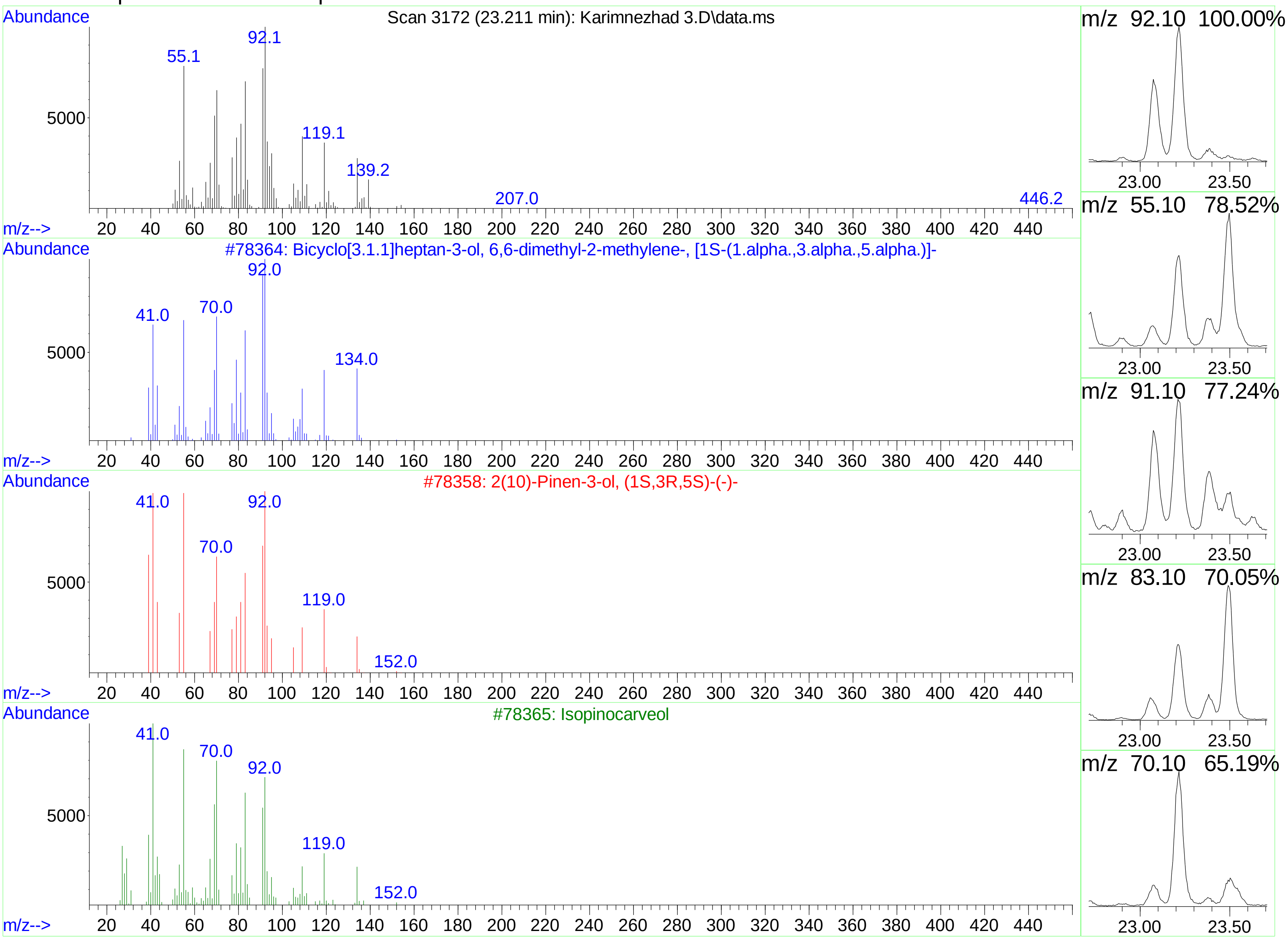

Data File: D:\msdchem\1\data\Karimnezhad 3.D
Sample : M12

Peak Number: 19 at 23.211 min Area: 29193609 Area % 0.21

The 3 best hits from each library.	Ref#	CAS#	Qual
D:\Database\W10N14.L			
1 Bicyclo[3.1.1]heptan-3-ol, 6,6-d...	78364	000547-61-5	90
2 2(10)-Pinen-3-ol, (1S,3R,5S)-(-)-	78358	000547-61-5	90
3 Isopinocarveol	78365	006712-79-4	81

Unknown Spectrum based on Apex

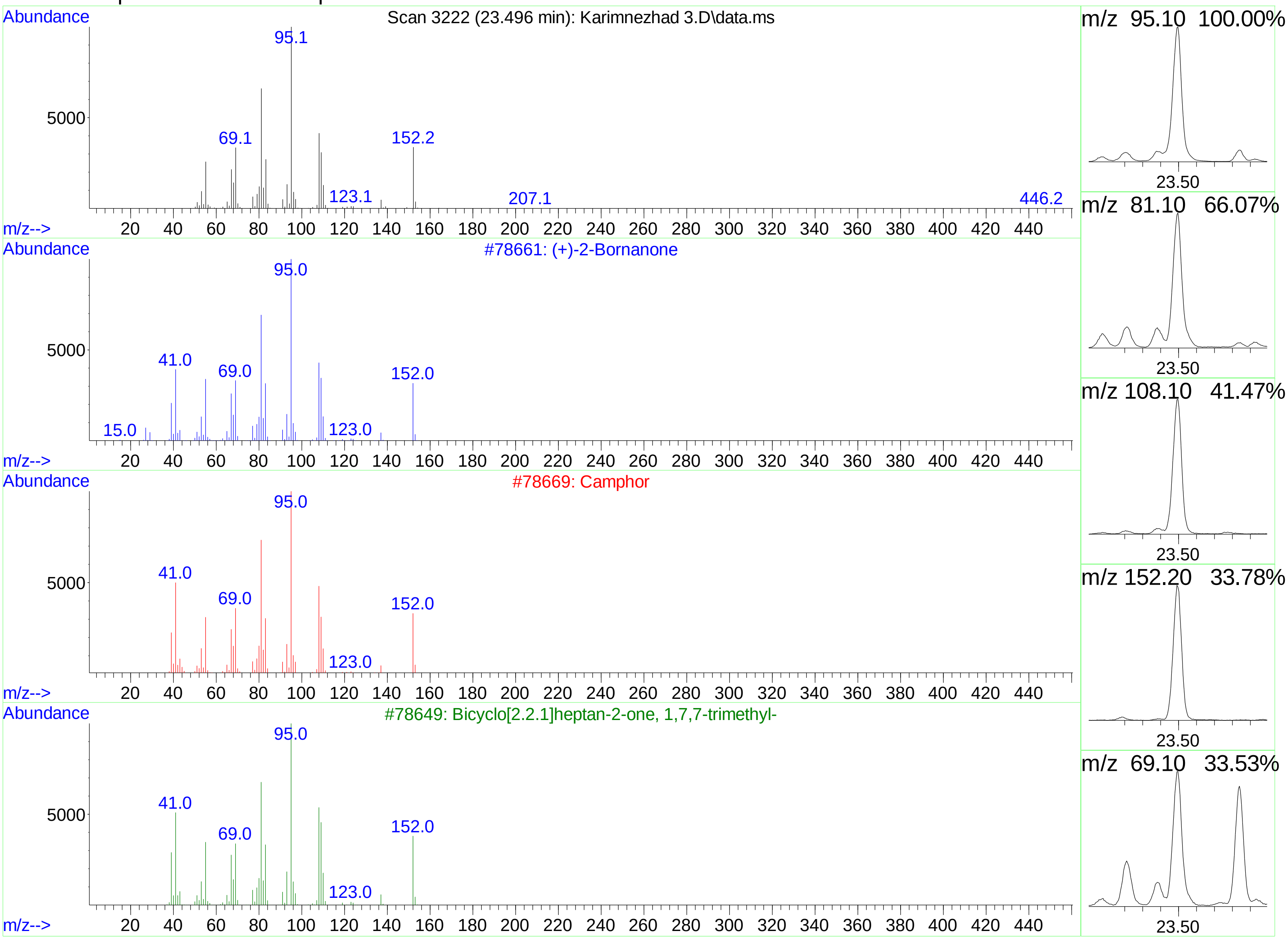

Data File: D:\msdchem\1\data\Karimnezhad 3.D
Sample : M12

Peak Number: 20 at 23.496 min Area: 107116251 Area % 0.78

The 3 best hits from each library.	Ref\#	CAS\#	Qual
D:\Database\W10N14.L			
1 (+)-2-Bornanone	78661	000464-49-3	98
2 Camphor	78669	000076-22-2	98
3 Bicyclo[2.2.1]heptan-2-one, 1,7,...	78649	000076-22-2	98

Library Search Report - ChemStation Integrator

Unknown Spectrum based on Apex

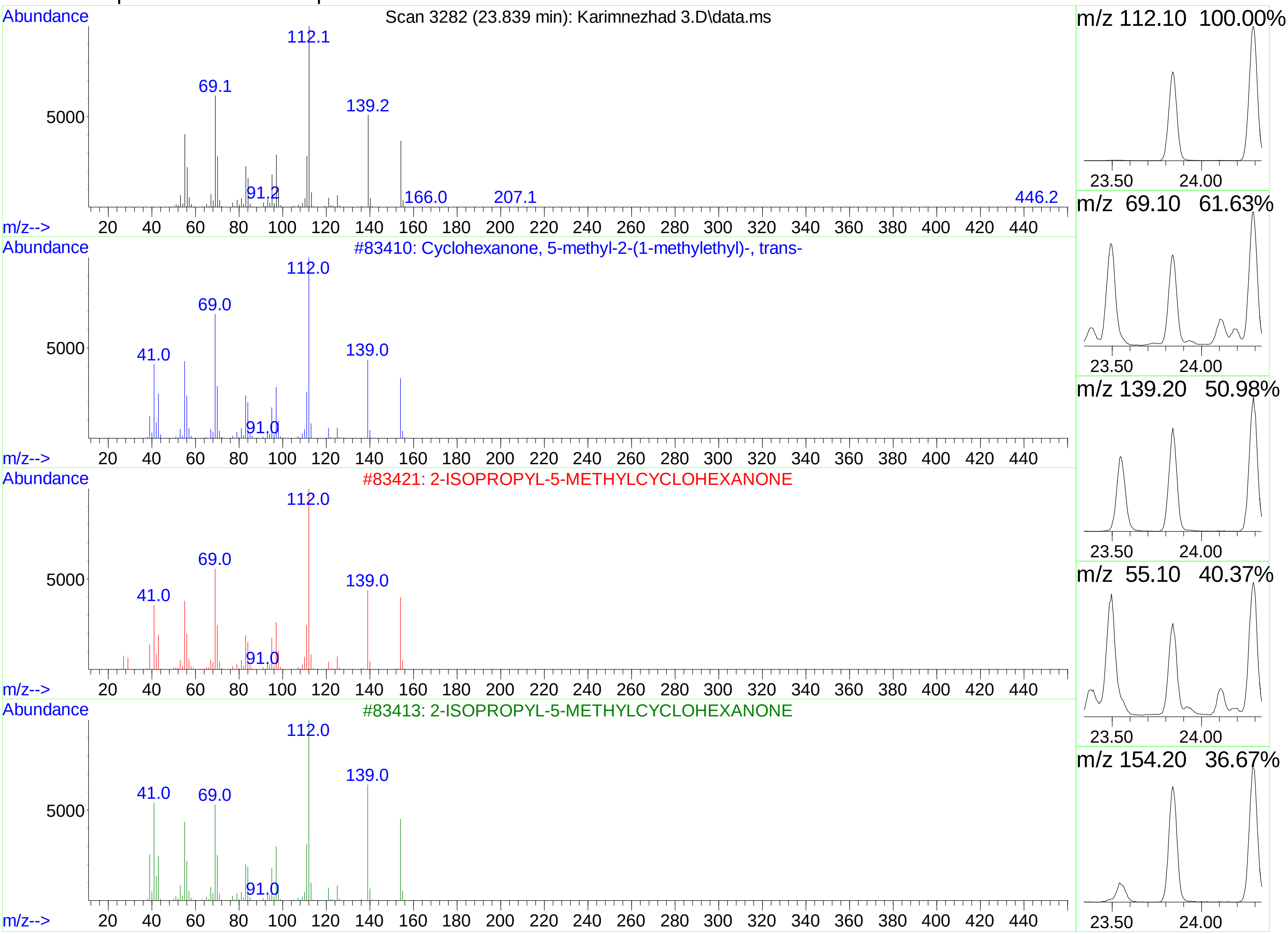

Data File: D:\msdchem\1\data\Karimnezhad 3.D
Sample : M12

Peak Number: 21 at 23.839 min Area: 34504562 Area % 0.25

The 3 best hits from each library.	Ref\#	CAS\#	Qual
D:\Database\W10N14.L			
1 Cyclohexanone, 5-methyl-2-(1-met...	83410	000089-80-5	98
2 2-ISOPROPYL-5-METHYLCYCLOHEXANONE	83421	014073-97-3	98
3 2-ISOPROPYL-5-METHYLCYCLOHEXANONE	83413	000089-80-5	98

Library Search Report - ChemStation Integrator

Unknown Spectrum based on Apex

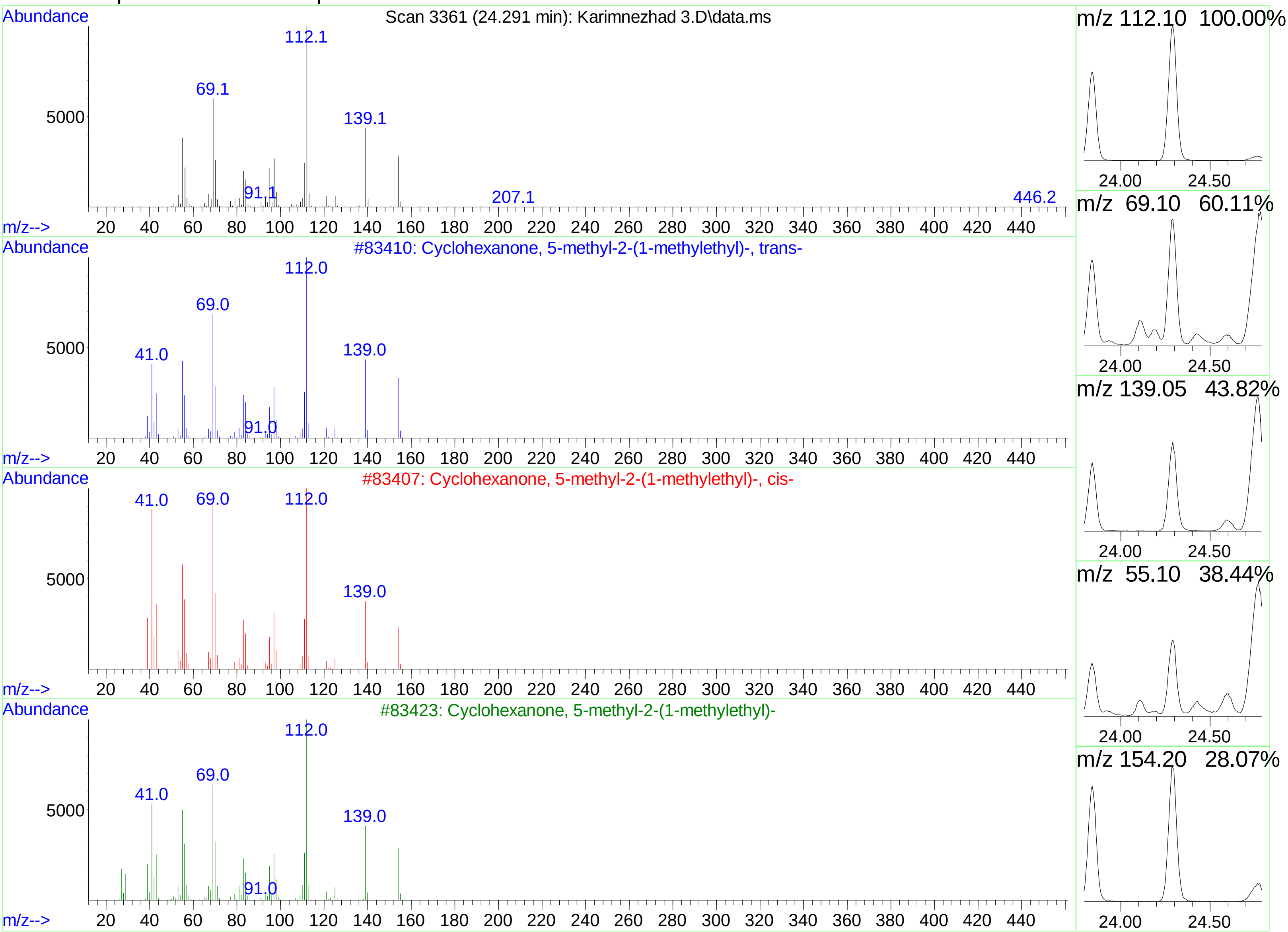

Data File: D:\msdchem\1\data\Karimnezhad 3.D
Sample : M12

Peak Number: 22 at 24.291 min Area: 70529971 Area % 0.51

The 3 best hits from each library.	Ref#	CAS#	Qual
D:\Database\W10N14.L			
1 Cyclohexanone, 5-methyl-2-(1-met...	83410	000089-80-5	98
2 Cyclohexanone, 5-methyl-2-(1-met...	83407	000491-07-6	97
3 Cyclohexanone, 5-methyl-2-(1-met...	83423	010458-14-7	97

Unknown Spectrum based on Apex

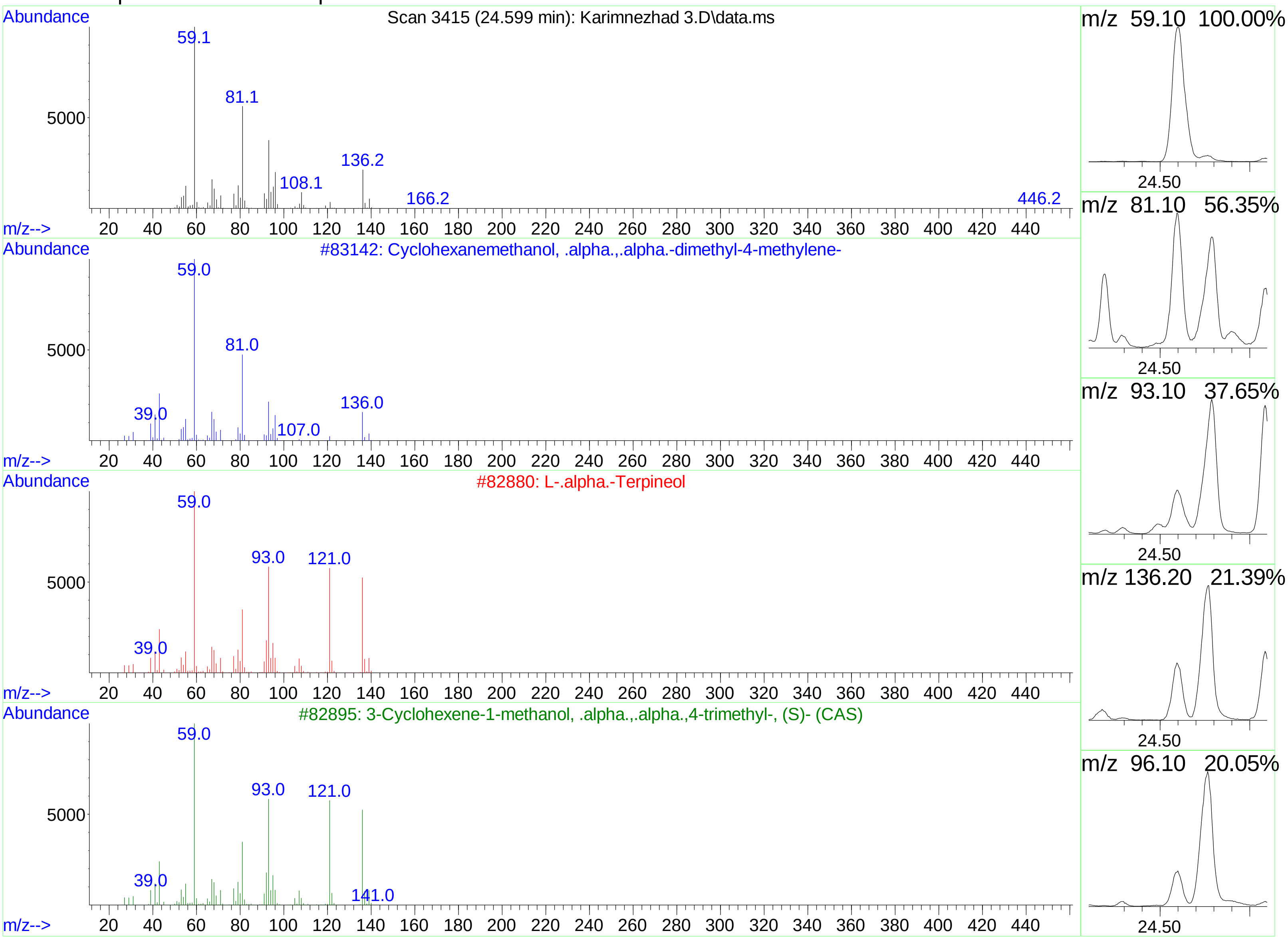

Data File: D:\msdchem\1\data\Karimnezhad 3.D
Sample : M12

Peak Number: 23 at 24.599 min Area: 55060198 Area % 0.40

The 3 best hits from each library.	Ref#	CAS#	Qual
D:\Database\W10N14.L			
1 Cyclohexanemethanol, .alpha.,.al...	83142	007299-42-5	80
2 L-.alpha.-Terpineol	82880	010482-56-1	59
3 3-Cyclohexene-1-methanol, .alpha...	82895	010482-56-1	59

Library Search Report - ChemStation Integrator

Unknown Spectrum based on Apex

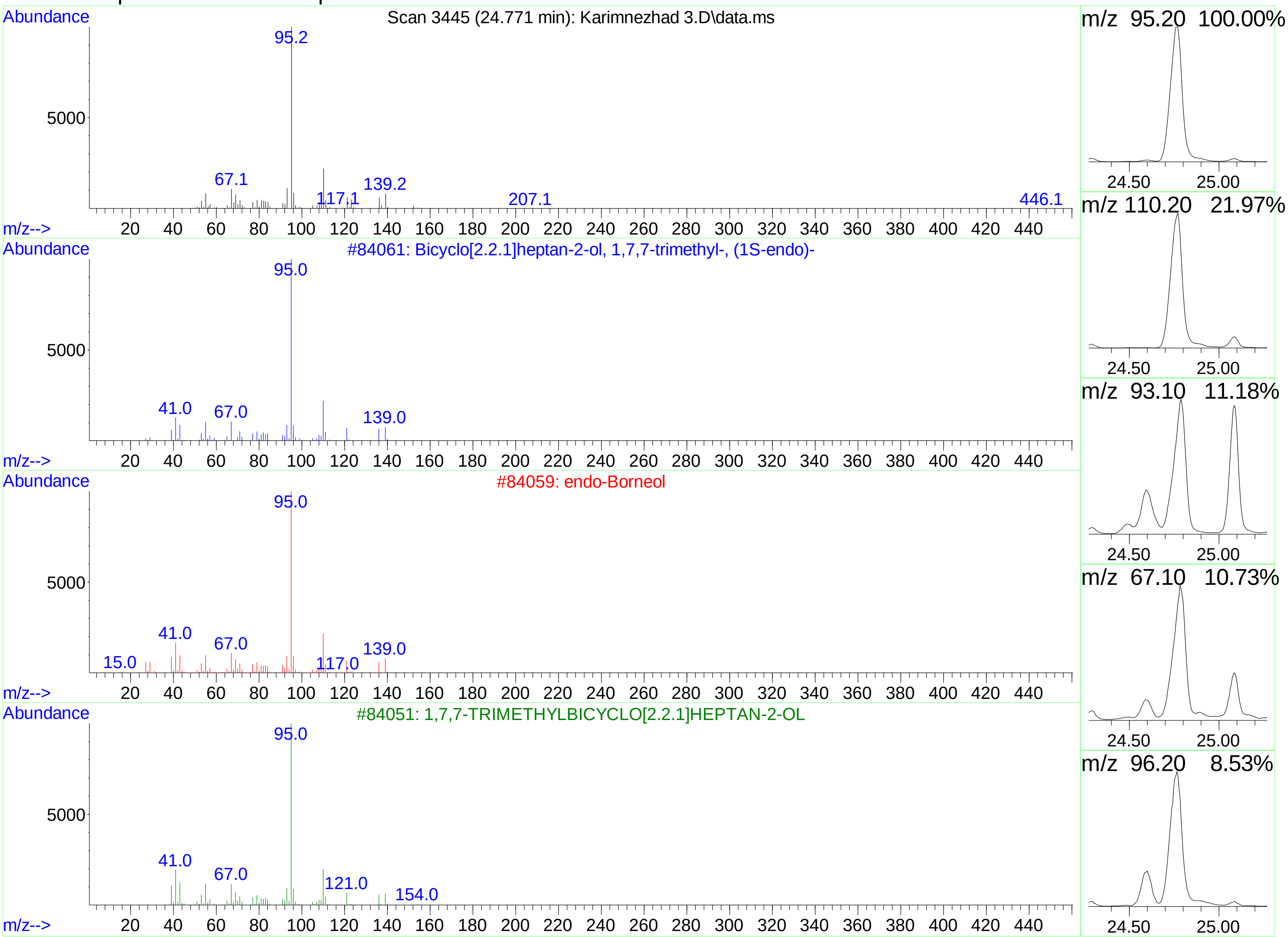

Data File: D:\msdchem\1\data\Karimnezhad 3.D
Sample : M12

Peak Number: 24 at 24.771 min Area: 298875820 Area % 2.18

The 3 best hits from each library. Ref\# CAS\# Qual

D:\Database\W10N14.L

1 Bicyclo[2.2.1]heptan-2-ol, 1,7,7... 84061 000464-45-9 95
2 endo-Borneol 84059 000507-70-0 95
3 1,7,7-TRIMETHYLBICYCLO[2.2.1]HEP... 84051 000464-45-9 94

Unknown Spectrum based on Apex

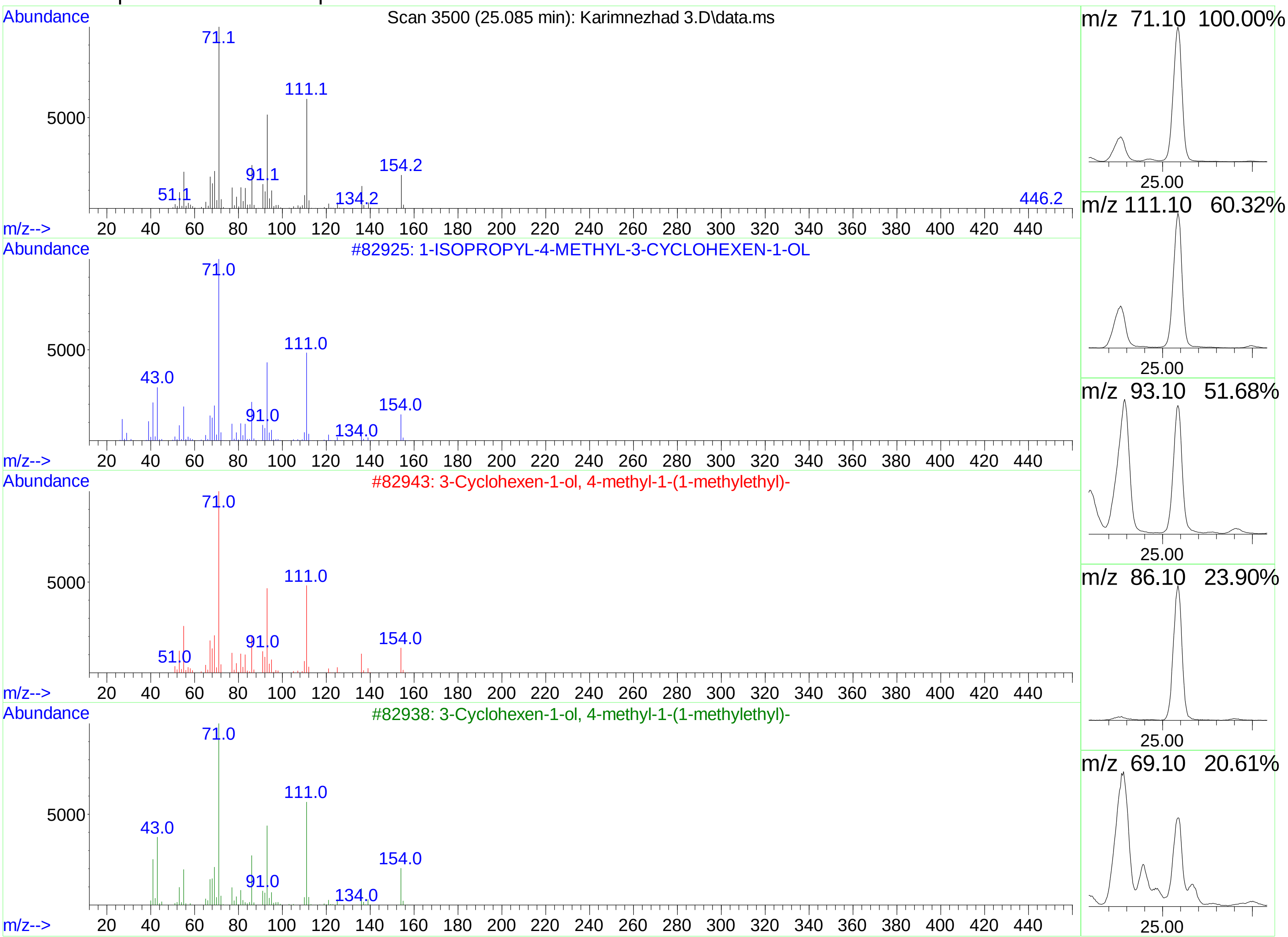

Data File: D:\msdchem\1\data\Karimnezhad 3.D
Sample : M12

Peak Number: 25 at 25.085 min Area: 95904762 Area % 0.70

The 3 best hits from each library. Ref\# CAS\# Qual

D:\Database\W10N14.L

1	1-ISOPROPYL-4-METHYL-3-CYCLOHEXE...	82925	000562-74-3	98
2	3-Cyclohexen-1-ol, 4-methyl-1-(1...	82943	000562-74-3	98
3	3-Cyclohexen-1-ol, 4-methyl-1-(1...	82938	000562-74-3	97

Unknown Spectrum based on Apex

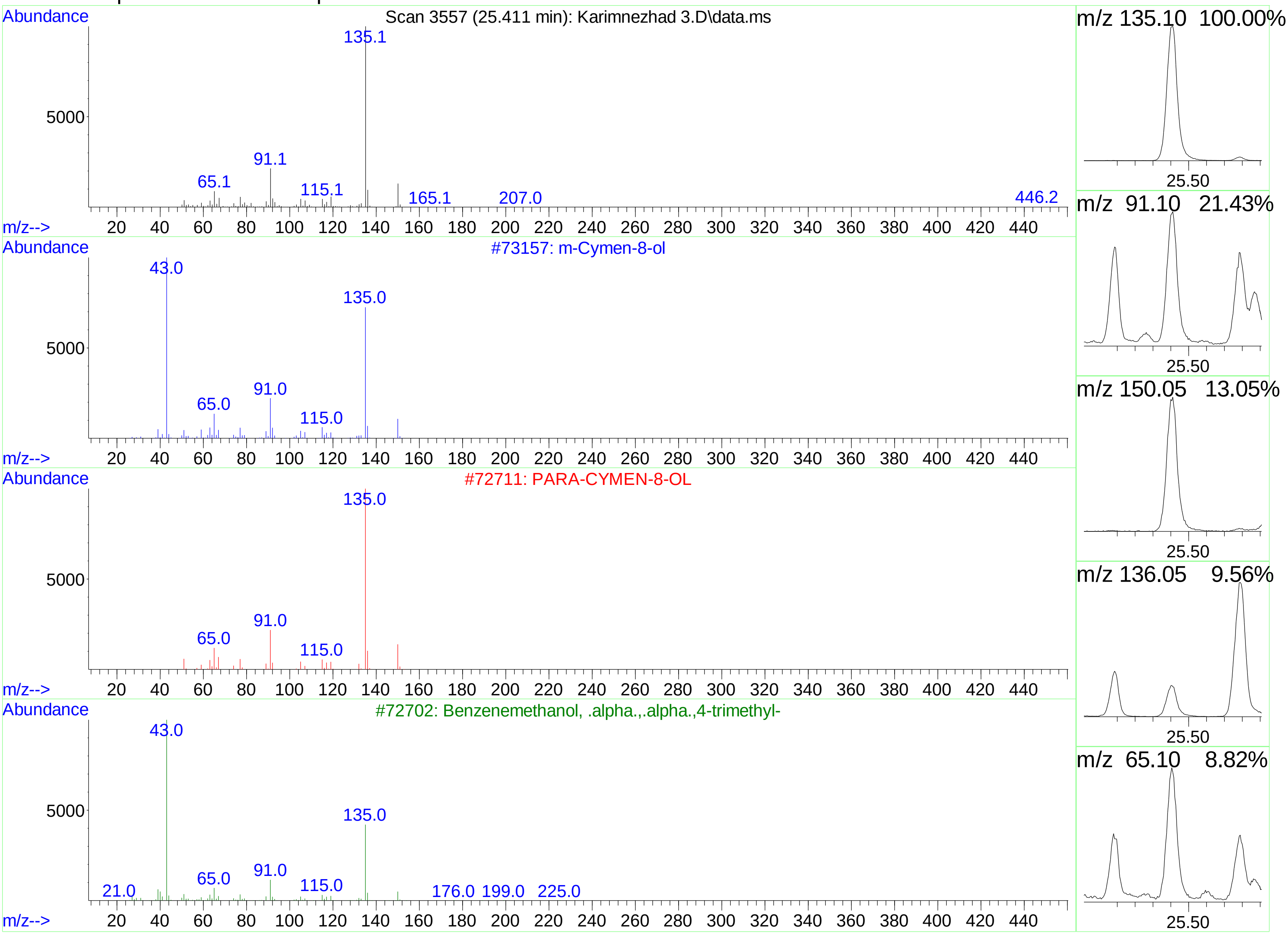

Data File: D:\msdchem\1\data\Karimnezhad 3.D
Sample : M12

Peak Number: 26 at 25.411 min Area: 48626944 Area % 0.35

The 3 best hits from each library.	Ref\#	CAS\#	Qual
D:\Database\W10N14.L			
1 m-Cymen-8-ol	73157	005208-37-7	91
2 PARA-CYMEN-8-OL	72711	001197-01-9	90
3 Benzenemethanol, .alpha.,.alpha....	72702	001197-01-9	87

Unknown Spectrum based on Apex

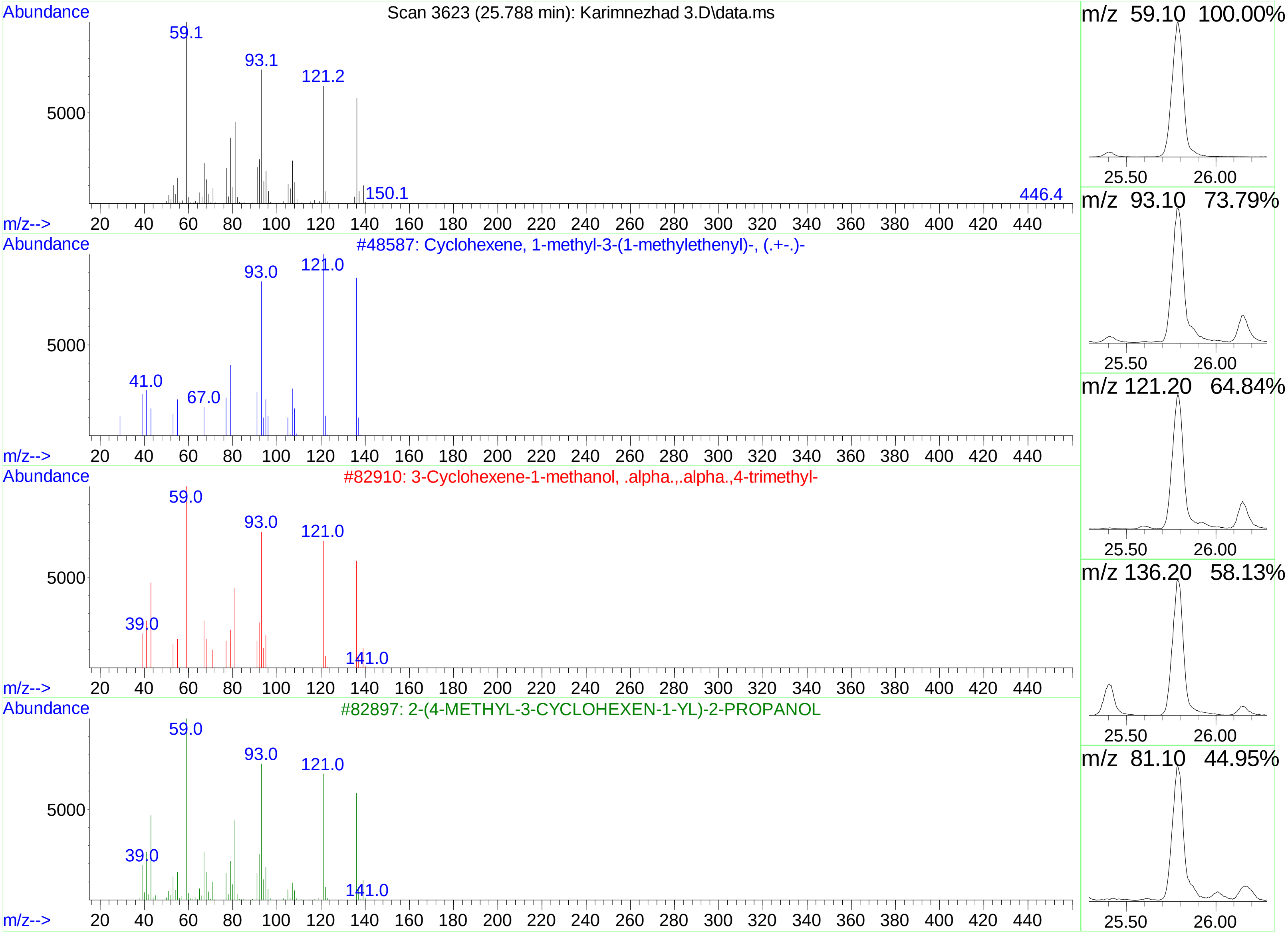

Data File: D:\msdchem\1\data\Karimnezhad 3.D
Sample : M12

Peak Number: 27 at 25.788 min Area: 106771995 Area % 0.78

The 3 best hits from each library. Ref\# CAS\# Qual

D:\Database\W10N14.L

1	Cyclohexene, 1-methyl-3-(1-methy...	48587	000499-03-6	95
2	3-Cyclohexene-1-methanol, .alpha...	82910	000098-55-5	87
3	2-(4-METHYL-3-CYCLOHEXEN-1-YL)-2...	82897	000098-55-5	87

Unknown Spectrum based on Apex

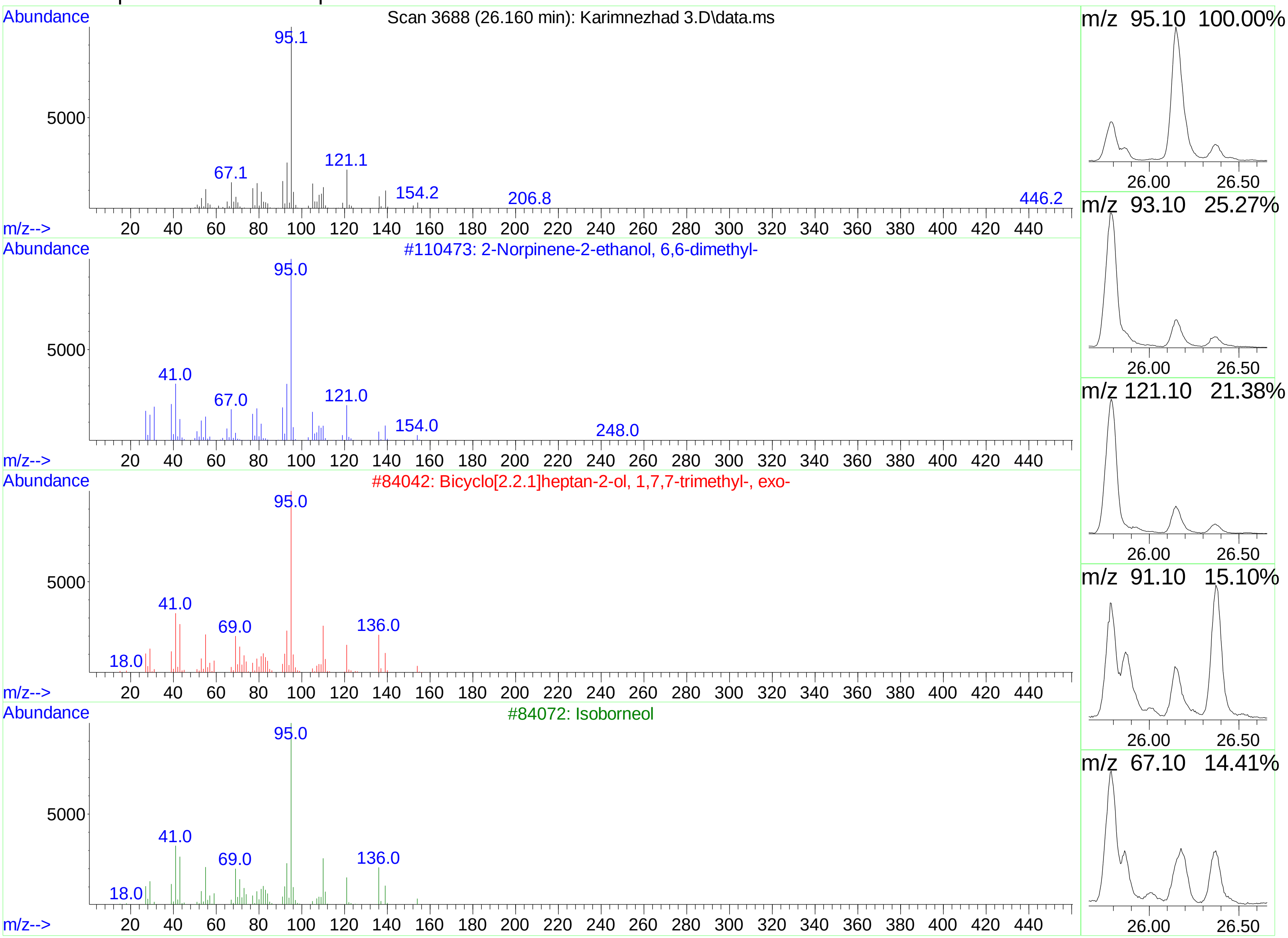

Data File: D:\msdchem\1\data\Karimnezhad 3.D
Sample : M12

Peak Number: 28 at 26.160 min Area: 30449515 Area % 0.22

The 3 best hits from each library. Ref\# CAS\# Qual

D:\Database\W10N14.L

1	2-Norpinene-2-ethanol, 6,6-dimet...	110473	000128-50-7	91
2	Bicyclo[2.2.1]heptan-2-ol, 1,7,7...	84042	000124-76-5	86
3	Isoborneol	84072	000124-76-5	86

Unknown Spectrum based on Apex

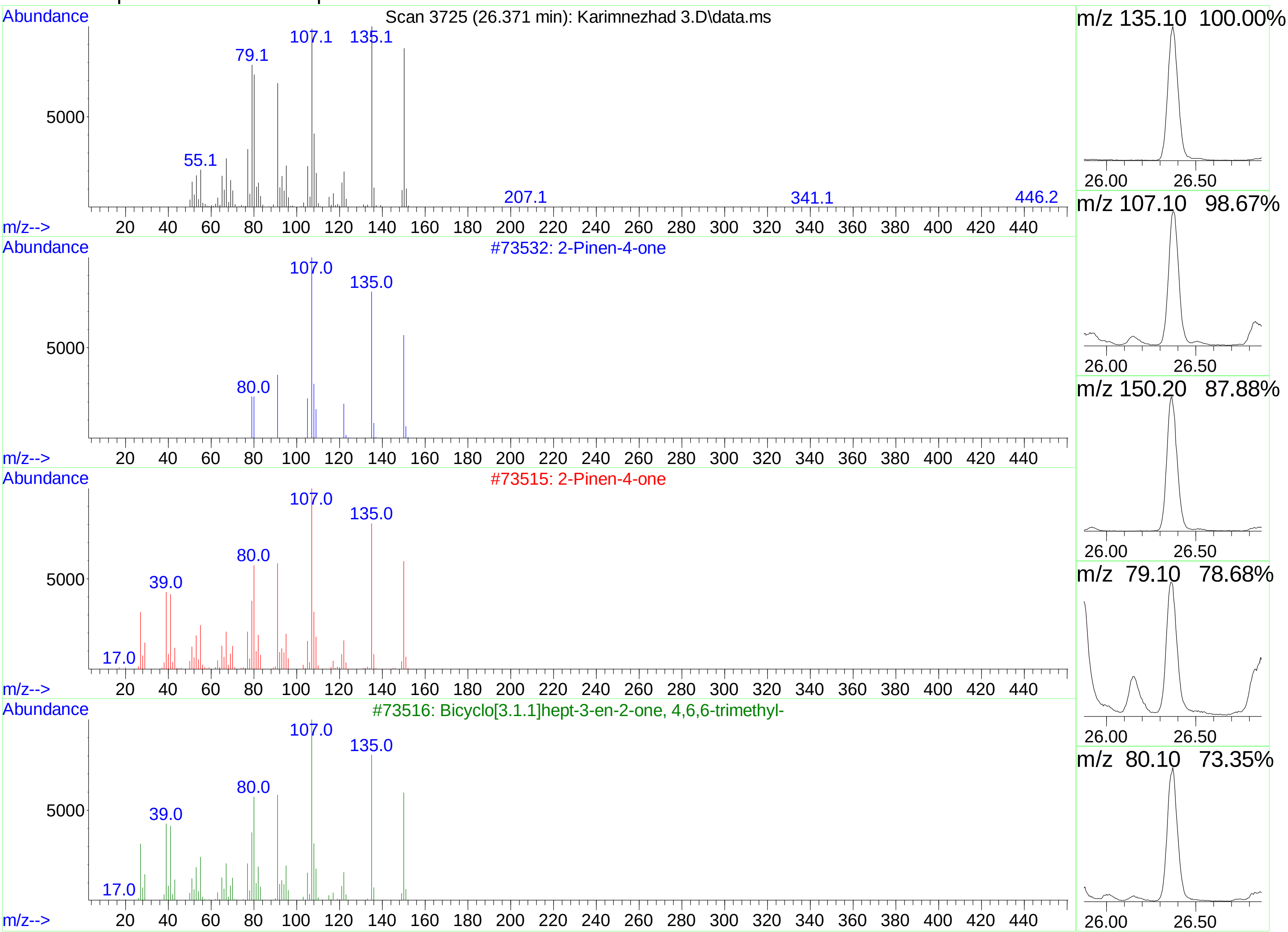

Data File: D:\msdchem\1\data\Karimnezhad 3.D
Sample : M12

Peak Number: 29 at 26.371 min Area: 44328111 Area % 0.32

The 3 best hits from each library. Ref\# CAS\# Qual

D:\Database\W10N14.L

1	2-Pinen-4-one	73532	000080-57-9	96
2	2-Pinen-4-one	73515	000080-57-9	96
3	Bicyclo[3.1.1]hept-3-en-2-one, 4...	73516	000080-57-9	96

Unknown Spectrum based on Apex

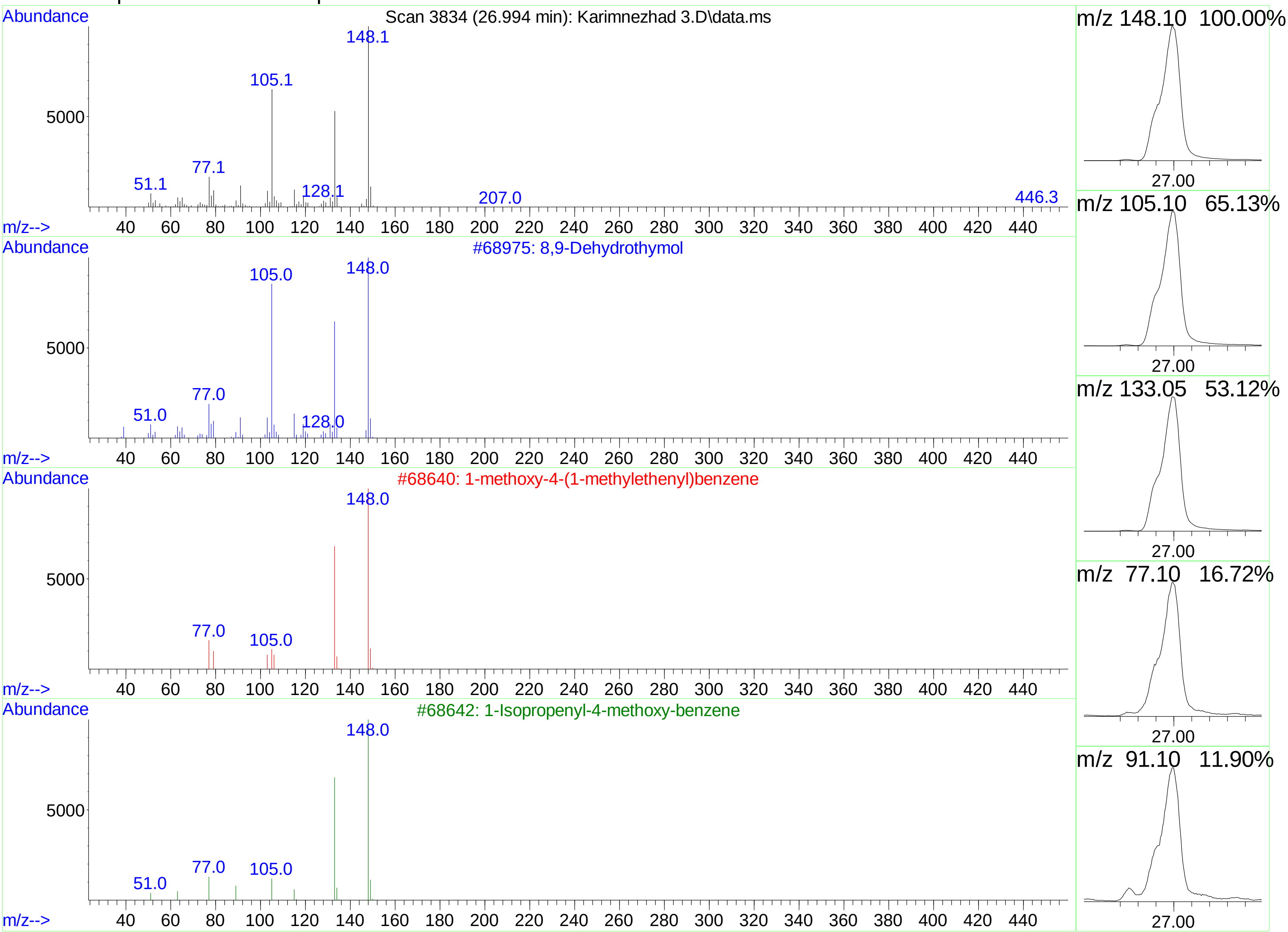

Data File: D:\msdchem\1\data\Karimnezhad 3.D
Sample : M12

Peak Number: 30 at 26.994 min Area: 490930702 Area % 3.57

The 3 best hits from each library.	Ref#	CAS#	Qual
D:\Database\W10N14.L			
1 8,9-Dehydrothymol	68975	018612-99-2	95
2 1-methoxy-4-(1-methylethenyl)ben...	68640	2000068-64-0	81
3 1-Isopropenyl-4-methoxy-benzene	68642	2000068-64-2	80

Unknown Spectrum based on Apex

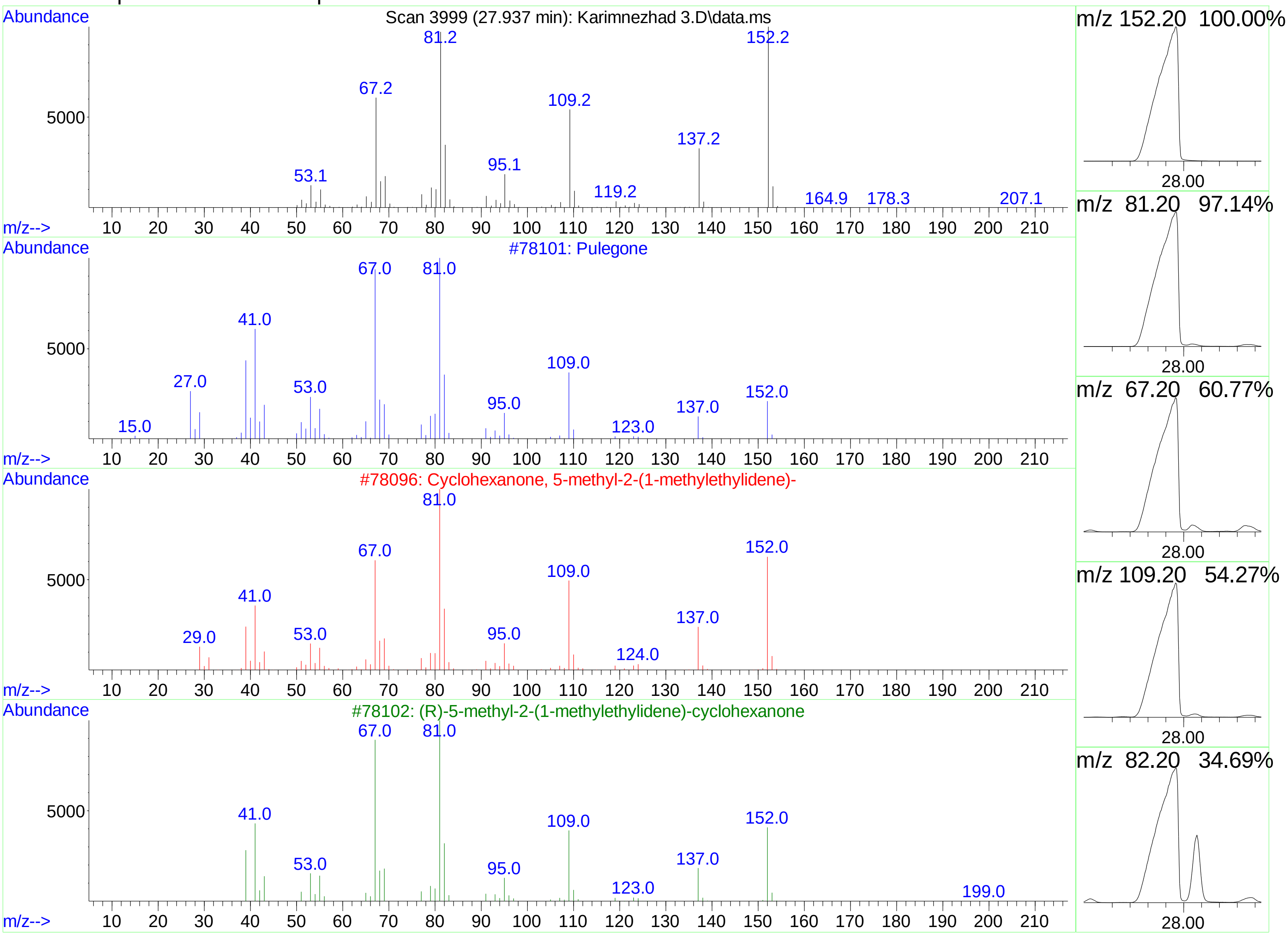

Data File: D:\msdchem\1\data\Karimnezhad 3.D
Sample : M12

Peak Number: 31 at 27.937 min Area: 1405728815 Area % 10.23

The 3 best hits from each library.	Ref#	CAS#	Qual
D:\Database\W10N14.L			
1 Pulegone	78101	000089-82-7	97
2 Cyclohexanone, 5-methyl-2-(1-met...	78096	015932-80-6	96
3 (R)-5-methyl-2-(1-methylethylide...	78102	000089-82-7	96

Unknown Spectrum based on Apex

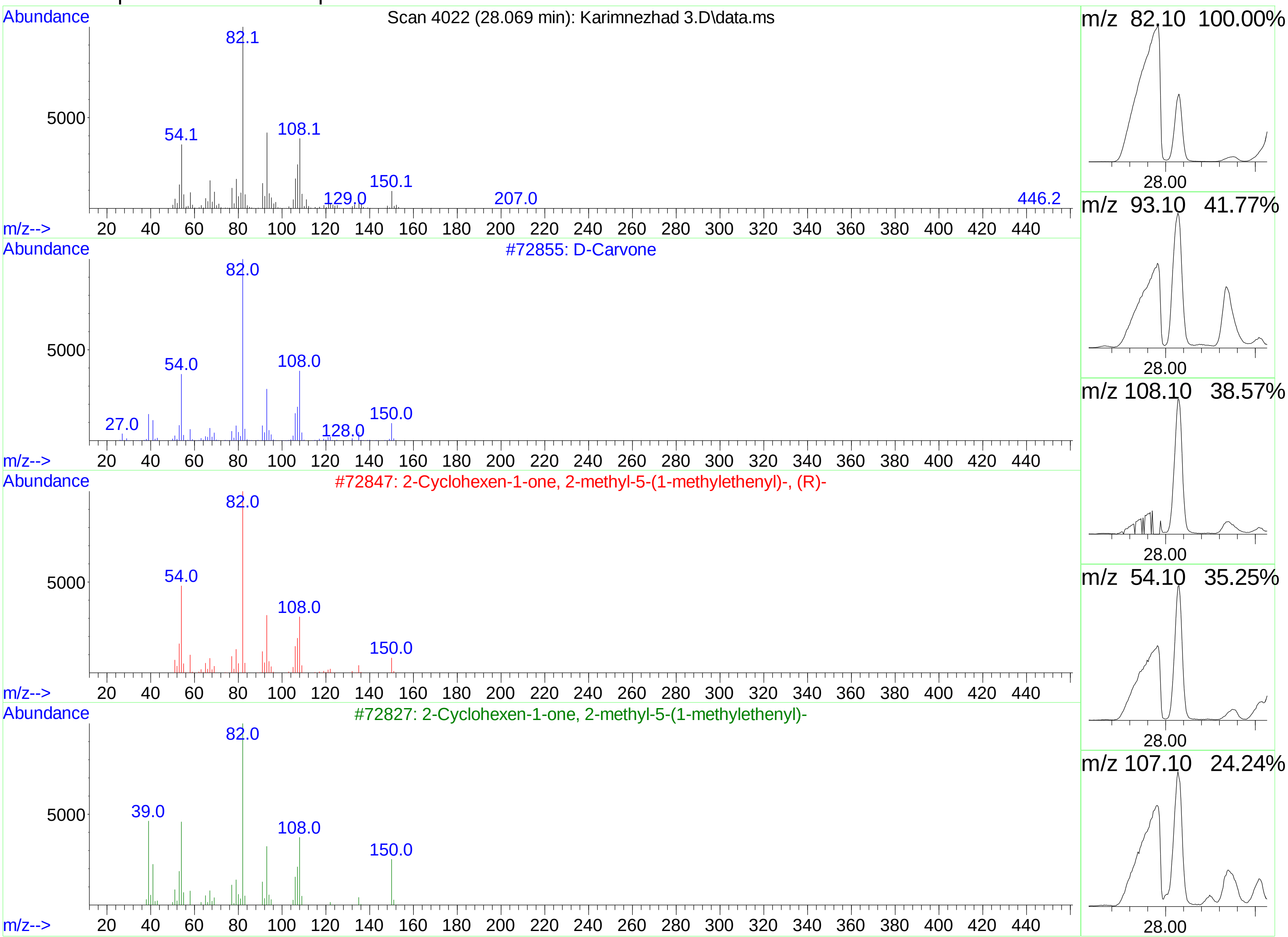

Data File: D:\msdchem\1\data\Karimnezhad 3.D
Sample : M12

Peak Number: 32 at 28.069 min Area: 89800601 Area % 0.65

The 3 best hits from each library. Ref\# CAS\# Qual

D:\Database\W10N14.L

		Ref#	CAS#	Qual
1	D-Carvone	72855	002244-16-8	96
2	2-Cyclohexen-1-one, 2-methyl-5-(...	72847	006485-40-1	95
3	2-Cyclohexen-1-one, 2-methyl-5-(...	72827	000099-49-0	95

Unknown Spectrum based on Apex

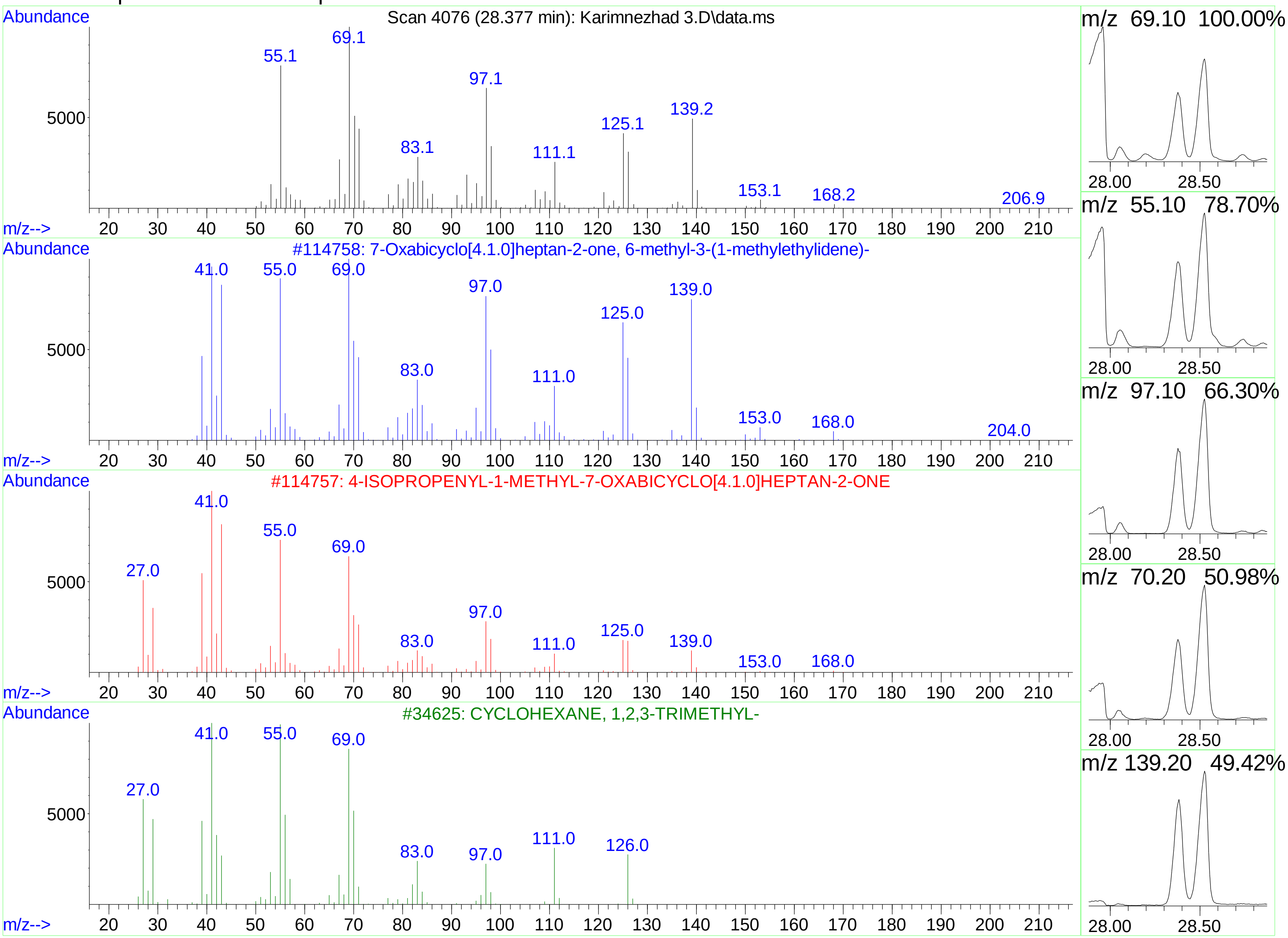

Data File: D:\msdchem\1\data\Karimnezhad 3.D
Sample : M12

Peak Number: 33 at 28.377 min Area: 110059313 Area % 0.80

The 3 best hits from each library. Ref\# CAS\# Qual

D:\Database\W10N14.L

1	7-Oxabicyclo[4.1.0]heptan-2-one,...	114758	035178-55-3	98
2	4-ISOPROPENYL-1-METHYL-7-OXABICY...	114757	035178-55-3	52
3	CYCLOHEXANE, 1,2,3-TRIMETHYL-	34625	001678-97-3	45

Unknown Spectrum based on Apex

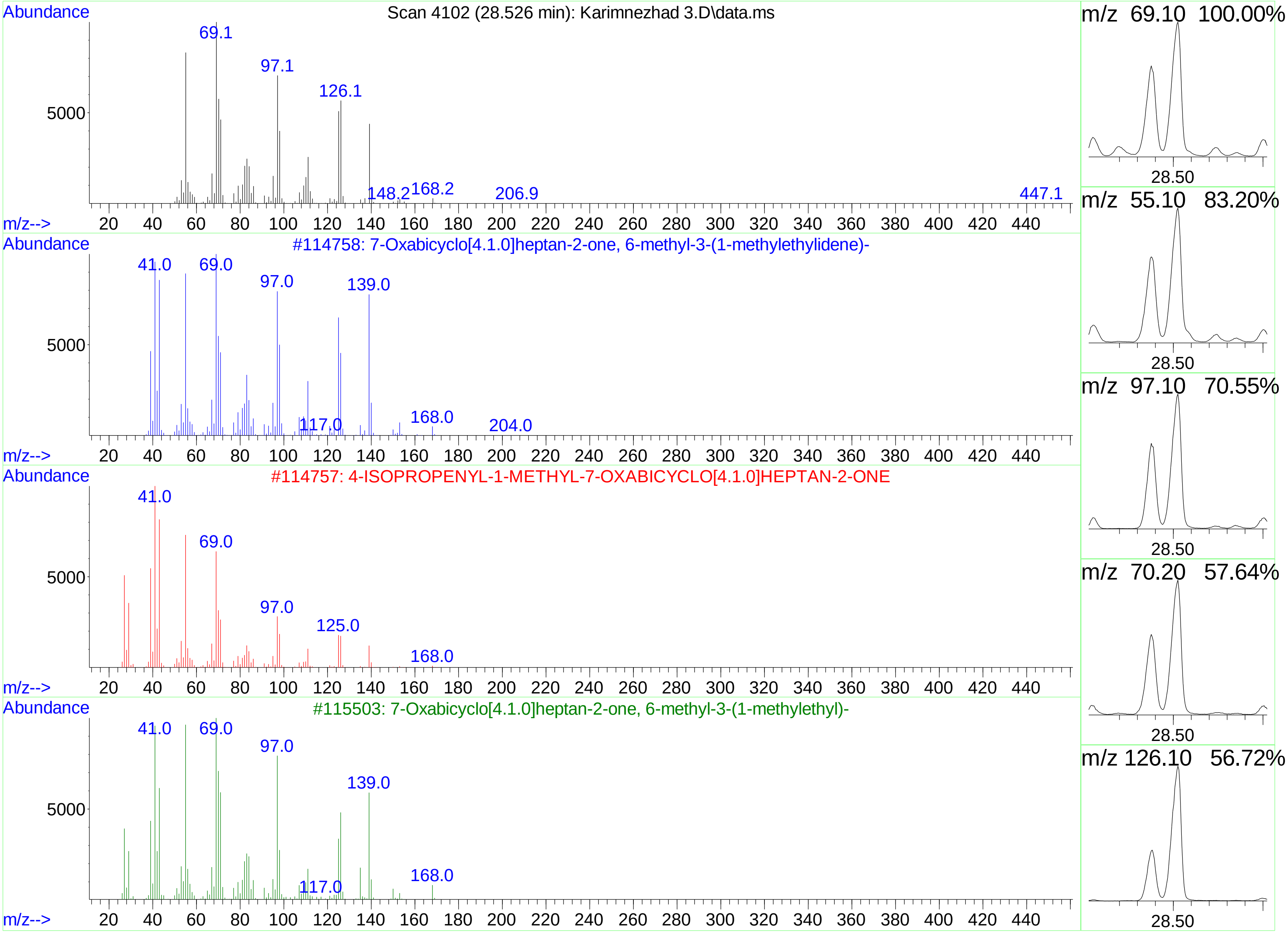

Data File: D:\msdchem\1\data\Karimnezhad 3.D
Sample : M12

Peak Number: 34 at 28.526 min Area: 164443863 Area % 1.20

The 3 best hits from each library.	Ref#	CAS#	Qual
D:\Database\W10N14.L			
1 7-Oxabicyclo[4.1.0]heptan-2-one,...	114758	035178-55-3	94
2 4-ISOPROPENYL-1-METHYL-7-OXABICY...	114757	035178-55-3	93
3 7-Oxabicyclo[4.1.0]heptan-2-one,...	115503	005286-38-4	87

Unknown Spectrum based on Apex

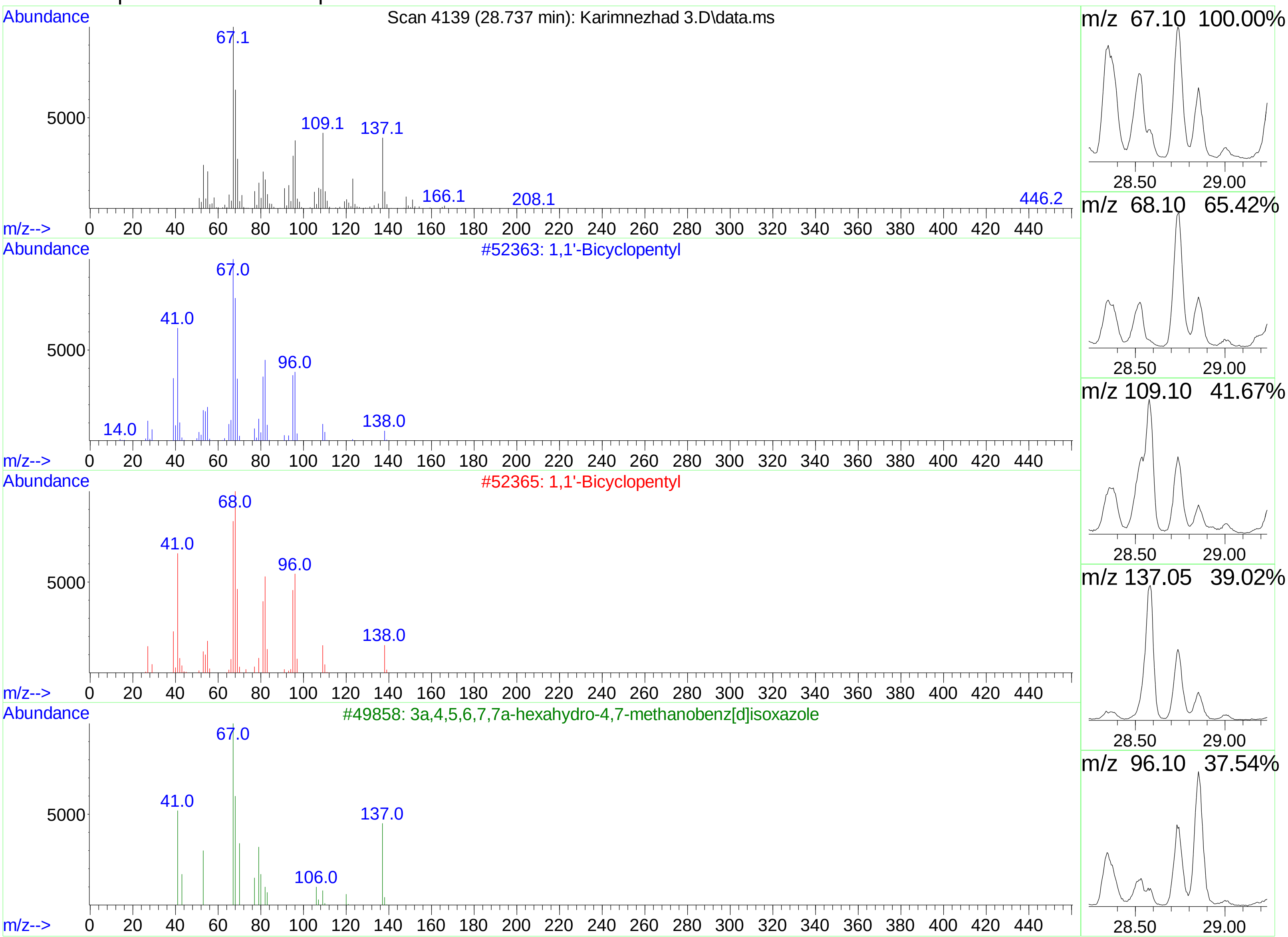

Data File: D:\msdchem\1\data\Karimnezhad 3.D
Sample : M12

Peak Number: 35 at 28.737 min Area: 23283606 Area % 0.17

The 3 best hits from each library.	Ref\#	CAS\#	Qual
D:\Database\W10N14.L			
1 1,1'-Bicyclopentyl	52363	001636-39-1	52
2 1,1'-Bicyclopentyl	52365	001636-39-1	50
3 3a,4,5,6,7,7a-hexahydro-4,7-meth...	49858	015166-80-0	49

Unknown Spectrum based on Apex

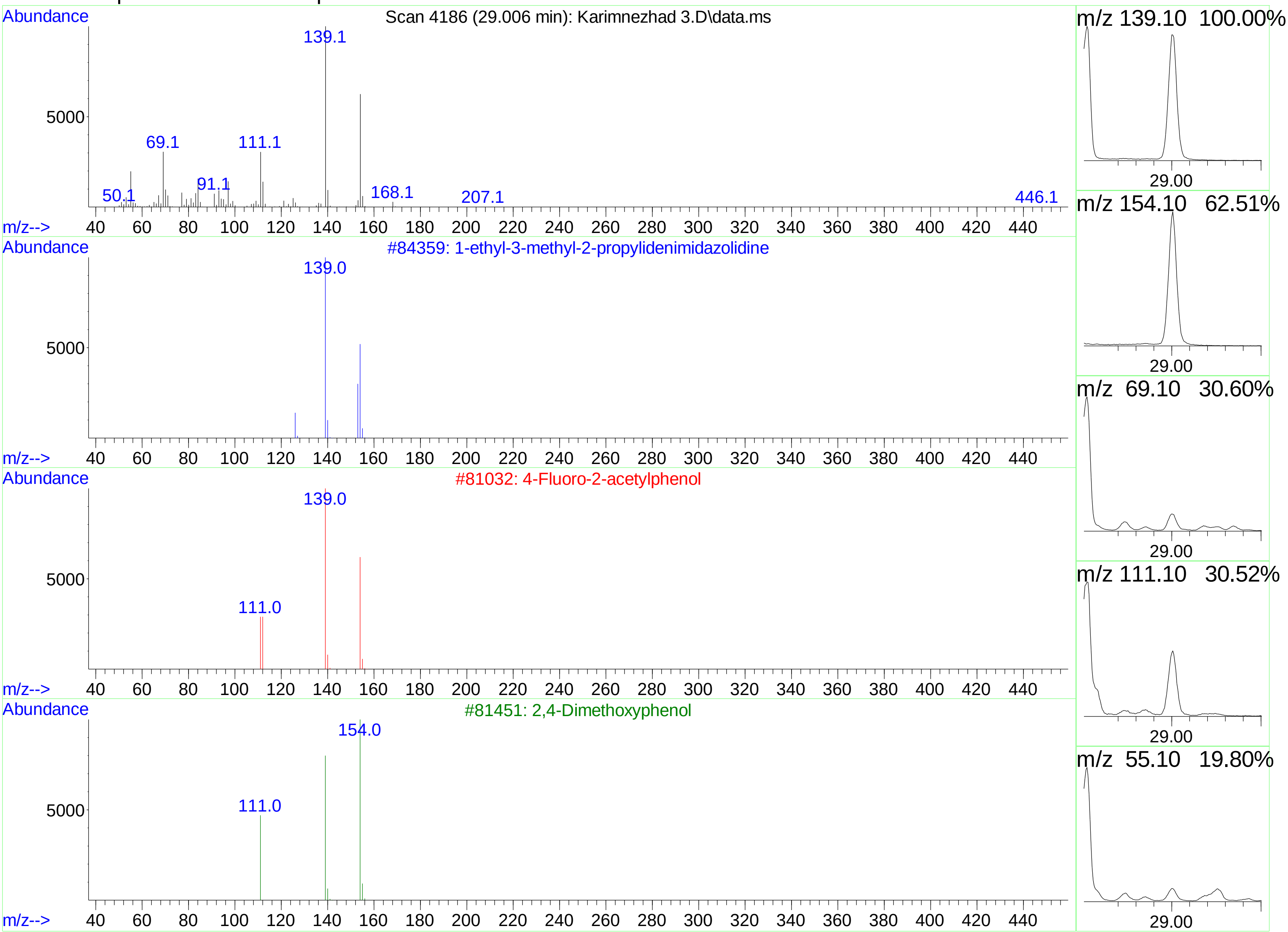

Data File: D:\msdchem\1\data\Karimnezhad 3.D
Sample : M12

Peak Number: 36 at 29.006 min Area: 24541303 Area % 0.18

The 3 best hits from each library.	Ref#	CAS#	Qual
D:\Database\W10N14.L			
1 1-ethyl-3-methyl-2-propylidenimi...	84359	109153-29-9	83
2 4-Fluoro-2-acetylphenol	81032	000394-32-1	80
3 2,4-Dimethoxyphenol	81451	013330-65-9	80

Unknown Spectrum based on Apex

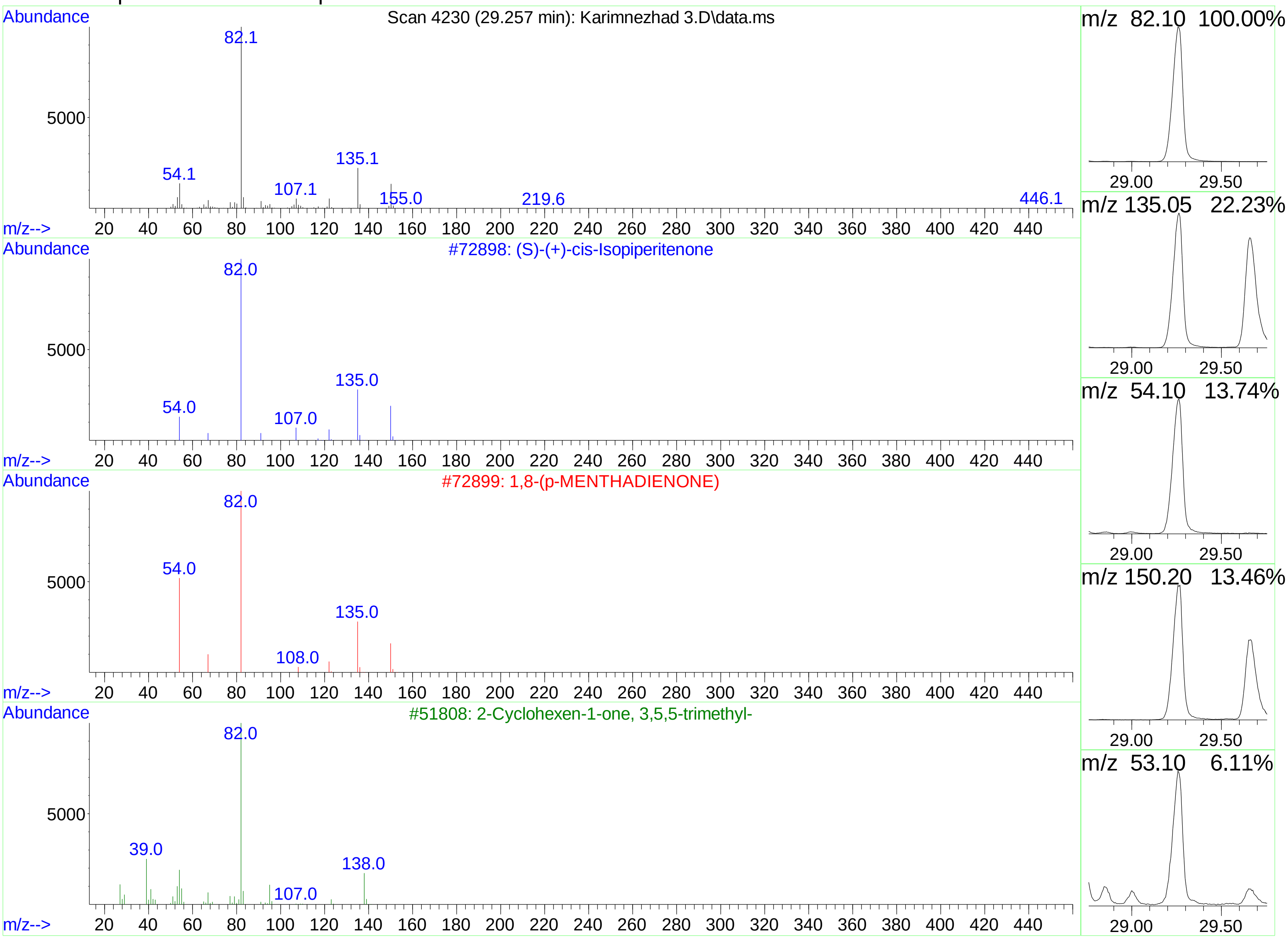

Data File: D:\msdchem\1\data\Karimnezhad 3.D
Sample : M12

Peak Number: 37 at 29.257 min Area: 135688340 Area % 0.99

The 3 best hits from each library.	Ref#	CAS#	Qual
D:\Database\W10N14.L			
1 (S)-(+)-cis-Isopiperitenone	72898	2000072-89-8	91
2 1,8-(p-MENTHADIENONE)	72899	2000072-89-9	72
3 2-Cyclohexen-1-one, 3,5,5-trimet...	51808	000078-59-1	50

Unknown Spectrum based on Apex

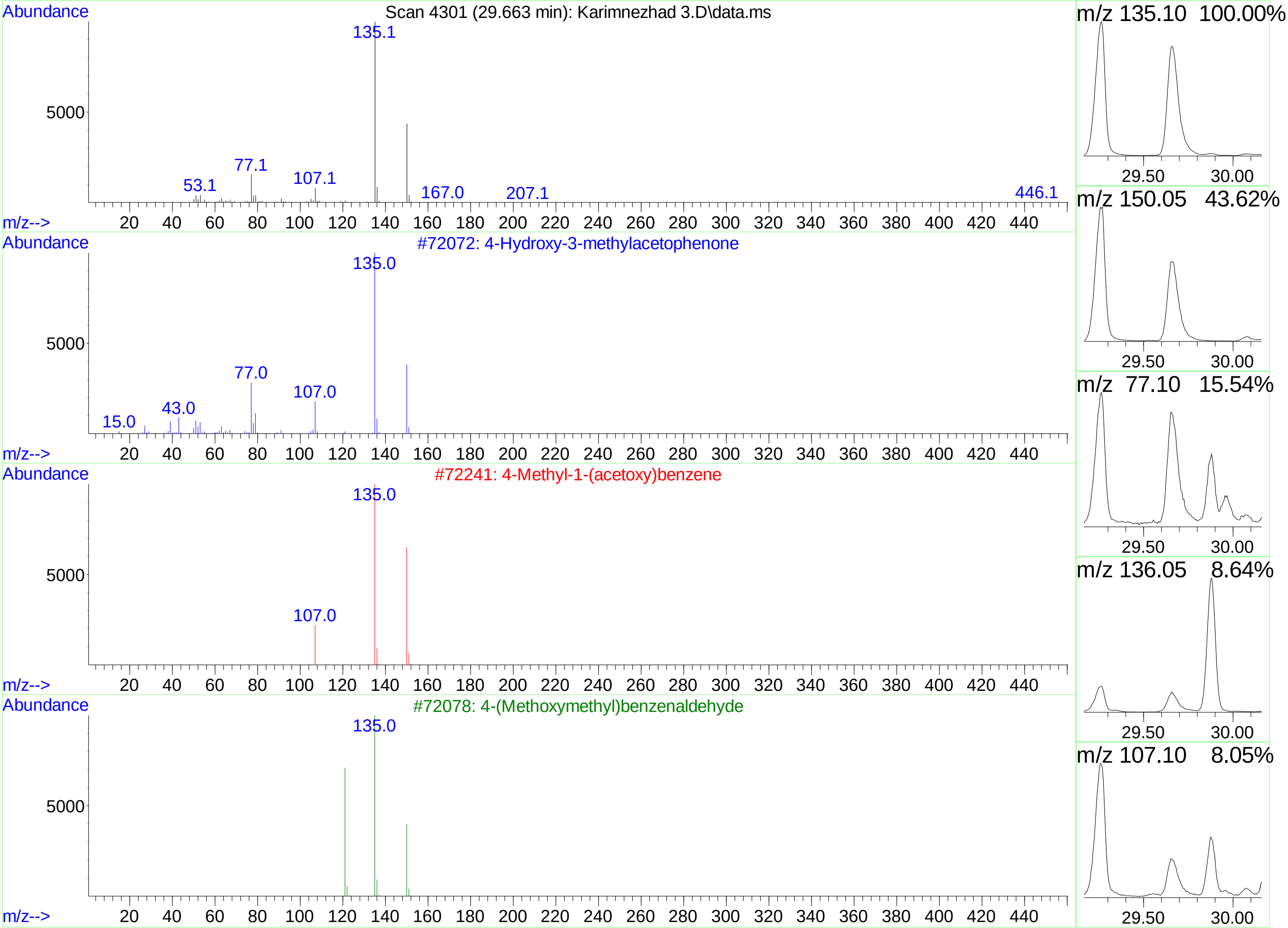

Data File: D:\msdchem\1\data\Karimnezhad 3.D
Sample : M12

Peak Number: 38 at 29.663 min Area: 24606767 Area % 0.18

The 3 best hits from each library.	Ref\#	CAS\#	Qual
D:\Database\W10N14.L			
1 4-Hydroxy-3-methylacetophenone	72072	000876-02-8	91
2 4-Methyl-1-(acetoxy)benzene	72241	000140-39-6	90
3 4-(Methoxymethyl)benzenaldehyde	72078	2000072-07-8	90

Library Search Report - ChemStation Integrator

Unknown Spectrum based on Apex

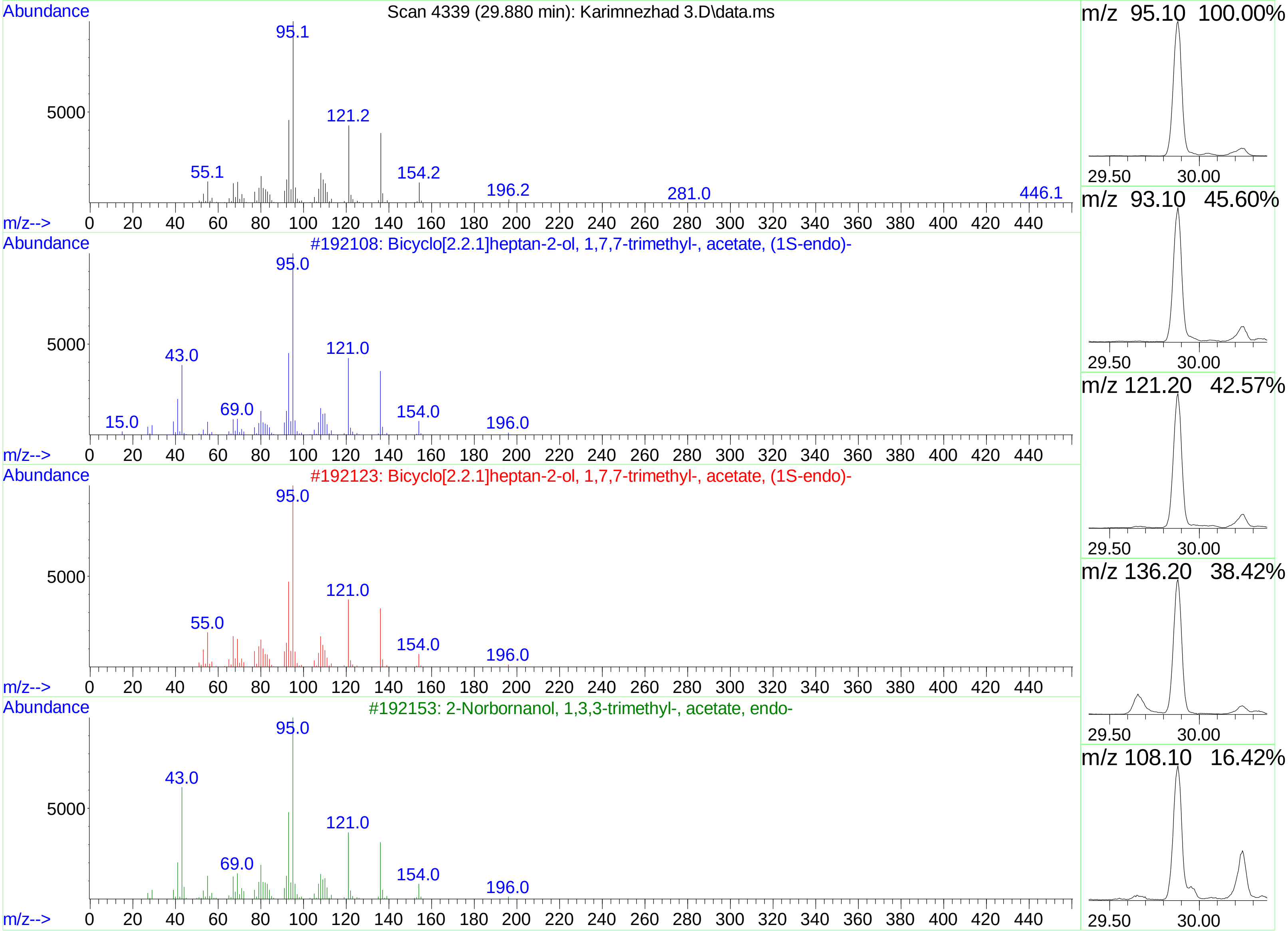

Data File: D:\msdchem\1\data\Karimnezhad 3.D
Sample : M12

Peak Number: 39 at 29.880 min Area: 92332926 Area % 0.67

The 3 best hits from each library. Ref\# CAS\# Qual

D:\Database\W10N14.L

1	Bicyclo[2.2.1]heptan-2-ol, 1,7,7...	192108	005655-61-8	99
2	Bicyclo[2.2.1]heptan-2-ol, 1,7,7...	192123	005655-61-8	99
3	2-Norbornanol, 1,3,3-trimethyl-,...	192153	004057-31-2	98

Unknown Spectrum based on Apex

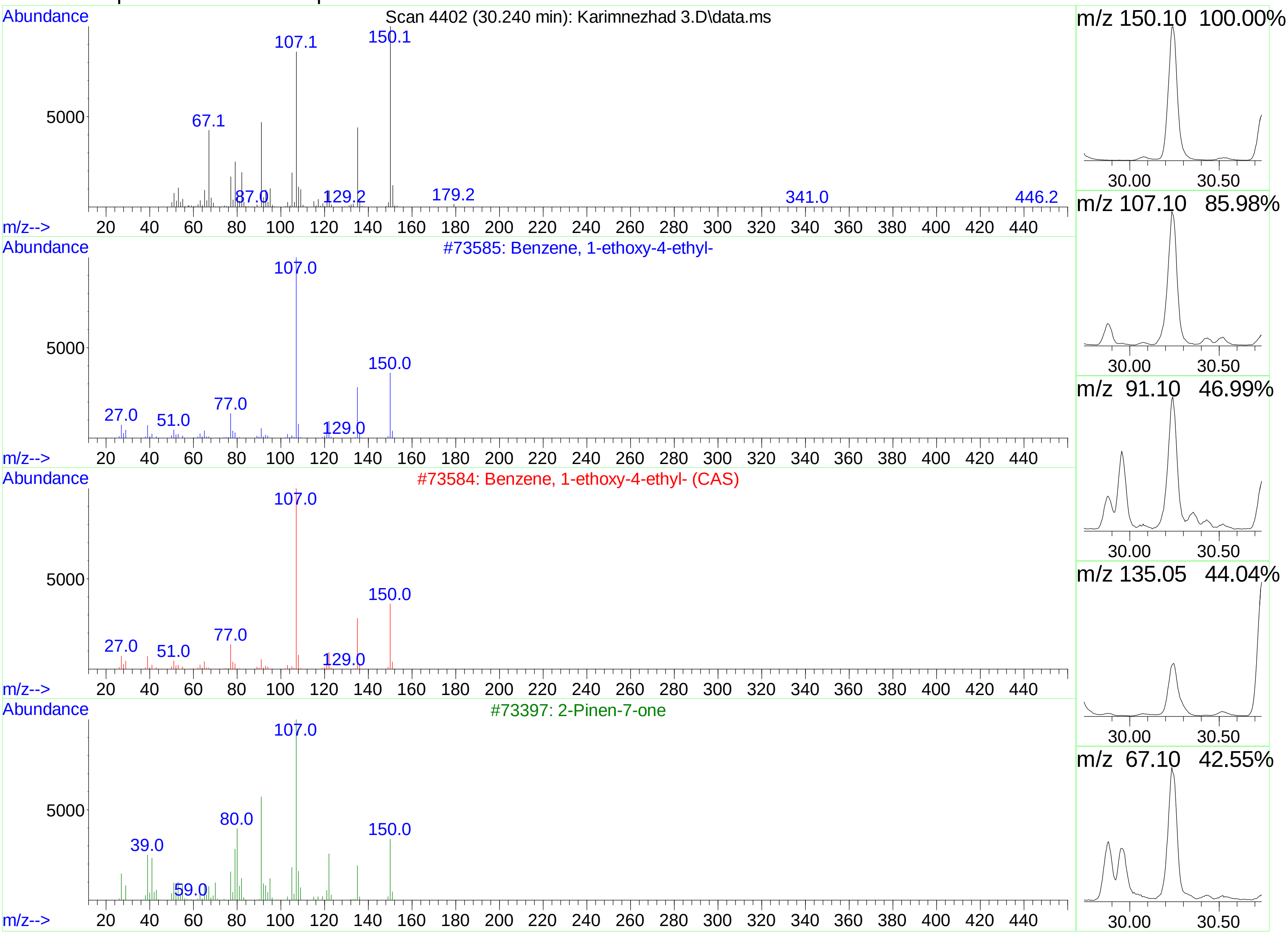

Data File: D:\msdchem\1\data\Karimnezhad 3.D
Sample : M12

Peak Number: 40 at 30.240 min Area: 62358264 Area % 0.45

The 3 best hits from each library.	Ref\#	CAS\#	Qual
D:\Database\W10N14.L			
1 Benzene, 1-ethoxy-4-ethyl-	73585	001585-06-4	89
2 Benzene, 1-ethoxy-4-ethyl- (CAS)	73584	001585-06-4	89
3 2-Pinen-7-one	73397	000473-06-3	81

Library Search Report - ChemStation Integrator

Unknown Spectrum based on Apex

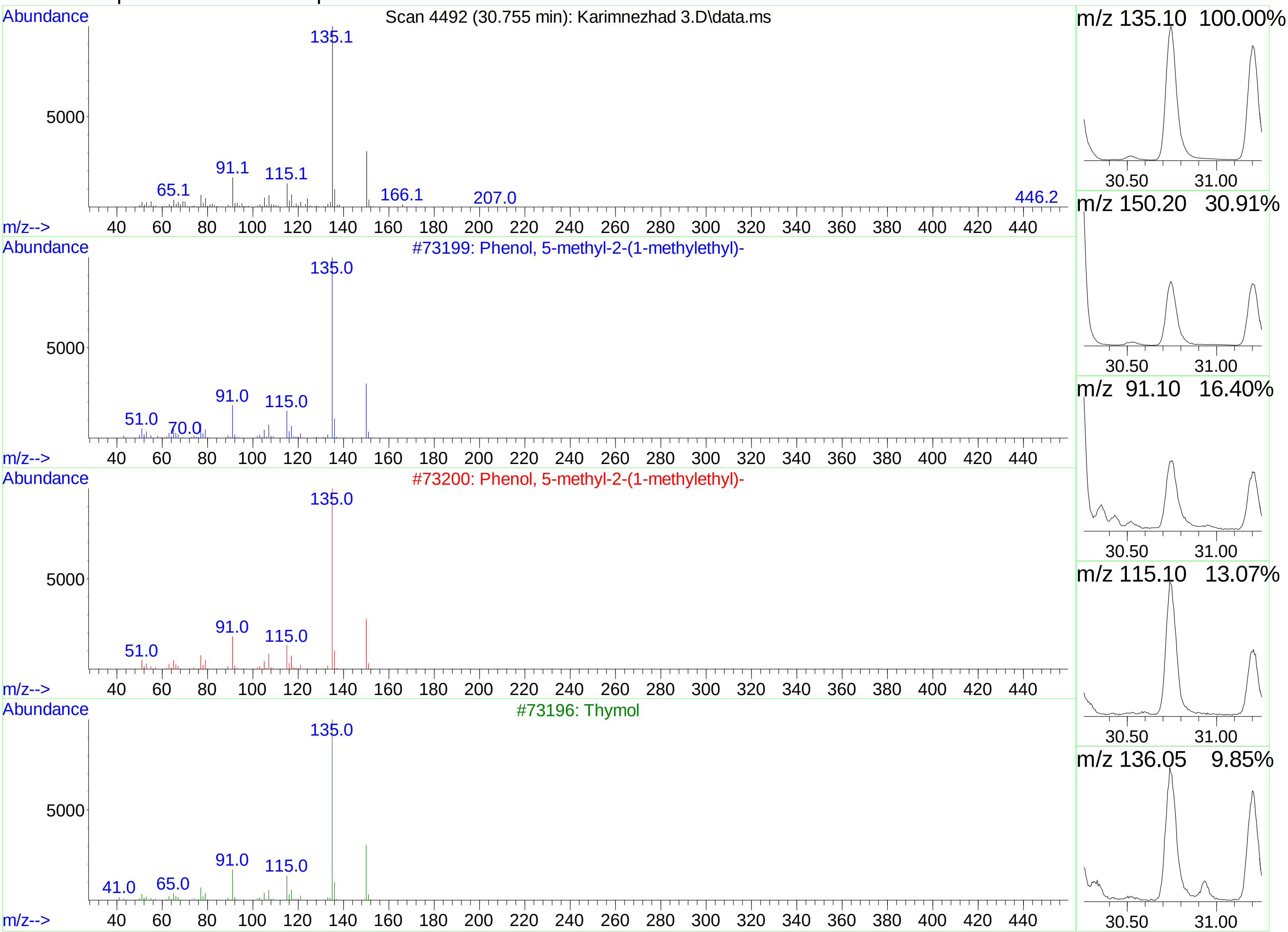

Data File: D:\msdchem\1\data\Karimnezhad 3.D
Sample : M12

Peak Number: 41 at 30.755 min Area: 49101459 Area % 0.36

The 3 best hits from each library.	Ref#	CAS#	Qual
D:\Database\W10N14.L			
1 Phenol, 5-methyl-2-(1-methylethyl)-	73199	000089-83-8	95
2 Phenol, 5-methyl-2-(1-methylethyl)-	73200	000089-83-8	95
3 Thymol	73196	000089-83-8	94

Unknown Spectrum based on Apex

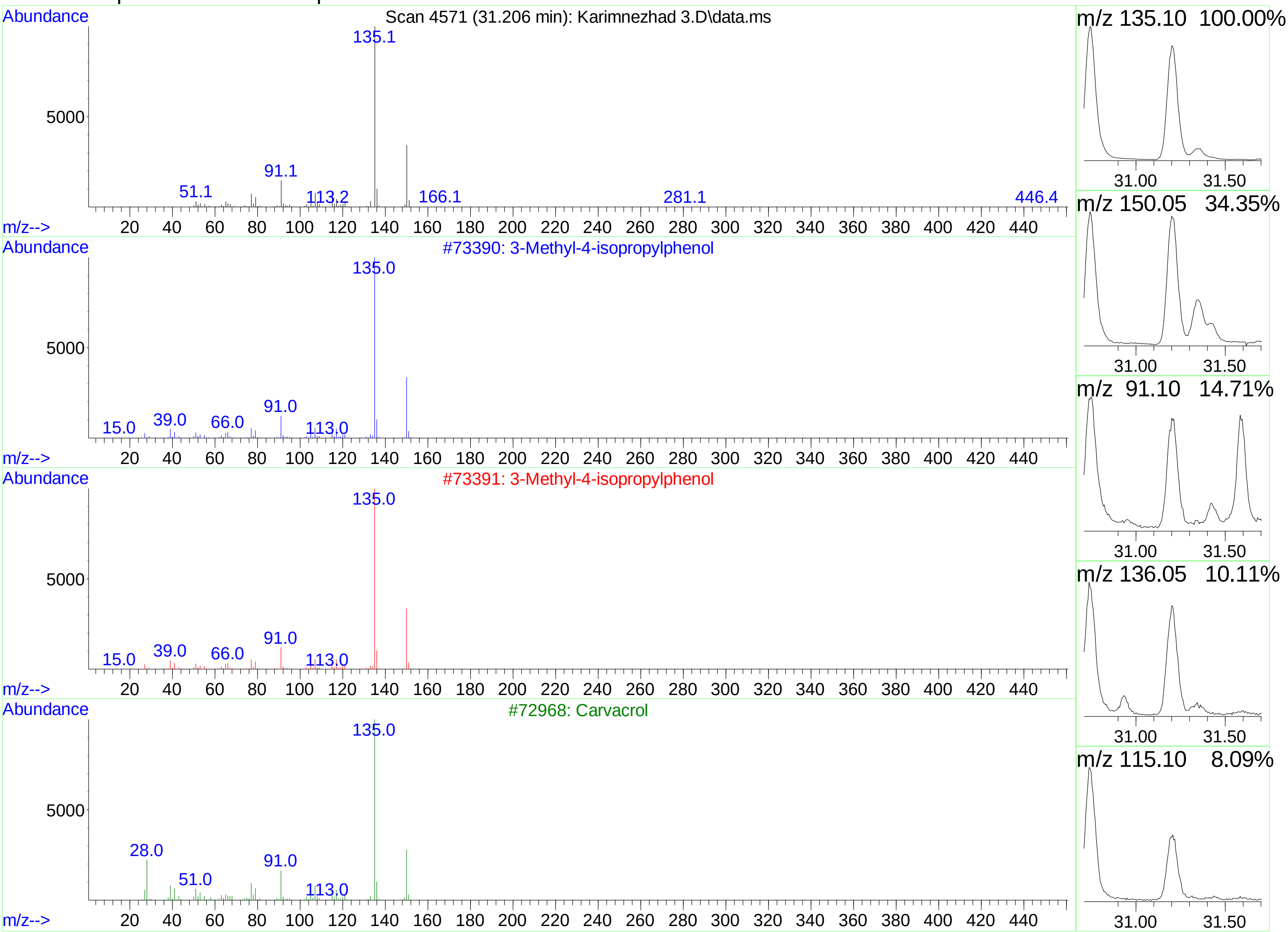

Data File: D:\msdchem\1\data\Karimnezhad 3.D
Sample : M12

Peak Number: 42 at 31.206 min Area: 25252607 Area % 0.18

The 3 best hits from each library.	Ref#	CAS#	Qual
D:\Database\W10N14.L			
1 3-Methyl-4-isopropylphenol	73390	003228-02-2	94
2 3-Methyl-4-isopropylphenol	73391	003228-02-2	94
3 Carvacrol	72968	000499-75-2	94

Unknown Spectrum based on Apex

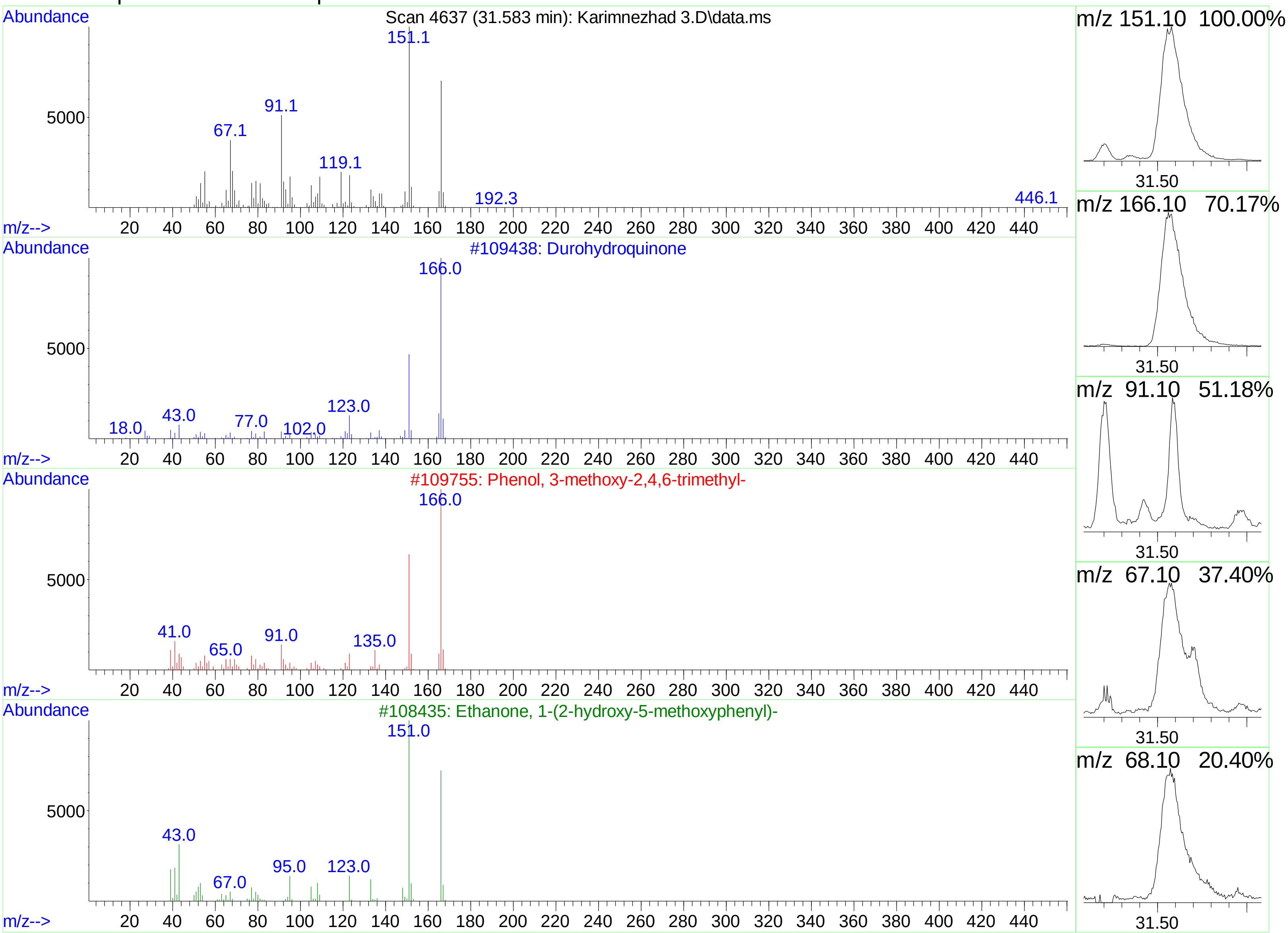

Data File: D:\msdchem\1\data\Karimnezhad 3.D
Sample : M12

Peak Number: 43 at 31.583 min Area: 51566947 Area % 0.38

The 3 best hits from each library.	Ref\#	CAS\#	Qual
D:\Database\W10N14.L			
1 Durohydroquinone	109438	000527-18-4	81
2 Phenol, 3-methoxy-2,4,6-trimethyl-	109755	034883-05-1	76
3 Ethanone, 1-(2-hydroxy-5-methoxy...	108435	000705-15-7	76

Library Search Report - ChemStation Integrator

Unknown Spectrum based on Apex

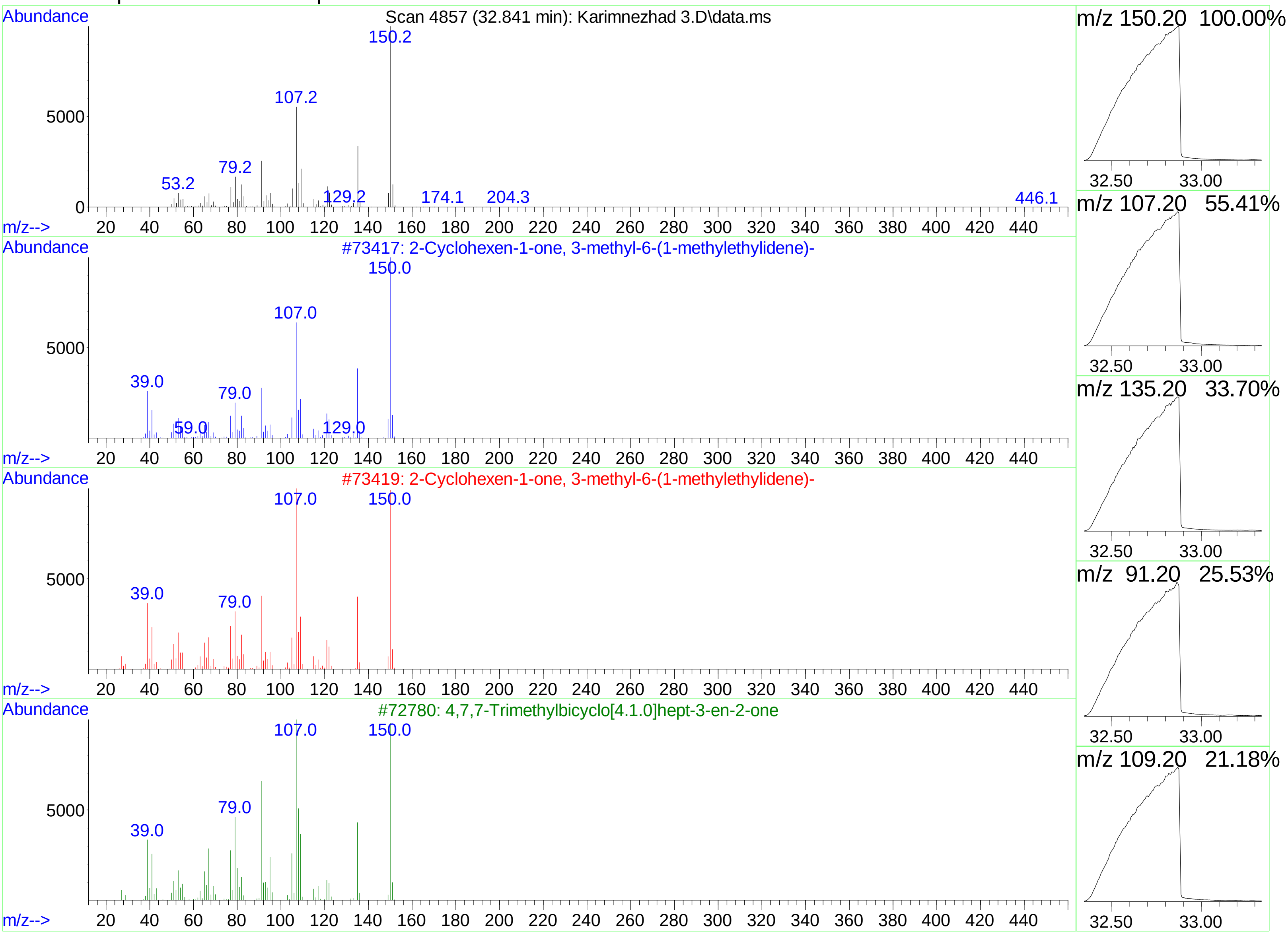

Data File: D:\msdchem\1\data\Karimnezhad 3.D
Sample : M12

Peak Number: 44 at 32.841 min Area: 4731108521 Area % 34.44

The 3 best hits from each library.	Ref#	CAS#	Qual
D:\Database\W10N14.L			
1 2-Cyclohexen-1-one, 3-methyl-6-(...	73417	000491-09-8	98
2 2-Cyclohexen-1-one, 3-methyl-6-(...	73419	000491-09-8	97
3 4,7,7-Trimethylbicyclo[4.1.0]hep...	72780	081800-50-2	93

Unknown Spectrum based on Apex

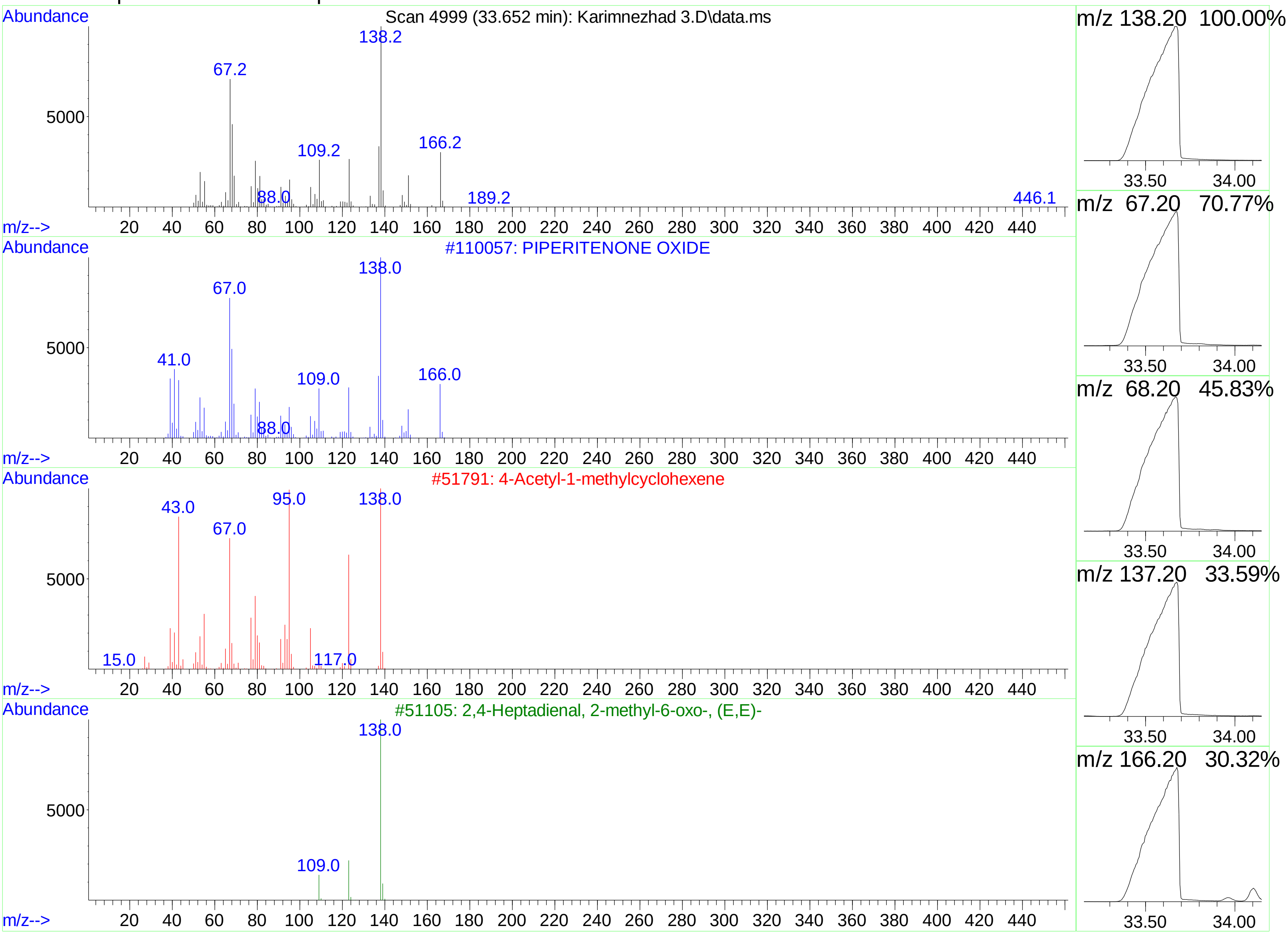

Data File: D:\msdchem\1\data\Karimnezhad 3.D
Sample : M12

Peak Number: 45 at 33.652 min Area: 1887267444 Area % 13.74

The 3 best hits from each library. Ref\# CAS\# Qual

D:\Database\W10N14.L

	Name	Ref#	CAS#	Qual
1	PIPERITENONE OXIDE	110057	003564-96-3	99
2	4-Acetyl-1-methylcyclohexene	51791	006090-09-1	60
3	2,4-Heptadienal, 2-methyl-6-oxo-...	51105	129454-99-5	58

Unknown Spectrum based on Apex

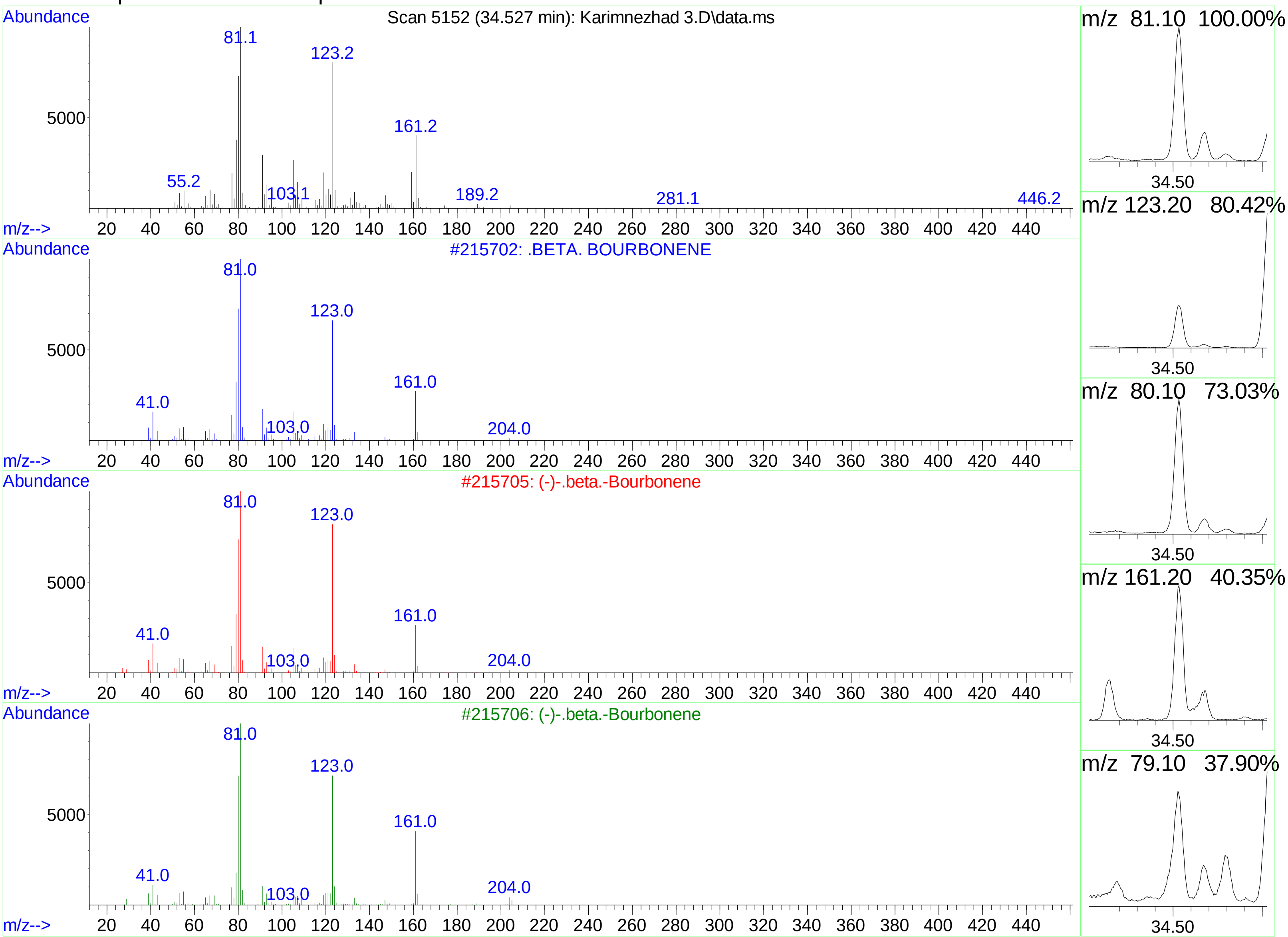

Data File: D:\msdchem\1\data\Karimnezhad 3.D
Sample : M12

Peak Number: 46 at 34.527 min Area: 36180777 Area % 0.26

The 3 best hits from each library.	Ref\#	CAS\#	Qual
D:\Database\W10N14.L			
1 .BETA. BOURBONENE	215702	005208-59-3	96
2 (-)-.beta.-Bourbonene	215705	005208-59-3	96
3 (-)-.beta.-Bourbonene	215706	005208-59-3	87

Unknown Spectrum based on Apex

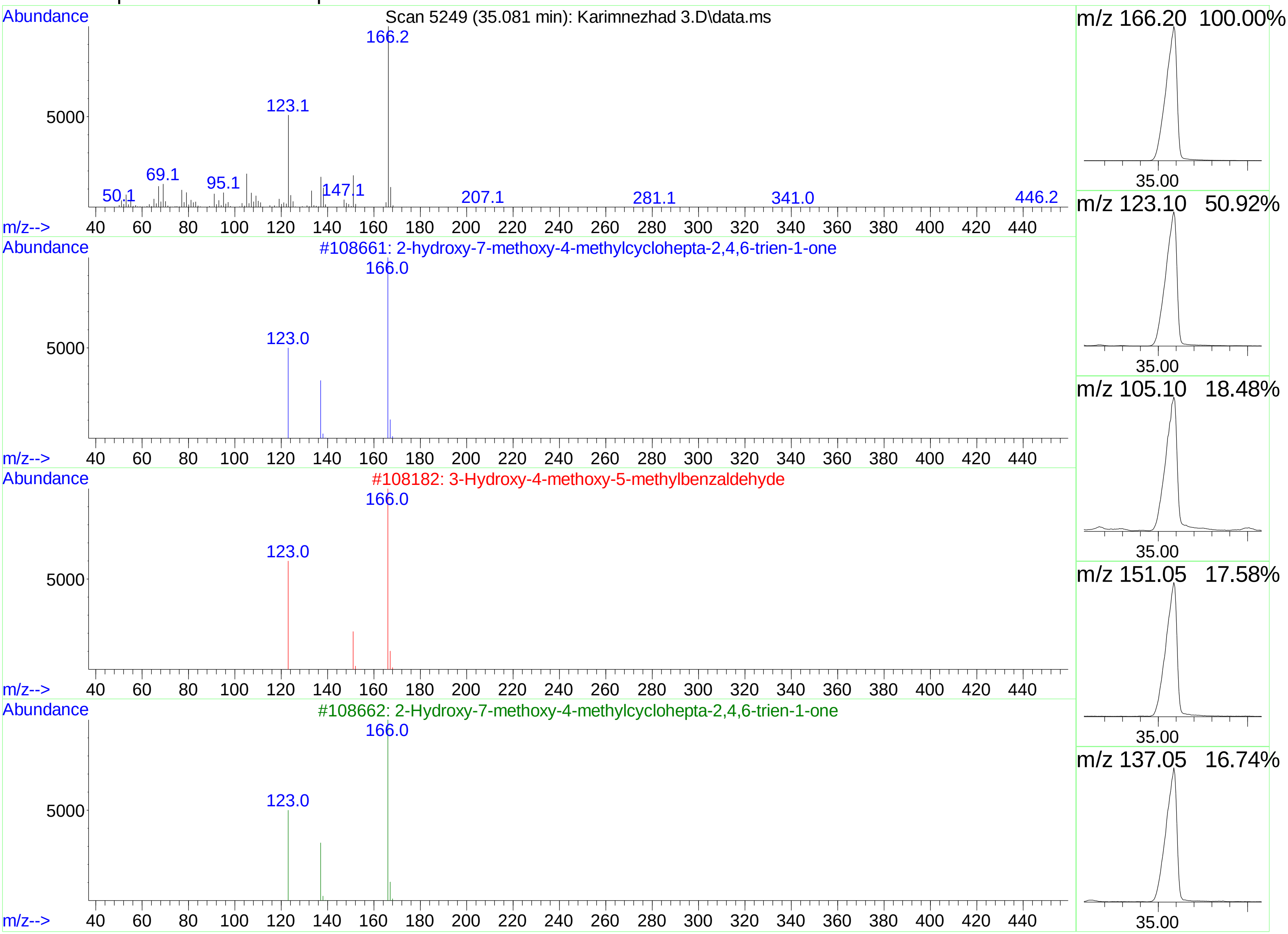

Data File: D:\msdchem\1\data\Karimnezhad 3.D
Sample : M12

Peak Number: 47 at 35.081 min Area: 358301123 Area % 2.61

The 3 best hits from each library. Ref\# CAS\# Qual

D:\Database\W10N14.L

1	2-hydroxy-7-methoxy-4-methylcycl...	108661	2000108-66-1	64
2	3-Hydroxy-4-methoxy-5-methylbenz...	108182	2000108-18-2	64
3	2-Hydroxy-7-methoxy-4-methylcycl...	108662	2000108-66-2	64

Library Search Report - ChemStation Integrator

Unknown Spectrum based on Apex

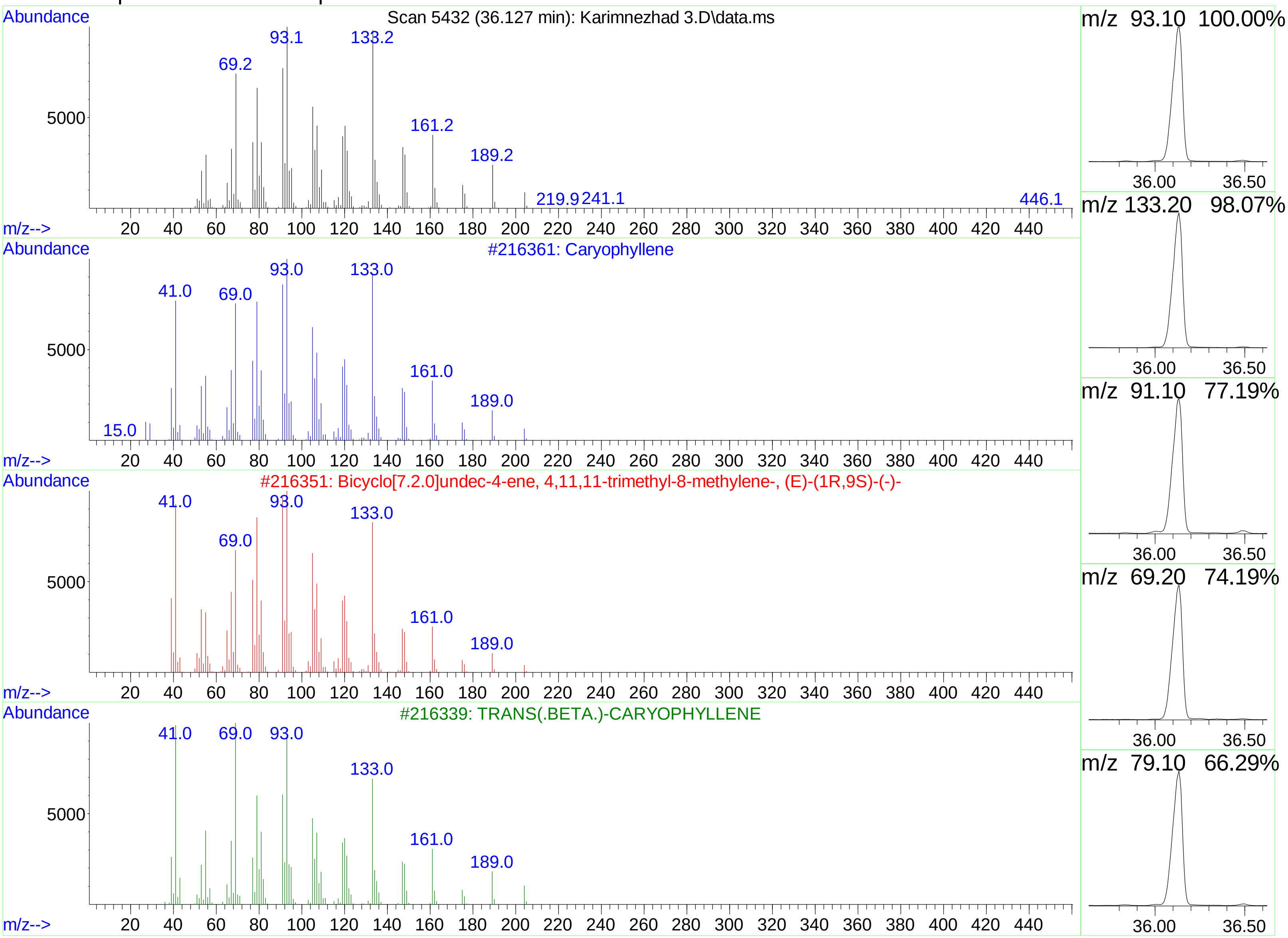

Data File: D:\msdchem\1\data\Karimnezhad 3.D
Sample : M12

Peak Number: 48 at 36.127 min Area: 319437002 Area % 2.33

The 3 best hits from each library.	Ref\#	CAS\#	Qual
D:\Database\W10N14.L			
1 Caryophyllene	216361	000087-44-5	99
2 Bicyclo[7.2.0]undec-4-ene, 4,11,...	216351	000087-44-5	99
3 TRANS(.BETA.)-CARYOPHYLLENE	216339	2000216-33-9	99

Library Search Report - ChemStation Integrator

Unknown Spectrum based on Apex

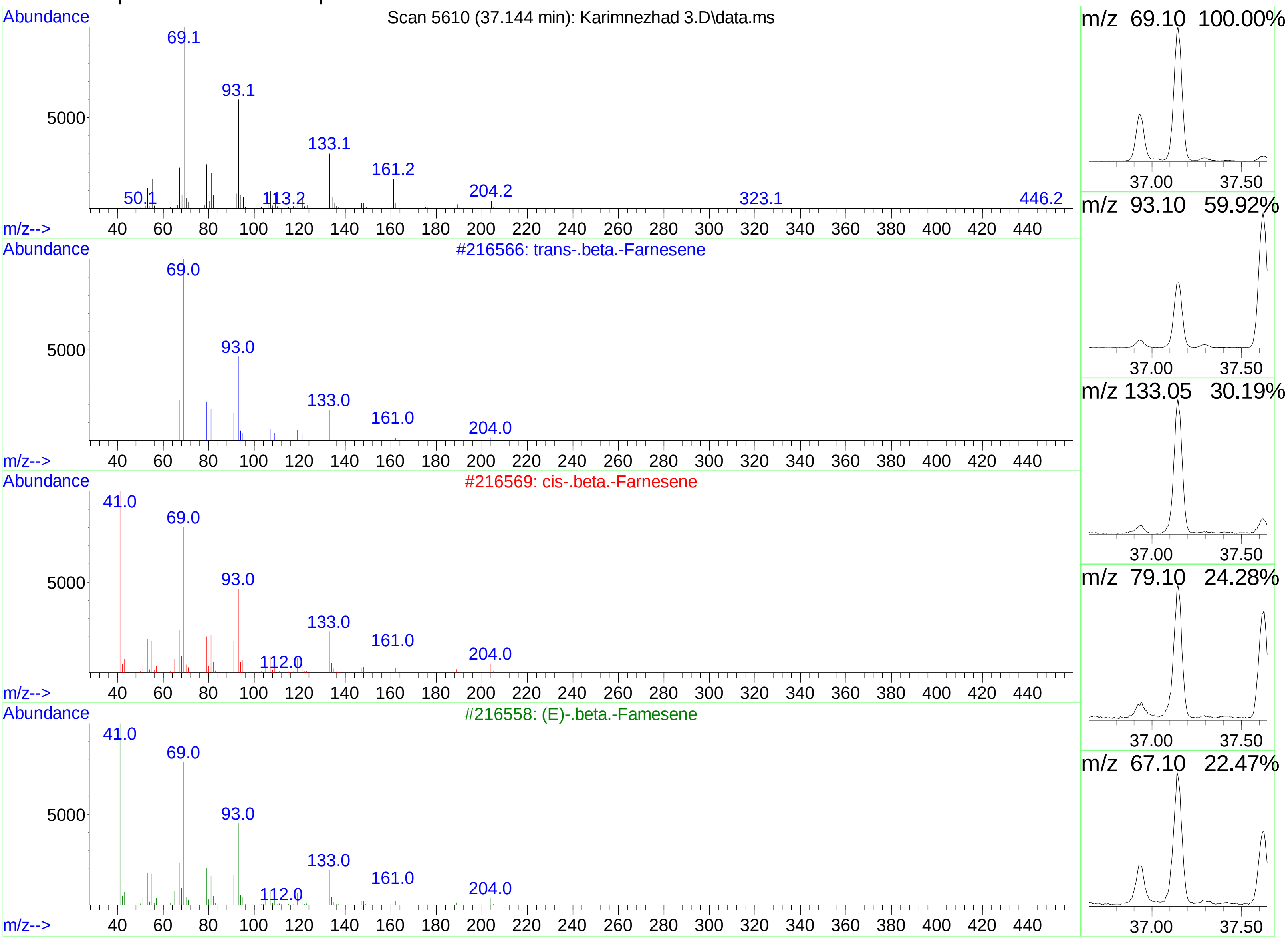

Data File: D:\msdchem\1\data\Karimnezhad 3.D
Sample : M12

Peak Number: 49 at 37.144 min Area: 39615461 Area % 0.29

The 3 best hits from each library. Ref\# CAS\# Qual

D:\Database\W10N14.L

1	trans-.beta.-Farnesene	216566	000502-60-3	97
2	cis-.beta.-Farnesene	216569	028973-97-9	97
3	(E)-.beta.-Famesene	216558	018794-84-8	96

Unknown Spectrum based on Apex

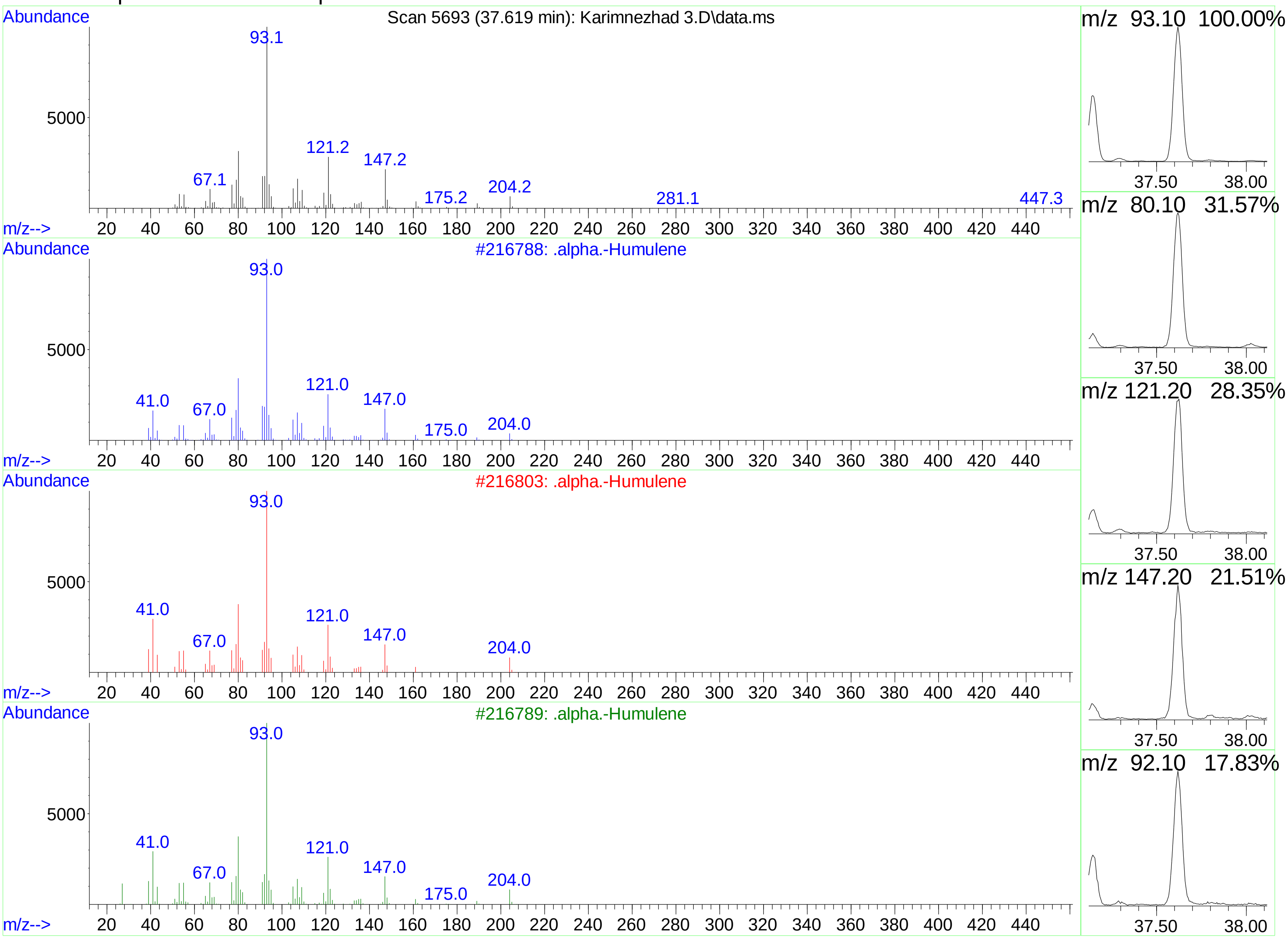

Data File: D:\msdchem\1\data\Karimnezhad 3.D
Sample : M12

Peak Number: 50 at 37.619 min Area: 50013637 Area % 0.36

The 3 best hits from each library.	Ref#	CAS#	Qual
D:\Database\W10N14.L			
1 .alpha.-Humulene	216788	006753-98-6	99
2 .alpha.-Humulene	216803	006753-98-6	98
3 .alpha.-Humulene	216789	006753-98-6	98

Unknown Spectrum based on Apex

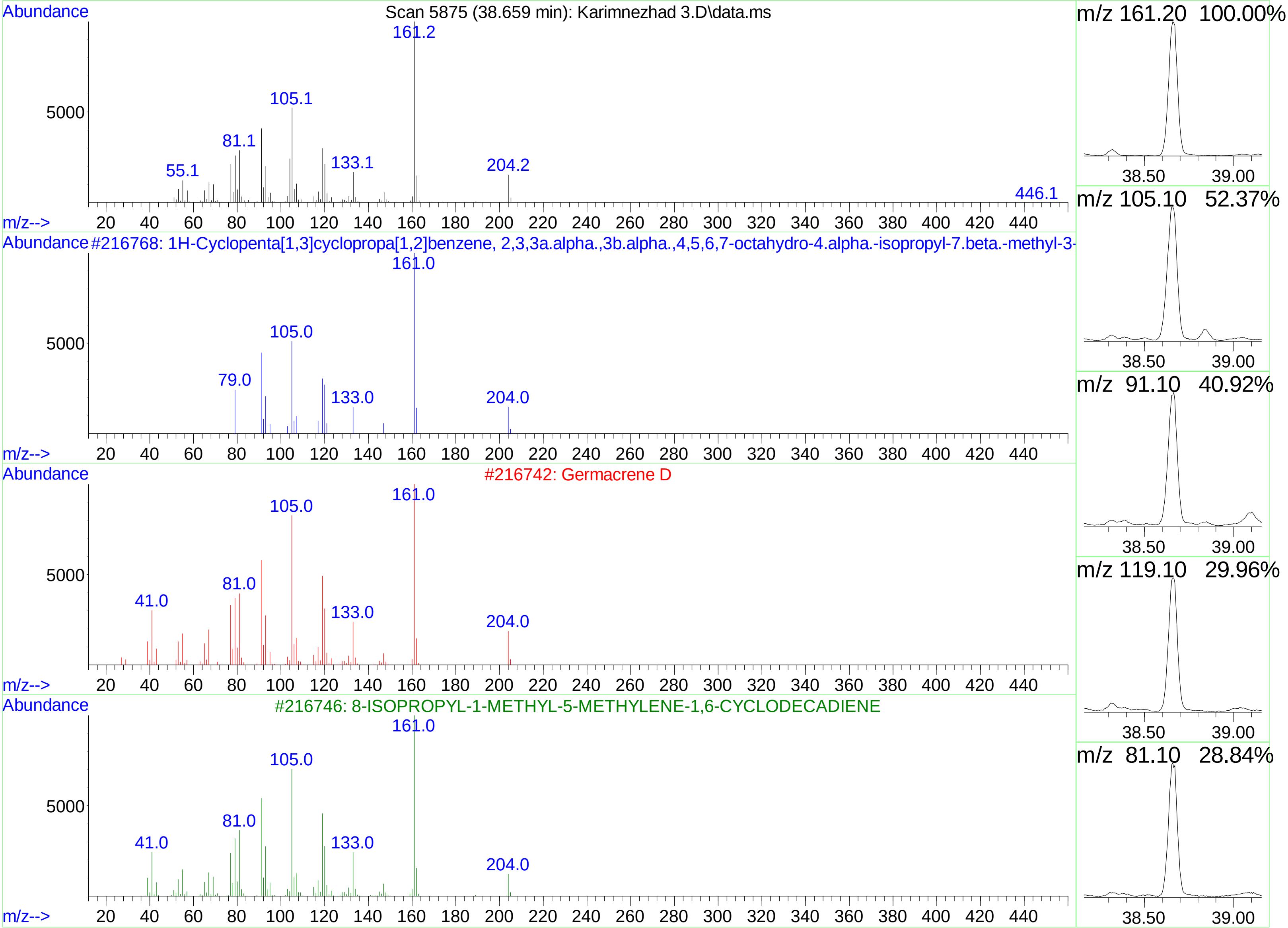

Data File: D:\msdchem\1\data\Karimnezhad 3.D
Sample : M12

Peak Number: 51 at 38.659 min Area: 89583845 Area % 0.65

The 3 best hits from each library.	Ref\#	CAS\#	Qual
D:\Database\W10N14.L			
1 1H-Cyclopenta[1,3]cyclopropa[1,2...	216768	013744-15-5	99
2 Germacrene D	216742	023986-74-5	99
3 8-ISOPROPYL-1-METHYL-5-METHYLENE...	216746	023986-74-5	98

Unknown Spectrum based on Apex

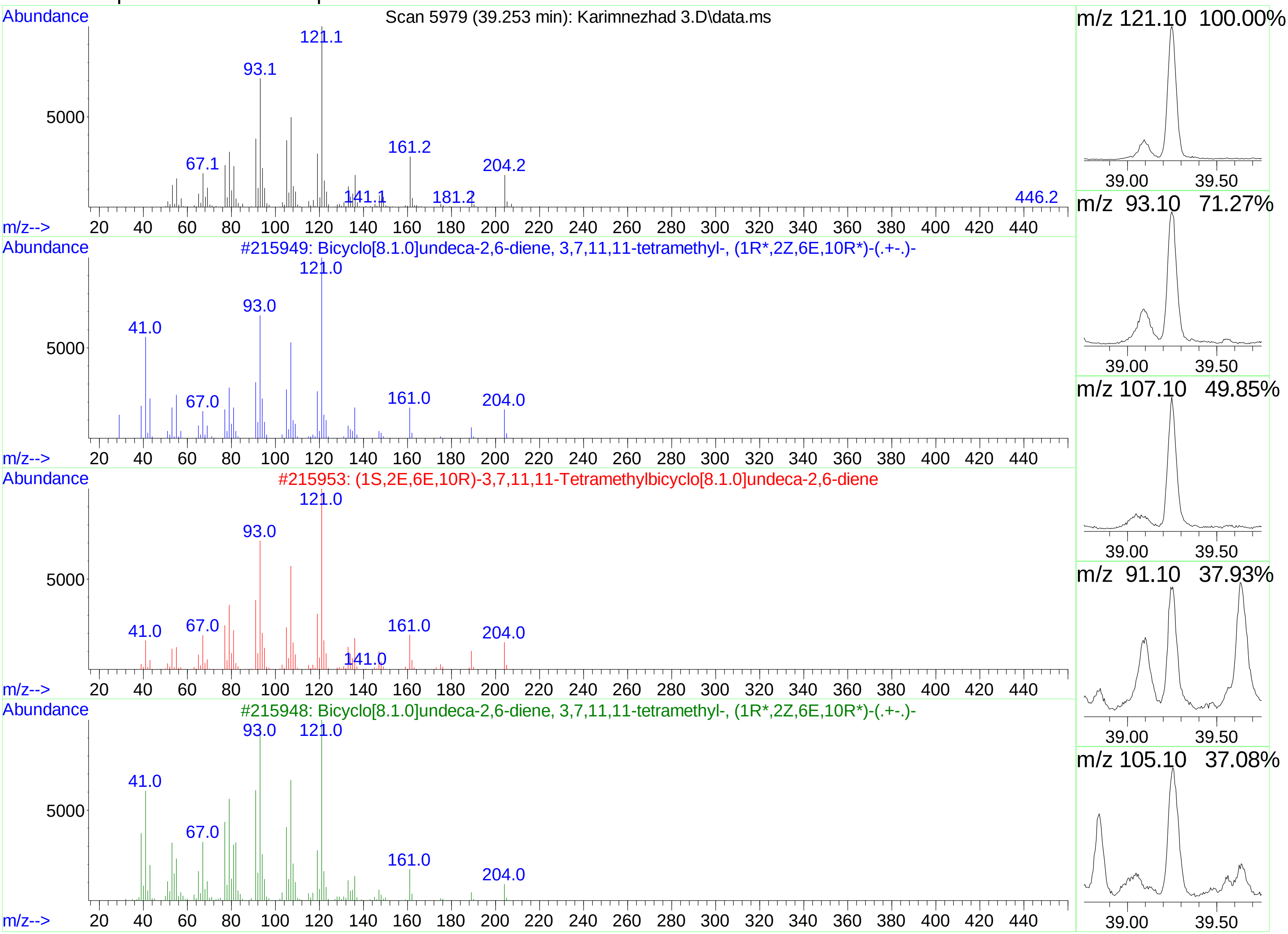

Data File: D:\msdchem\1\data\Karimnezhad 3.D
Sample : M12

Peak Number: 52 at 39.253 min Area: 25604440 Area % 0.19

The 3 best hits from each library. Ref\# CAS\# Qual

D:\Database\W10N14.L

1 Bicyclo[8.1.0]undeca-2,6-diene, ... 215949 100762-46-7 98
2 (1S,2E,6E,10R)-3,7,11,11-Tetrame... 215953 024703-35-3 95
3 Bicyclo[8.1.0]undeca-2,6-diene, ... 215948 100762-46-7 94

Unknown Spectrum based on Apex

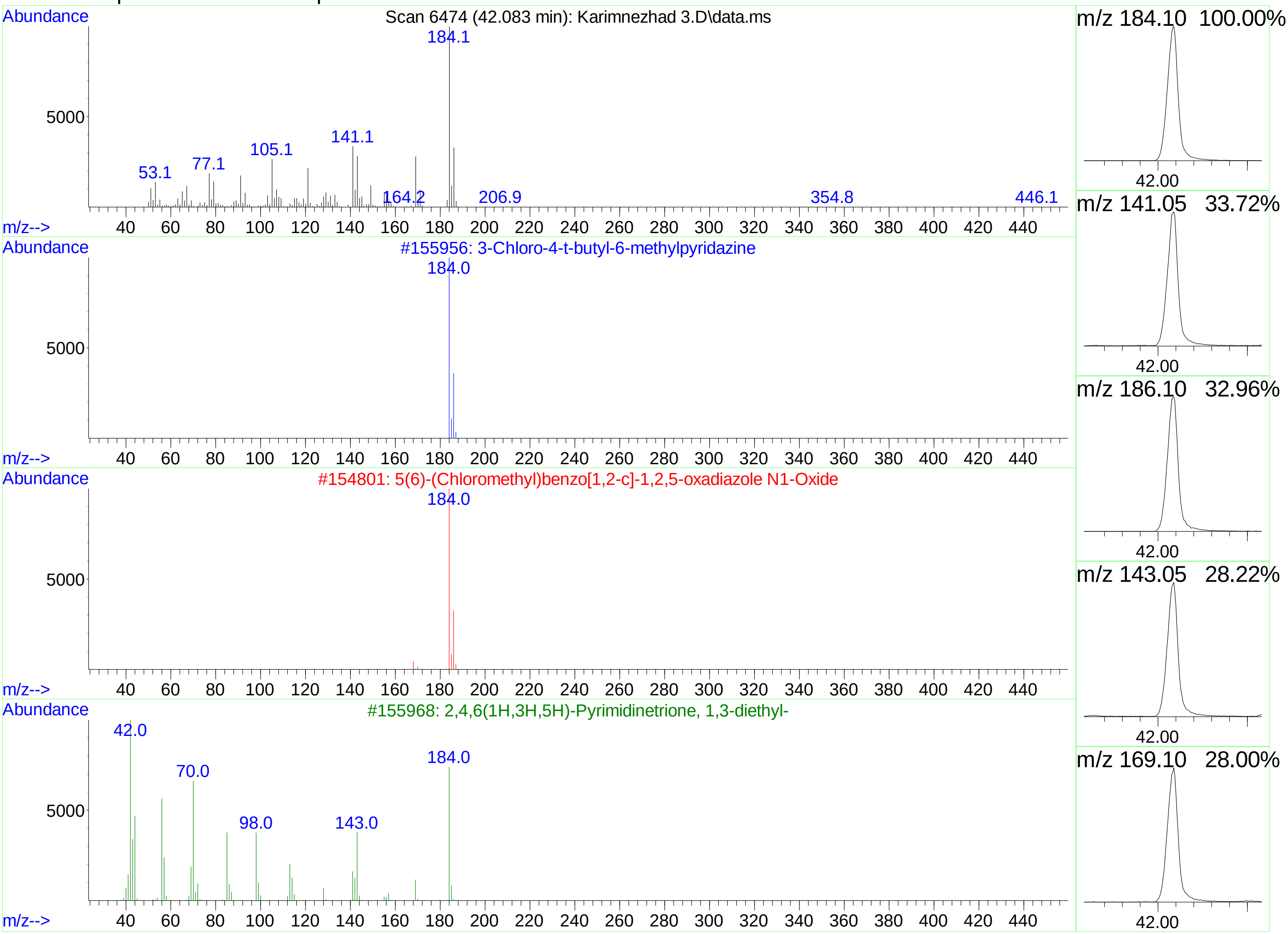

Data File: D:\msdchem\1\data\Karimnezhad 3.D
Sample : M12

Peak Number: 53 at 42.083 min Area: 102069443 Area % 0.74

The 3 best hits from each library. Ref\# CAS\# Qual

D:\Database\W10N14.L

1	3-Chloro-4-t-butyl-6-methylpyrid...	155956	2000155-95-6	83
2	5(6)-(Chloromethyl)benzo[1,2-c]-...	154801	2000154-80-1	59
3	2,4,6(1H,3H,5H)-Pyrimidinetrione...	155968	032479-73-5	58

Unknown Spectrum based on Apex

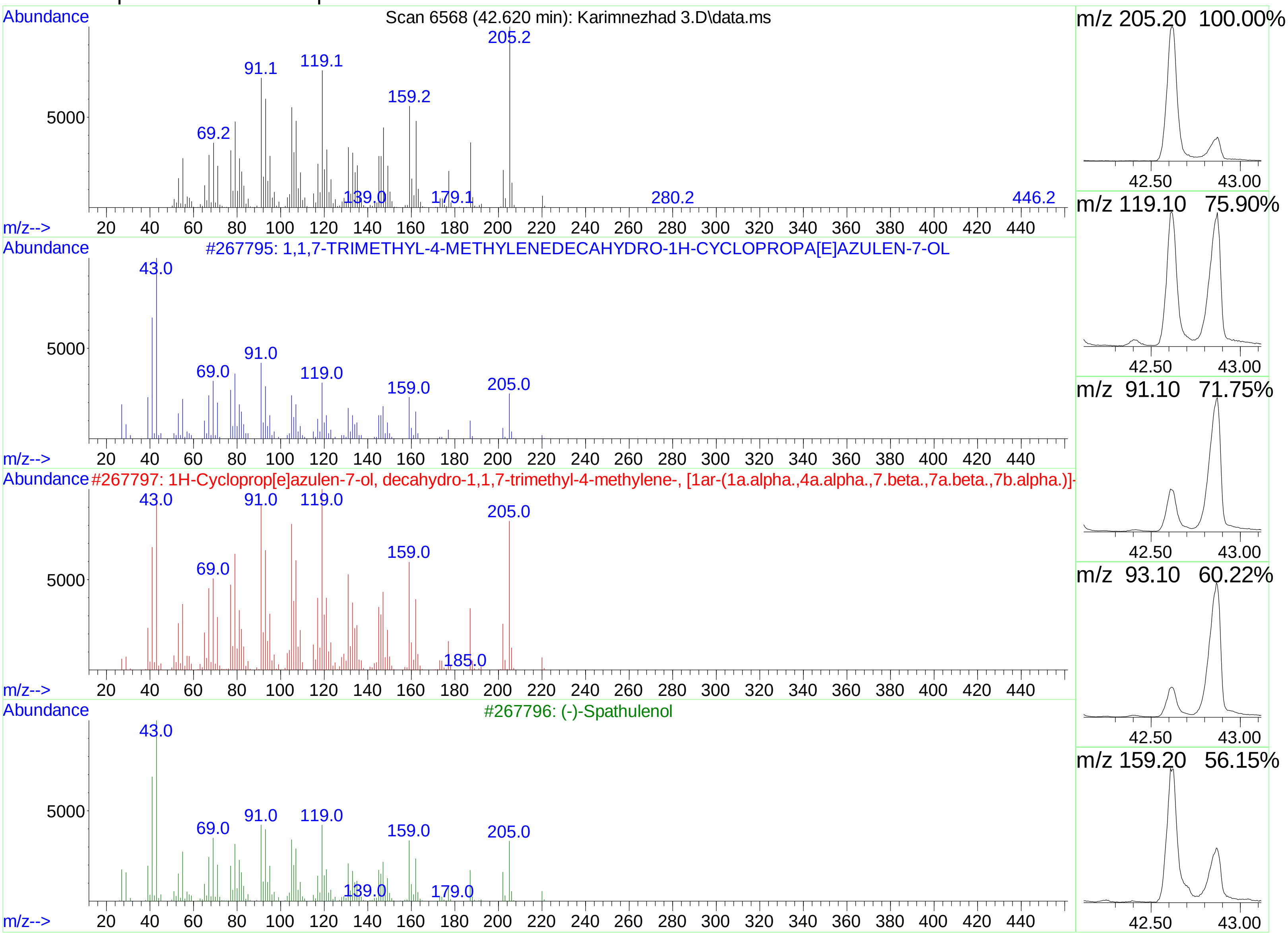

Data File: D:\msdchem\1\data\Karimnezhad 3.D
Sample : M12

Peak Number: 54 at 42.620 min Area: 110484714 Area % 0.80

The 3 best hits from each library. Ref\# CAS\# Qual

D:\Database\W10N14.L

	Name	Ref#	CAS#	Qual
1	1,1,7-TRIMETHYL-4-METHYLENEDECAH...	267795	077171-55-2	98
2	1H-Cycloprop[e]azulen-7-ol, deca...	267797	006750-60-3	98
3	(-)-Spathulenol	267796	077171-55-2	93

Unknown Spectrum based on Apex

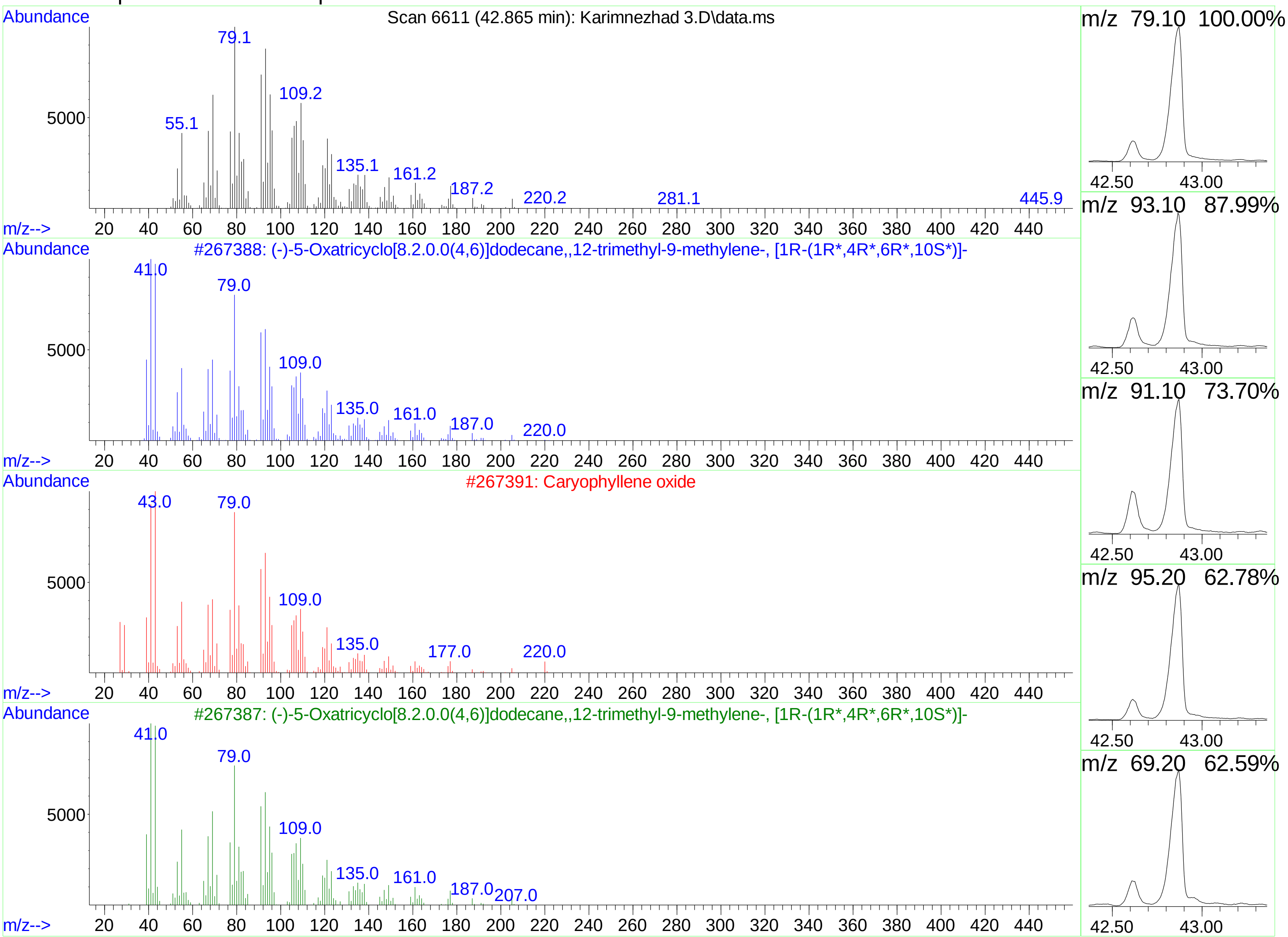

Data File: D:\msdchem\1\data\Karimnezhad 3.D
Sample : M12

Peak Number: 55 at 42.865 min Area: 372265002 Area % 2.71

The 3 best hits from each library.	Ref#	CAS#	Qual
D:\Database\W10N14.L			
1 (-)-5-Oxatricyclo[8.2.0.0(4,6)]d...	267388	001139-30-6	99
2 Caryophyllene oxide	267391	001139-30-6	99
3 (-)-5-Oxatricyclo[8.2.0.0(4,6)]d...	267387	001139-30-6	95

Unknown Spectrum based on Apex

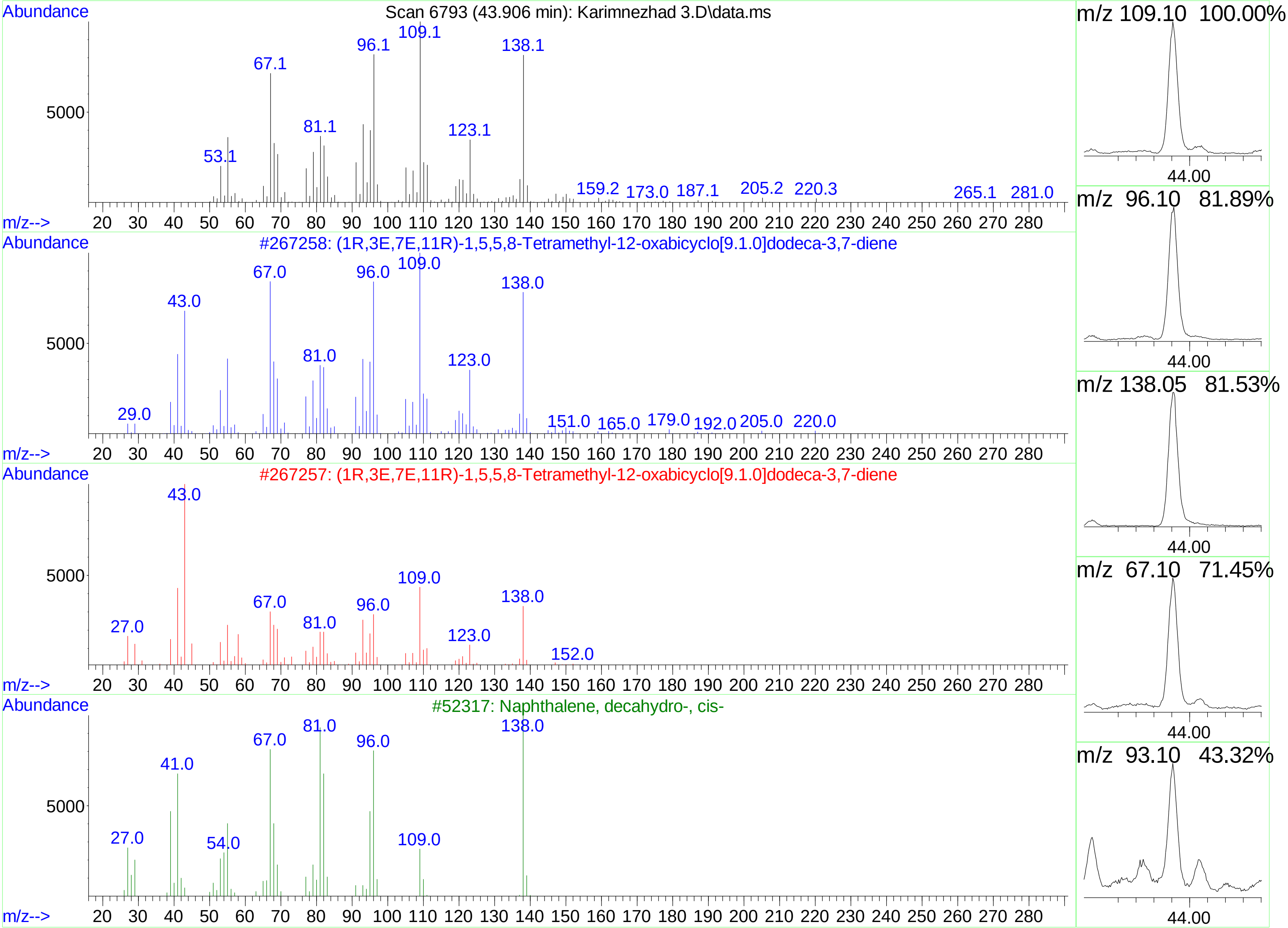

Data File: D:\msdchem\1\data\Karimnezhad 3.D
Sample : M12

Peak Number: 56 at 43.906 min Area: 31749841 Area % 0.23

The 3 best hits from each library. Ref\# CAS\# Qual

D:\Database\W10N14.L

1	(1R,3E,7E,11R)-1,5,5,8-Tetrameth...	267258	019888-34-7	99
2	(1R,3E,7E,11R)-1,5,5,8-Tetrameth...	267257	019888-34-7	87
3	Naphthalene, decahydro-, cis-	52317	000493-01-6	78

Unknown Spectrum based on Apex

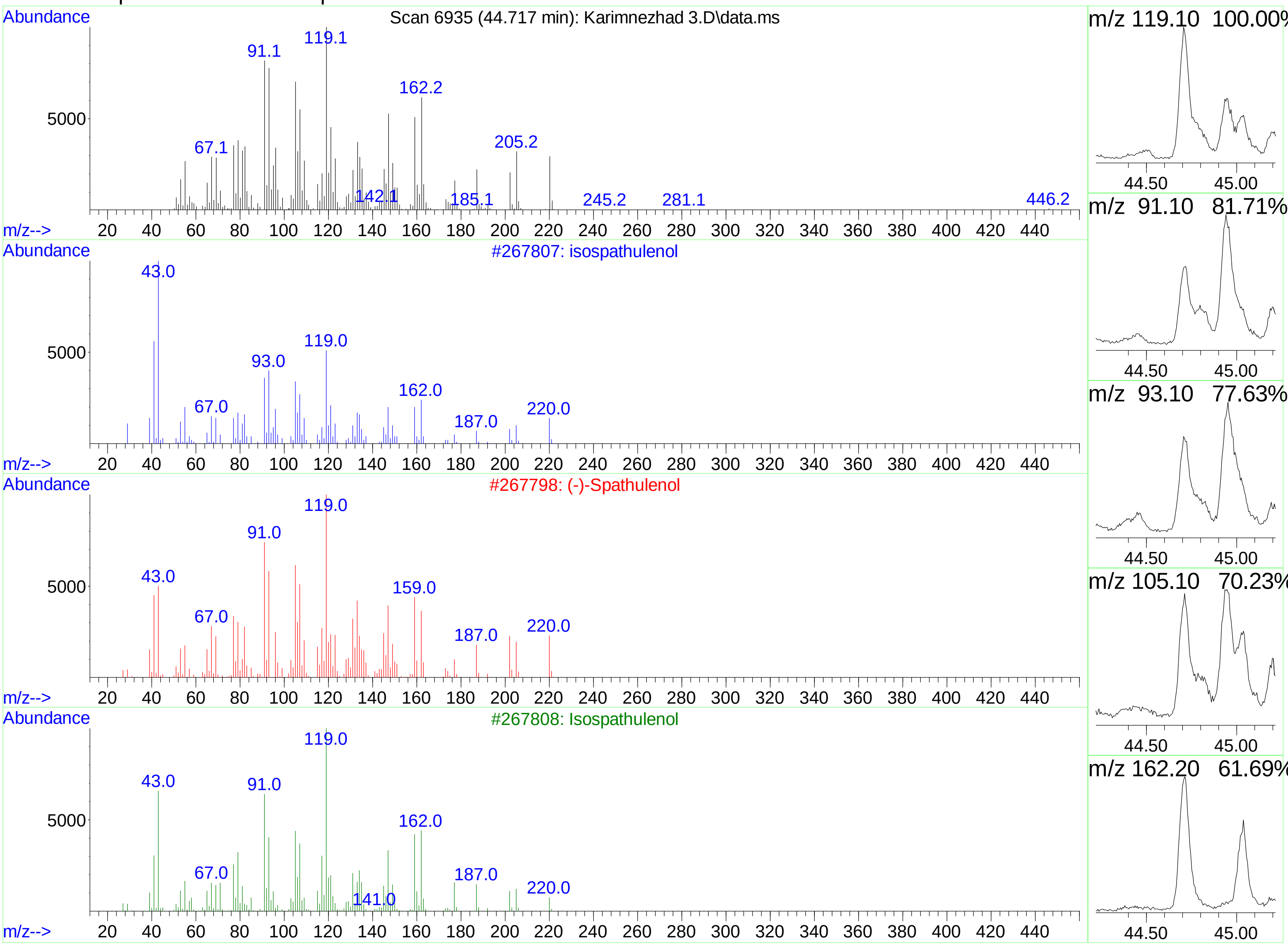

Data File: D:\msdchem\1\data\Karimnezhad 3.D
Sample : M12

Peak Number: 57 at 44.717 min Area: 36648334 Area % 0.27

The 3 best hits from each library.	Ref#	CAS#	Qual
D:\Database\W10N14.L			
1 isospathulenol	267807	2000267-80-7	99
2 (-)-Spathulenol	267798	077171-55-2	86
3 Isospathulenol	267808	088395-46-4	62

Unknown Spectrum based on Apex

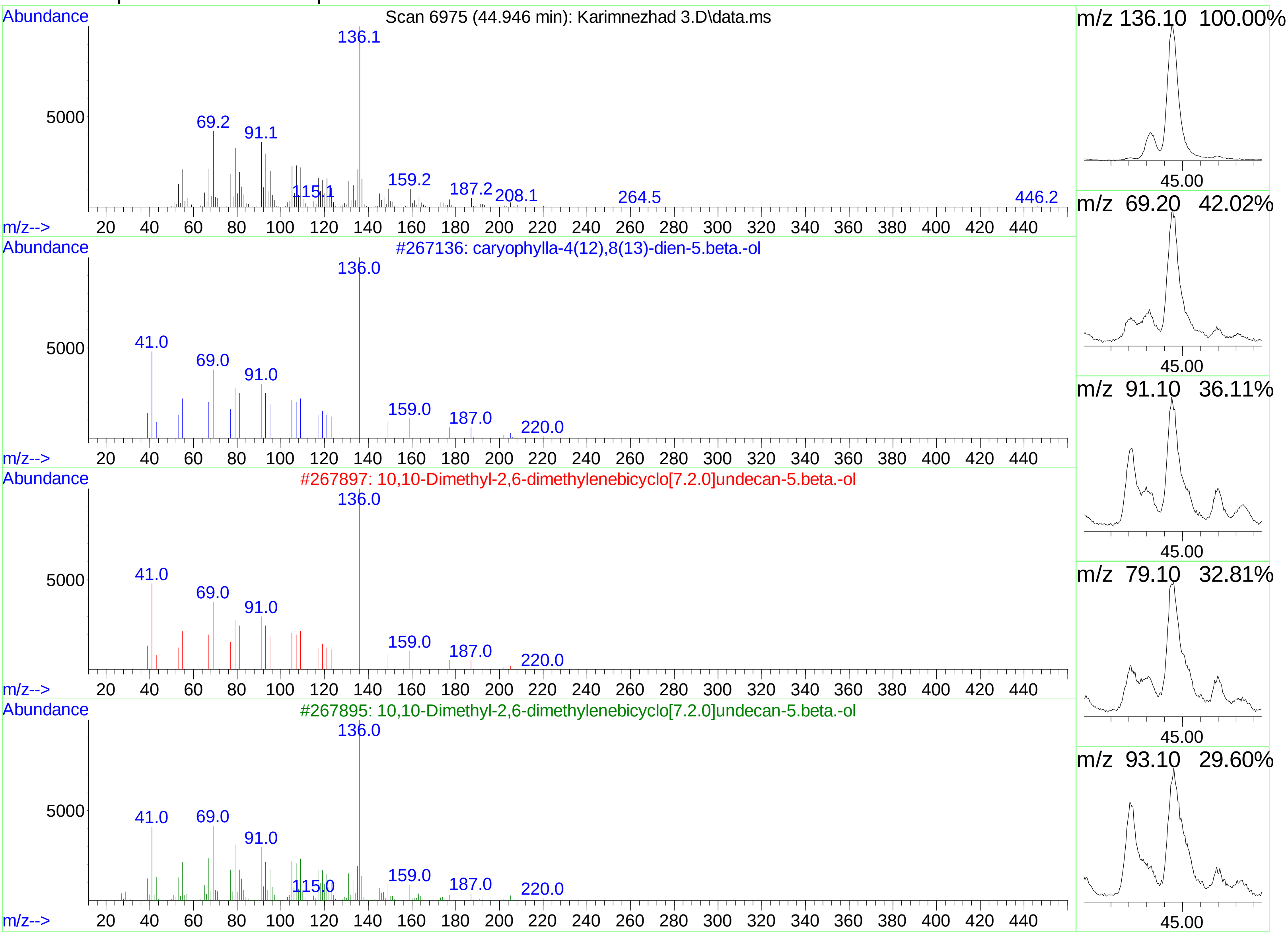

Data File: D:\msdchem\1\data\Karimnezhad 3.D
Sample : M12

Peak Number: 58 at 44.946 min Area: 60927642 Area % 0.44

The 3 best hits from each library.	Ref#	CAS#	Qual
D:\Database\W10N14.L			
1 caryophylla-4(12),8(13)-dien-5.b...	267136	2000267-13-6	98
2 10,10-Dimethyl-2,6-dimethylenebi...	267897	019431-80-2	98
3 10,10-Dimethyl-2,6-dimethylenebi...	267895	019431-80-2	96

Unknown Spectrum based on Apex

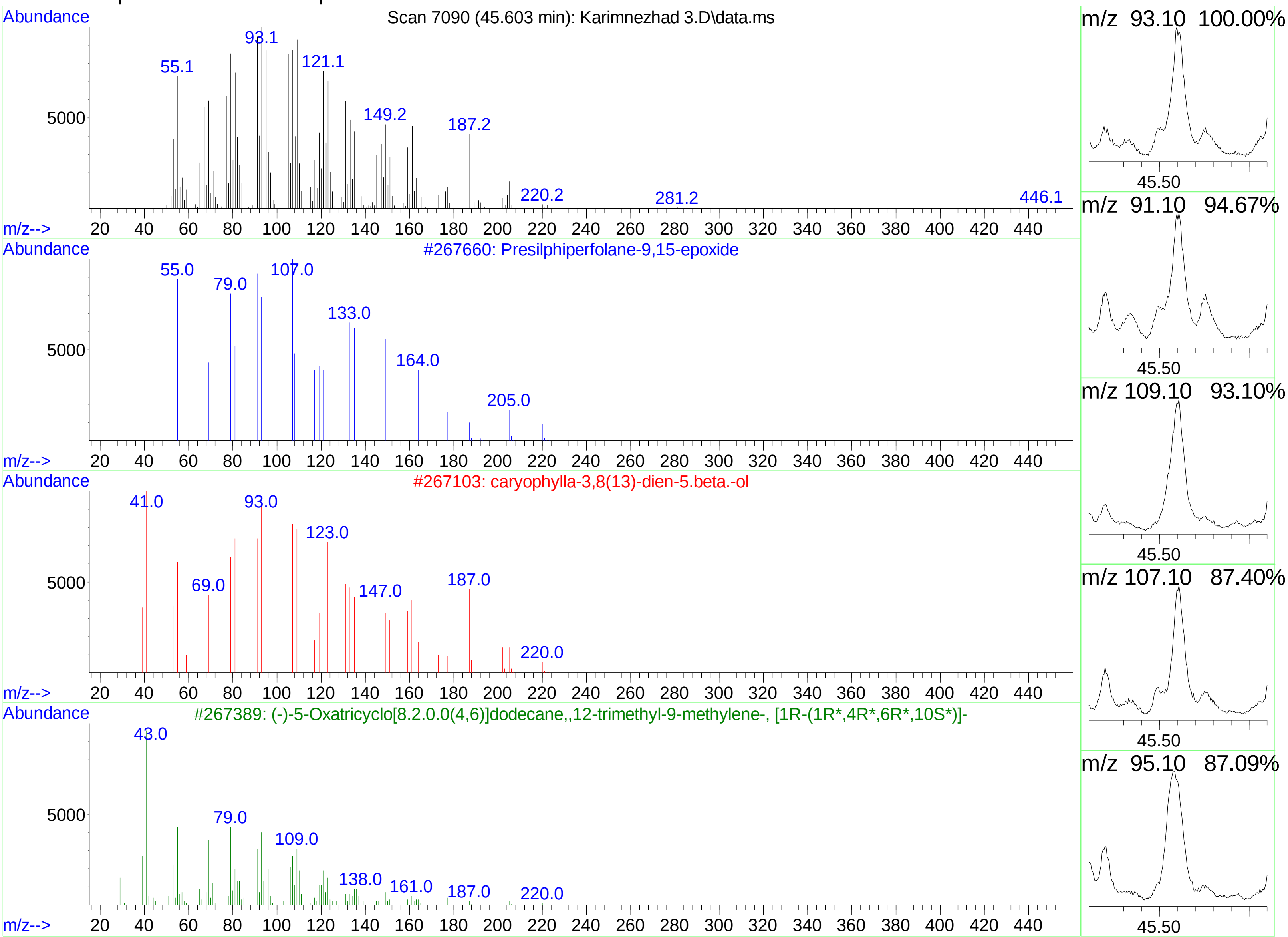

Data File: D:\msdchem\1\data\Karimnezhad 3.D
Sample : M12

Peak Number: 59 at 45.603 min Area: 47104976 Area % 0.34

The 3 best hits from each library.	Ref#	CAS#	Qual
D:\Database\W10N14.L			
1 Presilphiperfolane-9,15-epoxide	267660	2000267-66-0	95
2 caryophylla-3,8(13)-dien-5.beta.-ol	267103	2000267-10-3	64
3 (-)-5-Oxatricyclo[8.2.0.0(4,6)]d...	267389	001139-30-6	62

Unknown Spectrum based on Apex

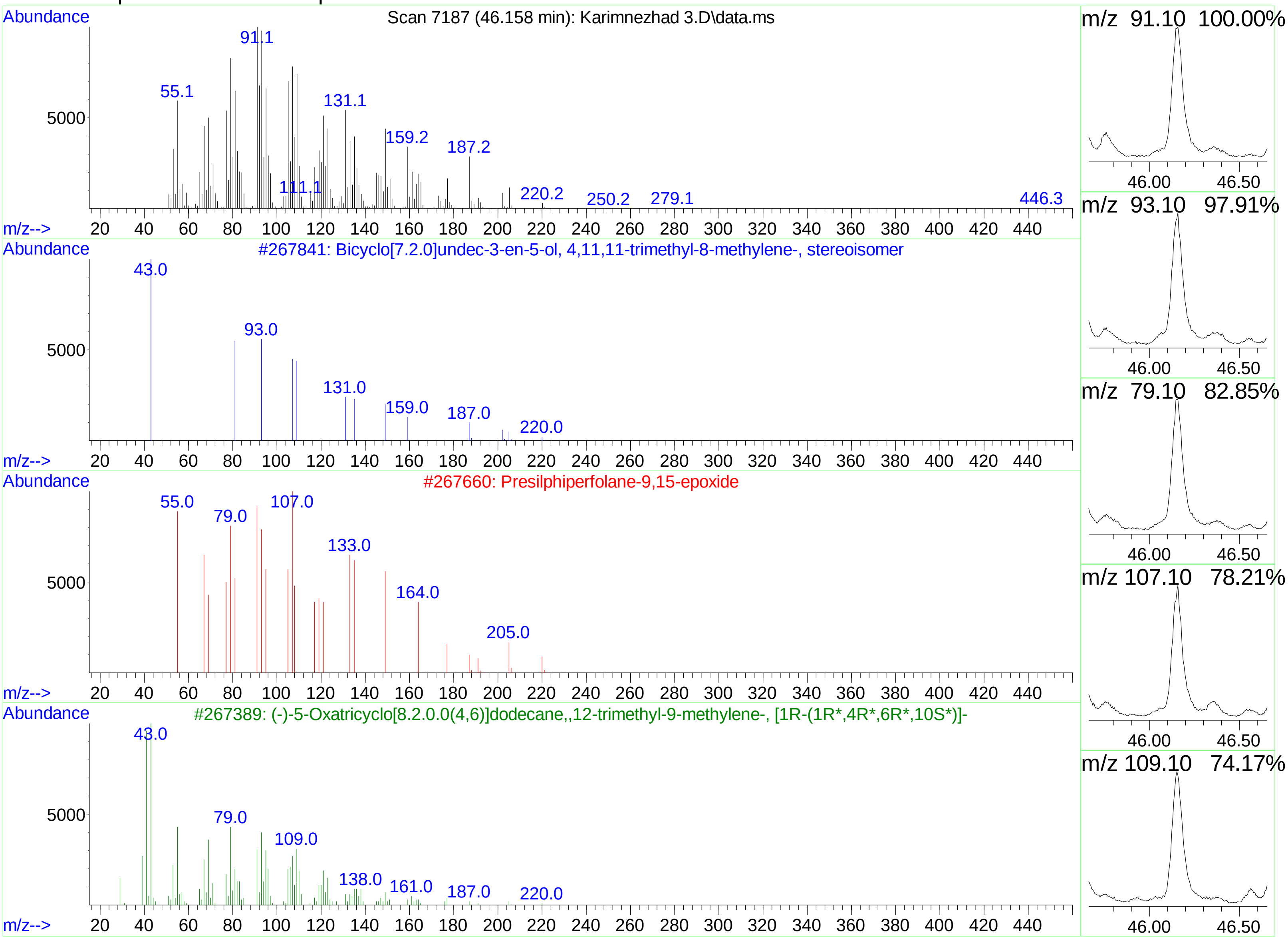

Data File: D:\msdchem\1\data\Karimnezhad 3.D
Sample : M12

Peak Number: 60 at 46.158 min Area: 64223030 Area % 0.47

The 3 best hits from each library.	Ref#	CAS#	Qual
D:\Database\W10N14.L			
1 Bicyclo[7.2.0]undec-3-en-5-ol, 4...	267841	032214-89-4	90
2 Presilphiperfolane-9,15-epoxide	267660	2000267-66-0	83
3 (-)-5-Oxatricyclo[8.2.0.0(4,6)]d...	267389	001139-30-6	83

Unknown Spectrum based on Apex

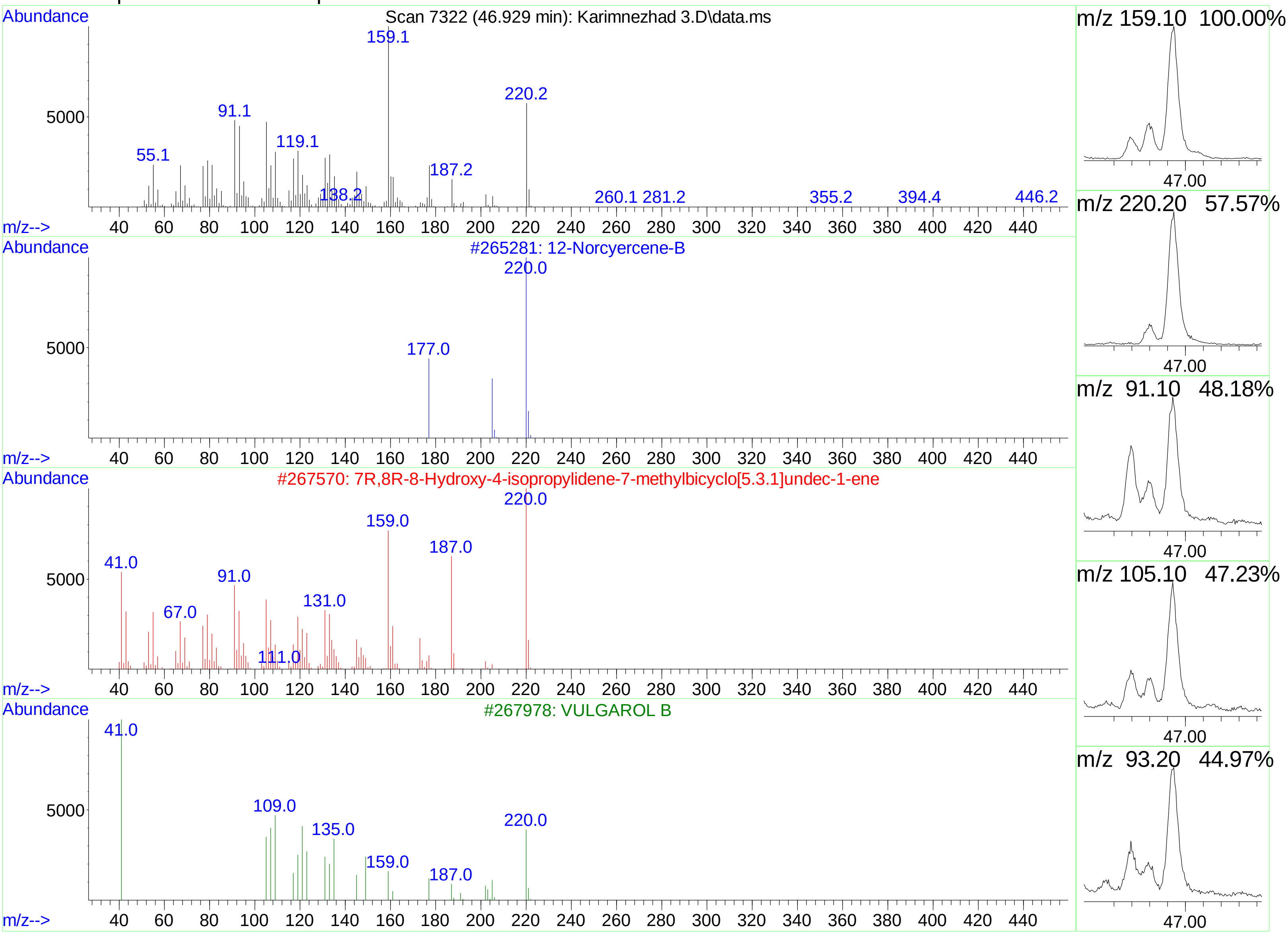

Data File: D:\msdchem\1\data\Karimnezhad 3.D
Sample : M12

Peak Number: 61 at 46.929 min Area: 28717645 Area % 0.21

The 3 best hits from each library.	Ref#	CAS#	Qual
D:\Database\W10N14.L			
1 12-Norcyercene-B	265281	2000265-28-1	90
2 7R,8R-8-Hydroxy-4-isopropylidene...	267570	161362-94-3	49
3 VULGAROL B	267978	011056-03-4	43

Library Search Report - ChemStation Integrator

Unknown Spectrum based on Apex

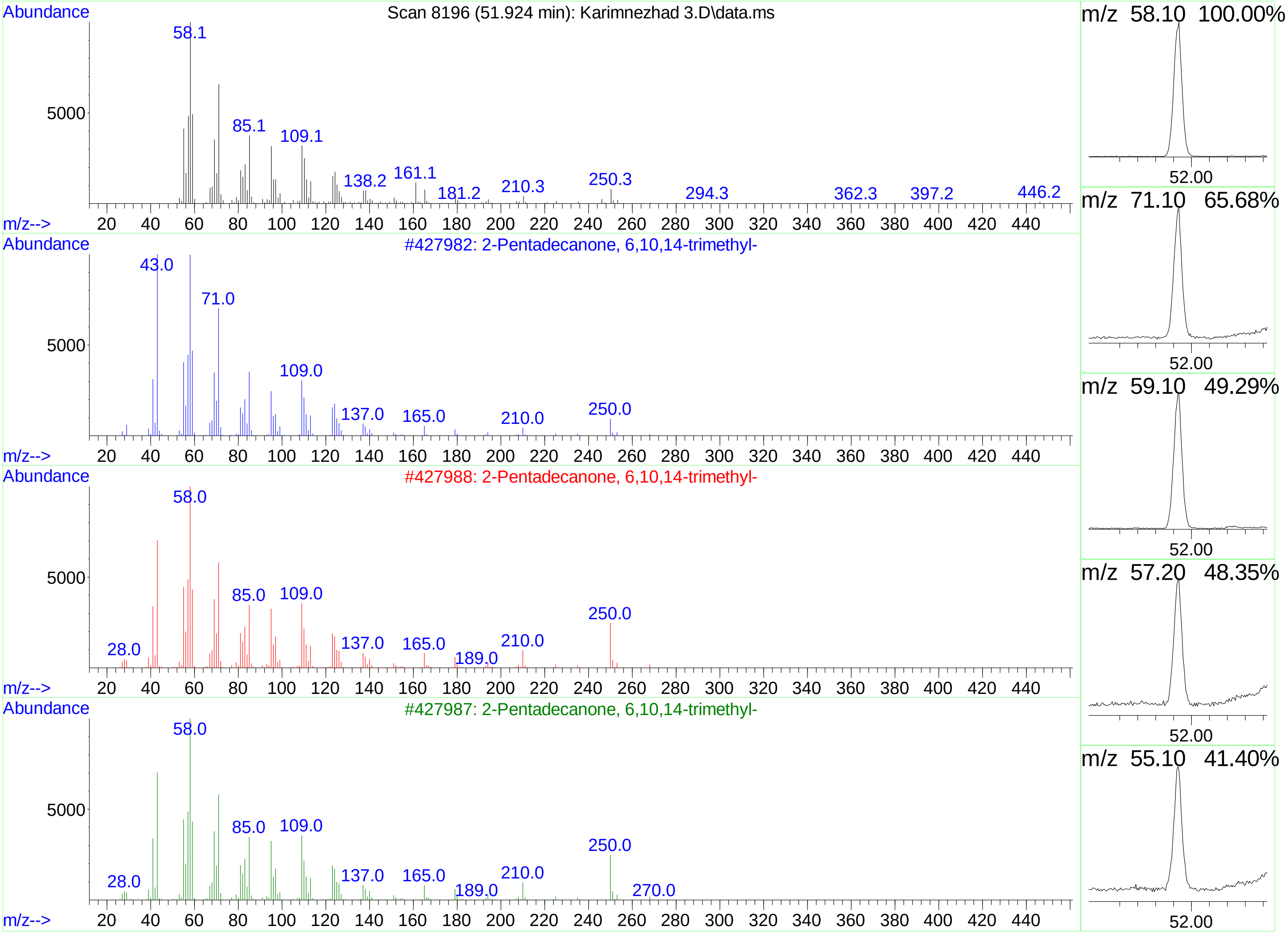

Data File: D:\msdchem\1\data\Karimnezhad 3.D
Sample : M12

Peak Number: 62 at 51.924 min Area: 21160560 Area % 0.15

The 3 best hits from each library.	Ref#	CAS#	Qual
D:\Database\W10N14.L			
1 2-Pentadecanone, 6,10,14-trimethyl-	427982	000502-69-2	99
2 2-Pentadecanone, 6,10,14-trimethyl-	427988	000502-69-2	94
3 2-Pentadecanone, 6,10,14-trimethyl-	427987	000502-69-2	94

Unknown Spectrum based on Apex

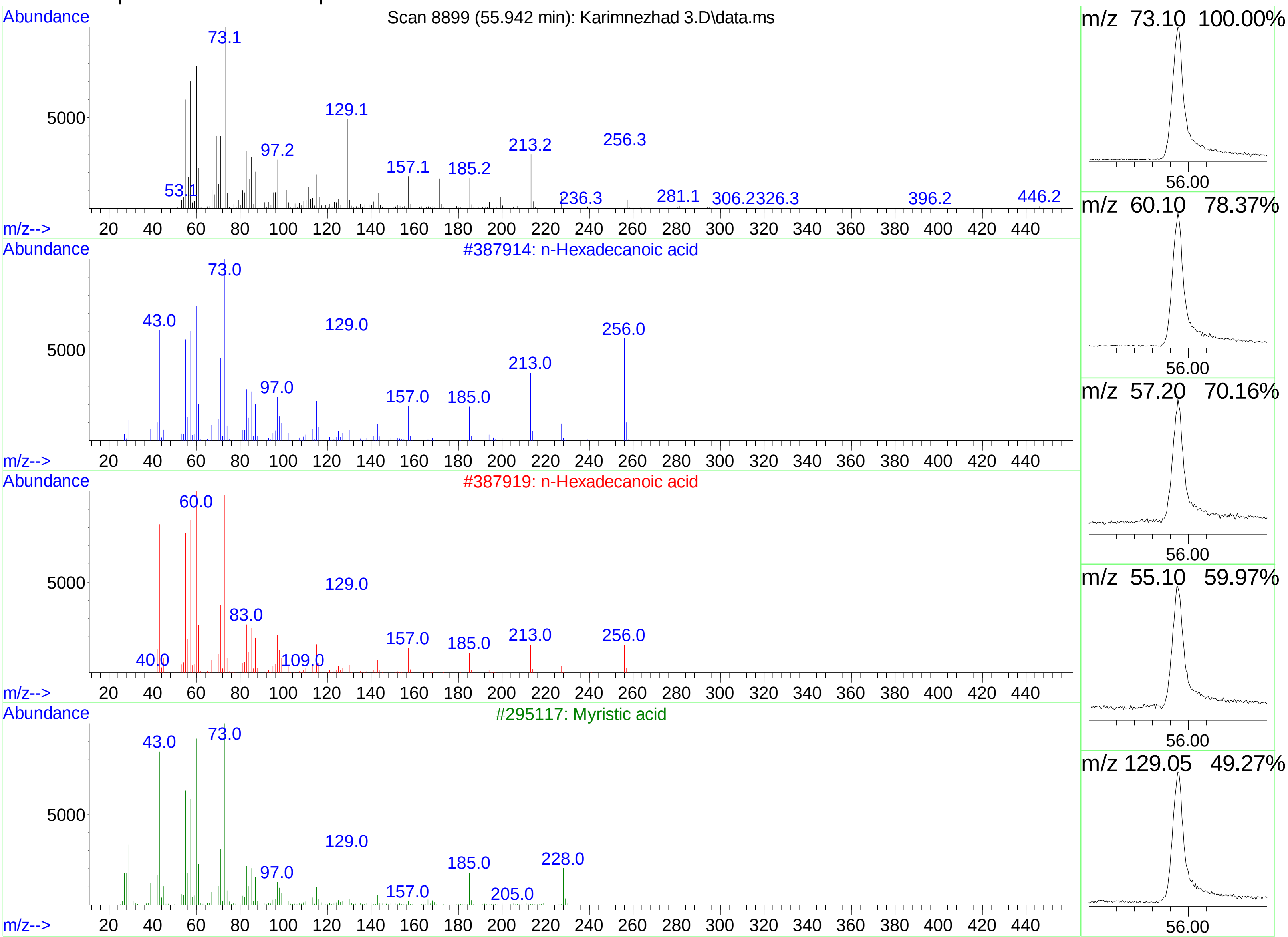

Data File: D:\msdchem\1\data\Karimnezhad 3.D
Sample : M12

Peak Number: 63 at 55.942 min Area: 31005923 Area % 0.23

The 3 best hits from each library.	Ref\#	CAS\#	Qual
D:\Database\W10N14.L			
1 n-Hexadecanoic acid	387914	000057-10-3	99
2 n-Hexadecanoic acid	387919	000057-10-3	99
3 Myristic acid	295117	000544-63-8	97

Unknown Spectrum based on Apex

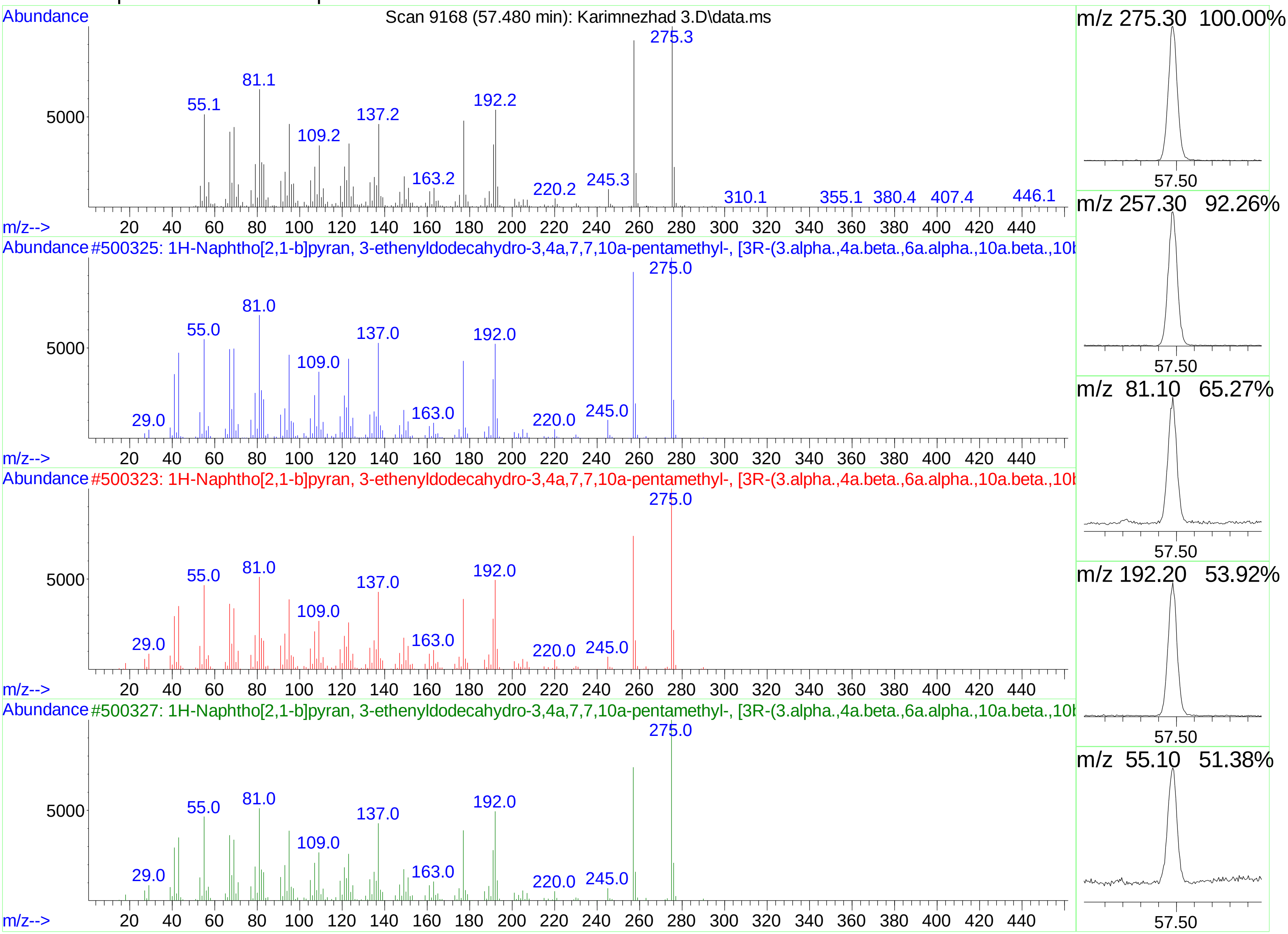

Data File: D:\msdchem\1\data\Karimnezhad 3.D
Sample : M12

Peak Number: 64 at 57.480 min Area: 32122033 Area % 0.23

The 3 best hits from each library. Ref\# CAS\# Qual

D:\Database\W10N14.L

		Ref#	CAS#	Qual
1	1H-Naphtho[2,1-b]pyran, 3-etheny...	500325	000596-84-9	99
2	1H-Naphtho[2,1-b]pyran, 3-etheny...	500323	000596-84-9	94
3	1H-Naphtho[2,1-b]pyran, 3-etheny...	500327	000596-84-9	94
