# Supplementary material for: Comparison of quantity, quality and antibacterial activity of essential oil Mentha longifolia (L.) L. under different traditional and modern extraction methods
Source: PLoS One. 2024 Jul 10;19(7):e0301558. doi: 10.1371/journal.pone.0301558 (PMC11236116; doi:10.1371/journal.pone.0301558)
Supplement: S2 File — (ZIP) [file pone.0301558.s002.zip › Karimnezhad/M14/PrintText+summery.pdf]

Library Search Report

Data Path : D:\msdchem\1\data\
Data File : Karimnezhad 4.D
Acq On : 15 Mar 2022 10:08
Operator : Jafari
Sample : M14
Misc :
ALS Vial : 32 Sample Multiplier: 1

Search Libraries: D:\Database\W10N14.L Minimum Quality: 0

Unknown Spectrum: Apex
Integration Events: ChemStation Integrator - events.e

Pk#	RT	Area%	Library/ID	Ref#	CAS#	Qual
1	11.552	0.05	D:\Database\W10N14.L			
			Furan, 2,5-diethyltetrahydro-	36992	041239-48-9	95
			Furan, 2,5-diethyltetrahydro-	36996	041239-48-9	64
			Furan, 2,5-diethyltetrahydro-	36993	041239-48-9	60
2	13.243	0.76	D:\Database\W10N14.L			
			2-Pinene	49092	000080-56-8	97
			2-Pinene	49090	000080-56-8	96
			(1S)-2,6,6-Trimethylbicyclo[3.1.1]hept-2-ene	49099	007785-26-4	96
3	14.015	0.25	D:\Database\W10N14.L			
			2,2-dimethyl-3-methylene-bicyclo [2.2.1]heptane	49190	000079-92-5	97
			2,2-dimethyl-3-methylene-bicyclo [2.2.1]heptane	49207	000079-92-5	97
			2,2-dimethyl-3-methylene-bicyclo [2.2.1]heptane	49205	000079-92-5	96
4	14.986	0.53	D:\Database\W10N14.L			
			4(10)-Thujene	48867	003387-41-5	97
			Bicyclo[3.1.0]hexane, 4-methylene-1-(1-methylethyl)-	48855	003387-41-5	96
			4(10)-Thujene	48857	003387-41-5	96
5	15.284	1.00	D:\Database\W10N14.L			
			4(10)-Thujene	48862	003387-41-5	94
			2(10)-Pinene	48535	000127-91-3	94
			2(10)-Pinene, (1S,5S)-(-)-	48549	018172-67-3	94
6	15.615	0.22	D:\Database\W10N14.L			
			.beta.-Myrcene	48617	000123-35-3	96
			1,6-Octadiene, 7-methyl-3-methylene-	48627	000123-35-3	95
			1,6-Octadiene, 7-methyl-3-methylene-	48620	000123-35-3	94
7	15.992	0.27	D:\Database\W10N14.L			
			3-Octanol	40544	000589-98-0	90
			ETHYL AMYL CARBINOL	40533	000589-98-0	83
			3-Octanol	40543	000589-98-0	83
8	17.038	0.09	D:\Database\W10N14.L			

Library Search Report

Data Path : D:\msdchem\1\data\
Data File : Karimnezhad 4.D
Acq On : 15 Mar 2022 10:08
Operator : Jafari
Sample : M14
Misc :
ALS Vial : 32 Sample Multiplier: 1

Search Libraries: D:\Database\W10N14.L Minimum Quality: 0

Unknown Spectrum: Apex
Integration Events: ChemStation Integrator - events.e

Pk#	RT	Area%	Library/ID	Ref#	CAS#	Qual
			1,3-Cyclohexadiene, 1-methyl-4-(1-methylethyl)-	48422	000099-86-5	98
			1,3-Cyclohexadiene, 1-methyl-4-(1-methylethyl)-	48418	000099-86-5	97
			Cyclohexene, 1-methyl-4-(1-methylethylidene)-	48893	000586-62-9	97
9	17.415	0.09	D:\Database\W10N14.L			
			o-Cymene	45121	000527-84-4	97
			Benzene, 1-methyl-4-(1-methylethyl)-	45372	000099-87-6	95
			Benzene, 1-methyl-2-(1-methylethyl)-	45118	000527-84-4	95
10	17.661	0.82	D:\Database\W10N14.L			
			D-Limonene	48457	005989-27-5	99
			Cyclohexene, 1-methyl-4-(1-methylethenyl)-	48442	000138-86-3	98
			Cyclohexene, 1-methyl-4-(1-methylethenyl)-	48478	000138-86-3	98
11	17.878	3.27	D:\Database\W10N14.L			
			2-Oxabicyclo[2.2.2]octane, 1,3,3-trimethyl-	83024	000470-82-6	98
			2-Oxabicyclo[2.2.2]octane, 1,3,3-trimethyl-	83030	000470-82-6	98
			2-Oxabicyclo[2.2.2]octane, 1,3,3-trimethyl-	83023	000470-82-6	96
12	19.004	0.18	D:\Database\W10N14.L			
			1,4-Cyclohexadiene, 1-methyl-4-(1-methylethyl)-	48401	000099-85-4	97
			1,4-Cyclohexadiene, 1-methyl-4-(1-methylethyl)-	48392	000099-85-4	96
			.gamma.-Terpinene	48386	000099-85-4	96
13	19.673	0.37	D:\Database\W10N14.L			
			Bicyclo[3.1.0]hexan-2-ol, 2-methyl-5-(1-methylethyl)-, (1.alpha.,2.alpha.,5.alpha.)-	83607	017699-16-0	98
			5-Isopropyl-2-methylbicyclo[3.1.0]hexan-2-ol #	83612	000546-79-2	96
			Bicyclo[3.1.0]hexan-2-ol, 2-methyl	83613	015537-55-0	96

Data Path : D:\msdchem\1\data\
Data File : Karimnezhad 4.D
Acq On : 15 Mar 2022 10:08
Operator : Jafari
Sample : M14
Misc :
ALS Vial : 32 Sample Multiplier: 1

Search Libraries: D:\Database\W10N14.L Minimum Quality: 0

Unknown Spectrum: Apex
Integration Events: ChemStation Integrator - events.e

Pk# RT Area% Library/ID Ref# CAS# Qual

-5-(1-methylethyl)-, (1.alpha.,2.b
eta.,5.alpha.)-

14 20.582 0.19 D:\Database\W10N14.L
Benzene, 1-methyl-4-(1-methylethen 42687 001195-32-0 97
yl)-
Benzene, methyl(1-methylethenyl)- 42656 026444-18-8 95
Benzene, 1-methyl-4-(1-methylethen 42682 001195-32-0 94
yl)-

15 20.873 0.14 D:\Database\W10N14.L
Butanoic acid, 2-methyl-, 3-methyl 126281 027625-35-0 86
butyl ester
Butanoic acid, 2-methyl-, 3-methyl 126288 027625-35-0 86
butyl ester
isopentyl 2-methylbutanoate 126284 027625-35-0 83

16 21.182 0.21 D:\Database\W10N14.L
Bicyclo[3.1.0]hexan-2-ol, 2-methyl 83607 017699-16-0 93
-5-(1-methylethyl)-, (1.alpha.,2.a
lpha.,5.alpha.)-
4-Thujanol, cis-(.+-.)- 83603 015826-82-1 52
p-Menth-8-en-1-ol, stereoisomer 83201 007299-40-3 50

17 21.782 0.13 D:\Database\W10N14.L
3-Octanol, acetate 126339 004864-61-3 91
3-Octanol, acetate 126344 004864-61-3 50
Succinic acid, pentyl 2-propylpent 532628 2000532-62-8 43
yl ester

18 23.079 0.08 D:\Database\W10N14.L
Bicyclo[3.1.0]hexan-3-ol, 4-methyl 78257 000471-16-9 87
ene-1-(1-methylethyl)-, [1S-(1.alp
ha.,3.beta.,5.alpha.)]-
Bicyclo[3.1.0]hexan-3-ol, 4-methyl 78251 003310-02-9 83
ene-1-(1-methylethyl)-, (1.alpha.,
3.alpha.,5.alpha.)-
4(10)-Thujen-3-ol, (1S,3R,5S)-(+)- 78253 000471-16-9 80

19 23.216 0.18 D:\Database\W10N14.L
6,6-DIMETHYL-2-METHYLENEBICYCLO[3. 78355 000547-61-5 93
1.1]HEPTAN-3-OL
Bicyclo[3.1.1]heptan-3-ol, 6,6-dim 78363 000547-61-5 93

Data Path : D:\msdchem\1\data\
Data File : Karimnezhad 4.D
Acq On : 15 Mar 2022 10:08
Operator : Jafari
Sample : M14
Misc :
ALS Vial : 32 Sample Multiplier: 1

Search Libraries: D:\Database\W10N14.L Minimum Quality: 0

Unknown Spectrum: Apex
Integration Events: ChemStation Integrator - events.e

Pk#	RT	Area%	Library/ID	Ref#	CAS#	Qual
			ethyl-2-methylene-, [1S-(1.alpha., 3.alpha.,5.alpha.)]-			
			2(10)-Pinen-3-ol, (1S,3R,5S)-(-)-	78358	000547-61-5	93
20	23.394	0.13	D:\Database\W10N14.L			
			trans-Verbenol	77673	001820-09-3	94
			2-Pinen-4-ol, trans-	77665	001820-09-3	64
			Bicyclo[3.2.0]hept-6-en-2-ol, 1,4, 4-trimethyl-, (1.alpha.,2.alpha.,5 .alpha.)-	77857	014590-83-1	49
21	23.559	0.28	D:\Database\W10N14.L			
			(1S,6R)-3,7,7-Trimethylbicyclo[4.1 .0]hept-3-en-2,5-dione	104284	2000104-28-4	86
			2,3,4,5,6-Pentamethylphenol	104739	2000104-73-9	83
			1(2H)-Naphthalenone, 3,4,5,6,7,8-h exahydro-7-methyl-	104860	059177-21-8	83
22	23.845	0.20	D:\Database\W10N14.L			
			Cyclohexanone, 5-methyl-2-(1-methy lethyl)-, trans-	83410	000089-80-5	98
			2-ISOPROPYL-5-METHYLCYCLOHEXANONE	83413	000089-80-5	98
			Cyclohexanone, 5-methyl-2-(1-methy lethyl)-	83424	010458-14-7	97
23	24.199	0.23	D:\Database\W10N14.L			
			3,6-DIMETHYL-4,5,6,7-TETRAHYDRO-1- BENZOFURAN	72661	000494-90-6	93
			Benzofuran, 4,5,6,7-tetrahydro-3,6 -dimethyl-	72657	000494-90-6	76
			Benzofuran, 4,5,6,7-tetrahydro-3,6 -dimethyl-	72656	000494-90-6	76
24	24.297	0.23	D:\Database\W10N14.L			
			L-MENTHONE	83439	010458-14-7	98
			Cyclohexanone, 5-methyl-2-(1-methy lethyl)-	83423	010458-14-7	98
			Cyclohexanone, 5-methyl-2-(1-methy lethyl)-, cis-	83396	000491-07-6	98
25	24.605	0.30	D:\Database\W10N14.L			
			Cyclohexanemethanol, .alpha.,.alph a.-dimethyl-4-methylene-	83142	007299-42-5	90

Data Path : D:\msdchem\1\data\
Data File : Karimnezhad 4.D
Acq On : 15 Mar 2022 10:08
Operator : Jafari
Sample : M14
Misc :
ALS Vial : 32 Sample Multiplier: 1

Search Libraries: D:\Database\W10N14.L Minimum Quality: 0

Unknown Spectrum: Apex
Integration Events: ChemStation Integrator - events.e

Pk#	RT	Area%	Library/ID	Ref#	CAS#	Qual
			3-Cyclohexene-1-methanol, .alpha., .alpha.,4-trimethyl-	82889	010482-56-1	53
			3-Cyclohexene-1-methanol, .alpha., .alpha.,4-trimethyl-	82891	010482-56-1	53
26	24.782	1.45	D:\Database\W10N14.L			
			endo-Borneol	84059	000507-70-0	94
			1,7,7-TRIMETHYLBICYCLO[2.2.1]HEPTA N-2-OL	84051	000464-45-9	94
			Bicyclo[2.2.1]heptan-2-ol, 1,7,7-t rimethyl-, (1S-endo)-	84056	000464-45-9	90
27	25.085	0.40	D:\Database\W10N14.L			
			3-Cyclohexen-1-ol, 4-methyl-1-(1-m ethylethyl)-	82943	000562-74-3	98
			1-ISOPROPYL-4-METHYL-3-CYCLOHEXEN- 1-OL	82925	000562-74-3	98
			3-Cyclohexen-1-ol, 4-methyl-1-(1-m ethylethyl)-, (R)-	82932	020126-76-5	96
28	25.451	0.54	D:\Database\W10N14.L			
			PARA-CYMEN-8-OL	72711	001197-01-9	93
			Adamantane, 1-methyl-	73870	000768-91-2	83
			3-Methyl-6-hydroxybenzo[c]-dihydro furan	72145	2000072-14-5	83
29	25.805	0.52	D:\Database\W10N14.L			
			.alpha.-Terpineol	82900	000098-55-5	90
			Cyclohexene, 5-methyl-3-(1-methyle thenyl)-, trans-(-)-	48797	056816-08-1	60
			Cyclohexene, 5-methyl-3-(1-methyle thenyl)-, trans-(-)-	48798	056816-08-1	60
30	26.371	0.10	D:\Database\W10N14.L			
			7a-Methyl-1,2,5,6,7,7a-hexahydroin den-4-one	72739	2000072-73-9	70
			2-Adamantanone	73369	000700-58-3	58
			(3E,5Z)-1,3,5-Undecatriene	73975	051447-08-6	58
31	26.840	0.46	D:\Database\W10N14.L			
			2-Hydroxy-2-isopropyl-5-methylcycl ohexanone	115823	000000-00-0	58
			Cyclohexanol, 2-methylene-, (S)-	19554	061187-70-0	43

Data Path : D:\msdchem\1\data\
Data File : Karimnezhad 4.D
Acq On : 15 Mar 2022 10:08
Operator : Jafari
Sample : M14
Misc :
ALS Vial : 32 Sample Multiplier: 1

Search Libraries: D:\Database\W10N14.L Minimum Quality: 0

Unknown Spectrum: Apex
Integration Events: ChemStation Integrator - events.e

Pk#	RT	Area%	Library/ID	Ref#	CAS#	Qual
			1,3-Cyclohexanedione, 5-isopropyl-	82473	018456-87-6	41
32	27.160	2.86	D:\Database\W10N14.L			
			8,9-Dehydrothymol	68975	018612-99-2	95
			1-methoxy-4-(1-methylethenyl)benzene	68640	2000068-64-0	81
			1-Isopropenyl-4-methoxy-benzene	68642	2000068-64-2	72
33	27.989	9.03	D:\Database\W10N14.L			
			(R)-5-methyl-2-(1-methylethylidene)-cyclohexanone	78102	000089-82-7	97
			Pulegone	78101	000089-82-7	97
			Cyclohexanone, 5-methyl-2-(1-methylethylidene)-	78096	015932-80-6	96
34	28.406	0.62	D:\Database\W10N14.L			
			7-Oxabicyclo[4.1.0]heptan-2-one, 6-methyl-3-(1-methylethylidene)-	114758	035178-55-3	97
			4-ISOPROPENYL-1-METHYL-7-OXABICYCLO[4.1.0]HEPTAN-2-ONE	114757	035178-55-3	49
			3-Hexene, 2,2,5,5-tetramethyl-, (Z)-	55805	000692-47-7	46
35	28.566	1.38	D:\Database\W10N14.L			
			4-ISOPROPENYL-1-METHYL-7-OXABICYCLO[4.1.0]HEPTAN-2-ONE	114757	035178-55-3	83
			7-Oxabicyclo[4.1.0]heptan-2-one, 6-methyl-3-(1-methylethyl)-	115503	005286-38-4	81
			7-Oxabicyclo[4.1.0]heptan-2-one, 6-methyl-3-(1-methylethylidene)-	114758	035178-55-3	80
36	28.766	0.22	D:\Database\W10N14.L			
			Naphthalene, decahydro-	52330	000091-17-8	52
			NAPHTHALENE, DECAHYDRO-	52324	000091-17-8	52
			1,1'-Bicyclopentyl	52365	001636-39-1	50
37	28.875	0.11	D:\Database\W10N14.L			
			2-Cyclopenten-1-one, 3-methyl-	8660	002758-18-1	50
			1,6-Octadiene, 3,7-dimethyl-, (S)-	52472	010281-55-7	43
			1-BUTYL-2-ETHYL-1-CYCLOPROPENE	31910	050915-91-8	38
38	29.029	0.17	D:\Database\W10N14.L			
			2,4-Dimethoxyphenol	81451	013330-65-9	86

Data Path : D:\msdchem\1\data\
Data File : Karimnezhad 4.D
Acq On : 15 Mar 2022 10:08
Operator : Jafari
Sample : M14
Misc :
ALS Vial : 32 Sample Multiplier: 1

Search Libraries: D:\Database\W10N14.L Minimum Quality: 0

Unknown Spectrum: Apex
Integration Events: ChemStation Integrator - events.e

Pk# RT Area% Library/ID Ref# CAS# Qual

4-Fluoro-2-acetylphenol 81032 000394-32-1 86
1-ethyl-3-methyl-2-propylidenimida 84359 109153-29-9 83
zolidine

39 29.286 0.92 D:\Database\W10N14.L
(S)-(+)-cis-Isopiperitenone 72898 2000072-89-8 91
1,8-(p-MENTHADIENONE) 72899 2000072-89-9 72
2-Cyclohexen-1-one, 3,5-dimethyl- 31340 001123-09-7 50

40 29.680 0.27 D:\Database\W10N14.L
4-Methyl-1-(acetoxy)benzene 72241 000140-39-6 90
4-(Methoxymethyl)benzenaldehyde 72078 2000072-07-8 90
4-Hydroxy-3-methylacetophenone 72072 000876-02-8 87

41 29.892 0.64 D:\Database\W10N14.L
Bicyclo[2.2.1]heptan-2-ol, 1,7,7-t 192108 005655-61-8 99
rimethyl-, acetate, (1S-endo)-
Bicyclo[2.2.1]heptan-2-ol, 1,7,7-t 192123 005655-61-8 99
rimethyl-, acetate, (1S-endo)-
2-Norbornanol, 1,3,3-trimethyl-, a 192153 004057-31-2 98
cetate, endo-

42 30.246 0.29 D:\Database\W10N14.L
Benzene, 1-ethoxy-4-ethyl- 73585 001585-06-4 89
Benzene, 1-ethoxy-4-ethyl- (CAS) 73584 001585-06-4 89
2,4-Cycloheptadien-1-one, 2,6,6-tr 73012 000503-93-5 86
imethyl-

43 30.984 0.37 D:\Database\W10N14.L
Phenol, 5-methyl-2-(1-methylethyl) 73197 000089-83-8 95
Phenol, 5-methyl-2-(1-methylethyl) 73200 000089-83-8 95
Thymol 73196 000089-83-8 95

44 31.441 0.47 D:\Database\W10N14.L
Carvacrol 72968 000499-75-2 94
Carvacrol 72969 000499-75-2 94
Carvacrol 72963 000499-75-2 91

45 31.607 0.27 D:\Database\W10N14.L
2,6-Dimethyl-2,6-octadiene-1,8-dio 380078 036052-53-6 38
l diacetate
2-Oxabicyclo[3.3.1]non-3-ene, 4,8- 78948 049576-27-4 38
dimethyl-, endo-

Data Path : D:\msdchem\1\data\
Data File : Karimnezhad 4.D
Acq On : 15 Mar 2022 10:08
Operator : Jafari
Sample : M14
Misc :
ALS Vial : 32 Sample Multiplier: 1

Search Libraries: D:\Database\W10N14.L Minimum Quality: 0

Unknown Spectrum: Apex
Integration Events: ChemStation Integrator - events.e

Pk#	RT	Area%	Library/ID	Ref#	CAS#	Qual
			2-propyl cyclopentanone	33966	2000033-96-6	35
46	33.001	31.13	D:\Database\W10N14.L			
			2-Cyclohexen-1-one, 3-methyl-6-(1-methylethylidene)-	73417	000491-09-8	98
			2-Cyclohexen-1-one, 3-methyl-6-(1-methylethylidene)-	73419	000491-09-8	95
			2-Cyclohexen-1-one, 3-methyl-6-(1-methylethylidene)-	73416	000491-09-8	91
47	33.904	19.12	D:\Database\W10N14.L			
			PIPERITENONE OXIDE	110057	003564-96-3	99
			2-[3'-Hydroxypropyl]-1,4-benzoquinone	108313	2000108-31-3	86
			2-Ethyl-3-methoxypyrazine	51317	025680-58-4	58
48	34.253	0.33	D:\Database\W10N14.L			
			2',6'-Dihydroxy-3'-methylacetophenone	108340	029183-78-6	87
			5,6-Dimethyl-3,4,5,6-tetrahydro-2H-cyclopenta[b]pyran-7-one	110049	2000110-04-9	83
			3-tert-Butylcatechol	109768	2000109-76-8	83
49	34.584	0.45	D:\Database\W10N14.L			
			.BETA. BOURBONENE	215702	005208-59-3	99
			(-)-.beta.-Bourbonene	215705	005208-59-3	98
			(-)-.beta.-Bourbonene	215706	005208-59-3	96
50	34.721	0.15	D:\Database\W10N14.L			
			2,4-DIISOPROPENYL-1-METHYL-1-VINYL CYCLOHEXANE	216297	000515-13-9	97
			Cyclohexane, 1-ethenyl-1-methyl-2,4-bis(1-methylethenyl)-, [1S-(1.alpha.,2.beta.,4.beta.)]-	216301	000515-13-9	93
			.beta.-Elemene	216300	000515-13-9	93
51	35.144	1.55	D:\Database\W10N14.L			
			4,6-DIETHYL-2-METHOXYPYRIMIDINE	110182	2000110-18-2	64
			3-Hydroxy-4-methoxy-5-methylbenzaldehyde	108182	2000108-18-2	64
			2-Hydroxy-7-methoxy-4-methylcyclohepta-2,4,6-trien-1-one	108662	2000108-66-2	59

Data Path : D:\msdchem\1\data\
Data File : Karimnezhad 4.D
Acq On : 15 Mar 2022 10:08
Operator : Jafari
Sample : M14
Misc :
ALS Vial : 32 Sample Multiplier: 1

Search Libraries: D:\Database\W10N14.L Minimum Quality: 0

Unknown Spectrum: Apex
Integration Events: ChemStation Integrator - events.e

Pk#	RT	Area%	Library/ID	Ref#	CAS#	Qual
52	36.202	3.81	D:\Database\W10N14.L			
			Caryophyllene	216361	000087-44-5	99
			TRANS(.BETA.)-CARYOPHYLLENE	216339	2000216-33-9	99
			Bicyclo[7.2.0]undec-4-ene, 4,11,11 -trimethyl-8-methylene-, (E)-(1R,9 S)-(-)-	216351	000087-44-5	99
53	36.527	0.08	D:\Database\W10N14.L			
			(1R,2S,6S,7S,8S)-8-Isopropyl-1-met hyl-3-methylenetricyclo[4.4.0.02,7]decane-rel-	216027	018252-44-3	98
			1H-Cyclopenta[1,3]cyclopropa[1,2]b enzene, 2,3,3a.alpha.,3b.alpha.,4, 5,6,7-octahydro-4.alpha.-isopropyl -7.beta.-methyl-3-methylene-	216768	013744-15-5	98
			8-ISOPROPYL-1-METHYL-5-METHYLENE-1 ,6-CYCLODECADIENE	216746	023986-74-5	97
54	36.962	0.14	D:\Database\W10N14.L			
			5,9-Undecadien-2-one, 6,10-dimethy l-, (E)-	186308	003796-70-1	93
			5,9-Undecadien-2-one, 6,10-dimethy l-, (E)-	186310	003796-70-1	86
			5,9-Undecadien-2-one, 6,10-dimethy l-	186306	000689-67-8	86
55	37.173	0.65	D:\Database\W10N14.L			
			(E)-.beta.-Famesene	216558	018794-84-8	96
			trans-.beta.-Farnesene	216566	000502-60-3	96
			(1S,5S,6R)-6-Methyl-2-methylene-6- (4-methylpent-3-en-1-yl)bicyclo[3. 1.1]heptane	216863	015438-94-5	95
56	37.648	0.50	D:\Database\W10N14.L			
			.alpha.-Humulene	216788	006753-98-6	99
			1,4,7,-Cycloundecatriene, 1,5,9,9- tetramethyl-, Z,Z,Z-	216754	2000216-75-4	98
			.alpha.-Humulene	216803	006753-98-6	98
57	38.048	0.41	D:\Database\W10N14.L			
			2-Cyclohexen-1-one, 3-methyl-6-(1- methylethylidene)-	73417	000491-09-8	55
			2-Pinen-4-one	73532	000080-57-9	55

Data Path : D:\msdchem\1\data\
Data File : Karimnezhad 4.D
Acq On : 15 Mar 2022 10:08
Operator : Jafari
Sample : M14
Misc :
ALS Vial : 32 Sample Multiplier: 1

Search Libraries: D:\Database\W10N14.L Minimum Quality: 0

Unknown Spectrum: Apex
Integration Events: ChemStation Integrator - events.e

Pk#	RT	Area%	Library/ID	Ref#	CAS#	Qual
			(+-)-m-Menthadien-6-one	73578	2000073-57-8	55
58	38.419	0.19	D:\Database\W10N14.L			
			3-Buten-2-one, 4-(2,6,6-trimethyl-1-cyclohexen-1-yl)-	179332	014901-07-6	97
			trans-.beta.-Ionone	179340	000079-77-6	97
			(3E)-4-(2,6,6-TRIMETHYL-1-CYCLOHEXEN-1-YL)-3-BUTEN-2-ONE	179346	000079-77-6	96
59	38.699	1.32	D:\Database\W10N14.L			
			8-ISOPROPYL-1-METHYL-5-METHYLENE-1,6-CYCLODECADIENE	216746	023986-74-5	98
			1H-Cyclopenta[1,3]cyclopropa[1,2]benzene, 2,3,3a.alpha.,3b.alpha.,4,5,6,7-octahydro-4.alpha.-isopropyl-7.beta.-methyl-3-methylene-	216768	013744-15-5	98
			1,6-Cyclodecadiene, 1-methyl-5-methylene-8-(1-methylethyl)-, [s-(E,E)]-	216750	023986-74-5	98
60	39.122	0.16	D:\Database\W10N14.L			
			(1S,6R)-3,7,7-Trimethylbicyclo[4.1.0]hept-3-en-2,5-dione	104284	2000104-28-4	90
			1-(4-Ethyl-2-hydroxyphenyl)ethanon	103396	2000103-39-6	83
			3-tert-Butylanisole	105065	2000105-06-5	83
61	39.271	0.44	D:\Database\W10N14.L			
			Bicyclo[8.1.0]undeca-2,6-diene, 3,7,11,11-tetramethyl-, (1R*,2Z,6E,10R*)-(.+-.)-	215949	100762-46-7	99
			Bicyclo[8.1.0]undeca-2,6-diene, 3,7,11,11-tetramethyl-, (1R*,2Z,6E,10R*)-(.+-.)-	215950	100762-46-7	98
			Bicyclogermacrene	215957	067650-90-2	91
62	39.665	0.17	D:\Database\W10N14.L			
			1-(3-Methyl-cyclopent-2-enyl)-cyclohexene	100819	2000100-81-9	94
			p-Methylcinnamic acid	98953	001866-39-3	64
			1-phenylpyrazolidin-3-one	99453	000092-43-3	59
63	39.945	0.08	D:\Database\W10N14.L			
			Naphthalene, 1,2,3,4,4a,5,6,8a-oct	215867	039029-41-9	98

Data Path : D:\msdchem\1\data\
Data File : Karimnezhad 4.D
Acq On : 15 Mar 2022 10:08
Operator : Jafari
Sample : M14
Misc :
ALS Vial : 32 Sample Multiplier: 1

Search Libraries: D:\Database\W10N14.L Minimum Quality: 0

Unknown Spectrum: Apex
Integration Events: ChemStation Integrator - events.e

Pk#	RT	Area%	Library/ID	Ref#	CAS#	Qual
			ahydro-7-methyl-4-methylene-1-(1-m ethylethyl)-, (1.alpha.,4a.beta.,8 a.alpha.)-			
			.gamma.-Cadinene	215866	039029-41-9	98
			Naphthalene, 1,2,3,4,4a,5,6,8a-oct ahydro-7-methyl-4-methylene-1-(1-m ethylethyl)-, (1.alpha.,4a.beta.,8 a.alpha.)-	215864	039029-41-9	96
64	40.077	0.10	D:\Database\W10N14.L			
			.delta.-Cadinene	216928	000483-76-1	99
			.delta.-Cadinene	216931	000483-76-1	99
			.delta.-Cadinene	216937	000483-76-1	98
65	41.677	0.18	D:\Database\W10N14.L			
			caryophylla-3,8(13)-dien-5.beta.-o	267103	2000267-10-3	49
			1,6,10-Dodecatrien-3-ol, 3,7,11-tr imethyl-	275390	007212-44-4	42
			Nerolidol	275373	000142-50-7	38
66	42.140	0.99	D:\Database\W10N14.L			
			Cyclohexanecarboxylic acid, 1-meth yl-2-oxo-, ethyl ester	156877	005453-94-1	83
			3-Chloro-4-t-butyl-6-methylpyridaz ine	155956	2000155-95-6	83
			5(6)-(Chloromethyl)benzo[1,2-c]-1, 2,5-oxadiazole N1-Oxide	154801	2000154-80-1	59
67	42.671	0.91	D:\Database\W10N14.L			
			1H-Cycloprop[e]azulen-7-ol, decahy dro-1,1,7-trimethyl-4-methylene-, [1ar-(1a.alpha.,4a.alpha.,7.beta., 7a.beta.,7b.alpha.)]-	267797	006750-60-3	98
			1,1,7-TRIMETHYL-4-METHYLENEDECAHYD RO-1H-CYCLOPROPA[E]AZULEN-7-OL	267795	077171-55-2	98
			(-)-Spathulenol	267796	077171-55-2	95
68	42.917	3.40	D:\Database\W10N14.L			
			(-)-5-Oxatricyclo[8.2.0.0(4,6)]dod ecane,,12-trimethyl-9-methylene-, [1R-(1R*,4R*,6R*,10S*)]-	267388	001139-30-6	99
			(-)-5-Oxatricyclo[8.2.0.0(4,6)]dod ecane,,12-trimethyl-9-methylene-,	267387	001139-30-6	95

Data Path : D:\msdchem\1\data\
Data File : Karimnezhad 4.D
Acq On : 15 Mar 2022 10:08
Operator : Jafari
Sample : M14
Misc :
ALS Vial : 32 Sample Multiplier: 1

Search Libraries: D:\Database\W10N14.L Minimum Quality: 0

Unknown Spectrum: Apex
Integration Events: ChemStation Integrator - events.e

Pk#	RT	Area%	Library/ID	Ref#	CAS#	Qual
			[1R-(1R*,4R*,6R*,10S*)]-Caryophyllene oxide	267393	001139-30-6	91
69	43.923	0.28	D:\Database\W10N14.L			
			(1R,3E,7E,11R)-1,5,5,8-Tetramethyl-12-oxabicyclo[9.1.0]dodeca-3,7-diene	267258	019888-34-7	99
			Naphthalene, decahydro-, cis-	52317	000493-01-6	78
			Naphthalene, decahydro-	52331	000091-17-8	53
70	44.723	0.23	D:\Database\W10N14.L			
			isospathulenol	267807	2000267-80-7	99
			(-)-Spathulenol	267798	077171-55-2	95
			4,8,11,11-Tetramethyl-8-tricyclo[7.2.0.0(2,5).]undecen-4-ol	267697	2000267-69-7	50
71	44.957	0.34	D:\Database\W10N14.L			
			10,10-Dimethyl-2,6-dimethylenebicyclo[7.2.0]undecan-5.beta.-ol	267895	019431-80-2	98
			caryophylla-4(12),8(13)-dien-5.beta.-ol	267136	2000267-13-6	94
			10,10-Dimethyl-2,6-dimethylenebicyclo[7.2.0]undecan-5.beta.-ol	267897	019431-80-2	94
72	45.203	0.09	D:\Database\W10N14.L			
			3-Buten-2-one, 4-(2,6,6-trimethyl-1-cyclohexen-1-yl)-	179332	014901-07-6	52
			trans-.beta.-Ionone	179340	000079-77-6	52
			trans-.beta.-Ionone	179342	000079-77-6	47
73	45.603	0.22	D:\Database\W10N14.L			
			caryophylla-3,8(13)-dien-5.beta.-ol	267103	2000267-10-3	92
			Presilphiperfolane-9,15-epoxide	267660	2000267-66-0	91
			2H-Cyclopenta[a]pentalen-2-one, decahydro-3,4,4,6a-tetramethyl-, [3aR-(3a.alpha.,3b.beta.,6a.beta.,7a.alpha.)]-	267323	061228-70-4	56
74	46.163	0.36	D:\Database\W10N14.L			
			Bicyclo[7.2.0]undec-3-en-5-ol, 4,11,11-trimethyl-8-methylene-, stereoisomer	267841	032214-89-4	86
			(-)-5-Oxatricyclo[8.2.0.0(4,6)]dod	267389	001139-30-6	83

Data Path : D:\msdchem\1\data\
Data File : Karimnezhad 4.D
Acq On : 15 Mar 2022 10:08
Operator : Jafari
Sample : M14
Misc :
ALS Vial : 32 Sample Multiplier: 1

Search Libraries: D:\Database\W10N14.L Minimum Quality: 0

Unknown Spectrum: Apex
Integration Events: ChemStation Integrator - events.e

Pk#	RT	Area%	Library/ID	Ref#	CAS#	Qual
			ecane,,12-trimethyl-9-methylene-, [1R-(1R*,4R*,6R*,10S*)]-			
			2H-Cyclopenta[a]pentalen-2-one, decahydro-3,4,4,6a-tetramethyl-, [3aR-(3a.alpha.,3b.beta.,6a.beta.,7a.alpha.)]-	267323	061228-70-4	64
75	46.935	0.18	D:\Database\W10N14.L			
			Pyrazino[2',3':4,5]thieno[3,2-d]pyrimidine-4(1H)-thione	263301	056881-25-5	83
			(E)-3-(4-Aminophenyl)-3-phenyl-2-propenenitrile	265059	2000265-05-9	83
			12-Norcyercene-B	265281	2000265-28-1	83
76	51.930	0.34	D:\Database\W10N14.L			
			2-Pentadecanone, 6,10,14-trimethyl	427982	000502-69-2	99
			2-Pentadecanone, 6,10,14-trimethyl	427985	000502-69-2	95
			2-Pentadecanone, 6,10,14-trimethyl	427986	000502-69-2	95
77	55.937	0.10	D:\Database\W10N14.L			
			n-Hexadecanoic acid	387914	000057-10-3	99
			n-Hexadecanoic acid	387919	000057-10-3	99
			n-Hexadecanoic acid	387916	000057-10-3	97
78	57.485	0.32	D:\Database\W10N14.L			
			1H-Naphtho[2,1-b]pyran, 3-ethenyldodecahydro-3,4a,7,7,10a-pentamethyl-, [3R-(3.alpha.,4a.beta.,6a.alpha.,10a.beta.,10b.alpha.)]-	500325	000596-84-9	99
			1H-Naphtho[2,1-b]pyran, 3-ethenyldodecahydro-3,4a,7,7,10a-pentamethyl-, [3R-(3.alpha.,4a.beta.,6a.alpha.,10a.beta.,10b.alpha.)]-	500323	000596-84-9	91
			1H-Naphtho[2,1-b]pyran, 3-ethenyldodecahydro-3,4a,7,7,10a-pentamethyl-, [3R-(3.alpha.,4a.beta.,6a.alpha.,10a.beta.,10b.alpha.)]-	500327	000596-84-9	91
