# Supplementary material for: Comparison of quantity, quality and antibacterial activity of essential oil Mentha longifolia (L.) L. under different traditional and modern extraction methods
Source: PLoS One. 2024 Jul 10;19(7):e0301558. doi: 10.1371/journal.pone.0301558 (PMC11236116; doi:10.1371/journal.pone.0301558)
Supplement: S2 File — (ZIP) [file pone.0301558.s002.zip › Karimnezhad/M14/QualKarimnezhad 4.pdf]

Library Search Report

Data Path : D:\msdchem\1\data\
Data File : Karimnezhad 4.D
Acq On : 15 Mar 2022 10:08
Operator : Jafari
Sample : M14
Misc :
ALS Vial : 32 Sample Multiplier: 1

Search Libraries: D:\Database\W10N14.L Minimum Quality: 0

Unknown Spectrum: Apex
Integration Events: ChemStation Integrator - events.e

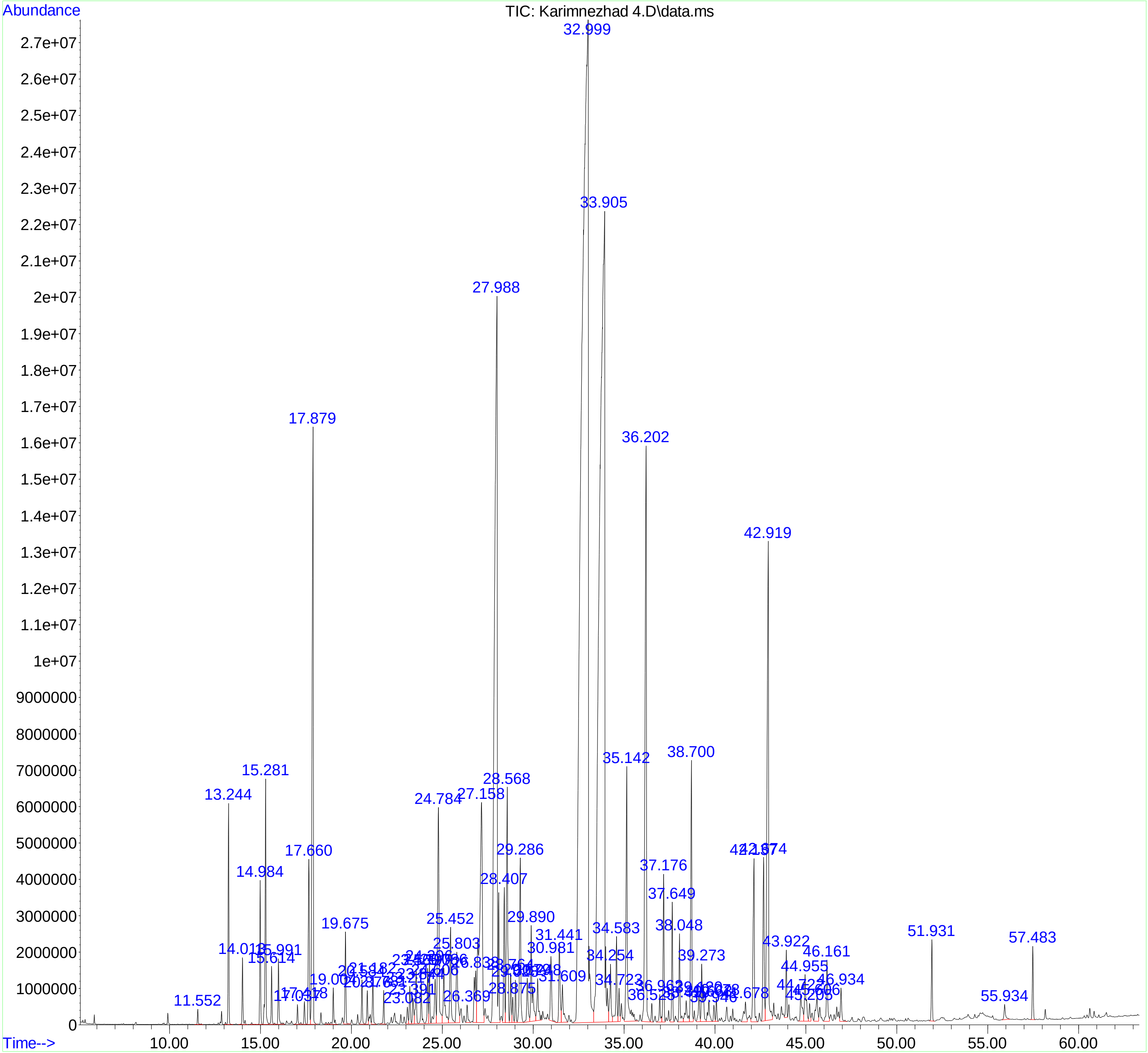

Unknown Spectrum based on Apex

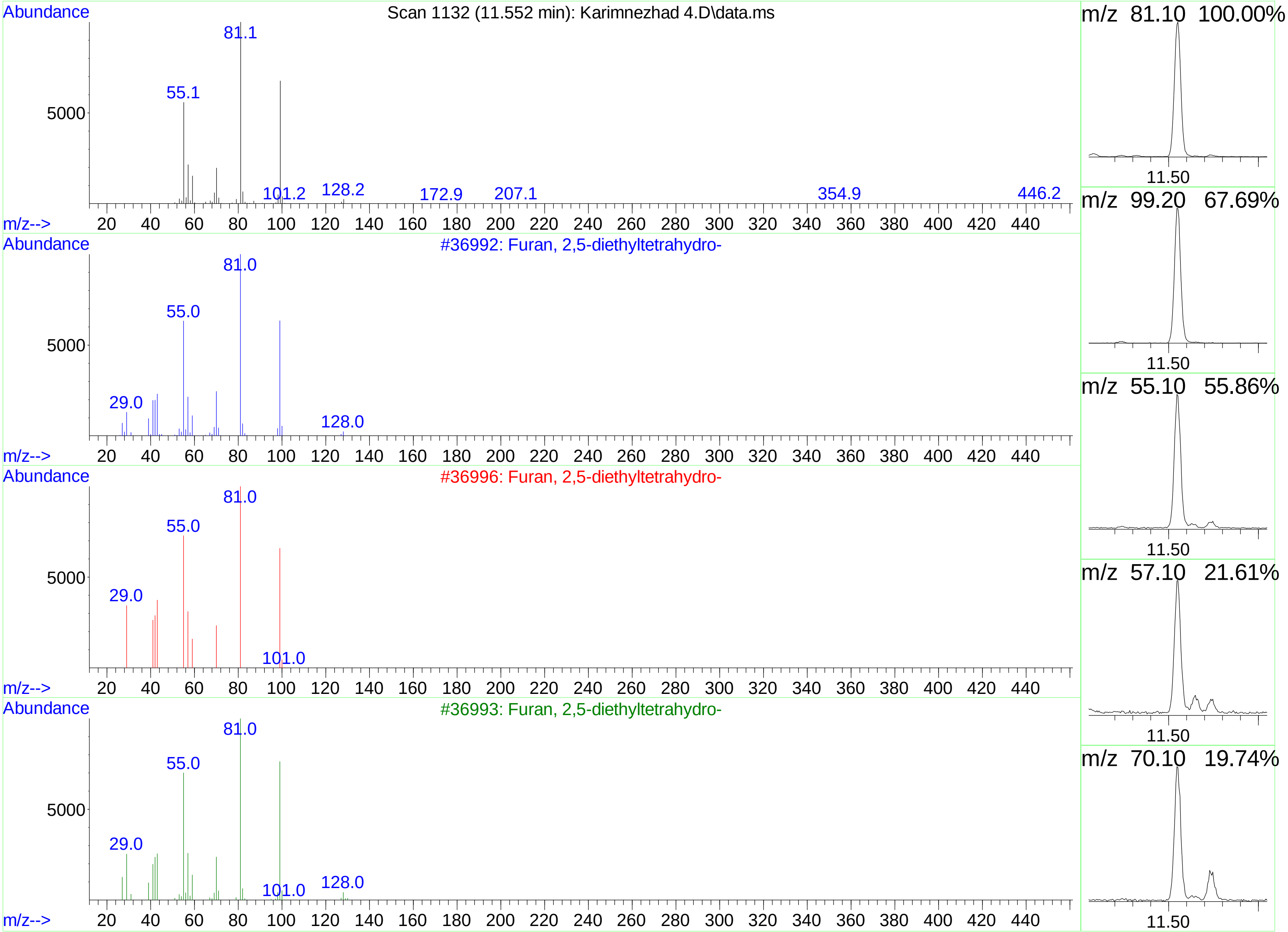

Data File: D:\msdchem\1\data\Karimnezhad 4.D
Sample : M14

Peak Number: 1 at 11.552 min Area: 12189020 Area % 0.05

The 3 best hits from each library.	Ref#	CAS#	Qual
D:\Database\W10N14.L			
1 Furan, 2,5-diethyltetrahydro-	36992	041239-48-9	95
2 Furan, 2,5-diethyltetrahydro-	36996	041239-48-9	64
3 Furan, 2,5-diethyltetrahydro-	36993	041239-48-9	60

Library Search Report - ChemStation Integrator

Unknown Spectrum based on Apex

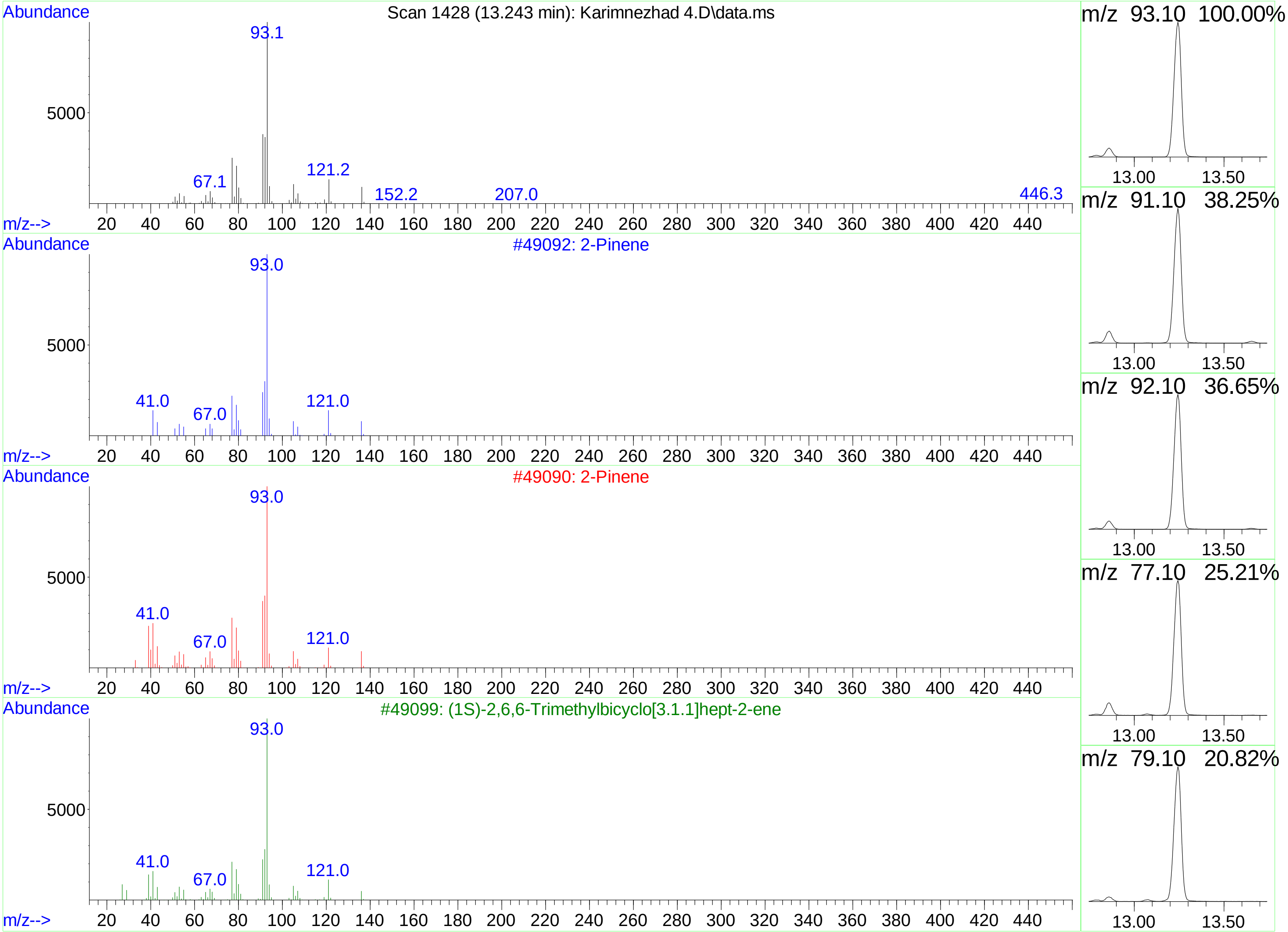

Data File: D:\msdchem\1\data\Karimnezhad 4.D
Sample : M14

Peak Number: 2 at 13.243 min Area: 174433867 Area % 0.76

The 3 best hits from each library.	Ref#	CAS#	Qual
D:\Database\W10N14.L			
1 2-Pinene	49092	000080-56-8	97
2 2-Pinene	49090	000080-56-8	96
3 (1S)-2,6,6-Trimethylbicyclo[3.1....	49099	007785-26-4	96

Unknown Spectrum based on Apex

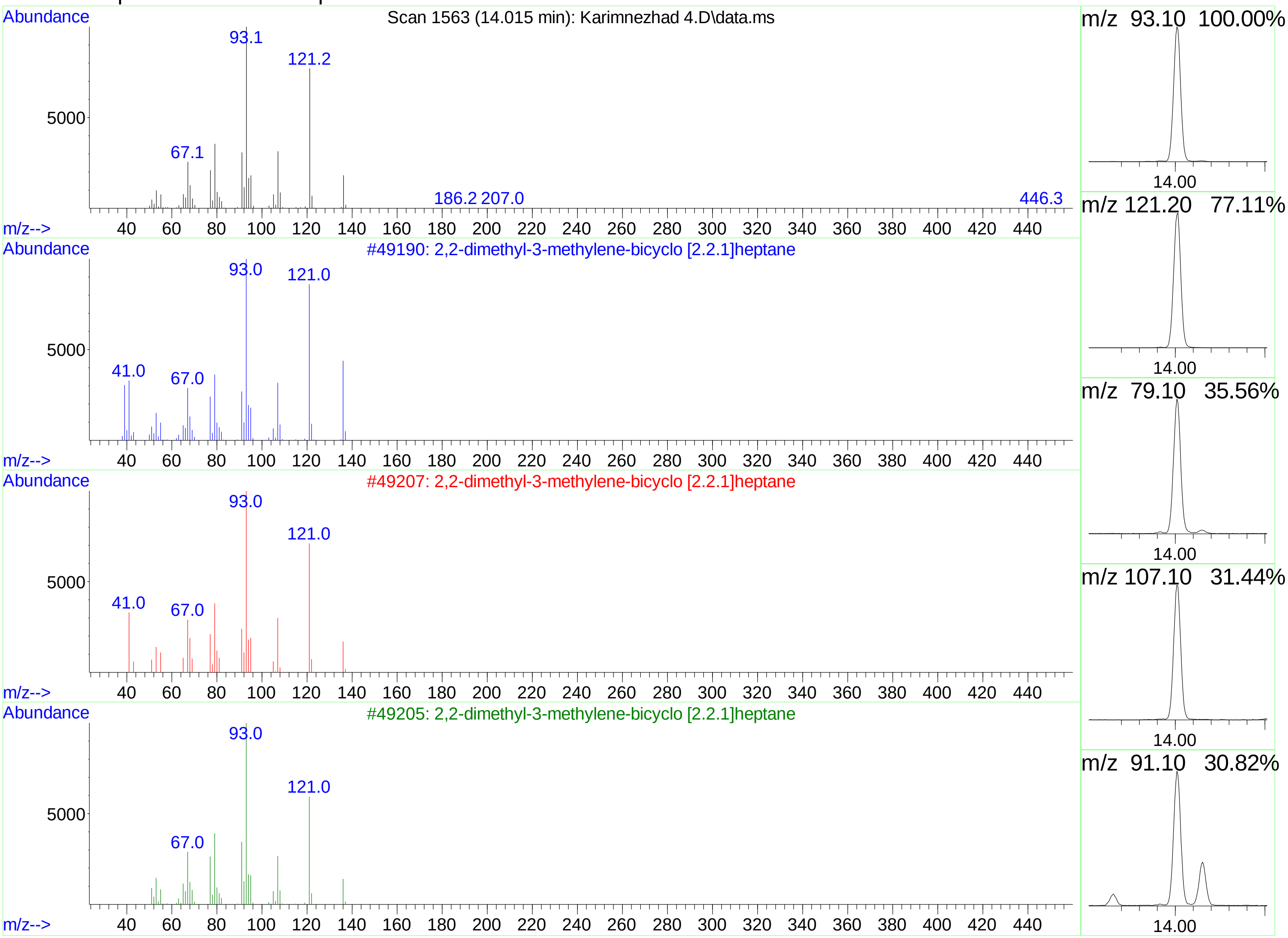

Data File: D:\msdchem\1\data\Karimnezhad 4.D
Sample : M14

Peak Number: 3 at 14.015 min Area: 56290229 Area % 0.25

The 3 best hits from each library.	Ref#	CAS#	Qual
D:\Database\W10N14.L			
1 2,2-dimethyl-3-methylene-bicyclo...	49190	000079-92-5	97
2 2,2-dimethyl-3-methylene-bicyclo...	49207	000079-92-5	97
3 2,2-dimethyl-3-methylene-bicyclo...	49205	000079-92-5	96

Library Search Report - ChemStation Integrator

Unknown Spectrum based on Apex

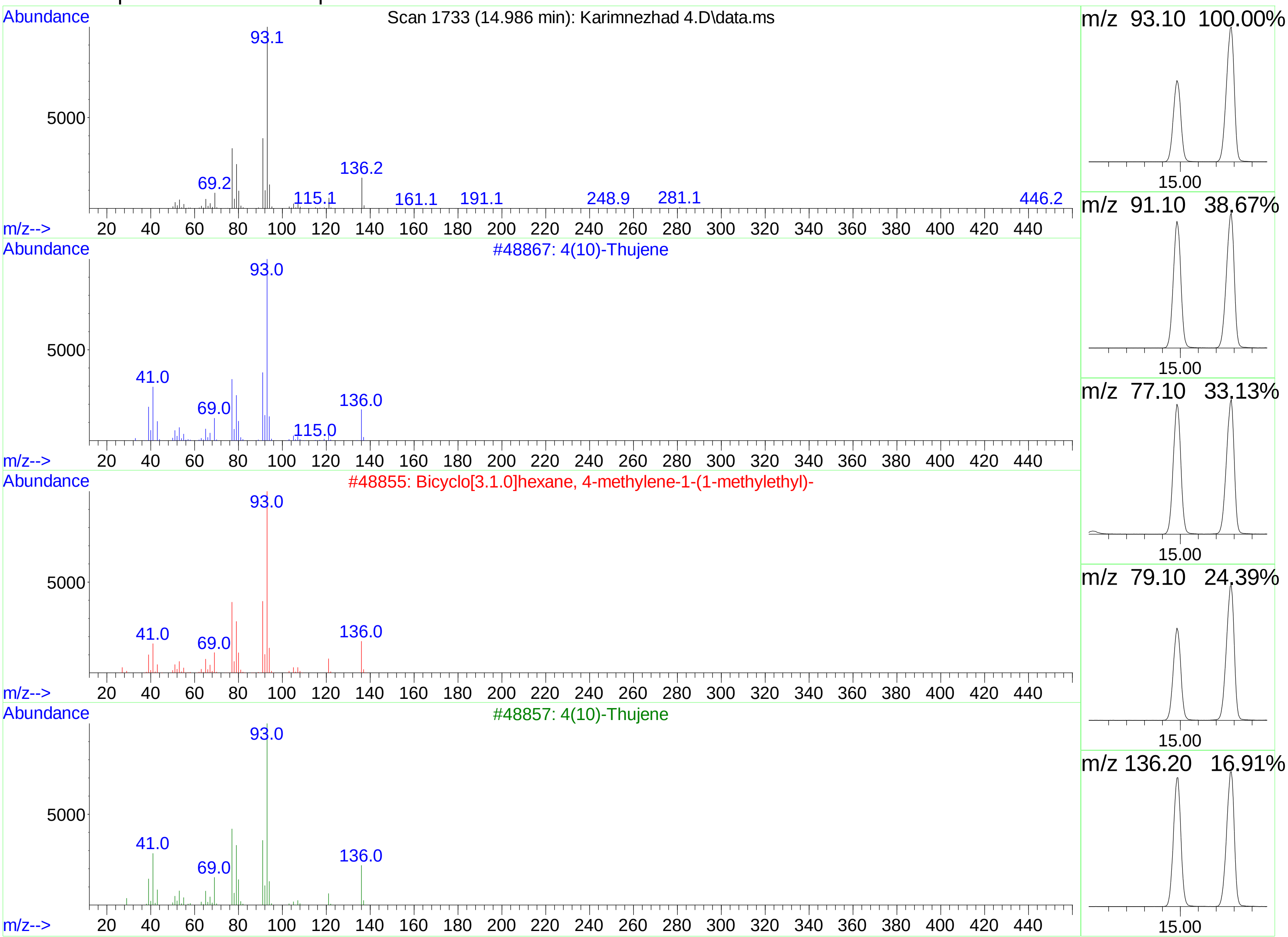

Data File: D:\msdchem\1\data\Karimnezhad 4.D
Sample : M14

Peak Number: 4 at 14.986 min Area: 121173079 Area % 0.53

The 3 best hits from each library.	Ref#	CAS#	Qual
D:\Database\W10N14.L			
1 4(10)-Thujene	48867	003387-41-5	97
2 Bicyclo[3.1.0]hexane, 4-methylen...	48855	003387-41-5	96
3 4(10)-Thujene	48857	003387-41-5	96

Unknown Spectrum based on Apex

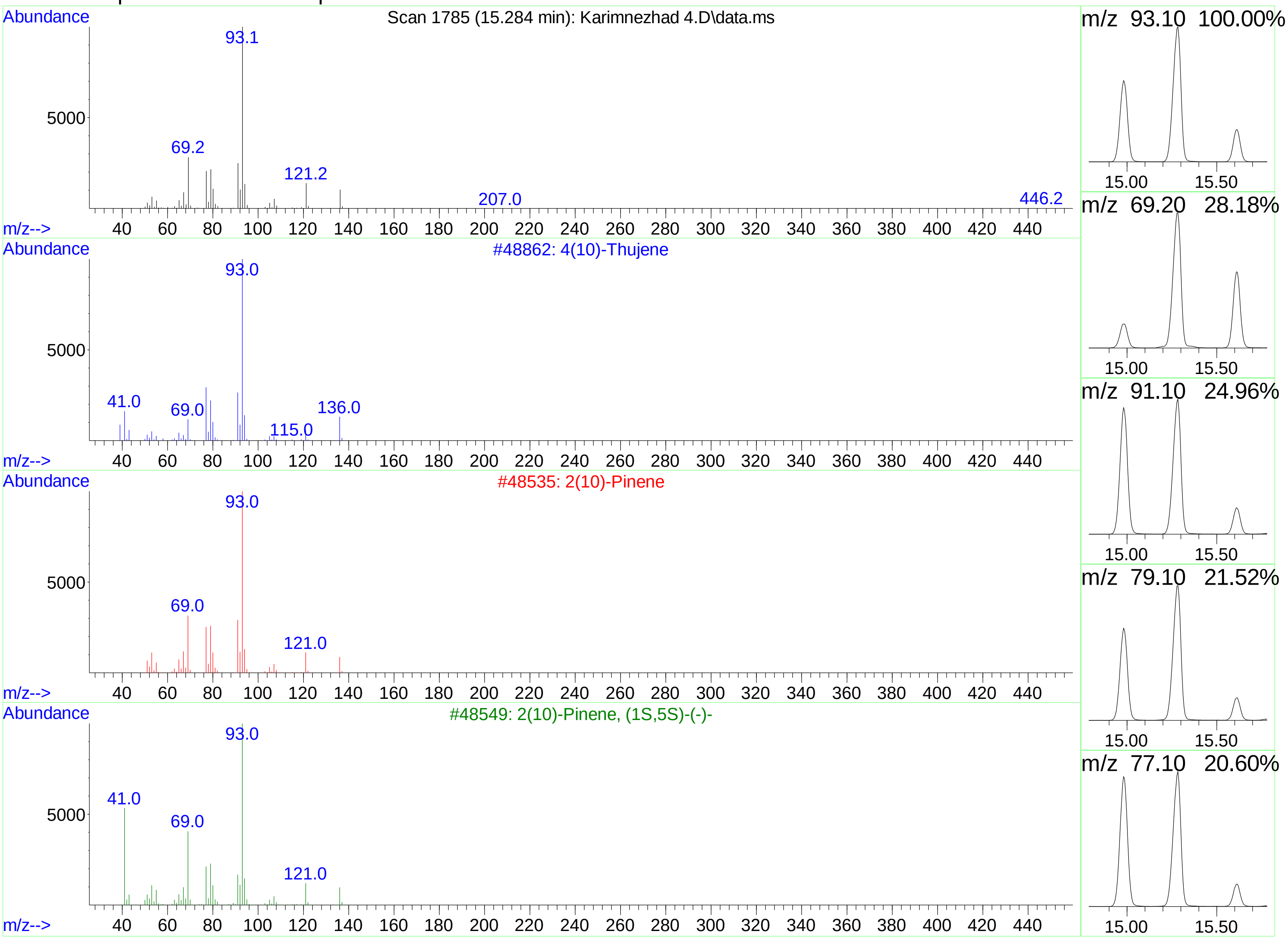

Data File: D:\msdchem\1\data\Karimnezhad 4.D
Sample : M14

Peak Number: 5 at 15.284 min Area: 228758178 Area % 1.00

The 3 best hits from each library. Ref\# CAS\# Qual

D:\Database\W10N14.L

	Name	Ref#	CAS#	Qual
1	4(10)-Thujene	48862	003387-41-5	94
2	2(10)-Pinene	48535	000127-91-3	94
3	2(10)-Pinene, (1S,5S)-(-)-	48549	018172-67-3	94

Unknown Spectrum based on Apex

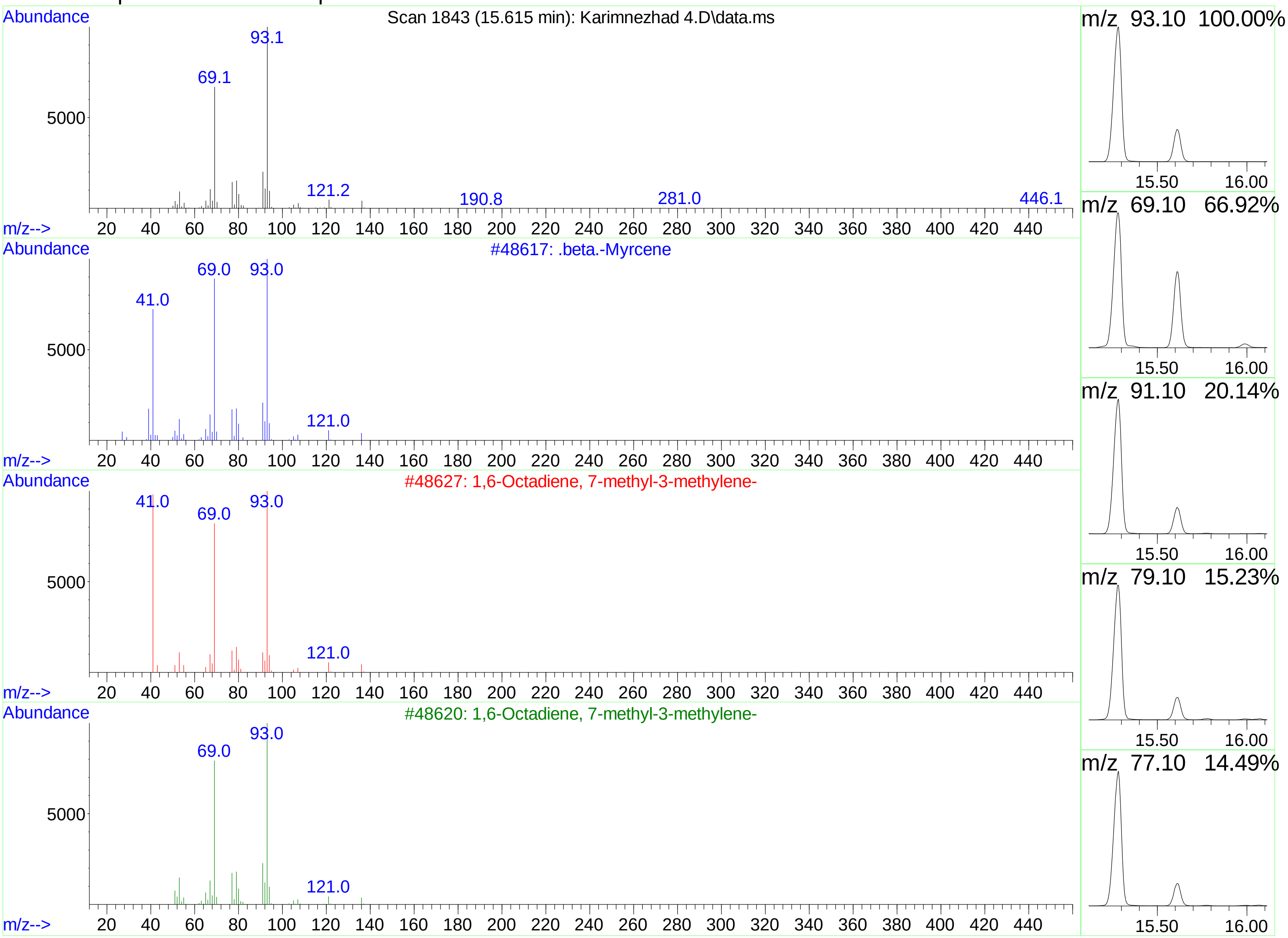

Data File: D:\msdchem\1\data\Karimnezhad 4.D
Sample : M14

Peak Number: 6 at 15.615 min Area: 49399559 Area % 0.22

The 3 best hits from each library.	Ref#	CAS#	Qual
D:\Database\W10N14.L			
1 .beta.-Myrcene	48617	000123-35-3	96
2 1,6-Octadiene, 7-methyl-3-methyl...	48627	000123-35-3	95
3 1,6-Octadiene, 7-methyl-3-methyl...	48620	000123-35-3	94

Unknown Spectrum based on Apex

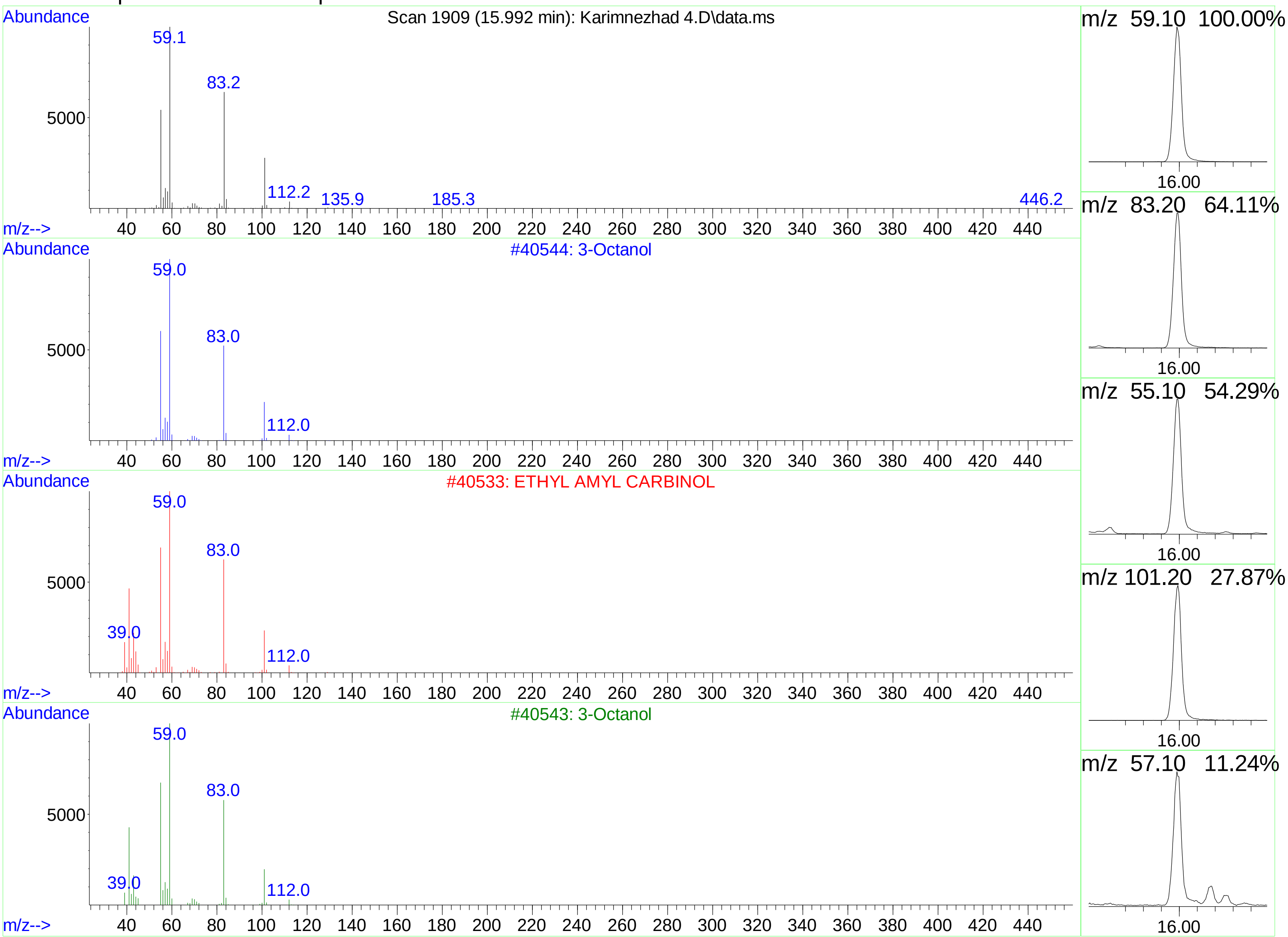

Data File: D:\msdchem\1\data\Karimnezhad 4.D
Sample : M14

Peak Number: 7 at 15.992 min Area: 60864537 Area % 0.27

The 3 best hits from each library.	Ref#	CAS#	Qual
D:\Database\W10N14.L			
1 3-Octanol	40544	000589-98-0	90
2 ETHYL AMYL CARBINOL	40533	000589-98-0	83
3 3-Octanol	40543	000589-98-0	83

Unknown Spectrum based on Apex

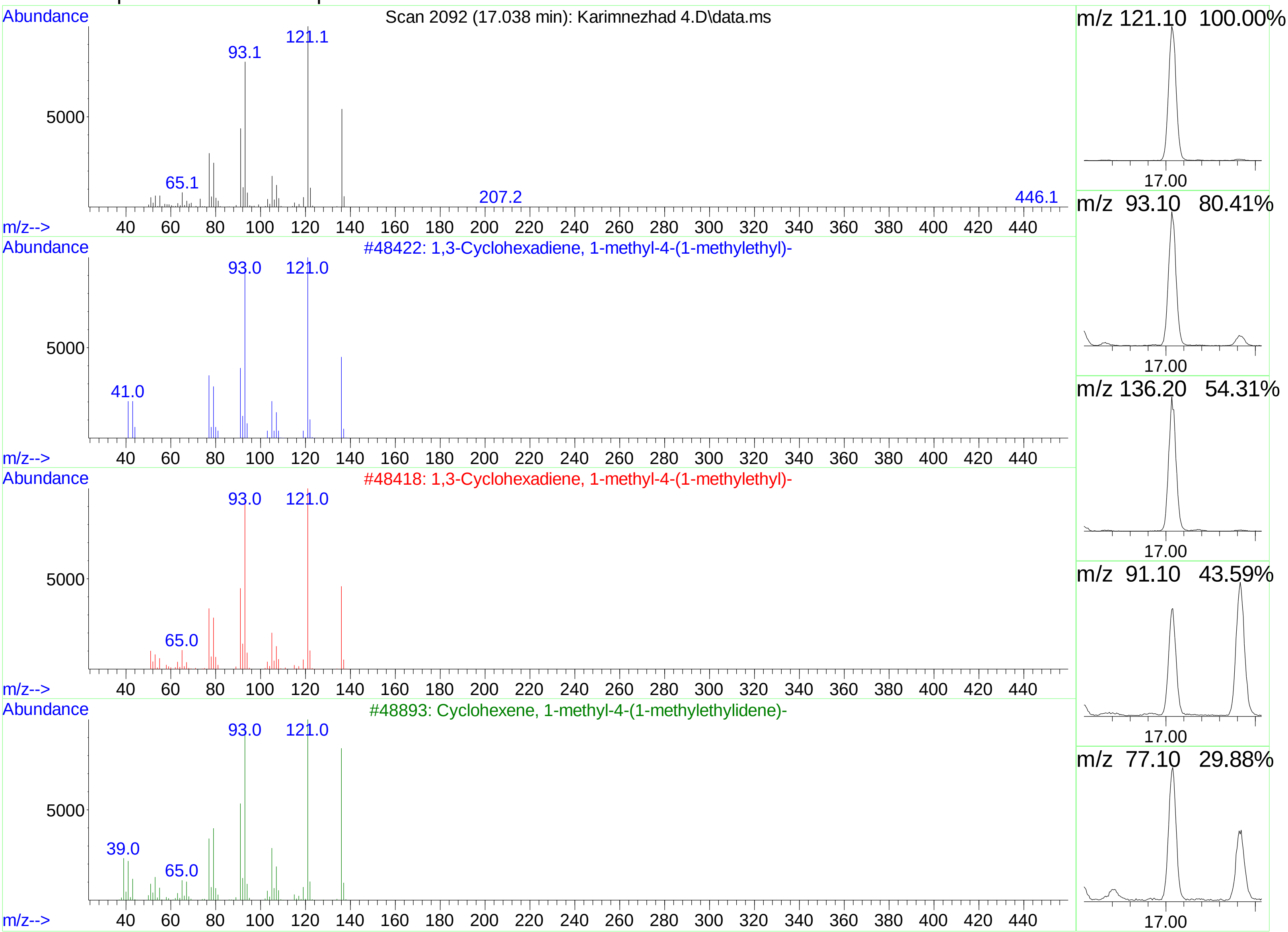

Data File: D:\msdchem\1\data\Karimnezhad 4.D
Sample : M14

Peak Number: 8 at 17.038 min Area: 20132039 Area % 0.09

The 3 best hits from each library.	Ref#	CAS#	Qual
D:\Database\W10N14.L			
1 1,3-Cyclohexadiene, 1-methyl-4-(...	48422	000099-86-5	98
2 1,3-Cyclohexadiene, 1-methyl-4-(...	48418	000099-86-5	97
3 Cyclohexene, 1-methyl-4-(1-methy...	48893	000586-62-9	97

Unknown Spectrum based on Apex

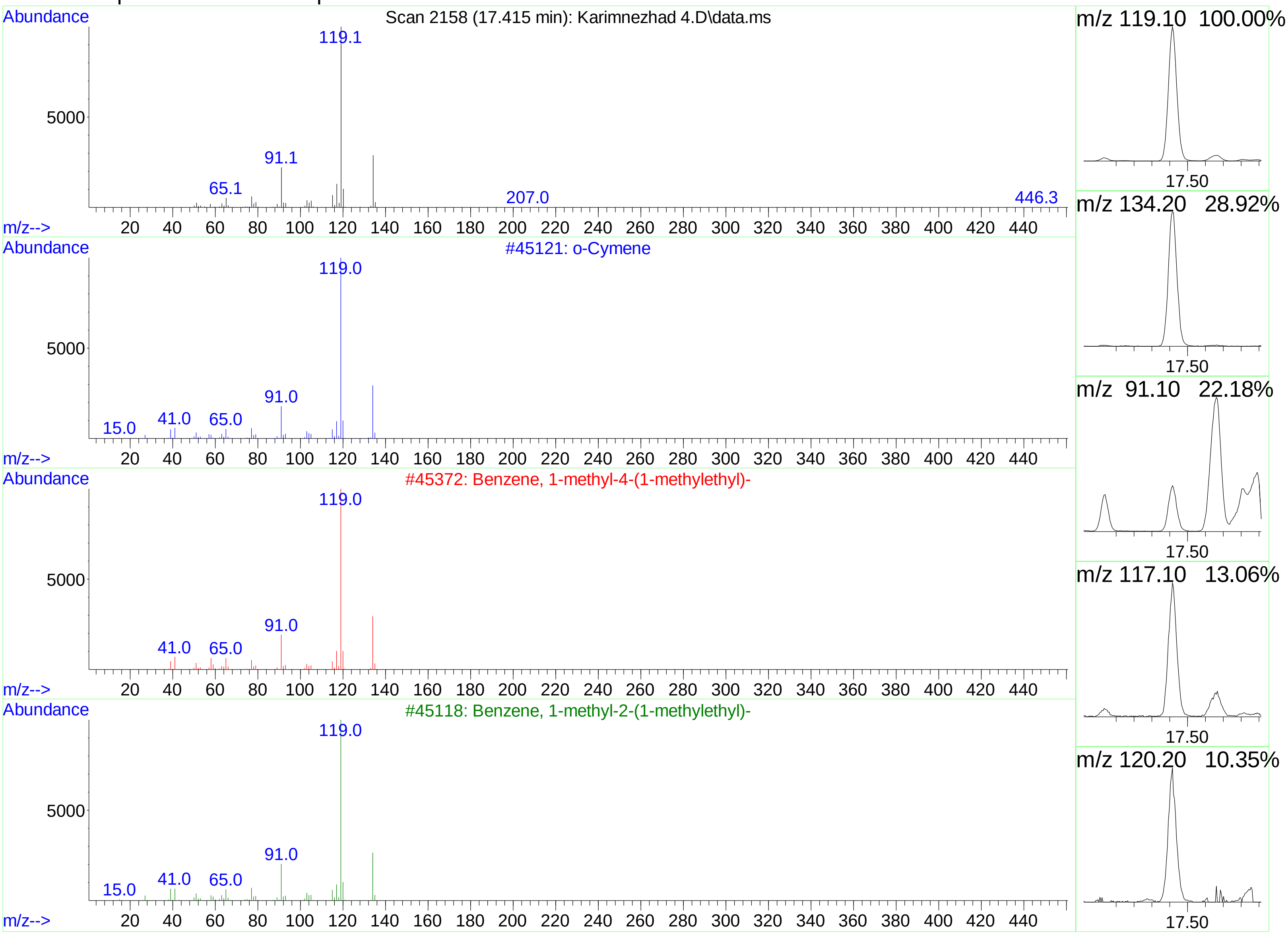

Data File: D:\msdchem\1\data\Karimnezhad 4.D
Sample : M14

Peak Number: 9 at 17.415 min Area: 20978320 Area % 0.09

The 3 best hits from each library.	Ref\#	CAS\#	Qual
D:\Database\W10N14.L			
1 o-Cymene	45121	000527-84-4	97
2 Benzene, 1-methyl-4-(1-methyleth...	45372	000099-87-6	95
3 Benzene, 1-methyl-2-(1-methyleth...	45118	000527-84-4	95

Unknown Spectrum based on Apex

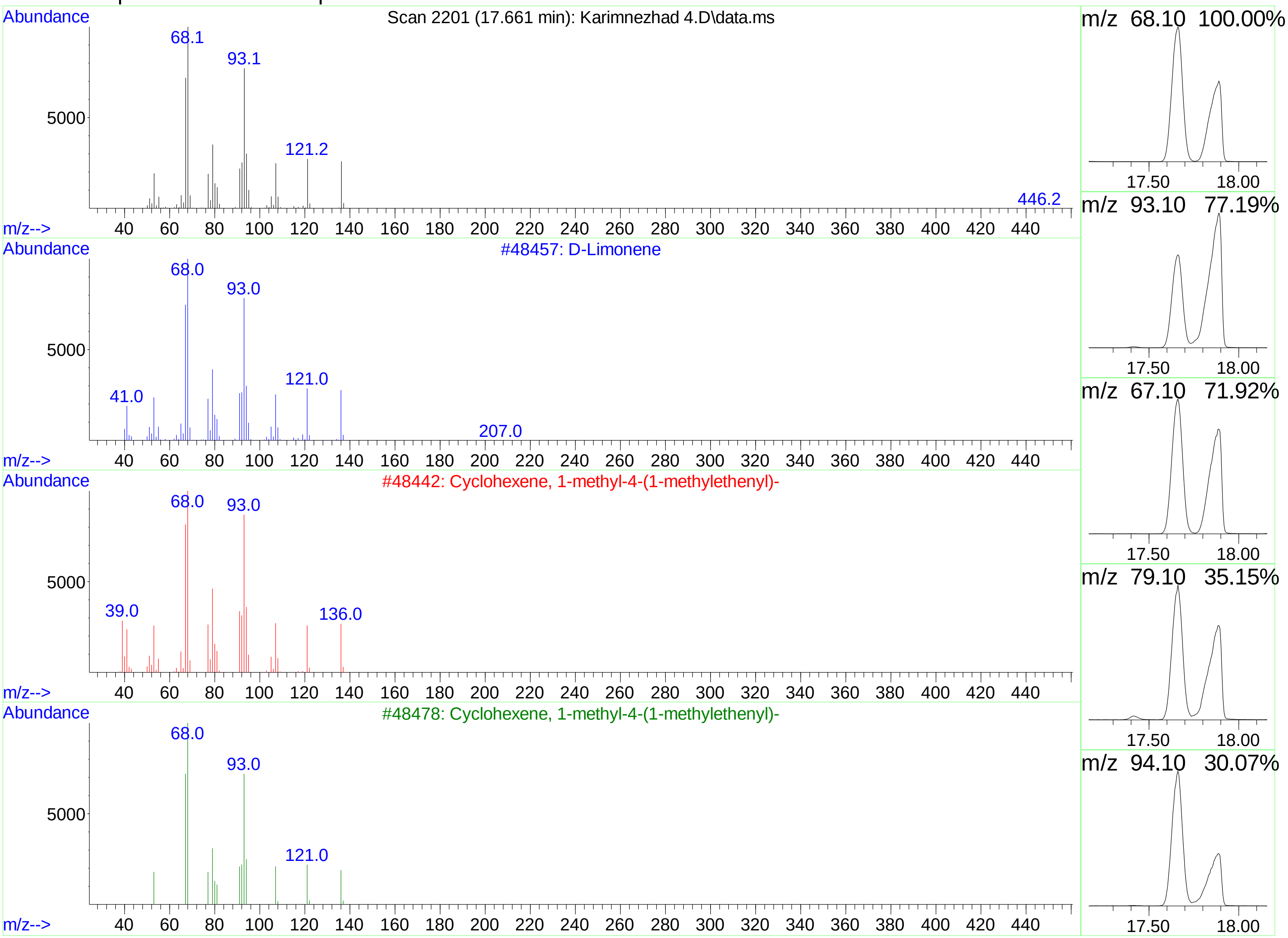

Data File: D:\msdchem\1\data\Karimnezhad 4.D
Sample : M14

Peak Number: 10 at 17.661 min Area: 187310320 Area % 0.82

The 3 best hits from each library.	Ref#	CAS#	Qual
D:\Database\W10N14.L			
1 D-Limonene	48457	005989-27-5	99
2 Cyclohexene, 1-methyl-4-(1-methy...	48442	000138-86-3	98
3 Cyclohexene, 1-methyl-4-(1-methy...	48478	000138-86-3	98

Unknown Spectrum based on Apex

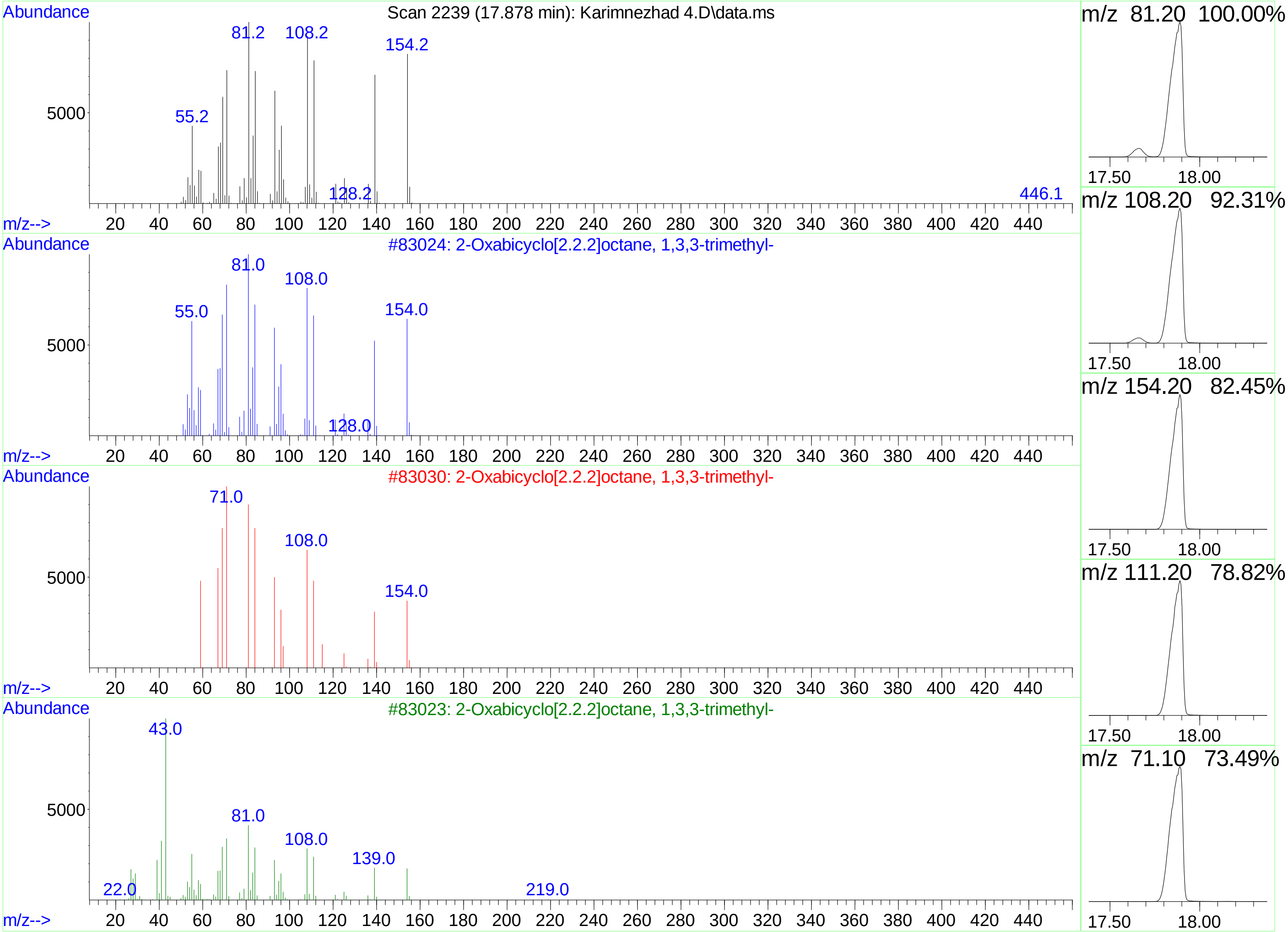

Data File: D:\msdchem\1\data\Karimnezhad 4.D
Sample : M14

Peak Number: 11 at 17.878 min Area: 749494620 Area % 3.27

The 3 best hits from each library.	Ref#	CAS#	Qual
D:\Database\W10N14.L			
1 2-Oxabicyclo[2.2.2]octane, 1,3,3...	83024	000470-82-6	98
2 2-Oxabicyclo[2.2.2]octane, 1,3,3...	83030	000470-82-6	98
3 2-Oxabicyclo[2.2.2]octane, 1,3,3...	83023	000470-82-6	96

Unknown Spectrum based on Apex

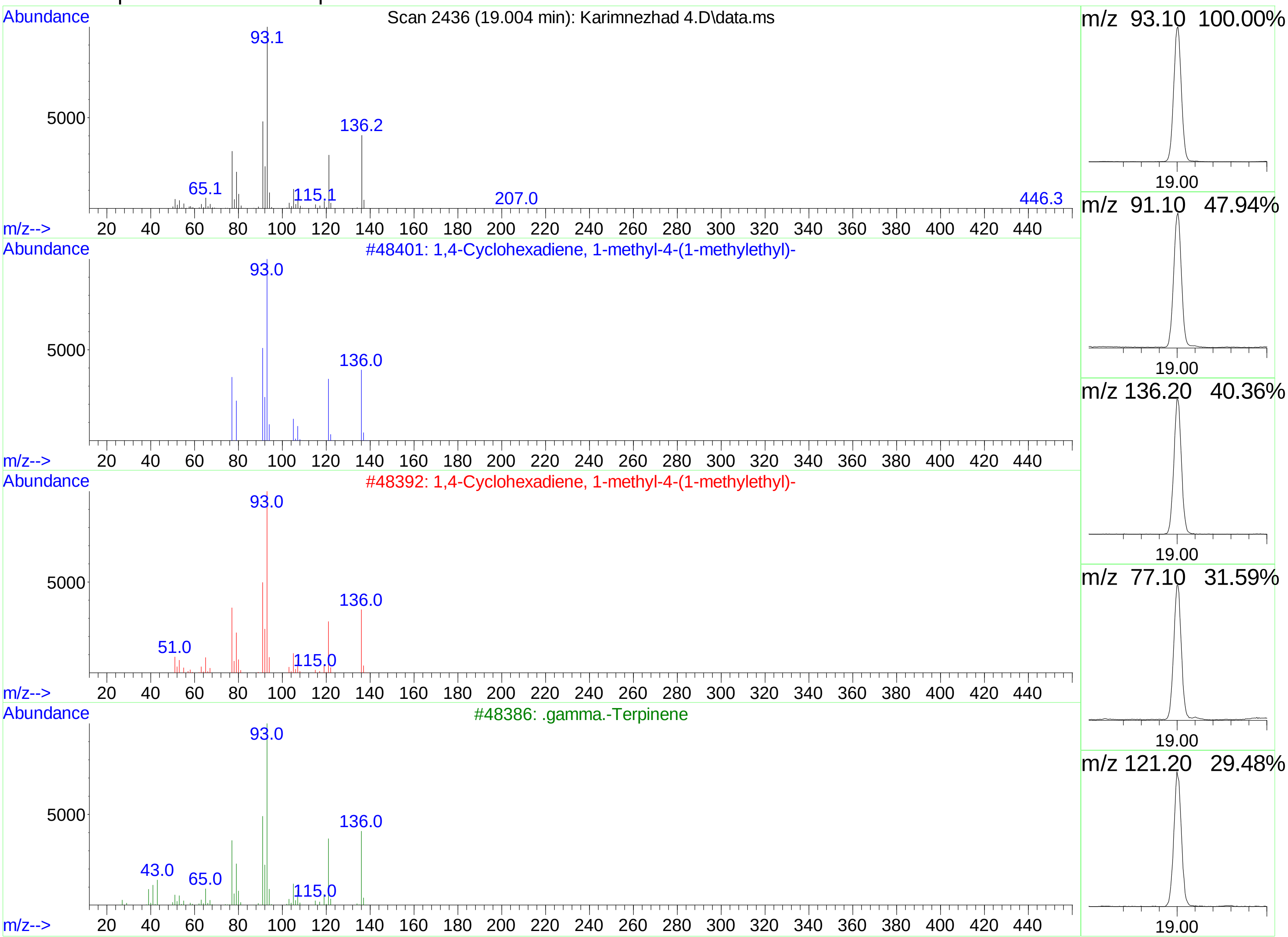

Data File: D:\msdchem\1\data\Karimnezhad 4.D
Sample : M14

Peak Number: 12 at 19.004 min Area: 40776383 Area % 0.18

The 3 best hits from each library.	Ref\#	CAS\#	Qual
D:\Database\W10N14.L			
1 1,4-Cyclohexadiene, 1-methyl-4-(...	48401	000099-85-4	97
2 1,4-Cyclohexadiene, 1-methyl-4-(...	48392	000099-85-4	96
3 .gamma.-Terpinene	48386	000099-85-4	96

Unknown Spectrum based on Apex

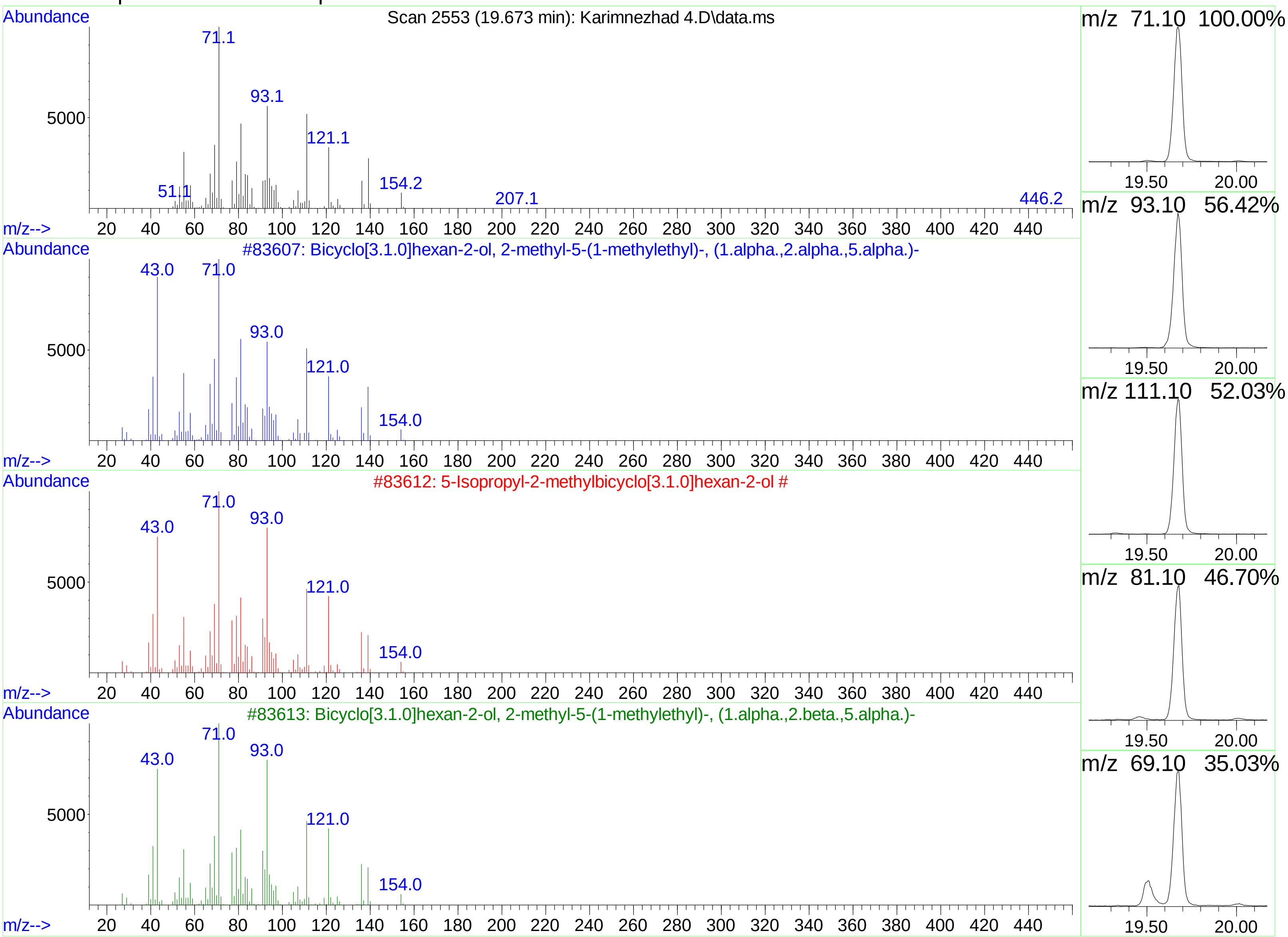

Data File: D:\msdchem\1\data\Karimnezhad 4.D
Sample : M14

Peak Number: 13 at 19.673 min Area: 85348946 Area % 0.37

The 3 best hits from each library.	Ref\#	CAS\#	Qual
D:\Database\W10N14.L			
1 Bicyclo[3.1.0]hexan-2-ol, 2-meth...	83607	017699-16-0	98
2 5-Isopropyl-2-methylbicyclo[3.1....	83612	000546-79-2	96
3 Bicyclo[3.1.0]hexan-2-ol, 2-meth...	83613	015537-55-0	96

Unknown Spectrum based on Apex

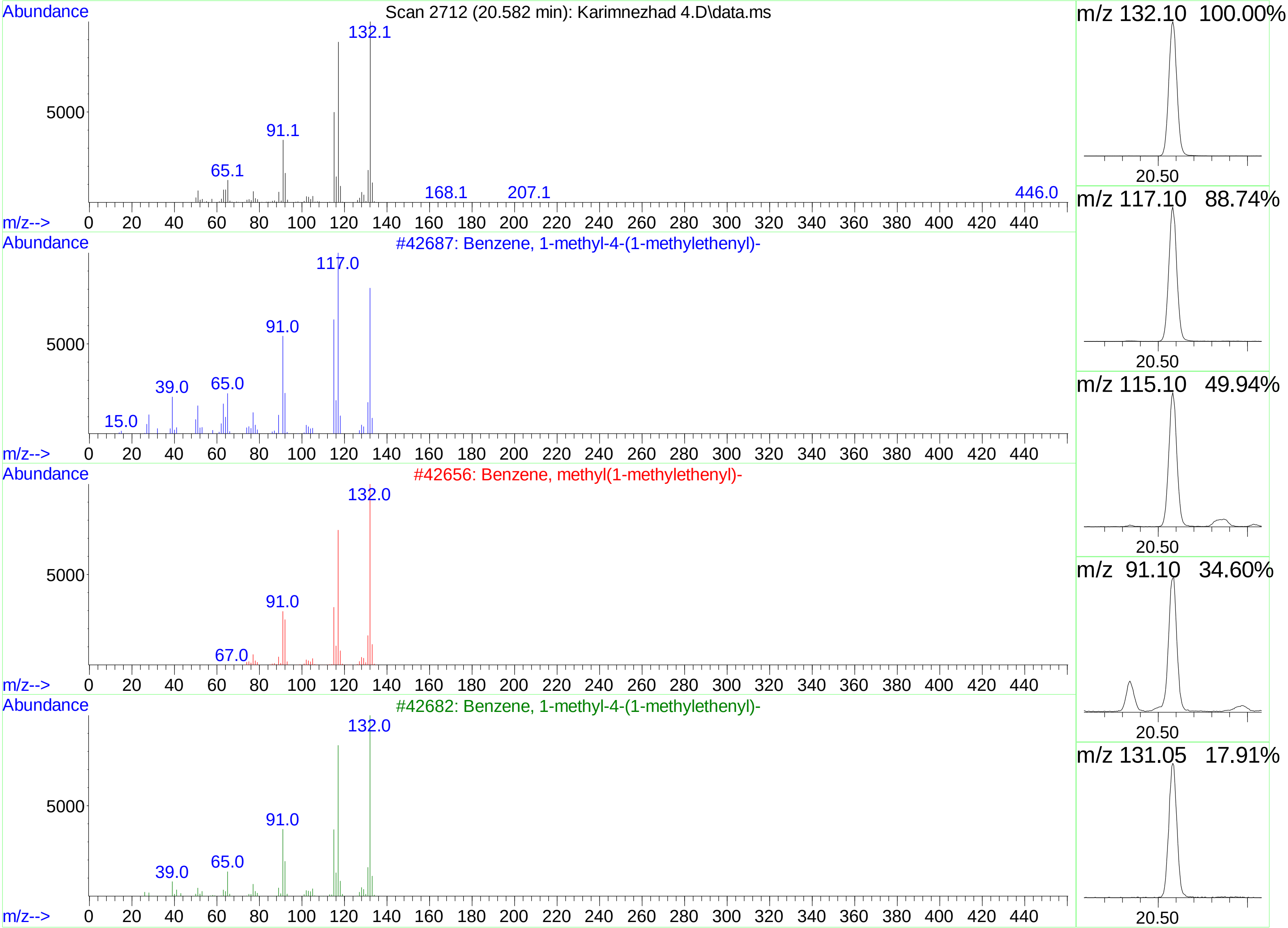

Data File: D:\msdchem\1\data\Karimnezhad 4.D
Sample : M14

Peak Number: 14 at 20.582 min Area: 42820947 Area % 0.19

The 3 best hits from each library.	Ref\#	CAS\#	Qual
D:\Database\W10N14.L			
1 Benzene, 1-methyl-4-(1-methyleth...	42687	001195-32-0	97
2 Benzene, methyl(1-methylethenyl)-	42656	026444-18-8	95
3 Benzene, 1-methyl-4-(1-methyleth...	42682	001195-32-0	94

Unknown Spectrum based on Apex

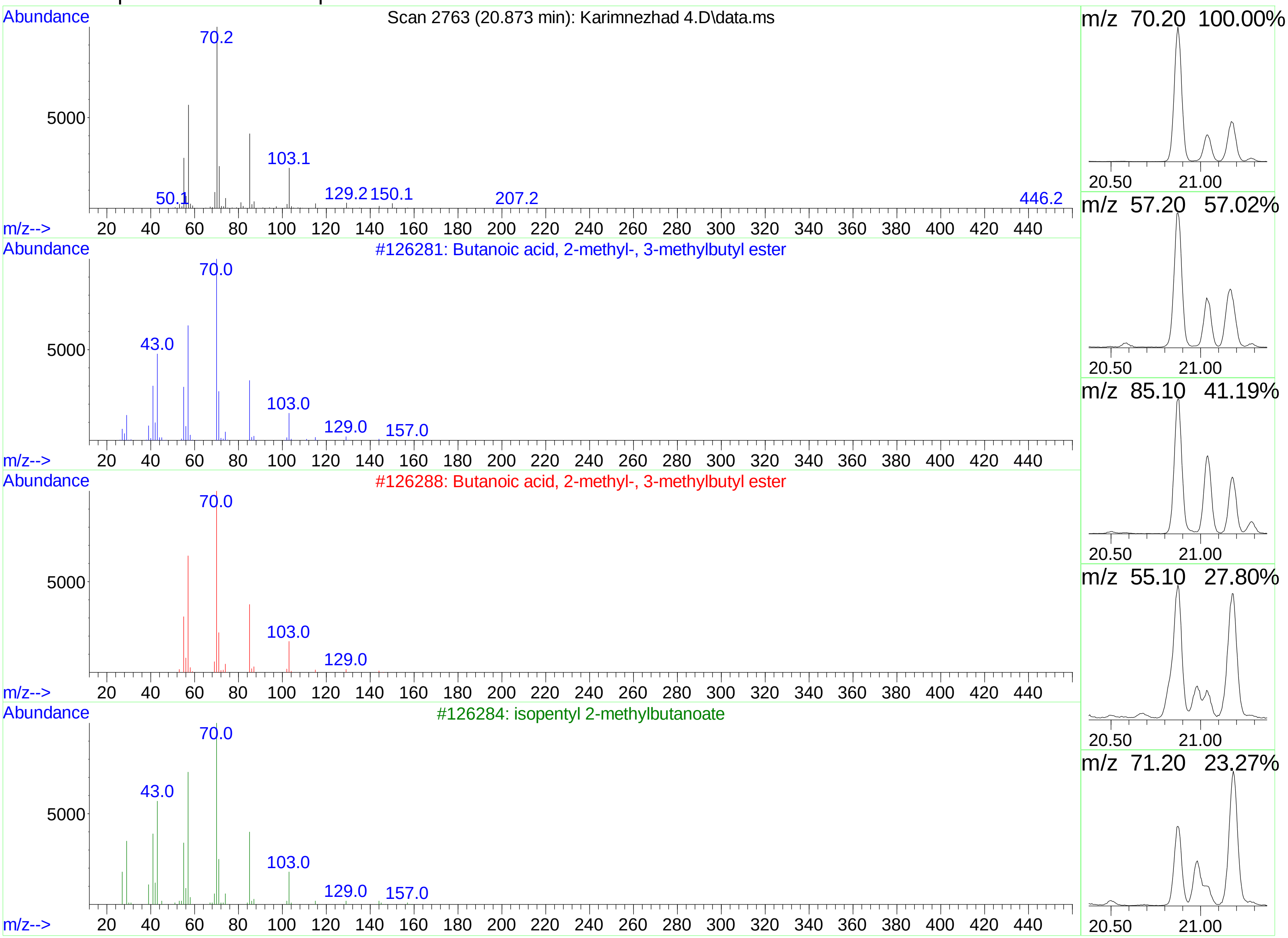

Data File: D:\msdchem\1\data\Karimnezhad 4.D
Sample : M14

Peak Number: 15 at 20.873 min Area: 31573622 Area % 0.14

The 3 best hits from each library. Ref\# CAS\# Qual

D:\Database\W10N14.L

1 Butanoic acid, 2-methyl-, 3-meth...	126281	027625-35-0	86
2 Butanoic acid, 2-methyl-, 3-meth...	126288	027625-35-0	86
3 isopentyl 2-methylbutanoate	126284	027625-35-0	83

Unknown Spectrum based on Apex

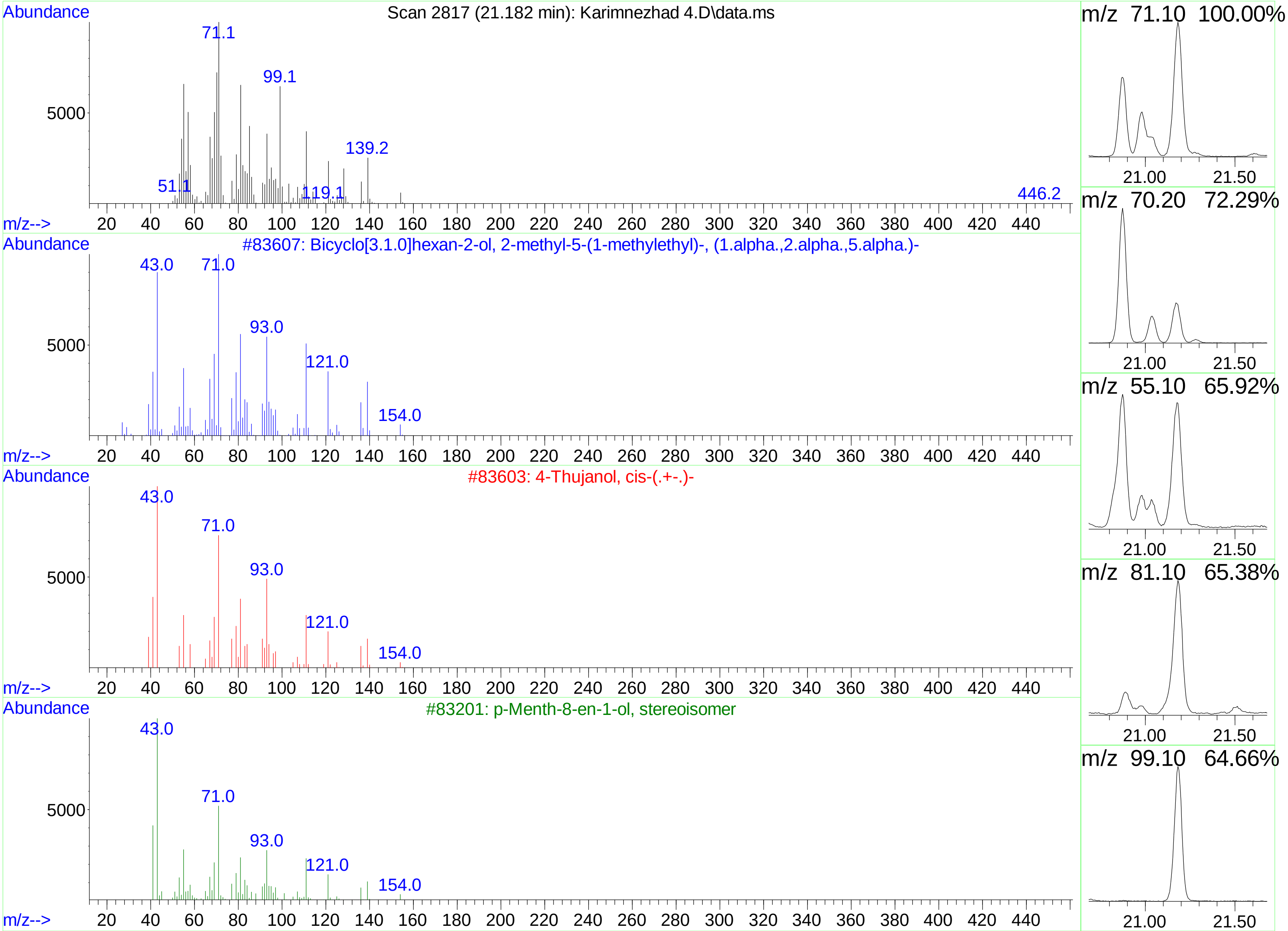

Data File: D:\msdchem\1\data\Karimnezhad 4.D
Sample : M14

Peak Number: 16 at 21.182 min Area: 48922303 Area % 0.21

The 3 best hits from each library.	Ref#	CAS#	Qual
D:\Database\W10N14.L			
1 Bicyclo[3.1.0]hexan-2-ol, 2-meth...	83607	017699-16-0	93
2 4-Thujanol, cis-(.+-.)-	83603	015826-82-1	52
3 p-Menth-8-en-1-ol, stereoisomer	83201	007299-40-3	50

Unknown Spectrum based on Apex

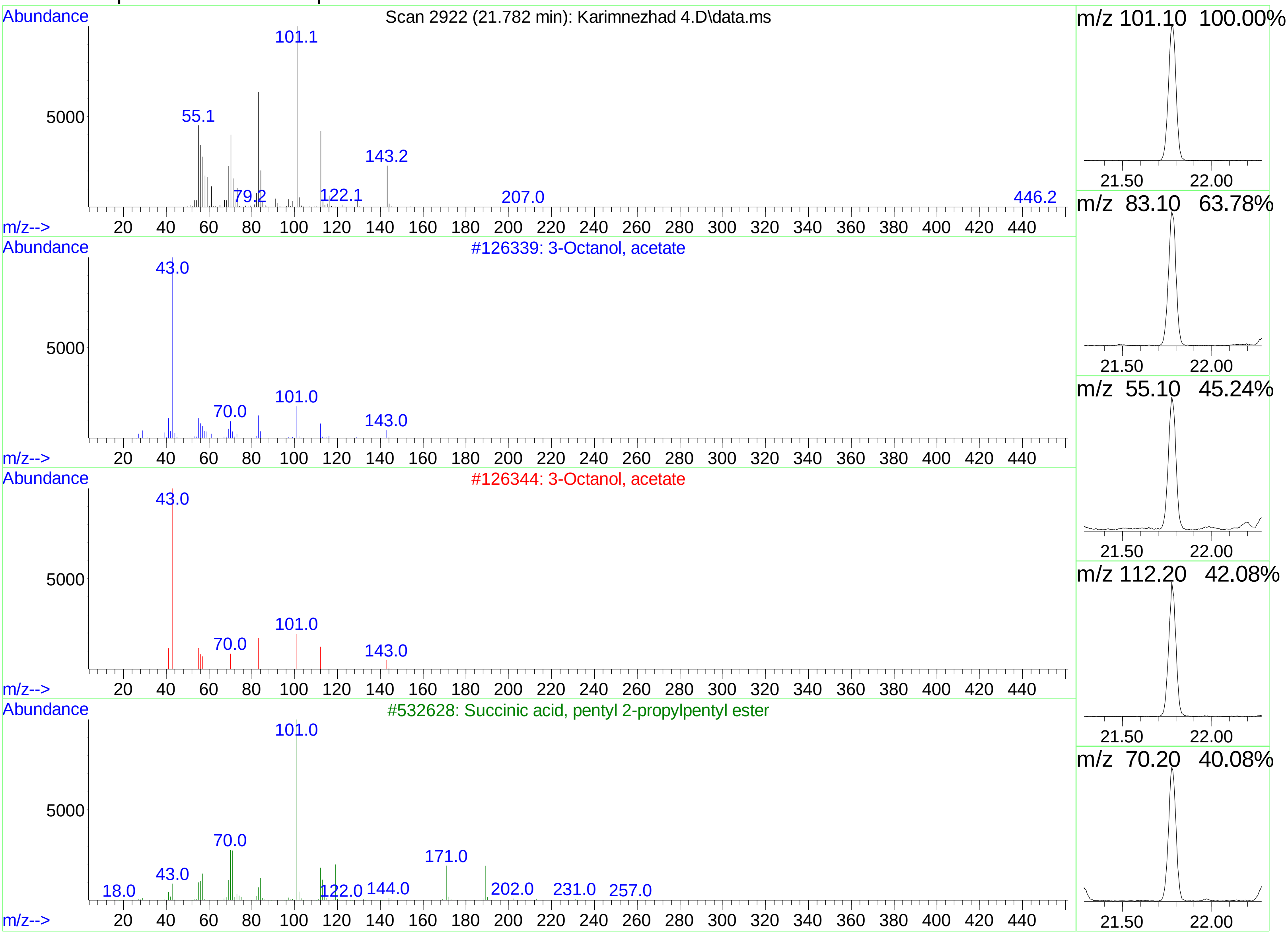

Data File: D:\msdchem\1\data\Karimnezhad 4.D
Sample : M14

Peak Number: 17 at 21.782 min Area: 29356567 Area % 0.13

The 3 best hits from each library.	Ref\#	CAS\#	Qual
D:\Database\W10N14.L			
1 3-Octanol, acetate	126339	004864-61-3	91
2 3-Octanol, acetate	126344	004864-61-3	50
3 Succinic acid, pentyl 2-propylpe...	532628	2000532-62-8	43

Unknown Spectrum based on Apex

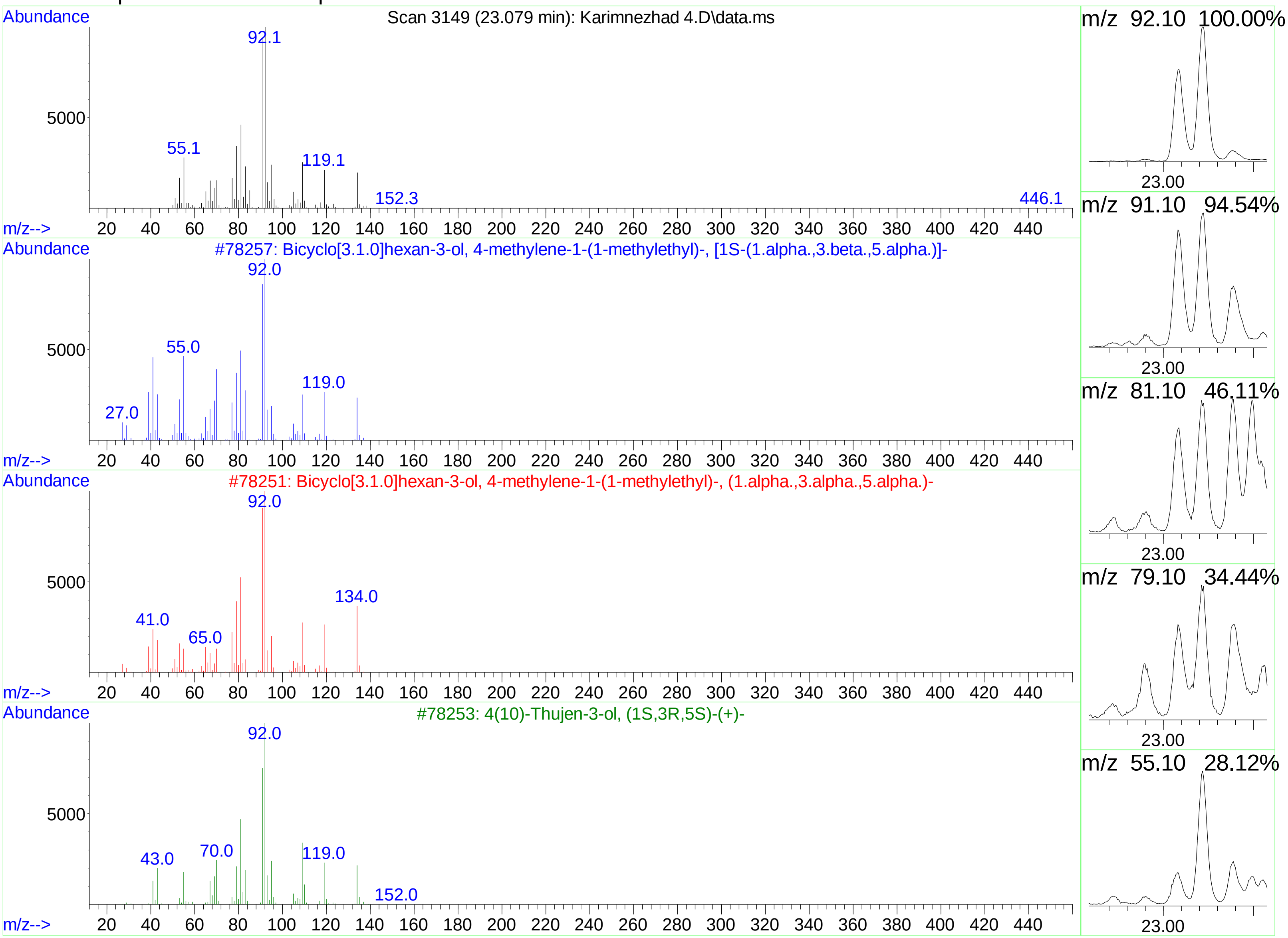

Data File: D:\msdchem\1\data\Karimnezhad 4.D
Sample : M14

Peak Number: 18 at 23.079 min Area: 19061413 Area % 0.08

The 3 best hits from each library.	Ref\#	CAS\#	Qual
D:\Database\W10N14.L			
1 Bicyclo[3.1.0]hexan-3-ol, 4-meth...	78257	000471-16-9	87
2 Bicyclo[3.1.0]hexan-3-ol, 4-meth...	78251	003310-02-9	83
3 4(10)-Thujen-3-ol, (1S,3R,5S)-(+)-	78253	000471-16-9	80

Unknown Spectrum based on Apex

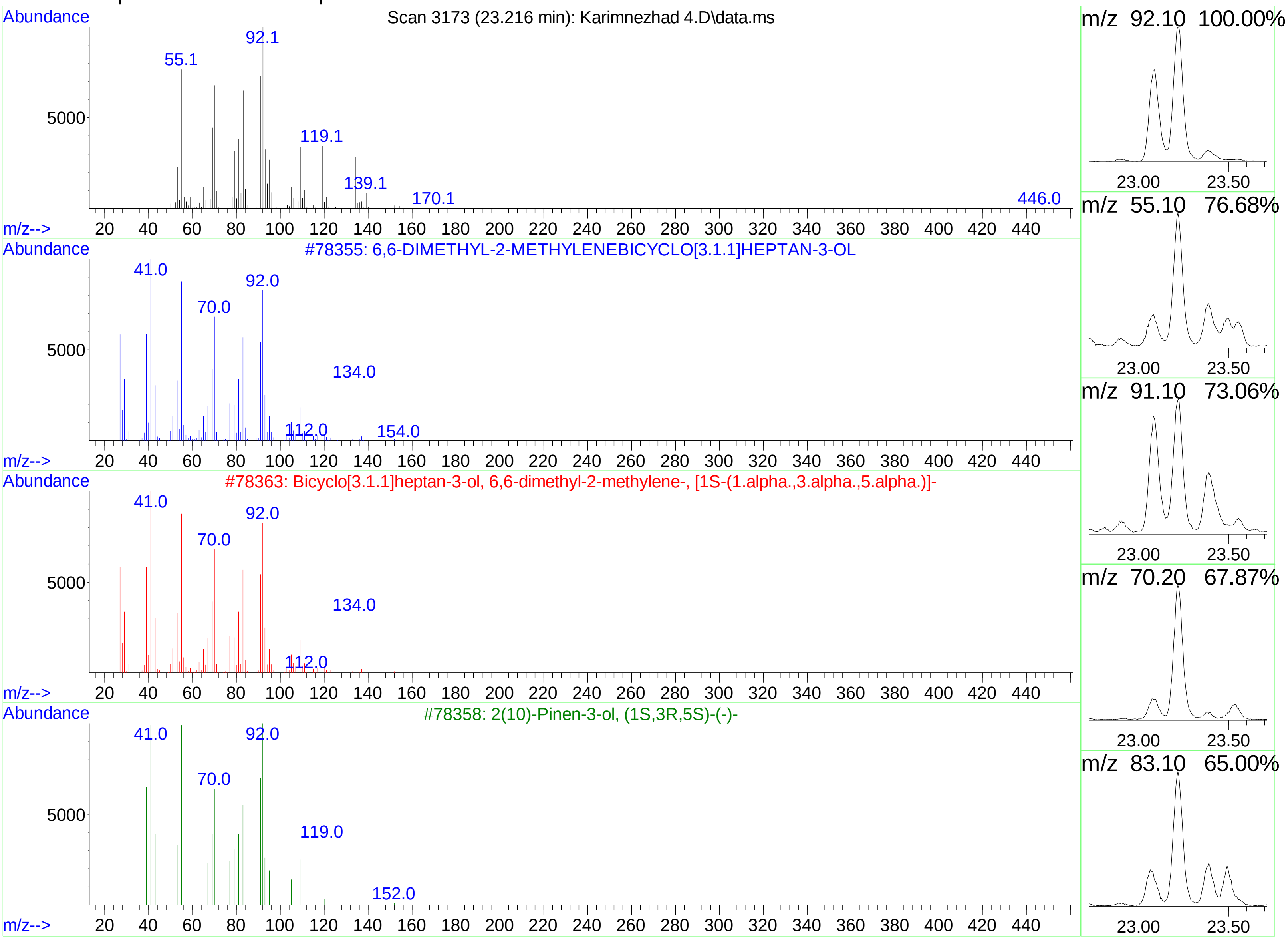

Data File: D:\msdchem\1\data\Karimnezhad 4.D
Sample : M14

Peak Number: 19 at 23.216 min Area: 40594526 Area % 0.18

The 3 best hits from each library. Ref\# CAS\# Qual

D:\Database\W10N14.L

1	6,6-DIMETHYL-2-METHYLENEBICYCLO[...	78355	000547-61-5	93
2	Bicyclo[3.1.1]heptan-3-ol, 6,6-d...	78363	000547-61-5	93
3	2(10)-Pinen-3-ol, (1S,3R,5S)-(-)-	78358	000547-61-5	93

Library Search Report - ChemStation Integrator

Unknown Spectrum based on Apex

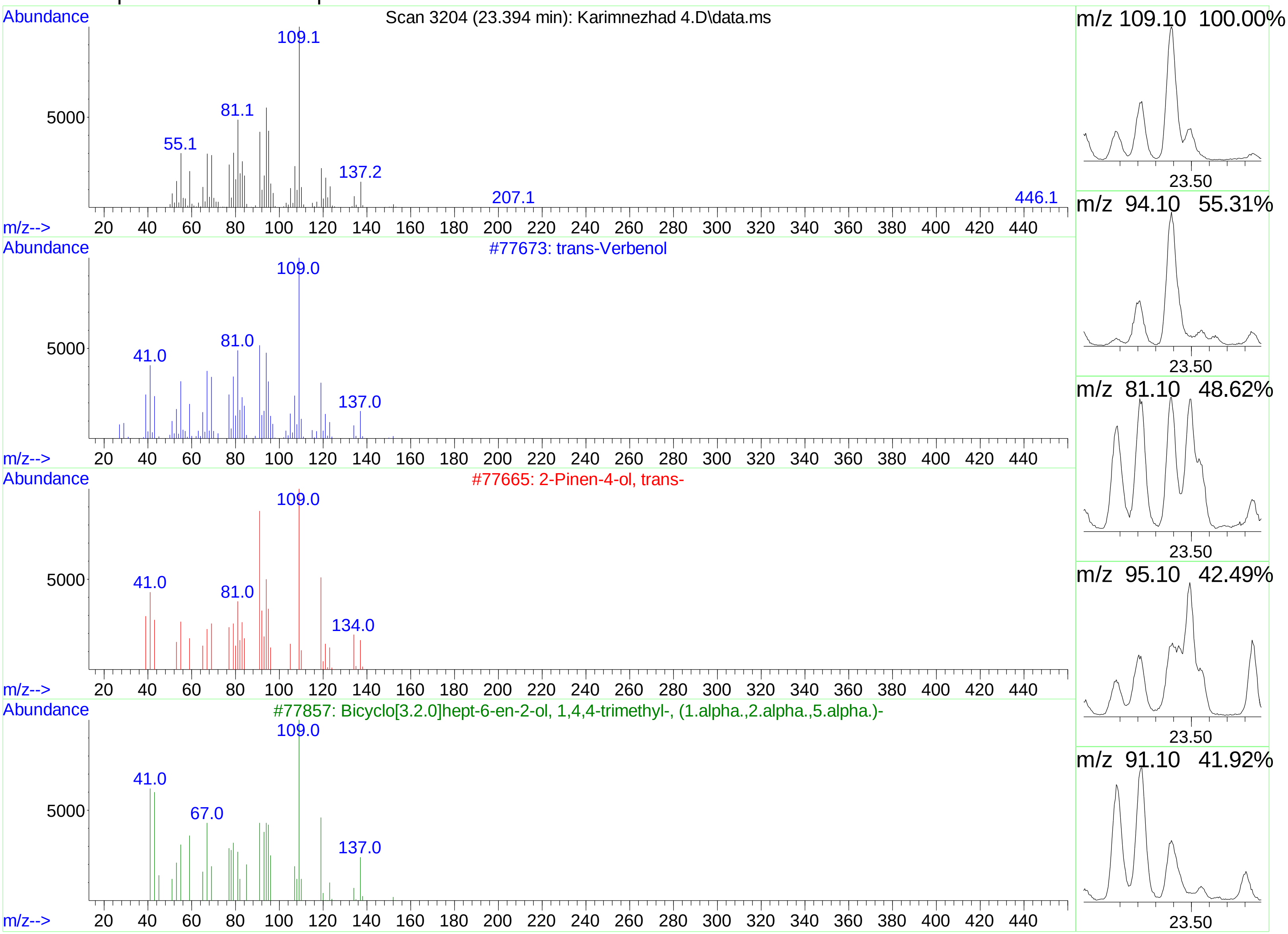

Data File: D:\msdchem\1\data\Karimnezhad 4.D
Sample : M14

Peak Number: 20 at 23.394 min Area: 30780507 Area % 0.13

The 3 best hits from each library.	Ref#	CAS#	Qual
D:\Database\W10N14.L			
1 trans-Verbenol	77673	001820-09-3	94
2 2-Pinen-4-ol, trans-	77665	001820-09-3	64
3 Bicyclo[3.2.0]hept-6-en-2-ol, 1,...	77857	014590-83-1	49

Unknown Spectrum based on Apex

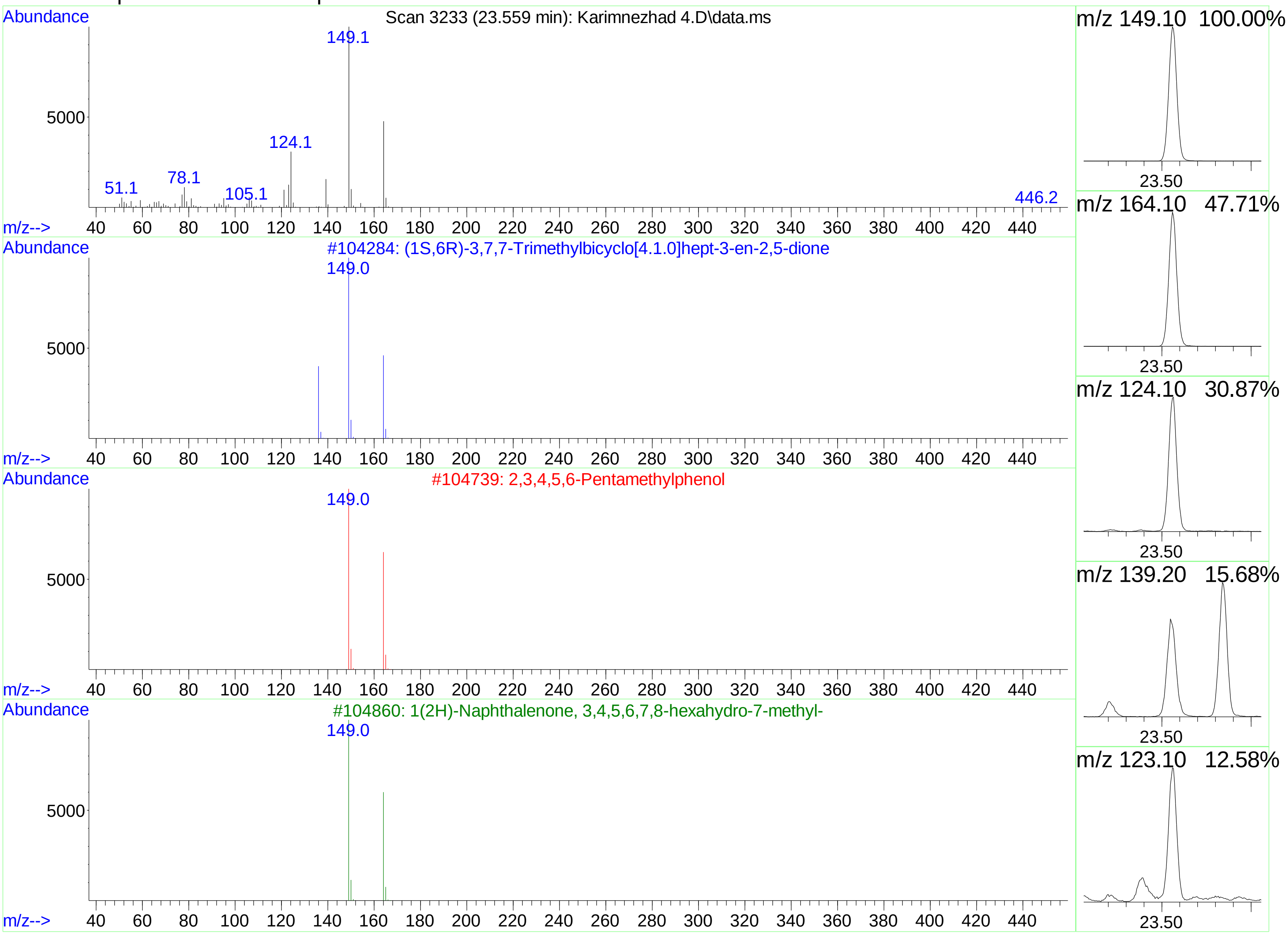

Data File: D:\msdchem\1\data\Karimnezhad 4.D
Sample : M14

Peak Number: 21 at 23.559 min Area: 63127318 Area % 0.28

The 3 best hits from each library. Ref\# CAS\# Qual

D:\Database\W10N14.L

1	(1S,6R)-3,7,7-Trimethylbicyclo[4...	104284	2000104-28-4	86
2	2,3,4,5,6-Pentamethylphenol	104739	2000104-73-9	83
3	1(2H)-Naphthalenone, 3,4,5,6,7,8...	104860	059177-21-8	83

Library Search Report - ChemStation Integrator

Unknown Spectrum based on Apex

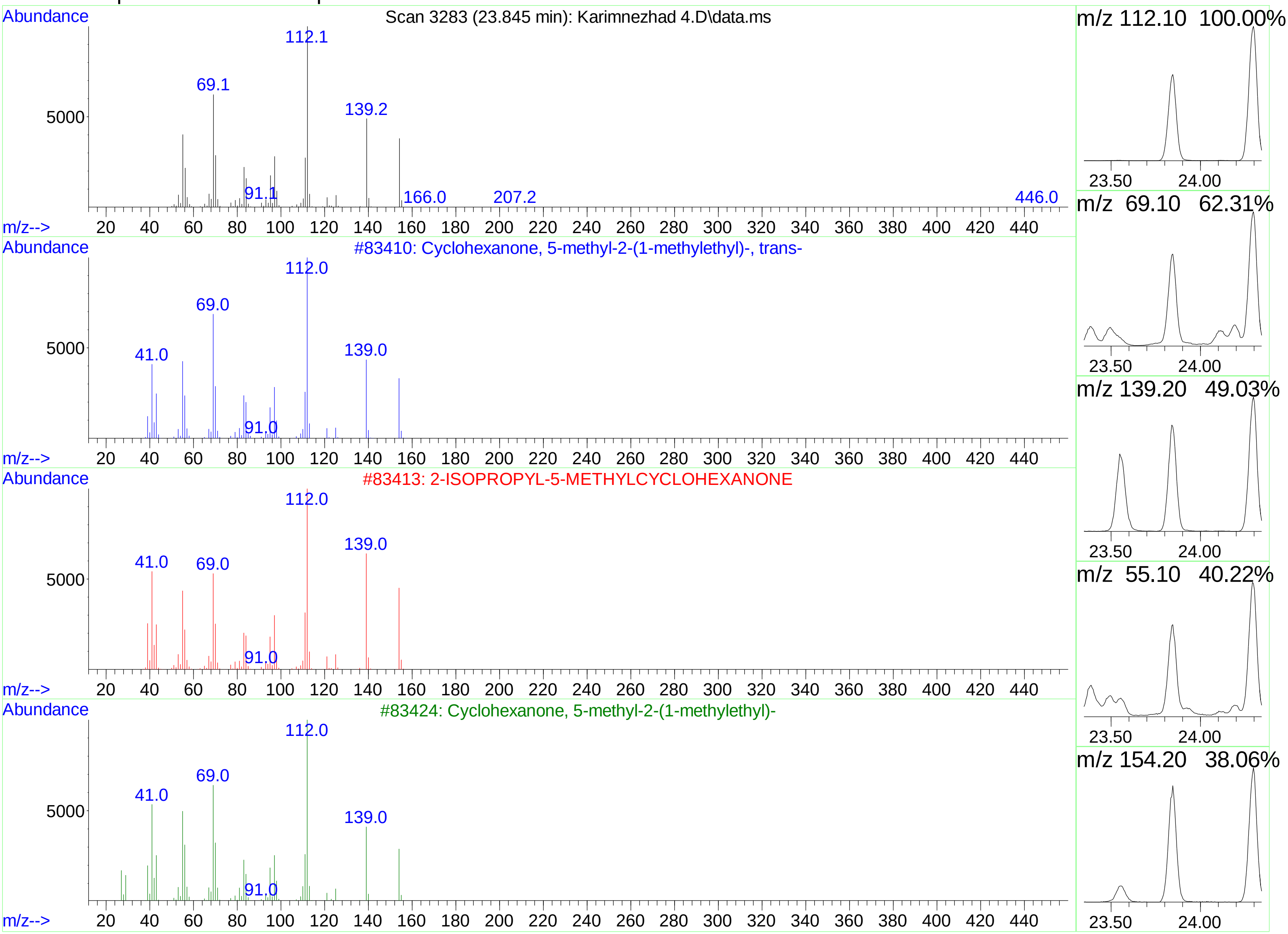

Data File: D:\msdchem\1\data\Karimnezhad 4.D
Sample : M14

Peak Number: 22 at 23.845 min Area: 45551929 Area % 0.20

The 3 best hits from each library. Ref\# CAS\# Qual

D:\Database\W10N14.L

1	Cyclohexanone, 5-methyl-2-(1-met...	83410	000089-80-5	98
2	2-ISOPROPYL-5-METHYLCYCLOHEXANONE	83413	000089-80-5	98
3	Cyclohexanone, 5-methyl-2-(1-met...	83424	010458-14-7	97

Unknown Spectrum based on Apex

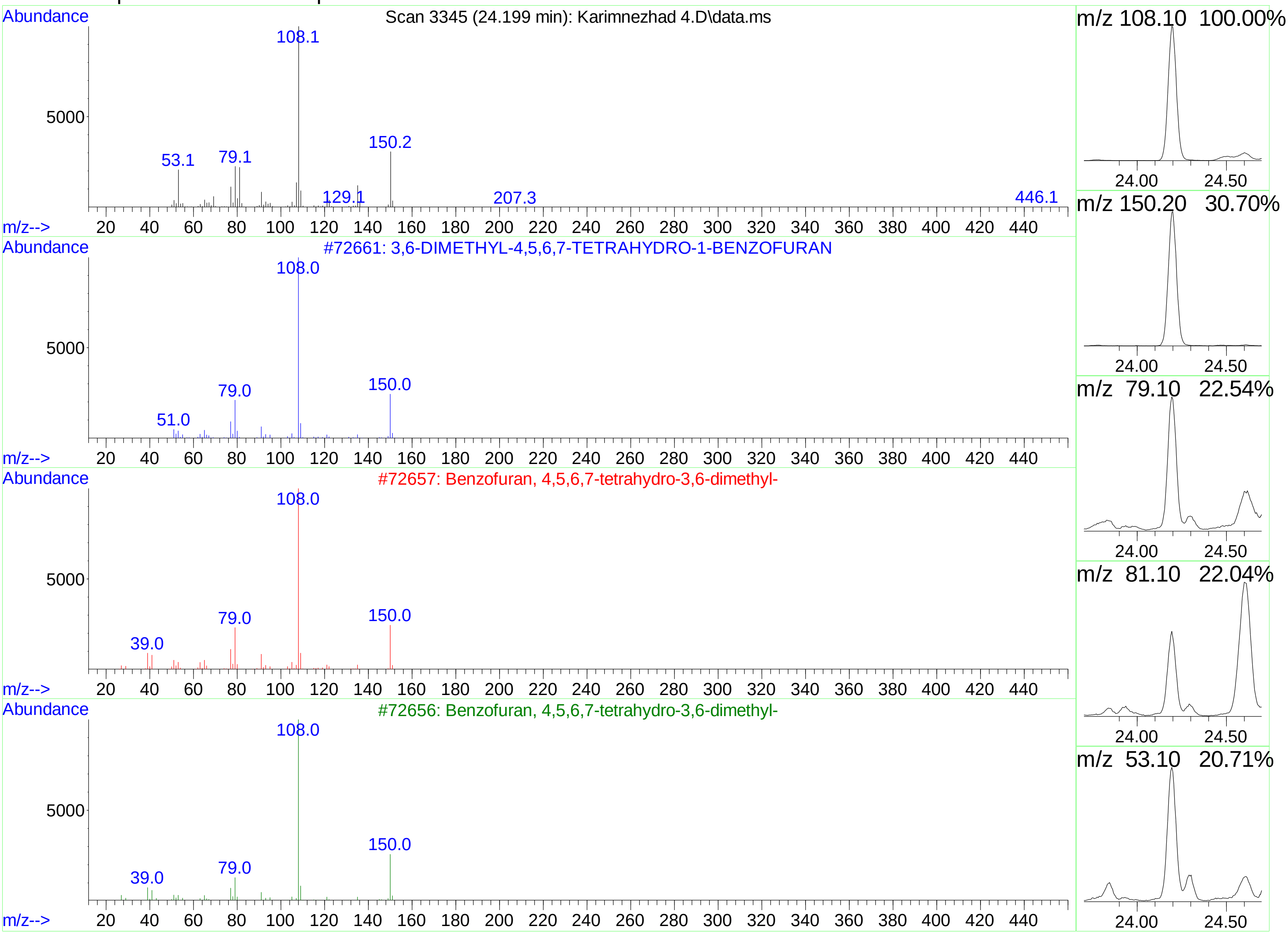

Data File: D:\msdchem\1\data\Karimnezhad 4.D
Sample : M14

Peak Number: 23 at 24.199 min Area: 52676285 Area % 0.23

The 3 best hits from each library. Ref\# CAS\# Qual

D:\Database\W10N14.L

1	3,6-DIMETHYL-4,5,6,7-TETRAHYDRO-...	72661	000494-90-6	93
2	Benzofuran, 4,5,6,7-tetrahydro-3...	72657	000494-90-6	76
3	Benzofuran, 4,5,6,7-tetrahydro-3...	72656	000494-90-6	76

Library Search Report - ChemStation Integrator

Unknown Spectrum based on Apex

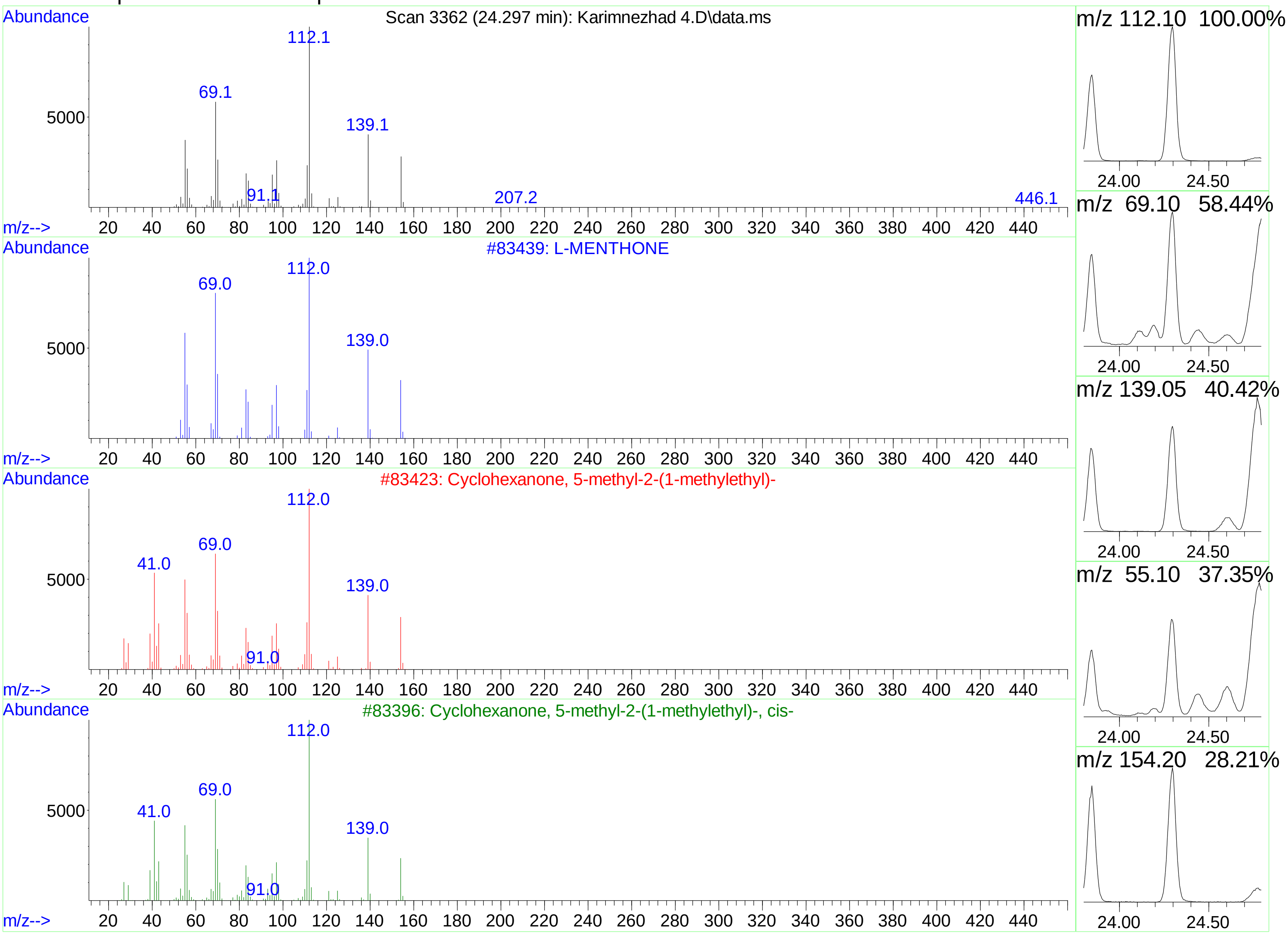

Data File: D:\msdchem\1\data\Karimnezhad 4.D
Sample : M14

Peak Number: 24 at 24.297 min Area: 52749702 Area % 0.23

The 3 best hits from each library.	Ref\#	CAS\#	Qual
D:\Database\W10N14.L			
1 L-MENTHONE	83439	010458-14-7	98
2 Cyclohexanone, 5-methyl-2-(1-met...	83423	010458-14-7	98
3 Cyclohexanone, 5-methyl-2-(1-met...	83396	000491-07-6	98

Unknown Spectrum based on Apex

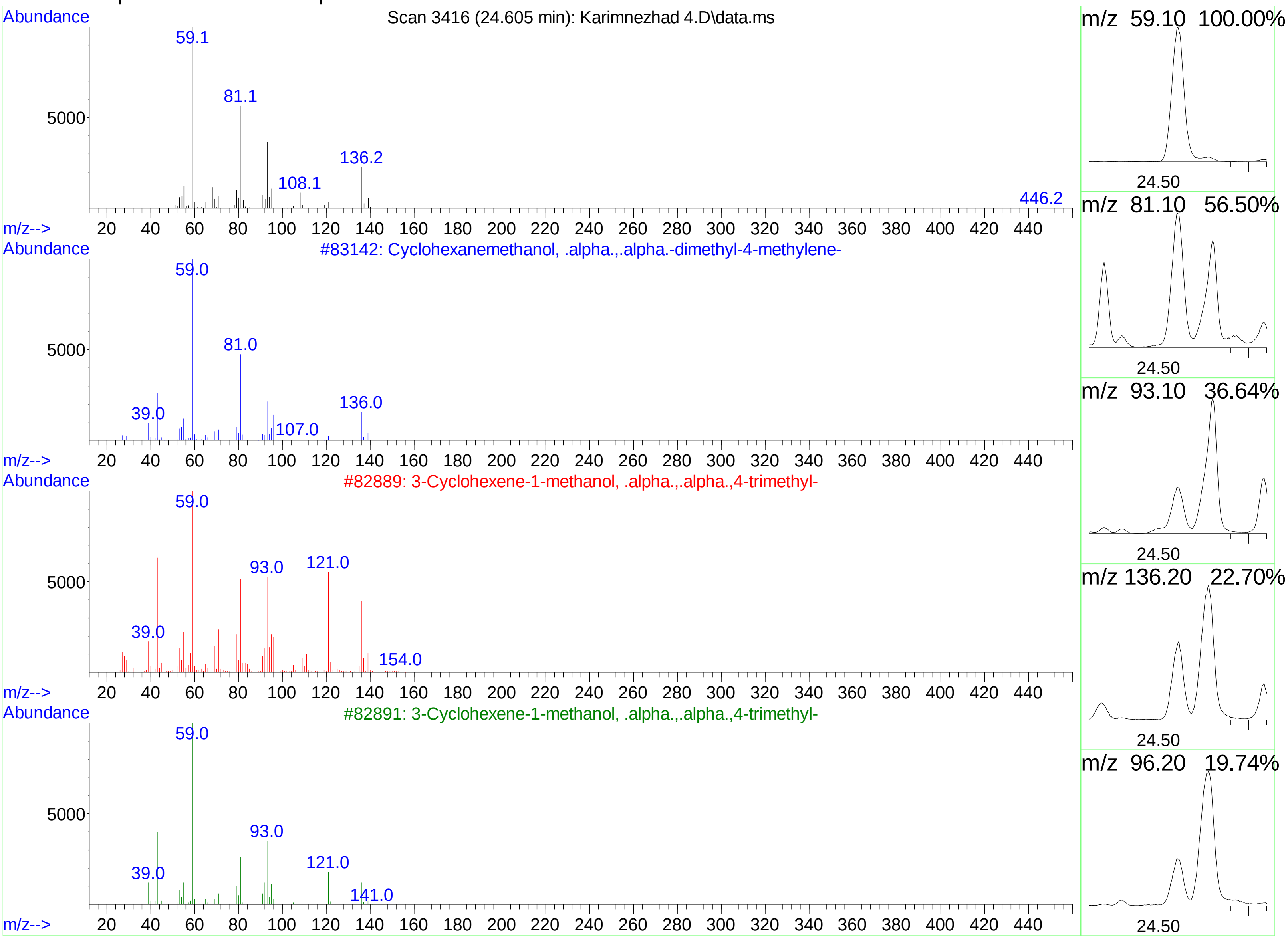

Data File: D:\msdchem\1\data\Karimnezhad 4.D
Sample : M14

Peak Number: 25 at 24.605 min Area: 68701253 Area % 0.30

The 3 best hits from each library.	Ref#	CAS#	Qual
D:\Database\W10N14.L			
1 Cyclohexanemethanol, .alpha.,.al...	83142	007299-42-5	90
2 3-Cyclohexene-1-methanol, .alpha...	82889	010482-56-1	53
3 3-Cyclohexene-1-methanol, .alpha...	82891	010482-56-1	53

Library Search Report - ChemStation Integrator

Unknown Spectrum based on Apex

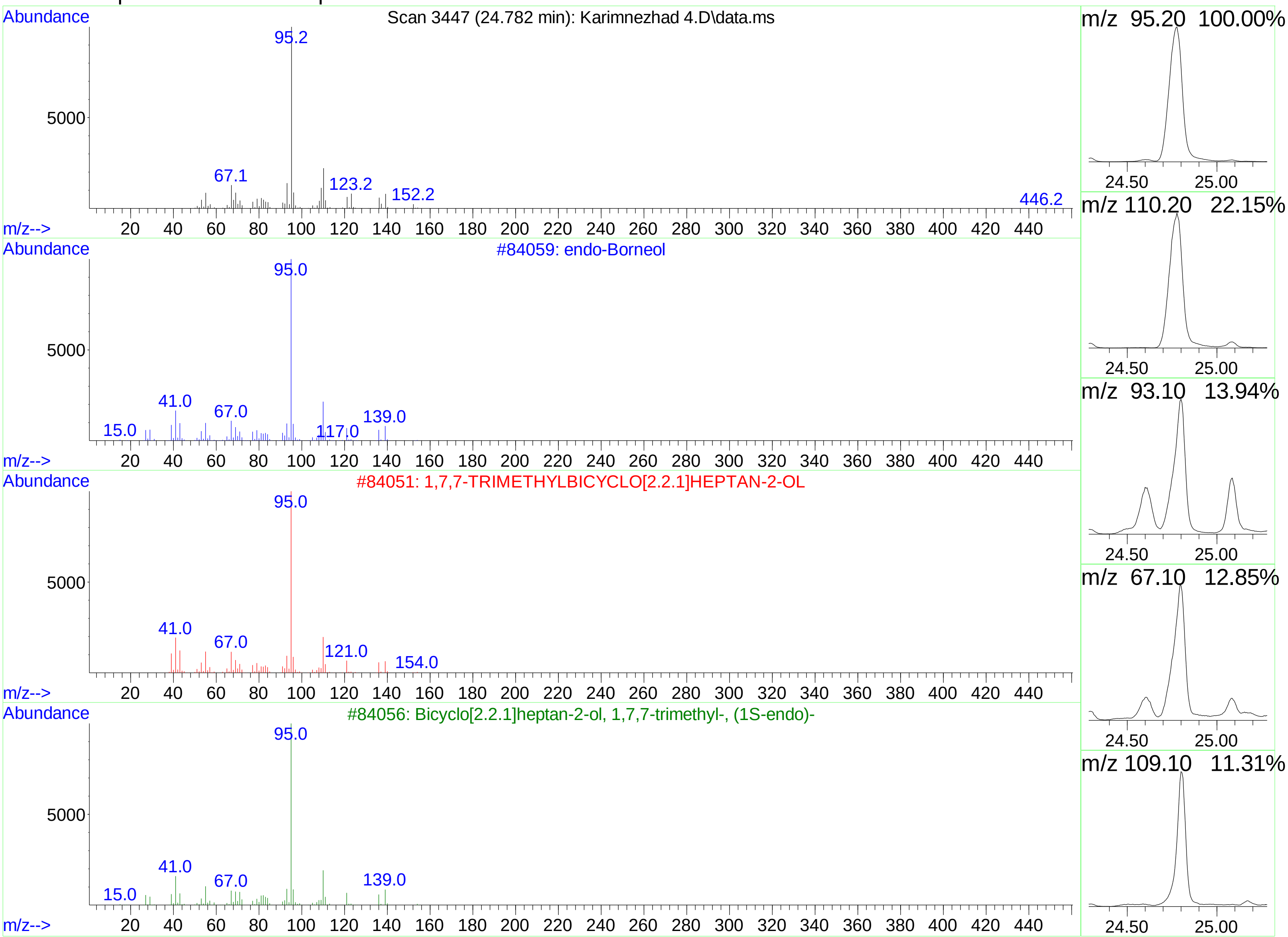

Data File: D:\msdchem\1\data\Karimnezhad 4.D
Sample : M14

Peak Number: 26 at 24.782 min Area: 332929030 Area % 1.45

The 3 best hits from each library.	Ref#	CAS#	Qual
D:\Database\W10N14.L			
1 endo-Borneol	84059	000507-70-0	94
2 1,7,7-TRIMETHYLBICYCLO[2.2.1]HEP...	84051	000464-45-9	94
3 Bicyclo[2.2.1]heptan-2-ol, 1,7,7...	84056	000464-45-9	90

Unknown Spectrum based on Apex

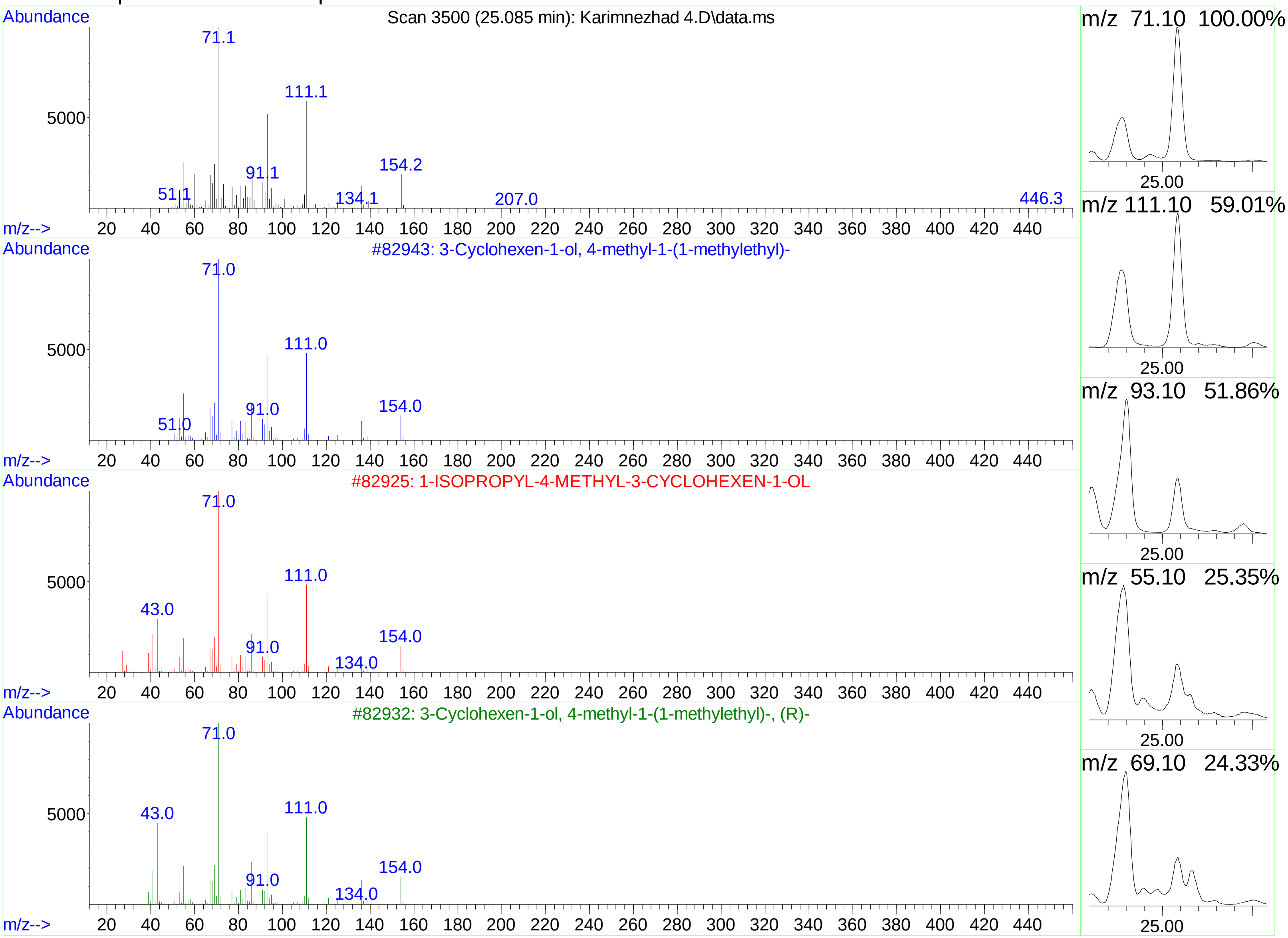

Data File: D:\msdchem\1\data\Karimnezhad 4.D
Sample : M14

Peak Number: 27 at 25.085 min Area: 91300238 Area % 0.40

The 3 best hits from each library.	Ref\#	CAS\#	Qual
D:\Database\W10N14.L			
1 3-Cyclohexen-1-ol, 4-methyl-1-(1...	82943	000562-74-3	98
2 1-ISOPROPYL-4-METHYL-3-CYCLOHEXE...	82925	000562-74-3	98
3 3-Cyclohexen-1-ol, 4-methyl-1-(1...	82932	020126-76-5	96

Library Search Report - ChemStation Integrator

Unknown Spectrum based on Apex

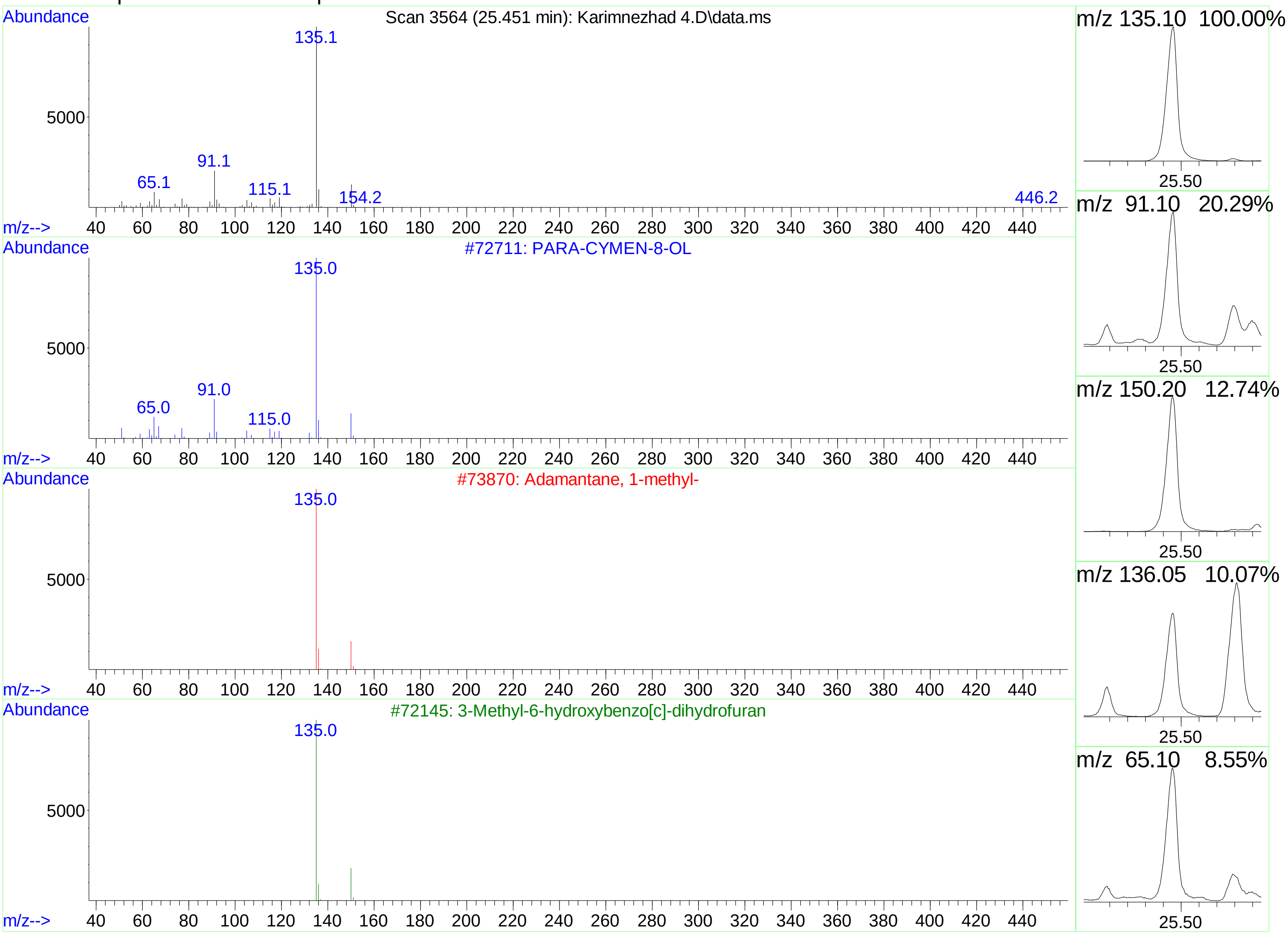

Data File: D:\msdchem\1\data\Karimnezhad 4.D
Sample : M14

Peak Number: 28 at 25.451 min Area: 124334171 Area % 0.54

The 3 best hits from each library.	Ref#	CAS#	Qual
D:\Database\W10N14.L			
1 PARA-CYMEN-8-OL	72711	001197-01-9	93
2 Adamantane, 1-methyl-	73870	000768-91-2	83
3 3-Methyl-6-hydroxybenzo[c]-dihyd...	72145	2000072-14-5	83

Unknown Spectrum based on Apex

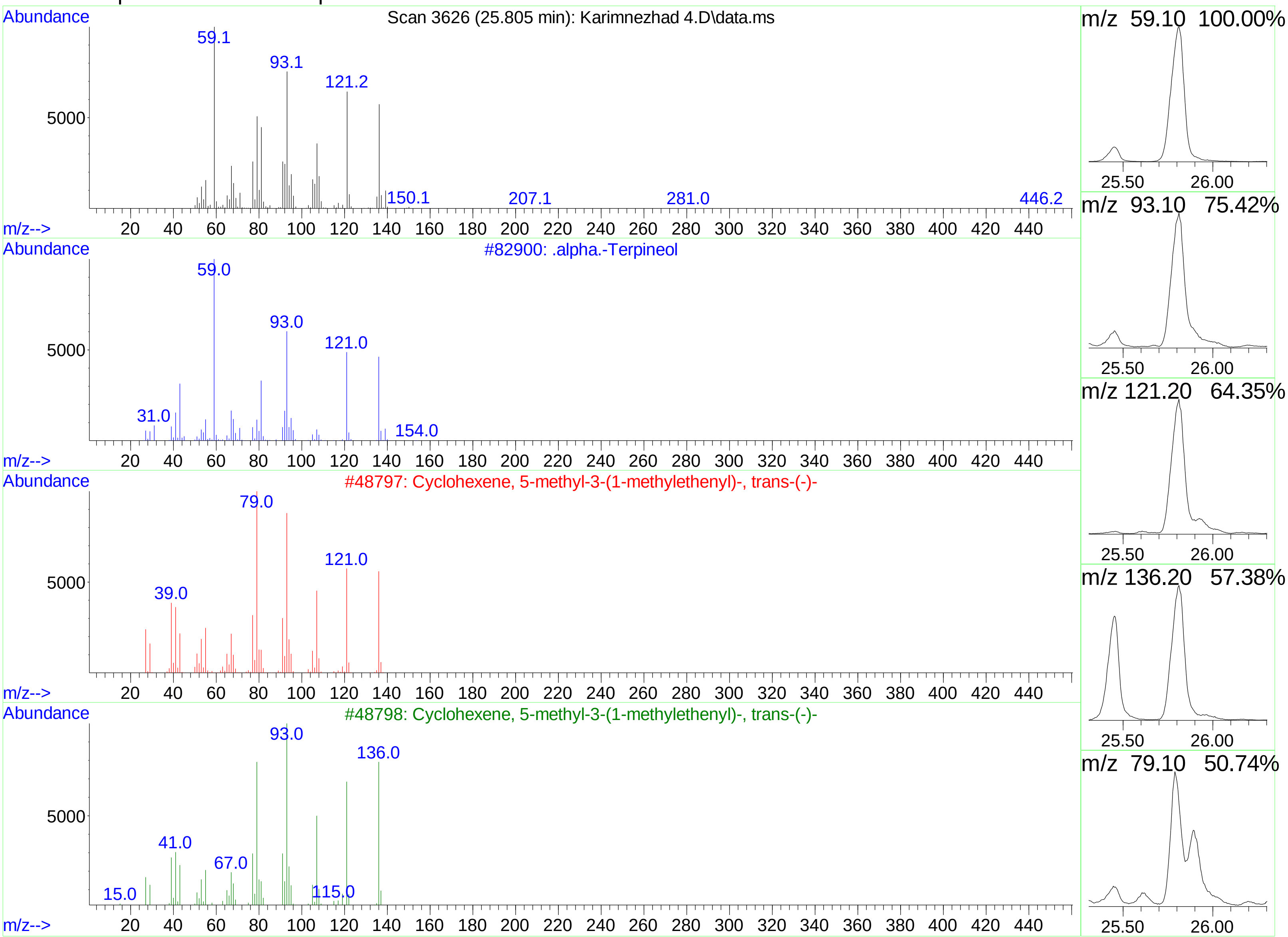

Data File: D:\msdchem\1\data\Karimnezhad 4.D
Sample : M14

Peak Number: 29 at 25.805 min Area: 118377787 Area % 0.52

The 3 best hits from each library.	Ref\#	CAS\#	Qual
D:\Database\W10N14.L			
1 .alpha.-Terpineol	82900	000098-55-5	90
2 Cyclohexene, 5-methyl-3-(1-methy...	48797	056816-08-1	60
3 Cyclohexene, 5-methyl-3-(1-methy...	48798	056816-08-1	60

Library Search Report - ChemStation Integrator

Unknown Spectrum based on Apex

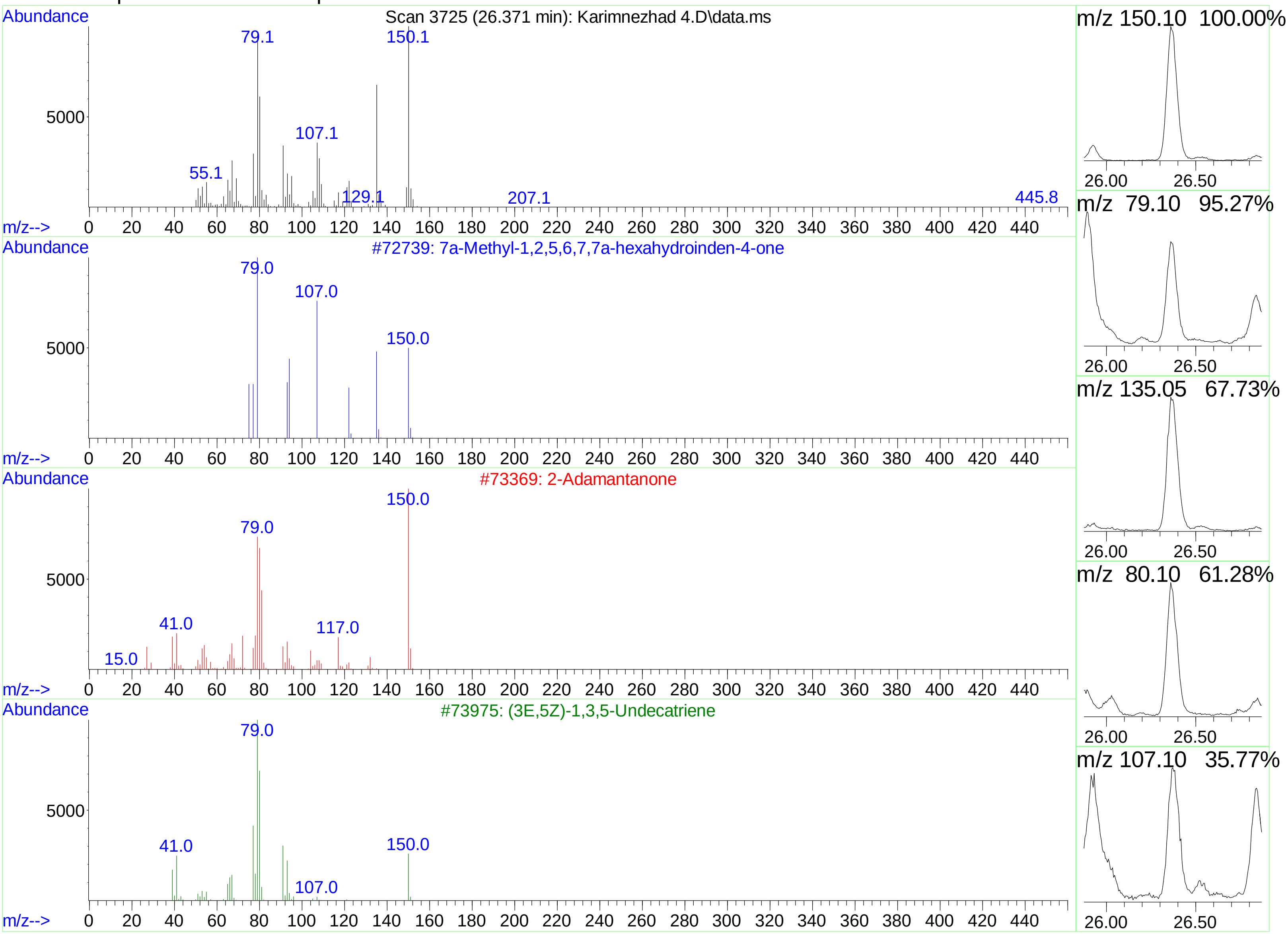

Data File: D:\msdchem\1\data\Karimnezhad 4.D
Sample : M14

Peak Number: 30 at 26.371 min Area: 22489960 Area % 0.10

The 3 best hits from each library.	Ref\#	CAS\#	Qual
D:\Database\W10N14.L			
1 7a-Methyl-1,2,5,6,7,7a-hexahydro...	72739	2000072-73-9	70
2 2-Adamantanone	73369	000700-58-3	58
3 (3E,5Z)-1,3,5-Undecatriene	73975	051447-08-6	58

Library Search Report - ChemStation Integrator

Unknown Spectrum based on Apex

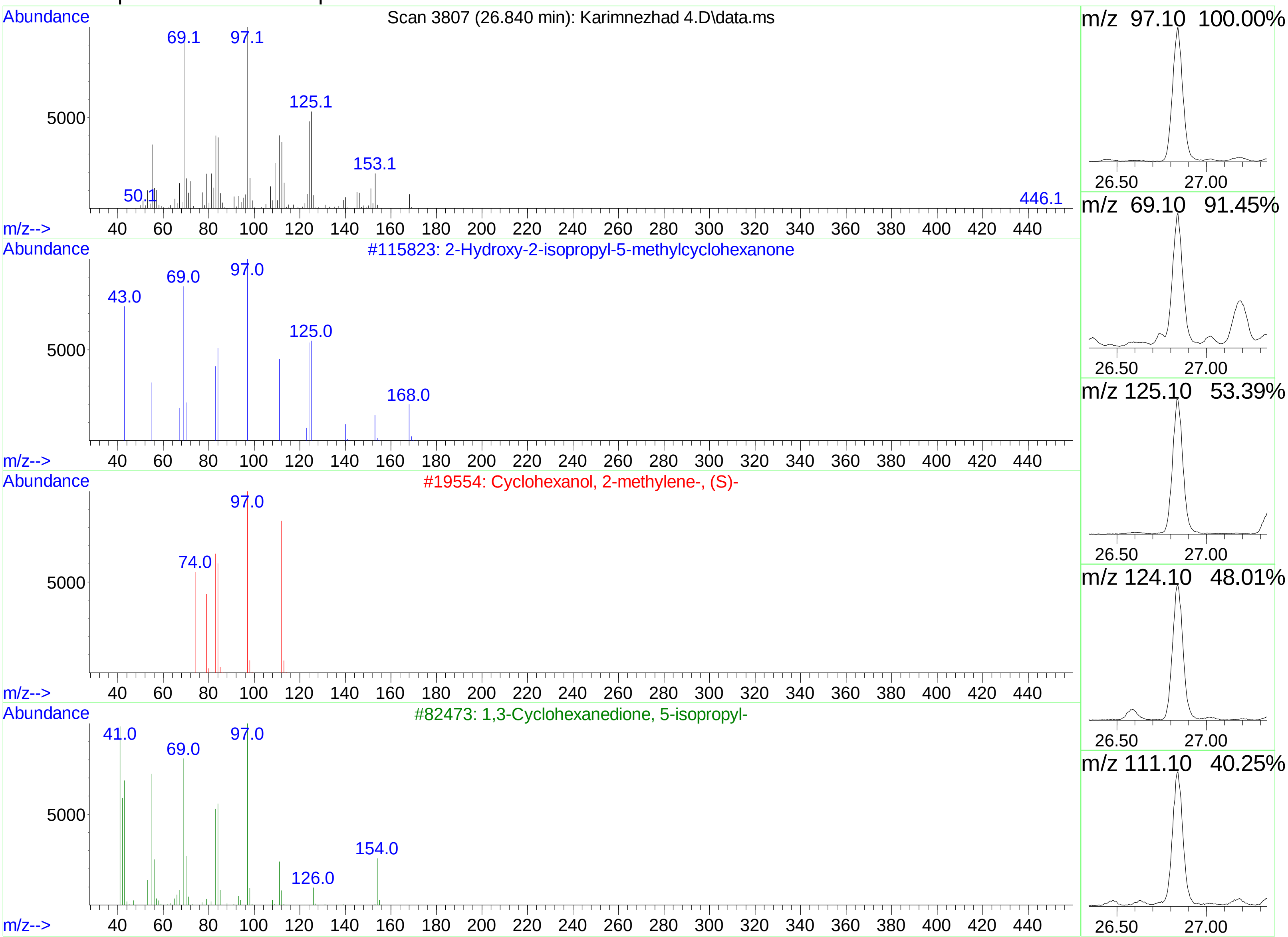

Data File: D:\msdchem\1\data\Karimnezhad 4.D
Sample : M14

Peak Number: 31 at 26.840 min Area: 105093677 Area % 0.46

The 3 best hits from each library.	Ref\#	CAS\#	Qual
D:\Database\W10N14.L			
1 2-Hydroxy-2-isopropyl-5-methylcy...	115823	000000-00-0	58
2 Cyclohexanol, 2-methylene-, (S)-	19554	061187-70-0	43
3 1,3-Cyclohexanedione, 5-isopropyl-	82473	018456-87-6	41

Unknown Spectrum based on Apex

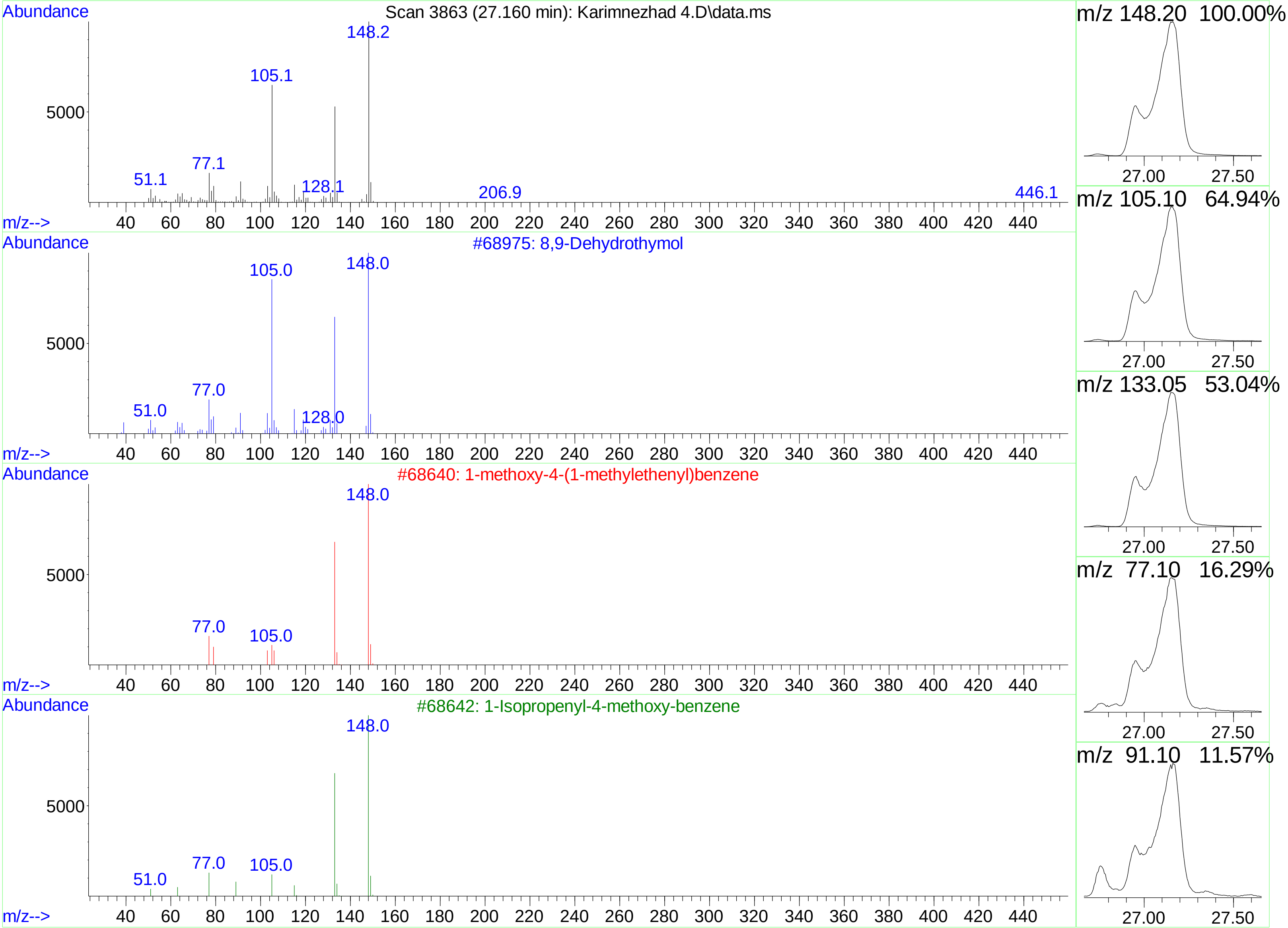

Data File: D:\msdchem\1\data\Karimnezhad 4.D
Sample : M14

Peak Number: 32 at 27.160 min Area: 654333818 Area % 2.86

The 3 best hits from each library. Ref\# CAS\# Qual

D:\Database\W10N14.L

		Ref\#	CAS\#	Qual
1	8,9-Dehydrothymol	68975	018612-99-2	95
2	1-methoxy-4-(1-methylethenyl)ben...	68640	2000068-64-0	81
3	1-Isopropenyl-4-methoxy-benzene	68642	2000068-64-2	72

Unknown Spectrum based on Apex

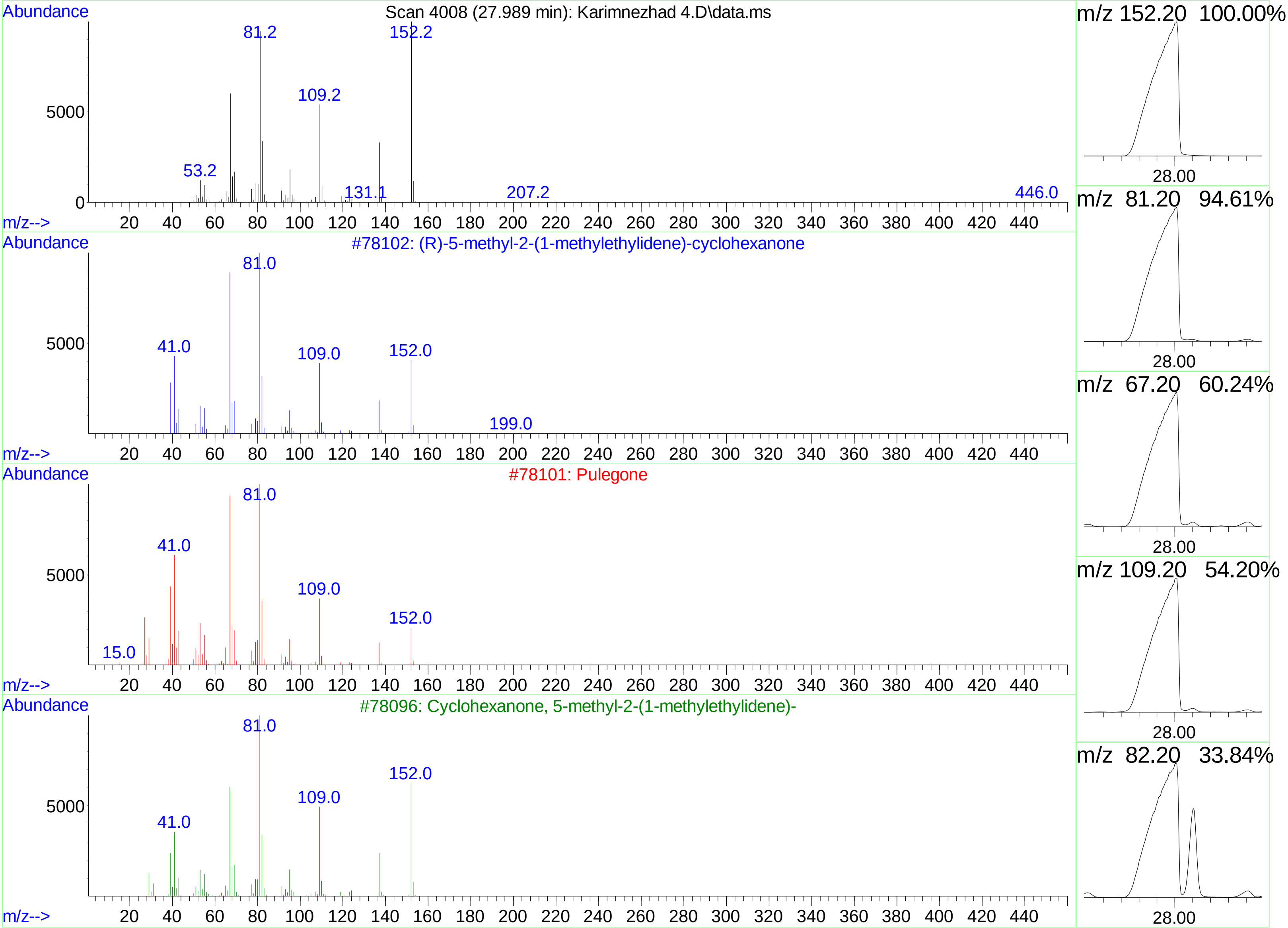

Data File: D:\msdchem\1\data\Karimnezhad 4.D
Sample : M14

Peak Number: 33 at 27.989 min Area: 2069303565 Area % 9.03

The 3 best hits from each library. Ref\# CAS\# Qual

D:\Database\W10N14.L

1	(R)-5-methyl-2-(1-methylethylide...	78102	000089-82-7	97
2	Pulegone	78101	000089-82-7	97
3	Cyclohexanone, 5-methyl-2-(1-met...	78096	015932-80-6	96

Unknown Spectrum based on Apex

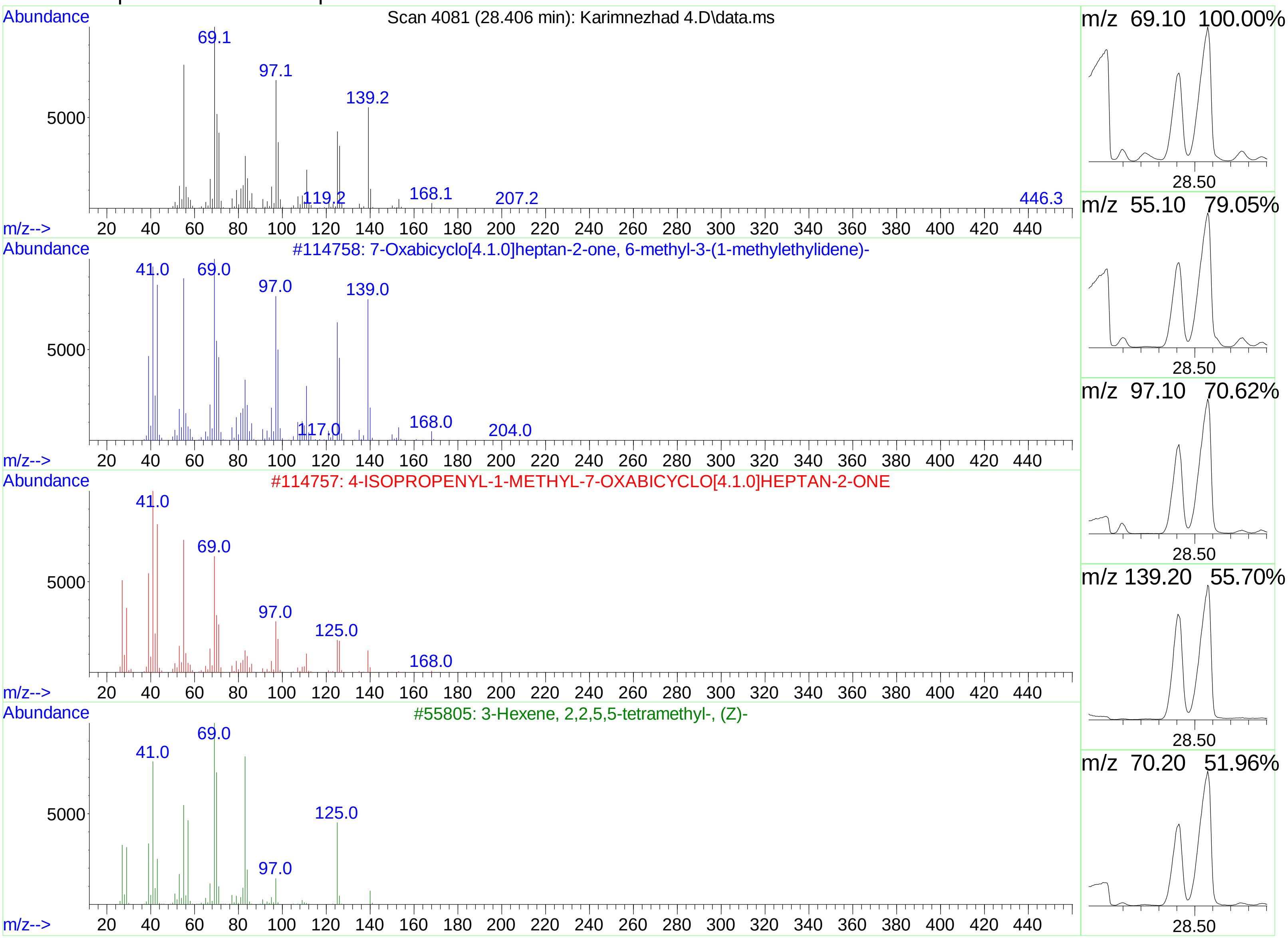

Data File: D:\msdchem\1\data\Karimnezhad 4.D
Sample : M14

Peak Number: 34 at 28.406 min Area: 142167273 Area % 0.62

The 3 best hits from each library. Ref\# CAS\# Qual

D:\Database\W10N14.L
1 7-Oxabicyclo[4.1.0]heptan-2-one,... 114758 035178-55-3 97
2 4-ISOPROPENYL-1-METHYL-7-OXABICY... 114757 035178-55-3 49
3 3-Hexene, 2,2,5,5-tetramethyl-, ... 55805 000692-47-7 46

Unknown Spectrum based on Apex

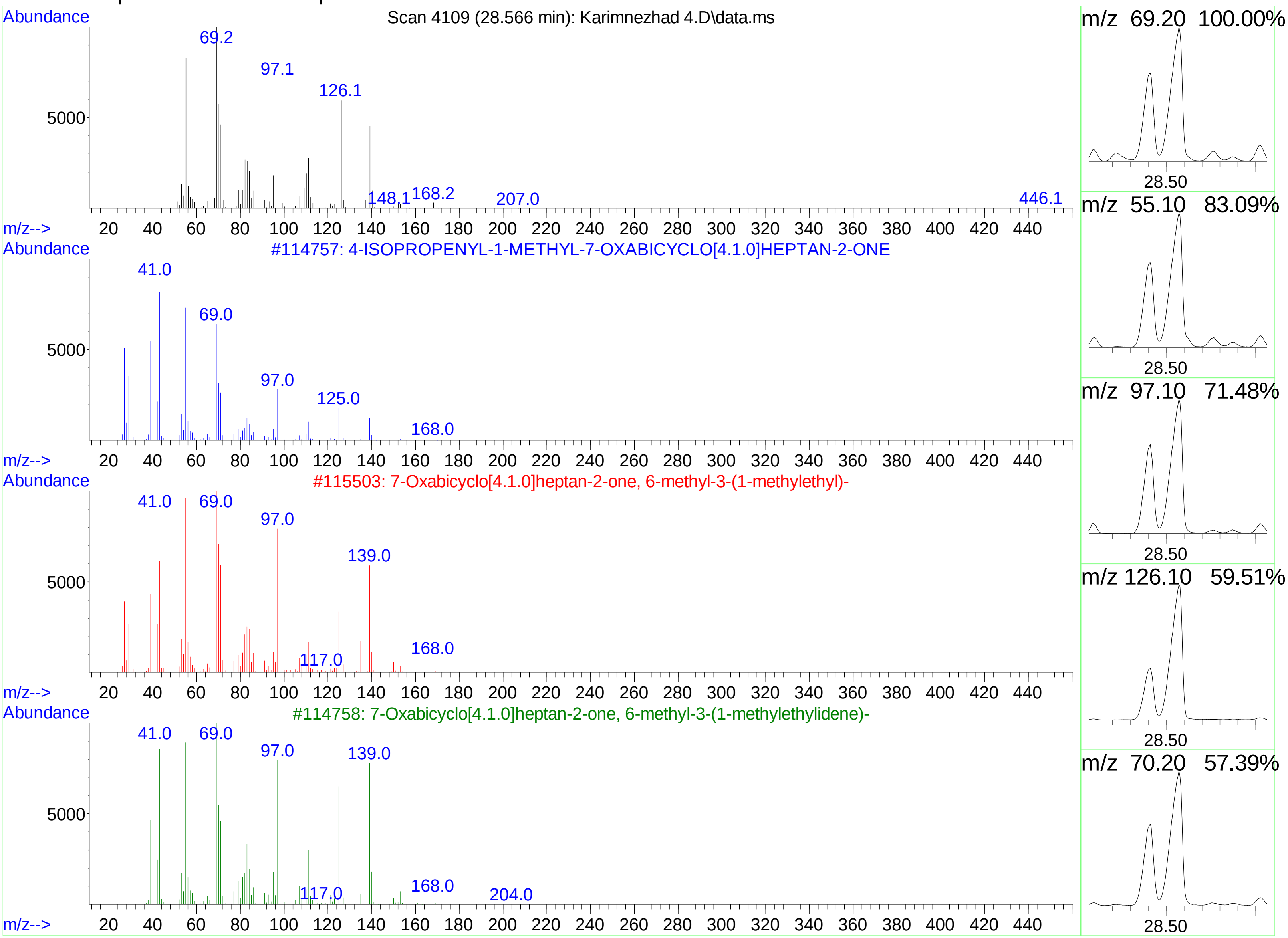

Data File: D:\msdchem\1\data\Karimnezhad 4.D
Sample : M14

Peak Number: 35 at 28.566 min Area: 316176189 Area % 1.38

The 3 best hits from each library.	Ref#	CAS#	Qual
D:\Database\W10N14.L			
1 4-ISOPROPENYL-1-METHYL-7-OXABICY...	114757	035178-55-3	83
2 7-Oxabicyclo[4.1.0]heptan-2-one,...	115503	005286-38-4	81
3 7-Oxabicyclo[4.1.0]heptan-2-one,...	114758	035178-55-3	80

Unknown Spectrum based on Apex

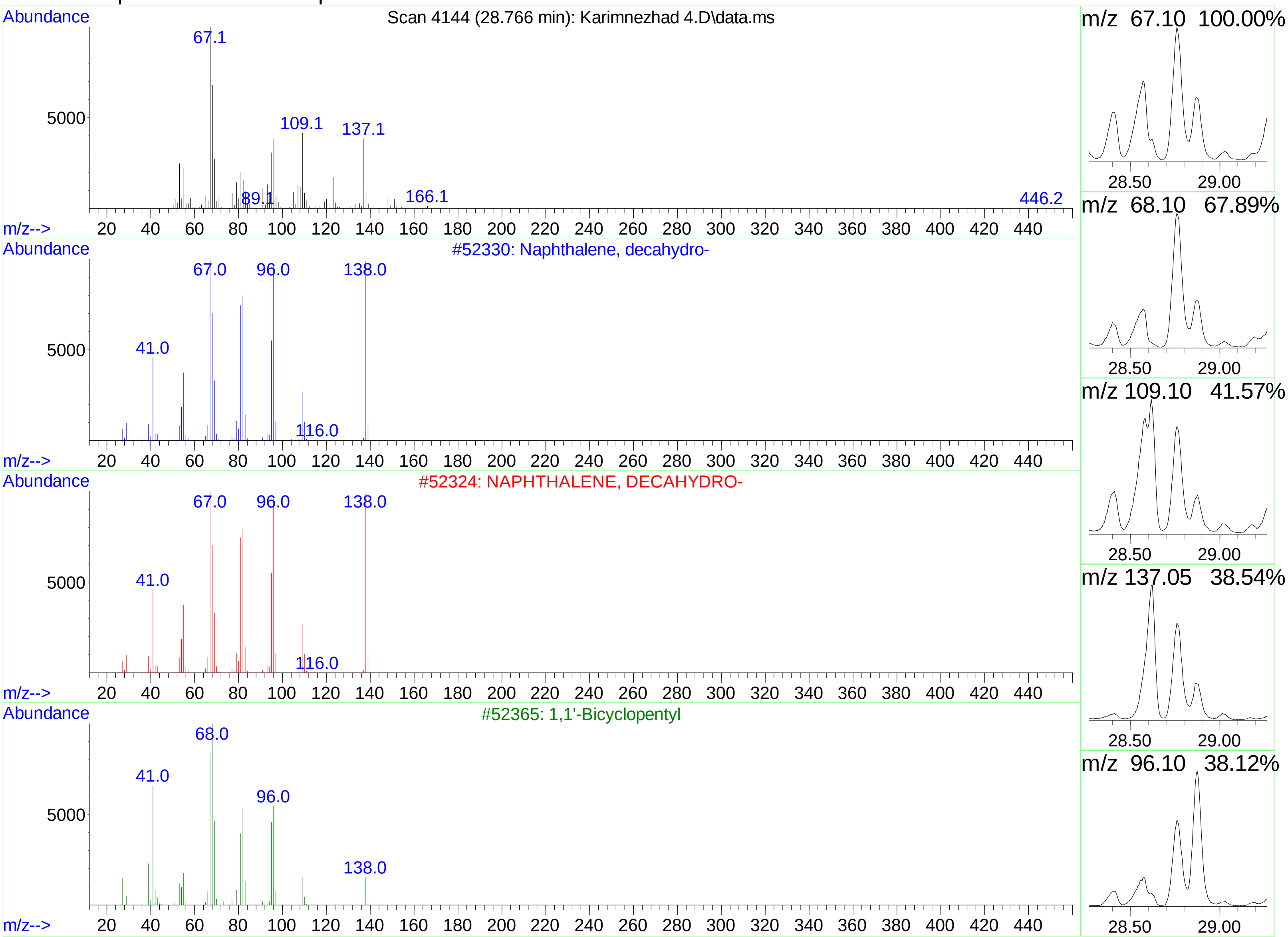

Data File: D:\msdchem\1\data\Karimnezhad 4.D
Sample : M14

Peak Number: 36 at 28.766 min Area: 50702936 Area % 0.22

The 3 best hits from each library.	Ref#	CAS#	Qual
D:\Database\W10N14.L			
1 Naphthalene, decahydro-	52330	000091-17-8	52
2 NAPHTHALENE, DECAHYDRO-	52324	000091-17-8	52
3 1,1'-Bicyclopentyl	52365	001636-39-1	50

Library Search Report - ChemStation Integrator

Unknown Spectrum based on Apex

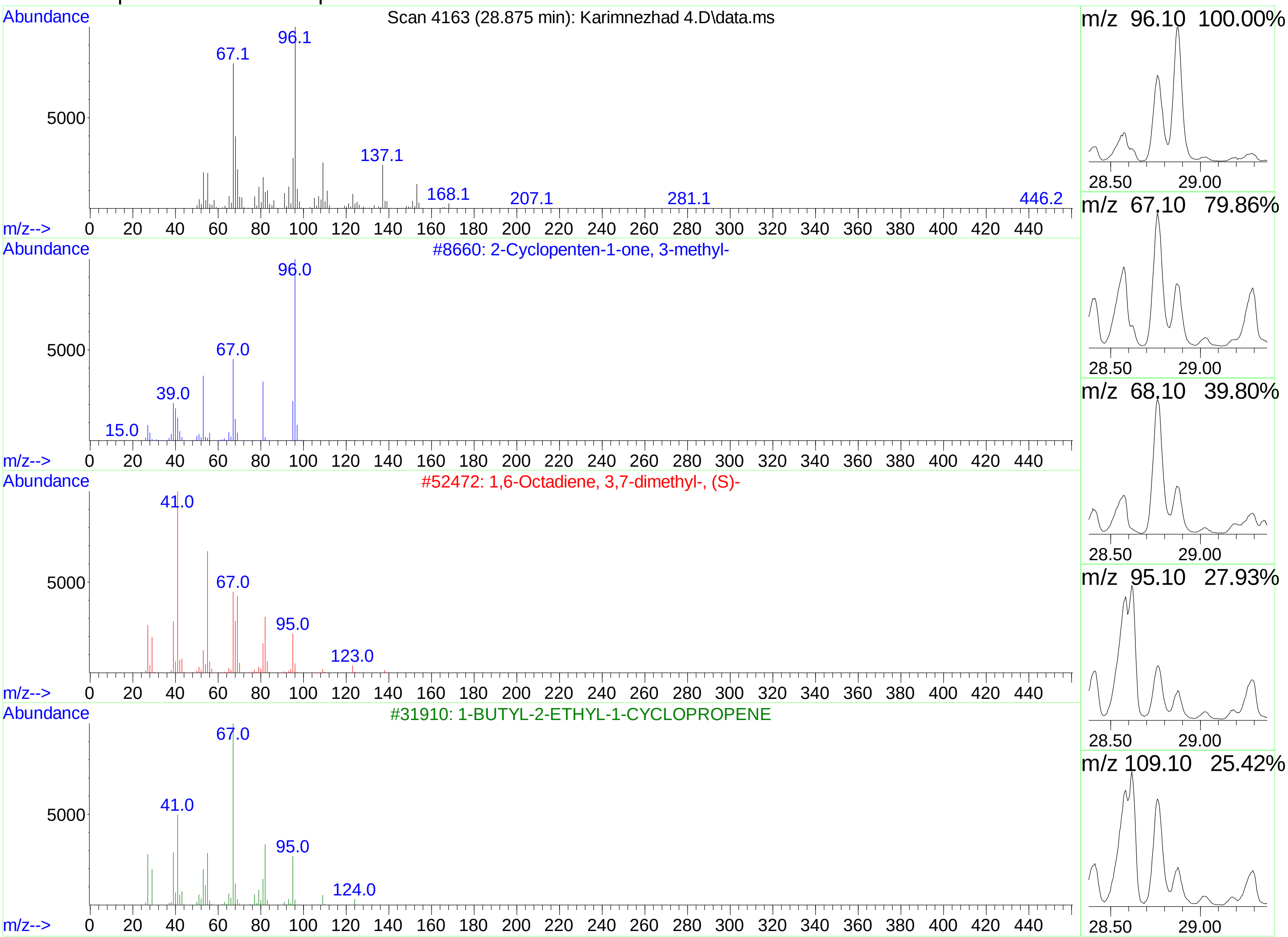

Data File: D:\msdchem\1\data\Karimnezhad 4.D
Sample : M14

Peak Number: 37 at 28.875 min Area: 25162026 Area % 0.11

The 3 best hits from each library. Ref\# CAS\# Qual

D:\Database\W10N14.L

		Ref\#	CAS\#	Qual
1	2-Cyclopenten-1-one, 3-methyl-	8660	002758-18-1	50
2	1,6-Octadiene, 3,7-dimethyl-, (S)-	52472	010281-55-7	43
3	1-BUTYL-2-ETHYL-1-CYCLOPROPENE	31910	050915-91-8	38

Unknown Spectrum based on Apex

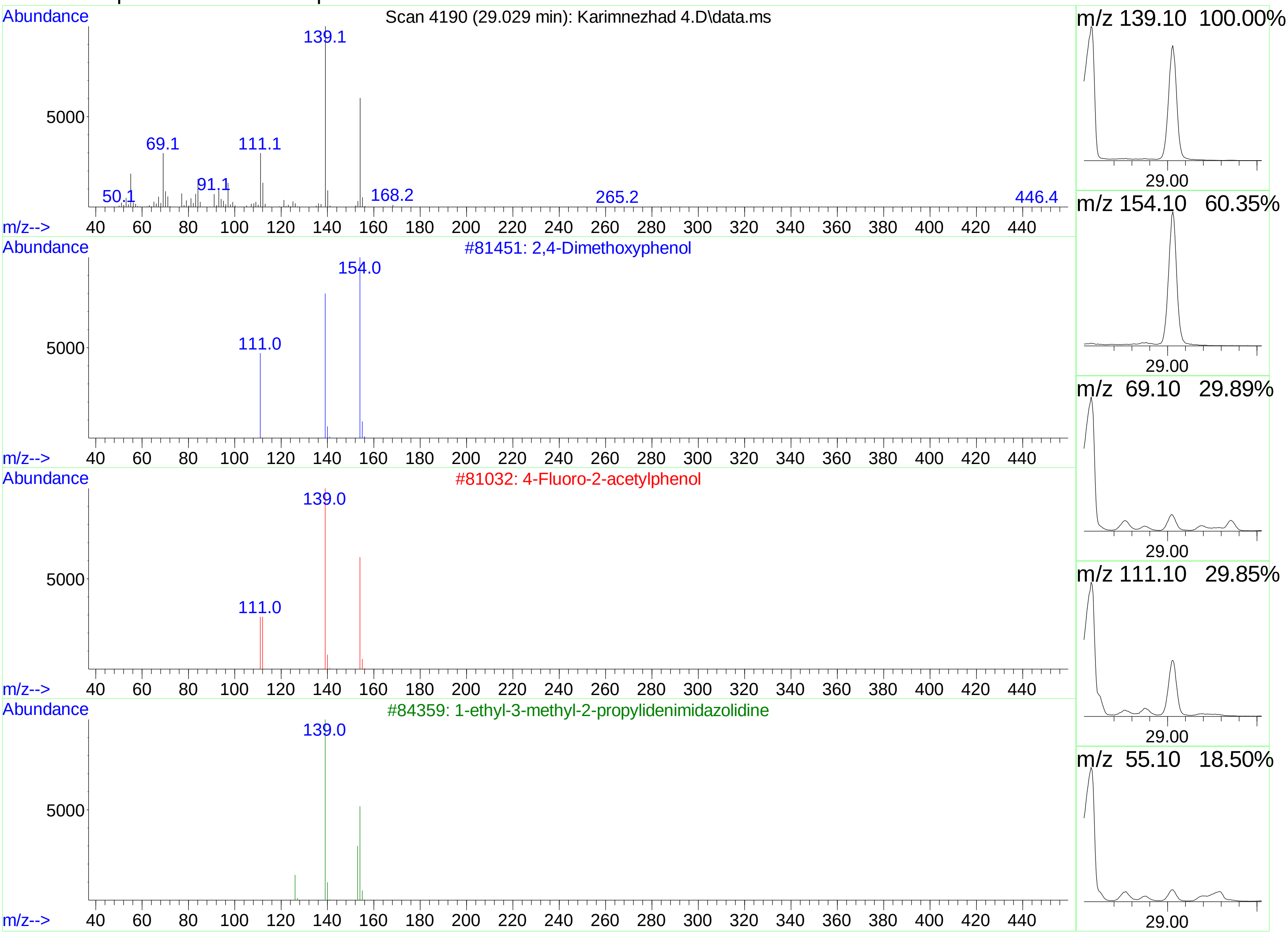

Data File: D:\msdchem\1\data\Karimnezhad 4.D
Sample : M14

Peak Number: 38 at 29.029 min Area: 39032289 Area % 0.17

The 3 best hits from each library.	Ref#	CAS#	Qual
D:\Database\W10N14.L			
1 2,4-Dimethoxyphenol	81451	013330-65-9	86
2 4-Fluoro-2-acetylphenol	81032	000394-32-1	86
3 1-ethyl-3-methyl-2-propylidenimi...	84359	109153-29-9	83

Unknown Spectrum based on Apex

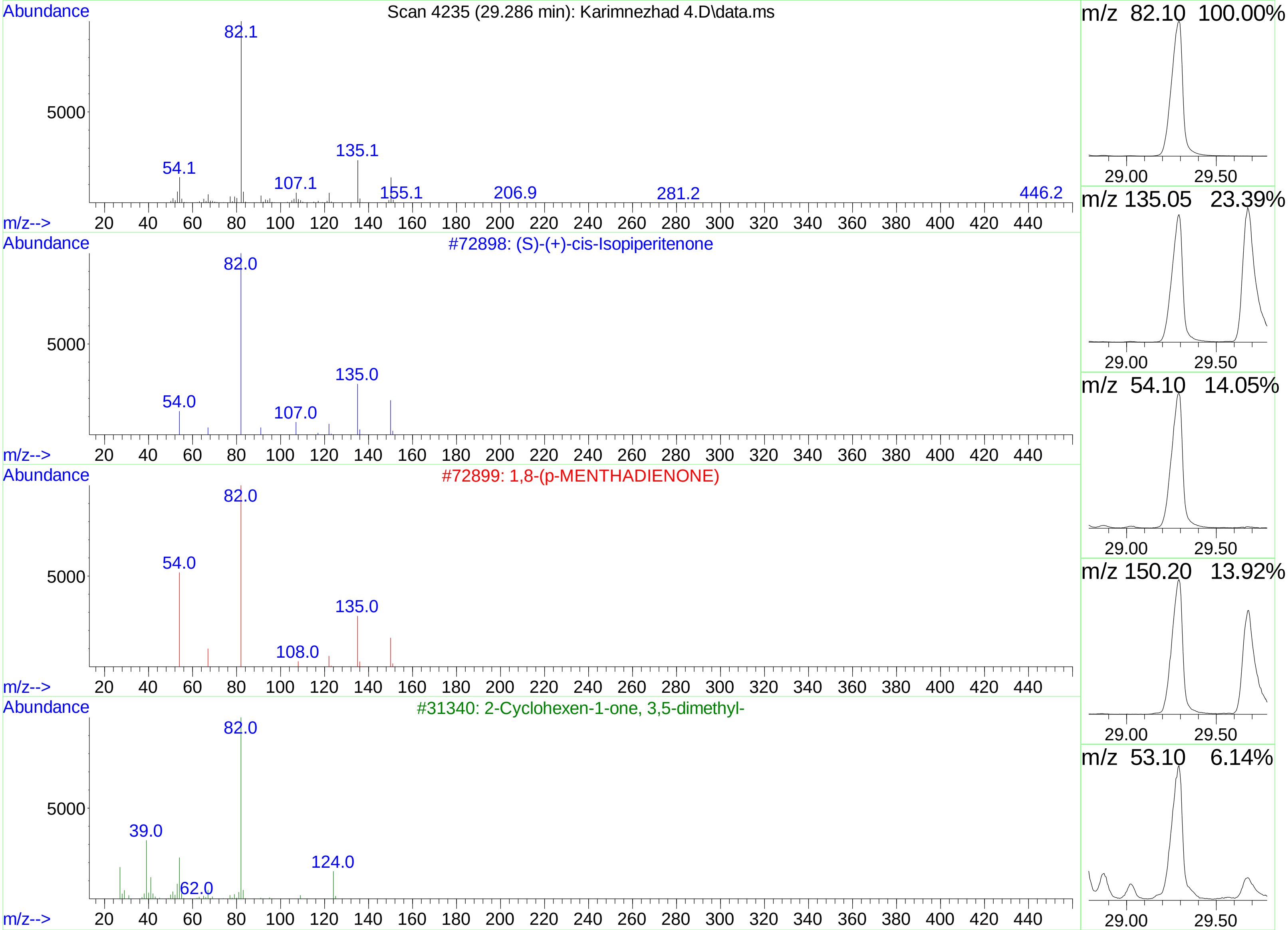

Data File: D:\msdchem\1\data\Karimnezhad 4.D
Sample : M14

Peak Number: 39 at 29.286 min Area: 210888958 Area % 0.92

The 3 best hits from each library.	Ref#	CAS#	Qual
D:\Database\W10N14.L			
1 (S)-(+)-cis-Isopiperitenone	72898	2000072-89-8	91
2 1,8-(p-MENTHADIENONE)	72899	2000072-89-9	72
3 2-Cyclohexen-1-one, 3,5-dimethyl-	31340	001123-09-7	50

Library Search Report - ChemStation Integrator

Unknown Spectrum based on Apex

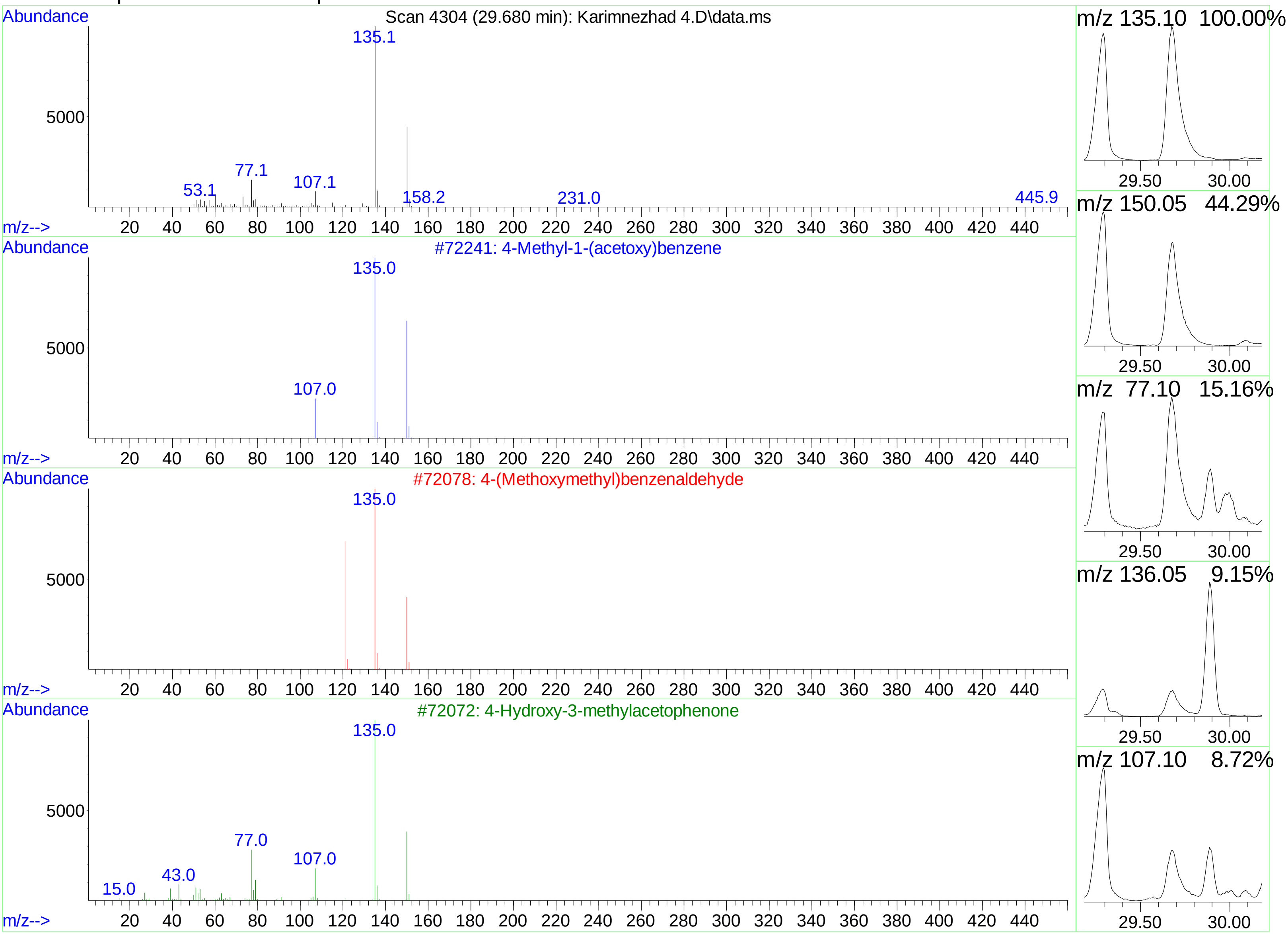

Data File: D:\msdchem\1\data\Karimnezhad 4.D
Sample : M14

Peak Number: 40 at 29.680 min Area: 61968837 Area % 0.27

The 3 best hits from each library.	Ref\#	CAS\#	Qual
D:\Database\W10N14.L			
1 4-Methyl-1-(acetoxy)benzene	72241	000140-39-6	90
2 4-(Methoxymethyl)benzenaldehyde	72078	2000072-07-8	90
3 4-Hydroxy-3-methylacetophenone	72072	000876-02-8	87

Unknown Spectrum based on Apex

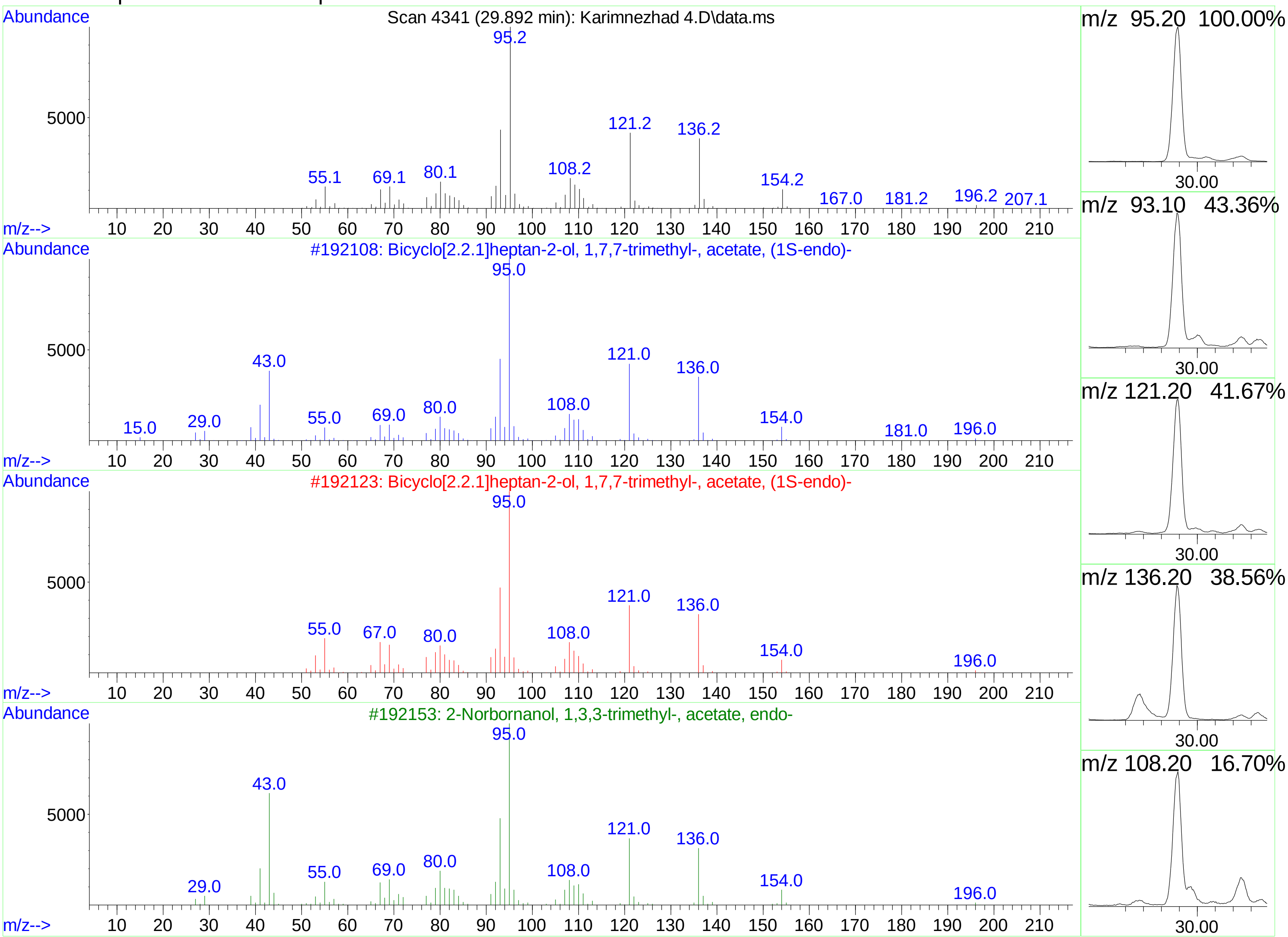

Data File: D:\msdchem\1\data\Karimnezhad 4.D
Sample : M14

Peak Number: 41 at 29.892 min Area: 145831474 Area % 0.64

The 3 best hits from each library. Ref\# CAS\# Qual

D:\Database\W10N14.L

		Ref\#	CAS\#	Qual
1	Bicyclo[2.2.1]heptan-2-ol, 1,7,7...	192108	005655-61-8	99
2	Bicyclo[2.2.1]heptan-2-ol, 1,7,7...	192123	005655-61-8	99
3	2-Norbornanol, 1,3,3-trimethyl-,...	192153	004057-31-2	98

Unknown Spectrum based on Apex

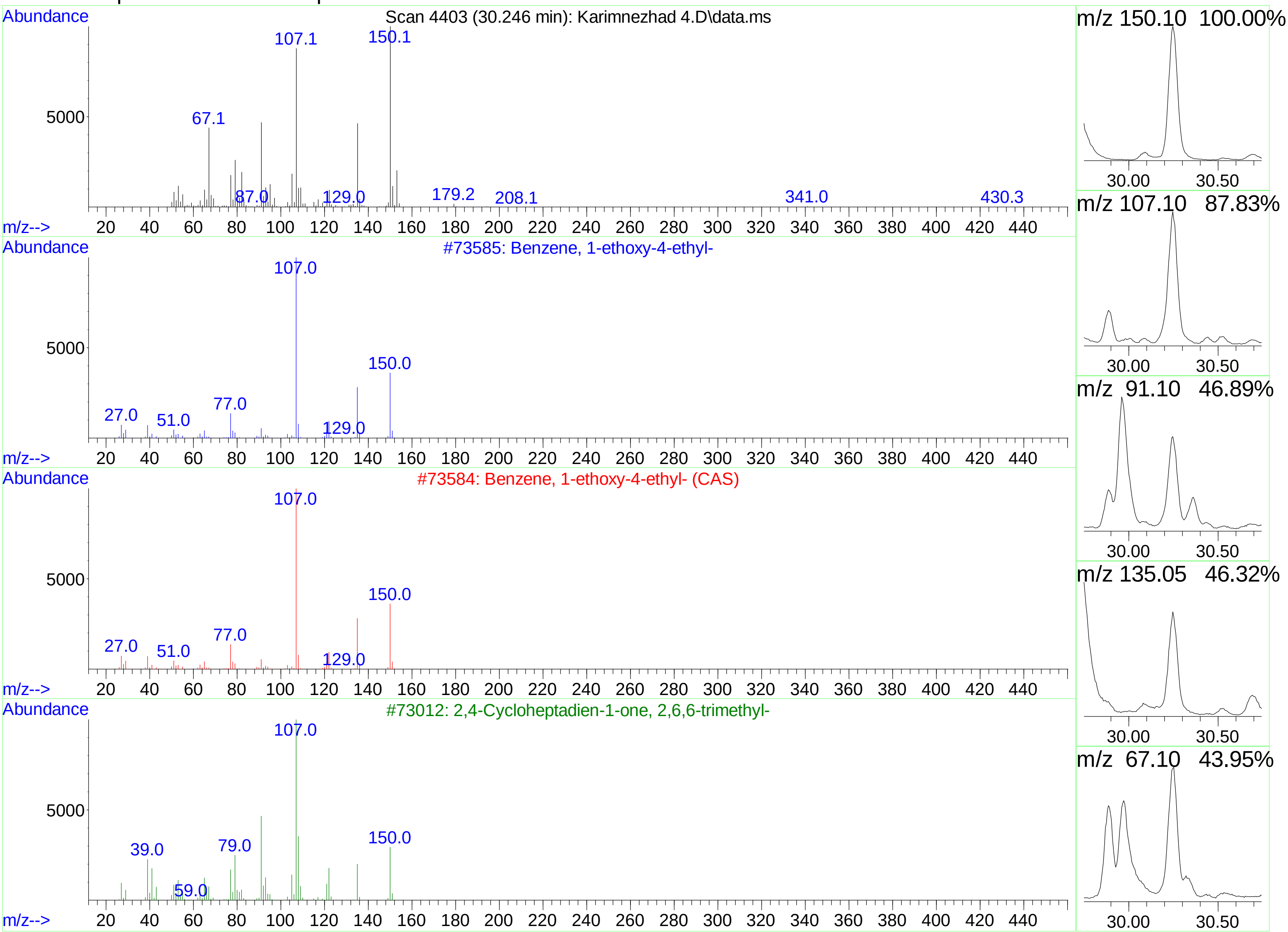

Data File: D:\msdchem\1\data\Karimnezhad 4.D
Sample : M14

Peak Number: 42 at 30.246 min Area: 65469679 Area % 0.29

The 3 best hits from each library. Ref\# CAS\# Qual

D:\Database\W10N14.L

1	Benzene, 1-ethoxy-4-ethyl-	73585	001585-06-4	89
2	Benzene, 1-ethoxy-4-ethyl- (CAS)	73584	001585-06-4	89
3	2,4-Cycloheptadien-1-one, 2,6,6-...	73012	000503-93-5	86

Unknown Spectrum based on Apex

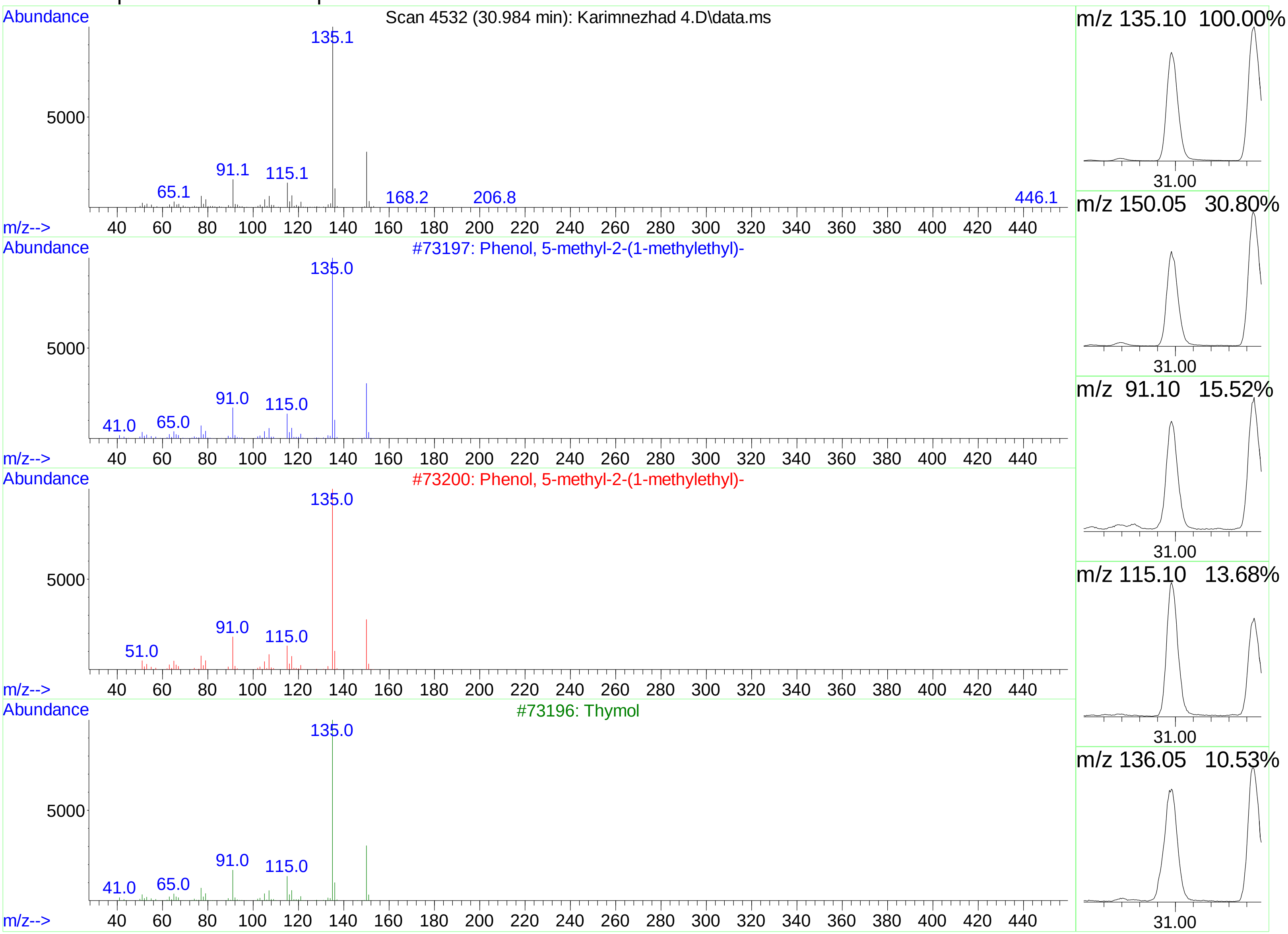

Data File: D:\msdchem\1\data\Karimnezhad 4.D
Sample : M14

Peak Number: 43 at 30.984 min Area: 83931183 Area % 0.37

The 3 best hits from each library.	Ref\#	CAS\#	Qual
D:\Database\W10N14.L			
1 Phenol, 5-methyl-2-(1-methylethyl)-	73197	000089-83-8	95
2 Phenol, 5-methyl-2-(1-methylethyl)-	73200	000089-83-8	95
3 Thymol	73196	000089-83-8	95

Unknown Spectrum based on Apex

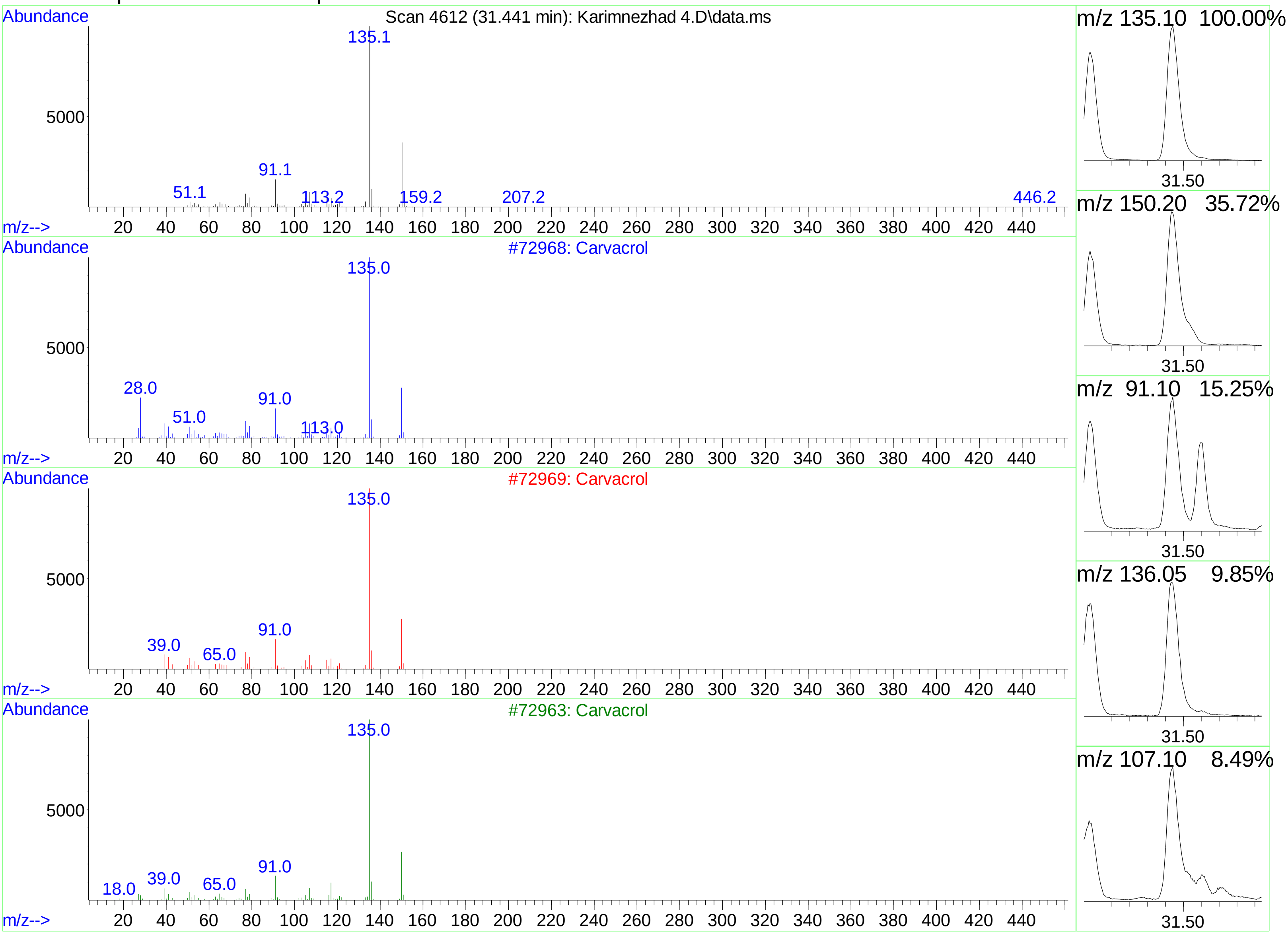

Data File: D:\msdchem\1\data\Karimnezhad 4.D
Sample : M14

Peak Number: 44 at 31.441 min Area: 107829395 Area % 0.47

The 3 best hits from each library.	Ref#	CAS#	Qual
D:\Database\W10N14.L			
1 Carvacrol	72968	000499-75-2	94
2 Carvacrol	72969	000499-75-2	94
3 Carvacrol	72963	000499-75-2	91

Unknown Spectrum based on Apex

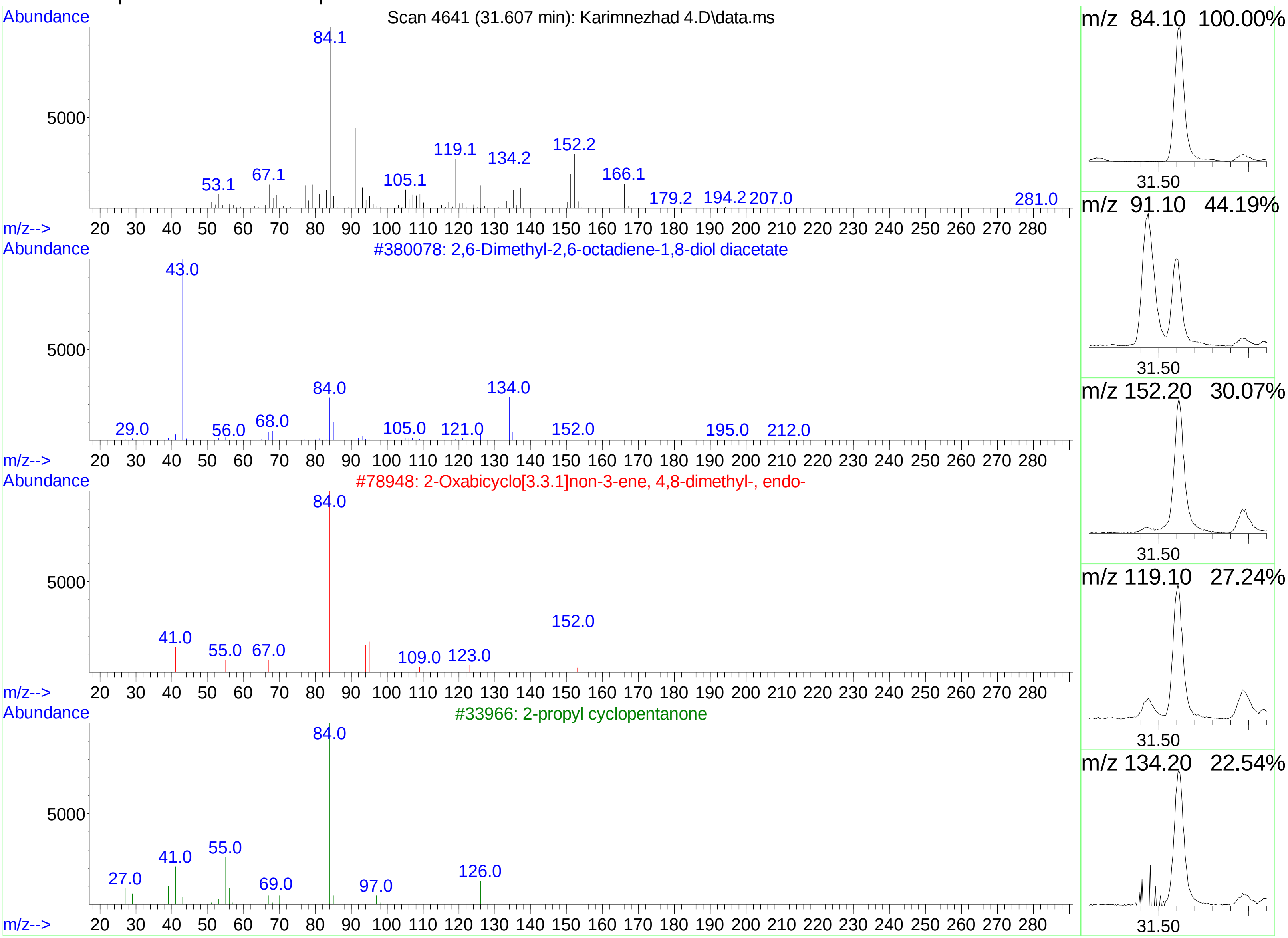

Data File: D:\msdchem\1\data\Karimnezhad 4.D
Sample : M14

Peak Number: 45 at 31.607 min Area: 62402226 Area % 0.27

The 3 best hits from each library.	Ref\#	CAS\#	Qual
D:\Database\W10N14.L			
1 2,6-Dimethyl-2,6-octadiene-1,8-d...	380078	036052-53-6	38
2 2-Oxabicyclo[3.3.1]non-3-ene, 4,...	78948	049576-27-4	38
3 2-propyl cyclopentanone	33966	2000033-96-6	35

Library Search Report - ChemStation Integrator

Unknown Spectrum based on Apex

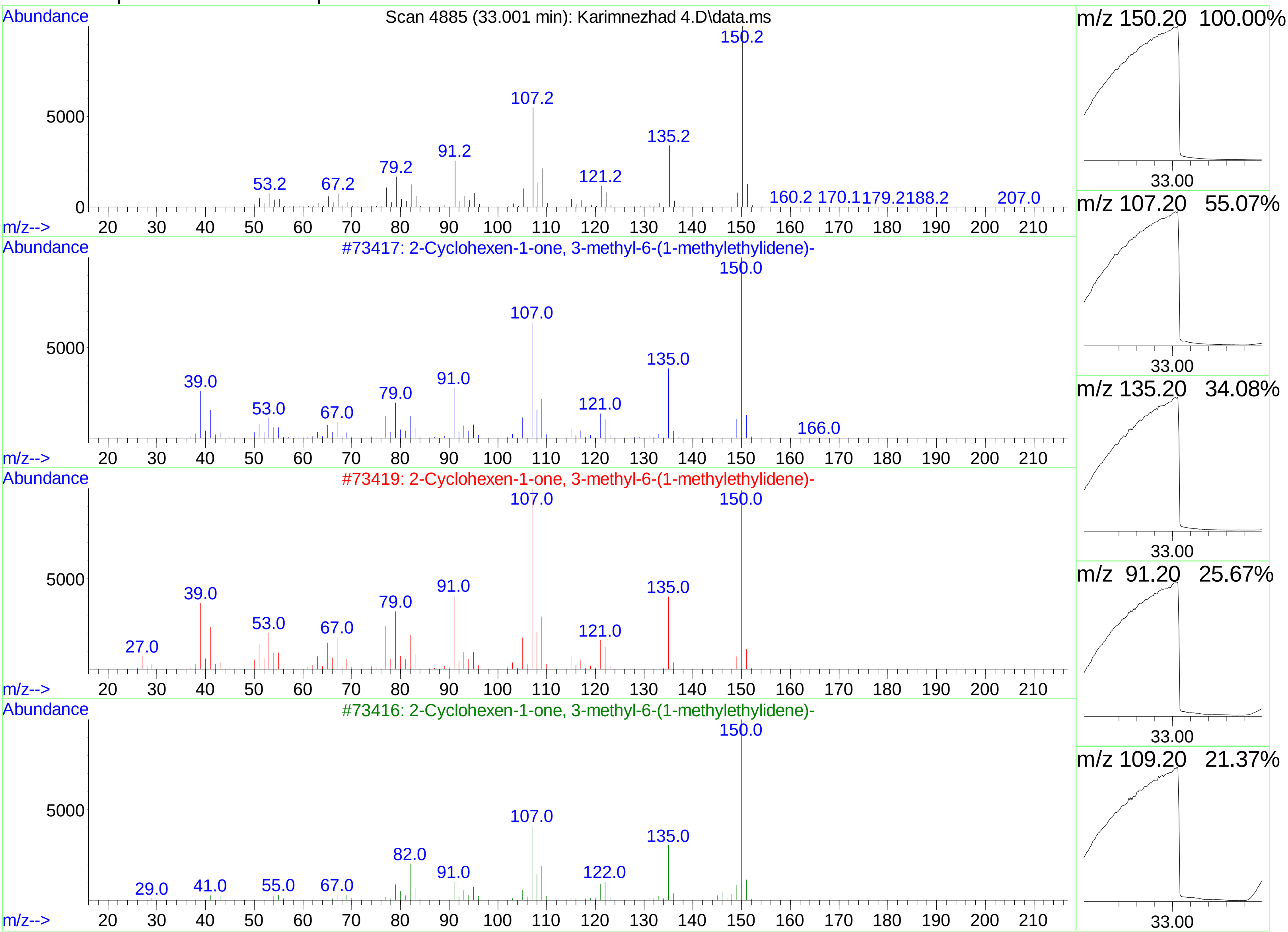

Data File: D:\msdchem\1\data\Karimnezhad 4.D
Sample : M14

Peak Number: 46 at 33.001 min Area: 7129367312 Area % 31.13

The 3 best hits from each library.	Ref\#	CAS\#	Qual
D:\Database\W10N14.L			
1 2-Cyclohexen-1-one, 3-methyl-6-(...	73417	000491-09-8	98
2 2-Cyclohexen-1-one, 3-methyl-6-(...	73419	000491-09-8	95
3 2-Cyclohexen-1-one, 3-methyl-6-(...	73416	000491-09-8	91

Unknown Spectrum based on Apex

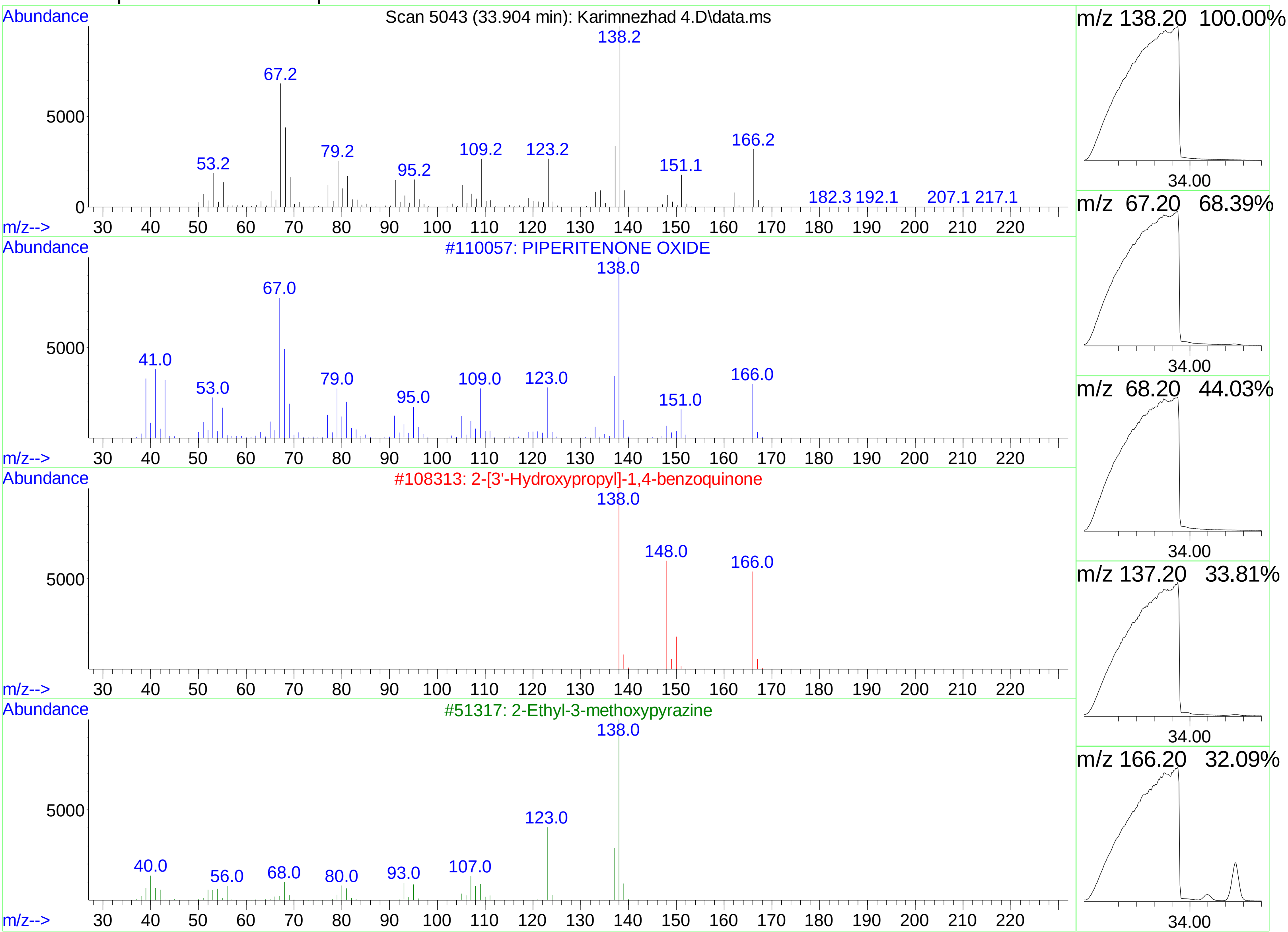

Data File: D:\msdchem\1\data\Karimnezhad 4.D
Sample : M14

Peak Number: 47 at 33.904 min Area: 4378441532 Area % 19.12

The 3 best hits from each library.	Ref#	CAS#	Qual
D:\Database\W10N14.L			
1 PIPERITENONE OXIDE	110057	003564-96-3	99
2 2-[3'-Hydroxypropyl]-1,4-benzoqu...	108313	2000108-31-3	86
3 2-Ethyl-3-methoxypyrazine	51317	025680-58-4	58

Library Search Report - ChemStation Integrator

Unknown Spectrum based on Apex

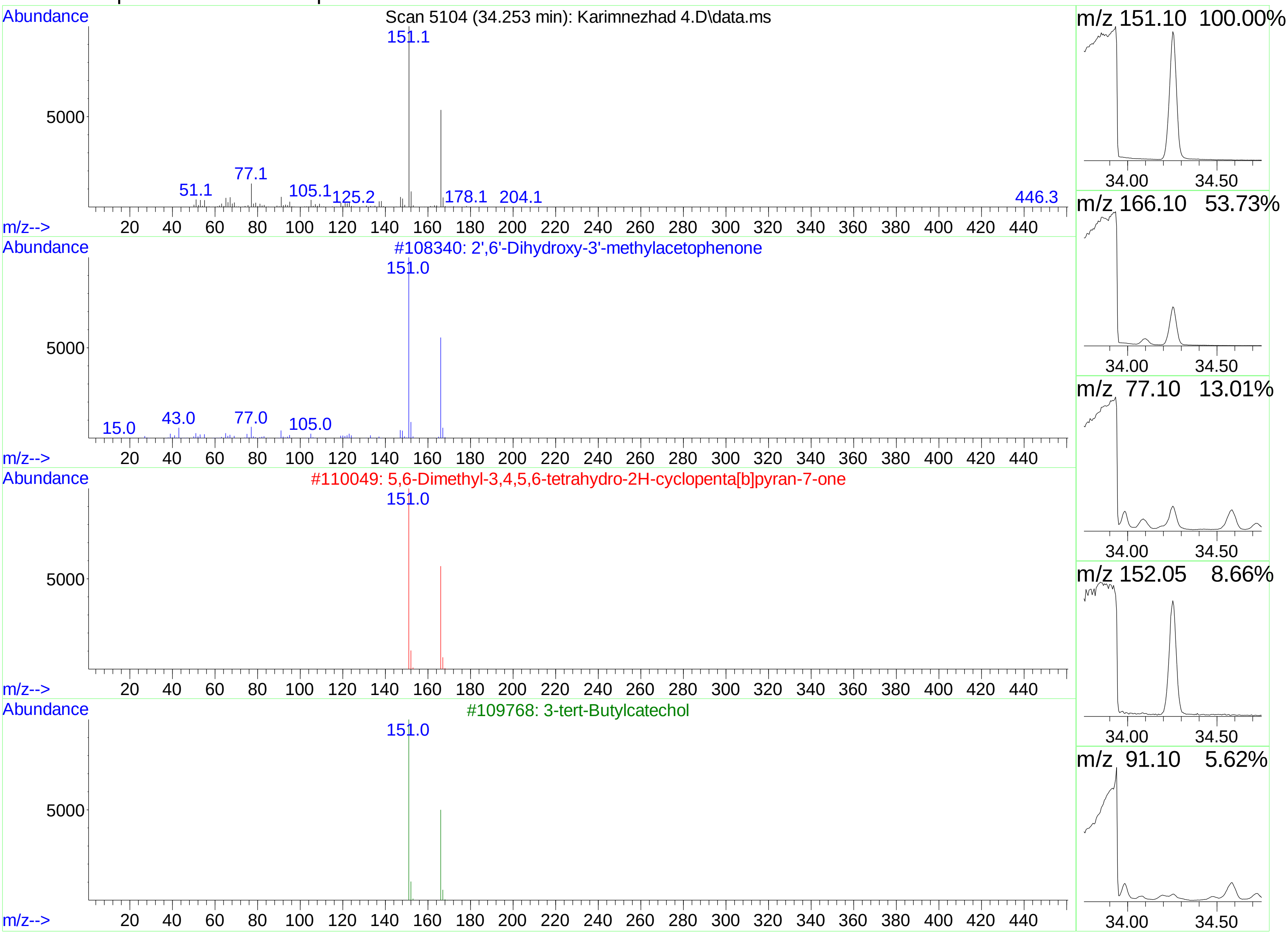

Data File: D:\msdchem\1\data\Karimnezhad 4.D
Sample : M14

Peak Number: 48 at 34.253 min Area: 76641552 Area % 0.33

The 3 best hits from each library.	Ref\#	CAS\#	Qual
D:\Database\W10N14.L			
1 2',6'-Dihydroxy-3'-methylacetoph...	108340	029183-78-6	87
2 5,6-Dimethyl-3,4,5,6-tetrahydro-...	110049	2000110-04-9	83
3 3-tert-Butylcatechol	109768	2000109-76-8	83

Unknown Spectrum based on Apex

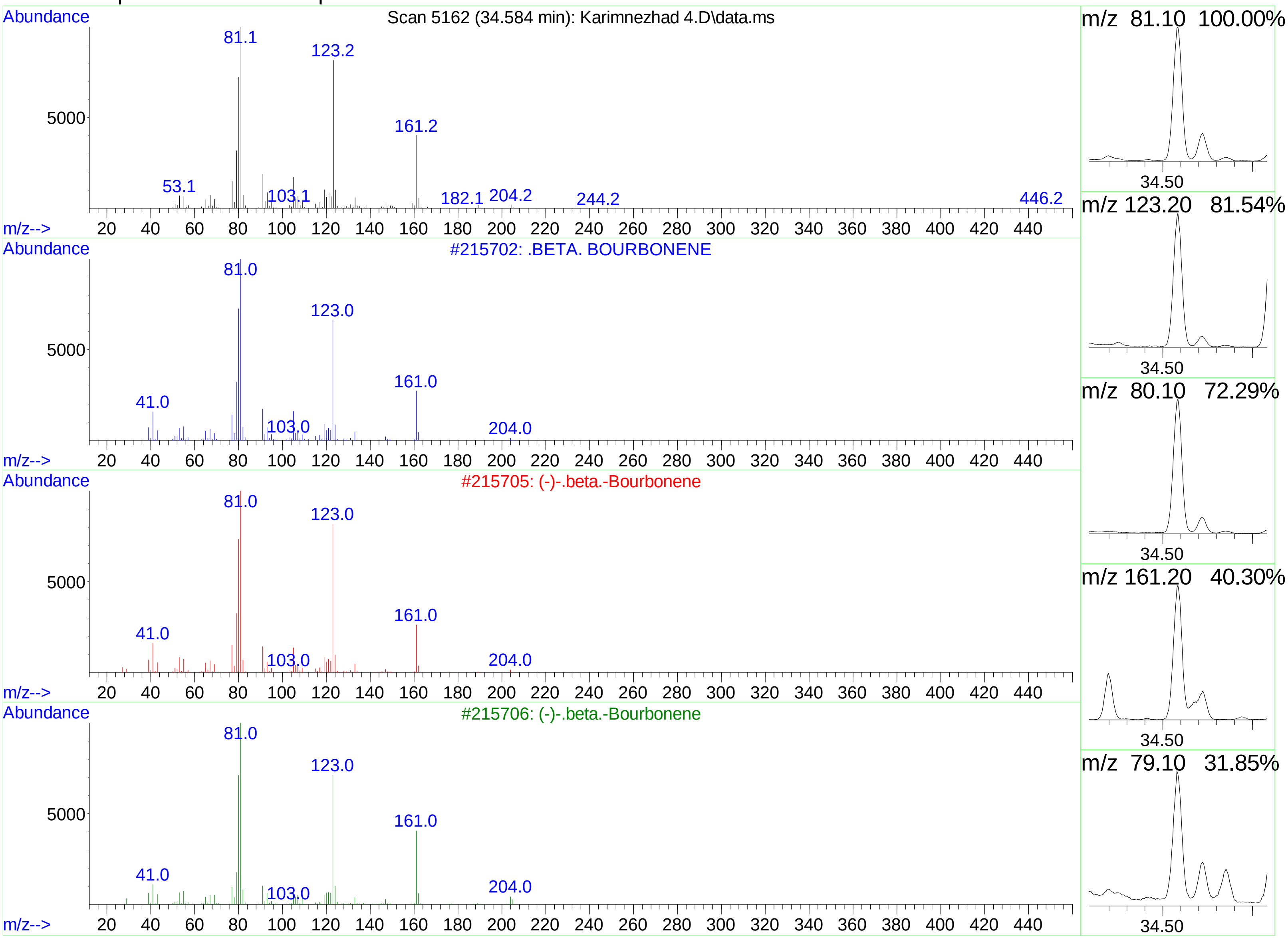

Data File: D:\msdchem\1\data\Karimnezhad 4.D
Sample : M14

Peak Number: 49 at 34.584 min Area: 102011731 Area % 0.45

The 3 best hits from each library.	Ref\#	CAS\#	Qual
D:\Database\W10N14.L			
1 .BETA. BOURBONENE	215702	005208-59-3	99
2 (-)-.beta.-Bourbonene	215705	005208-59-3	98
3 (-)-.beta.-Bourbonene	215706	005208-59-3	96

Unknown Spectrum based on Apex

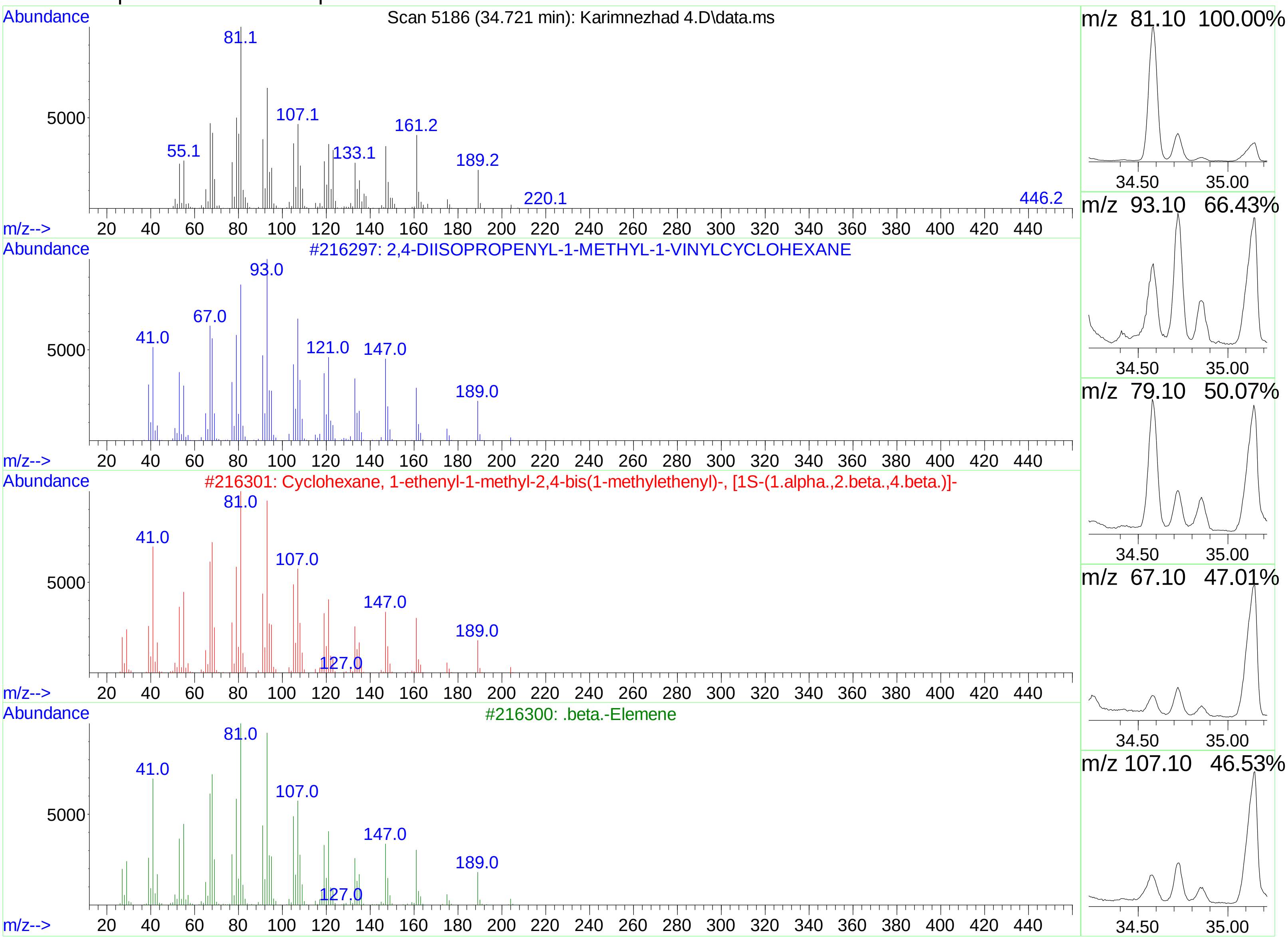

Data File: D:\msdchem\1\data\Karimnezhad 4.D
Sample : M14

Peak Number: 50 at 34.721 min Area: 34254198 Area % 0.15

The 3 best hits from each library. Ref\# CAS\# Qual

D:\Database\W10N14.L

1	2,4-DIISOPROPENYL-1-METHYL-1-VIN...	216297	000515-13-9	97
2	Cyclohexane, 1-ethenyl-1-methyl-...	216301	000515-13-9	93
3	.beta.-Elemene	216300	000515-13-9	93

Unknown Spectrum based on Apex

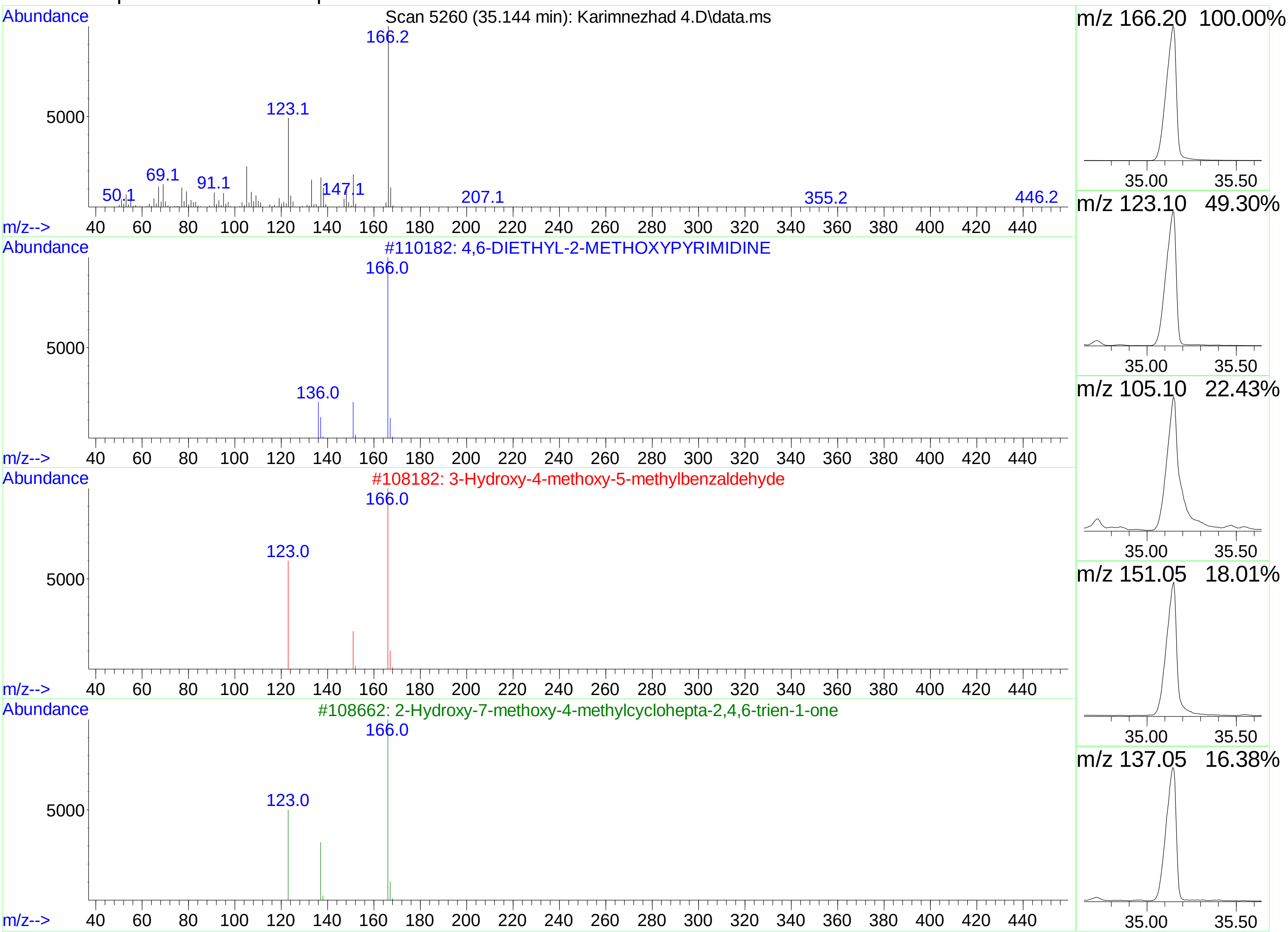

Data File: D:\msdchem\1\data\Karimnezhad 4.D
Sample : M14

Peak Number: 51 at 35.144 min Area: 356153711 Area % 1.55

The 3 best hits from each library.	Ref\#	CAS\#	Qual
D:\Database\W10N14.L			
1 4,6-DIETHYL-2-METHOXYPYRIMIDINE	110182	2000110-18-2	64
2 3-Hydroxy-4-methoxy-5-methylbenz...	108182	2000108-18-2	64
3 2-Hydroxy-7-methoxy-4-methylcycl...	108662	2000108-66-2	59

Unknown Spectrum based on Apex

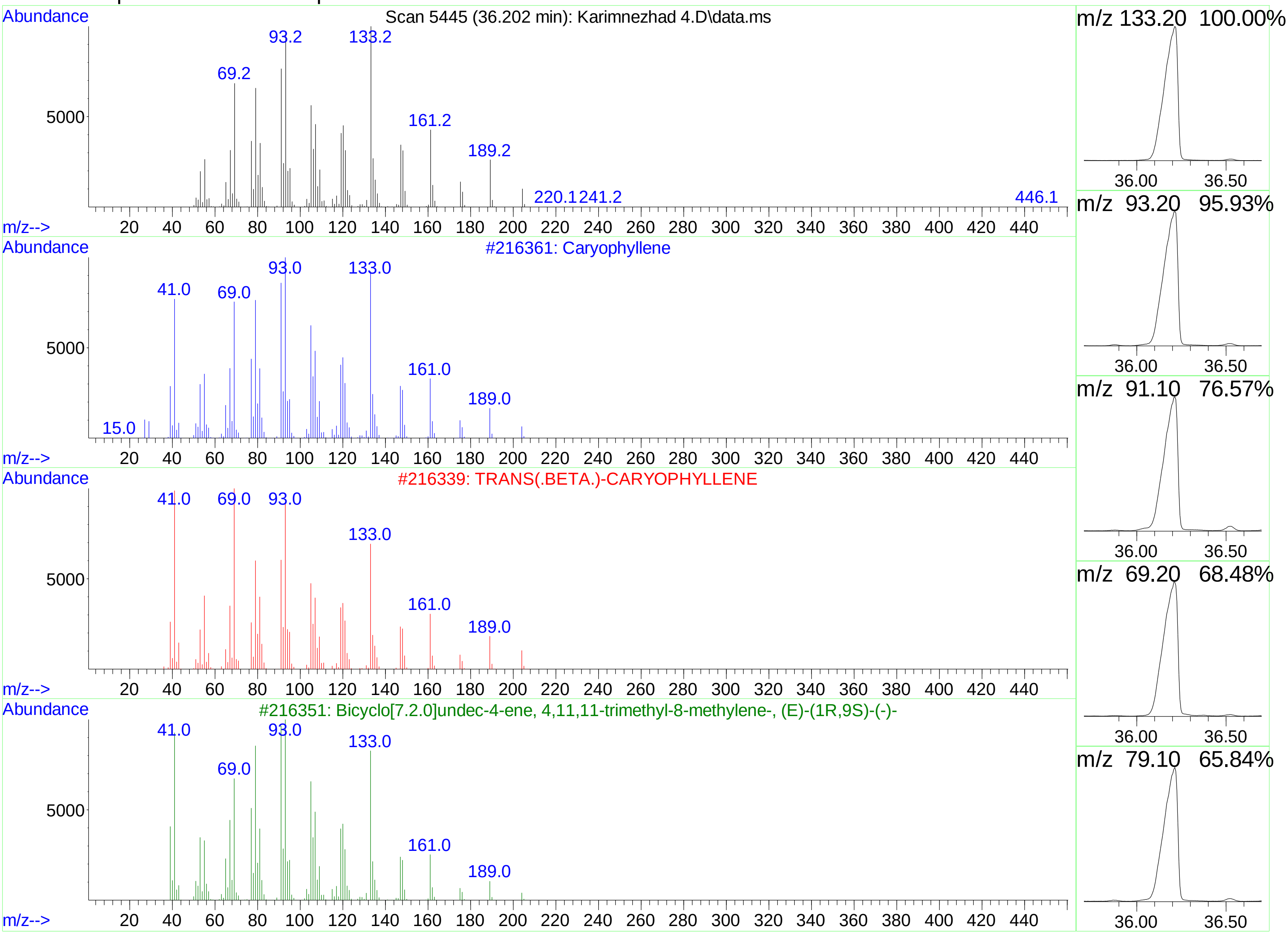

Data File: D:\msdchem\1\data\Karimnezhad 4.D
Sample : M14

Peak Number: 52 at 36.202 min Area: 872120227 Area % 3.81

The 3 best hits from each library.	Ref\#	CAS\#	Qual
D:\Database\W10N14.L			
1 Caryophyllene	216361	000087-44-5	99
2 TRANS(.BETA.)-CARYOPHYLLENE	216339	2000216-33-9	99
3 Bicyclo[7.2.0]undec-4-ene, 4,11,...	216351	000087-44-5	99

Unknown Spectrum based on Apex

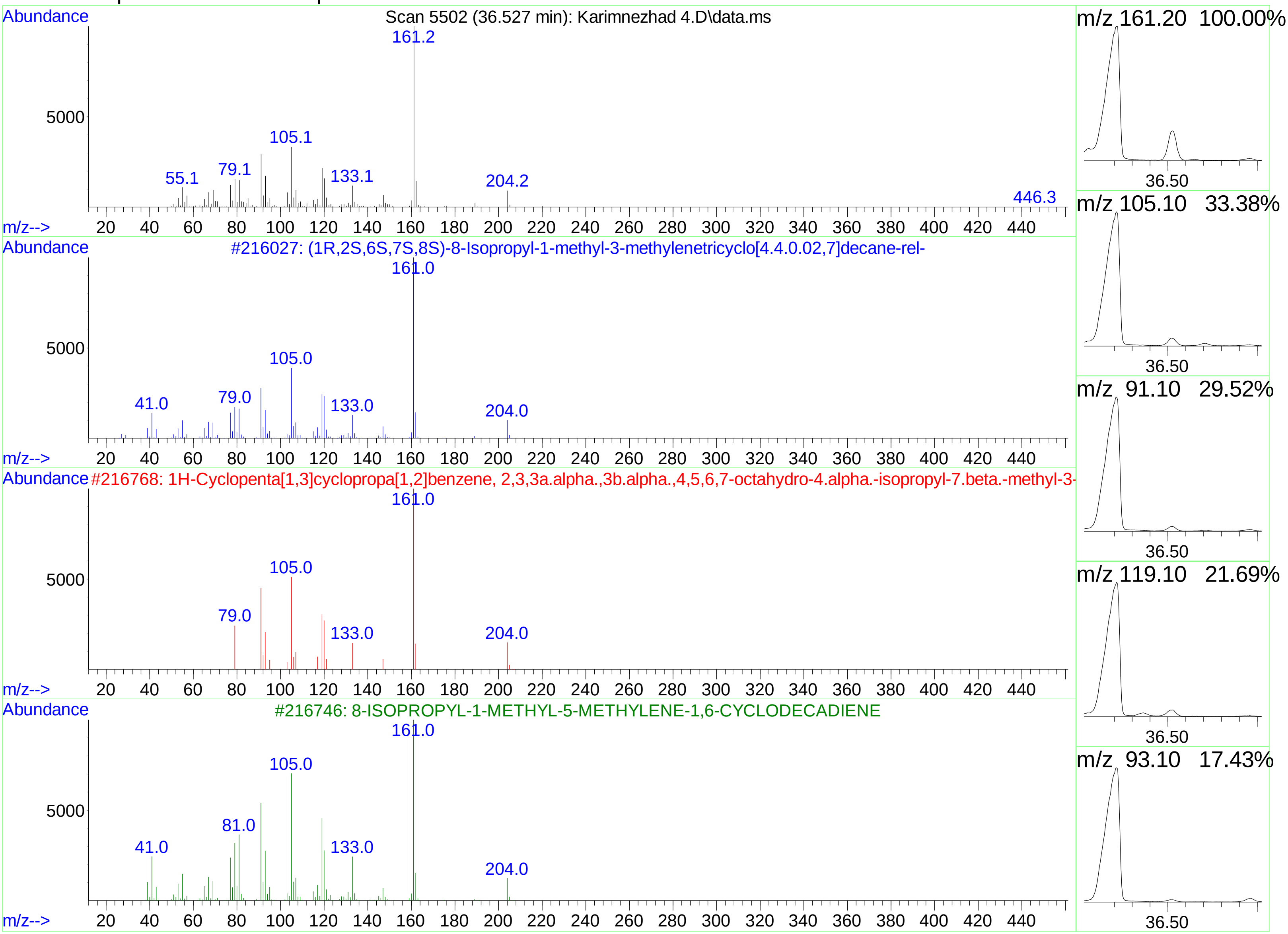

Data File: D:\msdchem\1\data\Karimnezhad 4.D
Sample : M14

Peak Number: 53 at 36.527 min Area: 17794718 Area % 0.08

The 3 best hits from each library. Ref\# CAS\# Qual

D:\Database\W10N14.L

		Ref#	CAS#	Qual
1	(1R,2S,6S,7S,8S)-8-Isopropyl-1-m...	216027	018252-44-3	98
2	1H-Cyclopenta[1,3]cyclopropa[1,2...	216768	013744-15-5	98
3	8-ISOPROPYL-1-METHYL-5-METHYLENE...	216746	023986-74-5	97

Unknown Spectrum based on Apex

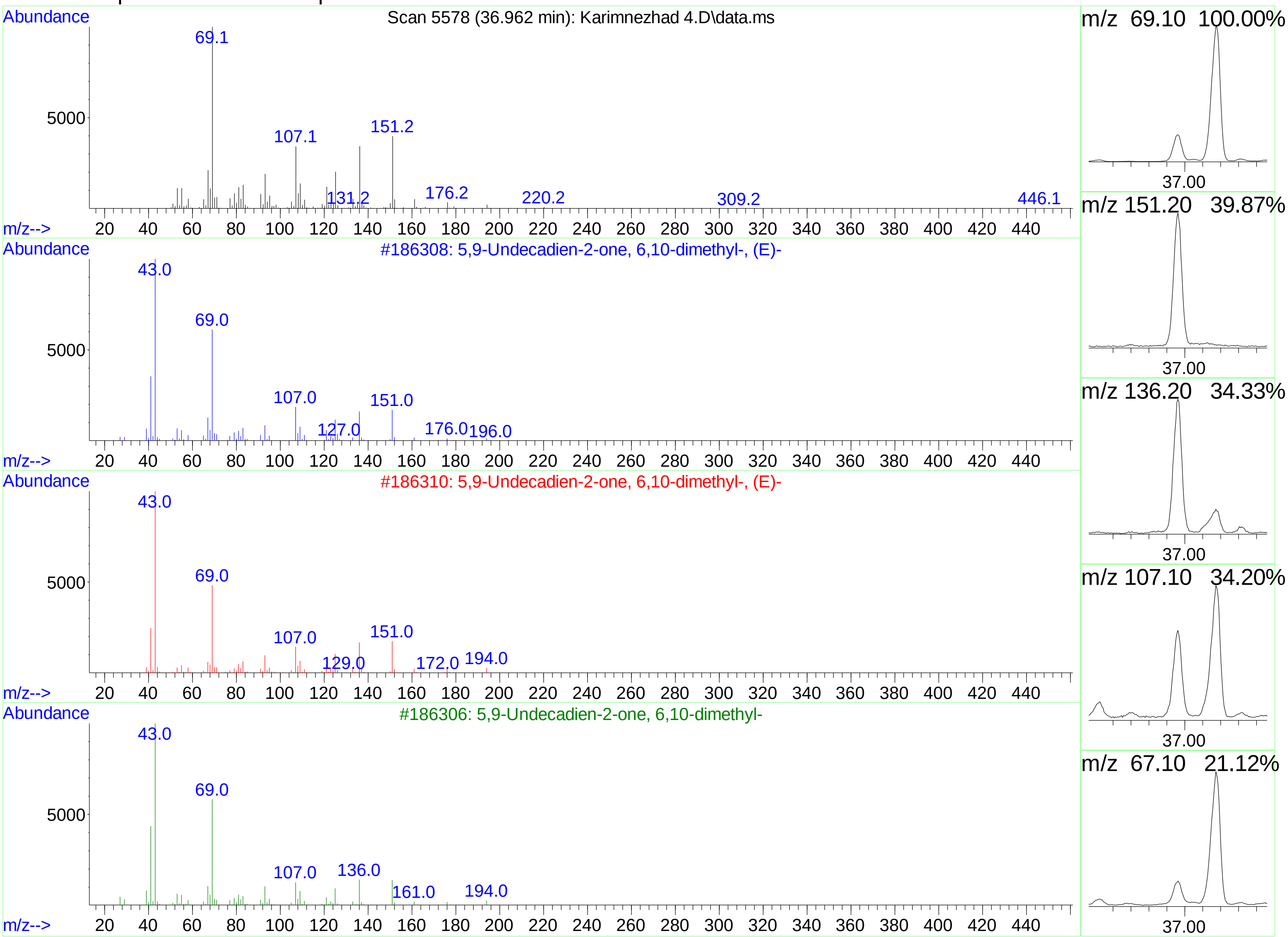

Data File: D:\msdchem\1\data\Karimnezhad 4.D
Sample : M14

Peak Number: 54 at 36.962 min Area: 32714241 Area % 0.14

The 3 best hits from each library.	Ref#	CAS#	Qual
D:\Database\W10N14.L			
1 5,9-Undecadien-2-one, 6,10-dimet...	186308	003796-70-1	93
2 5,9-Undecadien-2-one, 6,10-dimet...	186310	003796-70-1	86
3 5,9-Undecadien-2-one, 6,10-dimet...	186306	000689-67-8	86

Unknown Spectrum based on Apex

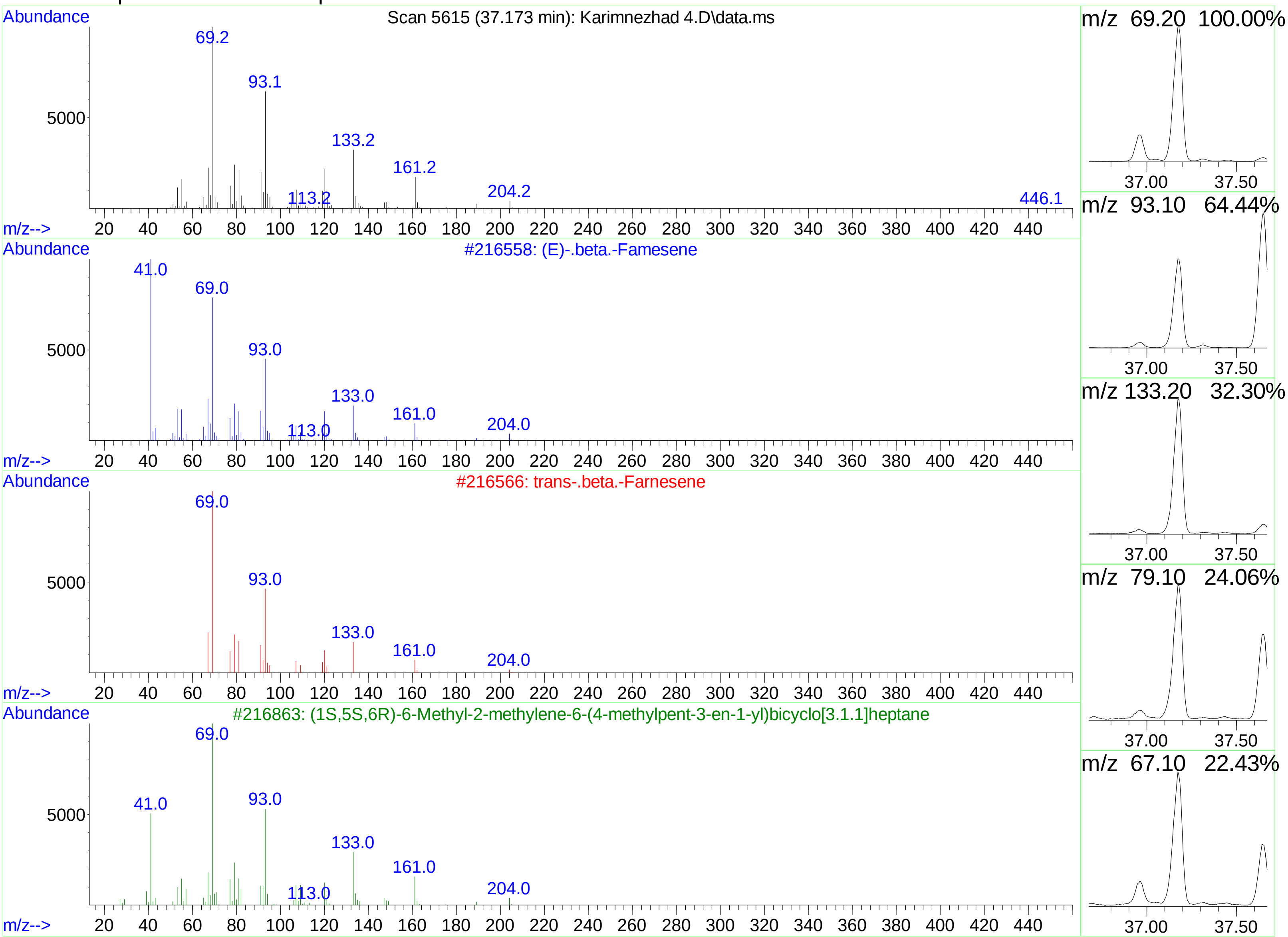

Data File: D:\msdchem\1\data\Karimnezhad 4.D
Sample : M14

Peak Number: 55 at 37.173 min Area: 148467107 Area % 0.65

The 3 best hits from each library.	Ref\#	CAS\#	Qual
D:\Database\W10N14.L			
1 (E)-.beta.-Famesene	216558	018794-84-8	96
2 trans-.beta.-Farnesene	216566	000502-60-3	96
3 (1S,5S,6R)-6-Methyl-2-methylene-...	216863	015438-94-5	95

Library Search Report - ChemStation Integrator

Unknown Spectrum based on Apex

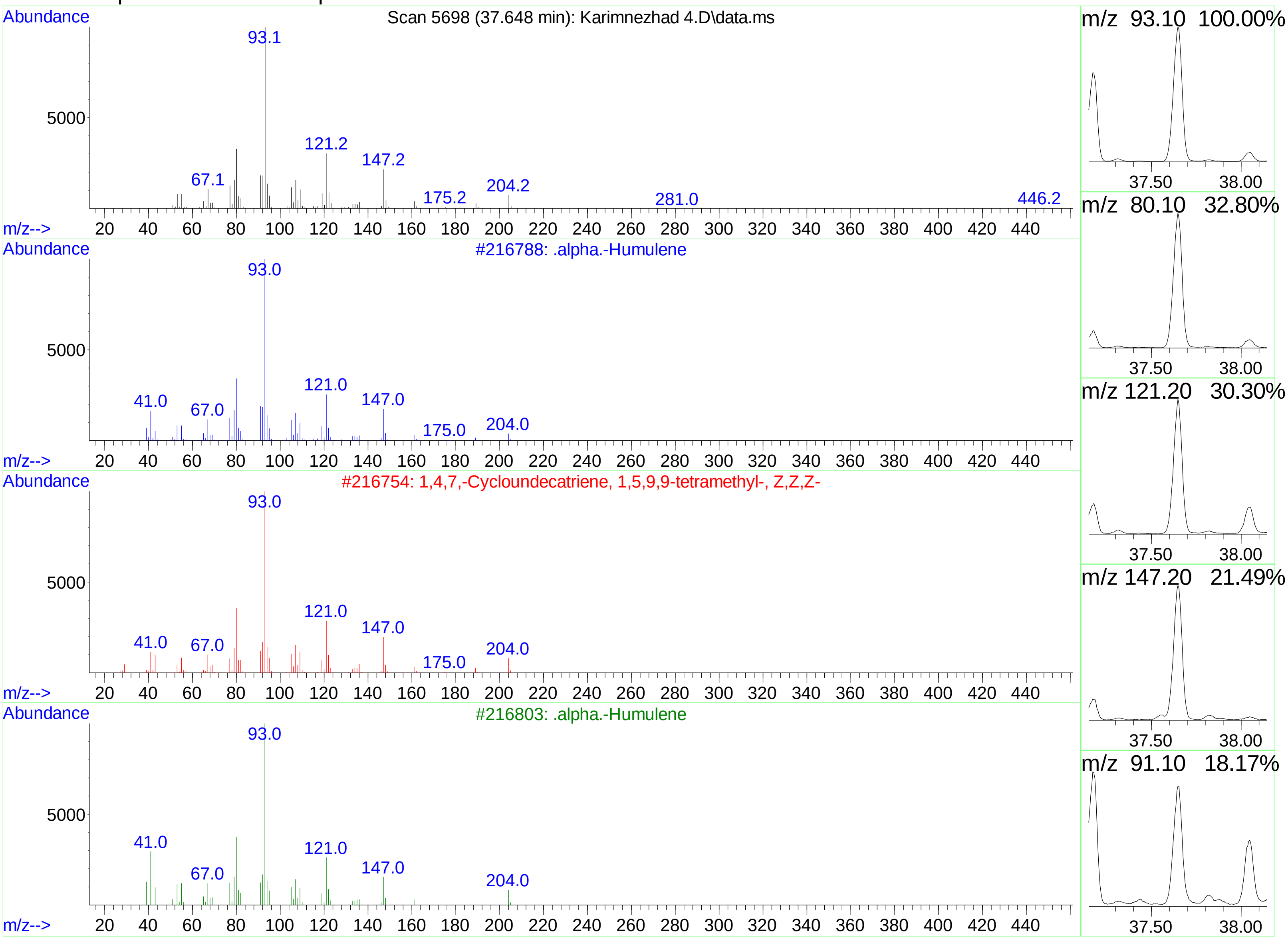

Data File: D:\msdchem\1\data\Karimnezhad 4.D
Sample : M14

Peak Number: 56 at 37.648 min Area: 114641056 Area % 0.50

The 3 best hits from each library. Ref\# CAS\# Qual

D:\Database\W10N14.L

1	.alpha.-Humulene	216788	006753-98-6	99
2	1,4,7,-Cycloundecatriene, 1,5,9,...	216754	2000216-75-4	98
3	.alpha.-Humulene	216803	006753-98-6	98

Unknown Spectrum based on Apex

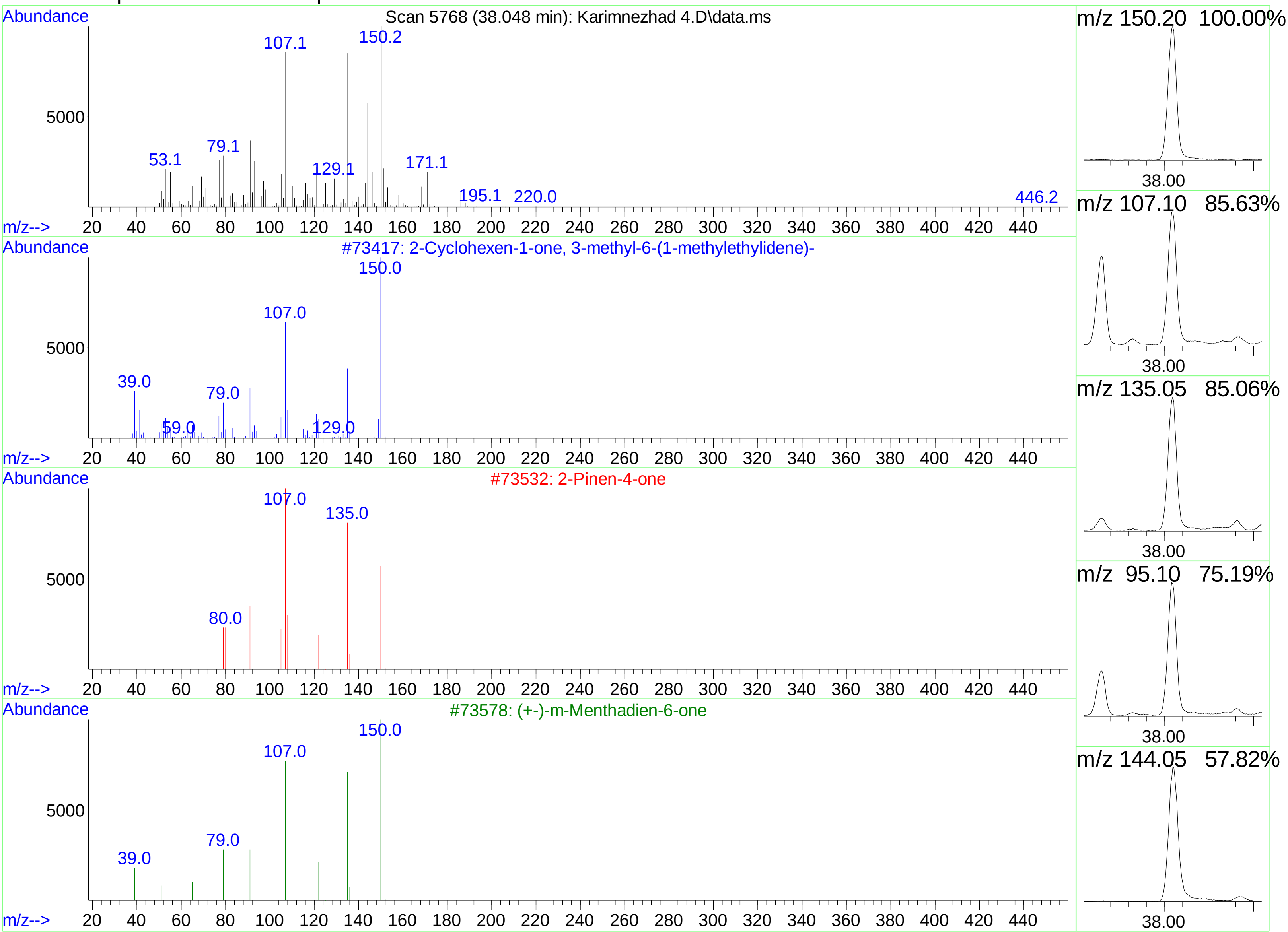

Data File: D:\msdchem\1\data\Karimnezhad 4.D
Sample : M14

Peak Number: 57 at 38.048 min Area: 94459119 Area % 0.41

The 3 best hits from each library.	Ref\#	CAS\#	Qual
D:\Database\W10N14.L			
1 2-Cyclohexen-1-one, 3-methyl-6-(...	73417	000491-09-8	55
2 2-Pinen-4-one	73532	000080-57-9	55
3 (+-)-m-Menthadien-6-one	73578	2000073-57-8	55

Unknown Spectrum based on Apex

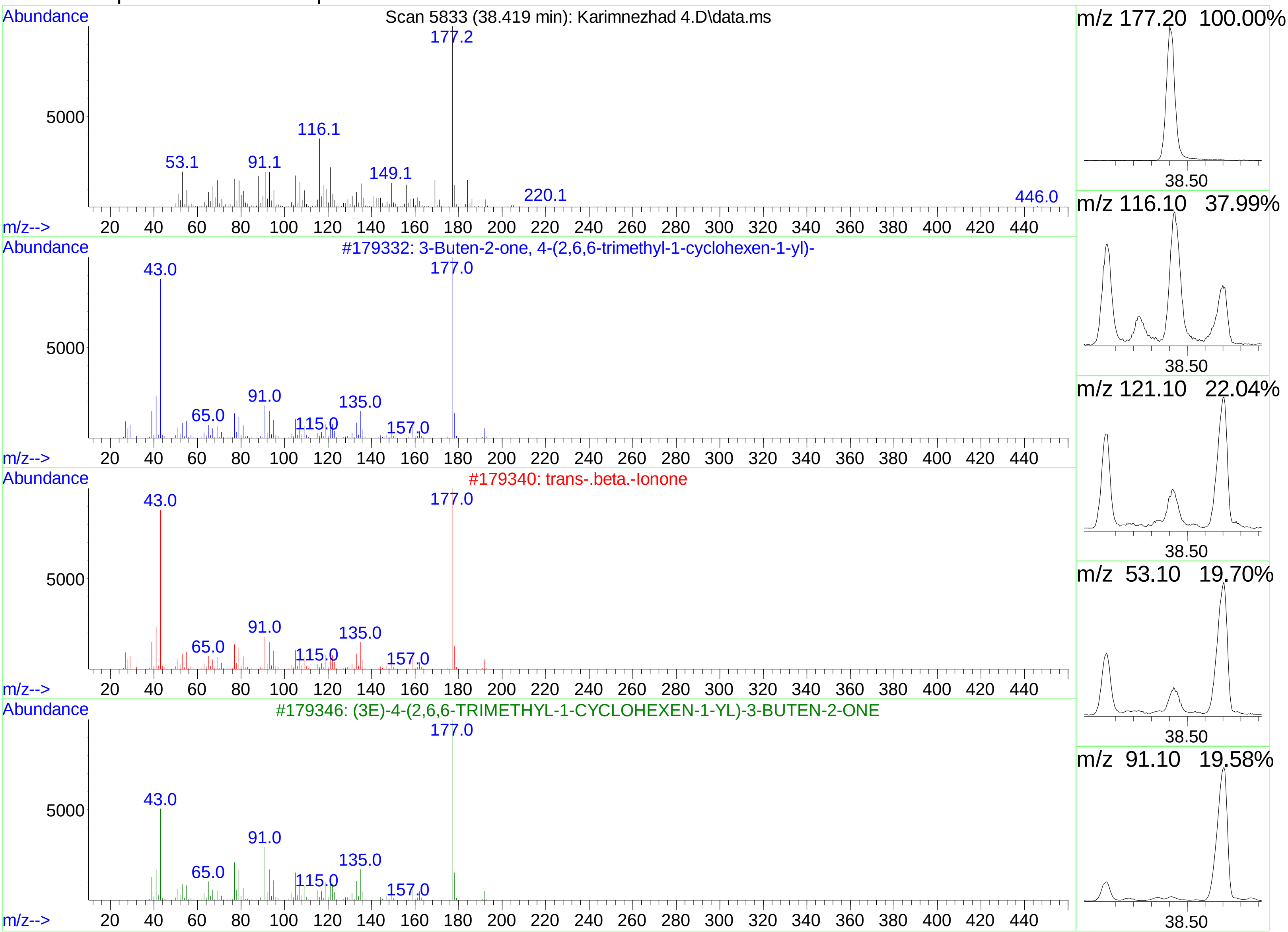

Data File: D:\msdchem\1\data\Karimnezhad 4.D
Sample : M14

Peak Number: 58 at 38.419 min Area: 43626383 Area % 0.19

The 3 best hits from each library. Ref\# CAS\# Qual

D:\Database\W10N14.L

1 3-Buten-2-one, 4-(2,6,6-trimethy... 179332 014901-07-6 97
2 trans-.beta.-Ionone 179340 000079-77-6 97
3 (3E)-4-(2,6,6-TRIMETHYL-1-CYCLOH... 179346 000079-77-6 96

Unknown Spectrum based on Apex

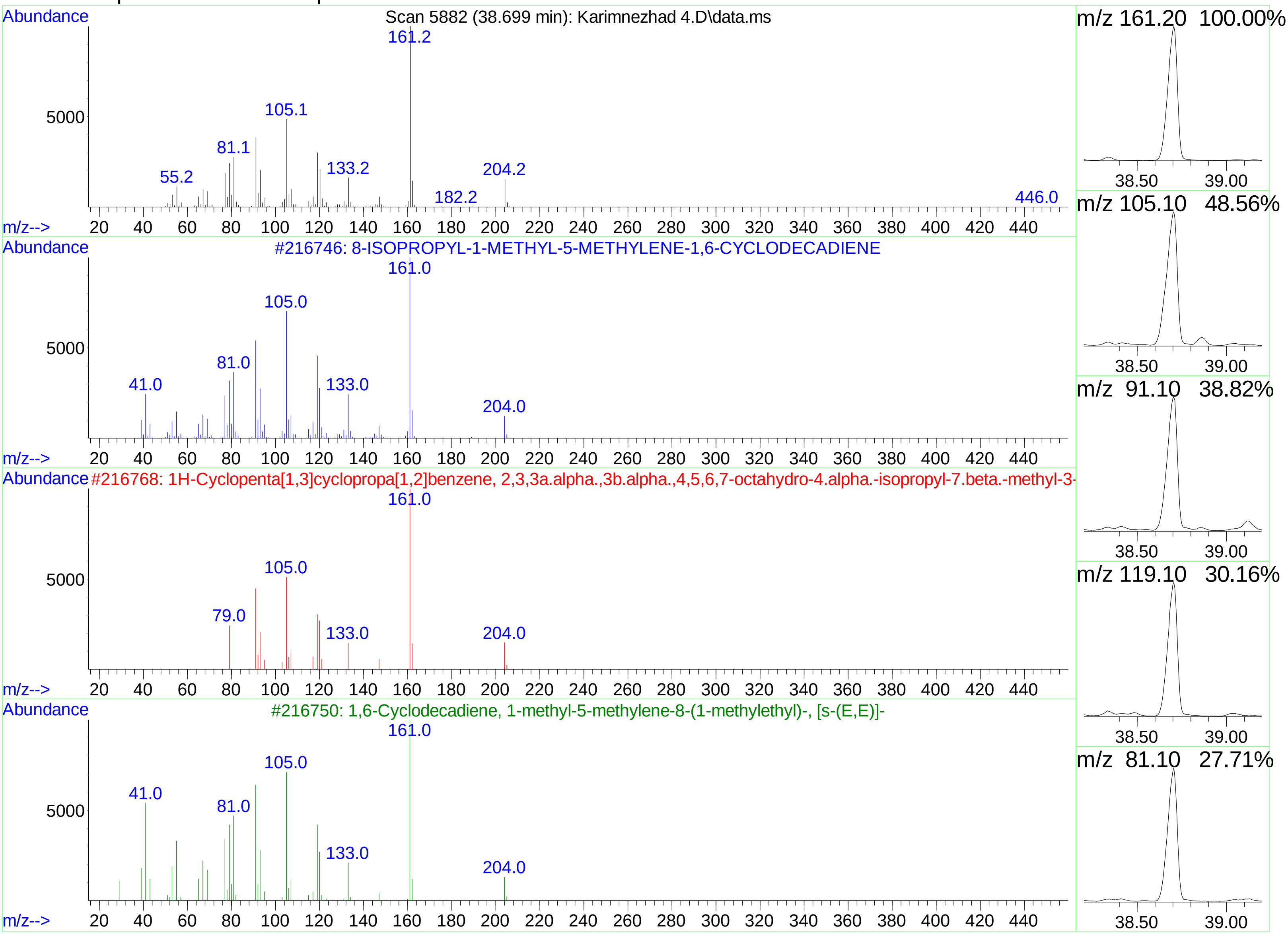

Data File: D:\msdchem\1\data\Karimnezhad 4.D
Sample : M14

Peak Number: 59 at 38.699 min Area: 302000683 Area % 1.32

The 3 best hits from each library. Ref\# CAS\# Qual

D:\Database\W10N14.L

1	8-ISOPROPYL-1-METHYL-5-METHYLENE...	216746	023986-74-5	98
2	1H-Cyclopenta[1,3]cyclopropa[1,2...	216768	013744-15-5	98
3	1,6-Cyclodecadiene, 1-methyl-5-m...	216750	023986-74-5	98

Unknown Spectrum based on Apex

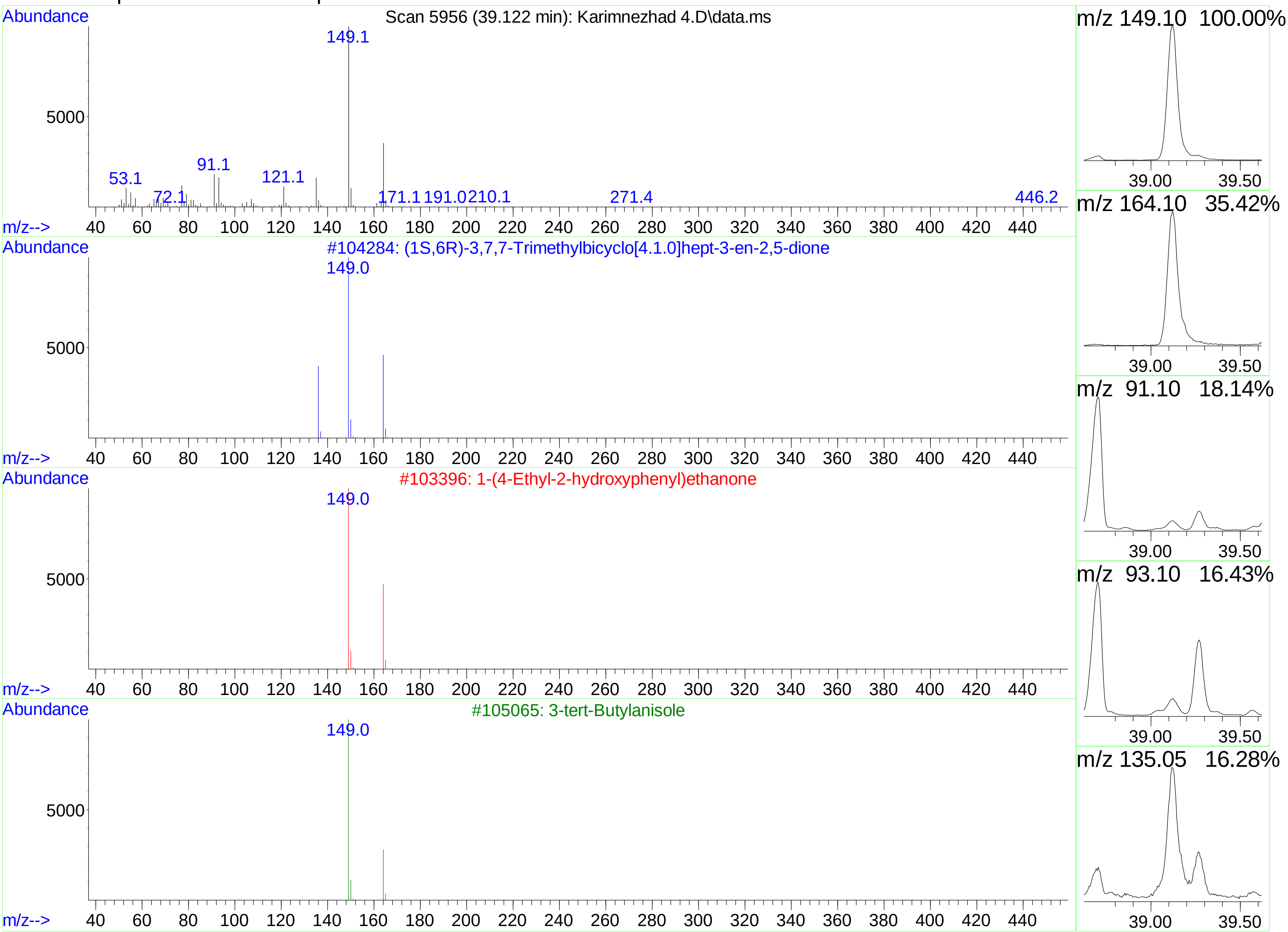

Data File: D:\msdchem\1\data\Karimnezhad 4.D
Sample : M14

Peak Number: 60 at 39.122 min Area: 36226788 Area % 0.16

The 3 best hits from each library.	Ref\#	CAS\#	Qual
D:\Database\W10N14.L			
1 (1S,6R)-3,7,7-Trimethylbicyclo[4...	104284	2000104-28-4	90
2 1-(4-Ethyl-2-hydroxyphenyl)ethanone	103396	2000103-39-6	83
3 3-tert-Butylanisole	105065	2000105-06-5	83

Unknown Spectrum based on Apex

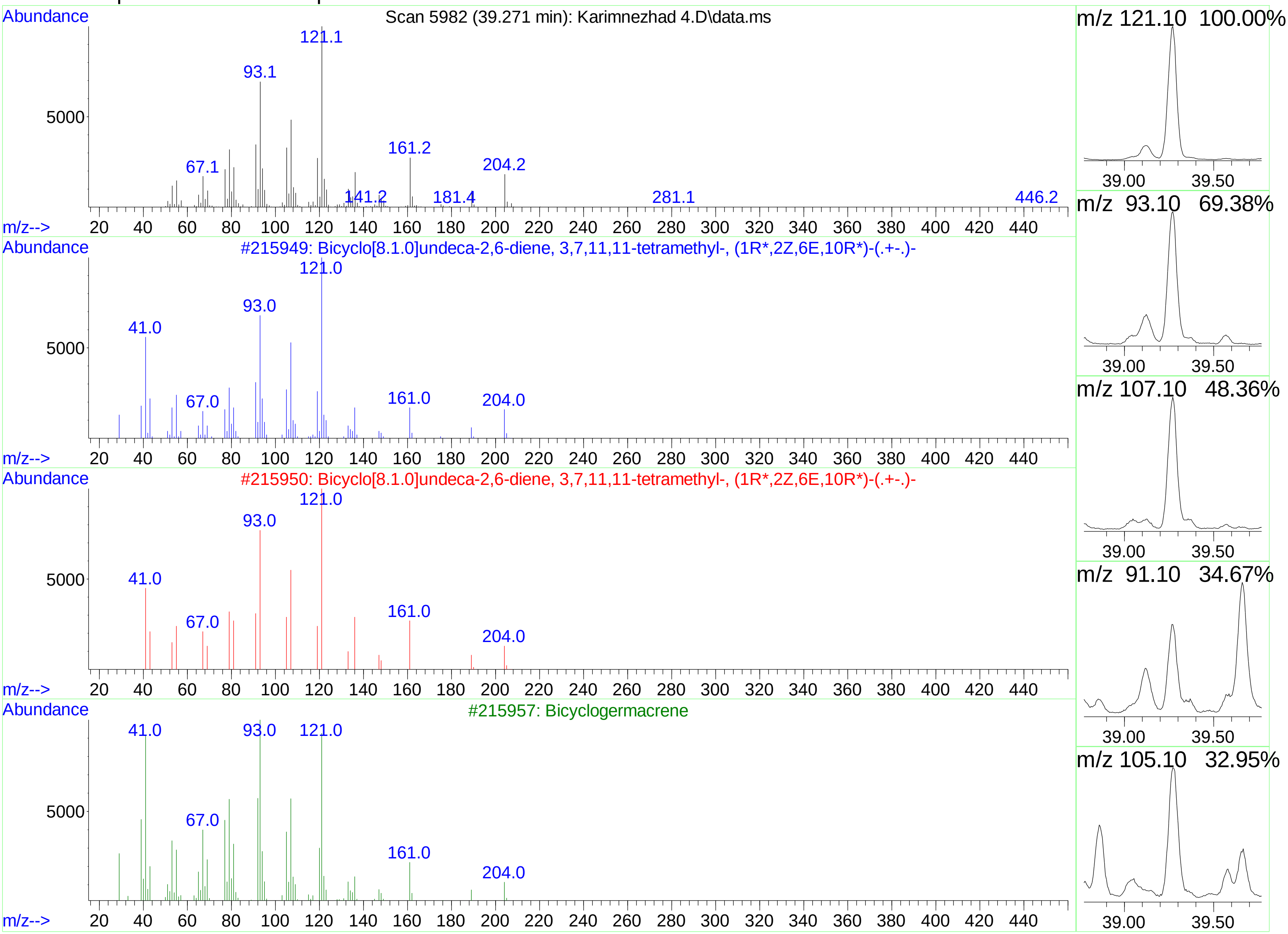

Data File: D:\msdchem\1\data\Karimnezhad 4.D
Sample : M14

Peak Number: 61 at 39.271 min Area: 100359798 Area % 0.44

The 3 best hits from each library.	Ref#	CAS#	Qual
D:\Database\W10N14.L			
1 Bicyclo[8.1.0]undeca-2,6-diene, ...	215949	100762-46-7	99
2 Bicyclo[8.1.0]undeca-2,6-diene, ...	215950	100762-46-7	98
3 Bicyclogermacrene	215957	067650-90-2	91

Unknown Spectrum based on Apex

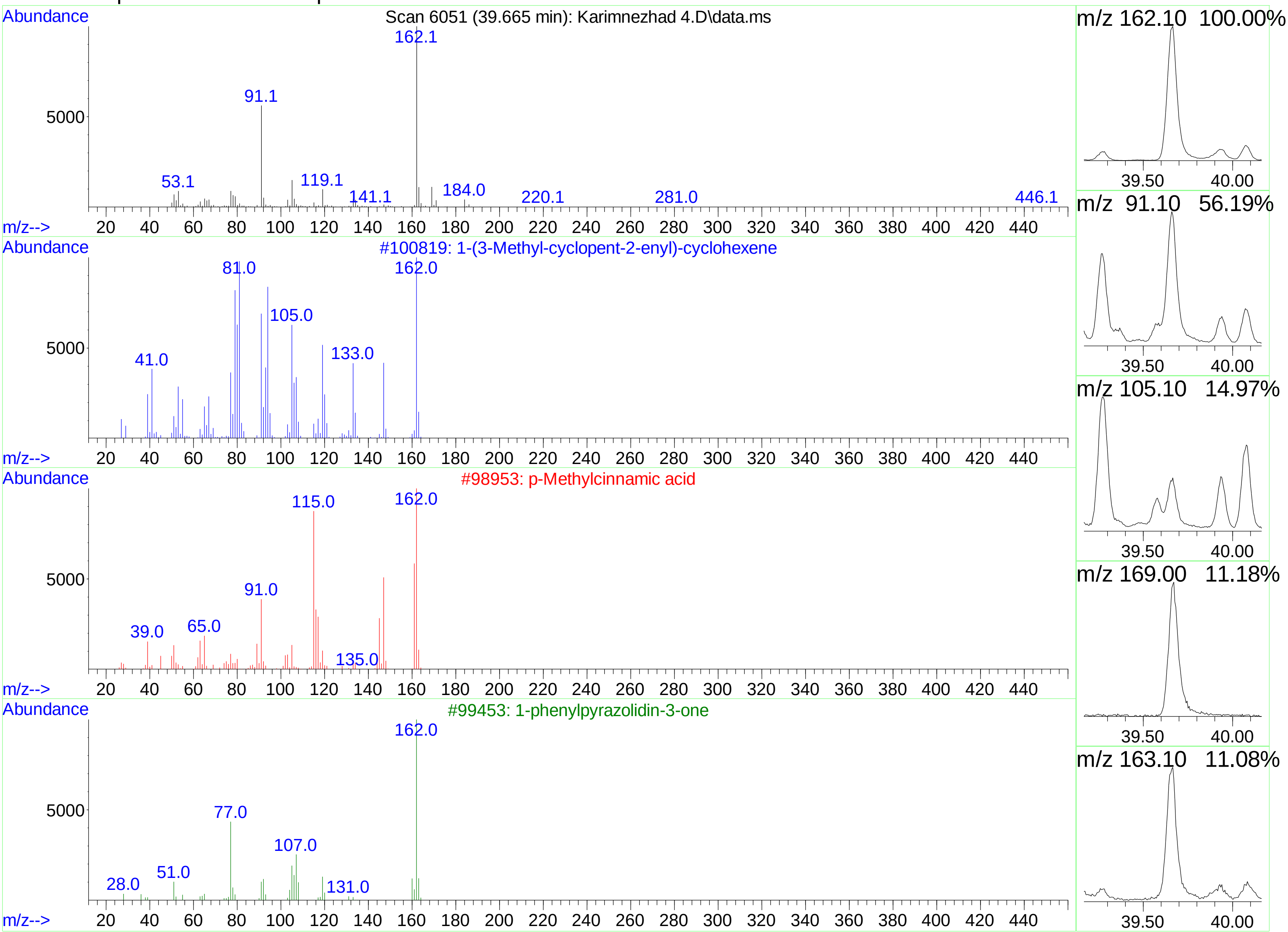

Data File: D:\msdchem\1\data\Karimnezhad 4.D
Sample : M14

Peak Number: 62 at 39.665 min Area: 37881774 Area % 0.17

The 3 best hits from each library.	Ref#	CAS#	Qual
D:\Database\W10N14.L			
1 1-(3-Methyl-cyclopent-2-enyl)-cy...	100819	2000100-81-9	94
2 p-Methylcinnamic acid	98953	001866-39-3	64
3 1-phenylpyrazolidin-3-one	99453	000092-43-3	59

Unknown Spectrum based on Apex

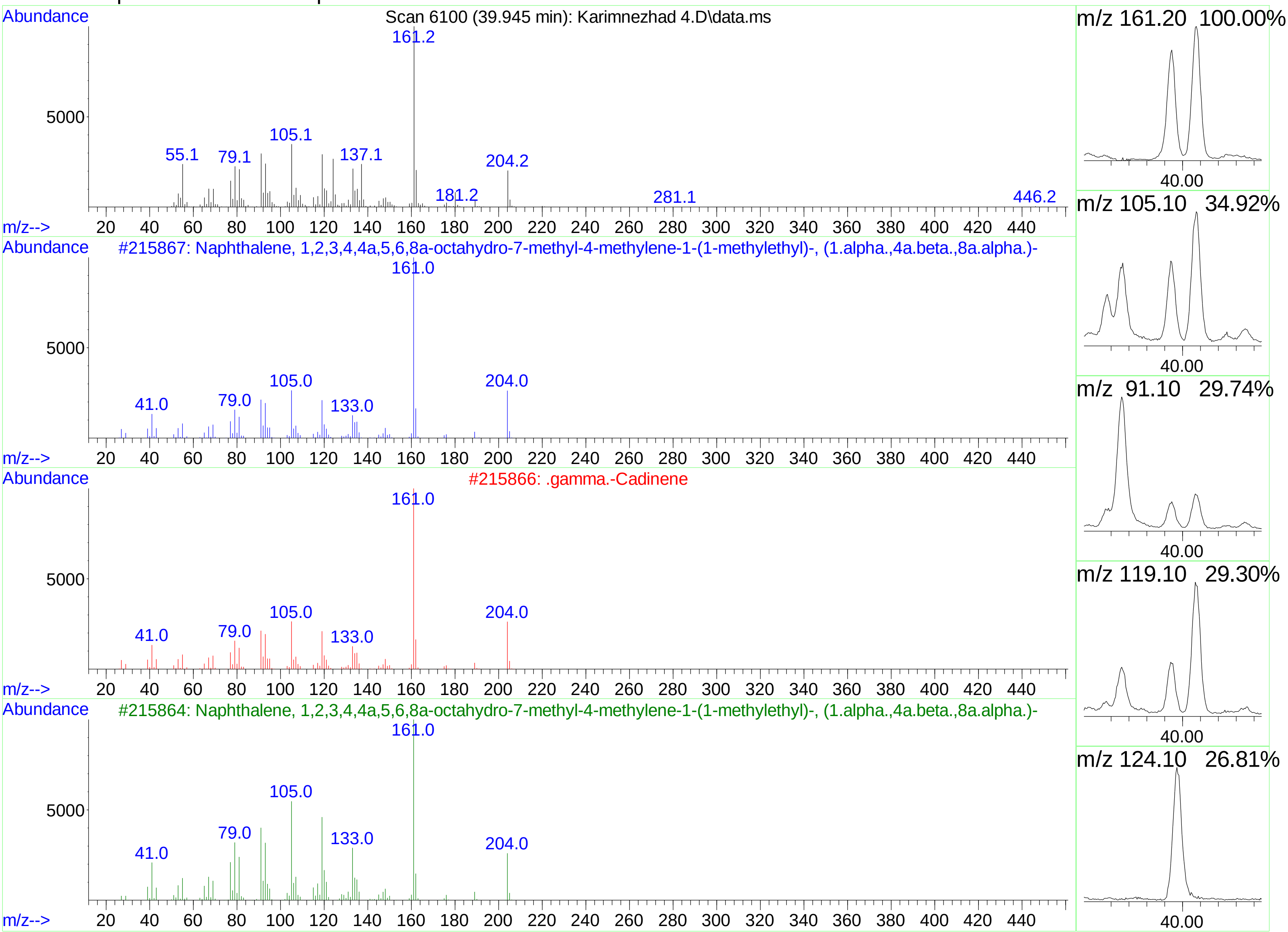

Data File: D:\msdchem\1\data\Karimnezhad 4.D
Sample : M14

Peak Number: 63 at 39.945 min Area: 18299558 Area % 0.08

The 3 best hits from each library.	Ref\#	CAS\#	Qual
D:\Database\W10N14.L			
1 Naphthalene, 1,2,3,4,4a,5,6,8a-o...	215867	039029-41-9	98
2 .gamma.-Cadinene	215866	039029-41-9	98
3 Naphthalene, 1,2,3,4,4a,5,6,8a-o...	215864	039029-41-9	96

Unknown Spectrum based on Apex

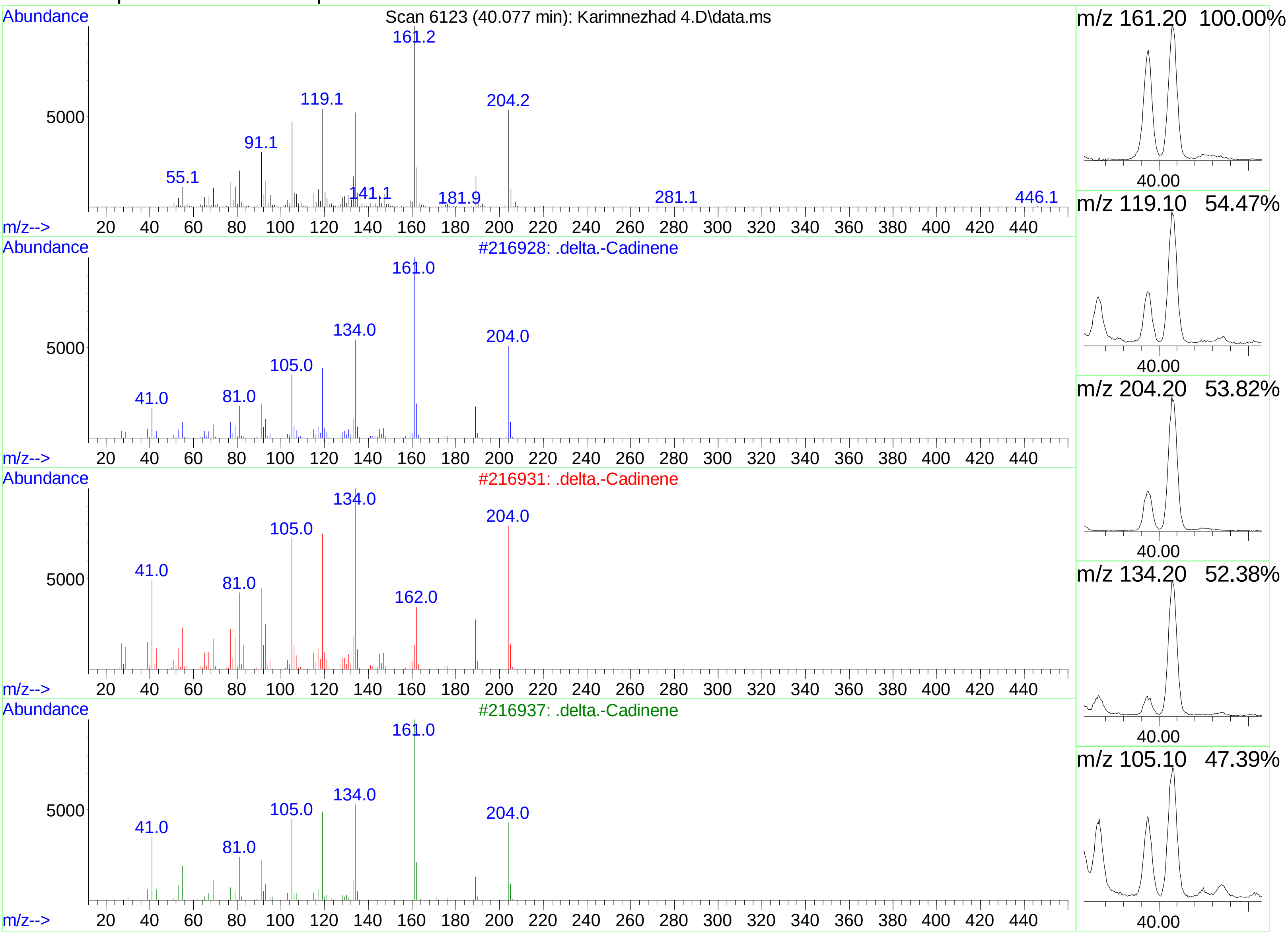

Data File: D:\msdchem\1\data\Karimnezhad 4.D
Sample : M14

Peak Number: 64 at 40.077 min Area: 21802365 Area % 0.10

The 3 best hits from each library.	Ref\#	CAS\#	Qual
D:\Database\W10N14.L			
1 .delta.-Cadinene	216928	000483-76-1	99
2 .delta.-Cadinene	216931	000483-76-1	99
3 .delta.-Cadinene	216937	000483-76-1	98

Unknown Spectrum based on Apex

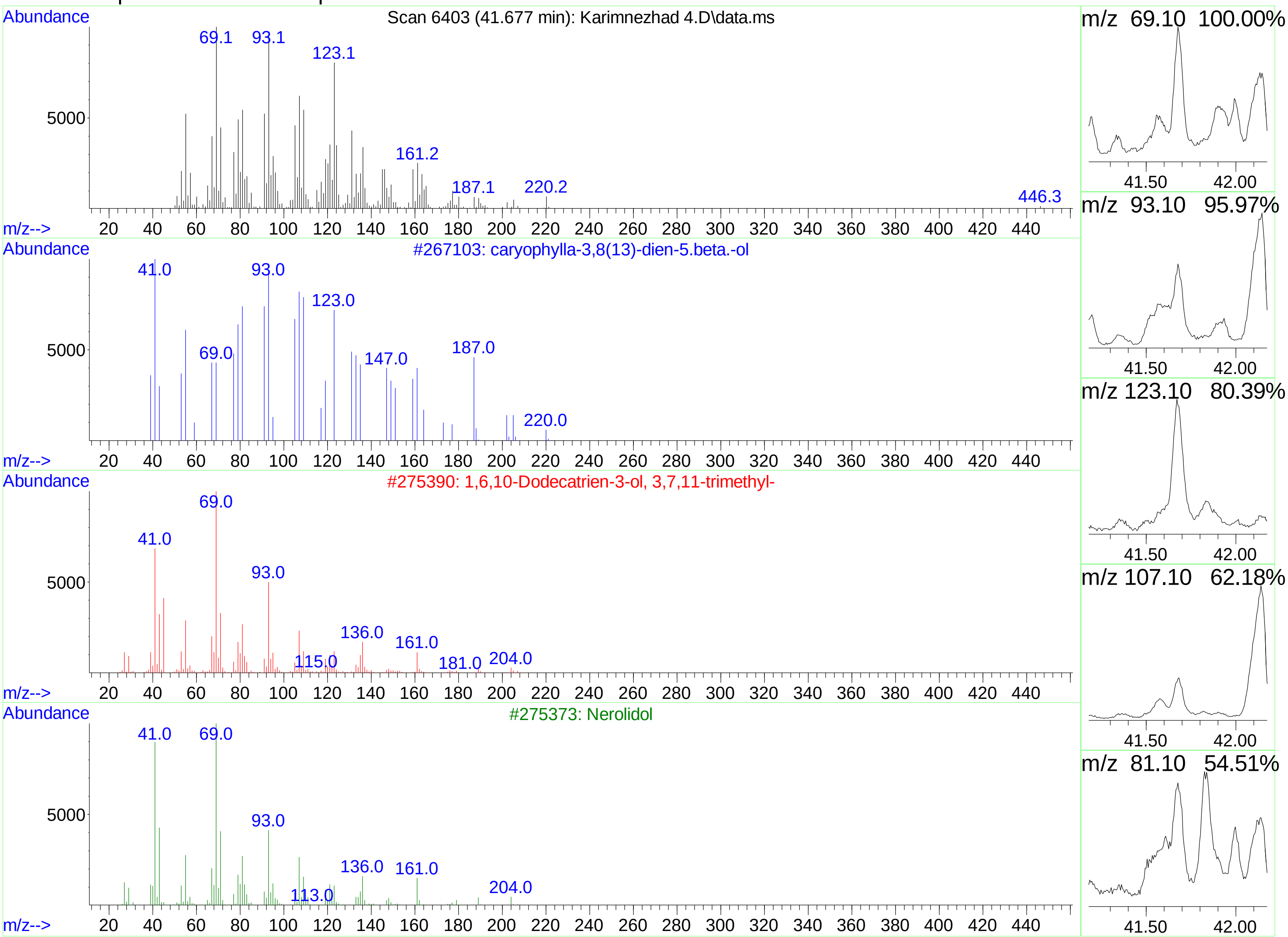

Data File: D:\msdchem\1\data\Karimnezhad 4.D
Sample : M14

Peak Number: 65 at 41.677 min Area: 42330905 Area % 0.18

The 3 best hits from each library.	Ref#	CAS#	Qual
D:\Database\W10N14.L			
1 caryophylla-3,8(13)-dien-5.beta.-ol	267103	2000267-10-3	49
2 1,6,10-Dodecatrien-3-ol, 3,7,11-...	275390	007212-44-4	42
3 Nerolidol	275373	000142-50-7	38

Unknown Spectrum based on Apex

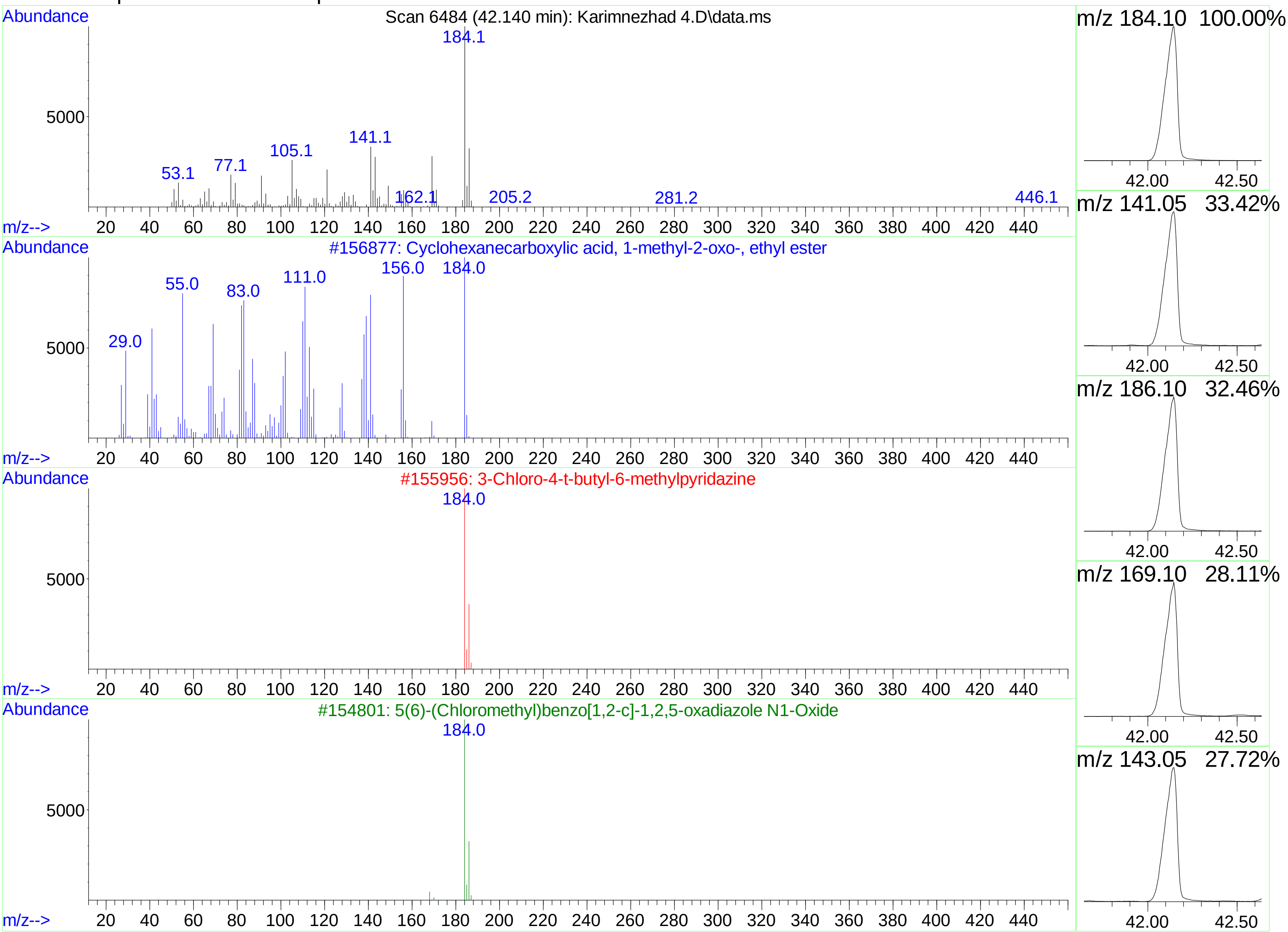

Data File: D:\msdchem\1\data\Karimnezhad 4.D
Sample : M14

Peak Number: 66 at 42.140 min Area: 226580234 Area % 0.99

The 3 best hits from each library.	Ref\#	CAS\#	Qual
D:\Database\W10N14.L			
1 Cyclohexanecarboxylic acid, 1-me...	156877	005453-94-1	83
2 3-Chloro-4-t-butyl-6-methylpyrid...	155956	2000155-95-6	83
3 5(6)-(Chloromethyl)benzo[1,2-c]-...	154801	2000154-80-1	59

Unknown Spectrum based on Apex

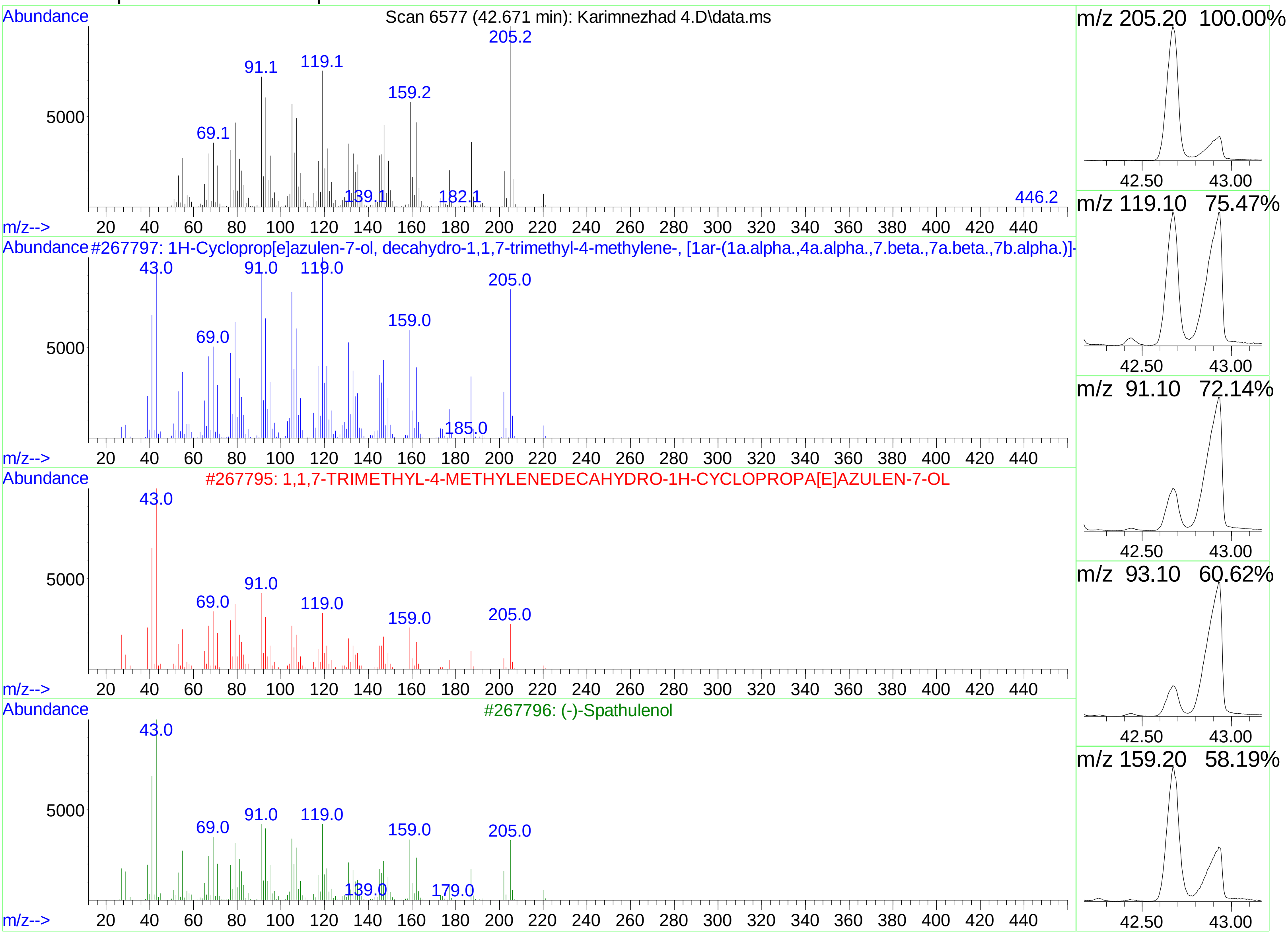

Data File: D:\msdchem\1\data\Karimnezhad 4.D
Sample : M14

Peak Number: 67 at 42.671 min Area: 209056556 Area % 0.91

The 3 best hits from each library. Ref\# CAS\# Qual

D:\Database\W10N14.L

1	1H-Cycloprop[e]azulen-7-ol, deca...	267797	006750-60-3	98
2	1,1,7-TRIMETHYL-4-METHYLENEDECAH...	267795	077171-55-2	98
3	(-)-Spathulenol	267796	077171-55-2	95

Unknown Spectrum based on Apex

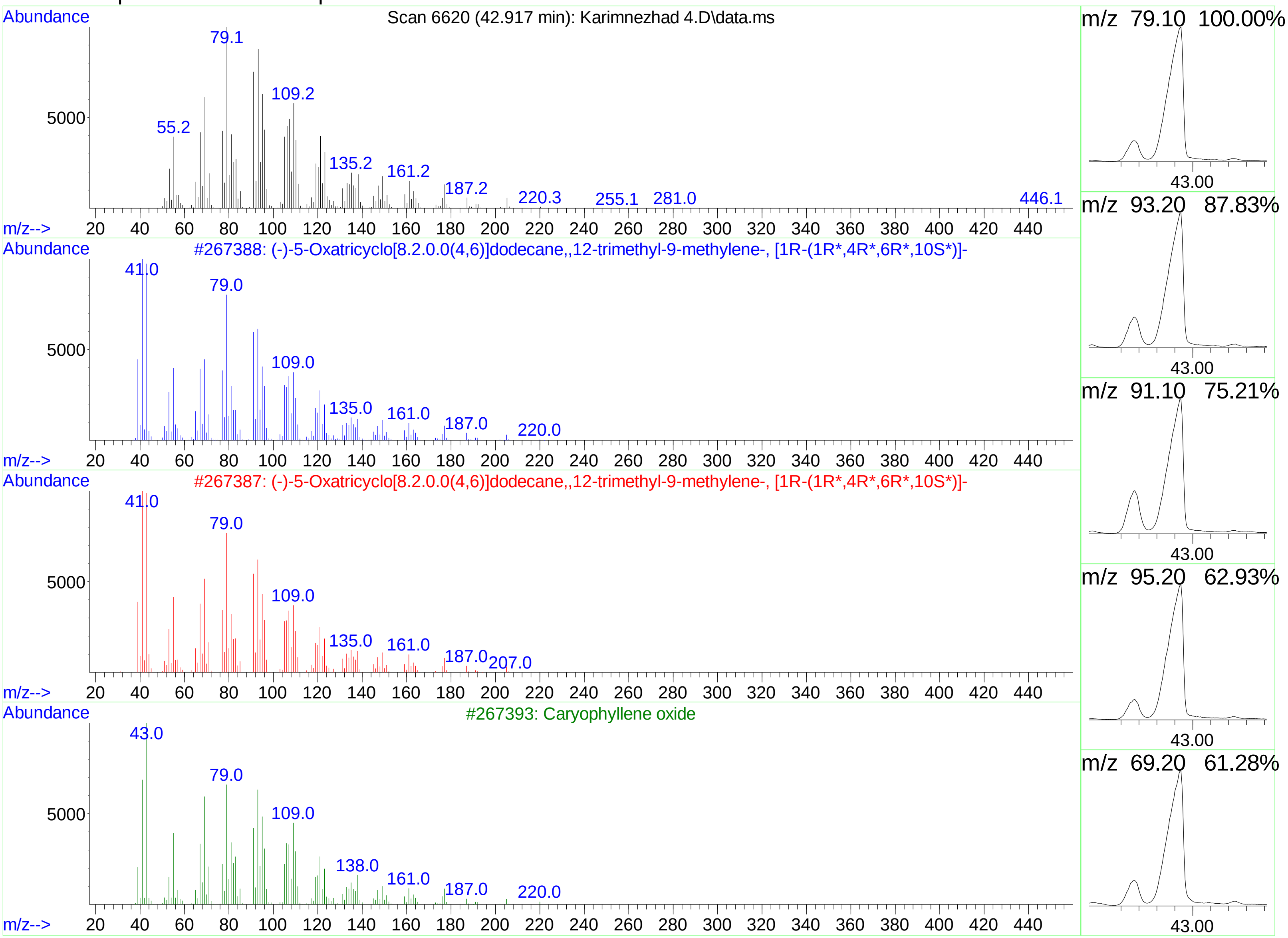

Data File: D:\msdchem\1\data\Karimnezhad 4.D
Sample : M14

Peak Number: 68 at 42.917 min Area: 778464994 Area % 3.40

The 3 best hits from each library.	Ref#	CAS#	Qual
D:\Database\W10N14.L			
1 (-)-5-Oxatricyclo[8.2.0.0(4,6)]d...	267388	001139-30-6	99
2 (-)-5-Oxatricyclo[8.2.0.0(4,6)]d...	267387	001139-30-6	95
3 Caryophyllene oxide	267393	001139-30-6	91

Library Search Report - ChemStation Integrator

Unknown Spectrum based on Apex

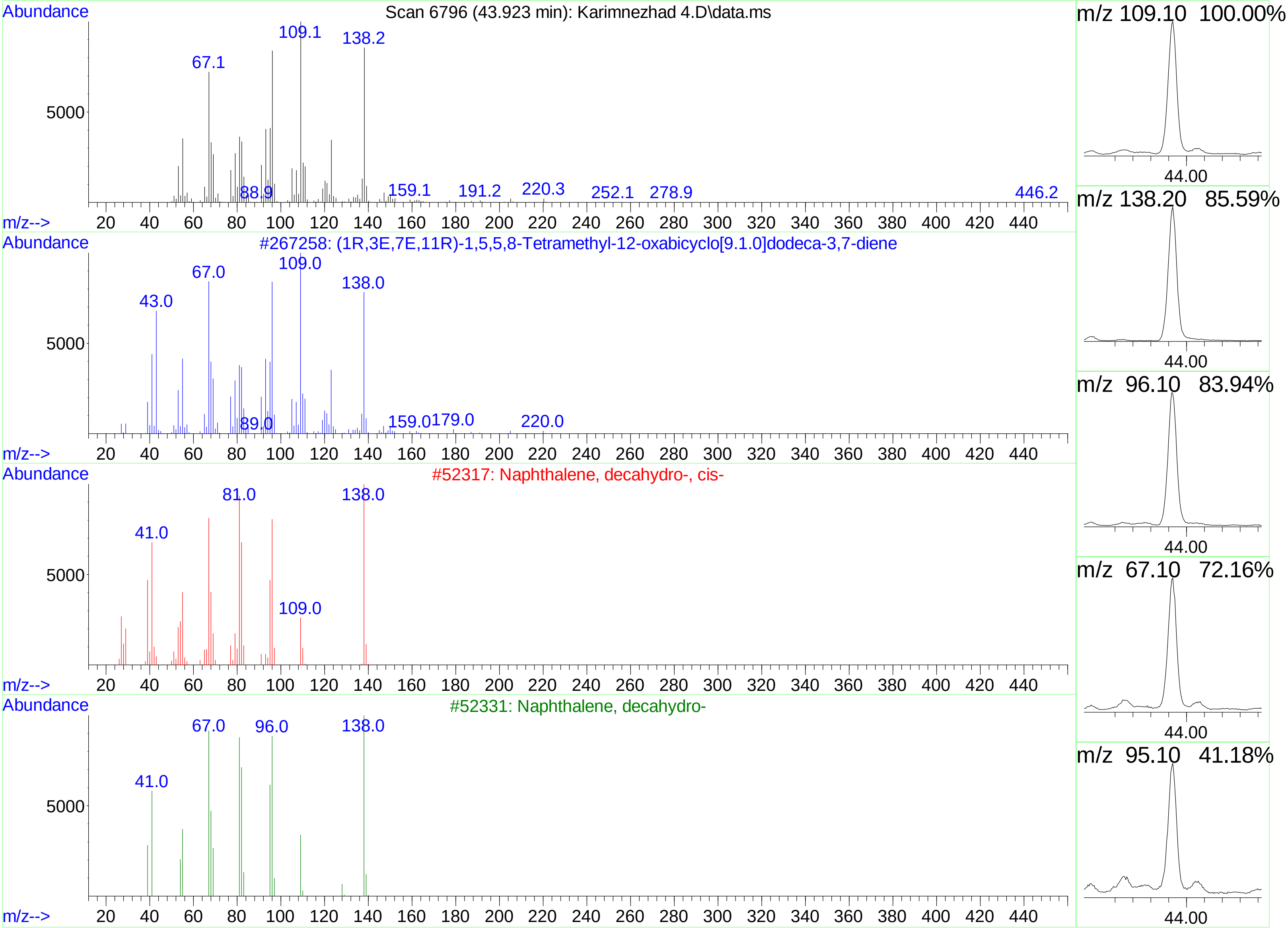

Data File: D:\msdchem\1\data\Karimnezhad 4.D
Sample : M14

Peak Number: 69 at 43.923 min Area: 63727992 Area % 0.28

The 3 best hits from each library.	Ref\#	CAS\#	Qual
D:\Database\W10N14.L			
1 (1R,3E,7E,11R)-1,5,5,8-Tetrameth...	267258	019888-34-7	99
2 Naphthalene, decahydro-, cis-	52317	000493-01-6	78
3 Naphthalene, decahydro-	52331	000091-17-8	53

Unknown Spectrum based on Apex

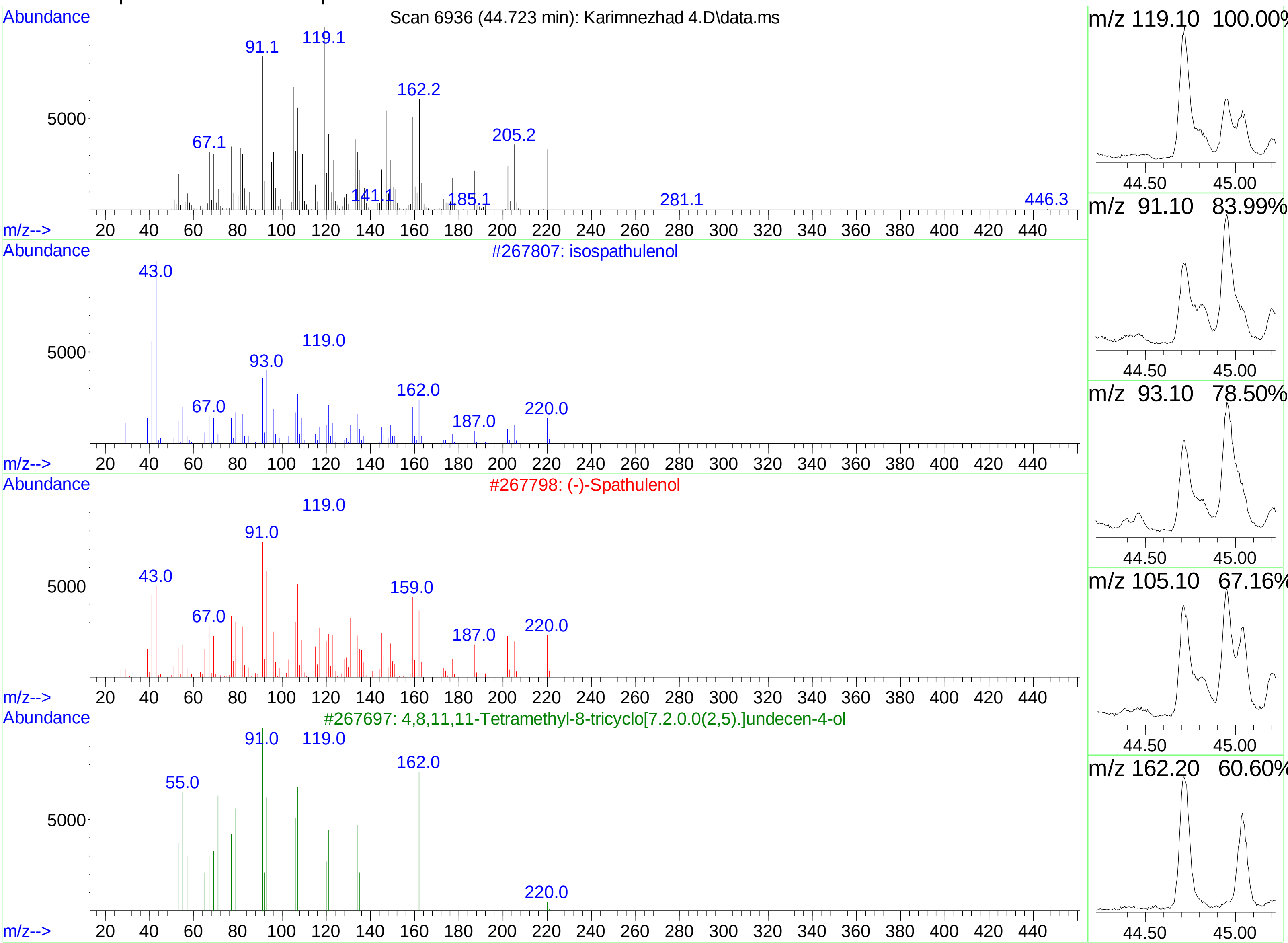

Data File: D:\msdchem\1\data\Karimnezhad 4.D
Sample : M14

Peak Number: 70 at 44.723 min Area: 53358265 Area % 0.23

The 3 best hits from each library. Ref\# CAS\# Qual

D:\Database\W10N14.L

1	isospathulenol	267807	2000267-80-7	99
2	(-)-Spathulenol	267798	077171-55-2	95
3	4,8,11,11-Tetramethyl-8-tricyclo...	267697	2000267-69-7	50

Library Search Report - ChemStation Integrator

Unknown Spectrum based on Apex

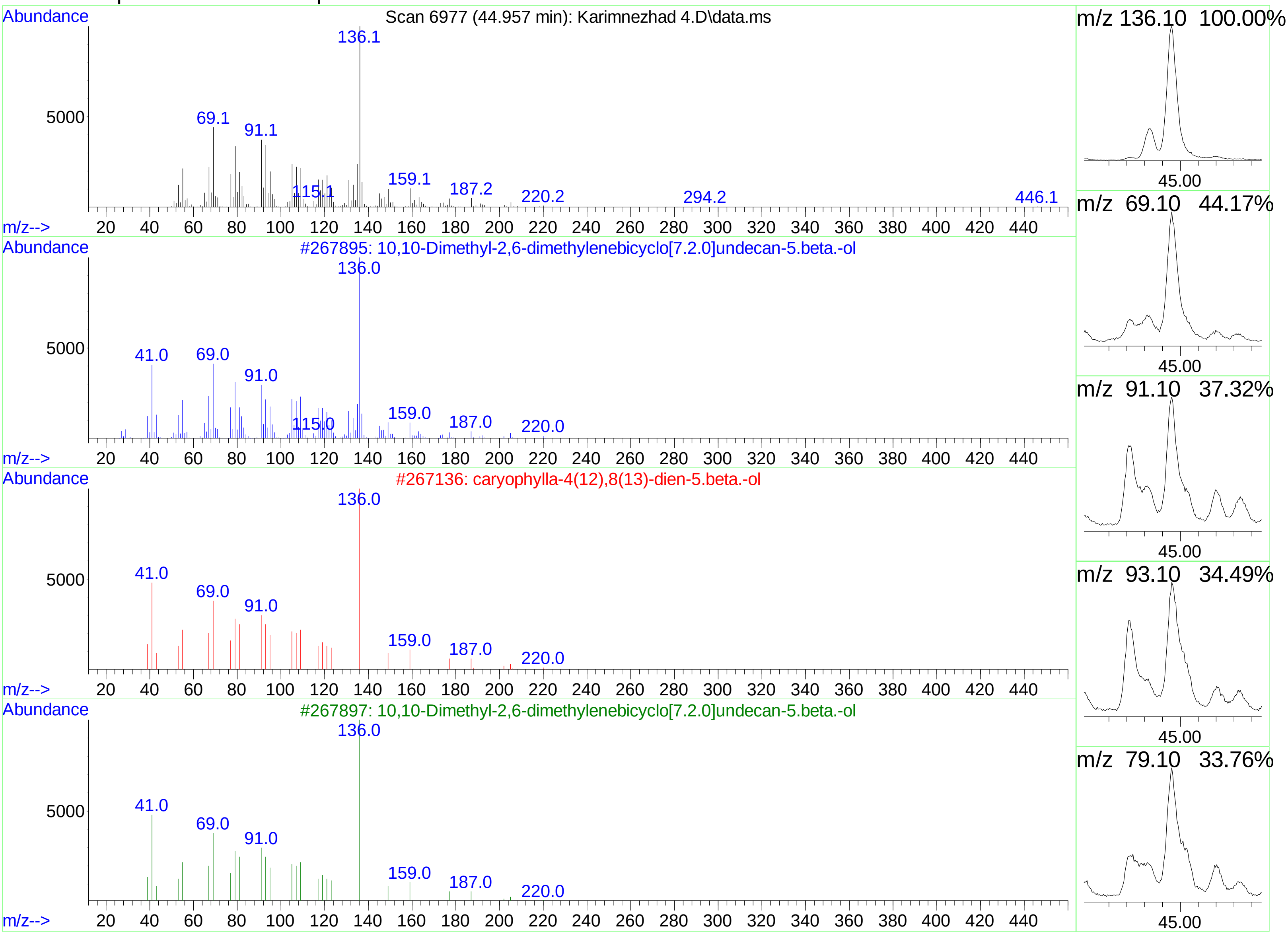

Data File: D:\msdchem\1\data\Karimnezhad 4.D
Sample : M14

Peak Number: 71 at 44.957 min Area: 79016630 Area % 0.34

The 3 best hits from each library.	Ref#	CAS#	Qual
D:\Database\W10N14.L			
1 10,10-Dimethyl-2,6-dimethylenebi...	267895	019431-80-2	98
2 caryophylla-4(12),8(13)-dien-5.b...	267136	2000267-13-6	94
3 10,10-Dimethyl-2,6-dimethylenebi...	267897	019431-80-2	94

Unknown Spectrum based on Apex

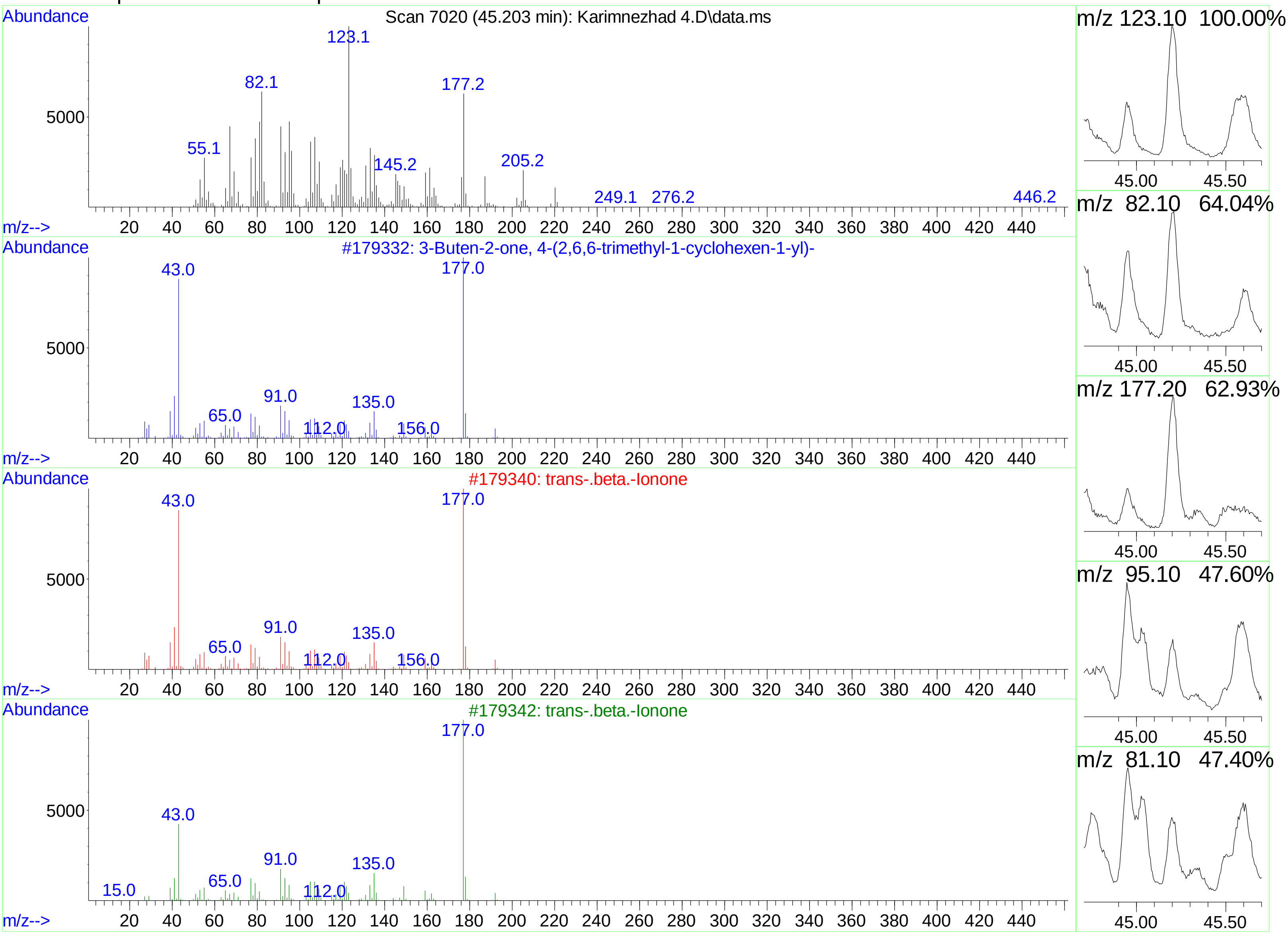

Data File: D:\msdchem\1\data\Karimnezhad 4.D
Sample : M14

Peak Number: 72 at 45.203 min Area: 21347460 Area % 0.09

The 3 best hits from each library. Ref\# CAS\# Qual

D:\Database\W10N14.L

		Ref\#	CAS\#	Qual
1	3-Buten-2-one, 4-(2,6,6-trimethy...	179332	014901-07-6	52
2	trans-.beta.-Ionone	179340	000079-77-6	52
3	trans-.beta.-Ionone	179342	000079-77-6	47

Library Search Report - ChemStation Integrator

Unknown Spectrum based on Apex

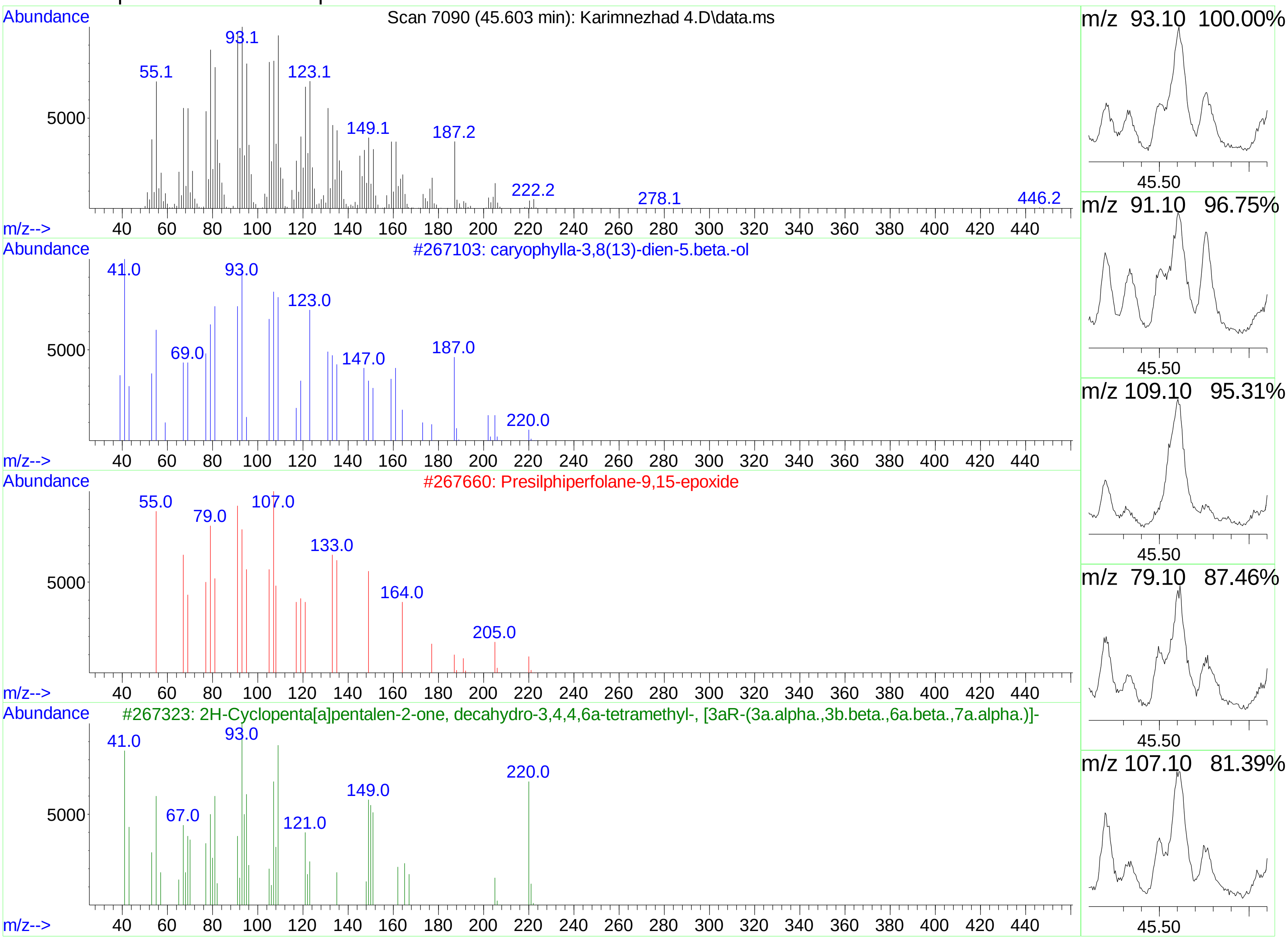

Data File: D:\msdchem\1\data\Karimnezhad 4.D
Sample : M14

Peak Number: 73 at 45.603 min Area: 51086342 Area % 0.22

The 3 best hits from each library.	Ref\#	CAS\#	Qual
D:\Database\W10N14.L			
1 caryophylla-3,8(13)-dien-5.beta.-ol	267103	2000267-10-3	92
2 Presilphiperfolane-9,15-epoxide	267660	2000267-66-0	91
3 2H-Cyclopenta[a]pentalen-2-one, ...	267323	061228-70-4	56

Unknown Spectrum based on Apex

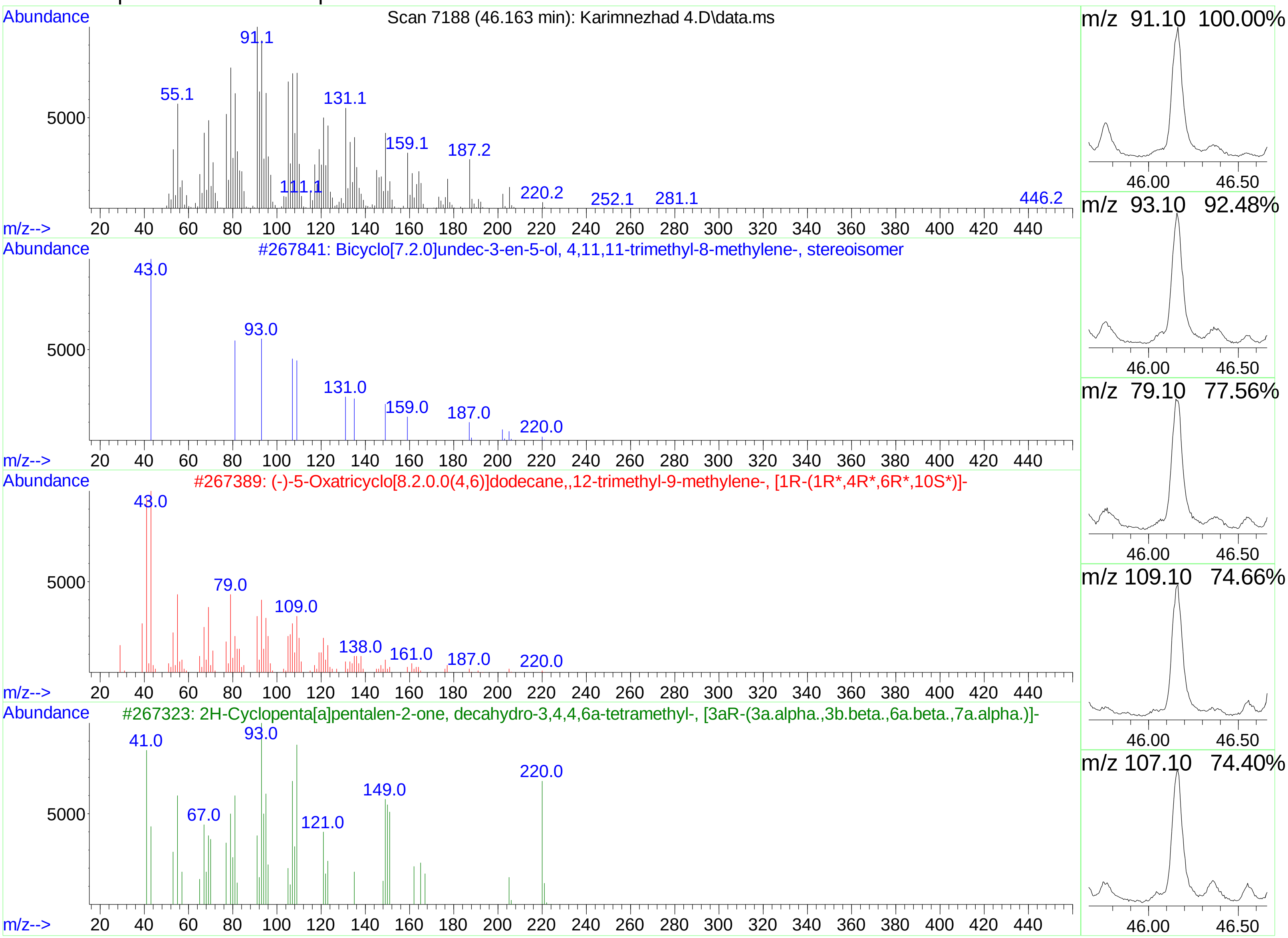

Data File: D:\msdchem\1\data\Karimnezhad 4.D
Sample : M14

Peak Number: 74 at 46.163 min Area: 82108029 Area % 0.36

The 3 best hits from each library.	Ref\#	CAS\#	Qual
D:\Database\W10N14.L			
1 Bicyclo[7.2.0]undec-3-en-5-ol, 4...	267841	032214-89-4	86
2 (-)-5-Oxatricyclo[8.2.0.0(4,6)]d...	267389	001139-30-6	83
3 2H-Cyclopenta[a]pentalen-2-one, ...	267323	061228-70-4	64

Unknown Spectrum based on Apex

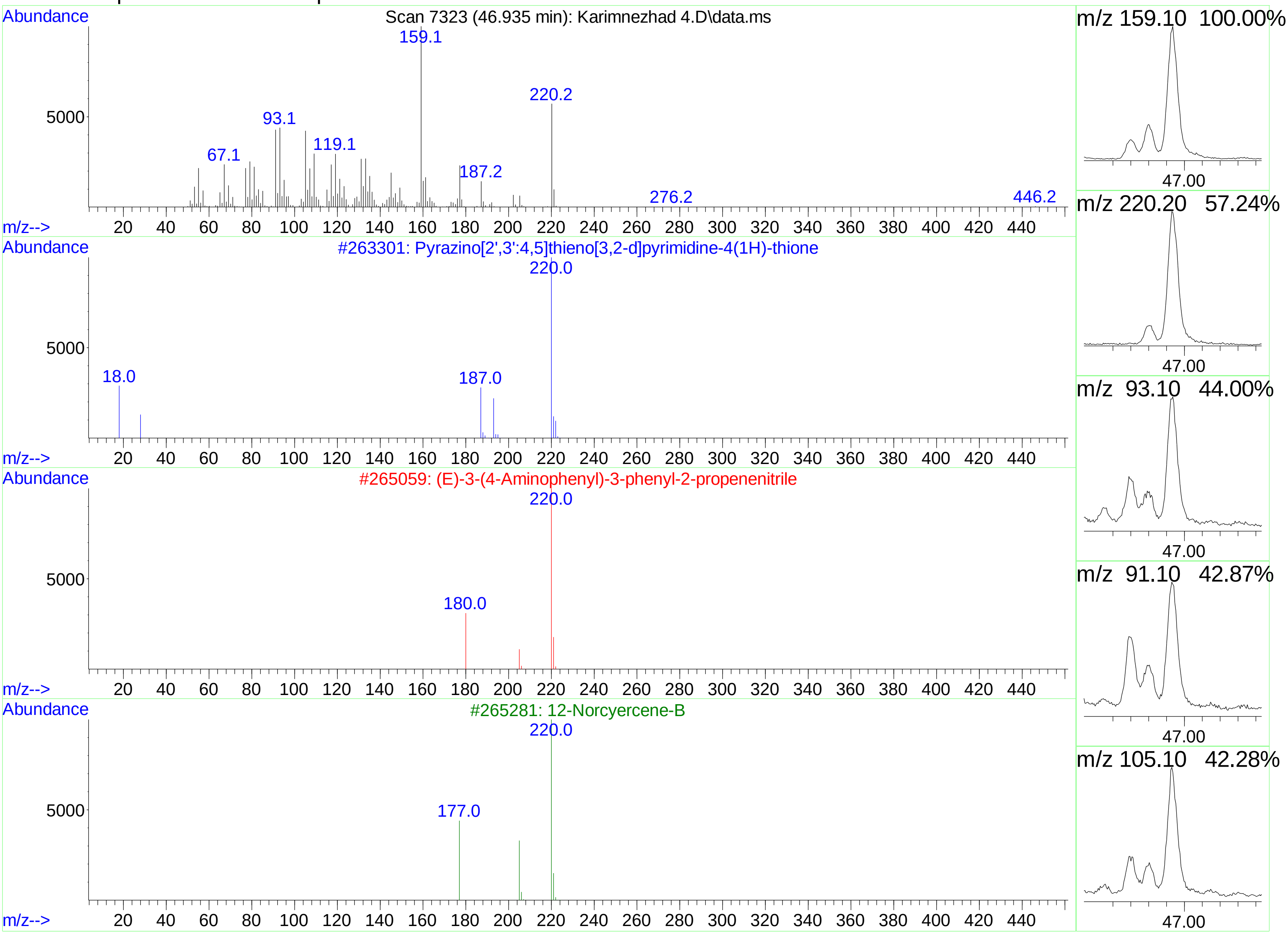

Data File: D:\msdchem\1\data\Karimnezhad 4.D
Sample : M14

Peak Number: 75 at 46.935 min Area: 42067013 Area % 0.18

The 3 best hits from each library. Ref\# CAS\# Qual

D:\Database\W10N14.L

1	Pyrazino[2',3':4,5]thieno[3,2-d]...	263301	056881-25-5	83
2	(E)-3-(4-Aminophenyl)-3-phenyl-2...	265059	2000265-05-9	83
3	12-Norcyercene-B	265281	2000265-28-1	83

Unknown Spectrum based on Apex

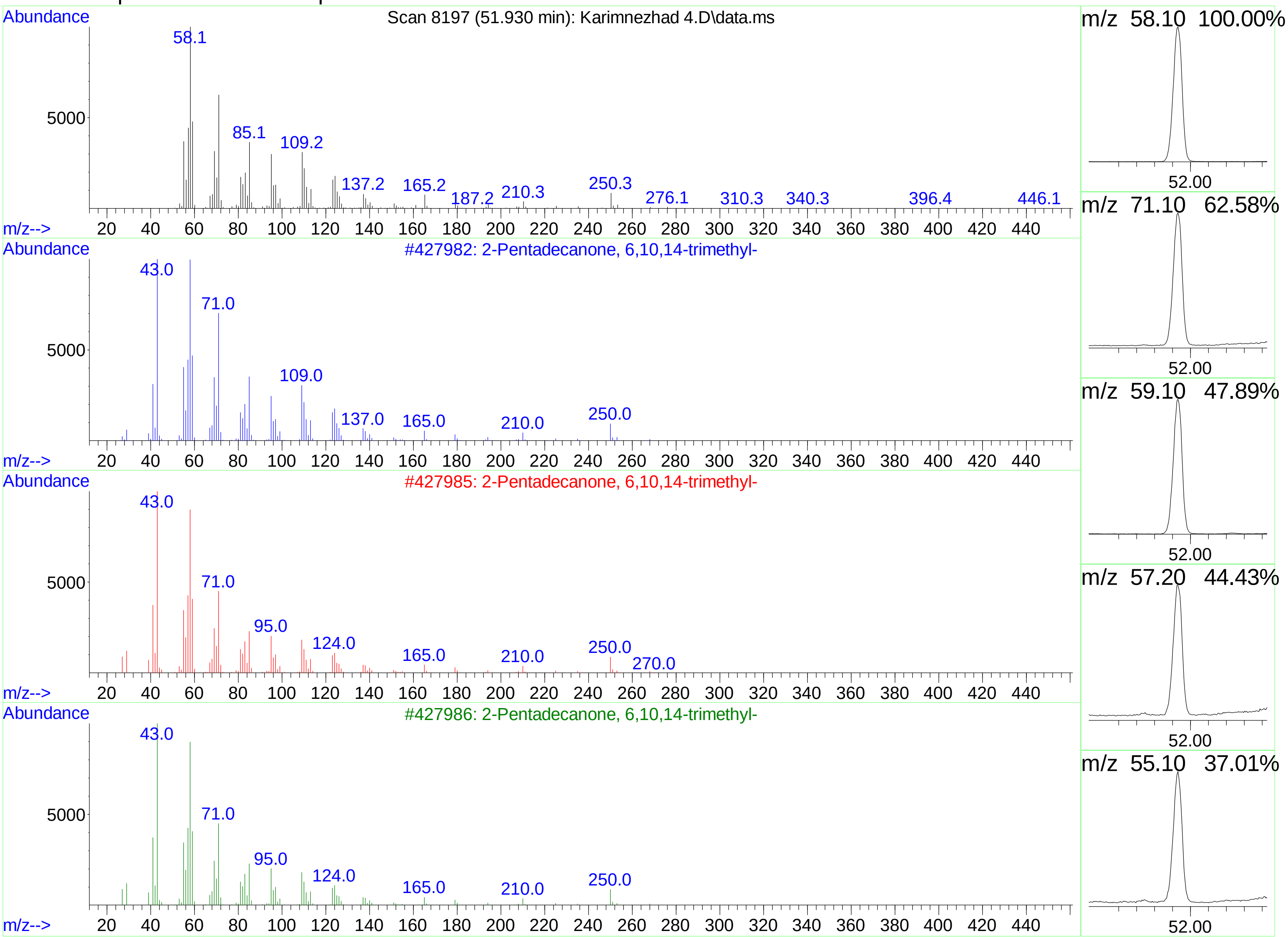

Data File: D:\msdchem\1\data\Karimnezhad 4.D
Sample : M14

Peak Number: 76 at 51.930 min Area: 78913812 Area % 0.34

The 3 best hits from each library.	Ref\#	CAS\#	Qual
D:\Database\W10N14.L			
1 2-Pentadecanone, 6,10,14-trimethyl-	427982	000502-69-2	99
2 2-Pentadecanone, 6,10,14-trimethyl-	427985	000502-69-2	95
3 2-Pentadecanone, 6,10,14-trimethyl-	427986	000502-69-2	95

Library Search Report - ChemStation Integrator

Unknown Spectrum based on Apex

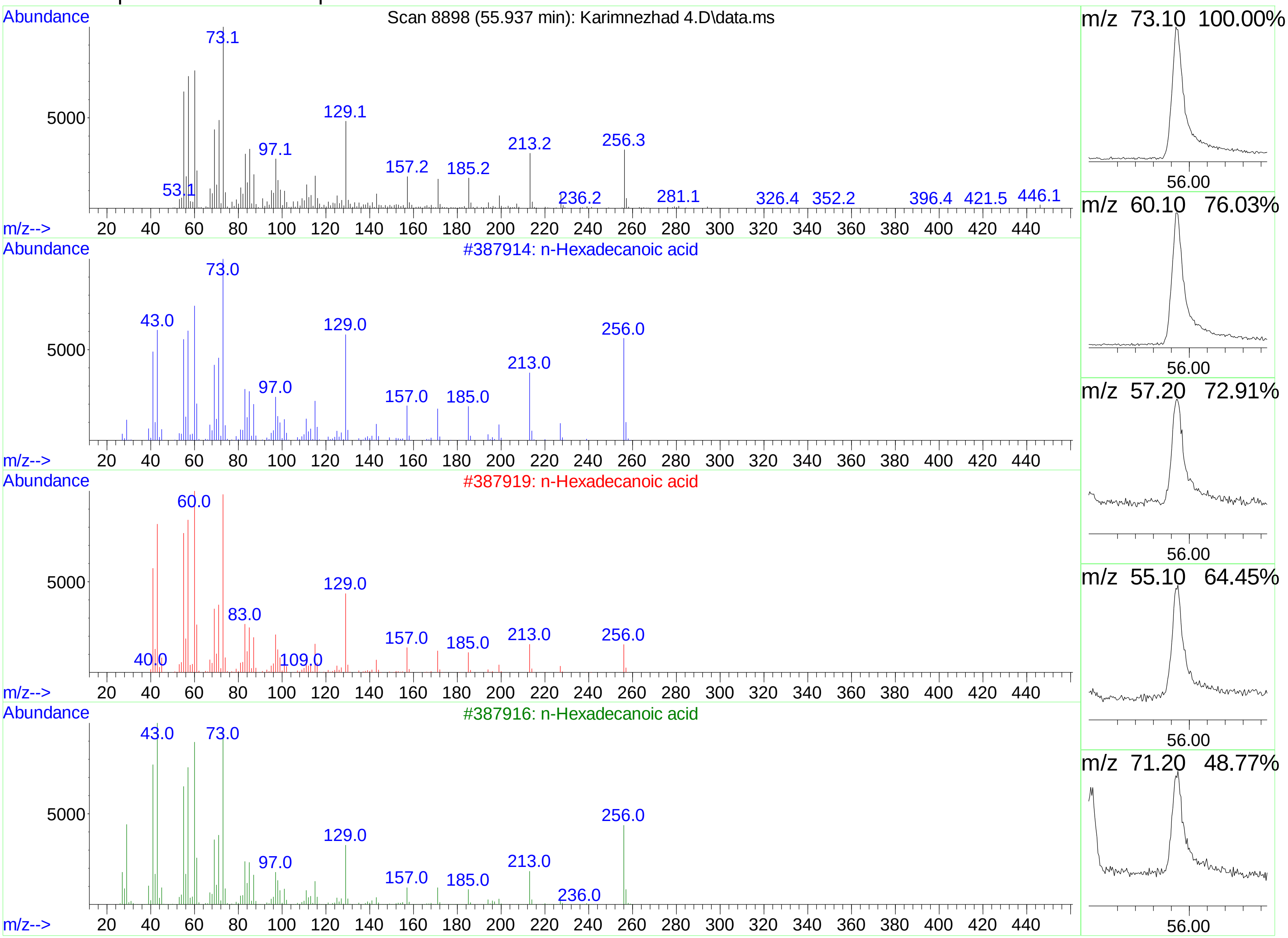

Data File: D:\msdchem\1\data\Karimnezhad 4.D
Sample : M14

Peak Number: 77 at 55.937 min Area: 23244824 Area % 0.10

The 3 best hits from each library.	Ref#	CAS#	Qual
D:\Database\W10N14.L			
1 n-Hexadecanoic acid	387914	000057-10-3	99
2 n-Hexadecanoic acid	387919	000057-10-3	99
3 n-Hexadecanoic acid	387916	000057-10-3	97

Unknown Spectrum based on Apex

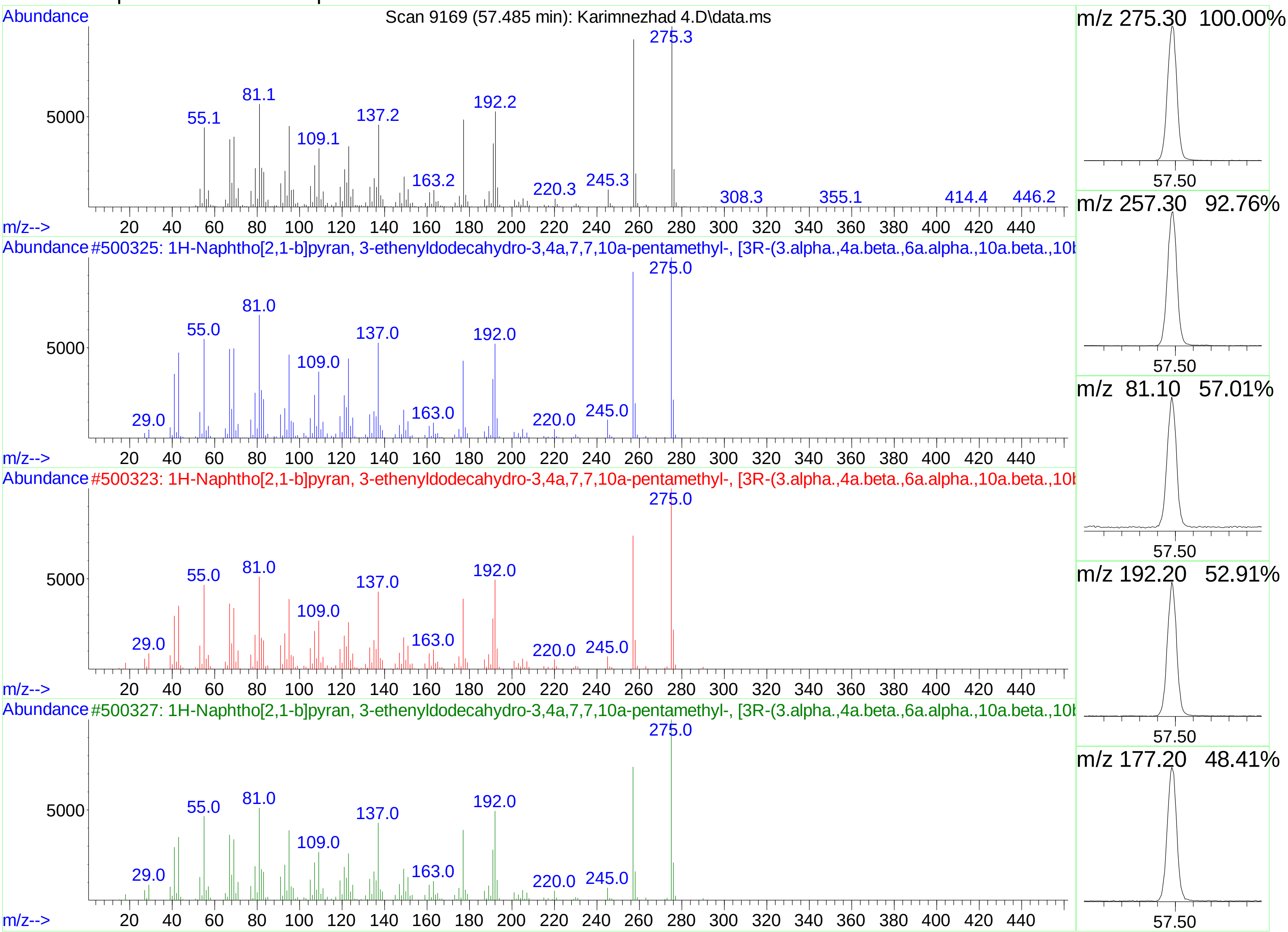

Data File: D:\msdchem\1\data\Karimnezhad 4.D
Sample : M14

Peak Number: 78 at 57.485 min Area: 73298527 Area % 0.32

The 3 best hits from each library.	Ref#	CAS#	Qual
D:\Database\W10N14.L			
1 1H-Naphtho[2,1-b]pyran, 3-etheny...	500325	000596-84-9	99
2 1H-Naphtho[2,1-b]pyran, 3-etheny...	500323	000596-84-9	91
3 1H-Naphtho[2,1-b]pyran, 3-etheny...	500327	000596-84-9	91
