# Supplementary material for: Comparison of quantity, quality and antibacterial activity of essential oil Mentha longifolia (L.) L. under different traditional and modern extraction methods
Source: PLoS One. 2024 Jul 10;19(7):e0301558. doi: 10.1371/journal.pone.0301558 (PMC11236116; doi:10.1371/journal.pone.0301558)
Supplement: S2 File — (ZIP) [file pone.0301558.s002.zip › Karimnezhad/M15/PrintText+summery.pdf]

Library Search Report

Data Path : D:\msdchem\1\data\
Data File : Karimnezhad 5.D
Acq On : 15 Mar 2022 11:23
Operator : Jafari
Sample : M15
Misc :
ALS Vial : 33 Sample Multiplier: 1

Search Libraries: D:\Database\W10N14.L Minimum Quality: 0

Unknown Spectrum: Apex
Integration Events: ChemStation Integrator - events.e

Pk#	RT	Area%	Library/ID	Ref#	CAS#	Qual
1	15.192	0.12	D:\Database\W10N14.L			
			1-Octen-3-ol	37097	003391-86-4	90
			1 OCTEN 3 OL	37089	003391-86-4	90
			1-Octen-3-ol	37088	003391-86-4	90
2	15.981	0.25	D:\Database\W10N14.L			
			3-Octanol	40543	000589-98-0	86
			3-Octanol	40544	000589-98-0	86
			3-Octanol	40529	000589-98-0	86
3	17.833	1.38	D:\Database\W10N14.L			
			2-Oxabicyclo[2.2.2]octane, 1,3,3-trimethyl-	83024	000470-82-6	98
			2-Oxabicyclo[2.2.2]octane, 1,3,3-trimethyl-	83030	000470-82-6	98
			2-Oxabicyclo[2.2.2]octane, 1,3,3-trimethyl-	83026	000470-82-6	95
4	19.667	0.33	D:\Database\W10N14.L			
			Bicyclo[3.1.0]hexan-2-ol, 2-methyl-5-(1-methylethyl)-, (1.alpha.,2.alpha.,5.alpha.)-	83607	017699-16-0	98
			p-Menth-8-en-1-ol, stereoisomer	83201	007299-40-3	94
			4-Thujanol, stereoisomer	83602	017699-16-0	93
5	21.182	0.19	D:\Database\W10N14.L			
			Bicyclo[3.1.0]hexan-2-ol, 2-methyl-5-(1-methylethyl)-, (1.alpha.,2.alpha.,5.alpha.)-	83607	017699-16-0	98
			p-Menth-8-en-1-ol, stereoisomer	83201	007299-40-3	76
			5-ISOPROPYL-2-METHYLBICYCLO[3.1.0]HEXAN-2-OL	83615	015826-82-1	55
6	23.079	0.12	D:\Database\W10N14.L			
			1,3-Cyclohexadiene, 2-methyl-5-(1-methylethyl)-, monoepoxide	77483	072138-69-3	95
			Bicyclo[3.1.0]hexan-3-ol, 4-methylene-1-(1-methylethyl)-, (1.alpha.,3.alpha.,5.alpha.)-	78251	003310-02-9	74
			4(10)-Thujen-3-ol, (1S,3R,5S)-(+)-	78261	000471-16-9	72
7	23.216	0.23	D:\Database\W10N14.L			
			2(10)-Pinen-3-ol, (1S,3R,5S)-(-)-	78358	000547-61-5	93

Data Path : D:\msdchem\1\data\
Data File : Karimnezhad 5.D
Acq On : 15 Mar 2022 11:23
Operator : Jafari
Sample : M15
Misc :
ALS Vial : 33 Sample Multiplier: 1

Search Libraries: D:\Database\W10N14.L Minimum Quality: 0

Unknown Spectrum: Apex
Integration Events: ChemStation Integrator - events.e

Pk# RT Area% Library/ID Ref# CAS# Qual

Bicyclo[3.1.1]heptan-3-ol, 6,6-dim 78361 000547-61-5 87
ethyl-2-methylene-, [1S-(1.alpha.,
3.alpha.,5.alpha.)]-
Bicyclo[3.1.1]heptan-3-ol, 6,6-dim 78364 000547-61-5 83
ethyl-2-methylene-, [1S-(1.alpha.,
3.alpha.,5.alpha.)]-

8 23.388 0.22 D:\Database\W10N14.L
trans-Verbenol 77673 001820-09-3 96
2-Pinen-4-ol 77674 000473-67-6 50
Verbenol 77671 000473-67-6 50

9 23.554 0.27 D:\Database\W10N14.L
1(2H)-Naphthalenone, 3,4,5,6,7,8-h 104860 059177-21-8 83
exahydro-7-methyl-
Benzaldehyde, 4-hydroxy-3-(1-methy 103934 126274-94-0 83
lethyl)-
1-(4-Ethyl-2-hydroxyphenyl)ethanon 103396 2000103-39-6 83

10 23.839 0.19 D:\Database\W10N14.L
2-ISOPROPYL-5-METHYLCYCLOHEXANONE 83421 014073-97-3 98
Cyclohexanone, 5-methyl-2-(1-methy 83410 000089-80-5 98
lethyl)-, trans-
2-ISOPROPYL-5-METHYLCYCLOHEXANONE 83413 000089-80-5 98

11 24.291 0.40 D:\Database\W10N14.L
L-MENTHONE 83439 010458-14-7 98
Cyclohexanone, 5-methyl-2-(1-methy 83410 000089-80-5 98
lethyl)-, trans-
Cyclohexanone, 5-methyl-2-(1-methy 83396 000491-07-6 98
lethyl)-, cis-

12 24.611 0.42 D:\Database\W10N14.L
Cyclohexanemethanol, .alpha.,.alph 83142 007299-42-5 90
a.-dimethyl-4-methylene-
3-Cyclohexene-1-methanol, .alpha., 82891 010482-56-1 53
.alpha.,4-trimethyl-
.alpha.-Terpineol 82900 000098-55-5 50

13 24.788 1.79 D:\Database\W10N14.L
1,7,7-TRIMETHYLBICYCLO[2.2.1]HEPTA 84051 000464-45-9 94
N-2-OL
endo-Borneol 84059 000507-70-0 94

Data Path : D:\msdchem\1\data\
Data File : Karimnezhad 5.D
Acq On : 15 Mar 2022 11:23
Operator : Jafari
Sample : M15
Misc :
ALS Vial : 33 Sample Multiplier: 1

Search Libraries: D:\Database\W10N14.L Minimum Quality: 0

Unknown Spectrum: Apex
Integration Events: ChemStation Integrator - events.e

Pk# RT Area% Library/ID Ref# CAS# Qual

Bicyclo[2.2.1]heptan-2-ol, 1,7,7-t 84056 000464-45-9 87
rimethyl-, (1S-endo)-

14 25.091 0.59 D:\Database\W10N14.L
1-ISOPROPYL-4-METHYL-3-CYCLOHEXEN- 82925 000562-74-3 98
1-OL
3-Cyclohexen-1-ol, 4-methyl-1-(1-m 82943 000562-74-3 98
ethylethyl)-
3-Cyclohexen-1-ol, 4-methyl-1-(1-m 82938 000562-74-3 97
ethylethyl)-

15 25.445 0.37 D:\Database\W10N14.L
PARA-CYMEN-8-OL 72711 001197-01-9 93
Benzenemethanol, 4-(1-methylethyl) 73095 000536-60-7 86
3-Methyl-6-hydroxybenzo[c]-dihydro 72145 2000072-14-5 83
furan

16 25.805 0.67 D:\Database\W10N14.L
Cyclohexene, 1-methyl-3-(1-methyle 48587 000499-03-6 89
thenyl)-, (.+-.)-
Tricyclo[2.2.1.0(2,6)]heptane, 1,3 48838 000488-97-1 55
,3-trimethyl-
Cyclohexene, 1-methyl-2-(1-methyle 48521 076480-15-4 53
thenyl)-

17 26.028 0.15 D:\Database\W10N14.L
(-)-trans-Isopiperitenol 78055 074410-00-7 95
2-Cyclohexen-1-ol, 3-methyl-6-(1-m 78051 000491-05-4 95
ethylethenyl)-
(-)-cis-Isopiperitenol 78050 096555-02-1 83

18 26.371 0.13 D:\Database\W10N14.L
1-Indanone, 4,5,6,7-tetrahydro-3-m 72733 018631-68-0 83
ethyl-
2,5-Methano-1H-inden-7(4H)-one, he 73572 027567-85-7 72
xahydro-
(E)-3-Methyl-4-propylidene-2-cyclo 72980 2000072-98-0 68
hexen-1-one

19 26.851 0.79 D:\Database\W10N14.L
3-Heptene, 2,2,3,5,6-pentamethyl- 116649 116164-06-8 41
1,2,3,5-tetramethylcyclohexane (1r 55961 019899-28-6 38
,2t,3c,5c)

Data Path : D:\msdchem\1\data\
Data File : Karimnezhad 5.D
Acq On : 15 Mar 2022 11:23
Operator : Jafari
Sample : M15
Misc :
ALS Vial : 33 Sample Multiplier: 1

Search Libraries: D:\Database\W10N14.L Minimum Quality: 0

Unknown Spectrum: Apex
Integration Events: ChemStation Integrator - events.e

Pk#	RT	Area%	Library/ID	Ref#	CAS#	Qual
			1,3-Cyclohexanedione, 5-isopropyl-	82473	018456-87-6	38
20	27.183	2.41	D:\Database\W10N14.L			
			8,9-Dehydrothymol	68975	018612-99-2	95
			1-methoxy-4-(1-methylethenyl)benzene	68640	2000068-64-0	81
			1(S)-Methyl-2(R)-phenylcyclopropanol	68865	2000068-86-5	72
21	27.994	10.81	D:\Database\W10N14.L			
			Pulegone	78101	000089-82-7	97
			Cyclohexanone, 5-methyl-2-(1-methylethylidene)-	78096	015932-80-6	96
			(R)-5-methyl-2-(1-methylethylidene)-cyclohexanone	78102	000089-82-7	96
22	28.406	0.76	D:\Database\W10N14.L			
			7-Oxabicyclo[4.1.0]heptan-2-one, 6-methyl-3-(1-methylethylidene)-	114758	035178-55-3	97
			4-ISOPROPENYL-1-METHYL-7-OXABICYCLO[4.1.0]HEPTAN-2-ONE	114757	035178-55-3	49
			1,2,3,5-tetramethylcyclohexane (1r,2t,3t,5c)	55964	019899-29-7	43
23	28.572	1.71	D:\Database\W10N14.L			
			7-Oxabicyclo[4.1.0]heptan-2-one, 6-methyl-3-(1-methylethyl)-	115503	005286-38-4	76
			7-Oxabicyclo[4.1.0]heptan-2-one, 6-methyl-3-(1-methylethylidene)-	114758	035178-55-3	76
			4-ISOPROPENYL-1-METHYL-7-OXABICYCLO[4.1.0]HEPTAN-2-ONE	114757	035178-55-3	60
24	28.766	0.28	D:\Database\W10N14.L			
			3a,4,5,6,7,7a-hexahydro-4,7-methanobenz[d]isoxazole	49858	015166-80-0	49
			1,1'-Bicyclopentyl	52365	001636-39-1	41
			1,1'-Bicyclopentyl	52366	001636-39-1	38
25	28.875	0.14	D:\Database\W10N14.L			
			HERBOXIDE SECOND ISOMER	77558	013679-86-2	43
			1-BUTYL-2-ETHYL-1-CYCLOPROPENE	31910	050915-91-8	30
			Cyclopropene, 1-butyl-2-ethyl-	31911	050915-91-8	30

Data Path : D:\msdchem\1\data\
Data File : Karimnezhad 5.D
Acq On : 15 Mar 2022 11:23
Operator : Jafari
Sample : M15
Misc :
ALS Vial : 33 Sample Multiplier: 1

Search Libraries: D:\Database\W10N14.L Minimum Quality: 0

Unknown Spectrum: Apex
Integration Events: ChemStation Integrator - events.e

Pk#	RT	Area%	Library/ID	Ref#	CAS#	Qual
26	29.029	0.19	D:\Database\W10N14.L			
			4-Fluoro-2-acetylphenol	81032	000394-32-1	86
			5-Fluoro-2-hydroxyacetophenone	81029	000394-32-1	74
			3-Acetyl-2,5-dimethylthiophene	81077	002530-10-1	72
27	29.292	1.24	D:\Database\W10N14.L			
			(S)-(+)-cis-Isopiperitenone	72898	2000072-89-8	91
			1,8-(p-MENTHADIENONE)	72899	2000072-89-9	72
			2-Cyclohexen-1-one, 3,5,5-trimethy l-	51811	000078-59-1	50
28	29.680	0.30	D:\Database\W10N14.L			
			4-(Methoxymethyl)benzenaldehyde	72078	2000072-07-8	90
			1(3aH)-Pentalenone, 4,5,6,6a-tetra hydro-2,6a-dimethyl-	72961	070640-02-7	90
			2-Cyclopenten-1-one, 3-ethenyl-2,4 ,4-trimethyl-	72789	104642-15-1	90
29	29.886	0.57	D:\Database\W10N14.L			
			Bicyclo[2.2.1]heptan-2-ol, 1,7,7-t rimethyl-, acetate, (1S-endo)-	192108	005655-61-8	99
			Bicyclo[2.2.1]heptan-2-ol, 1,7,7-t rimethyl-, acetate, (1S-endo)-	192123	005655-61-8	99
			Acetic acid, 1,7,7-trimethyl-bicyc lo[2.2.1]hept-2-yl ester	192116	092618-89-8	99
30	30.252	0.34	D:\Database\W10N14.L			
			Benzene, 1-ethoxy-4-ethyl-	73585	001585-06-4	93
			Benzene, 1-ethoxy-4-ethyl- (CAS)	73584	001585-06-4	93
			2,4-Cycloheptadien-1-one, 2,6,6-tr imethyl-	73011	000503-93-5	70
31	31.052	0.37	D:\Database\W10N14.L			
			Phenol, 5-methyl-2-(1-methylethyl)	73199	000089-83-8	95
			Phenol, 5-methyl-2-(1-methylethyl)	73197	000089-83-8	94
			Thymol	73186	000089-83-8	94
32	31.515	0.42	D:\Database\W10N14.L			
			Carvacrol	72969	000499-75-2	95
			Carvacrol	72968	000499-75-2	94
			Phenol, 5-methyl-2-(1-methylethyl)	73197	000089-83-8	91
33	33.035	38.41	D:\Database\W10N14.L			

Data Path : D:\msdchem\1\data\
Data File : Karimnezhad 5.D
Acq On : 15 Mar 2022 11:23
Operator : Jafari
Sample : M15
Misc :
ALS Vial : 33 Sample Multiplier: 1

Search Libraries: D:\Database\W10N14.L Minimum Quality: 0

Unknown Spectrum: Apex
Integration Events: ChemStation Integrator - events.e

Pk#	RT	Area%	Library/ID	Ref#	CAS#	Qual
			2-Cyclohexen-1-one, 3-methyl-6-(1-methylethylidene)-	73417	000491-09-8	98
			2-Cyclohexen-1-one, 3-methyl-6-(1-methylethylidene)-	73419	000491-09-8	95
			4,7,7-Trimethylbicyclo[4.1.0]hept-3-en-2-one	72780	081800-50-2	93
34	33.910	21.26	D:\Database\W10N14.L			
			PIPERITENONE OXIDE	110057	003564-96-3	98
			4-Acetyl-1-methylcyclohexene	51791	006090-09-1	60
			2,4-Heptadienal, 2-methyl-6-oxo-, (E,E)-	51105	129454-99-5	58
35	34.087	0.15	D:\Database\W10N14.L			
			Benzoic acid, 4-methoxy-, methyl e ster	108601	000121-98-2	60
			Benzoic acid, 4-methoxy-, methyl e ster	108599	000121-98-2	60
			METHYL-4-METHOXYBENZOATE	108595	000121-98-2	60
36	34.253	0.29	D:\Database\W10N14.L			
			2',6'-Dihydroxy-3'-methylacetophen one	108340	029183-78-6	87
			(5S*,6R*)-5,6-Dimethyl-3,4,5,6-tet rahydro-2H-cyclopenta[b]pyran-7-on	110048	2000110-04-8	83
			5,6-Dimethyl-3,4,5,6-tetrahydro-2H -cyclopenta[b]pyran-7-one	110049	2000110-04-9	83
37	34.584	0.17	D:\Database\W10N14.L			
			.BETA. BOURBONENE	215702	005208-59-3	99
			(-)-.beta.-Bourbonene	215705	005208-59-3	98
			(-)-.beta.-Bourbonene	215706	005208-59-3	97
38	34.836	0.18	D:\Database\W10N14.L			
			2-Cyclopenten-1-one, 3-methyl-2-(2 -pentenyl)-, (Z)-	104676	000488-10-8	99
			3-METHYL-2-PENT-2-ENYL-CYCLOPENT-2 -ENONE	104678	000488-10-8	98
			2-Cyclopenten-1-one, 3-methyl-2-(2 -pentenyl)-, (Z)-	104680	000488-10-8	98
39	35.116	0.84	D:\Database\W10N14.L			
			4,6-DIETHYL-2-METHOXYPYRIMIDINE	110182	2000110-18-2	72

Data Path : D:\msdchem\1\data\
Data File : Karimnezhad 5.D
Acq On : 15 Mar 2022 11:23
Operator : Jafari
Sample : M15
Misc :
ALS Vial : 33 Sample Multiplier: 1

Search Libraries: D:\Database\W10N14.L Minimum Quality: 0

Unknown Spectrum: Apex
Integration Events: ChemStation Integrator - events.e

Pk# RT Area% Library/ID Ref# CAS# Qual

2(1H)-Pyrimidinone, 5-acetyl-4-met 109135 093584-03-3 64
hyl-, 2-hydrazone
3-Hydroxy-4-methoxy-5-methylbenzal 108182 2000108-18-2 64
dehyde

40 36.162 2.04 D:\Database\W10N14.L
Caryophyllene 216361 000087-44-5 99
TRANS(.BETA.)-CARYOPHYLLENE 216339 2000216-33-9 99
Bicyclo[7.2.0]undec-4-ene, 4,11,11 216351 000087-44-5 99
-trimethyl-8-methylene-, (E)-(1R,9
S)-(-)-

41 36.956 0.13 D:\Database\W10N14.L
5,9-Undecadien-2-one, 6,10-dimethy 186308 003796-70-1 93
l-, (E)-
5,9-Undecadien-2-one, 6,10-dimethy 186309 003796-70-1 86
l-, (E)-
5,9-Undecadien-2-one, 6,10-dimethy 186310 003796-70-1 86
l-, (E)-

42 37.162 0.33 D:\Database\W10N14.L
(E)-.beta.-Famesene 216558 018794-84-8 96
trans-.beta.-Farnesene 216566 000502-60-3 96
(1S,5S,6R)-6-Methyl-2-methylene-6- 216863 015438-94-5 95
(4-methylpent-3-en-1-yl)bicyclo[3.
1.1]heptane

43 37.642 0.34 D:\Database\W10N14.L
.alpha.-Humulene 216788 006753-98-6 99
.alpha.-Humulene 216789 006753-98-6 98
.alpha.-Humulene 216795 006753-98-6 98

44 38.048 0.14 D:\Database\W10N14.L
1,3-Hexadiene, 4-chloro-2,3-dimeth 61659 105949-72-2 60
yl-, (E)-
1,3-Hexadiene, 4-chloro-2,3-dimeth 61661 105977-13-7 53
yl-, (Z)-
(Z)-4-Chloro-2,3-dimethyl-1,3-hexa 61660 105977-13-7 49
diene

45 38.408 0.11 D:\Database\W10N14.L
trans-.beta.-Ionone 179342 000079-77-6 97
3-Buten-2-one, 4-(2,6,6-trimethyl- 179341 014901-07-6 97

Data Path : D:\msdchem\1\data\
Data File : Karimnezhad 5.D
Acq On : 15 Mar 2022 11:23
Operator : Jafari
Sample : M15
Misc :
ALS Vial : 33 Sample Multiplier: 1

Search Libraries: D:\Database\W10N14.L Minimum Quality: 0

Unknown Spectrum: Apex
Integration Events: ChemStation Integrator - events.e

```
Pk#   RT  Area%        Library/ID              Ref#    CAS#   Qual
______________________________________________________________________
              1-cyclohexen-1-yl)-
              trans-.beta.-Ionone              179340 000079-77-6 96

 46  38.676  0.81 D:\Database\W10N14.L
              1H-Cyclopenta[1,3]cyclopropa[1,2]b 216768 013744-15-5 99
              enzene, 2,3,3a.alpha.,3b.alpha.,4,
              5,6,7-octahydro-4.alpha.-isopropyl
              -7.beta.-methyl-3-methylene-
              Germacrene D                     216742 023986-74-5 99
              8-ISOPROPYL-1-METHYL-5-METHYLENE-1 216746 023986-74-5 98
              ,6-CYCLODECADIENE

 47  39.111  0.12 D:\Database\W10N14.L
              (1S,6R)-3,7,7-Trimethylbicyclo[4.1 104284 2000104-28-4 90
              .0]hept-3-en-2,5-dione
              4'-(1-Fluorovinyl)acetophenone     103142 2000103-14-2 83
              3-tert-Butylanisole              105065 2000105-06-5 83

 48  39.265  0.20 D:\Database\W10N14.L
              Bicyclo[8.1.0]undeca-2,6-diene, 3, 215949 100762-46-7 99
              7,11,11-tetramethyl-, (1R*,2Z,6E,1
              0R*)-(.+-.)-
              (1S,2E,6E,10R)-3,7,11,11-Tetrameth 215953 024703-35-3 97
              ylbicyclo[8.1.0]undeca-2,6-diene
              Bicyclo[8.1.0]undeca-2,6-diene, 3, 215948 100762-46-7 97
              7,11,11-tetramethyl-, (1R*,2Z,6E,1
              0R*)-(.+-.)-

 49  42.111  0.75 D:\Database\W10N14.L
              Cyclohexanecarboxylic acid, 1-meth 156877 005453-94-1 83
              yl-2-oxo-, ethyl ester
              3-Chloro-4-t-butyl-6-methylpyridaz 155956 2000155-95-6 83
              ine
              2-Methylthiocyclohexa[c]thiophene  155216 000000-00-0 64

 50  42.648  0.85 D:\Database\W10N14.L
              1,1,7-TRIMETHYL-4-METHYLENEDECAHYD 267795 077171-55-2 98
              RO-1H-CYCLOPROPA[E]AZULEN-7-OL
              1H-Cycloprop[e]azulen-7-ol, decahy 267797 006750-60-3 98
              dro-1,1,7-trimethyl-4-methylene-,
              [1ar-(1a.alpha.,4a.alpha.,7.beta.,
              7a.beta.,7b.alpha.)]-
              (-)-Spathulenol                  267796 077171-55-2 95
```

Data Path : D:\msdchem\1\data\
Data File : Karimnezhad 5.D
Acq On : 15 Mar 2022 11:23
Operator : Jafari
Sample : M15
Misc :
ALS Vial : 33 Sample Multiplier: 1

Search Libraries: D:\Database\W10N14.L Minimum Quality: 0

Unknown Spectrum: Apex
Integration Events: ChemStation Integrator - events.e

Pk#	RT	Area%	Library/ID	Ref#	CAS#	Qual
51	42.900	2.99	D:\Database\W10N14.L			
			(-)-5-Oxatricyclo[8.2.0.0(4,6)]dodecane,,12-trimethyl-9-methylene-, [1R-(1R*,4R*,6R*,10S*)]-	267388	001139-30-6	99
			(-)-5-Oxatricyclo[8.2.0.0(4,6)]dodecane,,12-trimethyl-9-methylene-, [1R-(1R*,4R*,6R*,10S*)]-	267387	001139-30-6	95
			Caryophyllene oxide	267393	001139-30-6	94
52	43.917	0.24	D:\Database\W10N14.L			
			(1R,3E,7E,11R)-1,5,5,8-Tetramethyl-12-oxabicyclo[9.1.0]dodeca-3,7-diene	267258	019888-34-7	99
			(1R,3E,7E,11R)-1,5,5,8-Tetramethyl-12-oxabicyclo[9.1.0]dodeca-3,7-diene	267257	019888-34-7	87
			Naphthalene, decahydro-	52331	000091-17-8	78
53	44.717	0.20	D:\Database\W10N14.L			
			isospathulenol	267807	2000267-80-7	99
			(-)-Spathulenol	267798	077171-55-2	90
			1H-Cycloprop[e]azulen-7-ol, decahydro-1,1,7-trimethyl-4-methylene-	267802	006750-60-3	68
54	44.952	0.29	D:\Database\W10N14.L			
			caryophylla-4(12),8(13)-dien-5.beta.-ol	267136	2000267-13-6	99
			10,10-Dimethyl-2,6-dimethylenebicyclo[7.2.0]undecan-5.beta.-ol	267897	019431-80-2	99
			10,10-Dimethyl-2,6-dimethylenebicyclo[7.2.0]undecan-5.beta.-ol	267895	019431-80-2	98
55	45.603	0.18	D:\Database\W10N14.L			
			(-)-5-Oxatricyclo[8.2.0.0(4,6)]dodecane,,12-trimethyl-9-methylene-, [1R-(1R*,4R*,6R*,10S*)]-	267389	001139-30-6	78
			(1R,9R,E)-4,11,11-Trimethyl-8-methylenebicyclo[7.2.0]undec-4-ene	216344	068832-35-9	70
			3-METHYL-5-(2,6,6-TRIMETHYL-1-CYCLOHEXEN-1-YL)-1-PENTYN-3-OL	267107	2000267-10-7	64
56	46.158	0.31	D:\Database\W10N14.L			

Data Path : D:\msdchem\1\data\
Data File : Karimnezhad 5.D
Acq On : 15 Mar 2022 11:23
Operator : Jafari
Sample : M15
Misc :
ALS Vial : 33 Sample Multiplier: 1

Search Libraries: D:\Database\W10N14.L Minimum Quality: 0

Unknown Spectrum: Apex
Integration Events: ChemStation Integrator - events.e

Pk#	RT	Area%	Library/ID	Ref#	CAS#	Qual
			(-)-5-Oxatricyclo[8.2.0.0(4,6)]dodecane,,12-trimethyl-9-methylene-, [1R-(1R*,4R*,6R*,10S*)]-	267388	001139-30-6	68
			Bicyclo[7.2.0]undec-3-en-5-ol, 4,11,11-trimethyl-8-methylene-, stereoisomer	267841	032214-89-4	60
			2-(trans-2-Methylcyclohexyl)buta-1,3-diene	74040	2000074-04-0	56
57	46.929	0.15	D:\Database\W10N14.L			
			(E)-3-(4-Aminophenyl)-3-phenyl-2-propenenitrile	265059	2000265-05-9	90
			12-Norcyercene-B	265281	2000265-28-1	83
			2,3-Quinoxalinedione, 6,7-diamino-1,4-dihydro-1,4-dimethyl-	264958	2000264-95-8	40
58	51.924	0.18	D:\Database\W10N14.L			
			2-Pentadecanone, 6,10,14-trimethyl	427982	000502-69-2	99
			2-Pentadecanone, 6,10,14-trimethyl	427987	000502-69-2	94
			2-Pentadecanone, 6,10,14-trimethyl	427988	000502-69-2	94
59	57.480	0.21	D:\Database\W10N14.L			
			1H-Naphtho[2,1-b]pyran, 3-ethenyldodecahydro-3,4a,7,7,10a-pentamethyl-, [3R-(3.alpha.,4a.beta.,6a.alpha.,10a.beta.,10b.alpha.)]-	500325	000596-84-9	99
			1H-Naphtho[2,1-b]pyran, 3-ethenyldodecahydro-3,4a,7,7,10a-pentamethyl-, [3R-(3.alpha.,4a.beta.,6a.alpha.,10a.beta.,10b.alpha.)]-	500330	000596-84-9	95
			1H-Naphtho[2,1-b]pyran, 3-ethenyldodecahydro-3,4a,7,7,10a-pentamethyl-, [3R-(3.alpha.,4a.beta.,6a.alpha.,10a.beta.,10b.alpha.)]-	500327	000596-84-9	91
