# Supplementary material for: Comparison of quantity, quality and antibacterial activity of essential oil Mentha longifolia (L.) L. under different traditional and modern extraction methods
Source: PLoS One. 2024 Jul 10;19(7):e0301558. doi: 10.1371/journal.pone.0301558 (PMC11236116; doi:10.1371/journal.pone.0301558)
Supplement: S2 File — (ZIP) [file pone.0301558.s002.zip › Karimnezhad/M15/QualKarimnezhad 5.pdf]

Library Search Report

Data Path : D:\msdchem\1\data\
Data File : Karimnezhad 5.D
Acq On : 15 Mar 2022 11:23
Operator : Jafari
Sample : M15
Misc :
ALS Vial : 33 Sample Multiplier: 1

Search Libraries: D:\Database\W10N14.L Minimum Quality: 0

Unknown Spectrum: Apex
Integration Events: ChemStation Integrator - events.e

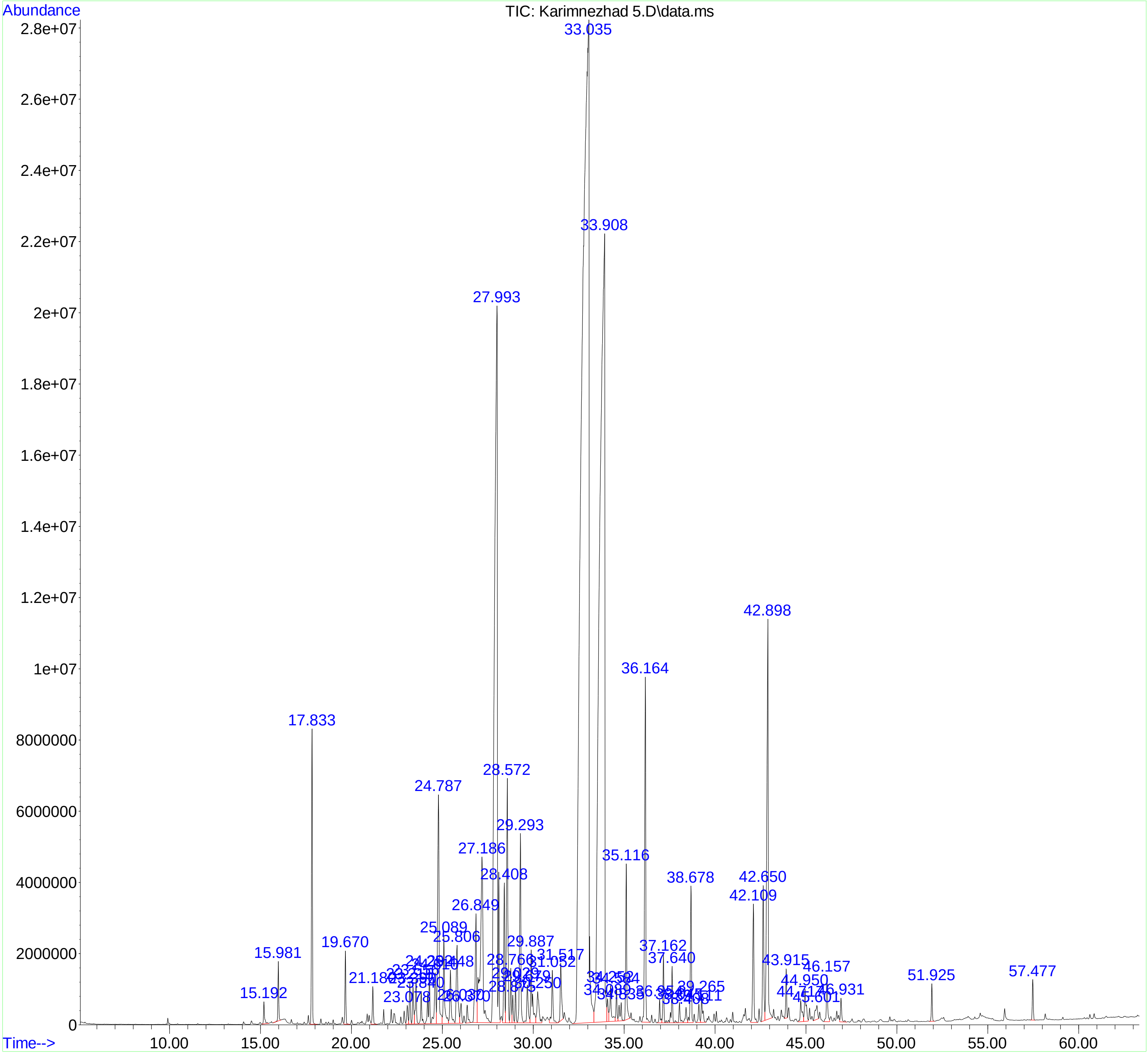

Unknown Spectrum based on Apex

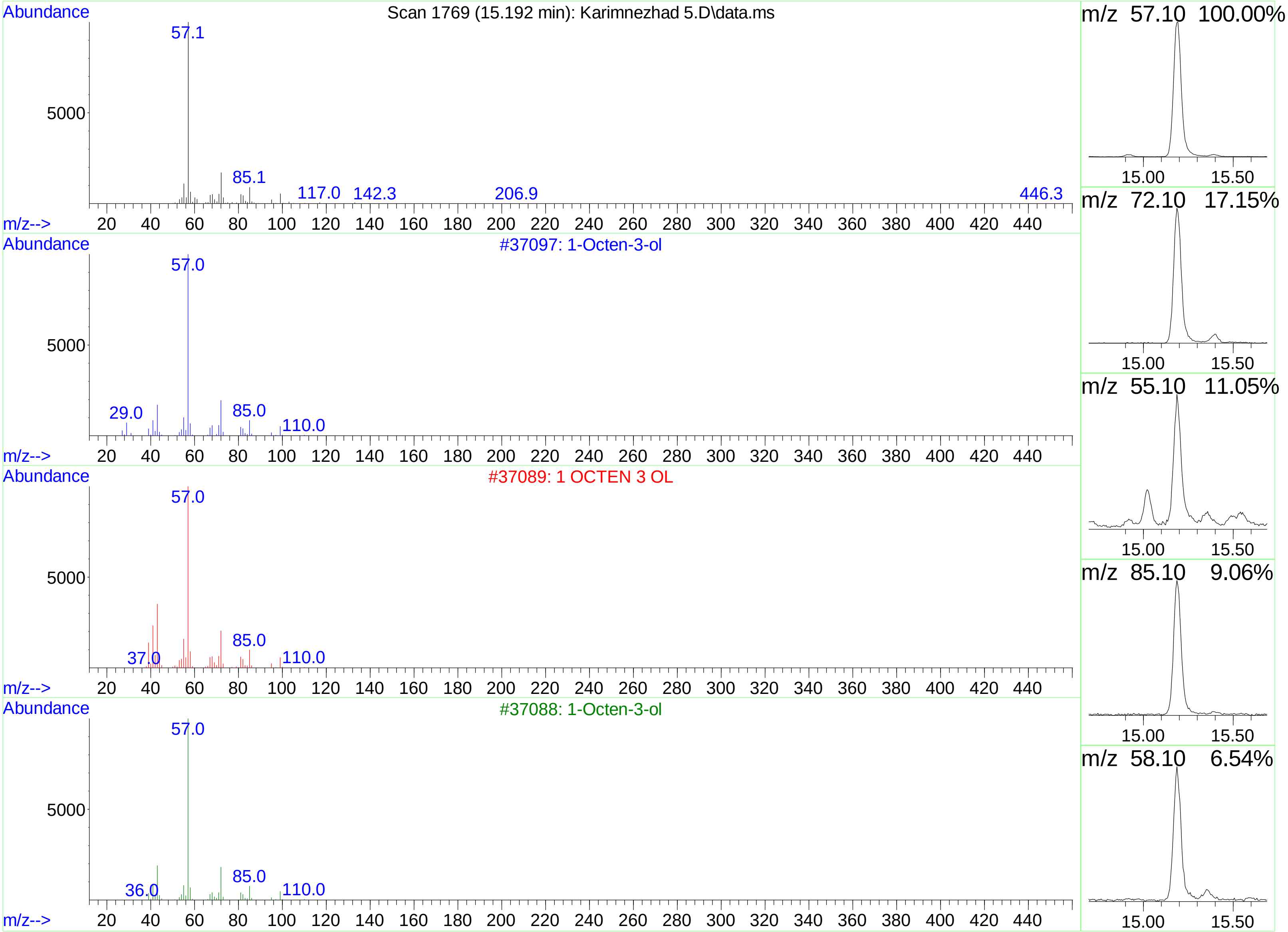


Data File: D:\msdchem\1\data\Karimnezhad 5.D
Sample : M15

Peak Number: 1 at 15.192 min Area: 24035734 Area % 0.12

| The 3 best hits from each library. | Ref\# | CAS\# | Qual |
|---|---|---|---|
| D:\Database\W10N14.L | | | |
| 1 1-Octen-3-ol | 37097 | 003391-86-4 | 90 |
| 2 1 OCTEN 3 OL | 37089 | 003391-86-4 | 90 |
| 3 1-Octen-3-ol | 37088 | 003391-86-4 | 90 |

Unknown Spectrum based on Apex

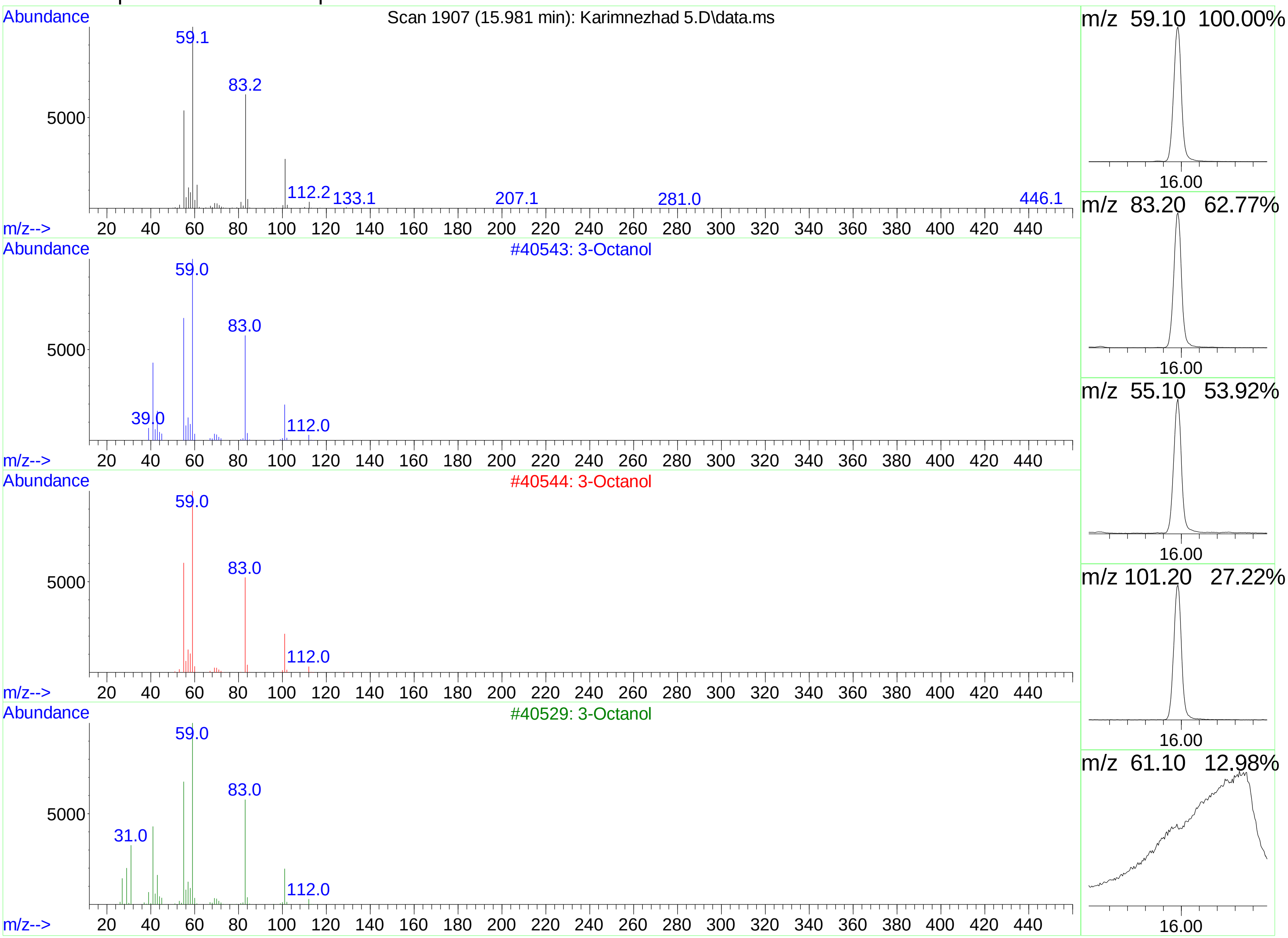


Data File: D:\msdchem\1\data\Karimnezhad 5.D
Sample : M15

Peak Number: 2 at 15.981 min Area: 50498187 Area % 0.25

| The 3 best hits from each library. | Ref\# | CAS\# | Qual |
|---|---|---|---|
| D:\Database\W10N14.L | | | |
| 1 3-Octanol | 40543 | 000589-98-0 | 86 |
| 2 3-Octanol | 40544 | 000589-98-0 | 86 |
| 3 3-Octanol | 40529 | 000589-98-0 | 86 |

# Library Search Report - ChemStation Integrator

Unknown Spectrum based on Apex

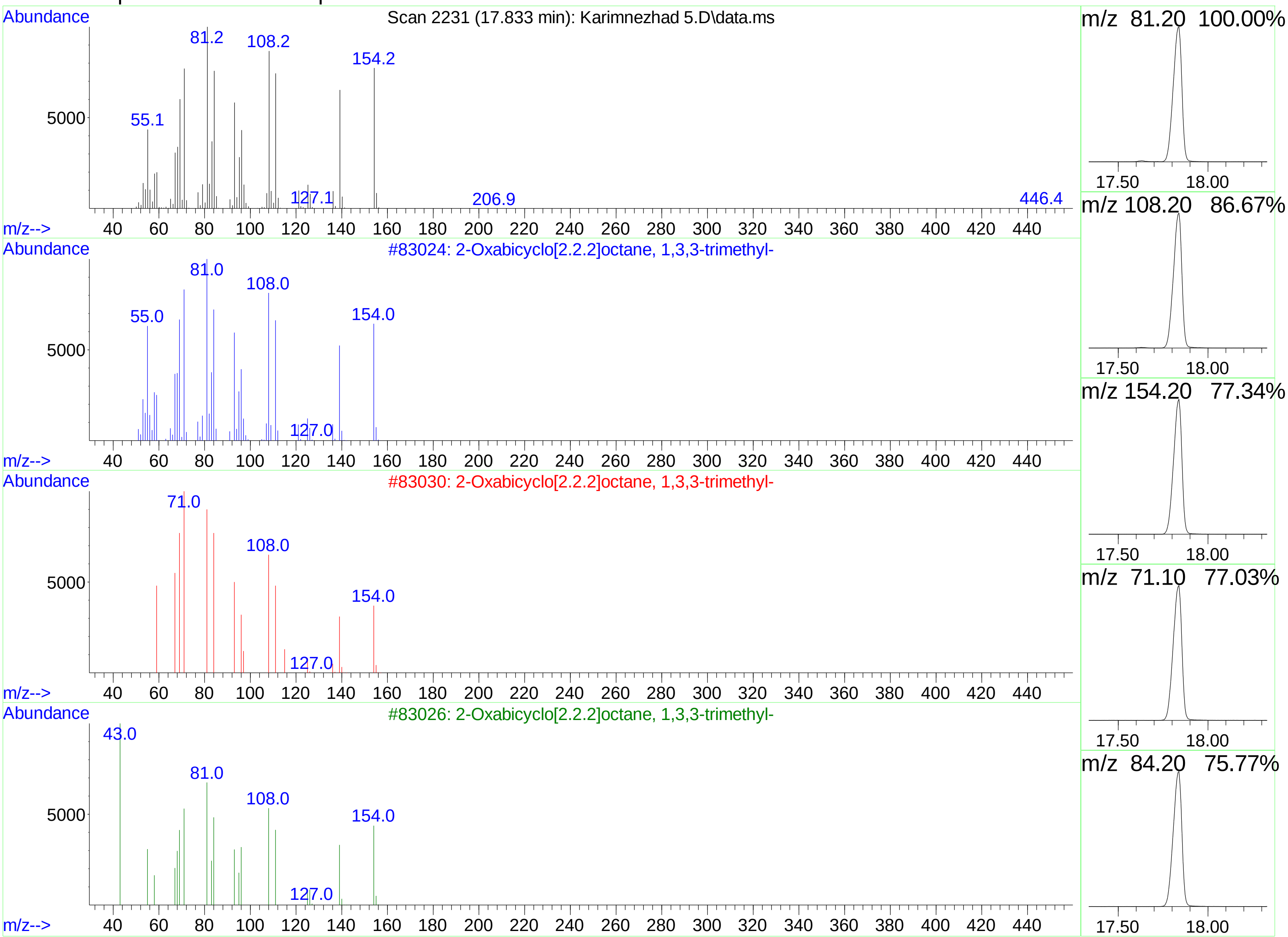


Data File: D:\msdchem\1\data\Karimnezhad 5.D
Sample : M15

Peak Number: 3 at 17.833 min Area: 278947915 Area % 1.38

| The 3 best hits from each library. | Ref# | CAS# | Qual |
|---|---|---|---|
| D:\Database\W10N14.L | | | |
| 1 2-Oxabicyclo[2.2.2]octane, 1,3,3... | 83024 | 000470-82-6 | 98 |
| 2 2-Oxabicyclo[2.2.2]octane, 1,3,3... | 83030 | 000470-82-6 | 98 |
| 3 2-Oxabicyclo[2.2.2]octane, 1,3,3... | 83026 | 000470-82-6 | 95 |

Unknown Spectrum based on Apex

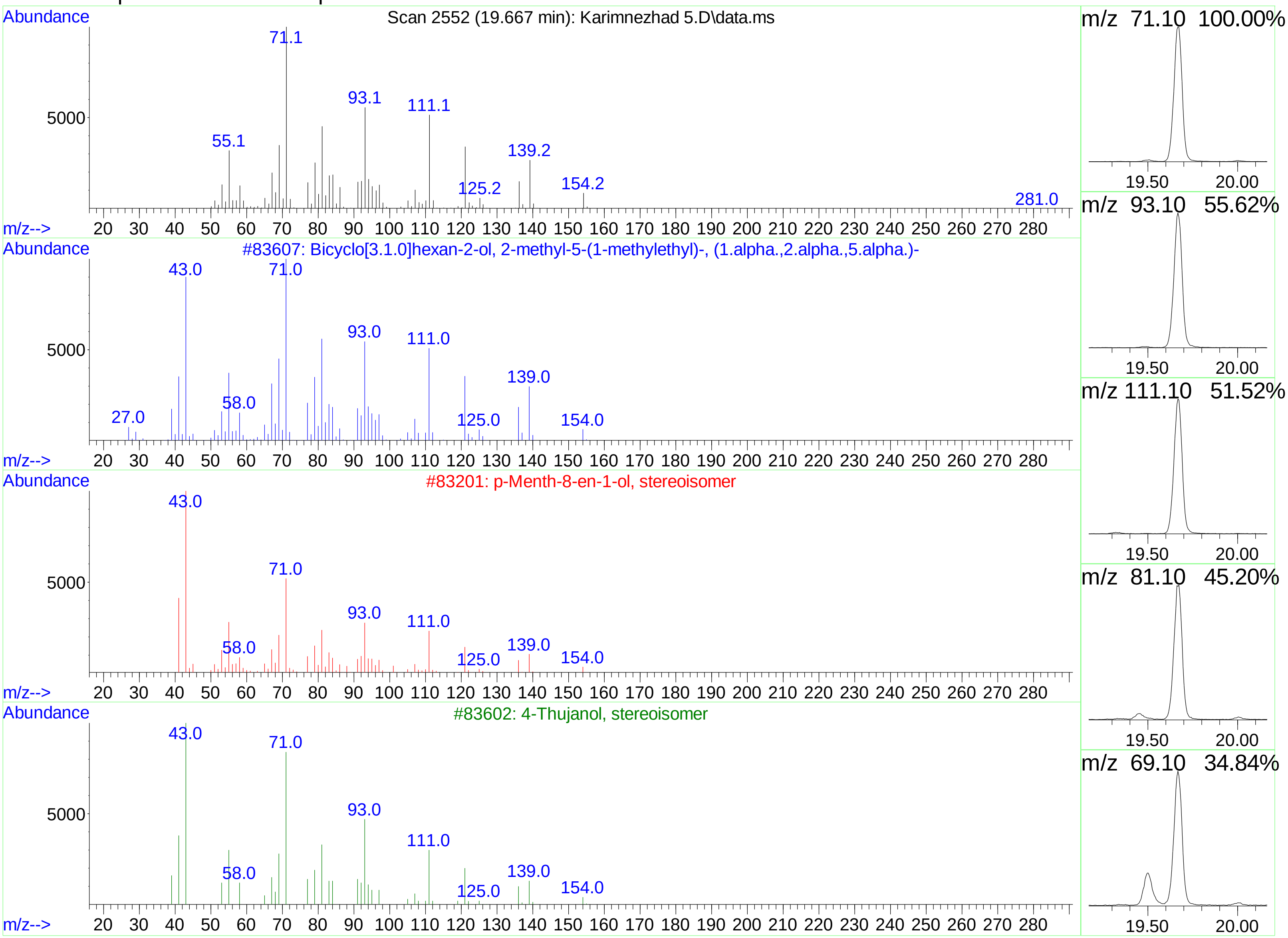


Data File: D:\msdchem\1\data\Karimnezhad 5.D
Sample : M15

Peak Number: 4 at 19.667 min Area: 67152146 Area % 0.33

| The 3 best hits from each library. | Ref\# | CAS\# | Qual |
|---|---|---|---|
| D:\Database\W10N14.L | | | |
| 1 Bicyclo[3.1.0]hexan-2-ol, 2-meth... | 83607 | 017699-16-0 | 98 |
| 2 p-Menth-8-en-1-ol, stereoisomer | 83201 | 007299-40-3 | 94 |
| 3 4-Thujanol, stereoisomer | 83602 | 017699-16-0 | 93 |

# Library Search Report - ChemStation Integrator

Unknown Spectrum based on Apex

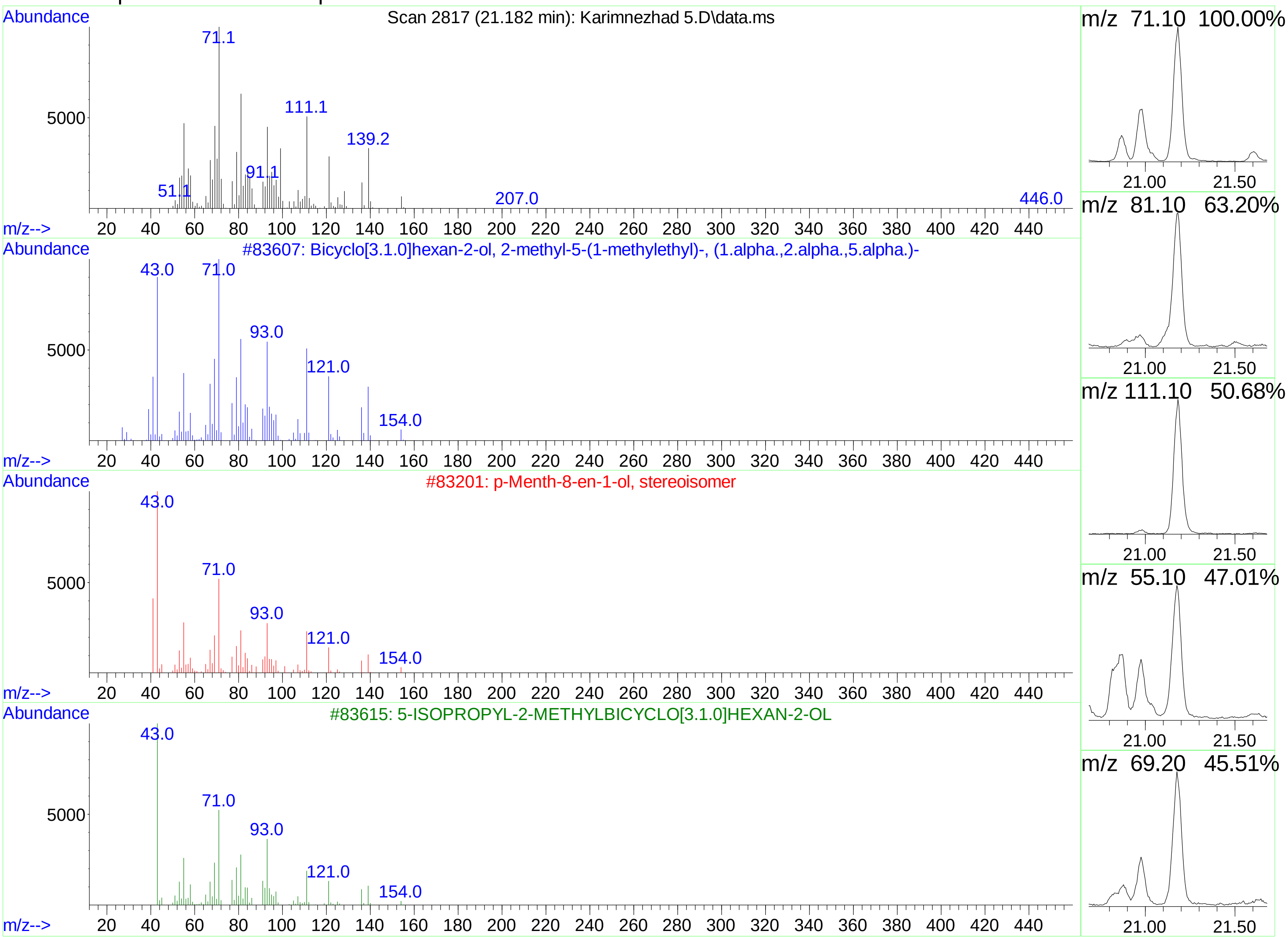


Data File: D:\msdchem\1\data\Karimnezhad 5.D
Sample : M15

Peak Number: 5 at 21.182 min Area: 39345498 Area % 0.19

| The 3 best hits from each library. | Ref# | CAS# | Qual |
|---|---|---|---|
| D:\Database\W10N14.L | | | |
| 1 Bicyclo[3.1.0]hexan-2-ol, 2-meth... | 83607 | 017699-16-0 | 98 |
| 2 p-Menth-8-en-1-ol, stereoisomer | 83201 | 007299-40-3 | 76 |
| 3 5-ISOPROPYL-2-METHYLBICYCLO[3.1.... | 83615 | 015826-82-1 | 55 |

Unknown Spectrum based on Apex

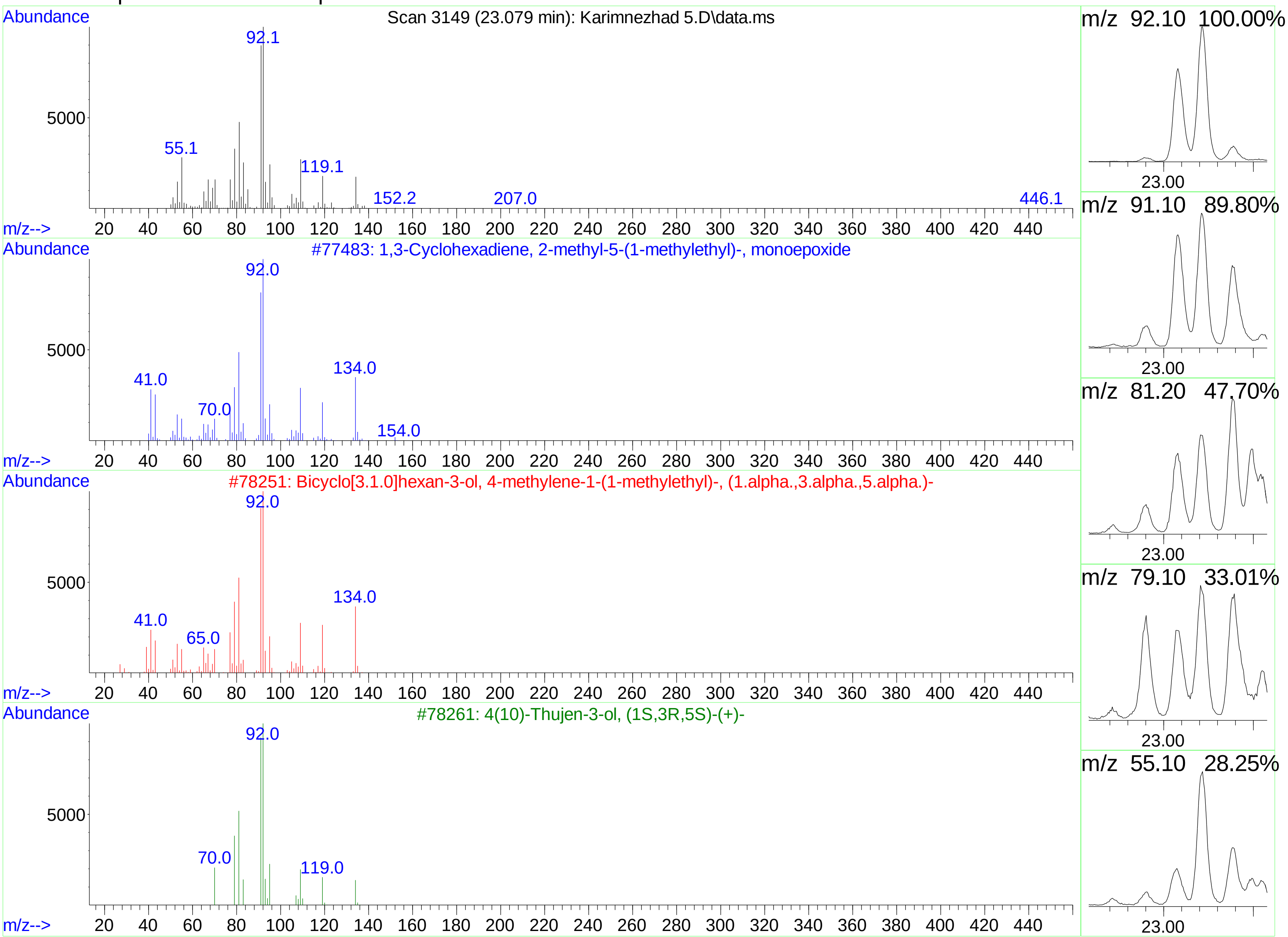


Data File: D:\msdchem\1\data\Karimnezhad 5.D
Sample : M15

Peak Number: 6 at 23.079 min Area: 23310449 Area % 0.12

The 3 best hits from each library. Ref\# CAS\# Qual

--------------------------------------------------------------------

D:\Database\W10N14.L

| | | | | |
|---|---|---|---|---|
| 1 | 1,3-Cyclohexadiene, 2-methyl-5-(... | 77483 | 072138-69-3 | 95 |
| 2 | Bicyclo[3.1.0]hexan-3-ol, 4-meth... | 78251 | 003310-02-9 | 74 |
| 3 | 4(10)-Thujen-3-ol, (1S,3R,5S)-(+)- | 78261 | 000471-16-9 | 72 |

Unknown Spectrum based on Apex

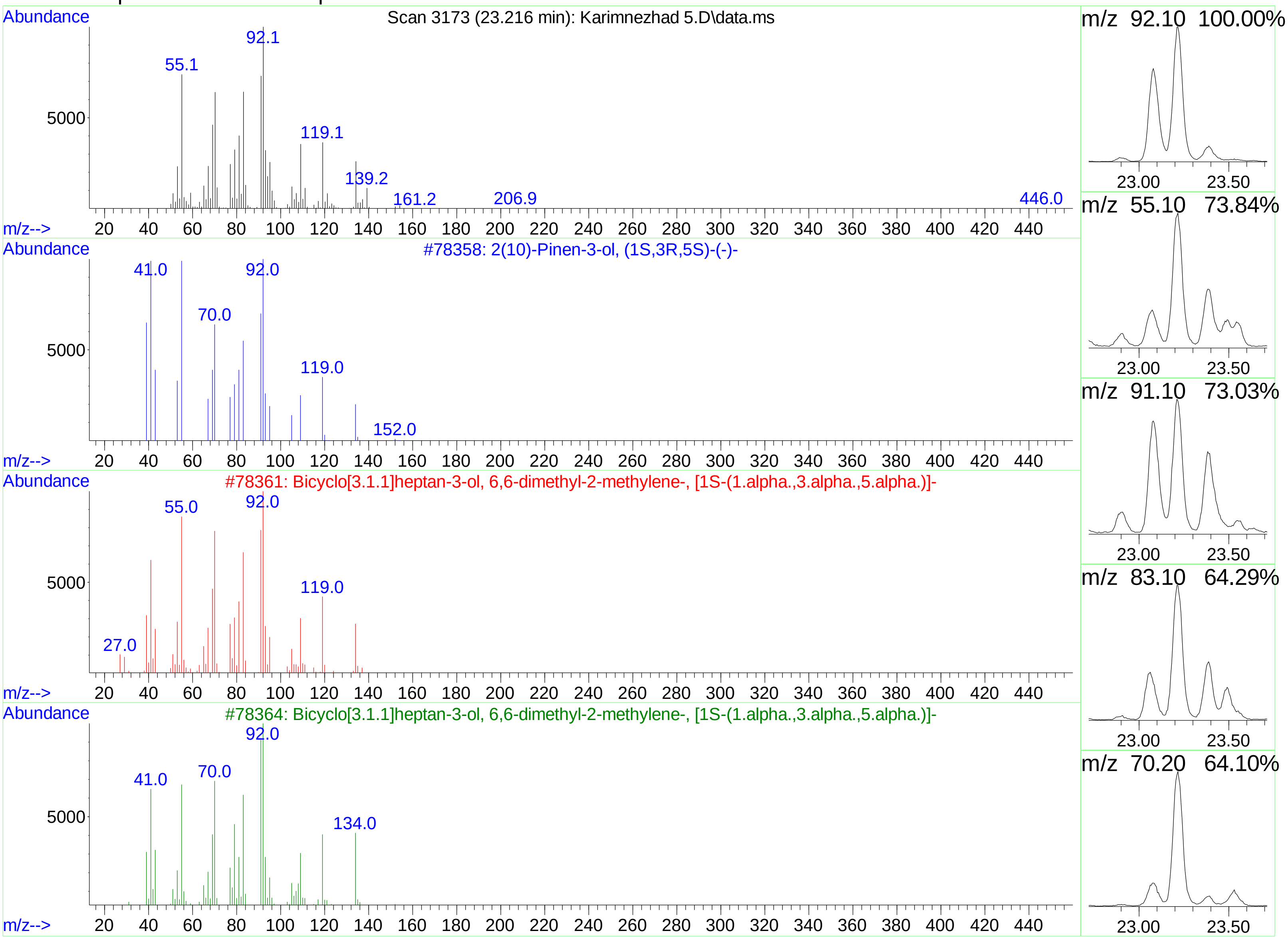


Data File: D:\msdchem\1\data\Karimnezhad 5.D
Sample : M15

Peak Number: 7 at 23.216 min Area: 46072470 Area % 0.23

The 3 best hits from each library. Ref\# CAS\# Qual

-------------------------------------------------------------------

D:\Database\W10N14.L

| | | Ref# | CAS# | Qual |
|---|---|---|---|---|
| 1 | 2(10)-Pinen-3-ol, (1S,3R,5S)-(-)- | 78358 | 000547-61-5 | 93 |
| 2 | Bicyclo[3.1.1]heptan-3-ol, 6,6-d... | 78361 | 000547-61-5 | 87 |
| 3 | Bicyclo[3.1.1]heptan-3-ol, 6,6-d... | 78364 | 000547-61-5 | 83 |

# Library Search Report - ChemStation Integrator

Unknown Spectrum based on Apex

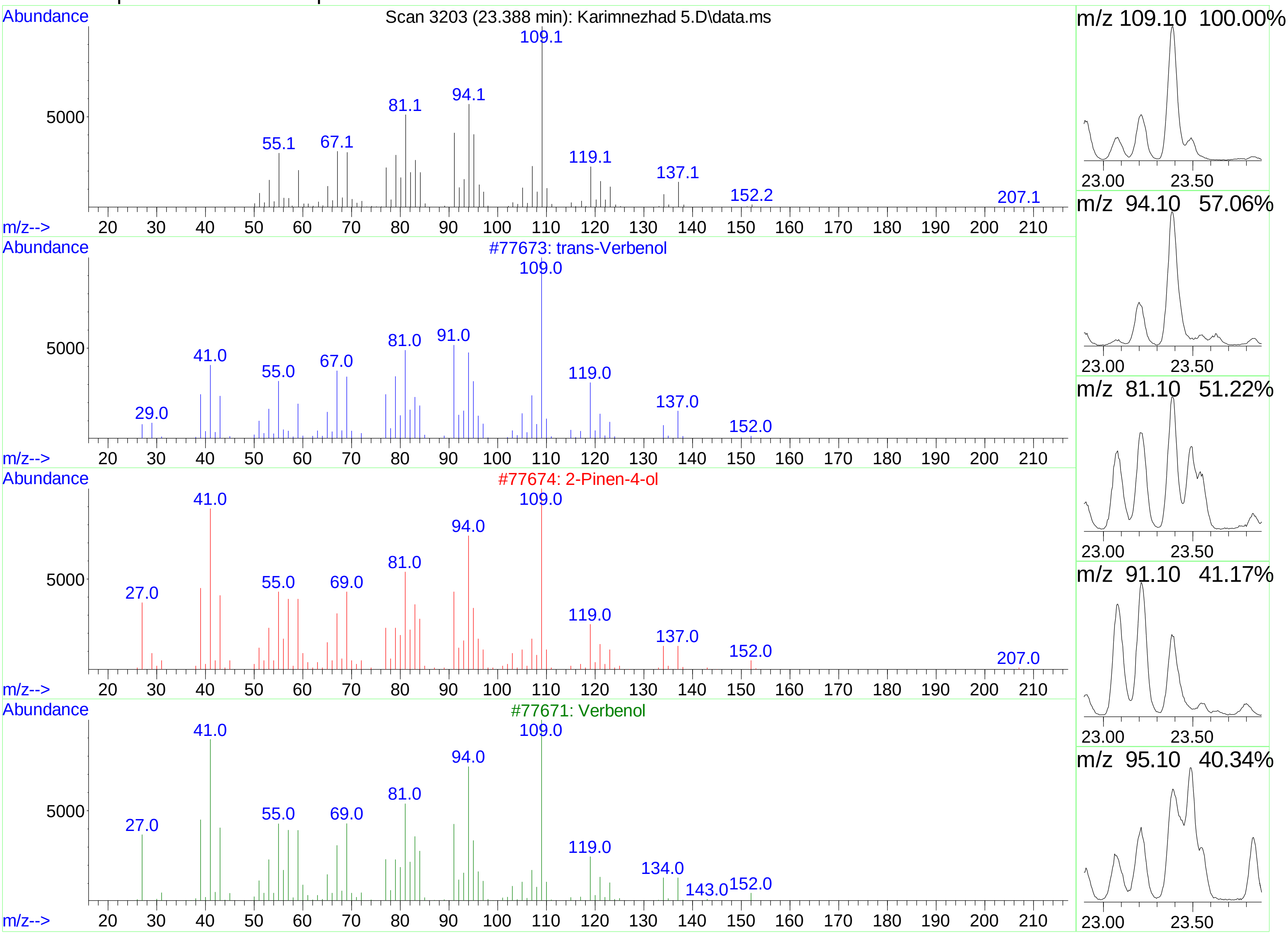


Data File: D:\msdchem\1\data\Karimnezhad 5.D
Sample : M15

Peak Number: 8 at 23.388 min Area: 43730735 Area % 0.22

| The 3 best hits from each library. | Ref# | CAS# | Qual |
|---|---|---|---|
| D:\Database\W10N14.L | | | |
| 1 trans-Verbenol | 77673 | 001820-09-3 | 96 |
| 2 2-Pinen-4-ol | 77674 | 000473-67-6 | 50 |
| 3 Verbenol | 77671 | 000473-67-6 | 50 |

# Library Search Report - ChemStation Integrator

Unknown Spectrum based on Apex

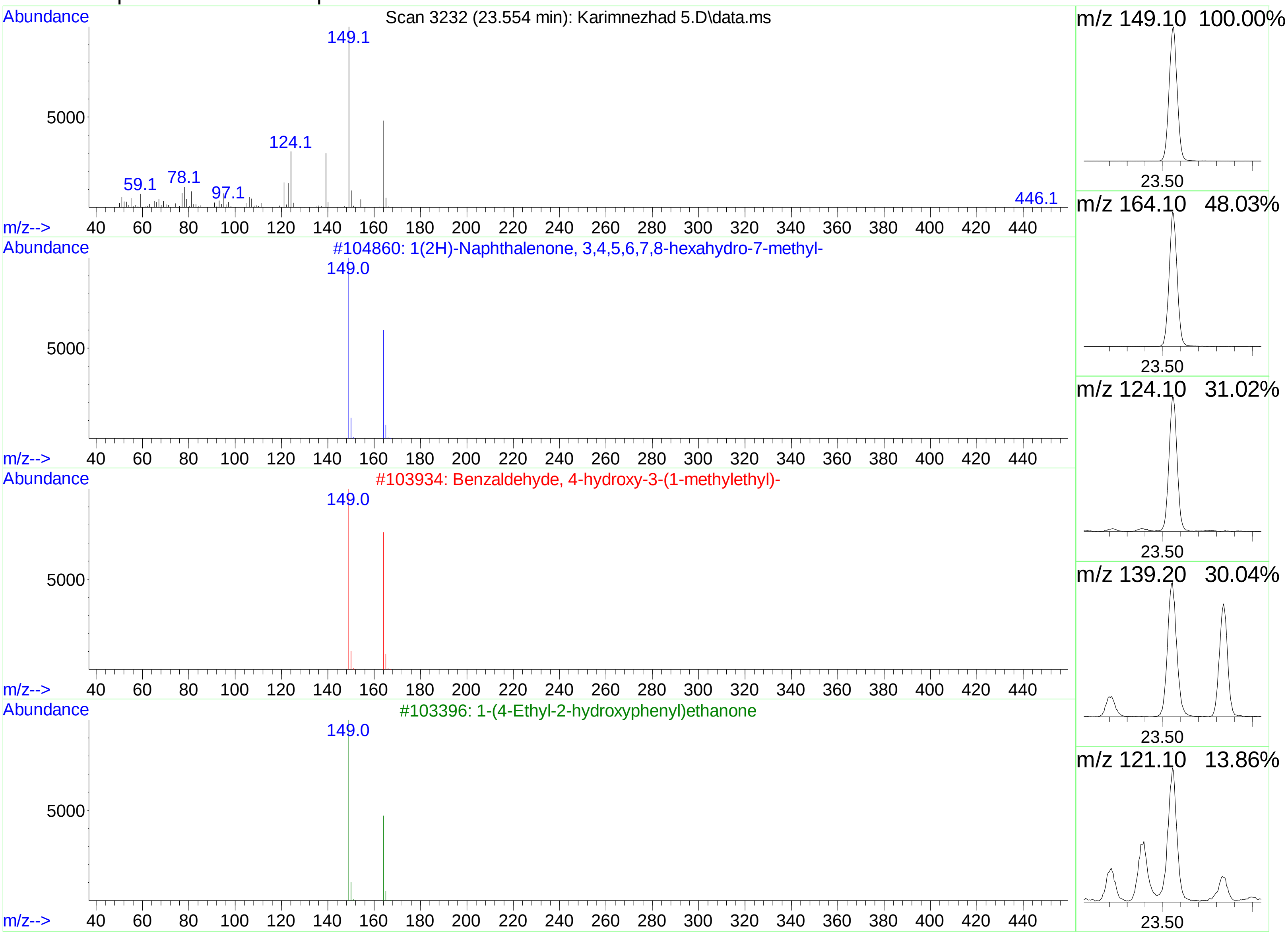


Data File: D:\msdchem\1\data\Karimnezhad 5.D
Sample : M15

Peak Number: 9 at 23.554 min Area: 54451820 Area % 0.27

| The 3 best hits from each library. | Ref\# | CAS\# | Qual |
|---|---|---|---|
| D:\Database\W10N14.L | | | |
| 1 1(2H)-Naphthalenone, 3,4,5,6,7,8... | 104860 | 059177-21-8 | 83 |
| 2 Benzaldehyde, 4-hydroxy-3-(1-met... | 103934 | 126274-94-0 | 83 |
| 3 1-(4-Ethyl-2-hydroxyphenyl)ethanone | 103396 | 2000103-39-6 | 83 |

Unknown Spectrum based on Apex

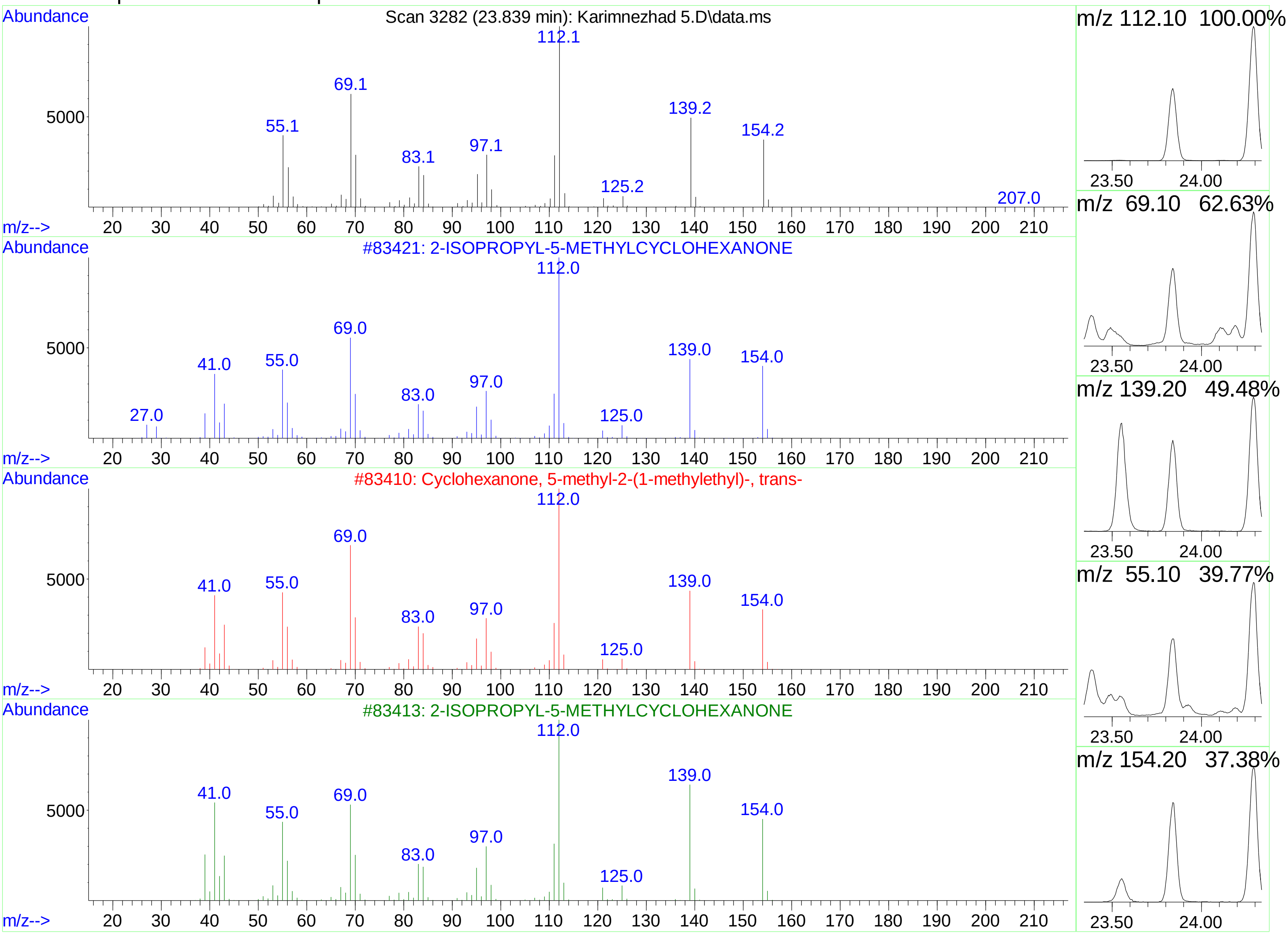


Data File: D:\msdchem\1\data\Karimnezhad 5.D
Sample : M15

Peak Number: 10 at 23.839 min Area: 37608464 Area % 0.19

| The 3 best hits from each library. | Ref\# | CAS\# | Qual |
|---|---|---|---|
| D:\Database\W10N14.L | | | |
| 1 2-ISOPROPYL-5-METHYLCYCLOHEXANONE | 83421 | 014073-97-3 | 98 |
| 2 Cyclohexanone, 5-methyl-2-(1-met... | 83410 | 000089-80-5 | 98 |
| 3 2-ISOPROPYL-5-METHYLCYCLOHEXANONE | 83413 | 000089-80-5 | 98 |

# Library Search Report - ChemStation Integrator

Unknown Spectrum based on Apex

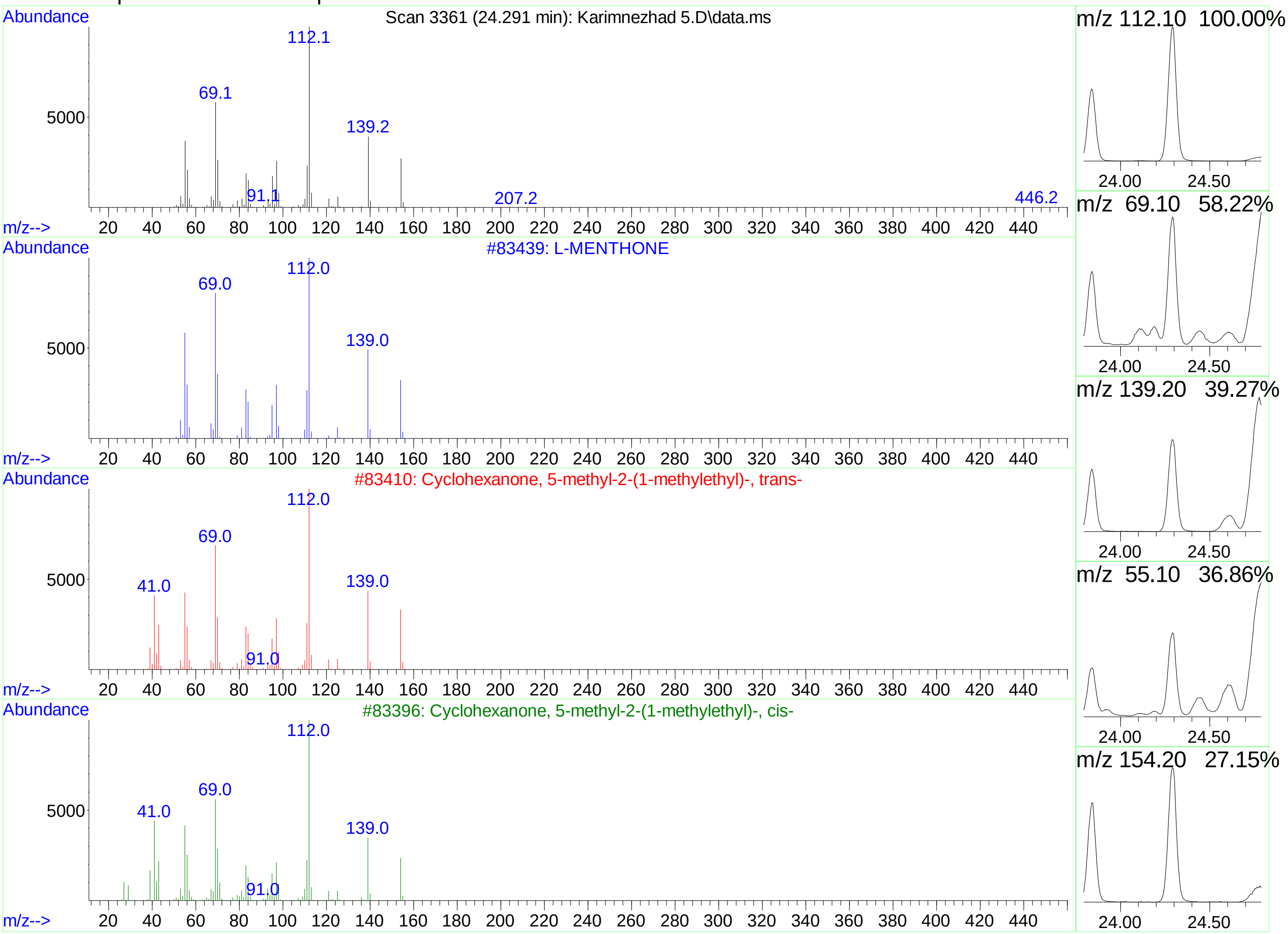


Data File: D:\msdchem\1\data\Karimnezhad 5.D
Sample : M15

Peak Number: 11 at 24.291 min Area: 81323438 Area % 0.40

| The 3 best hits from each library. | Ref\# | CAS\# | Qual |
|---|---|---|---|
| D:\Database\W10N14.L | | | |
| 1 L-MENTHONE | 83439 | 010458-14-7 | 98 |
| 2 Cyclohexanone, 5-methyl-2-(1-met... | 83410 | 000089-80-5 | 98 |
| 3 Cyclohexanone, 5-methyl-2-(1-met... | 83396 | 000491-07-6 | 98 |

# Library Search Report - ChemStation Integrator

Unknown Spectrum based on Apex

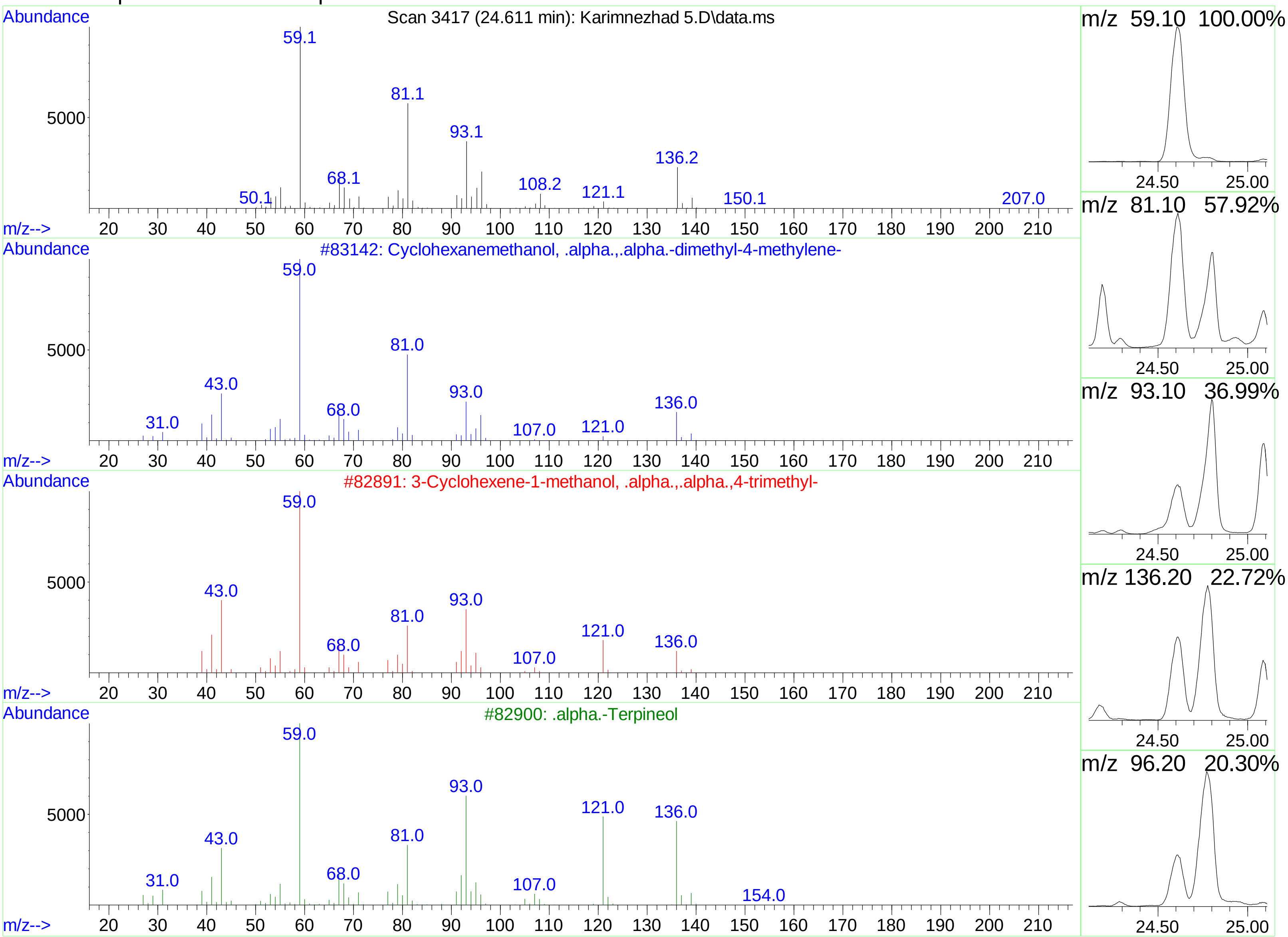


Data File: D:\msdchem\1\data\Karimnezhad 5.D
Sample : M15

Peak Number: 12 at 24.611 min Area: 84831781 Area % 0.42

| The 3 best hits from each library. | Ref# | CAS# | Qual |
|---|---|---|---|
| D:\Database\W10N14.L | | | |
| 1 Cyclohexanemethanol, .alpha.,.al... | 83142 | 007299-42-5 | 90 |
| 2 3-Cyclohexene-1-methanol, .alpha... | 82891 | 010482-56-1 | 53 |
| 3 .alpha.-Terpineol | 82900 | 000098-55-5 | 50 |

Unknown Spectrum based on Apex

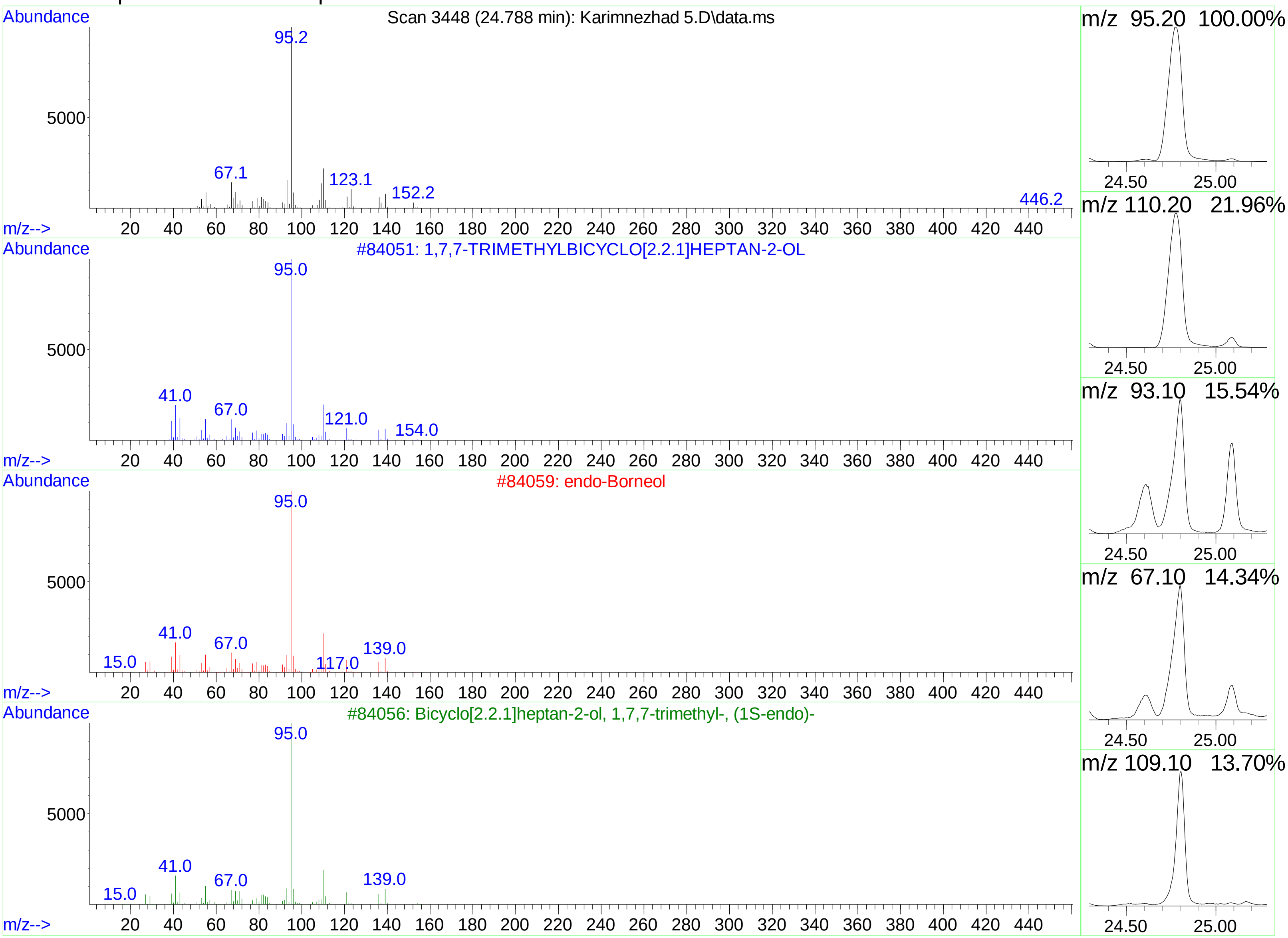


Data File: D:\msdchem\1\data\Karimnezhad 5.D
Sample : M15

Peak Number: 13 at 24.788 min Area: 361001153 Area % 1.79

| The 3 best hits from each library. | Ref# | CAS# | Qual |
|---|---|---|---|
| D:\Database\W10N14.L | | | |
| 1 1,7,7-TRIMETHYLBICYCLO[2.2.1]HEP... | 84051 | 000464-45-9 | 94 |
| 2 endo-Borneol | 84059 | 000507-70-0 | 94 |
| 3 Bicyclo[2.2.1]heptan-2-ol, 1,7,7... | 84056 | 000464-45-9 | 87 |

Unknown Spectrum based on Apex

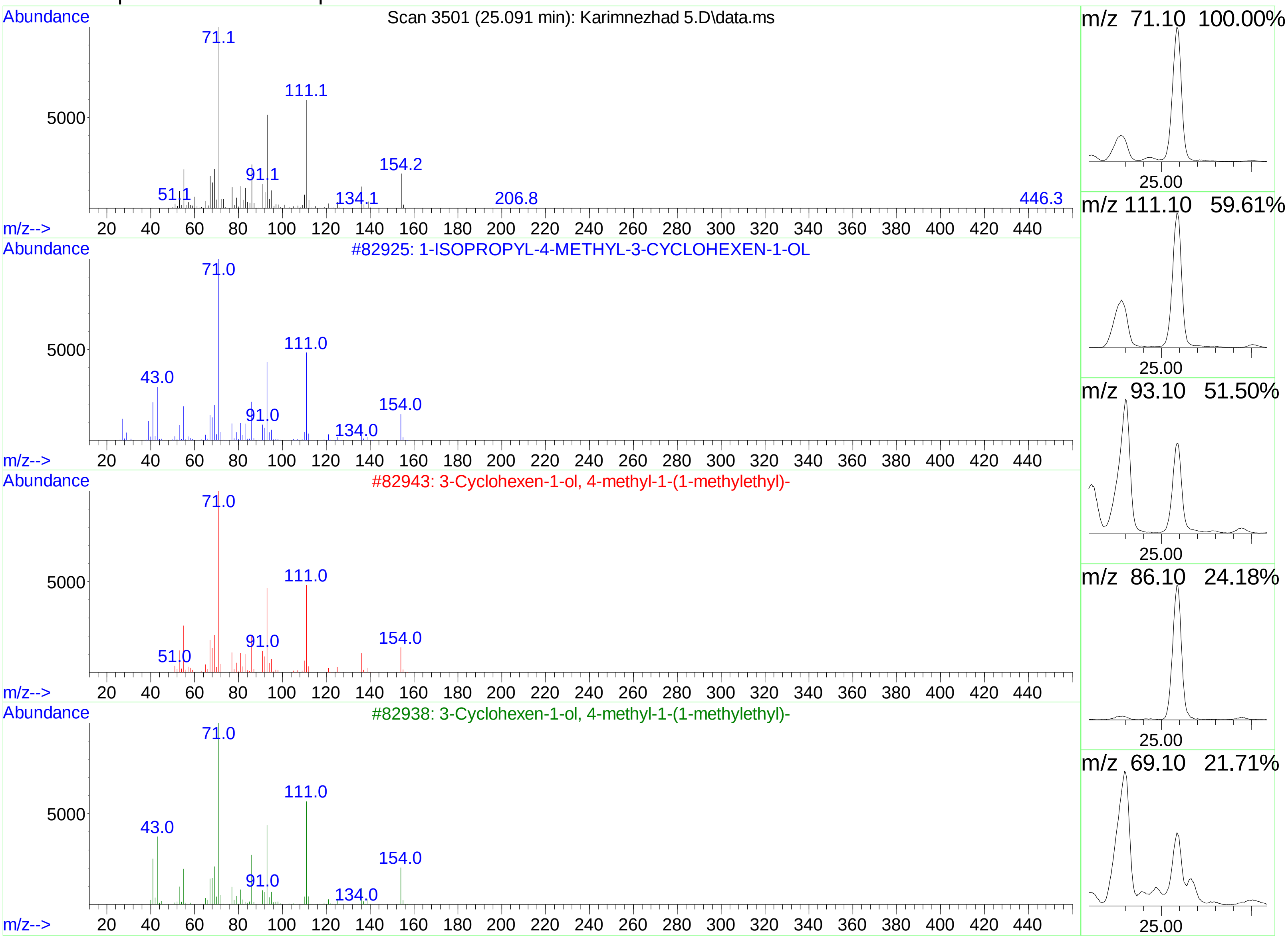


Data File: D:\msdchem\1\data\Karimnezhad 5.D
Sample : M15

Peak Number: 14 at 25.091 min Area: 120217567 Area % 0.59

| The 3 best hits from each library. | Ref\# | CAS\# | Qual |
|---|---|---|---|
| D:\Database\W10N14.L | | | |
| 1 1-ISOPROPYL-4-METHYL-3-CYCLOHEXE... | 82925 | 000562-74-3 | 98 |
| 2 3-Cyclohexen-1-ol, 4-methyl-1-(1... | 82943 | 000562-74-3 | 98 |
| 3 3-Cyclohexen-1-ol, 4-methyl-1-(1... | 82938 | 000562-74-3 | 97 |

# Library Search Report - ChemStation Integrator

Unknown Spectrum based on Apex

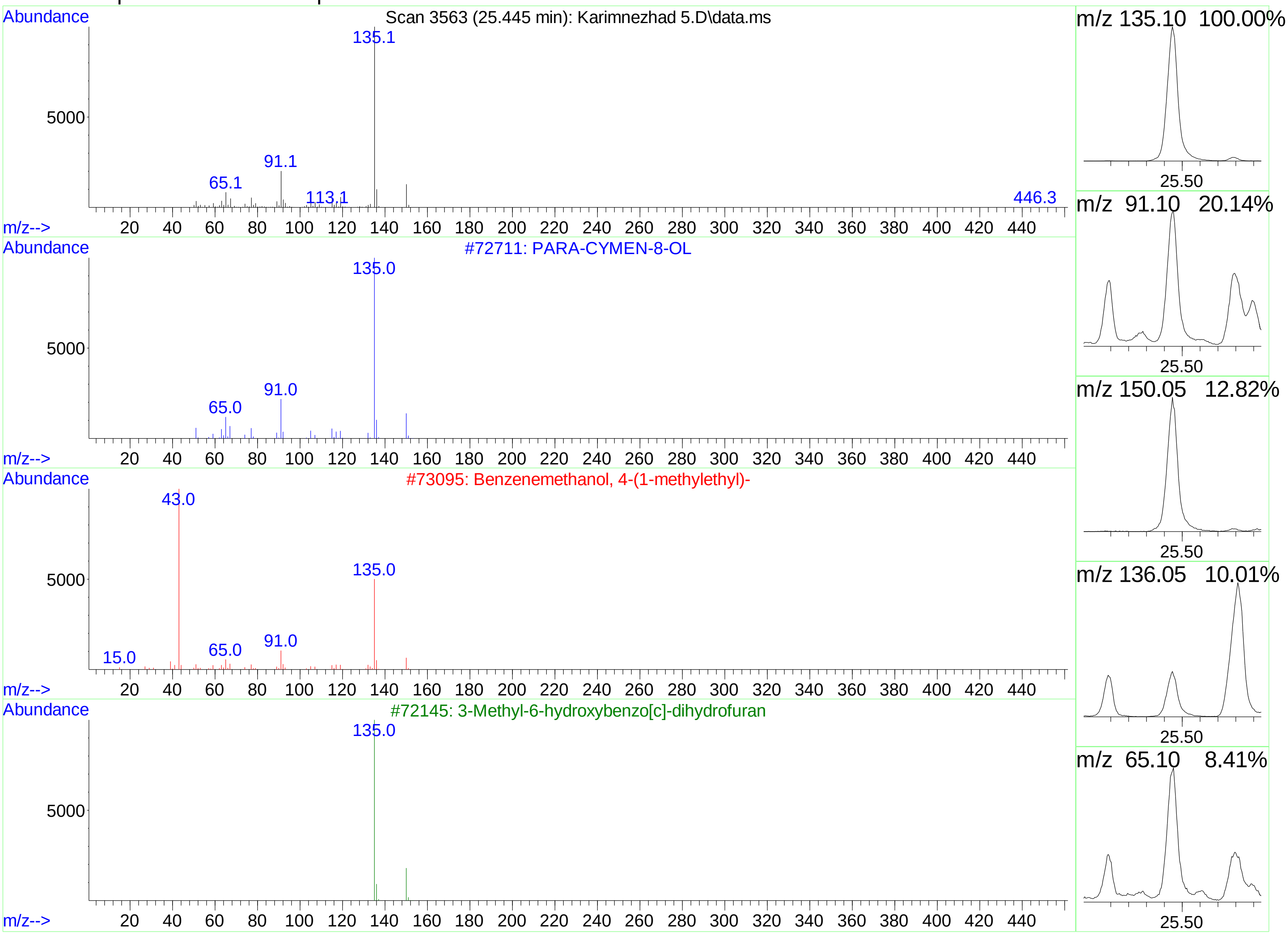


Data File: D:\msdchem\1\data\Karimnezhad 5.D
Sample : M15

Peak Number: 15 at 25.445 min Area: 75697668 Area % 0.37

| The 3 best hits from each library. | Ref# | CAS# | Qual |
|---|---|---|---|
| D:\Database\W10N14.L | | | |
| 1 PARA-CYMEN-8-OL | 72711 | 001197-01-9 | 93 |
| 2 Benzenemethanol, 4-(1-methylethyl)- | 73095 | 000536-60-7 | 86 |
| 3 3-Methyl-6-hydroxybenzo[c]-dihyd... | 72145 | 2000072-14-5 | 83 |

Unknown Spectrum based on Apex

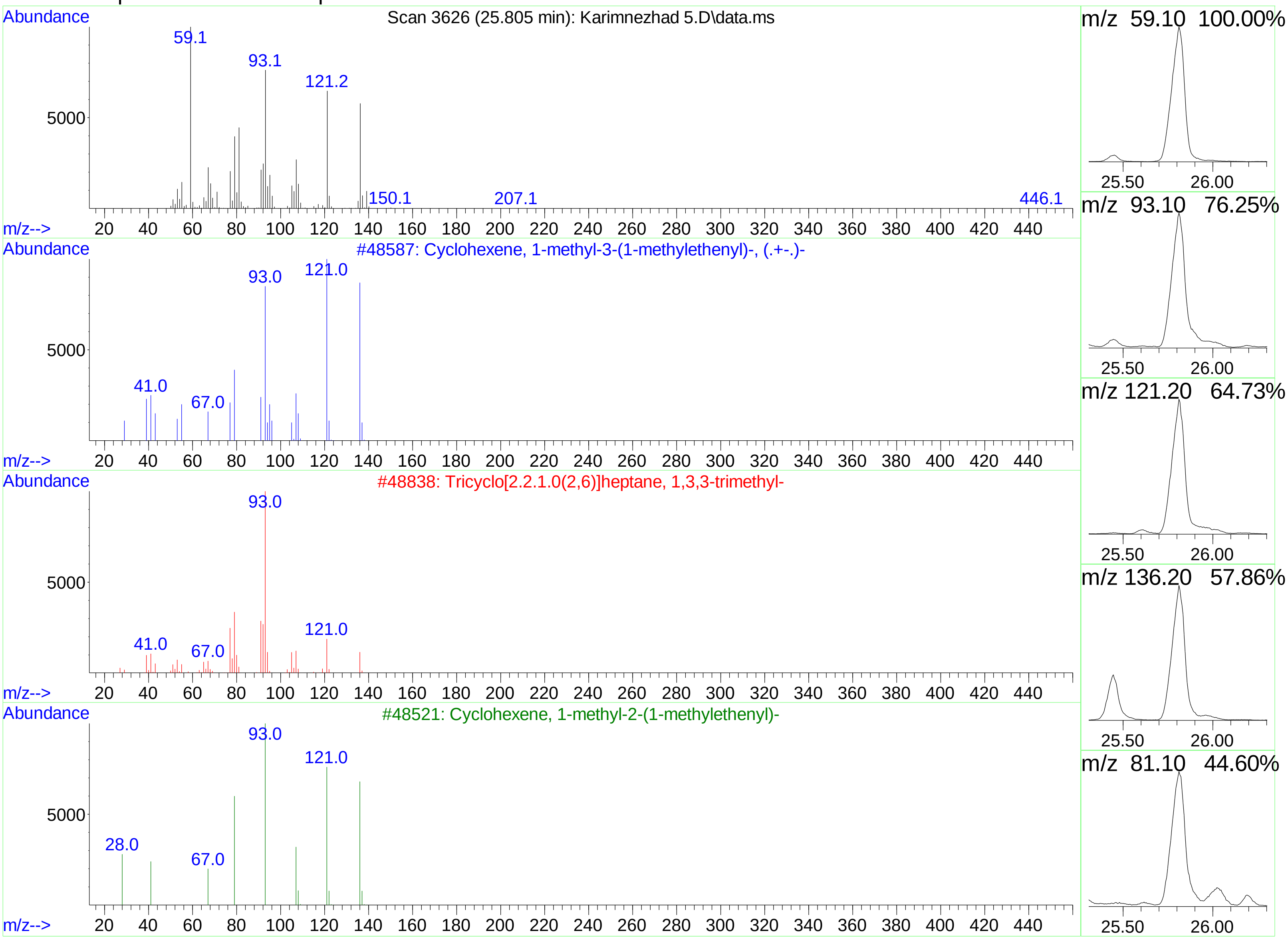


Data File: D:\msdchem\1\data\Karimnezhad 5.D
Sample : M15

Peak Number: 16 at 25.805 min Area: 134466527 Area % 0.67

| The 3 best hits from each library. | Ref# | CAS# | Qual |
|---|---|---|---|
| D:\Database\W10N14.L | | | |
| 1 Cyclohexene, 1-methyl-3-(1-methy... | 48587 | 000499-03-6 | 89 |
| 2 Tricyclo[2.2.1.0(2,6)]heptane, 1... | 48838 | 000488-97-1 | 55 |
| 3 Cyclohexene, 1-methyl-2-(1-methy... | 48521 | 076480-15-4 | 53 |

Unknown Spectrum based on Apex

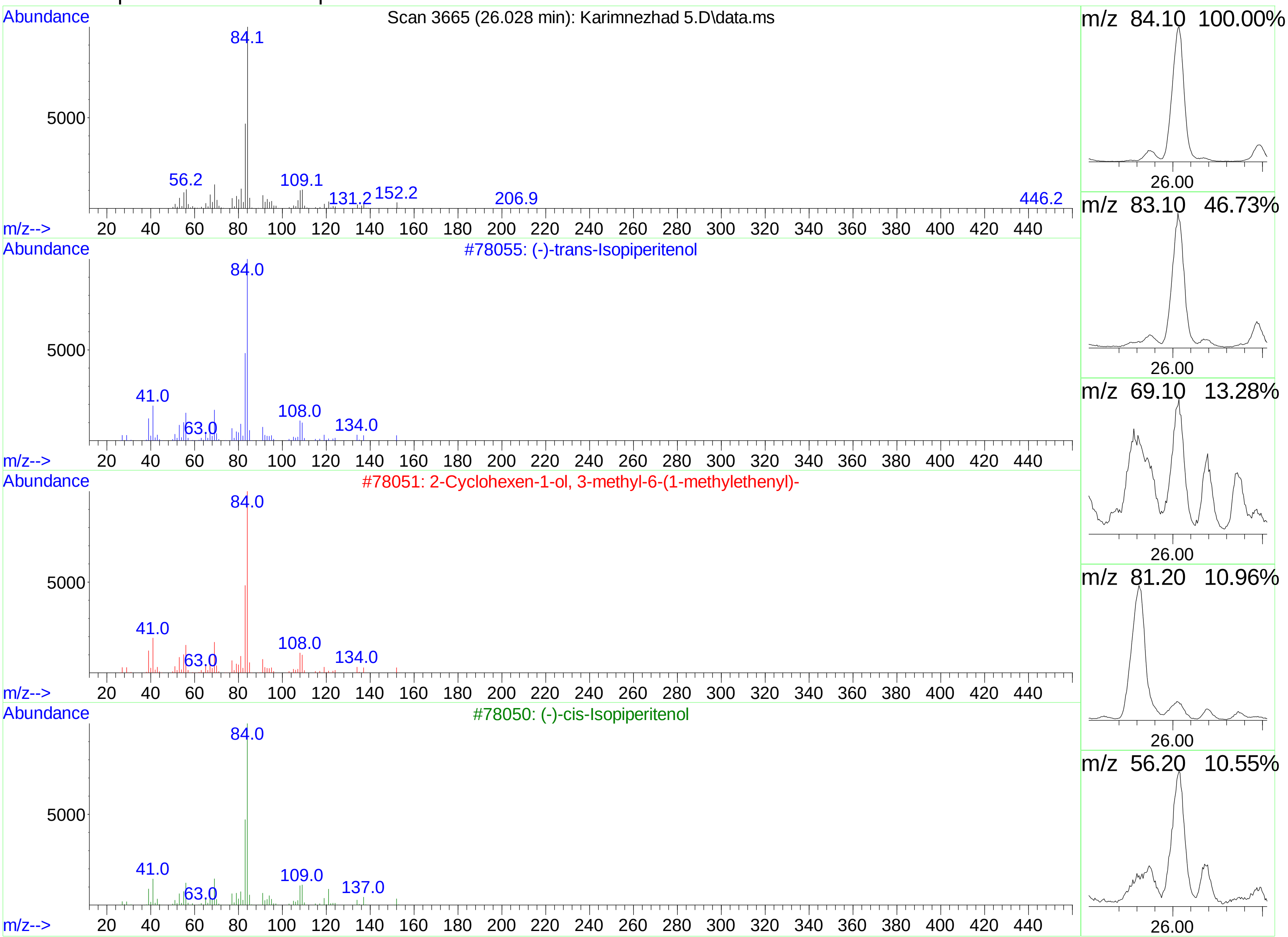


Data File: D:\msdchem\1\data\Karimnezhad 5.D
Sample : M15

Peak Number: 17 at 26.028 min Area: 29377358 Area % 0.15

| The 3 best hits from each library. | Ref# | CAS# | Qual |
|---|---|---|---|
| D:\Database\W10N14.L | | | |
| 1 (-)-trans-Isopiperitenol | 78055 | 074410-00-7 | 95 |
| 2 2-Cyclohexen-1-ol, 3-methyl-6-(1... | 78051 | 000491-05-4 | 95 |
| 3 (-)-cis-Isopiperitenol | 78050 | 096555-02-1 | 83 |

Unknown Spectrum based on Apex

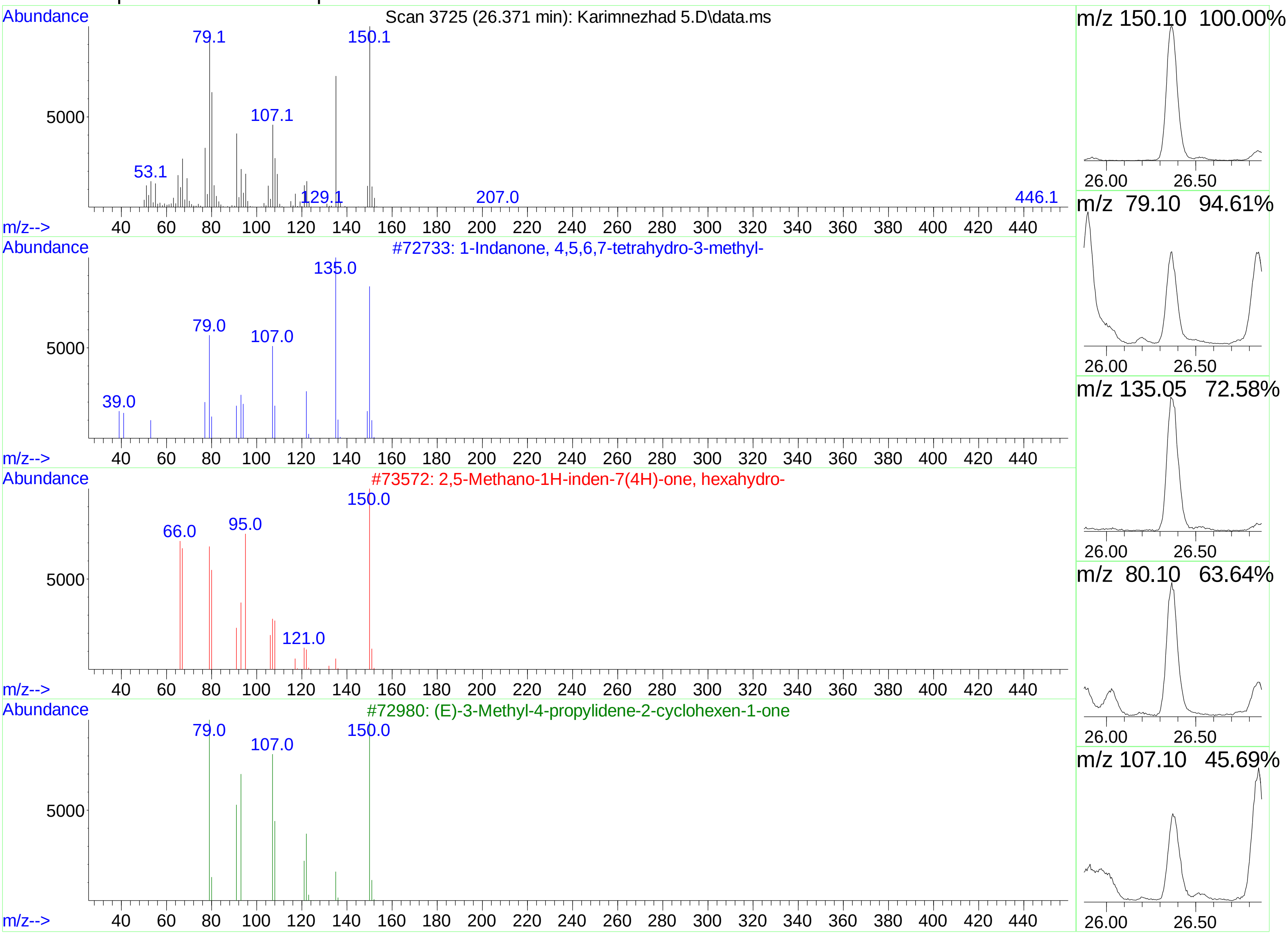


Data File: D:\msdchem\1\data\Karimnezhad 5.D
Sample : M15

Peak Number: 18 at 26.371 min Area: 25376188 Area % 0.13

| The 3 best hits from each library. | Ref# | CAS# | Qual |
|---|---|---|---|
| D:\Database\W10N14.L | | | |
| 1 1-Indanone, 4,5,6,7-tetrahydro-3... | 72733 | 018631-68-0 | 83 |
| 2 2,5-Methano-1H-inden-7(4H)-one, ... | 73572 | 027567-85-7 | 72 |
| 3 (E)-3-Methyl-4-propylidene-2-cyc... | 72980 | 2000072-98-0 | 68 |

Unknown Spectrum based on Apex

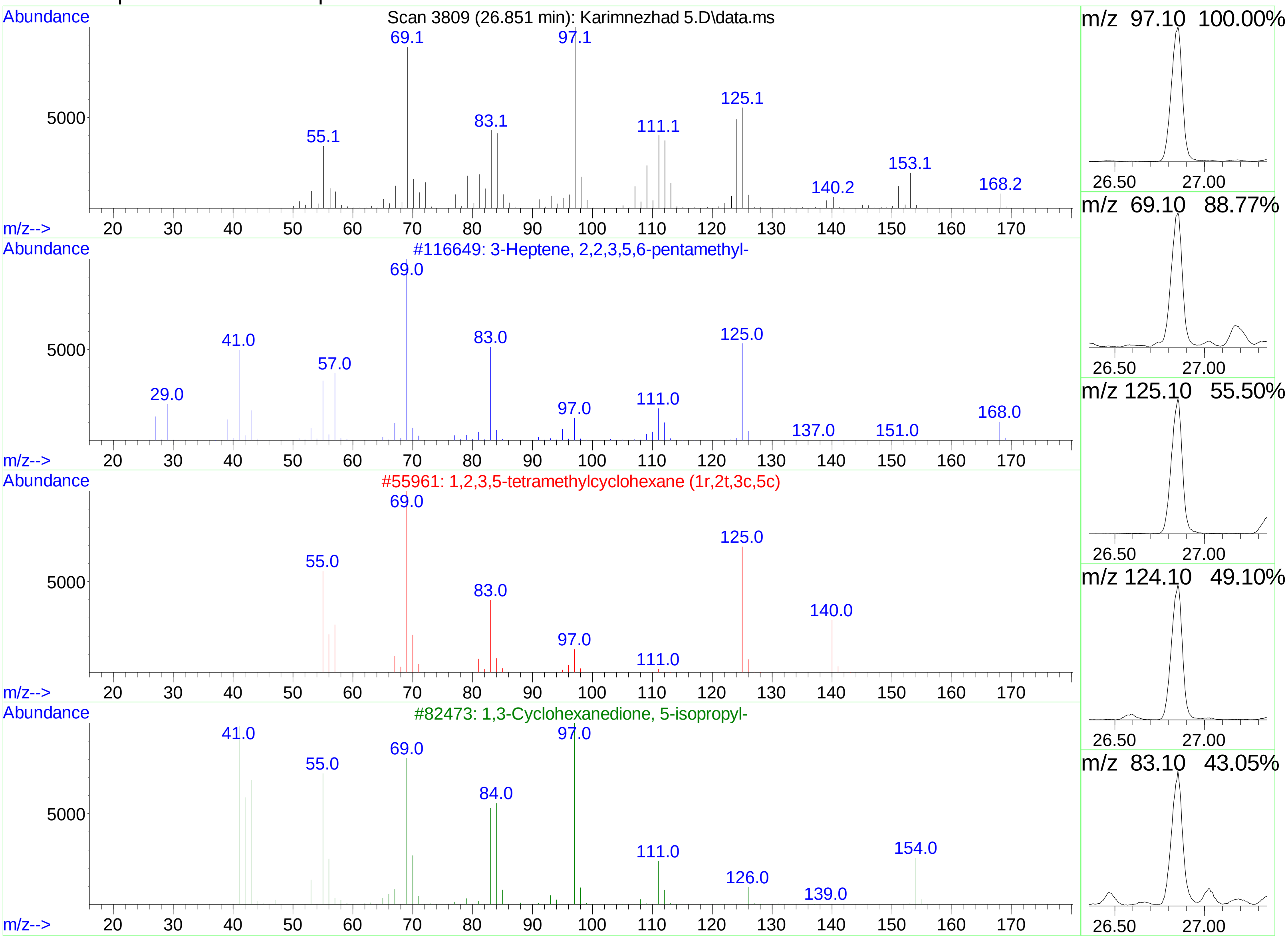


Data File: D:\msdchem\1\data\Karimnezhad 5.D
Sample : M15

Peak Number: 19 at 26.851 min Area: 159978381 Area % 0.79

| The 3 best hits from each library. | Ref\# | CAS\# | Qual |
|---|---|---|---|
| D:\Database\W10N14.L | | | |
| 1 3-Heptene, 2,2,3,5,6-pentamethyl- | 116649 | 116164-06-8 | 41 |
| 2 1,2,3,5-tetramethylcyclohexane (... | 55961 | 019899-28-6 | 38 |
| 3 1,3-Cyclohexanedione, 5-isopropyl- | 82473 | 018456-87-6 | 38 |

Unknown Spectrum based on Apex

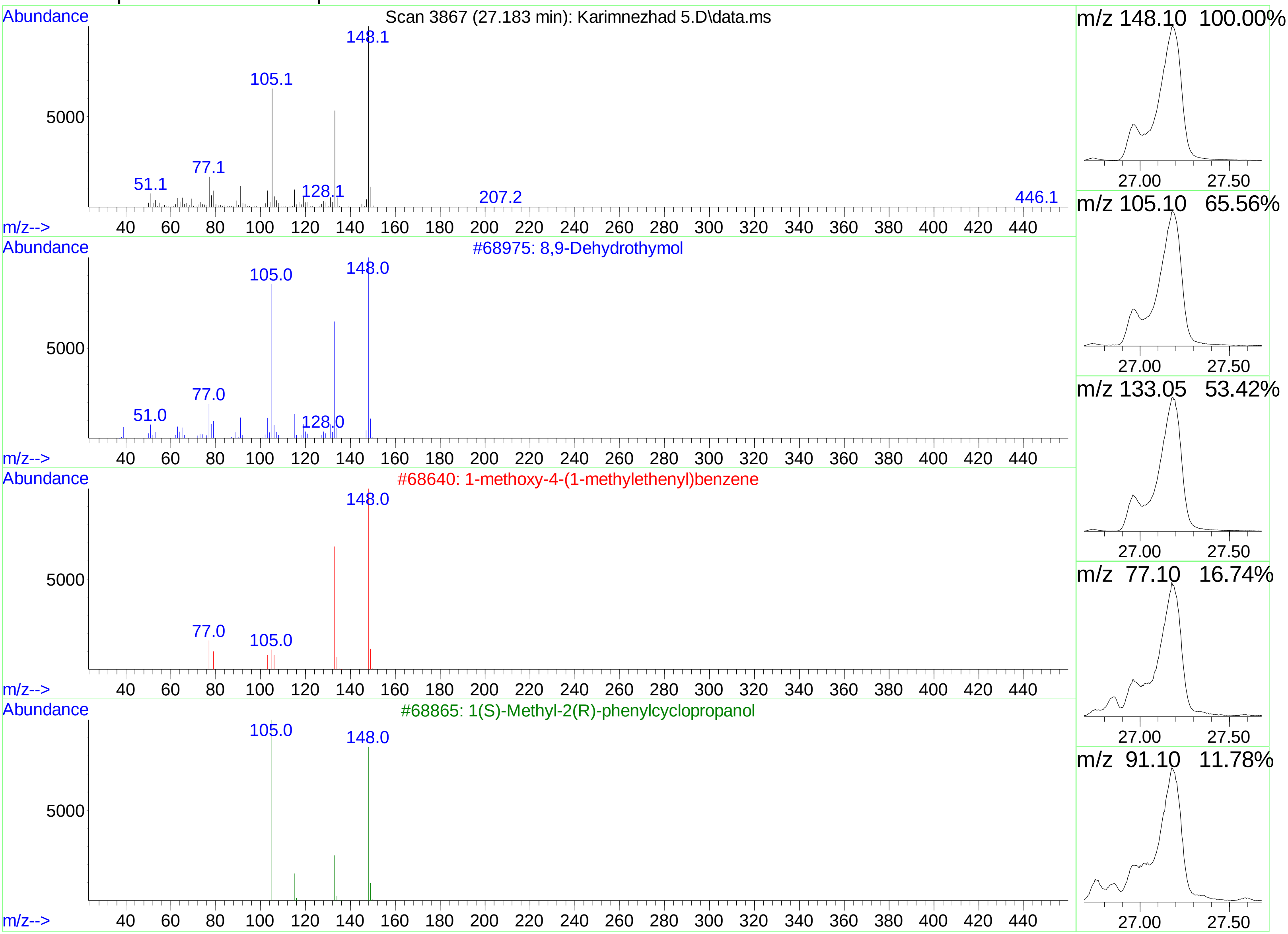


Data File: D:\msdchem\1\data\Karimnezhad 5.D
Sample : M15

Peak Number: 20 at 27.183 min Area: 486973833 Area % 2.41

| The 3 best hits from each library. | Ref# | CAS# | Qual |
|---|---|---|---|
| D:\Database\W10N14.L | | | |
| 1 8,9-Dehydrothymol | 68975 | 018612-99-2 | 95 |
| 2 1-methoxy-4-(1-methylethenyl)ben... | 68640 | 2000068-64-0 | 81 |
| 3 1(S)-Methyl-2(R)-phenylcycloprop... | 68865 | 2000068-86-5 | 72 |

Unknown Spectrum based on Apex

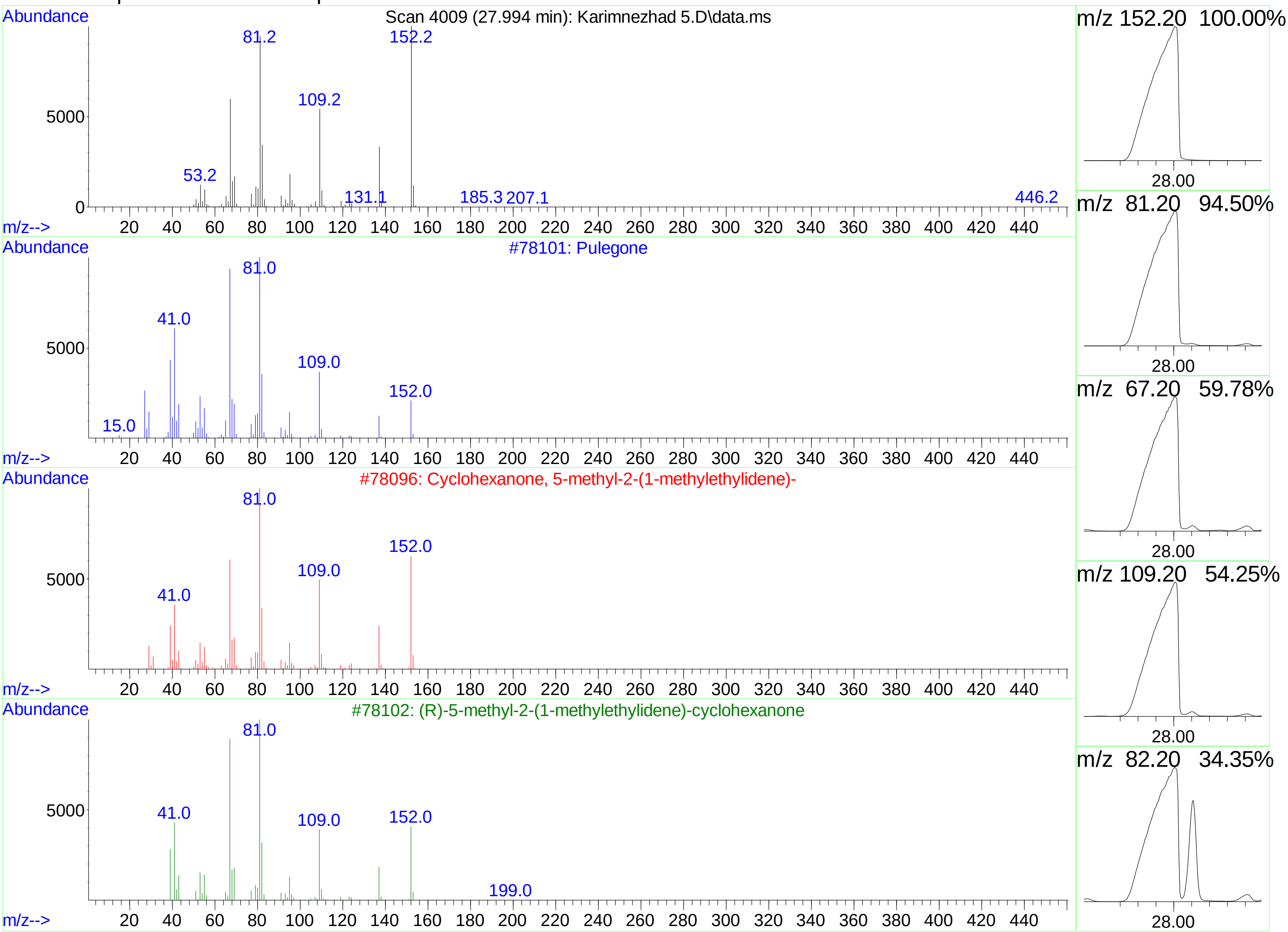


Data File: D:\msdchem\1\data\Karimnezhad 5.D
Sample : M15

Peak Number: 21 at 27.994 min Area: 2185040922 Area % 10.81

| The 3 best hits from each library. | Ref\# | CAS\# | Qual |
|---|---|---|---|
| D:\Database\W10N14.L | | | |
| 1 Pulegone | 78101 | 000089-82-7 | 97 |
| 2 Cyclohexanone, 5-methyl-2-(1-met... | 78096 | 015932-80-6 | 96 |
| 3 (R)-5-methyl-2-(1-methylethylide... | 78102 | 000089-82-7 | 96 |

Unknown Spectrum based on Apex

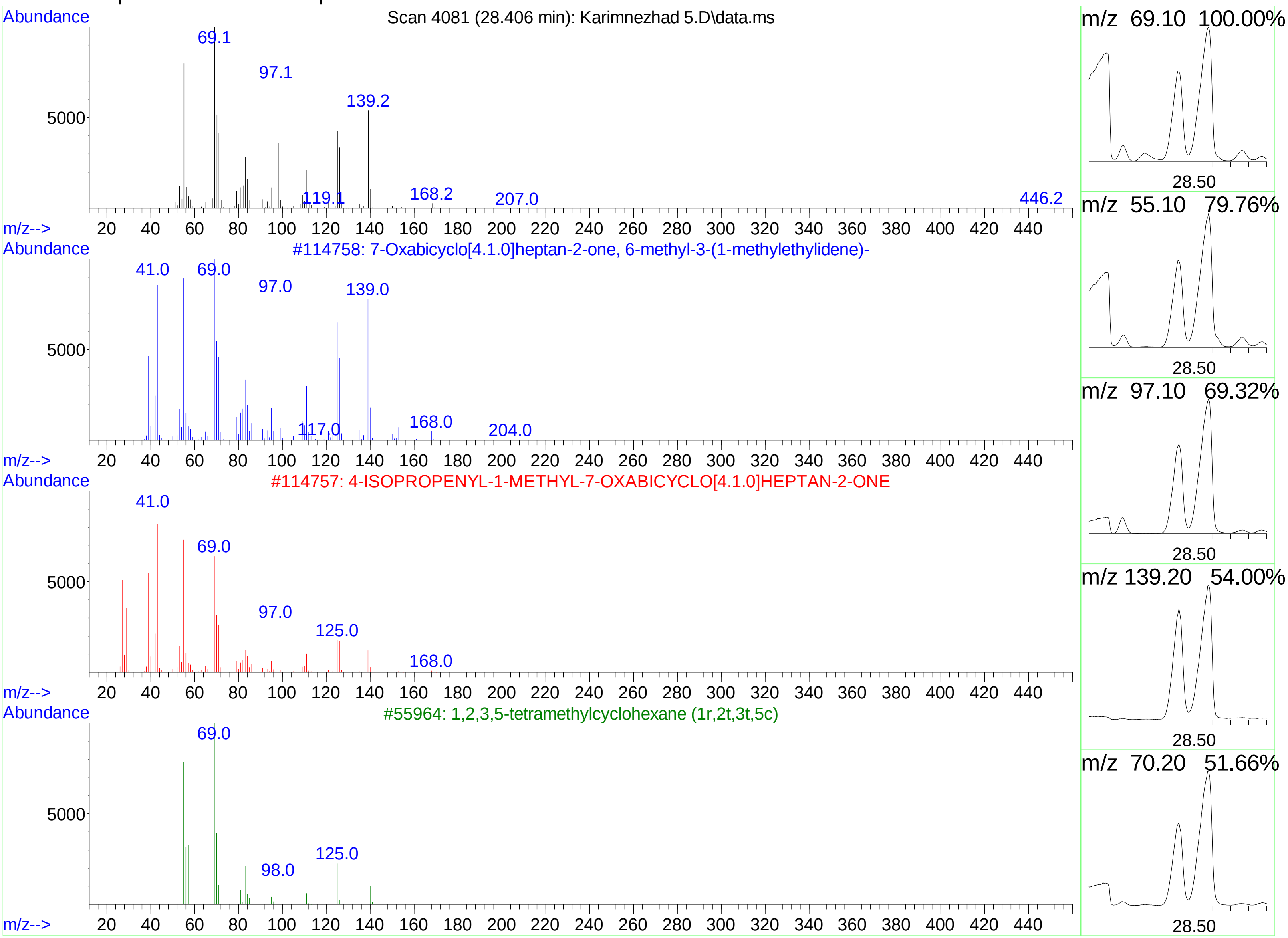


Data File: D:\msdchem\1\data\Karimnezhad 5.D
Sample : M15

Peak Number: 22 at 28.406 min Area: 154516641 Area % 0.76

The 3 best hits from each library. Ref\# CAS\# Qual

-------------------------------------------------------------------

D:\Database\W10N14.L

| | | Ref\# | CAS\# | Qual |
|---|---|---|---|---|
| 1 | 7-Oxabicyclo[4.1.0]heptan-2-one,... | 114758 | 035178-55-3 | 97 |
| 2 | 4-ISOPROPENYL-1-METHYL-7-OXABICY... | 114757 | 035178-55-3 | 49 |
| 3 | 1,2,3,5-tetramethylcyclohexane (... | 55964 | 019899-29-7 | 43 |

Unknown Spectrum based on Apex

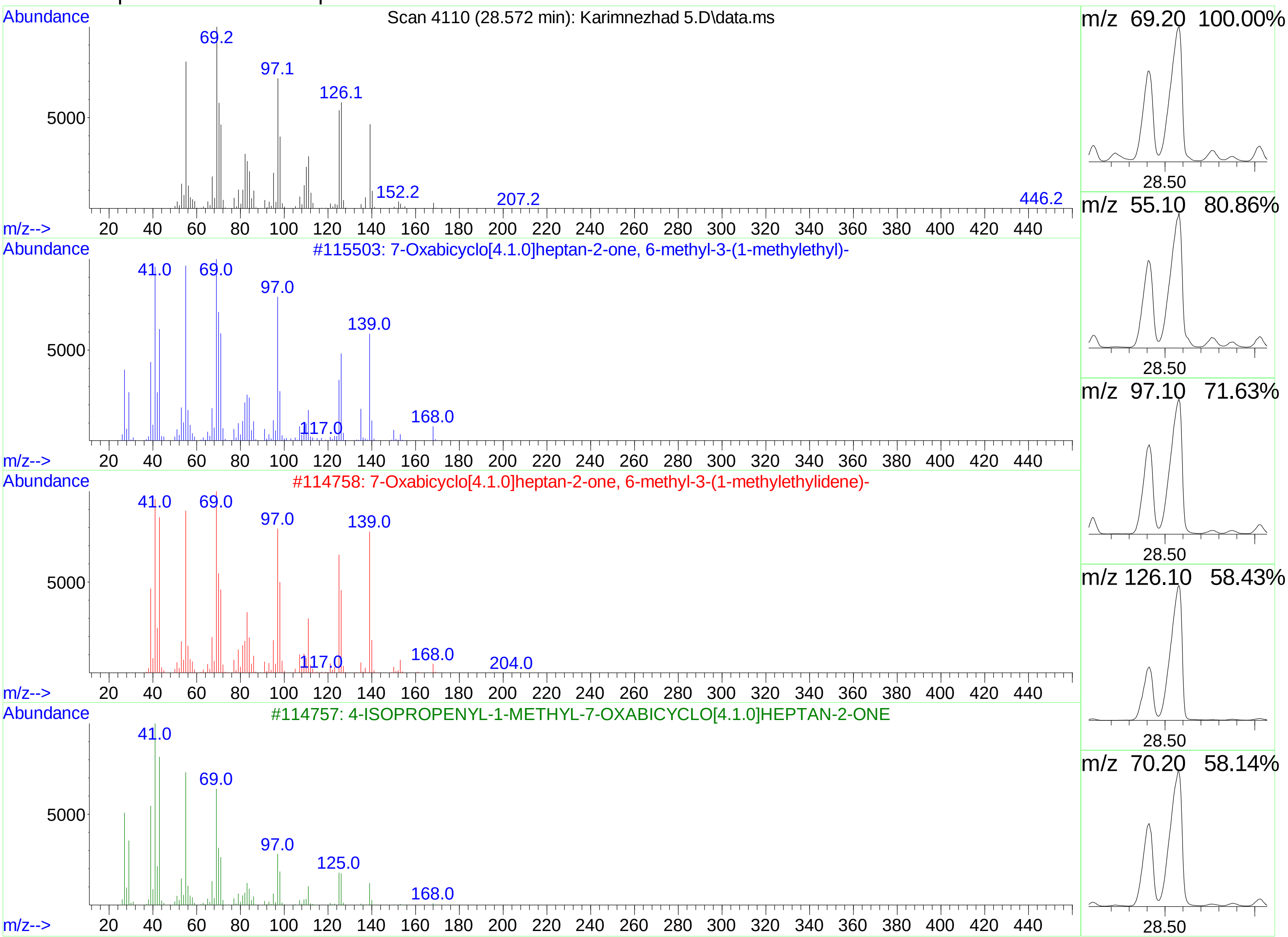


Data File: D:\msdchem\1\data\Karimnezhad 5.D
Sample : M15

Peak Number: 23 at 28.572 min Area: 345935749 Area % 1.71

The 3 best hits from each library. Ref\# CAS\# Qual

---------------------------------------------------------------------

D:\Database\W10N14.L

| | | | | |
|---|---|---|---|---|
| 1 | 7-Oxabicyclo[4.1.0]heptan-2-one,... | 115503 | 005286-38-4 | 76 |
| 2 | 7-Oxabicyclo[4.1.0]heptan-2-one,... | 114758 | 035178-55-3 | 76 |
| 3 | 4-ISOPROPENYL-1-METHYL-7-OXABICY... | 114757 | 035178-55-3 | 60 |

Unknown Spectrum based on Apex

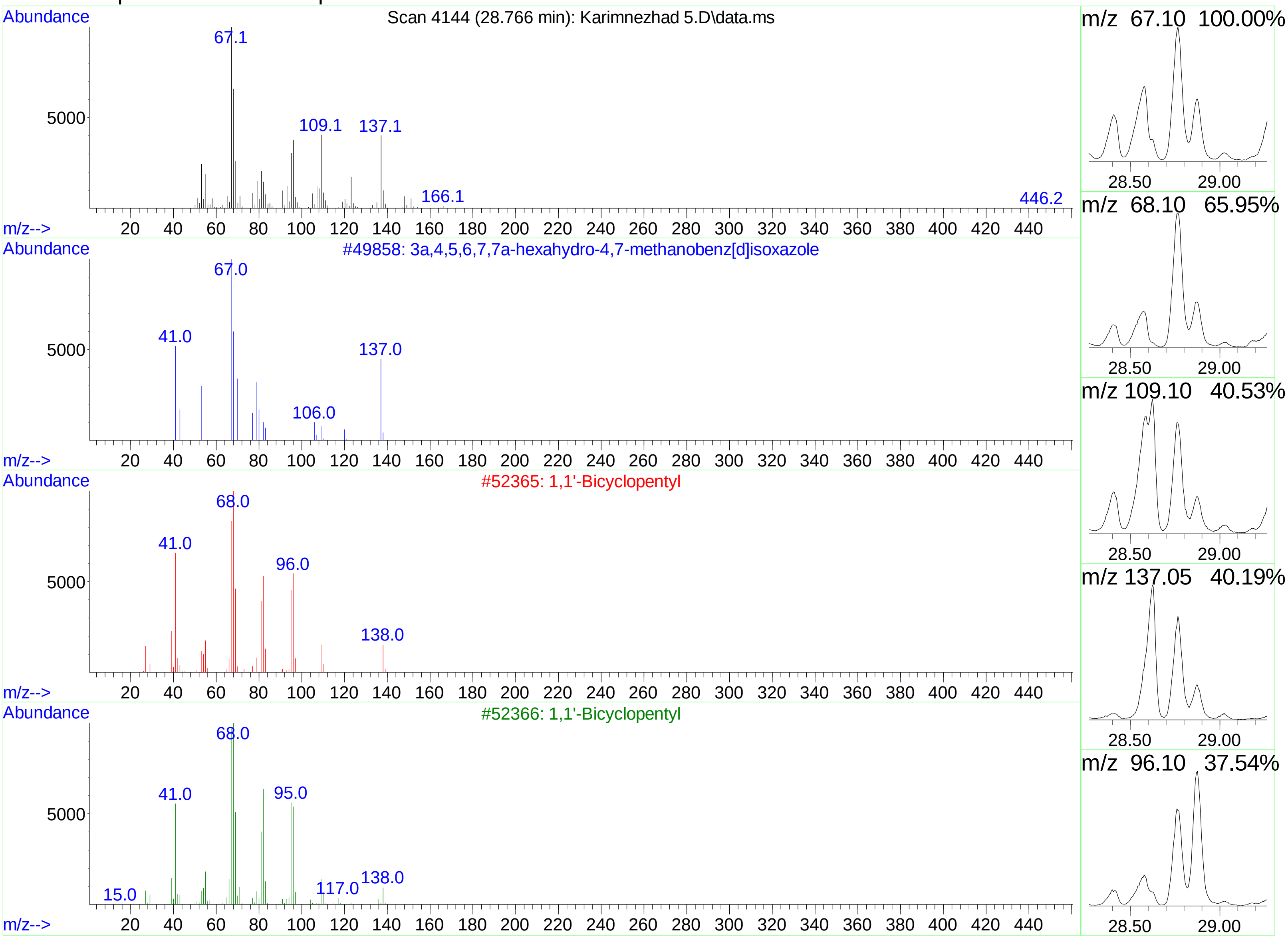


Data File: D:\msdchem\1\data\Karimnezhad 5.D
Sample : M15

Peak Number: 24 at 28.766 min Area: 57386792 Area % 0.28

| The 3 best hits from each library. | Ref\# | CAS\# | Qual |
|---|---|---|---|
| D:\Database\W10N14.L | | | |
| 1 3a,4,5,6,7,7a-hexahydro-4,7-meth... | 49858 | 015166-80-0 | 49 |
| 2 1,1'-Bicyclopentyl | 52365 | 001636-39-1 | 41 |
| 3 1,1'-Bicyclopentyl | 52366 | 001636-39-1 | 38 |

Unknown Spectrum based on Apex

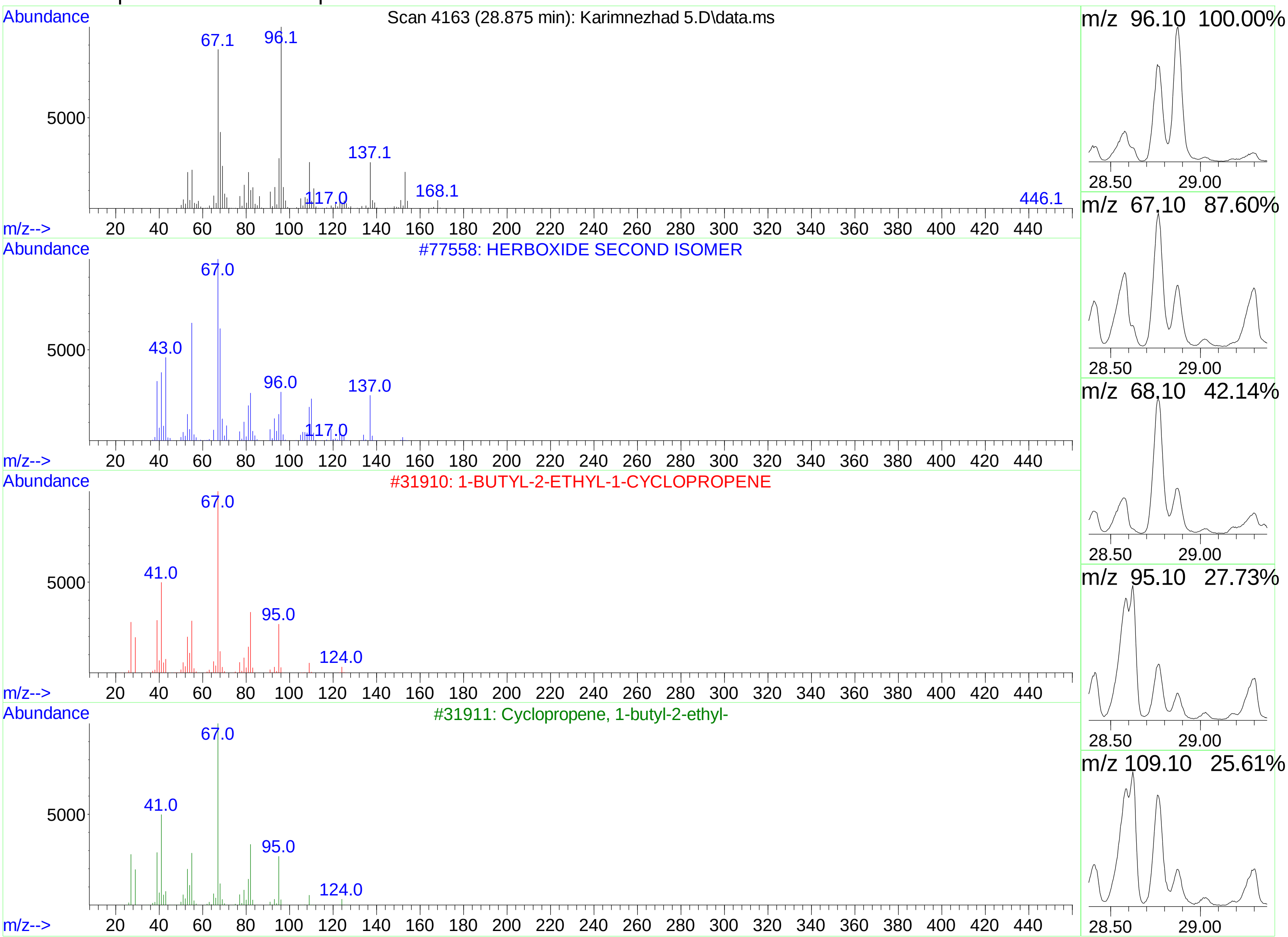


Data File: D:\msdchem\1\data\Karimnezhad 5.D
Sample : M15

Peak Number: 25 at 28.875 min Area: 28165775 Area % 0.14

| The 3 best hits from each library. | Ref\# | CAS\# | Qual |
|---|---|---|---|
| D:\Database\W10N14.L | | | |
| 1 HERBOXIDE SECOND ISOMER | 77558 | 013679-86-2 | 43 |
| 2 1-BUTYL-2-ETHYL-1-CYCLOPROPENE | 31910 | 050915-91-8 | 30 |
| 3 Cyclopropene, 1-butyl-2-ethyl- | 31911 | 050915-91-8 | 30 |

Unknown Spectrum based on Apex

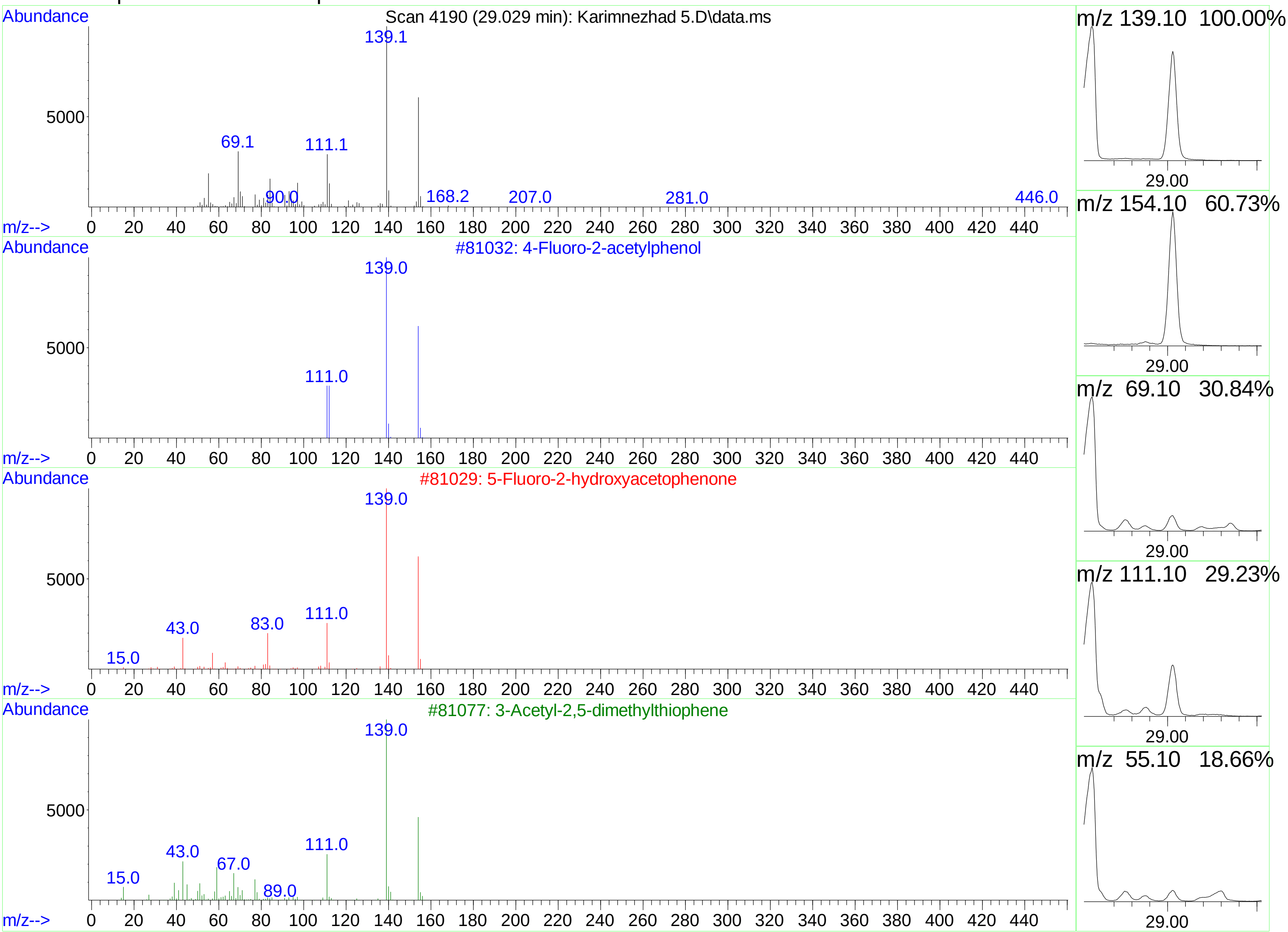


Data File: D:\msdchem\1\data\Karimnezhad 5.D
Sample : M15

Peak Number: 26 at 29.029 min Area: 37782010 Area % 0.19

| The 3 best hits from each library. | Ref\# | CAS\# | Qual |
|---|---|---|---|
| D:\Database\W10N14.L | | | |
| 1 4-Fluoro-2-acetylphenol | 81032 | 000394-32-1 | 86 |
| 2 5-Fluoro-2-hydroxyacetophenone | 81029 | 000394-32-1 | 74 |
| 3 3-Acetyl-2,5-dimethylthiophene | 81077 | 002530-10-1 | 72 |

Unknown Spectrum based on Apex

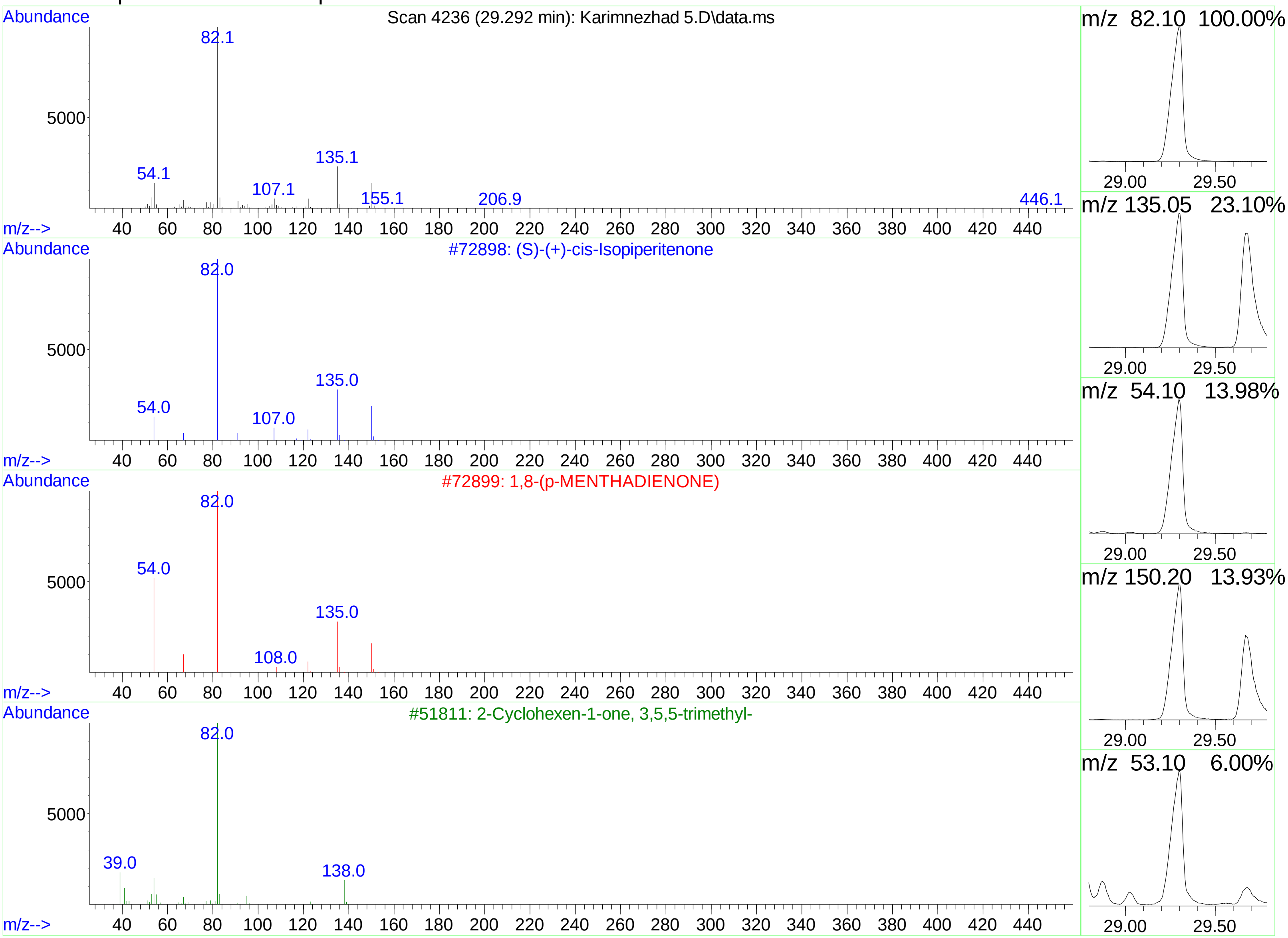


Data File: D:\msdchem\1\data\Karimnezhad 5.D
Sample : M15

Peak Number: 27 at 29.292 min Area: 249651563 Area % 1.24

| The 3 best hits from each library. | Ref# | CAS# | Qual |
|---|---|---|---|
| D:\Database\W10N14.L | | | |
| 1 (S)-(+)-cis-Isopiperitenone | 72898 | 2000072-89-8 | 91 |
| 2 1,8-(p-MENTHADIENONE) | 72899 | 2000072-89-9 | 72 |
| 3 2-Cyclohexen-1-one, 3,5,5-trimet... | 51811 | 000078-59-1 | 50 |

Unknown Spectrum based on Apex

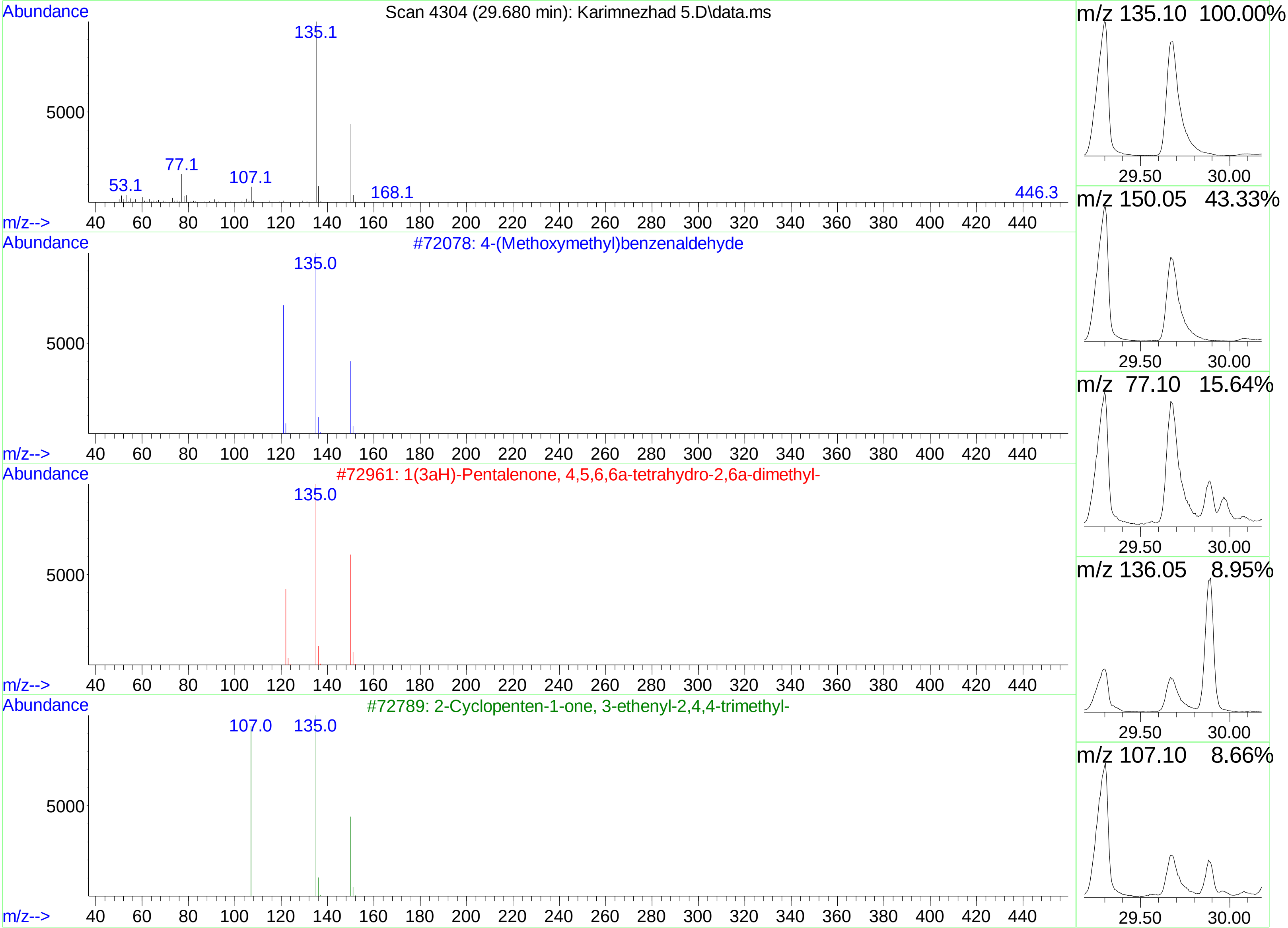


Data File: D:\msdchem\1\data\Karimnezhad 5.D
Sample : M15

Peak Number: 28 at 29.680 min Area: 60444871 Area % 0.30

| The 3 best hits from each library. | Ref\# | CAS\# | Qual |
|---|---|---|---|
| D:\Database\W10N14.L | | | |
| 1 4-(Methoxymethyl)benzenaldehyde | 72078 | 2000072-07-8 | 90 |
| 2 1(3aH)-Pentalenone, 4,5,6,6a-tet... | 72961 | 070640-02-7 | 90 |
| 3 2-Cyclopenten-1-one, 3-ethenyl-2... | 72789 | 104642-15-1 | 90 |

Unknown Spectrum based on Apex

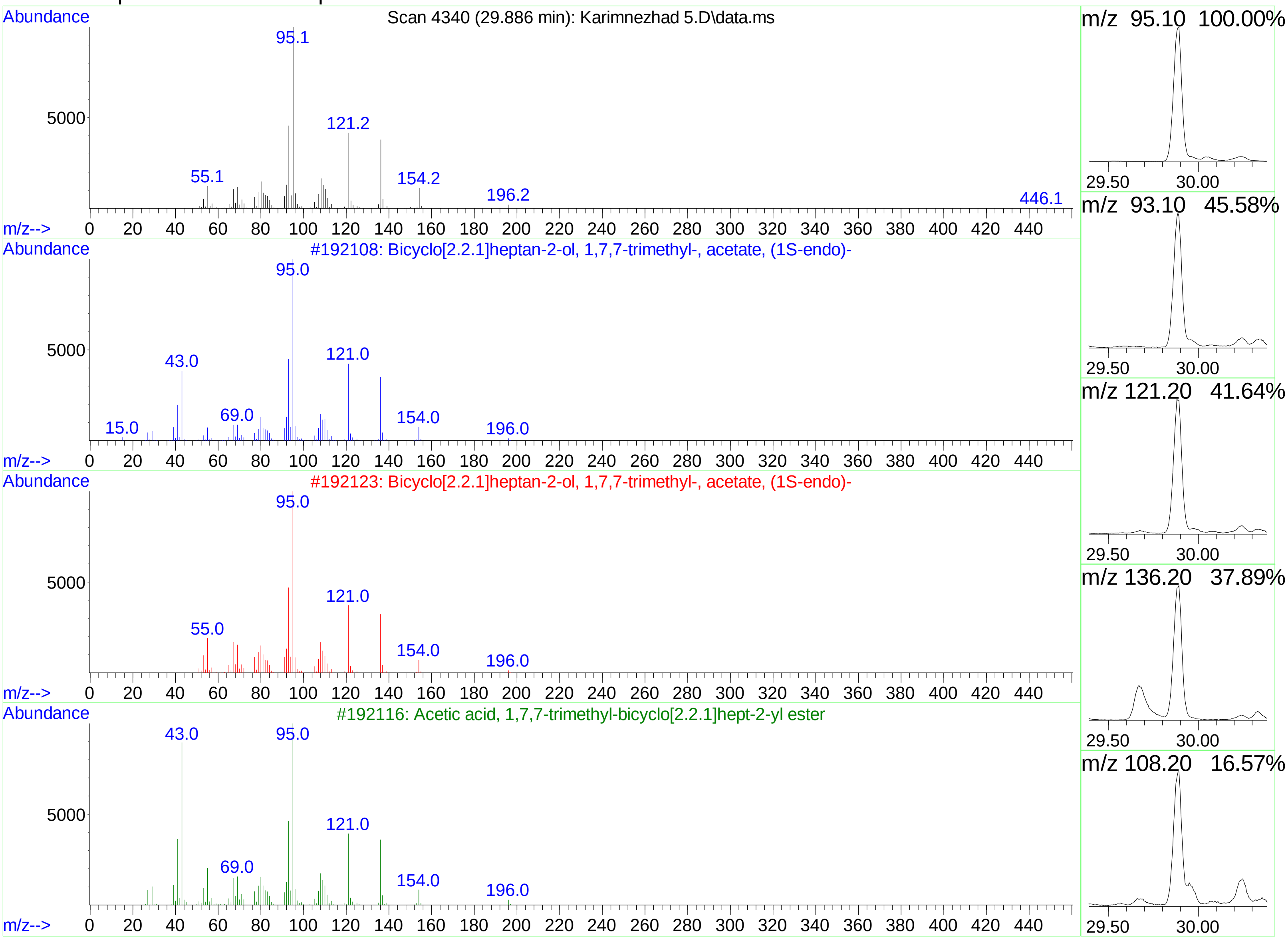


Data File: D:\msdchem\1\data\Karimnezhad 5.D
Sample : M15

Peak Number: 29 at 29.886 min Area: 114962600 Area % 0.57

| The 3 best hits from each library. | Ref\# | CAS\# | Qual |
|---|---|---|---|
| D:\Database\W10N14.L | | | |
| 1 Bicyclo[2.2.1]heptan-2-ol, 1,7,7... | 192108 | 005655-61-8 | 99 |
| 2 Bicyclo[2.2.1]heptan-2-ol, 1,7,7... | 192123 | 005655-61-8 | 99 |
| 3 Acetic acid, 1,7,7-trimethyl-bic... | 192116 | 092618-89-8 | 99 |

Unknown Spectrum based on Apex

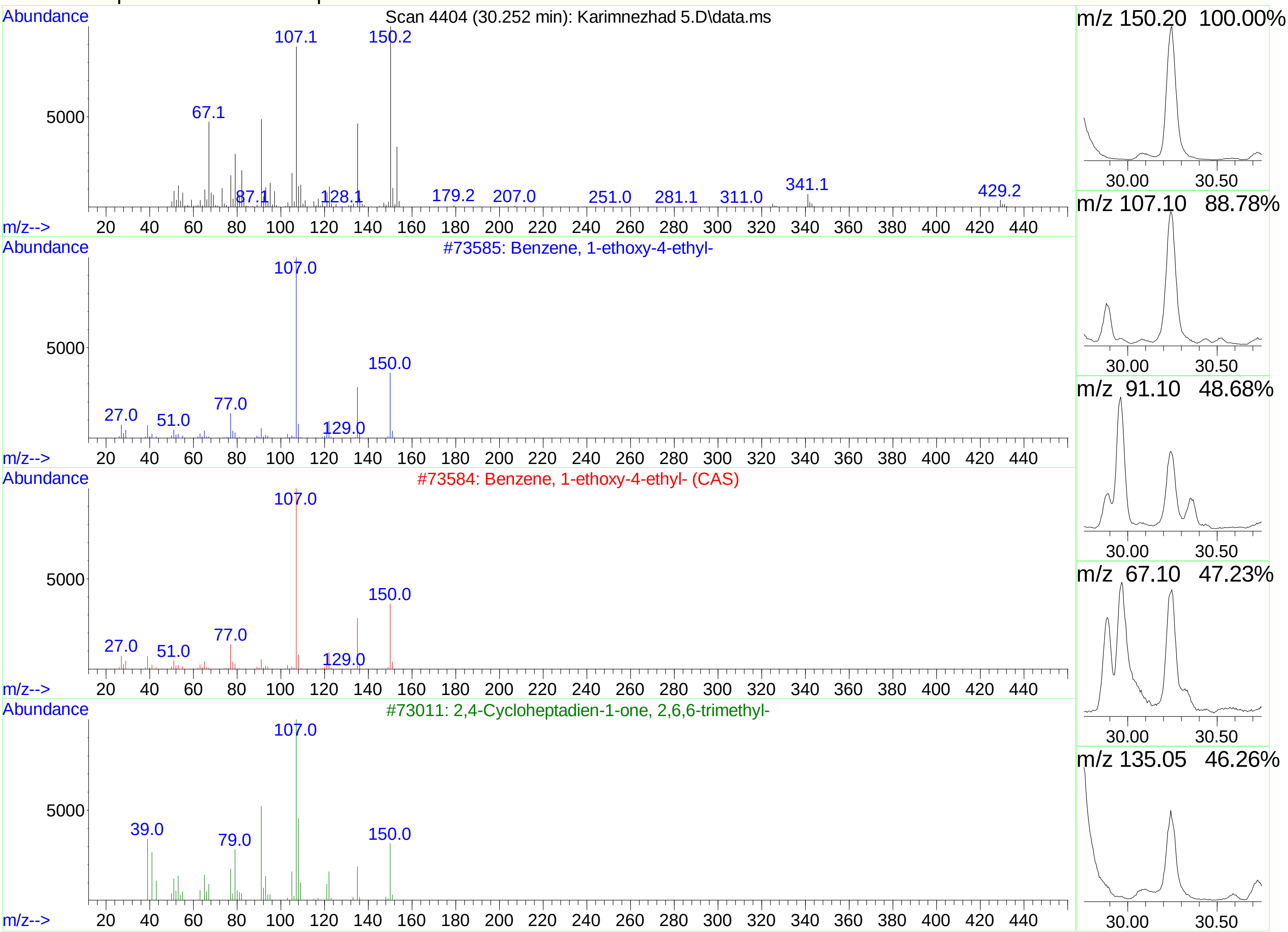


Data File: D:\msdchem\1\data\Karimnezhad 5.D
Sample : M15

Peak Number: 30 at 30.252 min Area: 68459080 Area % 0.34

The 3 best hits from each library. Ref\# CAS\# Qual

--------------------------------------------------------------------

D:\Database\W10N14.L

| | | Ref\# | CAS\# | Qual |
|---|---|---|---|---|
| 1 | Benzene, 1-ethoxy-4-ethyl- | 73585 | 001585-06-4 | 93 |
| 2 | Benzene, 1-ethoxy-4-ethyl- (CAS) | 73584 | 001585-06-4 | 93 |
| 3 | 2,4-Cycloheptadien-1-one, 2,6,6-... | 73011 | 000503-93-5 | 70 |

Unknown Spectrum based on Apex

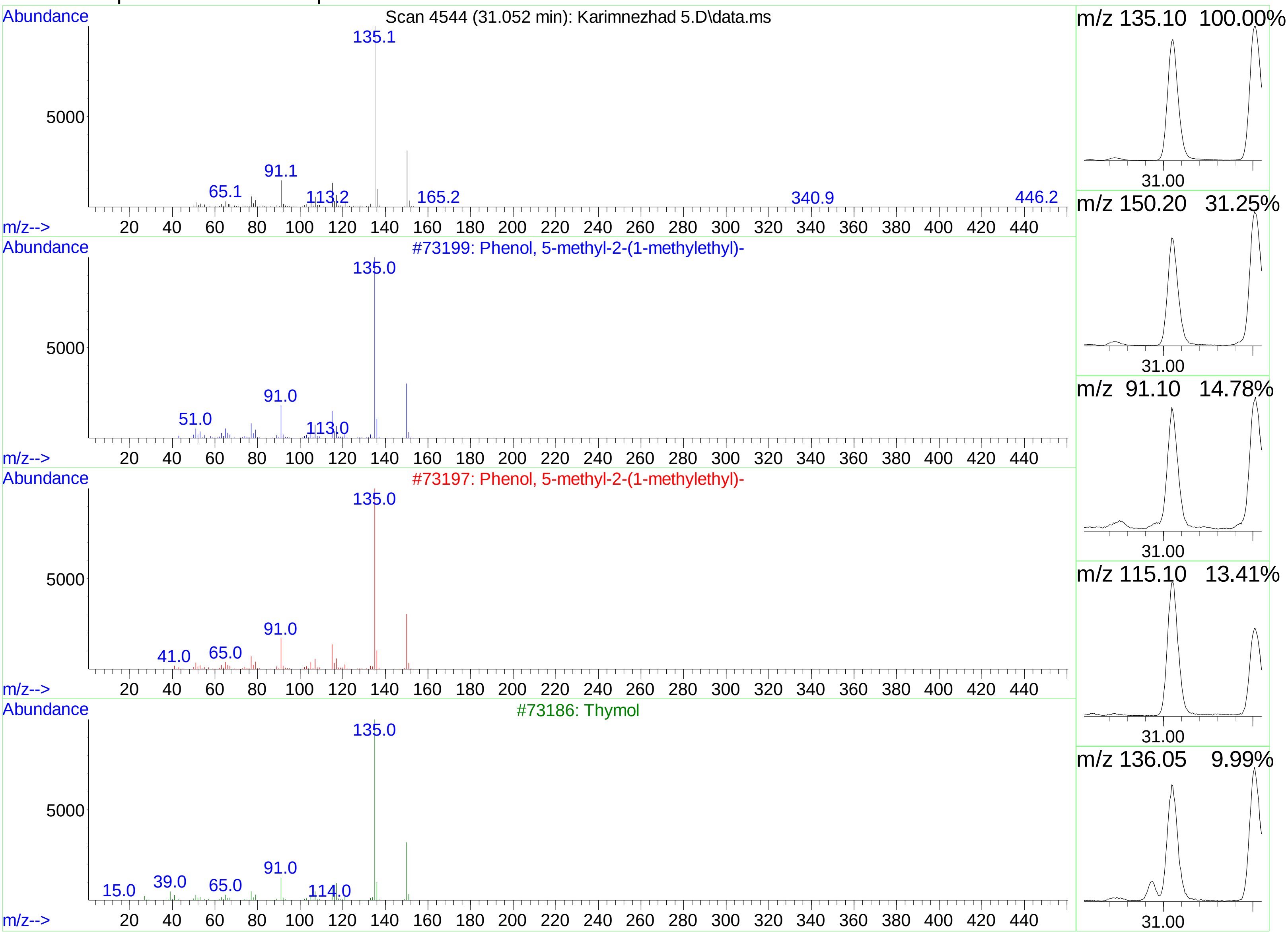


Data File: D:\msdchem\1\data\Karimnezhad 5.D
Sample : M15

Peak Number: 31 at 31.052 min Area: 74024858 Area % 0.37

| The 3 best hits from each library. | Ref# | CAS# | Qual |
|---|---|---|---|
| D:\Database\W10N14.L | | | |
| 1 Phenol, 5-methyl-2-(1-methylethyl)- | 73199 | 000089-83-8 | 95 |
| 2 Phenol, 5-methyl-2-(1-methylethyl)- | 73197 | 000089-83-8 | 94 |
| 3 Thymol | 73186 | 000089-83-8 | 94 |

# Library Search Report - ChemStation Integrator

Unknown Spectrum based on Apex

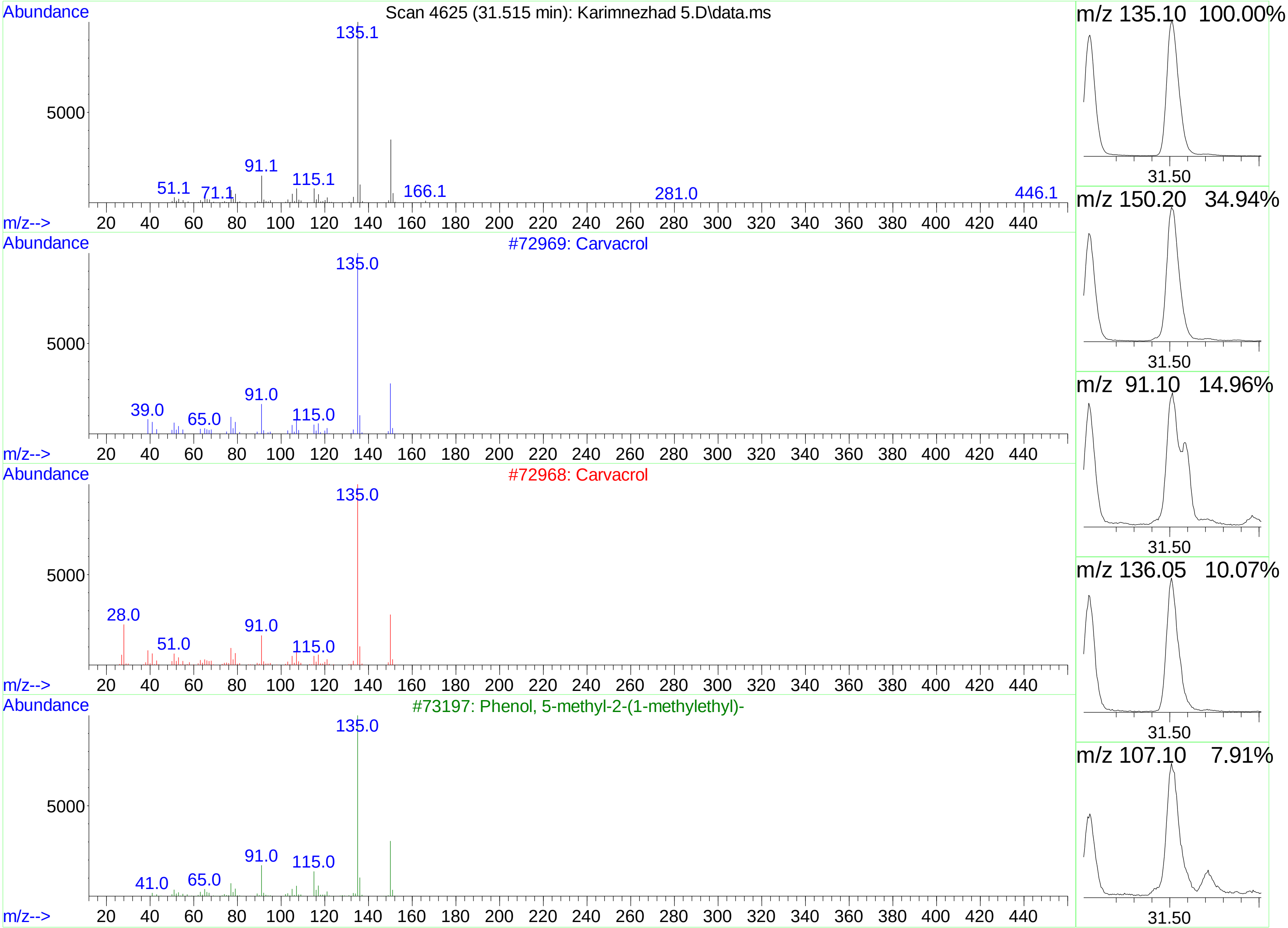


Data File: D:\msdchem\1\data\Karimnezhad 5.D
Sample : M15

Peak Number: 32 at 31.515 min Area: 84281469 Area % 0.42

The 3 best hits from each library. Ref\# CAS\# Qual

-----------------------------------------------------------------------

D:\Database\W10N14.L

| | | | |
|---|---|---|---|
| 1 Carvacrol | 72969 | 000499-75-2 | 95 |
| 2 Carvacrol | 72968 | 000499-75-2 | 94 |
| 3 Phenol, 5-methyl-2-(1-methylethyl)- | 73197 | 000089-83-8 | 91 |

# Library Search Report - ChemStation Integrator

Unknown Spectrum based on Apex

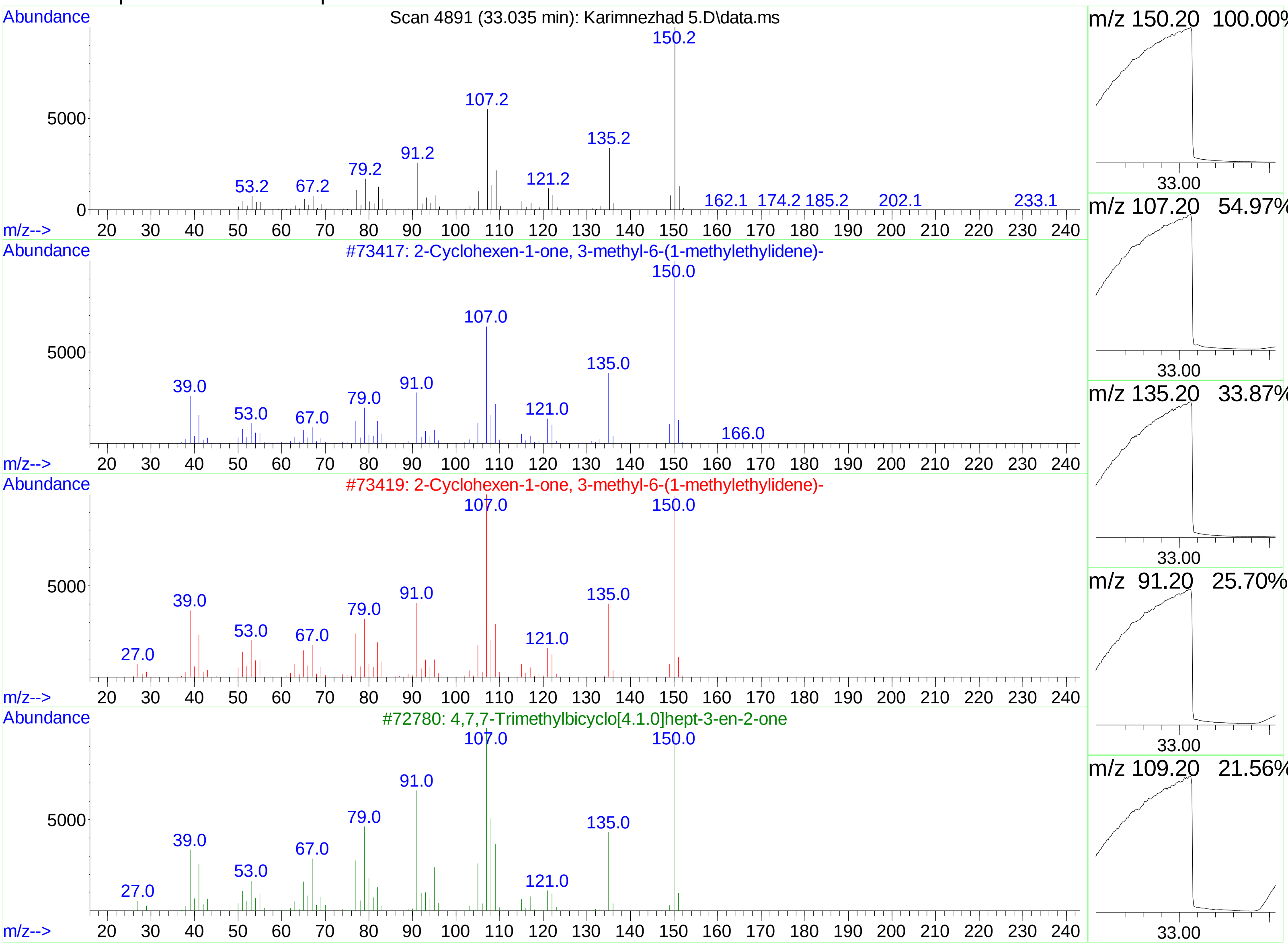


Data File: D:\msdchem\1\data\Karimnezhad 5.D
Sample : M15

Peak Number: 33 at 33.035 min Area: 7761297694 Area % 38.41

| The 3 best hits from each library. | Ref# | CAS# | Qual |
|---|---|---|---|
| D:\Database\W10N14.L | | | |
| 1 2-Cyclohexen-1-one, 3-methyl-6-(... | 73417 | 000491-09-8 | 98 |
| 2 2-Cyclohexen-1-one, 3-methyl-6-(... | 73419 | 000491-09-8 | 95 |
| 3 4,7,7-Trimethylbicyclo[4.1.0]hep... | 72780 | 081800-50-2 | 93 |

Unknown Spectrum based on Apex

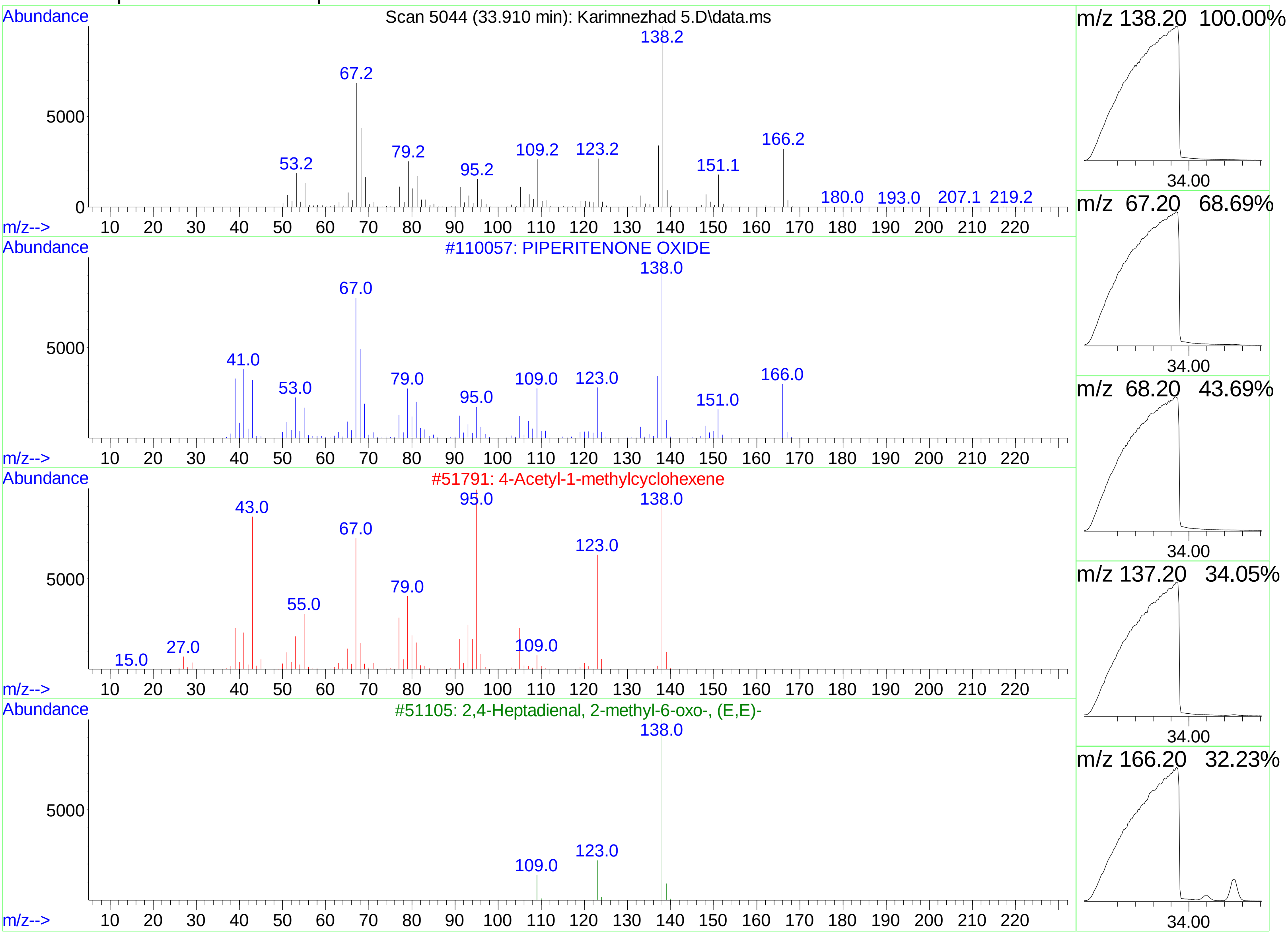


Data File: D:\msdchem\1\data\Karimnezhad 5.D
Sample : M15

Peak Number: 34 at 33.910 min Area: 4295763332 Area % 21.26

| The 3 best hits from each library. | Ref\# | CAS\# | Qual |
|---|---|---|---|
| D:\Database\W10N14.L | | | |
| 1 PIPERITENONE OXIDE | 110057 | 003564-96-3 | 98 |
| 2 4-Acetyl-1-methylcyclohexene | 51791 | 006090-09-1 | 60 |
| 3 2,4-Heptadienal, 2-methyl-6-oxo-... | 51105 | 129454-99-5 | 58 |

Unknown Spectrum based on Apex

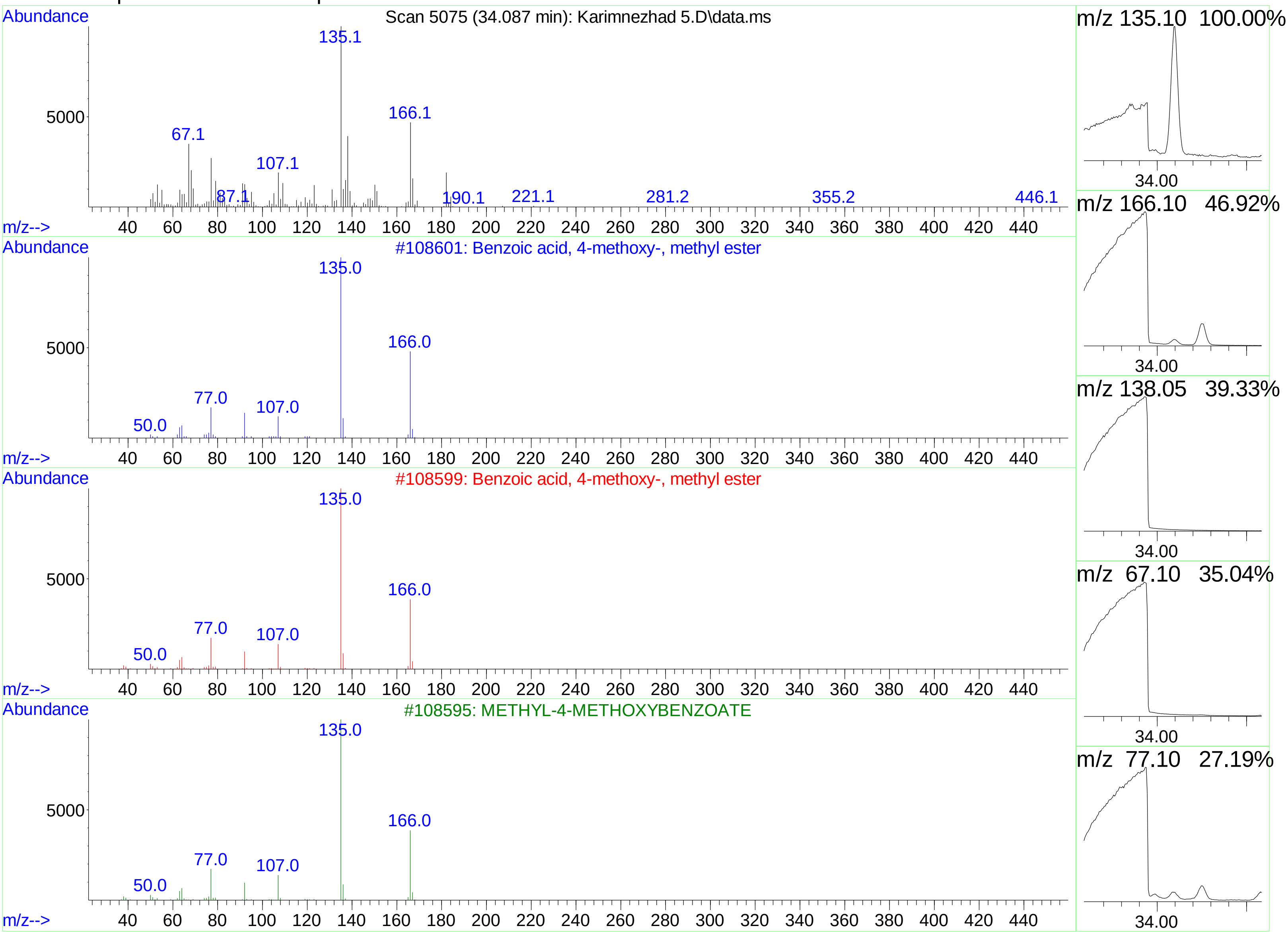


Data File: D:\msdchem\1\data\Karimnezhad 5.D
Sample : M15

Peak Number: 35 at 34.087 min Area: 30010517 Area % 0.15

| The 3 best hits from each library. | Ref# | CAS# | Qual |
|---|---|---|---|
| D:\Database\W10N14.L | | | |
| 1 Benzoic acid, 4-methoxy-, methyl... | 108601 | 000121-98-2 | 60 |
| 2 Benzoic acid, 4-methoxy-, methyl... | 108599 | 000121-98-2 | 60 |
| 3 METHYL-4-METHOXYBENZOATE | 108595 | 000121-98-2 | 60 |

Unknown Spectrum based on Apex

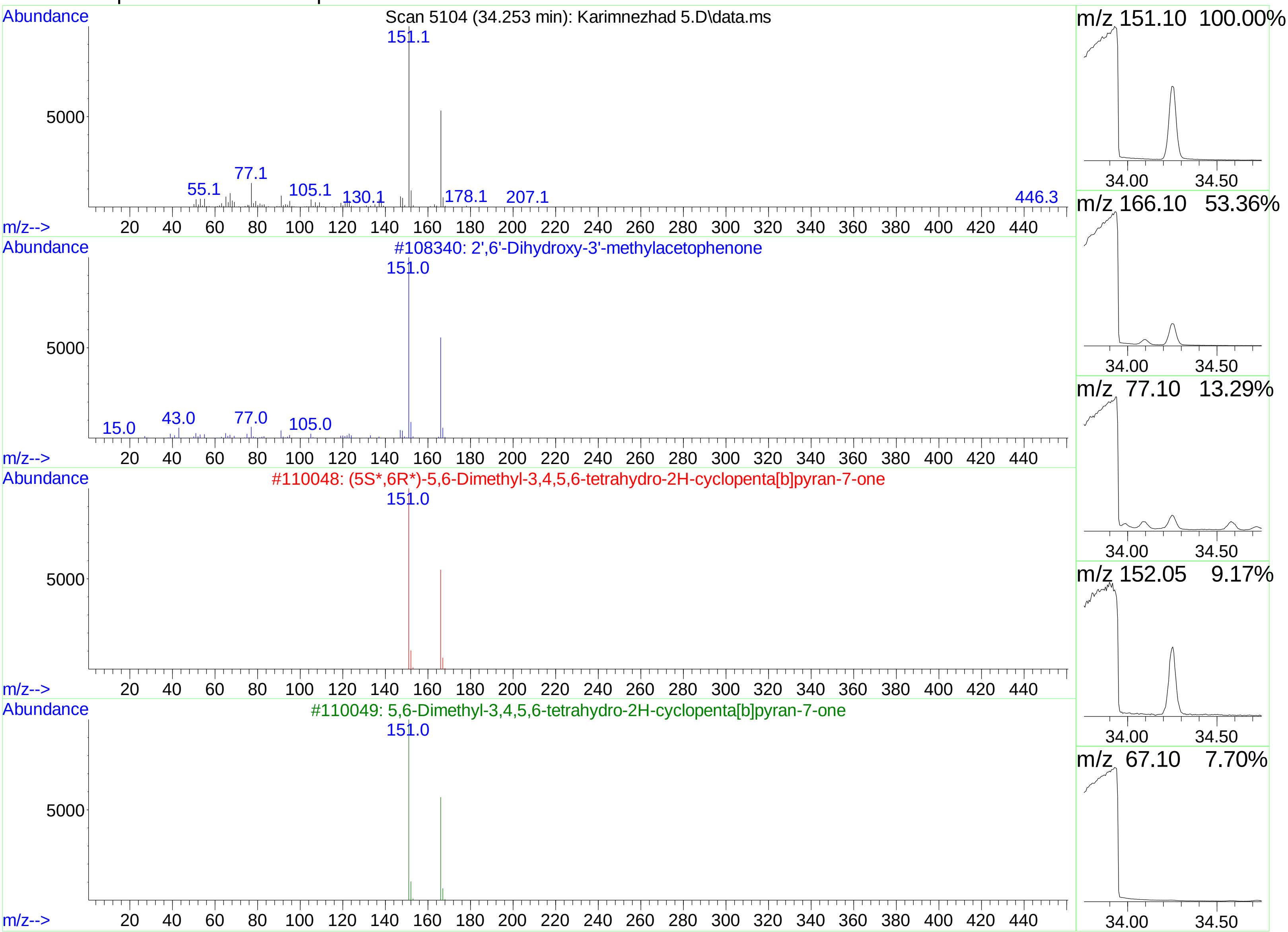


Data File: D:\msdchem\1\data\Karimnezhad 5.D
Sample : M15

Peak Number: 36 at 34.253 min Area: 59115465 Area % 0.29

| The 3 best hits from each library. | Ref# | CAS# | Qual |
|---|---|---|---|
| D:\Database\W10N14.L | | | |
| 1 2',6'-Dihydroxy-3'-methylacetoph... | 108340 | 029183-78-6 | 87 |
| 2 (5S*,6R*)-5,6-Dimethyl-3,4,5,6-t... | 110048 | 2000110-04-8 | 83 |
| 3 5,6-Dimethyl-3,4,5,6-tetrahydro-... | 110049 | 2000110-04-9 | 83 |

Unknown Spectrum based on Apex

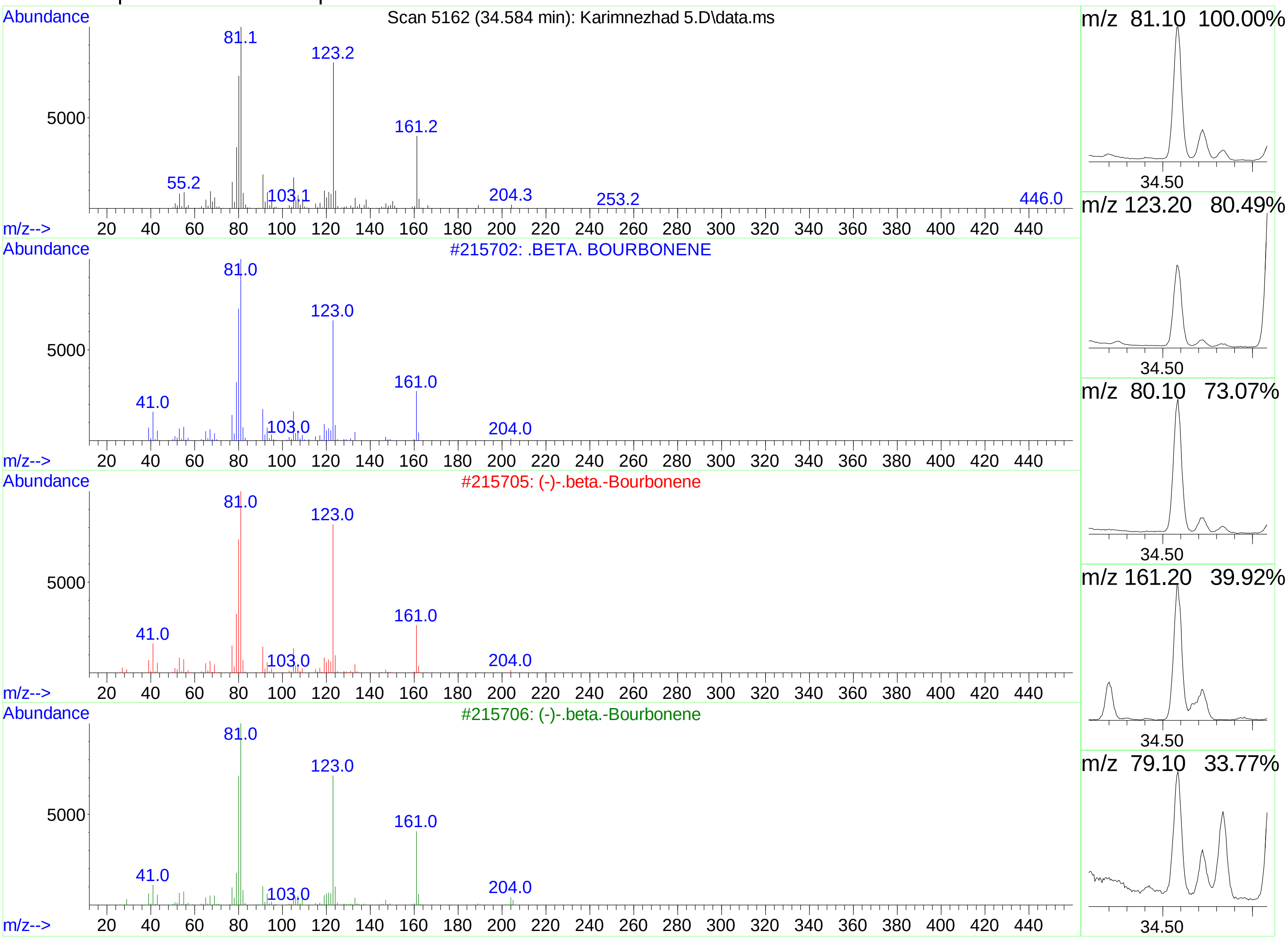


Data File: D:\msdchem\1\data\Karimnezhad 5.D
Sample : M15

Peak Number: 37 at 34.584 min Area: 34514693 Area % 0.17

| The 3 best hits from each library. | Ref\# | CAS\# | Qual |
|---|---|---|---|
| D:\Database\W10N14.L | | | |
| 1 .BETA. BOURBONENE | 215702 | 005208-59-3 | 99 |
| 2 (-)-.beta.-Bourbonene | 215705 | 005208-59-3 | 98 |
| 3 (-)-.beta.-Bourbonene | 215706 | 005208-59-3 | 97 |

Unknown Spectrum based on Apex

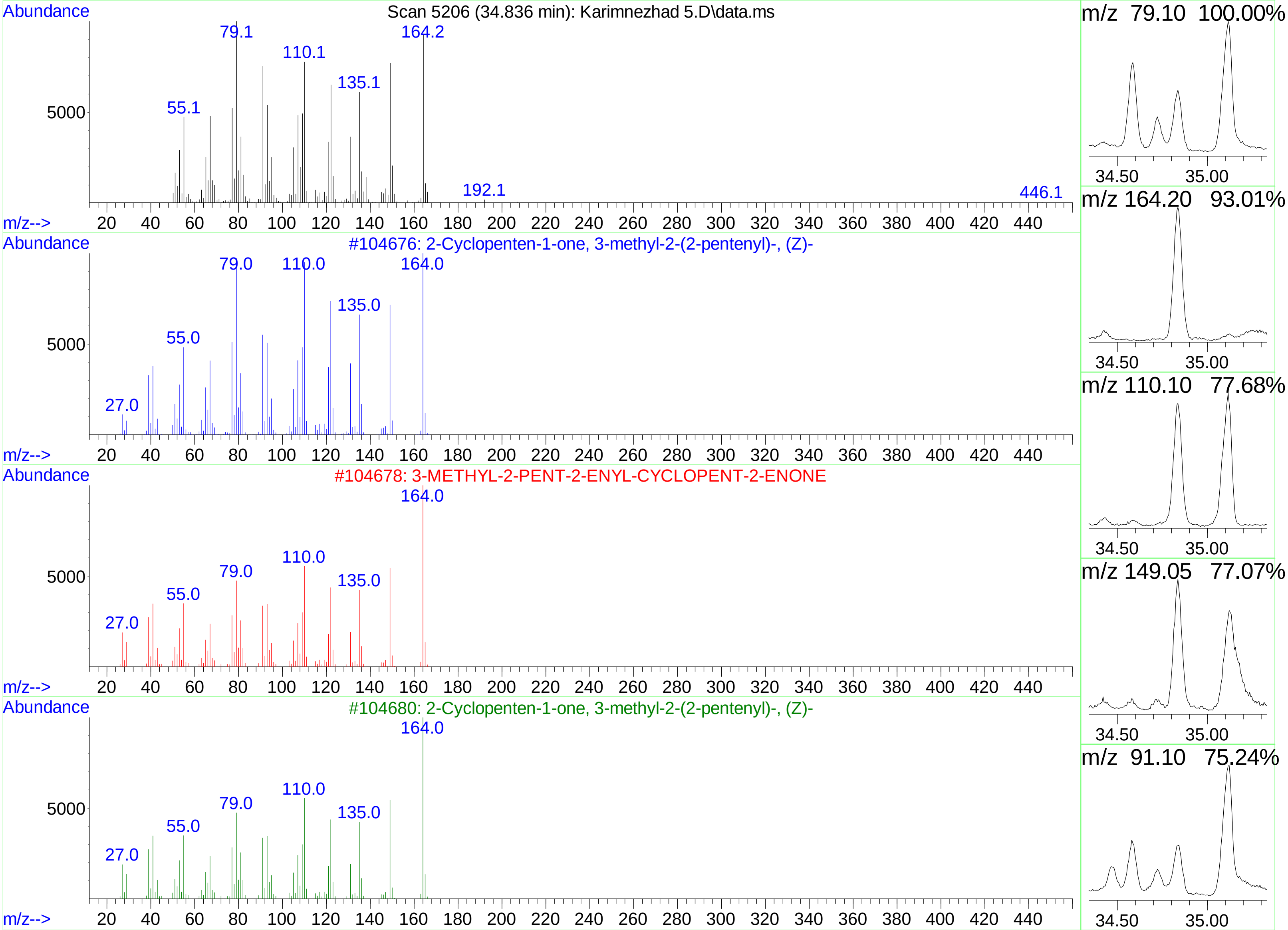


Data File: D:\msdchem\1\data\Karimnezhad 5.D
Sample : M15

Peak Number: 38 at 34.836 min Area: 35931624 Area % 0.18

The 3 best hits from each library. Ref\# CAS\# Qual

--------------------------------------------------------------------

D:\Database\W10N14.L

| | | | | |
|---|---|---|---|---|
| 1 | 2-Cyclopenten-1-one, 3-methyl-2-... | 104676 | 000488-10-8 | 99 |
| 2 | 3-METHYL-2-PENT-2-ENYL-CYCLOPENT... | 104678 | 000488-10-8 | 98 |
| 3 | 2-Cyclopenten-1-one, 3-methyl-2-... | 104680 | 000488-10-8 | 98 |

Unknown Spectrum based on Apex

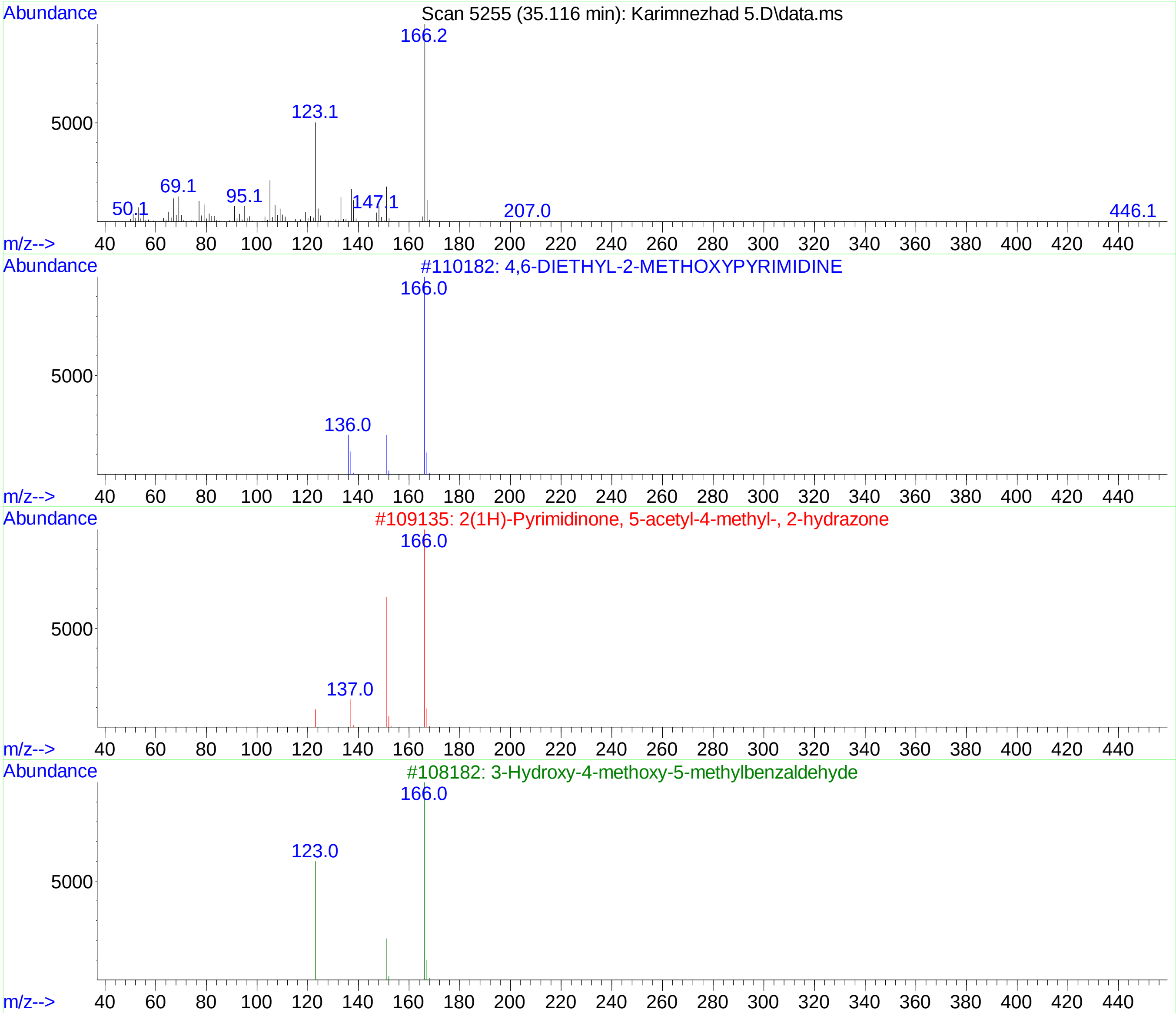


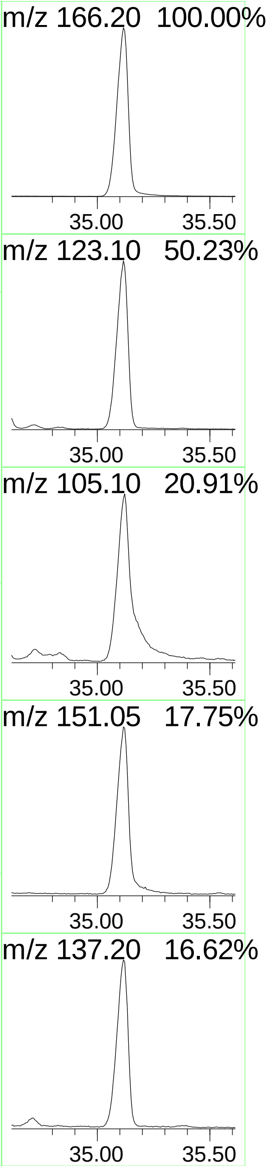


Data File: D:\msdchem\1\data\Karimnezhad 5.D
Sample : M15

Peak Number: 39 at 35.116 min Area: 170437767 Area % 0.84

| The 3 best hits from each library. | Ref\# | CAS\# | Qual |
|---|---|---|---|
| D:\Database\W10N14.L | | | |
| 1 4,6-DIETHYL-2-METHOXYPYRIMIDINE | 110182 | 2000110-18-2 | 72 |
| 2 2(1H)-Pyrimidinone, 5-acetyl-4-m... | 109135 | 093584-03-3 | 64 |
| 3 3-Hydroxy-4-methoxy-5-methylbenz... | 108182 | 2000108-18-2 | 64 |

Unknown Spectrum based on Apex

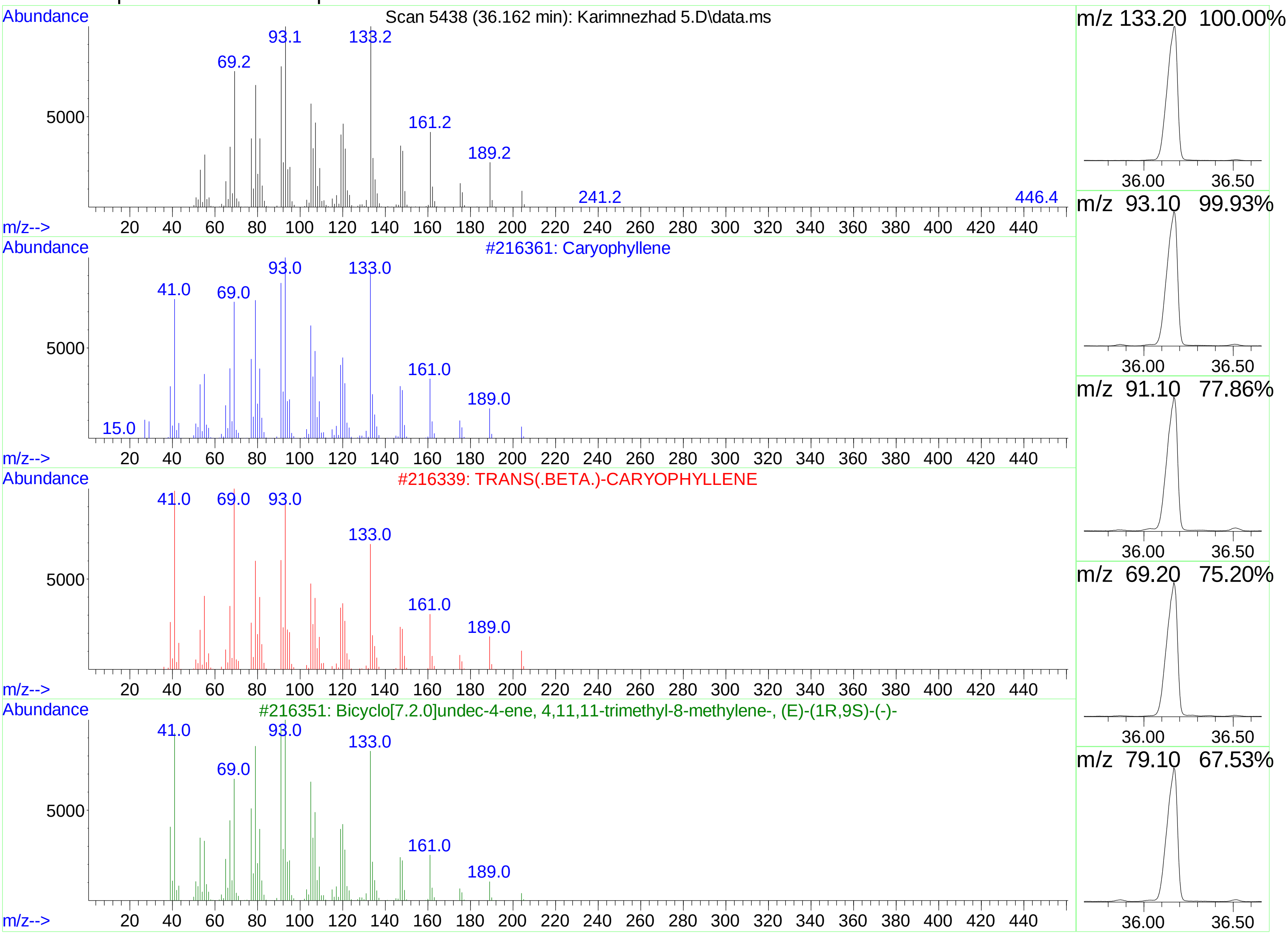


Data File: D:\msdchem\1\data\Karimnezhad 5.D
Sample : M15

Peak Number: 40 at 36.162 min Area: 411797293 Area % 2.04

| The 3 best hits from each library. | Ref\# | CAS\# | Qual |
|---|---|---|---|
| D:\Database\W10N14.L | | | |
| 1 Caryophyllene | 216361 | 000087-44-5 | 99 |
| 2 TRANS(.BETA.)-CARYOPHYLLENE | 216339 | 2000216-33-9 | 99 |
| 3 Bicyclo[7.2.0]undec-4-ene, 4,11,... | 216351 | 000087-44-5 | 99 |

Unknown Spectrum based on Apex

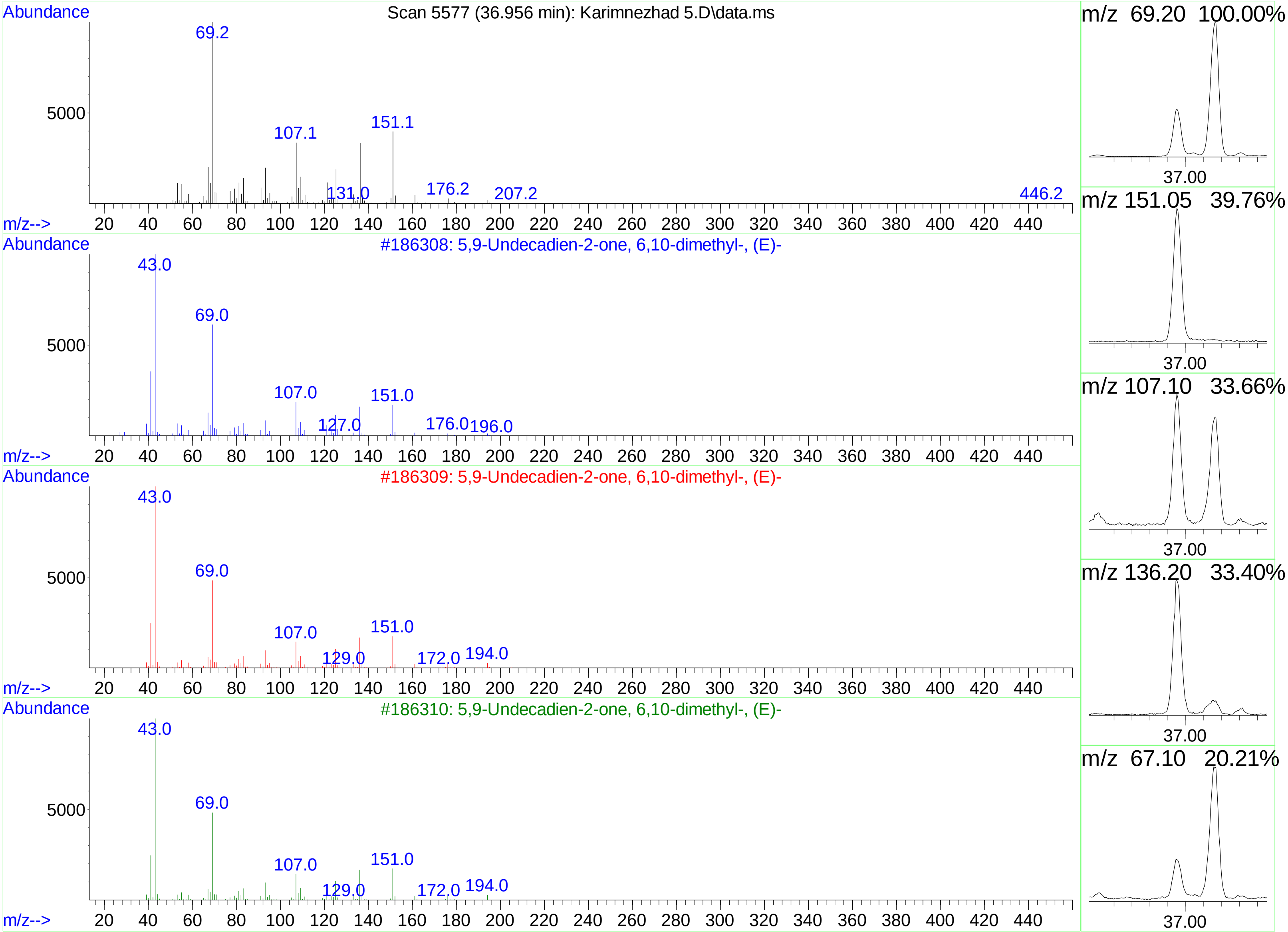


Data File: D:\msdchem\1\data\Karimnezhad 5.D
Sample : M15

Peak Number: 41 at 36.956 min Area: 25635209 Area % 0.13

The 3 best hits from each library. Ref\# CAS\# Qual
-------------------------------------------------------------------
D:\Database\W10N14.L

| | | | | |
|---|---|---|---|---|
| 1 | 5,9-Undecadien-2-one, 6,10-dimet... | 186308 | 003796-70-1 | 93 |
| 2 | 5,9-Undecadien-2-one, 6,10-dimet... | 186309 | 003796-70-1 | 86 |
| 3 | 5,9-Undecadien-2-one, 6,10-dimet... | 186310 | 003796-70-1 | 86 |

Unknown Spectrum based on Apex

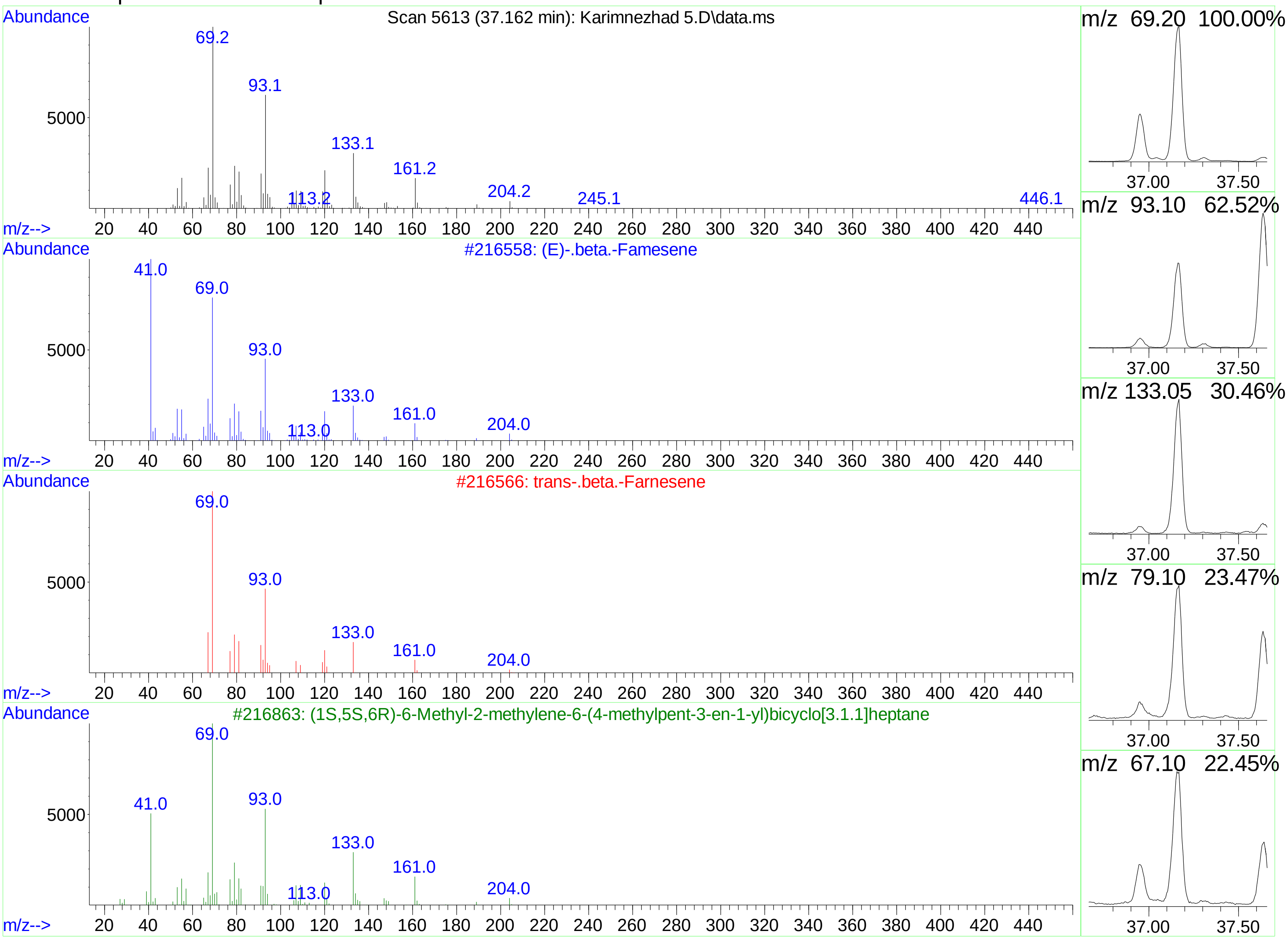


Data File: D:\msdchem\1\data\Karimnezhad 5.D
Sample : M15

Peak Number: 42 at 37.162 min Area: 67691327 Area % 0.33

The 3 best hits from each library. Ref\# CAS\# Qual

---

D:\Database\W10N14.L

| | | | |
|---|---|---|---|
| 1 (E)-.beta.-Famesene | 216558 | 018794-84-8 | 96 |
| 2 trans-.beta.-Farnesene | 216566 | 000502-60-3 | 96 |
| 3 (1S,5S,6R)-6-Methyl-2-methylene-... | 216863 | 015438-94-5 | 95 |

Unknown Spectrum based on Apex

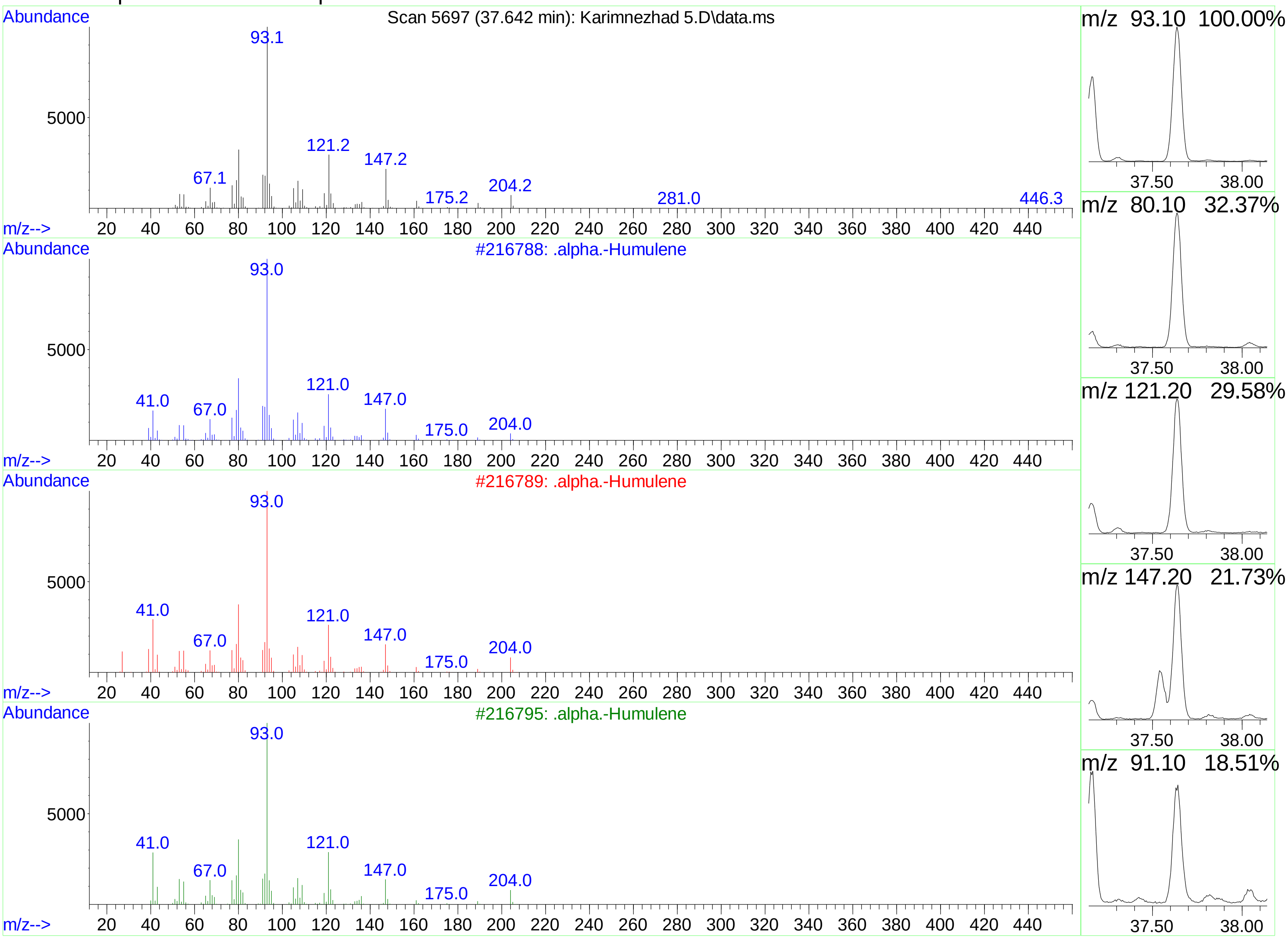


Data File: D:\msdchem\1\data\Karimnezhad 5.D
Sample : M15

Peak Number: 43 at 37.642 min Area: 68770008 Area % 0.34

| The 3 best hits from each library. | Ref\# | CAS\# | Qual |
|---|---|---|---|
| D:\Database\W10N14.L | | | |
| 1 .alpha.-Humulene | 216788 | 006753-98-6 | 99 |
| 2 .alpha.-Humulene | 216789 | 006753-98-6 | 98 |
| 3 .alpha.-Humulene | 216795 | 006753-98-6 | 98 |

Unknown Spectrum based on Apex

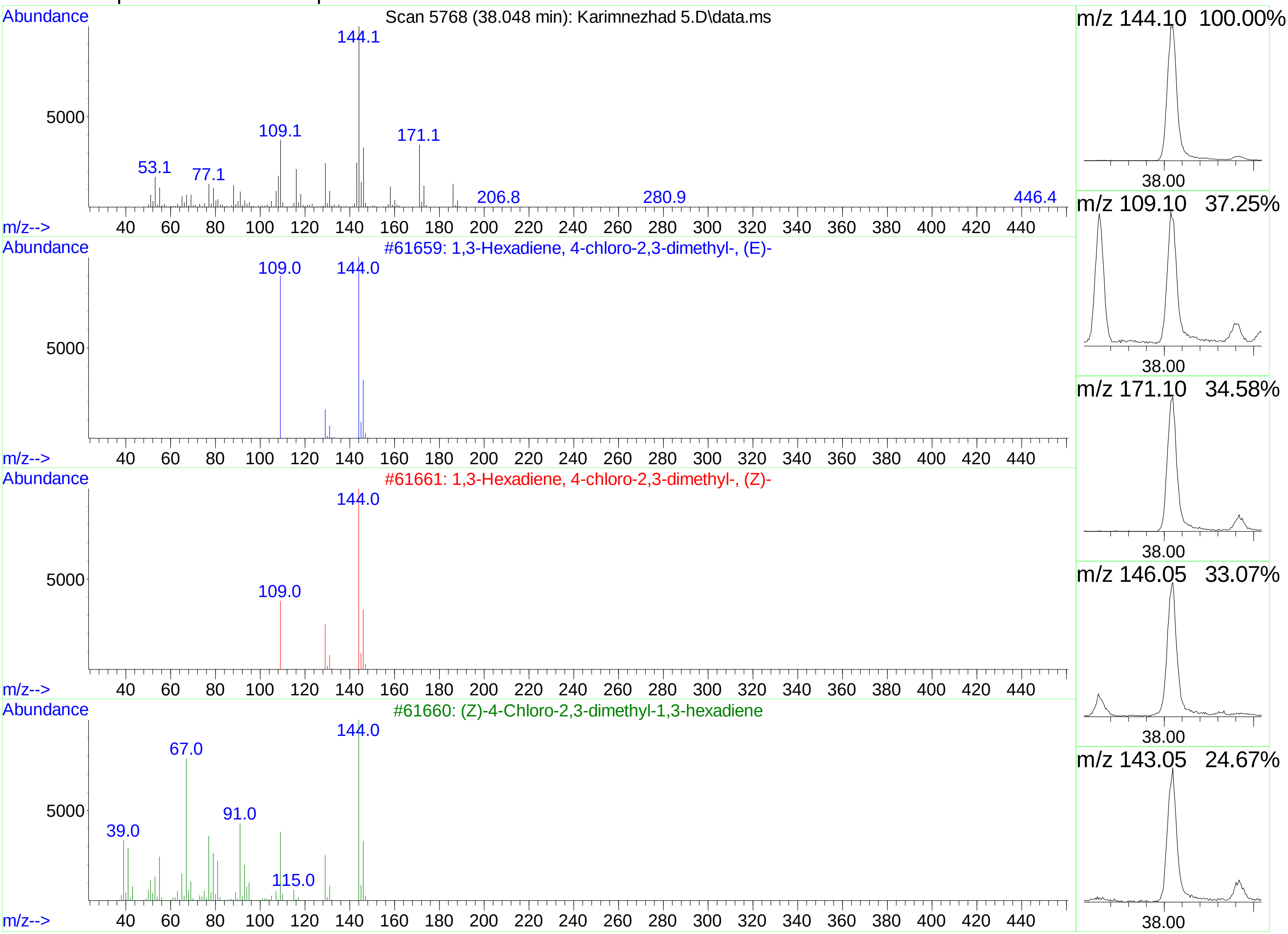


Data File: D:\msdchem\1\data\Karimnezhad 5.D
Sample : M15

Peak Number: 44 at 38.048 min Area: 27643447 Area % 0.14

| The 3 best hits from each library. | Ref\# | CAS\# | Qual |
|---|---|---|---|
| D:\Database\W10N14.L | | | |
| 1 1,3-Hexadiene, 4-chloro-2,3-dime... | 61659 | 105949-72-2 | 60 |
| 2 1,3-Hexadiene, 4-chloro-2,3-dime... | 61661 | 105977-13-7 | 53 |
| 3 (Z)-4-Chloro-2,3-dimethyl-1,3-he... | 61660 | 105977-13-7 | 49 |

Unknown Spectrum based on Apex

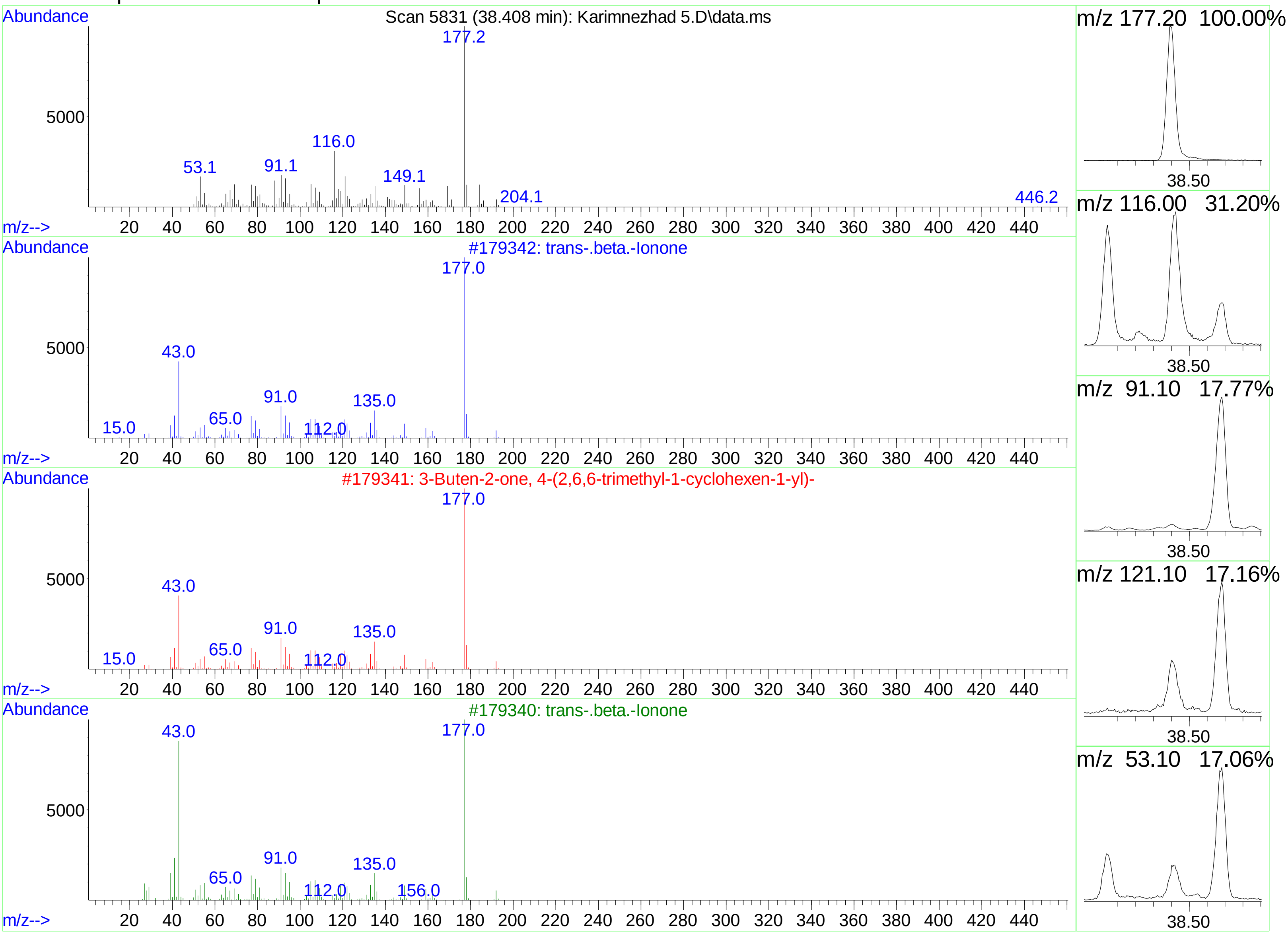


Data File: D:\msdchem\1\data\Karimnezhad 5.D
Sample : M15

Peak Number: 45 at 38.408 min Area: 22866788 Area % 0.11

| The 3 best hits from each library. | Ref\# | CAS\# | Qual |
|---|---|---|---|
| D:\Database\W10N14.L | | | |
| 1 trans-.beta.-Ionone | 179342 | 000079-77-6 | 97 |
| 2 3-Buten-2-one, 4-(2,6,6-trimethy... | 179341 | 014901-07-6 | 97 |
| 3 trans-.beta.-Ionone | 179340 | 000079-77-6 | 96 |

Unknown Spectrum based on Apex

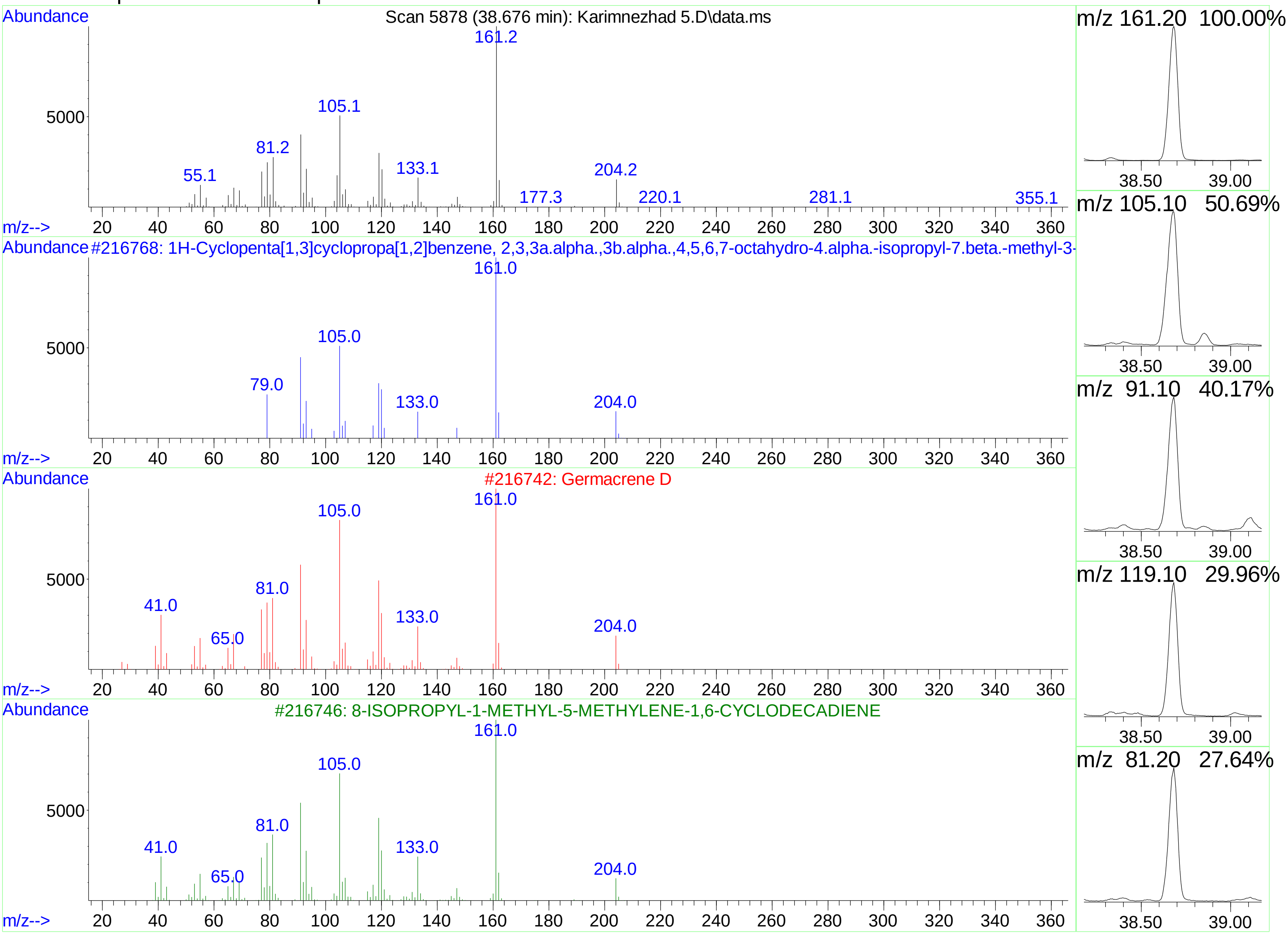


Data File: D:\msdchem\1\data\Karimnezhad 5.D
Sample : M15

Peak Number: 46 at 38.676 min Area: 163170913 Area % 0.81

| The 3 best hits from each library. | Ref# | CAS# | Qual |
|---|---|---|---|
| D:\Database\W10N14.L | | | |
| 1 1H-Cyclopenta[1,3]cyclopropa[1,2... | 216768 | 013744-15-5 | 99 |
| 2 Germacrene D | 216742 | 023986-74-5 | 99 |
| 3 8-ISOPROPYL-1-METHYL-5-METHYLENE... | 216746 | 023986-74-5 | 98 |

Unknown Spectrum based on Apex

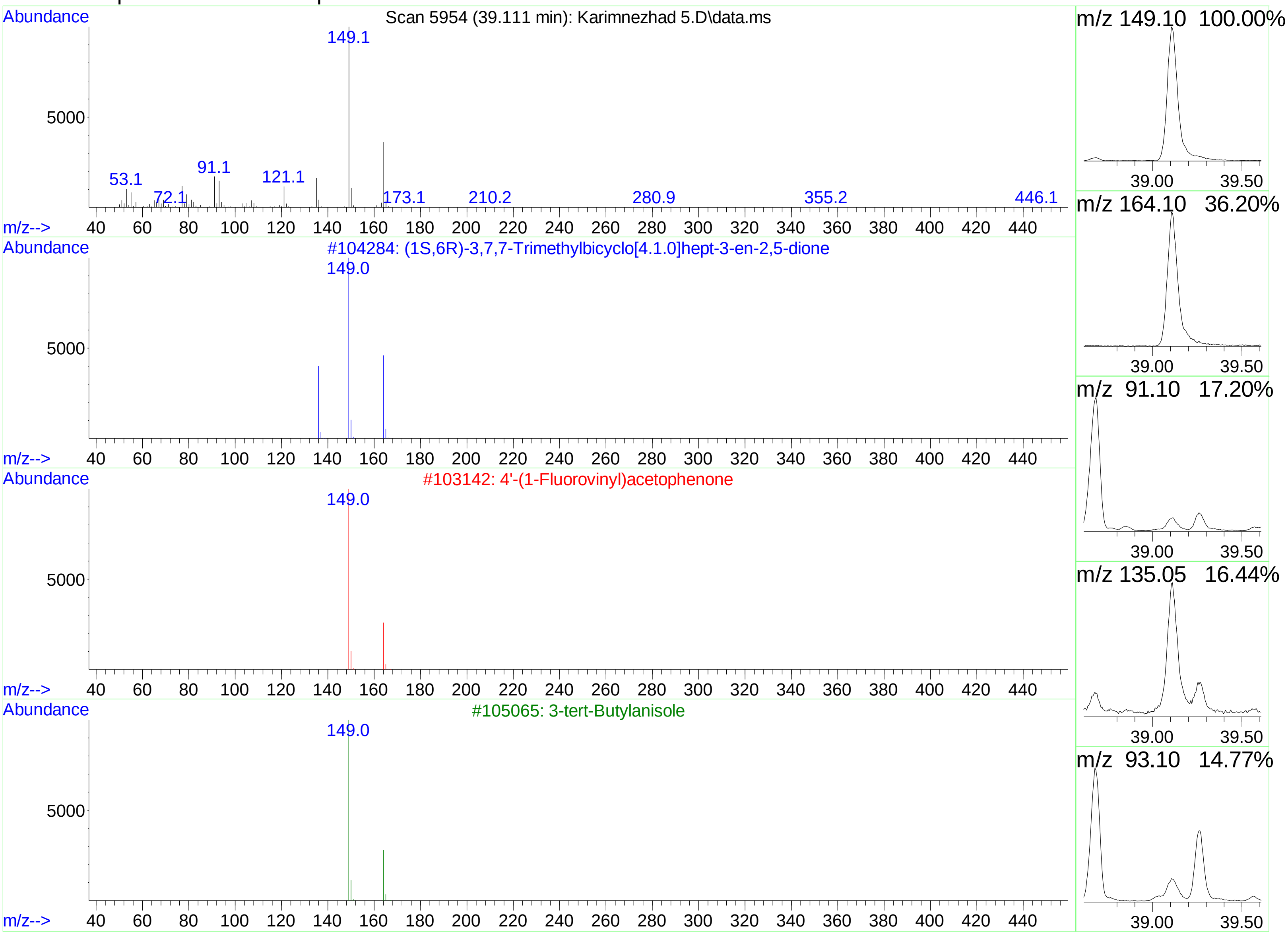


Data File: D:\msdchem\1\data\Karimnezhad 5.D
Sample : M15

Peak Number: 47 at 39.111 min Area: 23253834 Area % 0.12

| The 3 best hits from each library. | Ref# | CAS# | Qual |
|---|---|---|---|
| D:\Database\W10N14.L | | | |
| 1 (1S,6R)-3,7,7-Trimethylbicyclo[4... | 104284 | 2000104-28-4 | 90 |
| 2 4'-(1-Fluorovinyl)acetophenone | 103142 | 2000103-14-2 | 83 |
| 3 3-tert-Butylanisole | 105065 | 2000105-06-5 | 83 |

Unknown Spectrum based on Apex

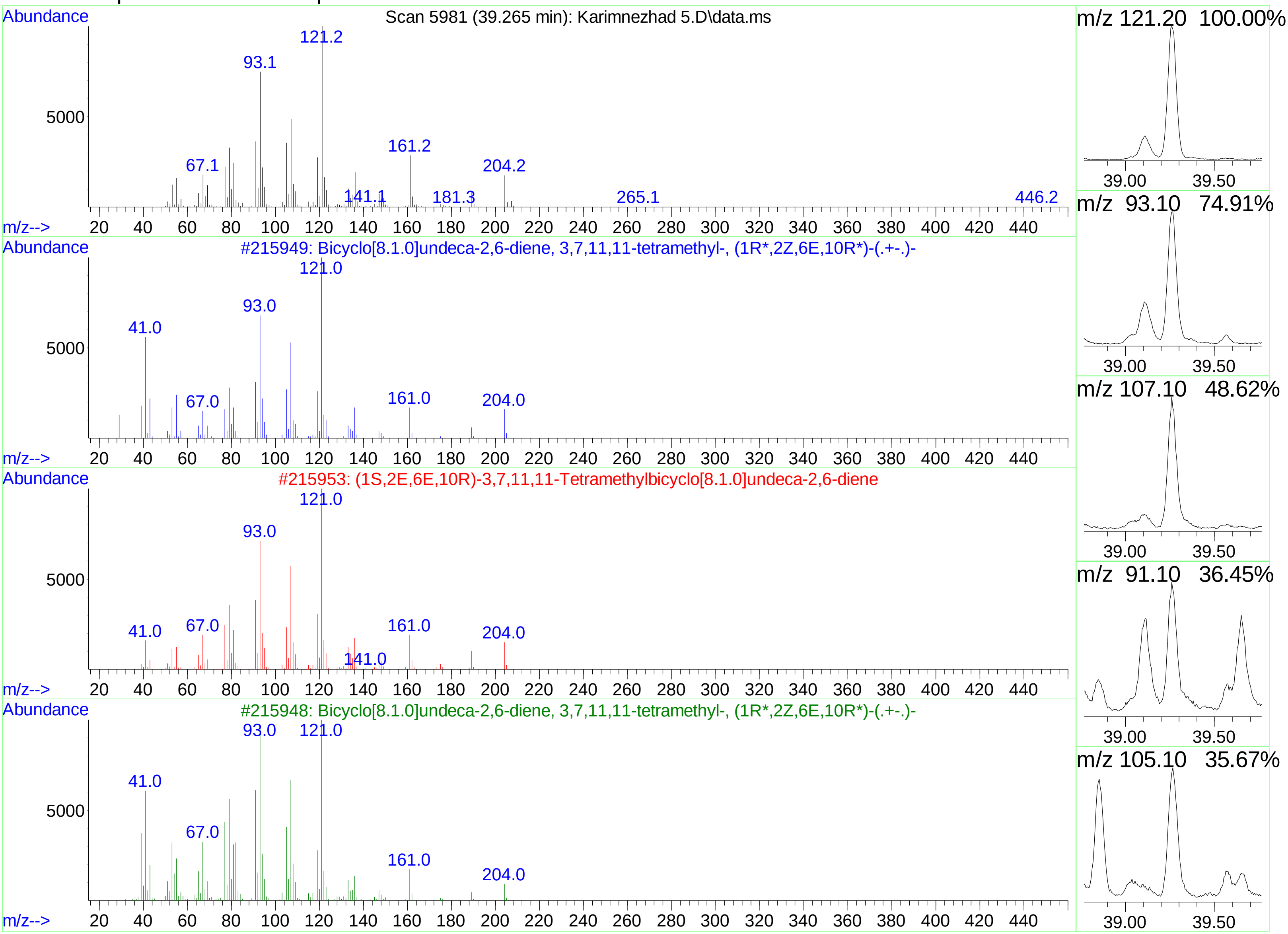


Data File: D:\msdchem\1\data\Karimnezhad 5.D
Sample : M15

Peak Number: 48 at 39.265 min Area: 40696968 Area % 0.20

The 3 best hits from each library. Ref\# CAS\# Qual

-----------------------------------------------------------------

D:\Database\W10N14.L

| | | | | |
|---|---|---|---|---|
| 1 | Bicyclo[8.1.0]undeca-2,6-diene, ... | 215949 | 100762-46-7 | 99 |
| 2 | (1S,2E,6E,10R)-3,7,11,11-Tetrame... | 215953 | 024703-35-3 | 97 |
| 3 | Bicyclo[8.1.0]undeca-2,6-diene, ... | 215948 | 100762-46-7 | 97 |

Unknown Spectrum based on Apex

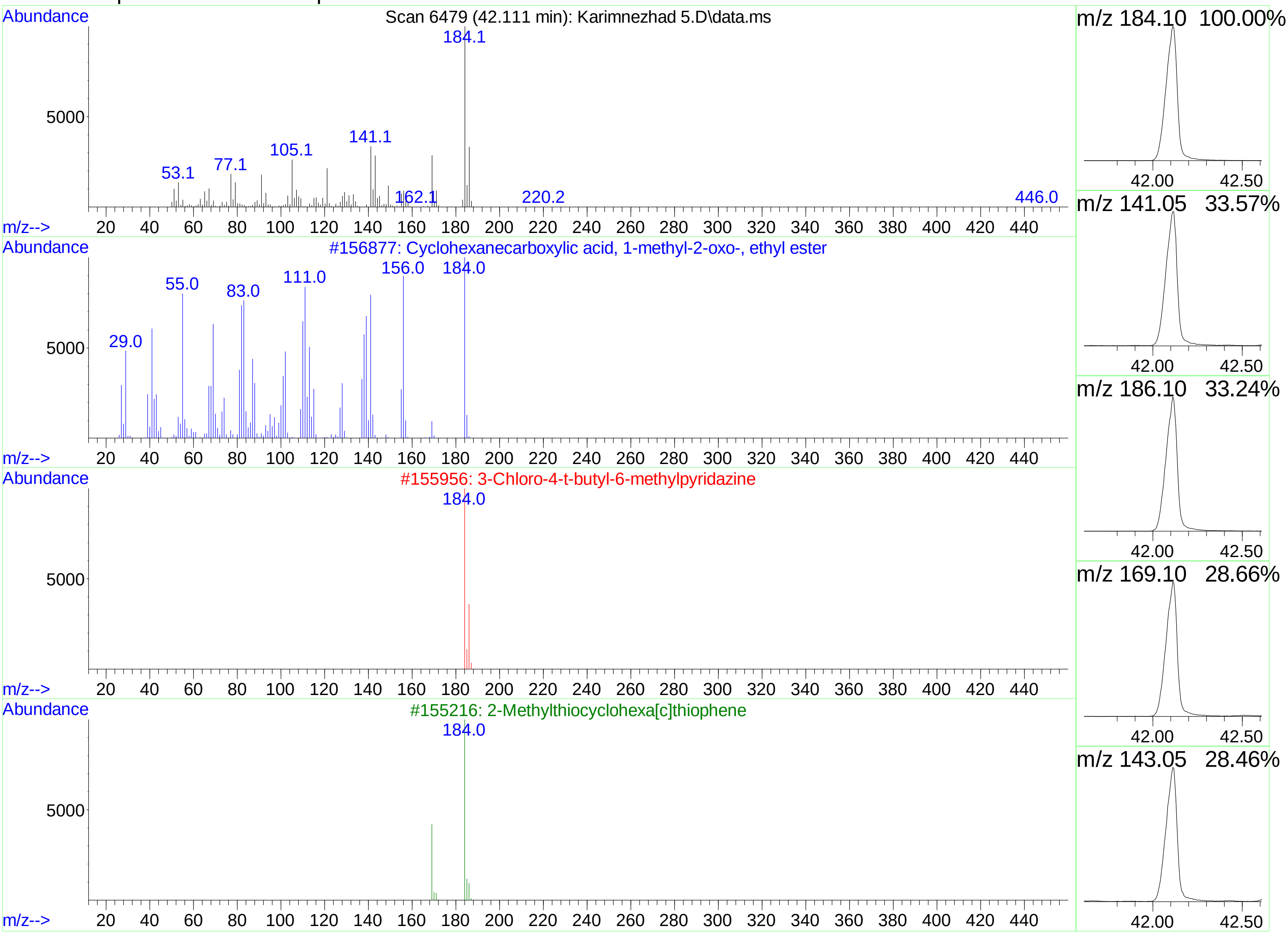


Data File: D:\msdchem\1\data\Karimnezhad 5.D
Sample : M15

Peak Number: 49 at 42.111 min Area: 152225979 Area % 0.75

| The 3 best hits from each library. | Ref\# | CAS\# | Qual |
|---|---|---|---|
| D:\Database\W10N14.L | | | |
| 1 Cyclohexanecarboxylic acid, 1-me... | 156877 | 005453-94-1 | 83 |
| 2 3-Chloro-4-t-butyl-6-methylpyrid... | 155956 | 2000155-95-6 | 83 |
| 3 2-Methylthiocyclohexa[c]thiophene | 155216 | 000000-00-0 | 64 |

Unknown Spectrum based on Apex

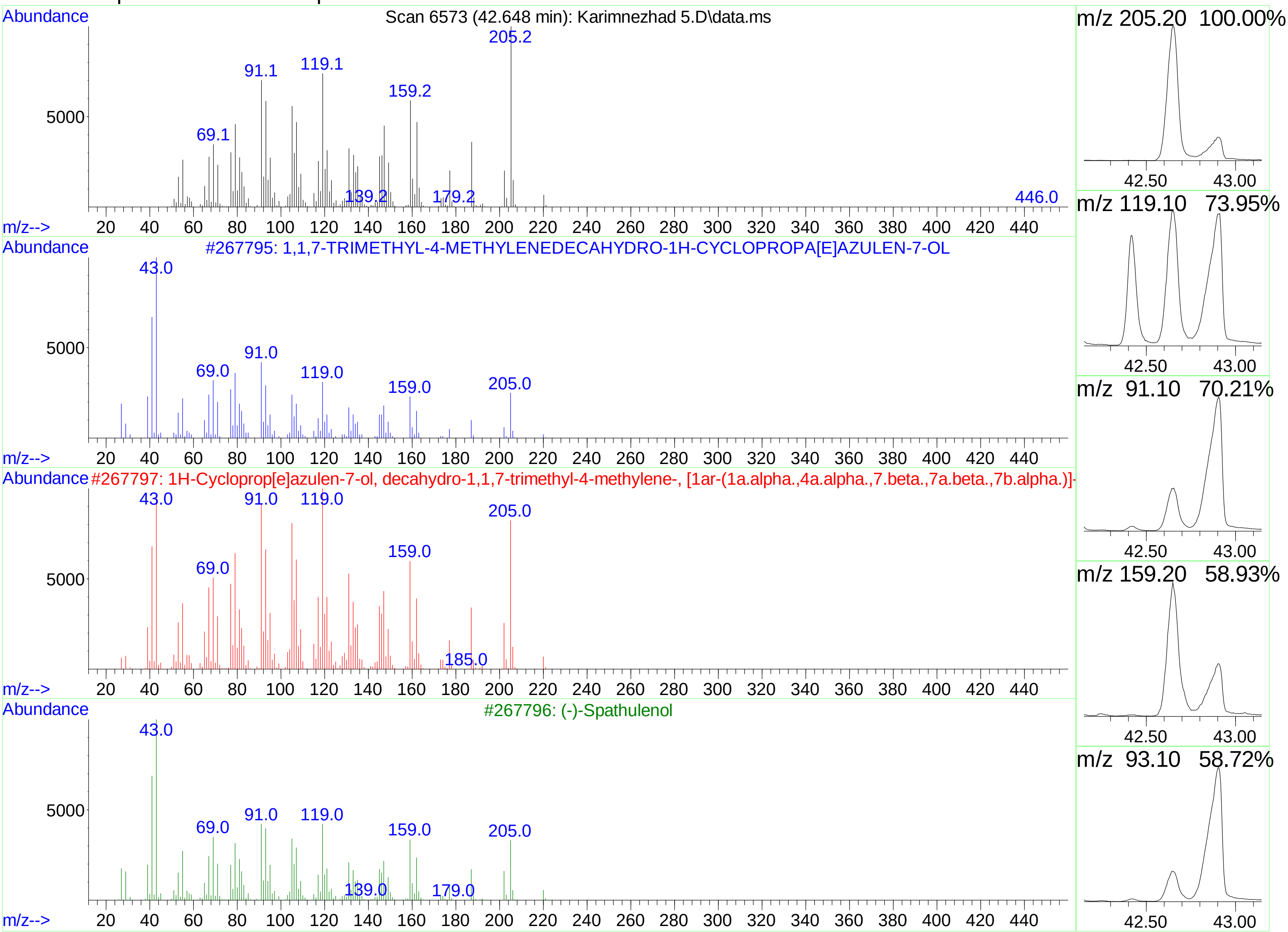


Data File: D:\msdchem\1\data\Karimnezhad 5.D
Sample : M15

Peak Number: 50 at 42.648 min Area: 171918069 Area % 0.85

| The 3 best hits from each library. | Ref\# | CAS\# | Qual |
|---|---|---|---|
| D:\Database\W10N14.L | | | |
| 1 1,1,7-TRIMETHYL-4-METHYLENEDECAH... | 267795 | 077171-55-2 | 98 |
| 2 1H-Cycloprop[e]azulen-7-ol, deca... | 267797 | 006750-60-3 | 98 |
| 3 (-)-Spathulenol | 267796 | 077171-55-2 | 95 |

Unknown Spectrum based on Apex

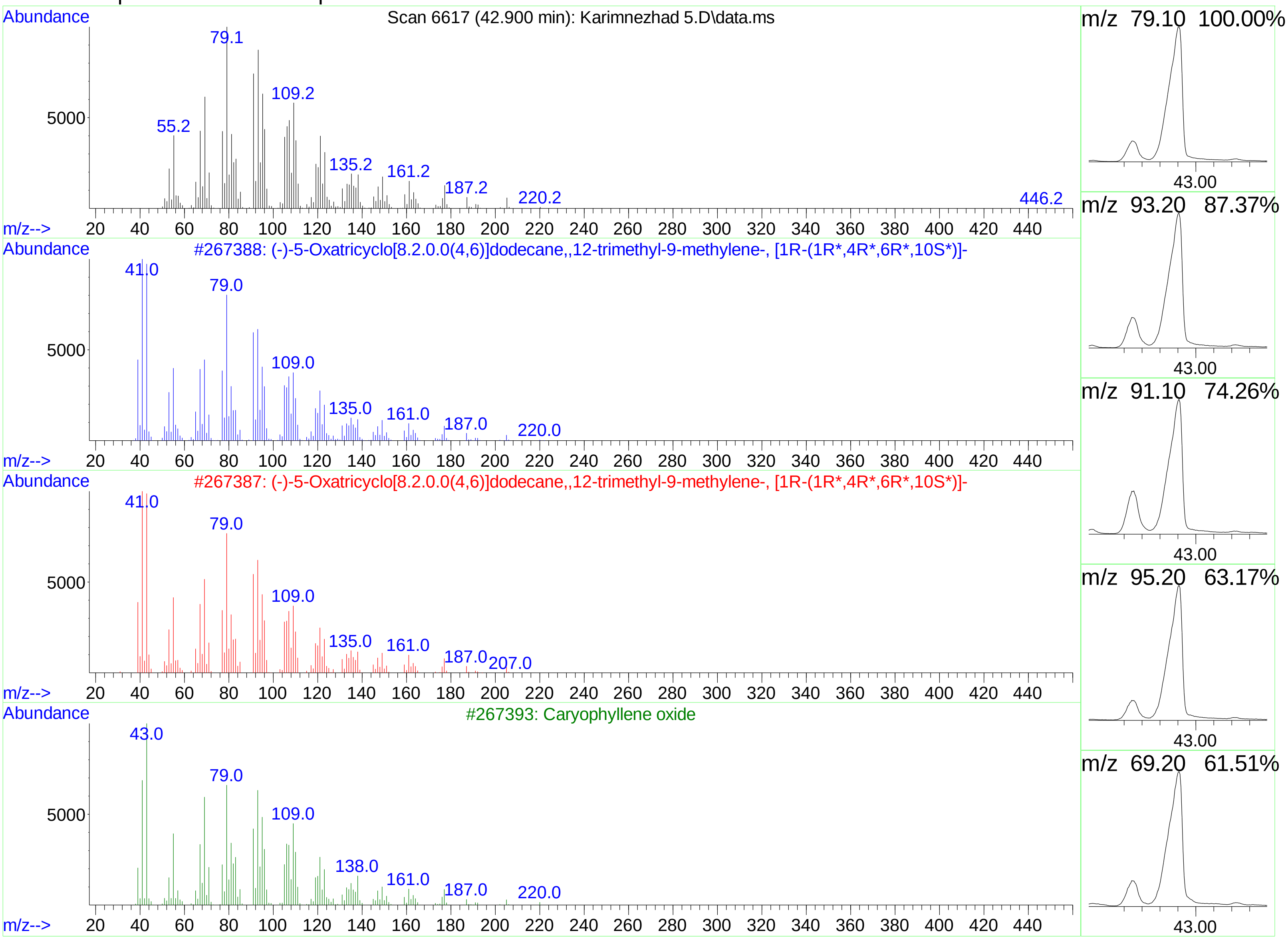


Data File: D:\msdchem\1\data\Karimnezhad 5.D
Sample : M15

Peak Number: 51 at 42.900 min Area: 605042002 Area % 2.99

| The 3 best hits from each library. | Ref\# | CAS\# | Qual |
|---|---|---|---|
| D:\Database\W10N14.L | | | |
| 1 (-)-5-Oxatricyclo[8.2.0.0(4,6)]d... | 267388 | 001139-30-6 | 99 |
| 2 (-)-5-Oxatricyclo[8.2.0.0(4,6)]d... | 267387 | 001139-30-6 | 95 |
| 3 Caryophyllene oxide | 267393 | 001139-30-6 | 94 |

Unknown Spectrum based on Apex

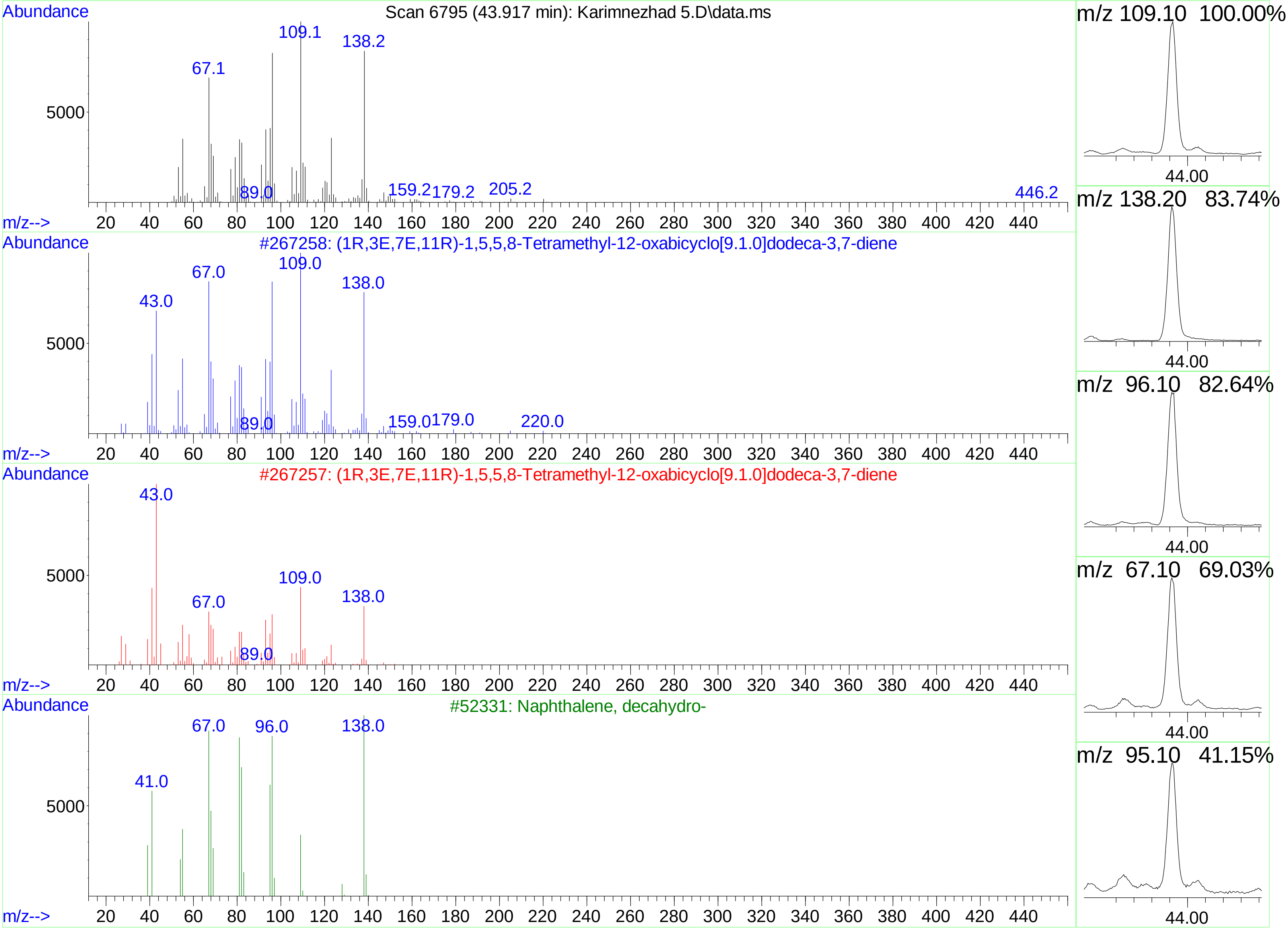


Data File: D:\msdchem\1\data\Karimnezhad 5.D
Sample : M15

Peak Number: 52 at 43.917 min Area: 47738006 Area % 0.24

The 3 best hits from each library. Ref\# CAS\# Qual

------------------------------------------------------------------

D:\Database\W10N14.L

| | | Ref# | CAS# | Qual |
|---|---|---|---|---|
| 1 | (1R,3E,7E,11R)-1,5,5,8-Tetrameth... | 267258 | 019888-34-7 | 99 |
| 2 | (1R,3E,7E,11R)-1,5,5,8-Tetrameth... | 267257 | 019888-34-7 | 87 |
| 3 | Naphthalene, decahydro- | 52331 | 000091-17-8 | 78 |

Unknown Spectrum based on Apex

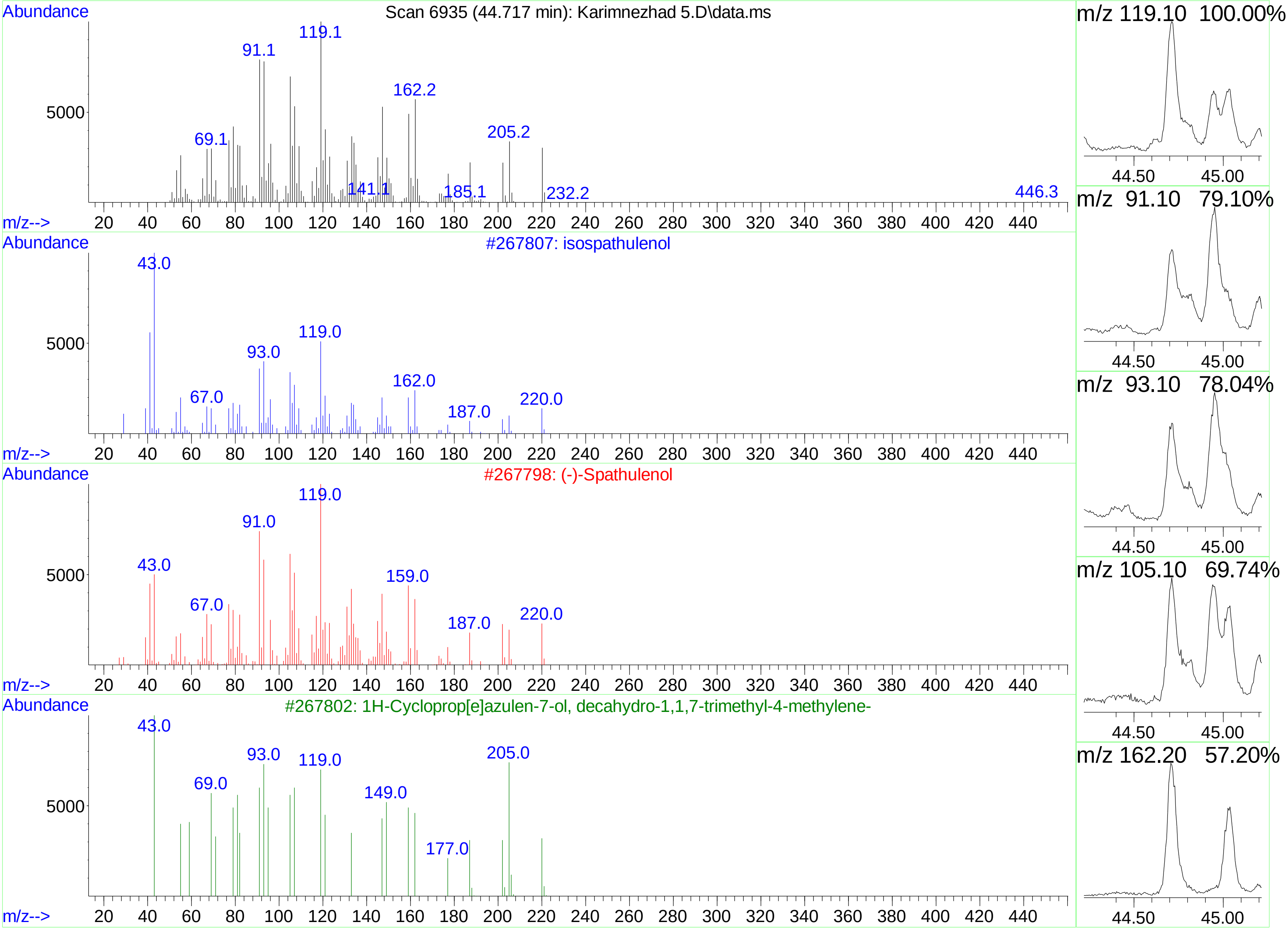


Data File: D:\msdchem\1\data\Karimnezhad 5.D
Sample : M15

Peak Number: 53 at 44.717 min Area: 40385101 Area % 0.20

| The 3 best hits from each library. | Ref# | CAS# | Qual |
|---|---|---|---|
| D:\Database\W10N14.L | | | |
| 1 isospathulenol | 267807 | 2000267-80-7 | 99 |
| 2 (-)-Spathulenol | 267798 | 077171-55-2 | 90 |
| 3 1H-Cycloprop[e]azulen-7-ol, deca... | 267802 | 006750-60-3 | 68 |

Unknown Spectrum based on Apex

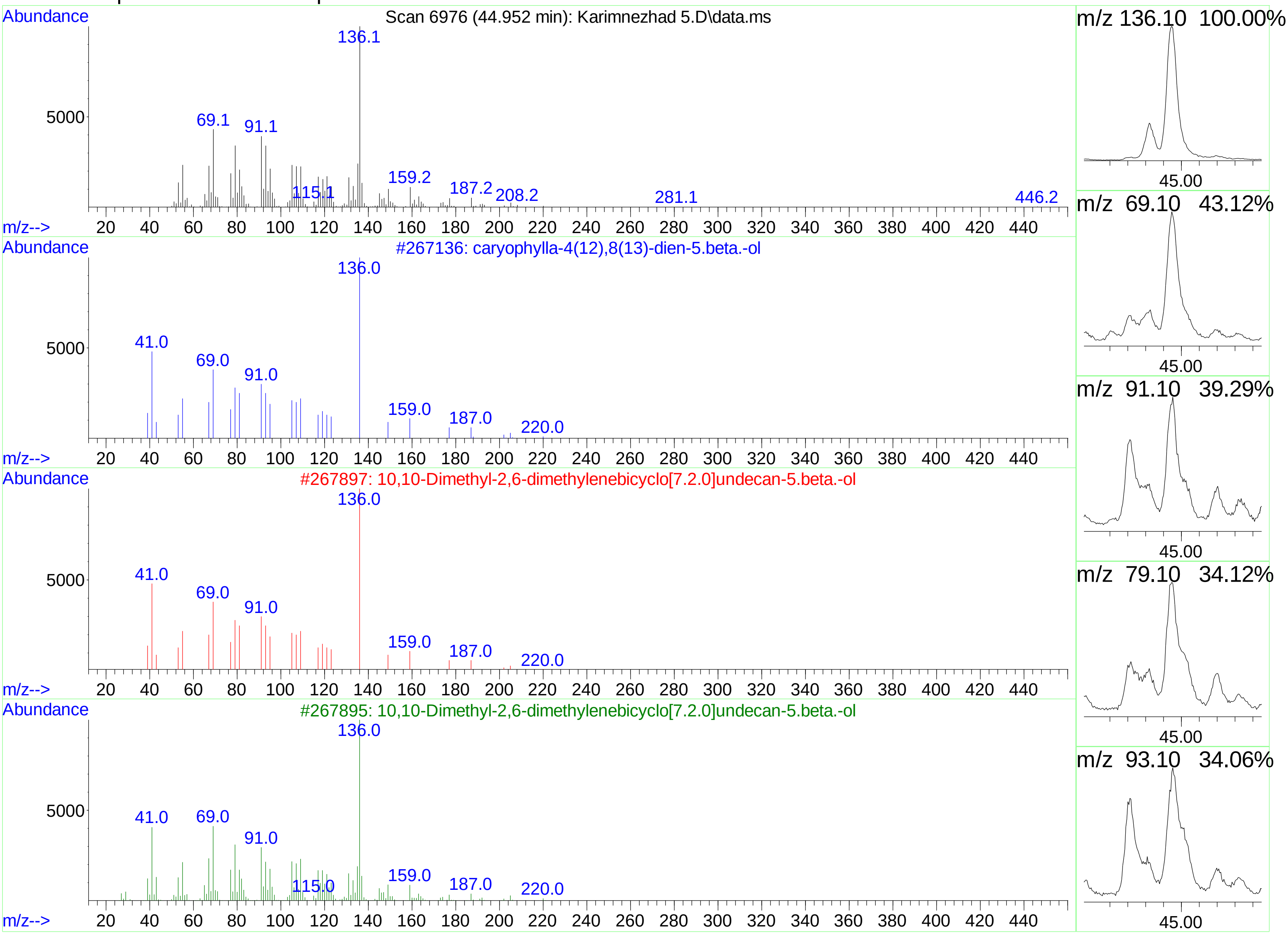


Data File: D:\msdchem\1\data\Karimnezhad 5.D
Sample : M15

Peak Number: 54 at 44.952 min Area: 58860708 Area % 0.29

| The 3 best hits from each library. | Ref# | CAS# | Qual |
|---|---|---|---|
| D:\Database\W10N14.L | | | |
| 1 caryophylla-4(12),8(13)-dien-5.b... | 267136 | 2000267-13-6 | 99 |
| 2 10,10-Dimethyl-2,6-dimethylenebi... | 267897 | 019431-80-2 | 99 |
| 3 10,10-Dimethyl-2,6-dimethylenebi... | 267895 | 019431-80-2 | 98 |

Unknown Spectrum based on Apex

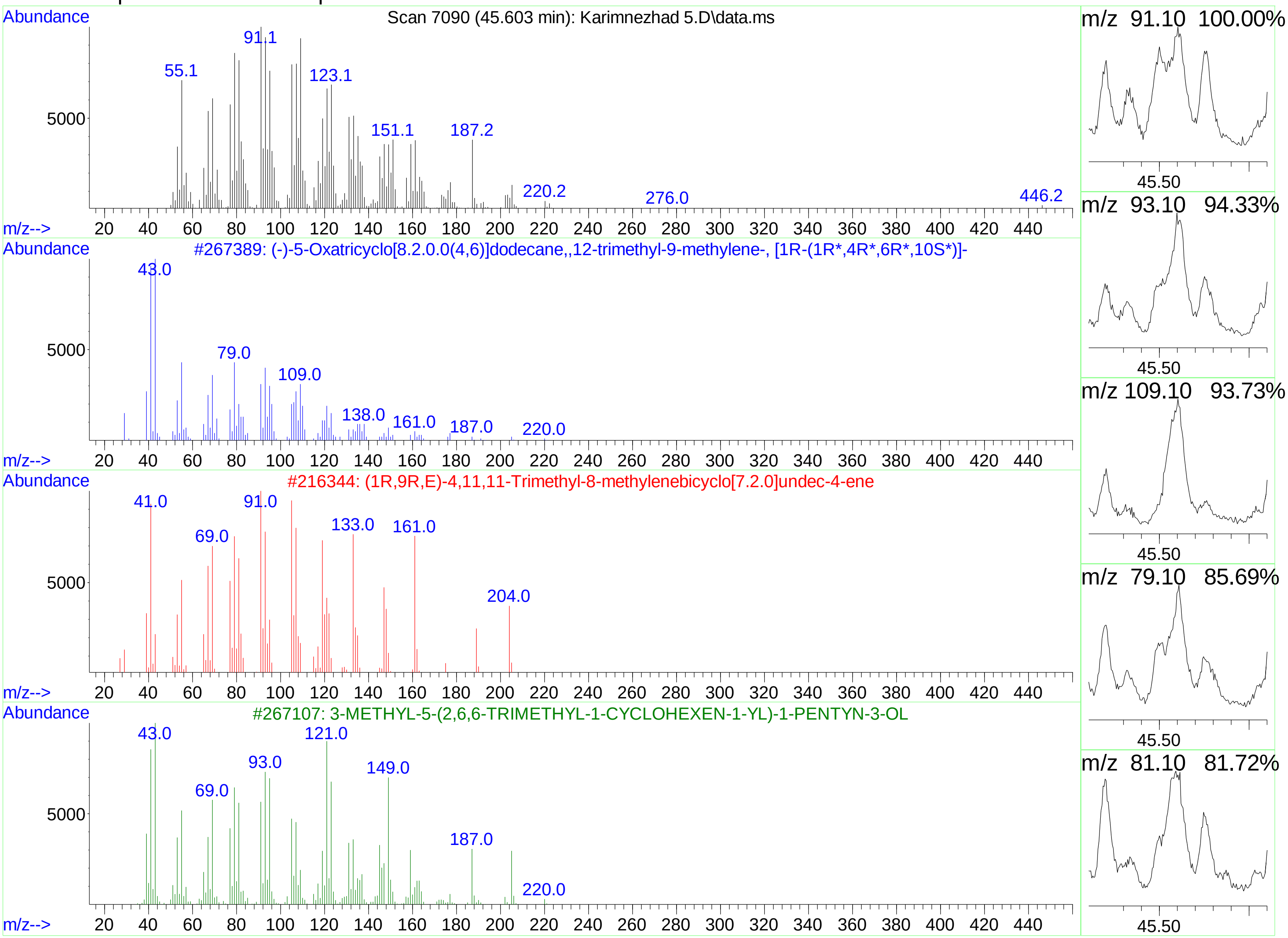


Data File: D:\msdchem\1\data\Karimnezhad 5.D
Sample : M15

Peak Number: 55 at 45.603 min Area: 35831195 Area % 0.18

The 3 best hits from each library. Ref\# CAS\# Qual

---

D:\Database\W10N14.L

| | | | | |
|---|---|---|---|---|
| 1 | (-)-5-Oxatricyclo[8.2.0.0(4,6)]d... | 267389 | 001139-30-6 | 78 |
| 2 | (1R,9R,E)-4,11,11-Trimethyl-8-me... | 216344 | 068832-35-9 | 70 |
| 3 | 3-METHYL-5-(2,6,6-TRIMETHYL-1-CY... | 267107 | 2000267-10-7 | 64 |

Unknown Spectrum based on Apex

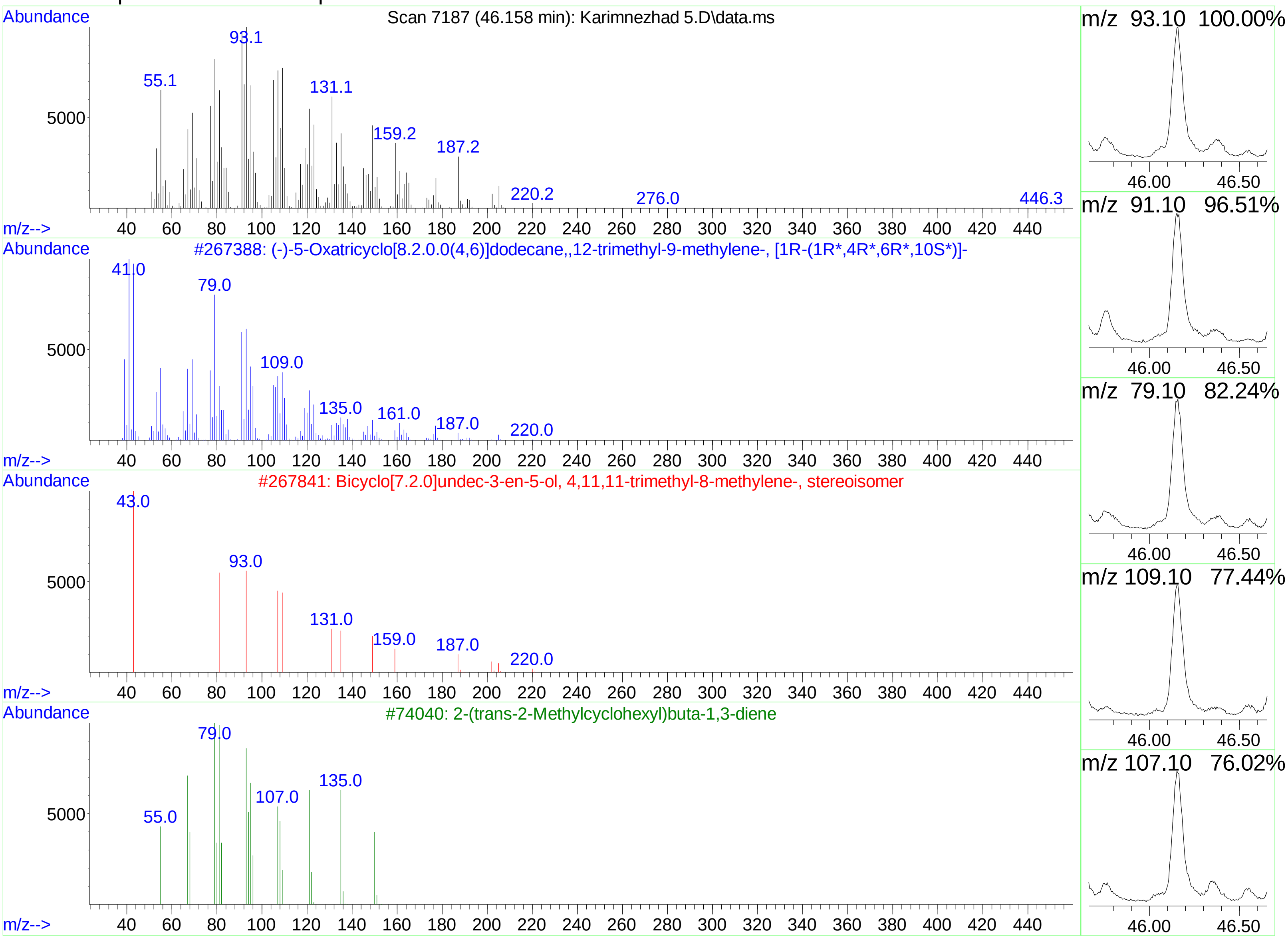


Data File: D:\msdchem\1\data\Karimnezhad 5.D
Sample : M15

Peak Number: 56 at 46.158 min Area: 62737181 Area % 0.31

| The 3 best hits from each library. | Ref\# | CAS\# | Qual |
|---|---|---|---|
| D:\Database\W10N14.L | | | |
| 1 (-)-5-Oxatricyclo[8.2.0.0(4,6)]d... | 267388 | 001139-30-6 | 68 |
| 2 Bicyclo[7.2.0]undec-3-en-5-ol, 4... | 267841 | 032214-89-4 | 60 |
| 3 2-(trans-2-Methylcyclohexyl)buta... | 74040 | 2000074-04-0 | 56 |

Unknown Spectrum based on Apex

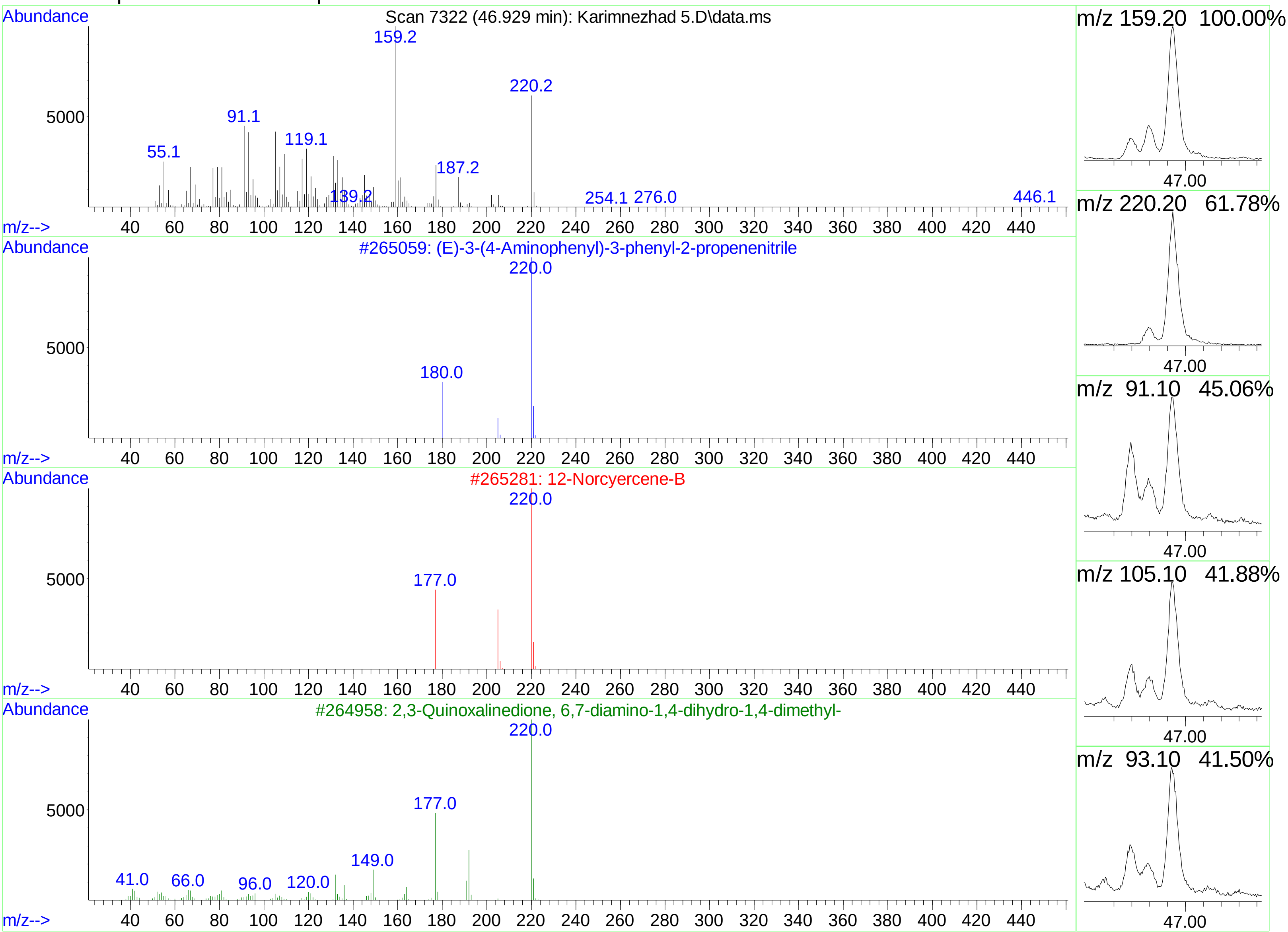


Data File: D:\msdchem\1\data\Karimnezhad 5.D
Sample : M15

Peak Number: 57 at 46.929 min Area: 30910996 Area % 0.15

| The 3 best hits from each library. | Ref# | CAS# | Qual |
|---|---|---|---|
| D:\Database\W10N14.L | | | |
| 1 (E)-3-(4-Aminophenyl)-3-phenyl-2... | 265059 | 2000265-05-9 | 90 |
| 2 12-Norcyercene-B | 265281 | 2000265-28-1 | 83 |
| 3 2,3-Quinoxalinedione, 6,7-diamin... | 264958 | 2000264-95-8 | 40 |

Unknown Spectrum based on Apex

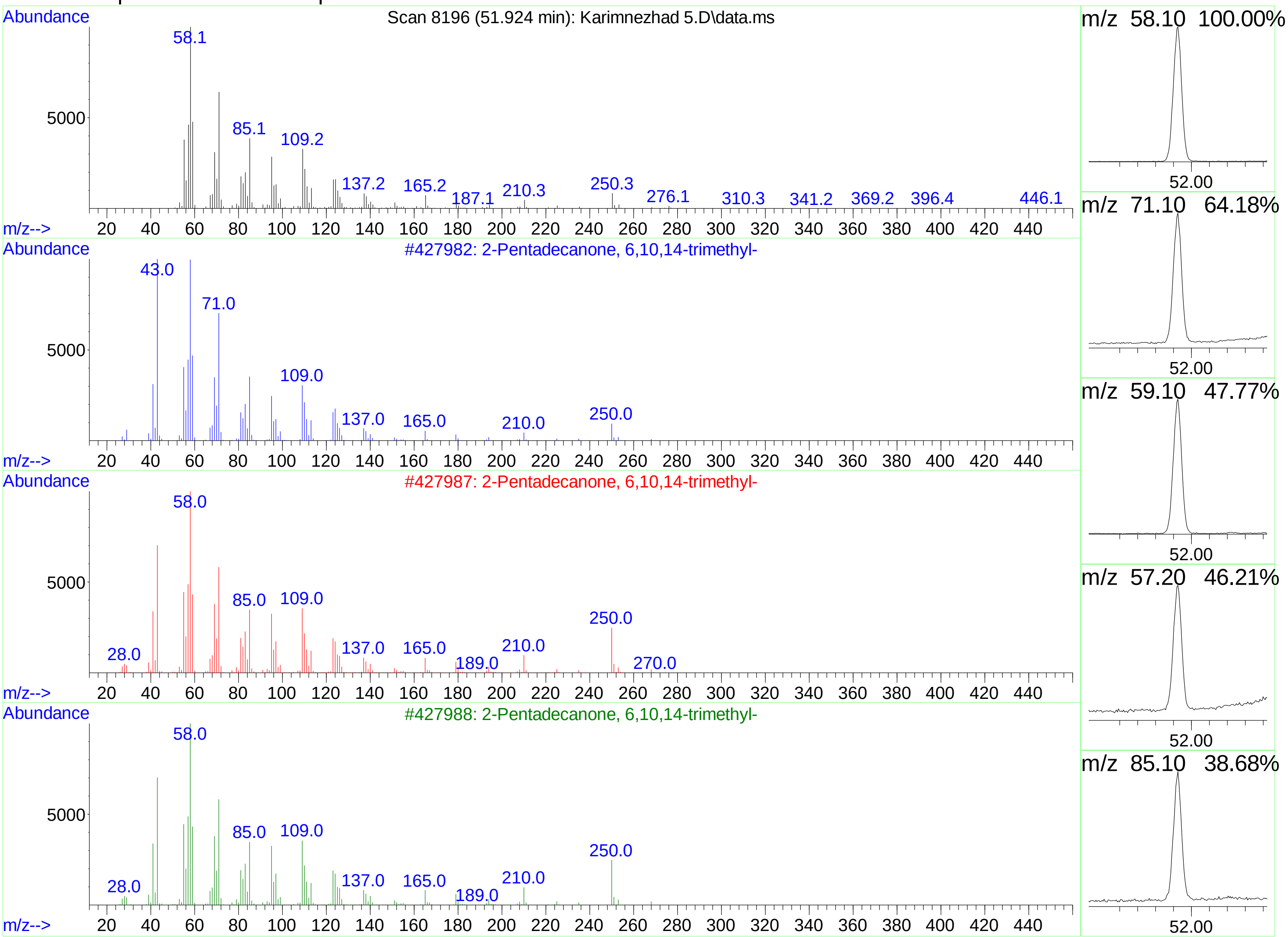


Data File: D:\msdchem\1\data\Karimnezhad 5.D
Sample : M15

Peak Number: 58 at 51.924 min Area: 35996796 Area % 0.18

The 3 best hits from each library. Ref\# CAS\# Qual

------------------------------------------------------------------

D:\Database\W10N14.L

| | | | | |
|---|---|---|---|---|
| 1 | 2-Pentadecanone, 6,10,14-trimethyl- | 427982 | 000502-69-2 | 99 |
| 2 | 2-Pentadecanone, 6,10,14-trimethyl- | 427987 | 000502-69-2 | 94 |
| 3 | 2-Pentadecanone, 6,10,14-trimethyl- | 427988 | 000502-69-2 | 94 |

Unknown Spectrum based on Apex

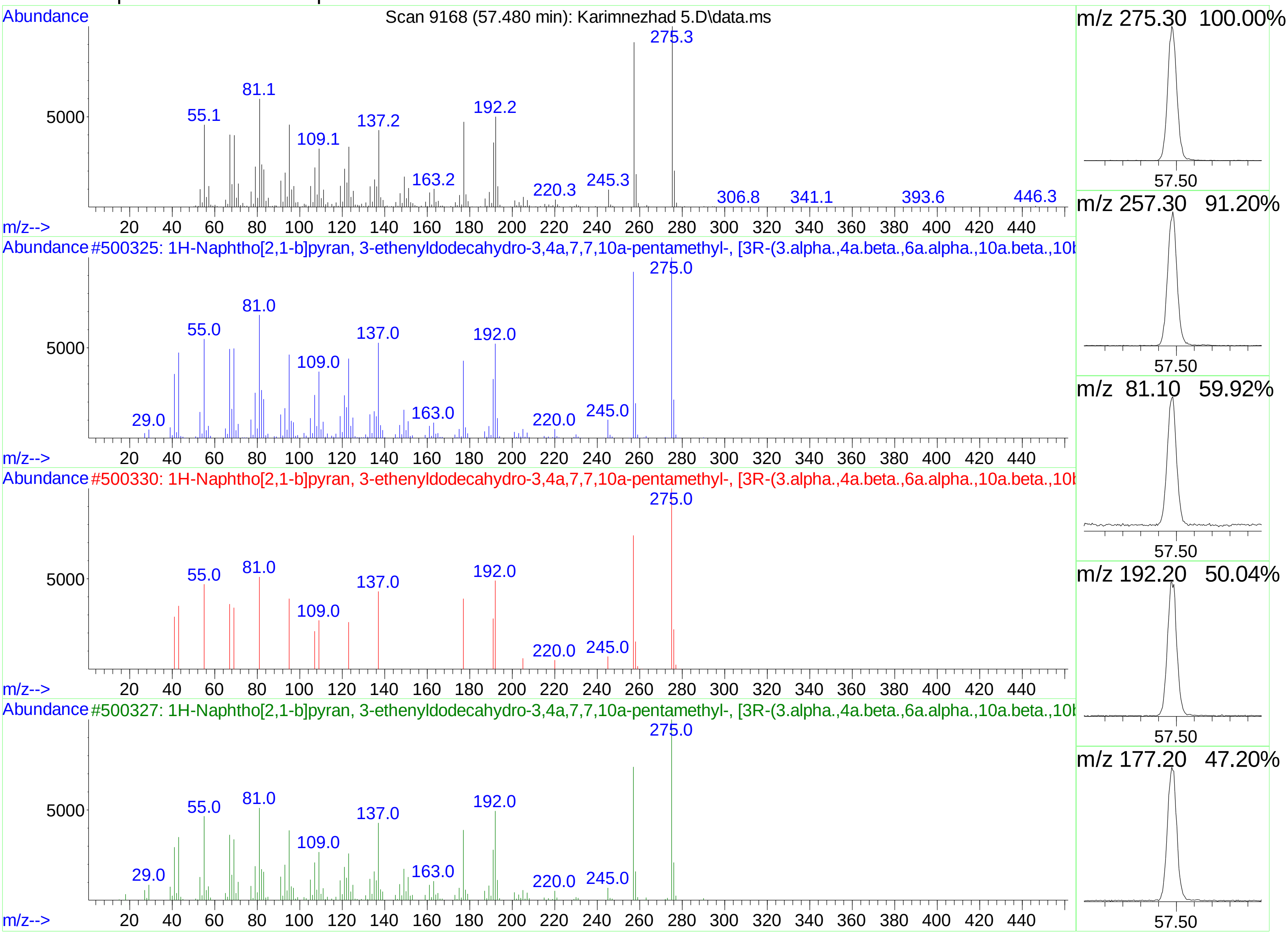


Data File: D:\msdchem\1\data\Karimnezhad 5.D
Sample : M15

Peak Number: 59 at 57.480 min Area: 41841834 Area % 0.21

| The 3 best hits from each library. | Ref# | CAS# | Qual |
|---|---|---|---|
| D:\Database\W10N14.L | | | |
| 1 1H-Naphtho[2,1-b]pyran, 3-etheny... | 500325 | 000596-84-9 | 99 |
| 2 1H-Naphtho[2,1-b]pyran, 3-etheny... | 500330 | 000596-84-9 | 95 |
| 3 1H-Naphtho[2,1-b]pyran, 3-etheny... | 500327 | 000596-84-9 | 91 |
